# Design, Synthesis, and Biological Activity of Isothiocyanate-Triazine Conjugates as AChE and BACE1 Inhibitors

**DOI:** 10.3390/biom16091280

**Published:** 2026-09-03

**Authors:** Kacper Górecki, Jakub Polakowski, Natalia Więckowska, Renata Grzywa, Justyna Frączyk, Beata Kolesińska, Agnieszka Wróbel-Tałałaj, Danuta Drozdowska, Łukasz Janczewski

**Affiliations:** 1 Institute of Organic Chemistry, Faculty of Chemistry, Lodz University of Technology, 116 Żeromskiego Str., 90-924 Łódź, Poland; kacper.gorecki@dokt.p.lodz.pl (K.G.); 243247@edu.p.lodz.pl (J.P.); 248428@edu.p.lodz.pl (N.W.); justyna.fraczyk@p.lodz.pl (J.F.); beata.kolesinska@p.lodz.pl (B.K.); 2 Department of Organic and Medicinal Chemistry, Faculty of Chemistry, Wrocław University of Science and Technology, Wybrzeże Wyspiańskiego 27, 50-370 Wrocław, Poland; renata.grzywa@pwr.edu.pl; 3 Department of Organic Chemistry, Medical University, 2a Mickiewicza Str., 15-222 Białystok, Poland; agnieszkawrobel9@gmail.com (A.W.-T.); danuta.drozdowska@umb.edu.pl (D.D.)

**Keywords:** isothiocyanates, 1,3,5-triazines, Alzheimer’s disease, acetylcholinesterase, β-secretase, isothiocyanate–triazine conjugates

## Abstract

The search for new compounds capable of inhibiting the activity of enzymes involved in the development of neurodegenerative diseases, including Alzheimer’s disease, has attracted continuing interest for many years. Natural isothiocyanates and their synthetic analogs, as well as compounds containing a 1,3,5-triazine core, constitute a group of molecules with complex mechanisms of action involving multiple molecular targets. Although literature data confirm the activity of both isothiocyanates and s-triazines, these two active fragments have not yet been combined, and it remains unclear whether a synergistic effect can be achieved. In this study, 11 novel isothiocyanate–triazine conjugates, consisting of a 1,3,5-triazine core substituted with aliphatic or cyclic secondary amines and a phosphorus analogue of isothiocyanate [6-(isothiocyanatohexyl)phosphonate)ethyl], were synthesized with yields of 46–80%. The pharmacokinetic profiles and parameters related to Lipinski’s rule of five were determined for all compounds. All compounds were evaluated as inhibitors of acetylcholinesterase (AChE) and β-secretase (BACE1). Compound **1i** showed the highest inhibitory activity against AChE (IC_50_ = 0.182 ± 0.02 µM), while compound **1b** exhibited the strongest inhibition of BACE1 (IC_50_ = 8.48 ± 2.18 µM). To explore the potential of these multifunctional derivatives, plausible enzyme-ligand binding models were proposed based on molecular docking simulations.

## 1. Introduction

Dementia, a decline in mental capacity resulting from changes in the brain, is an increasingly common condition among older people. According to data from the World Alzheimer’s Report 2024 [1], more than 55 million people worldwide currently suffer from dementia, and this number is steadily increasing. Forecasts suggest that by 2050 this number could reach as high as 139 million. The most common form of dementia is Alzheimer’s disease (AD), accounting for 60–80% of cases. AD is characterized by a decline in memory, thinking, and behavior that affects daily functioning. An estimated 6.7 million Americans aged 65 and older are living with AD today, and this number could rise to 13.8 million by 2060 without effective treatment [2]. Based on recent reports, there are approximately 250,000–300,000 people living in Poland with AD, and more than 500,000 people suffer from dementia [3].

Neurodegenerative diseases, often referred to as 21st-century diseases, are associated with progressive damage to neurons, the cells that constitute the nervous system. The development of AD is associated with the accumulation of abnormally folded proteins in the brain, including β-amyloid (Aβ) and tau protein, which form amyloid plaques and neurofibrillary tangles, respectively [4,5]. One of the best-known mechanisms leading to cognitive impairment in AD is the gradual loss of cholinergic neurons and a decrease in acetylcholine levels in the cerebral cortex and hippocampus. This deficit results from the toxic effects of Aβ oligomers, among other factors [6]. In response to these disturbances, drugs have been developed that inhibit acetylcholinesterase (AChE), an enzyme responsible for the hydrolysis of acetylcholine in the synaptic cleft. Inhibition of AChE leads to an increase in acetylcholine concentration in synapses and improved signal transmission in the central nervous system, which translates into an alleviation of cognitive dysfunction [7].

Due to their lipophilic nature, several ITCs can cross the blood–brain barrier, making them attractive candidates for the prevention and treatment of neurodegenerative disorders, including AD [8,9,10,11,12,13,14]. Isothiocyanates (ITCs) are naturally occurring low-molecular-weight compounds with the general formula R–N=C=S, where R represents an aliphatic, aromatic, or heterocyclic substituent. They occur mainly in cruciferous vegetables, including Brussels sprouts, wasabi, and horseradish, and are generated by myrosinase-catalyzed hydrolysis of glucosinolates [15,16,17]. Naturally occurring ITCs [18,19], such as benzyl isothiocyanate (BITC) [20], phenylethyl isothiocyanate (PEITC) [21,22], allyl isothiocyanate (AITC), sulforaphane (SFN) [23,24,25], erucin (ER), and 6-(methylsulfinyl)hexyl isothiocyanate (6-MSITC) [26], exhibit a broad spectrum of biological activities, including anticancer [27,28], antibacterial [29,30], antioxidant [31], and anti-inflammatory effects [32].

Their neuroprotective effects result from multiple complementary mechanisms. ITCs activate the Nrf2/ARE signaling pathway, thereby enhancing cellular antioxidant defenses, while suppression of NF-κB reduces neuroinflammation associated with disease progression. In addition, selected ITCs inhibit neuronal apoptosis, attenuate amyloid-β aggregation, modulate tau protein phosphorylation, and inhibit AChE, leading to increased acetylcholine levels and improved cholinergic neurotransmission [33,34].

The ability of ITCs to simultaneously target oxidative stress, neuroinflammation, protein aggregation, apoptosis, and cholinergic dysfunction highlights their potential as multifunctional agents for the management of AD. Burčul et al. [35] tested 11 aliphatic and aromatic ITCs as inhibitors of AChE and butyrylcholinesterase (BChE). They concluded that phenyl isothiocyanate and its derivatives showed the most promising inhibitory activity. 2-Methoxyphenyl ITC exhibited the strongest inhibition of AChE with an IC_50_ of 0.57 mM, while 3-methoxyphenyl ITC showed the highest inhibition of BChE (49.2% at 1.14 mM).

Triazines represent another class of compounds with a wide spectrum of pharmacological properties important for drug discovery research, including for the treatment of neurodegenerative diseases such as AD [36,37]. They exhibit antibacterial, antifungal, antiviral, anti-inflammatory, analgesic, anticancer, and antioxidant activities [38,39,40,41,42]. Particularly important in the context of AD are their neuroprotective properties. These properties are based on the modulation of inflammatory processes and the reduction in oxidative stress, which contributes to the protection of neurons from degeneration.

Numerous 1,3,5-triazine derivatives exhibit significant inhibitory activity against acetyl- and butyrylcholinesterase, and some have been designed as multi-target ligands combining cholinesterase inhibition with activity against β-secretase (BACE1), thereby interfering with the Aβ peptide formation pathway.

Zou et al. [43] synthesized a series of 2-phenylthiazole-1,3,5-triazine derivatives bearing various substituted secondary aromatic and aliphatic amines. Structure–activity relationship studies indicated that the incorporation of a thiomorpholine moiety was particularly favorable for cholinesterase inhibition, while molecular docking suggested that the most active derivatives were capable of interacting with both AChE and BChE. Further evidence for the therapeutic potential of 1,3,5-triazine derivatives was provided by Maliszewski et al. [44], who developed a series of nitrogen mustard analogs functionalized with dipeptide residues. Their study demonstrated that peptide-based modification of the triazine scaffold can yield multifunctional inhibitors capable of simultaneously targeting AChE and BACE1, highlighting this strategy as a promising approach for the design of multi-target-directed ligands. In a related study, Kułaga et al. [45] reported the synthesis of novel 1,3,5-triazine derivatives designed as BChE inhibitors. Their findings showed that combining the triazine core with nitrogen-containing substituents and fused heteroaromatic fragments, such as indole and benzothiophene, represents an effective strategy for the development of compounds with enhanced cholinesterase inhibitory properties.

**Figure 1:**
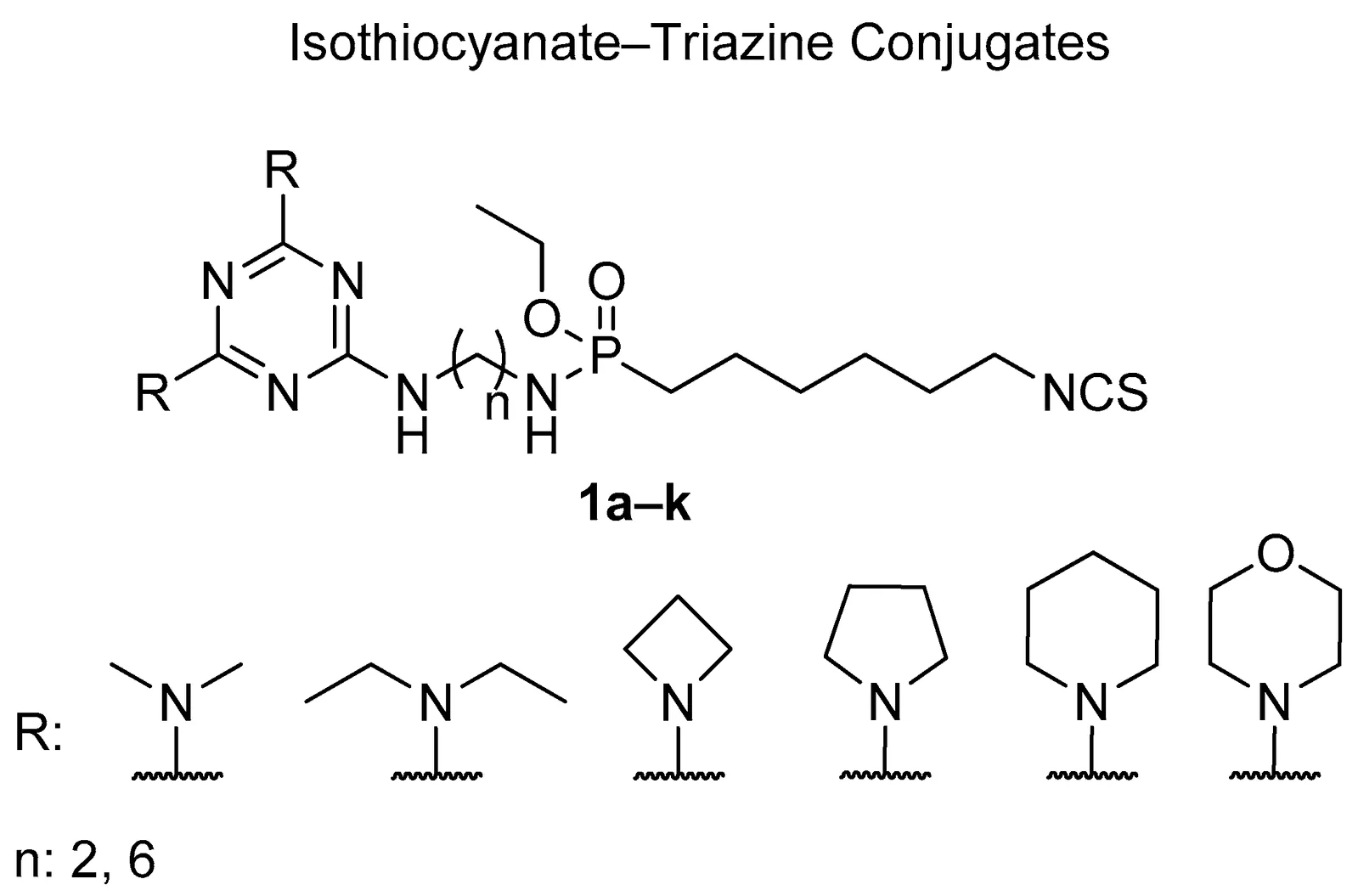
Structures of isothiocyanate–triazine conjugates.

The aim of this study was to design and synthesize novel isothiocyanate–triazine conjugates **1a**–**k** containing a substituted 1,3,5-triazine ring and diethyl 6-(isothiocyanatohexyl)phosphonate (phosphorus analog of isothiocyanate, P-ITC), and investigate their biological activity as inhibitors of AChE and BACE1 (Figure 1). The biological studies were conducted to determine whether the synthesized conjugates **1a**–**k** exhibit inhibitory activity against the aforementioned enzymes and whether a synergistic effect resulting from the combination of the 1,3,5-triazine ring and the P-ITC moiety can be observed.

The synthesized innovative isothiocyanate–triazine conjugates **1a**–**k** were based on a 1,3,5-triazine core substituted with identical or different amine groups, which are present in many drugs: dimethylamine, diethylamine, or cyclic amines including azetidine, pyrrolidine, piperidine, and morpholine. The third substituent, attached via an aliphatic linker (ethylenediamine or hexamethylenediamine), was a P-ITC, which has demonstrated anticancer properties both in vitro [46] and in vivo [47].

## 2. Materials and Methods

### 2.1. General Information

#### 2.1.1. Reagents

All reagents and solvents were purchased from Sigma-Aldrich (Poznań, Poland) and used as received.

#### 2.1.2. NMR Analysis

NMR spectra were recorded on a Bruker Avance II Plus 700 MHz spectrometer (Bruker Corporation, Billerica, MA, USA) at 700 MHz for ^1^H NMR, 176 MHz for ^13^C NMR, and 283 MHz for ^31^P NMR, and on a JEOL 400 MHz spectrometer (Tokyo, Japan) at 400 MHz for ^1^H NMR, 100.6 MHz for ^13^C NMR, and 161.8 MHz for ^31^P NMR, using CDCl_3_ as the solvent. ^1^H and ^13^C NMR spectra were referenced to the residual solvent peak based on literature data. Chemical shifts (δ) were reported in ppm and coupling constants (*J*) in Hz. ^13^C NMR spectra were recorded with proton decoupling.

#### 2.1.3. Microwave Conditions

A monomode microwave reactor (CEM Discover; CEM, Matthews, NC, USA) equipped with an IntelliVent pressure control system was used. The standard method was applied, and the maximum pressure was set to 250 psi. The temperature of the reaction mixtures was measured using an external infrared sensor.

#### 2.1.4. Flash Chromatography and TLC

Flash chromatography was performed using a glass column packed with Baker silica gel (30–60 μm). Thin-layer chromatography (TLC) was carried out on aluminum sheets coated with silica gel containing a 254 nm fluorescent indicator (Sigma-Aldrich, St. Louis, MO, USA).

#### 2.1.5. IR Analysis

IR spectra were measured on an FTIR Alpha Bruker (ATR) (Ettlingen, Germany) instrument and were reported in cm^−1^.

#### 2.1.6. Melting Points

Melting points were determined using a Cole-Parmer MP-400 Series apparatus (Chicago, IL, USA). Samples were heated at a rate of 5 °C/min.

#### 2.1.7. LC-MS Conditions

Liquid chromatography coupled with mass spectrometry (LC–MS) was used to analyze the compounds. A Vanquish Flex UHPLC system (Thermo Fisher Scientific, Waltham, MA, USA) and an ESI-Q-TOF Impact II mass spectrometer (Bruker) were used. Samples were diluted in a mixture of water and acetonitrile (1:1, *v*/*v*) with the addition of 0.1% formic acid. All reagents used for analysis were of LC–MS grade. The monoisotopic mass of the obtained products was confirmed by high-performance liquid chromatography coupled with mass spectrometry. Chromatographic separation was performed in a reversed-phase system using a Bruker Intensity Solo 2 column (100 × 2.1 mm, C18, 100 Å pore size) at 30 °C, with an injection volume of 1 μL and a flow rate of 0.3 mL/min. Gradient elution was carried out using water (component A) and acetonitrile (component B), both containing 0.1% formic acid, as follows: 0–2 min, 70/30 (A/B); 2–10 min, 50/50; 10–15 min, 0/100; 15–20 min, 0/100; 20–25 min, 70/30; 25–30 min, 70/30.

### 2.2. Synthesis of Monosubstituted 1,3,5-Triazines ***3a**–**f***

In a preheated 50 mL two-necked round-bottom flask equipped with a magnetic stir bar, a low-temperature thermometer, and a CaCl_2_ tube, cyanuric chloride (**2**) (0.92 g, 5 mmol) was dissolved in THF (15 mL) and DIPEA (0.9 mL, 5 mmol) was added dropwise. The reaction mixture was then cooled to –20 °C using a dry ice/acetone bath and either 2 M dimethylamine in THF (2.5 mL, 5 mmol) or diethylamine, azetidine, pyrrolidine, piperidine or morpholine (5 mmol) dissolved in THF (2 mL) was added. The reaction was carried out for 35 min. Then, after warming the reaction mixture to room temperature, the resulting precipitate was filtered under reduced pressure and the filtrate was evaporated under reduced pressure. The pure products **3a**–**f** were isolated by column chromatography using 100% DCM as the eluent with yields of 56–86% (**3a**: 85%; **3b**: 86%; **3c**: 62%; **3d**: 62%; **3e**: 66% and **3f**: 56%). The analytical data for compounds **3a**–**f** are provided in the Supplementary Materials S1.

### 2.3. Synthesis of Disubstituted 1,3,5-Triazines ***4a**–**j***

In a preheated 50 mL round-bottom flask equipped with a magnetic stir bar and a CaCl_2_ drying tube, compounds **3a**–**f** (1 mmol) were dissolved in THF (15 mL). NaHCO_3_ (1 mmol) was then added, followed by either a 2 M solution of dimethylamine in THF (10 mmol) or diethylamine, azetidine, pyrrolidine, piperidine, or morpholine (10 mmol) dissolved in THF (2 mL). The reaction mixture was stirred for 3 h at room temperature. After this time, the precipitate was filtered under reduced pressure, and the filtrate was concentrated using a rotary evaporator. The crude products were purified by column chromatography to afford compounds **4a**–**j** in yields of 41–94% (**4a**: 94%; **4b**: 85%; **4c**: 66%; **4d**: 43%; **4e**: 41%; **4f**: 84%; **4g**: 89%; **4h**: 59%; **4i**: 86%; **4j**: 51%). The analytical data for compounds **4a**–**j** are provided in the Supplementary Materials S2.

### 2.4. Synthesis of Trisubstituted 1,3,5-Triazines ***5a**–**j***

In a 50 mL round-bottom flask equipped with a reflux condenser and a CaCl_2_ tube, compounds **4a**–**j** (1 mmol) were dissolved in THF (10 mL). Potassium carbonate (0.208 g, 1.5 mmol) and ethylenediamine (1.6 mL, 25 mmol) were then added. The reaction was carried out for 6.5 h at 80 °C. After this time, the solvent was evaporated under reduced pressure. The residue was dissolved in water (20 mL) and extracted with chloroform (3 × 20 mL). The combined organic layers were washed with water (10 mL), then dried over anhydrous MgSO_4_. The solvent was evaporated under reduced pressure, yielding products **5a**–**j** in 67–99% yields (**5a**: 67%; **5b**: 82%; **5c**: >99%; **5d**: 95%; **5e**: 99%; **5f**: 85%; **5g**: 76%; **5h**: 90%; **5i**: 76%; **5j**: 88%). The analytical data for compounds **5a**–**j** and **5k** are provided in the Supplementary Materials S3.

### 2.5. Synthesis of Trisubstituted 1,3,5-Triazines ***5k***

In a preheated high-pressure vial (10 mL) equipped with a magnetic stir bar and a silicone cap with a Teflon insert, disubstituted 1,3,5-triazine **4a** (0.1 g, 0.5 mmol) was dissolved in THF (3 mL). Potassium carbonate (0.103 g, 1.5 mmol) and hexamethylenediamine (0.116 g, 1 mmol) were then added. The reaction was carried out in a microwave reactor at 100 °C for 60 min. After this time, the solvent was evaporated under reduced pressure. The residue was dissolved in water (5 mL) and extracted with chloroform (3 × 10 mL). The combined organic layers were washed with water (5 mL), then dried over anhydrous MgSO_4_. The solvent was evaporated under reduced pressure. Product **5k** was isolated with a yield of 86% (0.120 g). The analytical data for compound **5k** are provided in the Supplementary Materials S3.11.

### 2.6. Synthesis of 6-(Azidohexyl)phosphonate Diethyl (***6***)

In a preheated high-pressure vial (10 mL) equipped with a magnetic stir bar and a silicone cap with a Teflon insert, diethyl 6-(bromohexyl)phosphonate (0.602 g, 2 mmol) and sodium azide (0.331 g, 5.1 mmol) were suspended in water (3 mL). The reaction was carried out in a microwave reactor under the following conditions: dynamic mode, 130 °C, 150 W, 15 min, 250 psi. The contents of the vial were extracted with diethyl ether (2 × 10 mL), and the combined organic layers were washed with water (2 mL) and brine (2 mL), then dried over anhydrous magnesium sulfate. The drying agent was filtered off, and the solvent was evaporated under reduced pressure to give pure product **6** (0.490 g, 93%). The analytical data for compound **6** are provided in the Supplementary Materials S4.

### 2.7. Synthesis of Ethyl 6-(Azidohexyl)phosphonate Monoester (***7***)

In a preheated 10 mL high-pressure vial equipped with a magnetic stir bar and a silicone cap with a Teflon insert, diethyl 6-(azidohexyl)phosphonate (**6**) (0.263 g, 1 mmol) and lithium bromide (0.104 g, 1.2 mmol) were dissolved in acetone (3 mL). The reaction was carried out in a microwave reactor under the following conditions: dynamic mode, 110 °C, 150 W, 60 min, 250 psi. After this time, the solvent was evaporated under reduced pressure. The residue was dissolved in water (15 mL) and extracted with diethyl ether (3 × 10 mL). The aqueous layer was then acidified with concentrated HCl to pH 1 and extracted with dichloromethane (3 × 20 mL). The combined organic layers were dried over anhydrous MgSO_4_, and the solvent was evaporated under reduced pressure to give ethyl monoester **7** with a yield of 77% (0.181 g). The analytical data for compound **7** are provided in the Supplementary Materials S5.

### 2.8. Synthesis of Compounds ***8a**–**k***

In a 25 mL round-bottom flask equipped with a magnetic stir bar and a CaCl_2_ tube, ethyl monoester **7** (1 mmol) was dissolved in DCM (5 mL), and oxalyl chloride (0.34 mL, 4 mmol) and a catalytic amount of DMF (5 drops) were added dropwise. The reaction was carried out at room temperature for 1 h. A reflux condenser was then attached, and stirring was continued at 40 °C for 30 min. After this time, the solvent was evaporated under reduced pressure and co-evaporated with dichloromethane (2 × 5 mL). The residue was dissolved in DCM (5 mL) and cooled in an ice bath with stirring. In a separate flask, Et_3_N (0.42 mL, 3 mmol), triazines **5a**–**k** (1 mmol), and a catalytic amount of DMAP (5 mg) were dissolved in DCM (5 mL). This solution was added dropwise to the cooled reaction mixture. After 20 min, the ice bath was removed, and stirring was continued overnight at room temperature. The reaction mixture was then diluted with dichloromethane (50 mL) and washed successively with water (5 mL), 1 M NaOH (2 × 5 mL), and water (5 mL). The organic layer was dried over anhydrous MgSO_4_, and the solvent was evaporated under reduced pressure. The pure products **8a**–**k** were isolated by column chromatography in 41–77% yields (**8a**: 74%; **8b**: 57%; **8c**: 41%; **8d**: 52%; **8e**: 44%; **8f**: 77%; **8g**: 43%; **8h**: 48%; **8i**: 44%; **8j**: 46%; **8k**: 60%). The analytical data for compounds **8a**–**k** are provided in the Supplementary Materials S6.

### 2.9. Synthesis of Isothiocyanate–Triazine Conjugates ***1a**–**k***

In a 25 mL round-bottom flask equipped with a magnetic stir bar, compounds **8a**–**k** (1 mmol) were dissolved in toluene (5 mL) and cooled in an ice bath. Triphenylphosphine (0.314 g, 1.2 mmol) was then added, and the reaction mixture was stirred at room temperature for 3 h. After this time, carbon disulfide (0.48 mL, 8 mmol) was added dropwise, and stirring was continued for 72 h. The solvent was then evaporated under reduced pressure, and the final isothiocyanate–triazine conjugates **1a**–**k** were isolated by column chromatography in 46–80% yields (**1a**: 70%; **1b**: 58%; **1c**: 63%; **1d**: 54%; **1e**: 72%; **1f**: 46%; **1g**: 56%; **1h**: 48%; **1i**: 67%; **1j**: 66%; **1k**: 80%). Purity was determined by ^1^H, ^31^P and 13C NMR spectroscopy and based on UV-Vis chromatograms. The analytical data for compounds **1a**–**k** are provided in the Supplementary Materials S7.

### 2.10. In Vitro Inhibition Studies on Acetylcholinesterase (eeAChE)

The inhibitory activity of the target compounds against AChE from *Electrophorus electricus* (electric eel) (purified AChE—catalog number C3389) was assessed using a previously adapted Ellman’s spectrophotometric method with using a kit for testing AChE inhibitors (catalog number MAK324) (Sigma-Aldrich (St. Louis, MO, USA) [44]. The compounds were dissolved in DMSO and diluted using 0.1 M KH_2_PO_4_/K_2_HPO_4_ phosphate buffer (pH 7.5) at room temperature to yield the corresponding test concentrations 1 mM and 100 mM. Measurements were performed using a clear 96-well flat-bottom plate. The absorbance was read on an Infinite M200 fluorescence spectrophotometer (TECAN, Männedorf, Switzerland) (ex. 412 nm). All samples and controls were assayed in duplicate. There were two control wells: a no-enzyme well and a no-inhibitor control well containing the AChE Reference Enzyme. For each well, a reaction mixture was prepared by mixing into a clean tube 154 μL of Assay Buffer, 1 μL of Substrate (100 mM), and 0.5 μL of 5,5′-dithiobis(2-nitrobenzoic acid) (DTNB). The reaction was initiated by adding 45 μL of assay buffer, 5 μL of the enzyme and 5 μL of the test compound to the wells, giving final concentrations of 1, 10, 20, 50, and 100 µM. The plate was incubated for 15 min. Then, 150 μL of the reaction mixture was added to each well containing the compounds, as well as to the control (No Enzyme) and the No Inhibitor Control wells. After mixing, the absorbance was measured at 412 nm at 0 min and at 10 min. AChE inhibition was calculated as a percentage. To obtain accurate IC_50_ values, this test was repeated three times. An AChE inhibitor screening kit (Sigma-Aldrich) was used.

### 2.11. In Vitro Inhibition Studies on β-Secretase (esBACE1)

The inhibition of human BACE1 was assessed using a fluorescence resonance energy transfer (FRET) assay, according to a previously described protocol using a kit from Sigma-Aldrich (St. Louis, MO, USA) (catalog number CS0010) [44]. An increase in fluorescence intensity was observed after substrate cleavage by BACE1. A 96-well flat-bottomed plate was used for the fluorescence assay. The new compounds, prepared in assay buffer at concentrations of 1, 10, 20, 50, and 100 µM, were placed in separate wells of a black 96-well microplate together with 20 μL of BACE1 substrate and mixed. All samples, the negative control (no enzyme), and the positive control (enzyme activity provided) were assayed in duplicate. Immediately before measurement, 10 μL of BACE1 enzyme solution (diluted 10-fold with fluorescence assay buffer) was added. Fluorescence was measured immediately after enzyme addition (‘time zero’), and after incubation (37 °C; 2 h) on an Infinite M200 fluorescence spectrophotometer (TECAN, Männedorf, Switzerland) (ex. 320 nm; em. 405 nm). This test was repeated three times. BACE1 activity was calculated as percentage inhibition and reported as IC_50_ (μM). A BACE1 activity detection kit (Sigma-Aldrich) was used.

### 2.12. Statistical Analysis

The half-maximal inhibitory concentration IC_50_ values were determined from dose–response curves using SlideWrite Plus software Version 7.0, Advanced Graphics Software, Inc. (Trial Version, Advanced Graphics Software, Inc., Carlsbad, CA, USA). Non-linear regression analysis was applied to fit the experimental data points (percentage of inhibition vs. logarithmic concentration of the tested compound). The IC_50_ value was calculated as the concentration required to achieve 50% inhibition of the targeted activity relative to the negative control. Detailed fitting parameters and graphical representations of the dose–response curves are provided in the Supplementary Material.

### 2.13. In Silico Structural Profiling and Molecular Docking In Vitro

Based on the induced-fit mechanism characteristic of AChE and BACE1 upon substrate/ligand binding, crystal structures of both enzymes co-crystallized with ligands were selected for docking simulations. Structures of human beta-secretase 1 in complex with a hydroxyethylamine-based inhibitor (PDB ID: 3UQW) [48] and human acetylcholinesterase in complex with donepezil (PDB ID: 4EY7) [49] were prepared for docking by extracting the coordinates for a single protein chain (excluding ligands and water molecules) and adding hydrogen atoms. Human AChE and human BACE1 co-crystal structures bound to inhibitors were selected as docking templates to represent the target human enzymes and account for the induced-fit conformational states of their active sites. Although preliminary in vitro enzyme inhibition assays were conducted using *Electrophorus electricus* AChE as an established, cost-effective screening standard, human structural models were intentionally employed for docking to align with the ultimate goal of designing human-targeted ligands. This approach is further supported by the high evolutionary conservation of these enzymes, with sequence identity reaching 60.4% between human (UniProt ID: P22303) and electric eel (UniProt ID: O42275) AChE (and nearly 90% identity across the catalytic and binding site area), as well as 96.5% sequence identity between human (UniProt ID: P56817) and horse (Uniprot ID: A0A9L0SS89) BACE1.

Initial 3D structures of ligands **1a**–**k** and quercetin were subjected to conformational search and energy minimization using the RDConf tool (part of the chemistry toolkit RDKit Version 2020.03.4) [50,51] via the Galaxy platform (www.usegalaxy.org, Galaxy Version 2020.03.4 + galaxy0) [52]. The lowest-energy conformers, generated with the ETKDG algorithm and optimized with the MMFF force field, were selected as input geometries for docking simulations [53]. For all structures, protonation states and atom types were assigned using SPORES v. 1.28 [54].

Docking calculations were performed using The Protein–Ligand ANT System (PLANTS v. 1.2, Eberhard Karls Universität Tübingen, Tübingen, Germany) with ChemPLP scoring function [55,56,57]. The binding site was defined as a sphere centered on the central atom of the co-crystallized ligand in each enzyme complex. The docking procedure was validated by re-docking the co-crystallized ligands into their respective target enzymes. The re-docking protocol was executed using a binding site radius of 20 Å. For the hBACE1 inhibitor, comparison of the top-scoring docking pose with the crystallographic coordinates showed an RMSD value of 1.613 Å. For the re-docking of donepezil into the hAChE structure, the resulting RMSD value was 0.974 Å (calculated for heavy atoms, visualizations are provided in the Supplementary Materials). To explore potential binding sites, an initial blind docking was performed across the entire protein structure using search radii of 70 Å for hAChE and 50 Å for hBACE1. For both enzymes, this preliminary screening revealed that the vast majority of generated poses preferentially bound within the extended cavity surrounding the catalytic active site. Subsequent focused docking calculations were carried out using a refined radius of 20 Å with an unchanged binding site center for both enzymes. Blind docking simulations were performed in triplicate for each compound, whereas focused docking protocols and re-docking validations were executed using four independent runs. For each run, a total of 10 poses were generated. No random seed was specified manually. Donepezil (used for re-docking) and quercetin were included in docking simulations as reference control compounds. Low-molecular-weight reference inhibitors (altretamine, PEITC, and P-ITC) were excluded due to significant molecular weight discrepancies and their lack of multifunctional structural features, which were present in the tested compounds (**1a**–**k**).

Optimal docking poses for compounds **1a**–**k** were selected based on a consensus strategy: (1) visual screening of common binding orientations, (2) selection of the most populated cluster defined by core scaffold alignment (triazine ring with exogenous nitrogen atoms, RMSD ≤ 3 Å), and (3) final selection of the top-scoring pose within the dominant cluster based on its spatially consistent conformational profile. For reference control compounds, re-docking was validated using the co-crystallized structures, while quercetin was docked into hBACE1 using a two-step blind and focused docking approach. The final binding pose of quercetin was selected based on the docking score from the most populated cluster observed within the active site cavity. Protein–ligand interaction analyses for all selected poses were performed using the Protein–Ligand Interaction Profiler (PLIP v. 3.0.0) [58] Python v. 3.10.6package (visualizations and interaction tables are provided in the Supplementary Materials). Throughout the text, hydrogen bond distances are reported as heavy atom donor-acceptor (D···A) distances, whereas graphical representations and Supplementary Data provide hydrogen-acceptor (H···A) values. Amino acid residues were numbered according to the reference crystal structures.

### 2.14. ADMET-AI

Theoretical ADMET properties were calculated using ADMET-AI [59,60] (accessed on 15 January 2026) and are given in the Supplementary Materials.

## 3. Results

### 3.1. Chemistry

The first step in the preparation of the target compounds **1a**–**k** was the synthesis of trisubstituted 1,3,5-triazines **5a**–**k** with attached secondary aliphatic or cyclic amines and an unbranched linker containing two or six carbon atoms. The method of selective attachment of various nucleophiles to the triazine ring developed by our team allows the preparation of derivatives containing two or three different or identical substituents, depending on the temperature and the base used [61,62].

**Figure 2:**
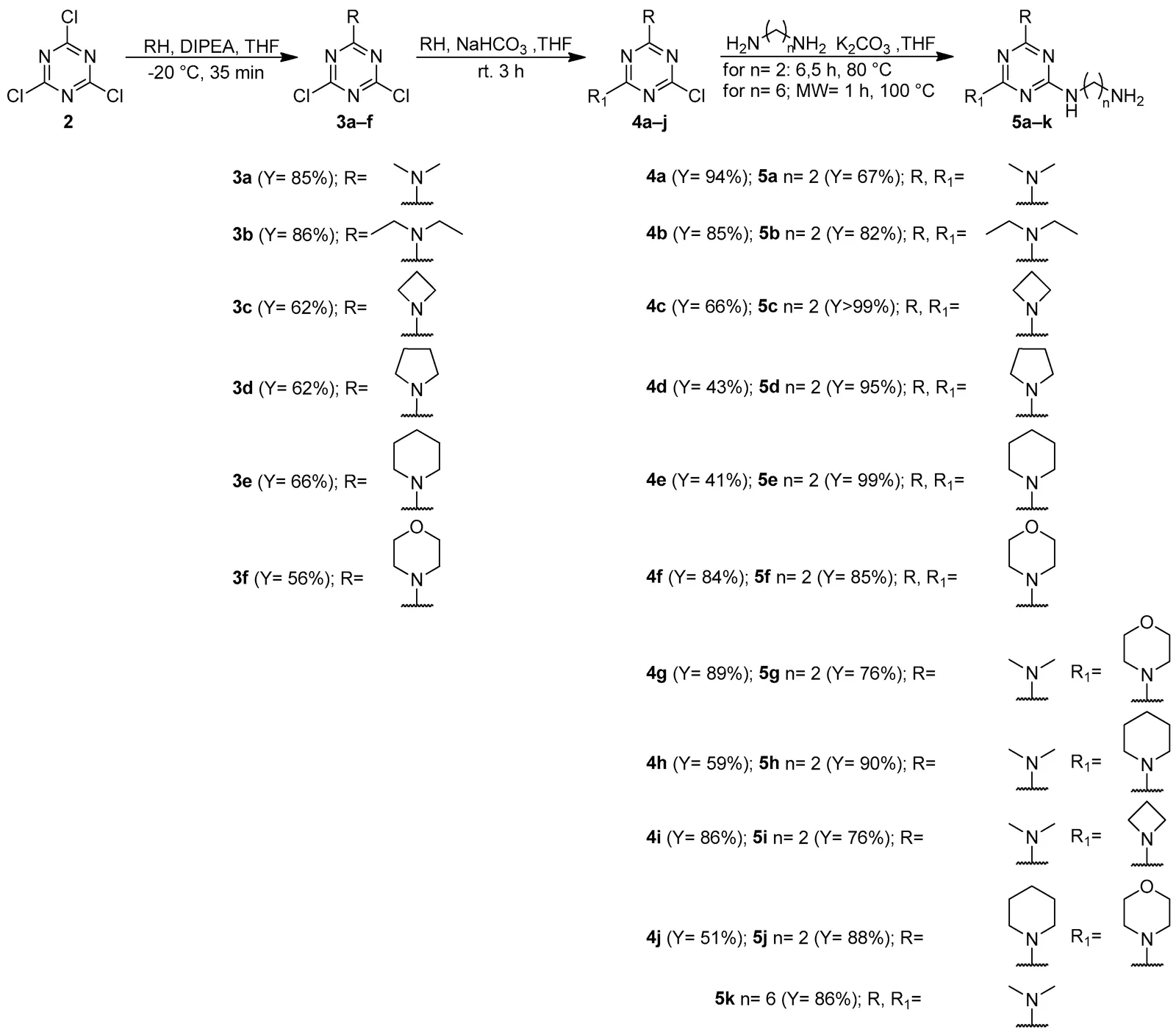
Synthesis of 1,3,5-triazines: **3a**–**f**, **4a**–**j** and **5a**–**k**.

Compounds **5a**–**k** were synthesized through a three-step procedure, shown in Figure 2, following the temperature-dependent stepwise nucleophilic substitution of cyanuric chloride (**2**). In the first step, cyanuric chloride (**2**), dissolved in THF, reacted with a secondary aliphatic or cyclic amine in the presence of *N*,*N*-diisopropylethylamine (DIPEA) at −20 °C for 35 min. These conditions were required for the selective substitution of the first chlorine atom. After filtration of the inorganic salt and evaporation of the solvent under reduced pressure, the residue was purified by flash chromatography using DCM as the eluent, affording intermediates **3a**–**f** in 56–86% yields. In the second step, triazines **3a**–**f** were dissolved in THF, followed by the addition of sodium bicarbonate and a second secondary aliphatic or cyclic amine. The reaction was carried out at room temperature for 3 h, allowing substitution of the second chlorine atom to afford disubstituted 1,3,5-triazine derivatives **4a**–**j**, which were isolated by column chromatography with yields of 41–94%. The final substitution was achieved by coupling intermediates **4a**–**j** with ethylenediamine in THF in the presence of potassium carbonate. Under conventional conditions, the reaction was performed at 80 °C for 6.5 h, providing the target compounds **5a**–**j**, which were isolated as white precipitates after solvent evaporation with yields of 67–99%. For compound **5k**, the final substitution was carried out under microwave irradiation at 100 °C for 60 min in THF with potassium carbonate and ethylenediamine, affording the desired product with an 86% yield after solvent evaporation.

**Figure 3:**
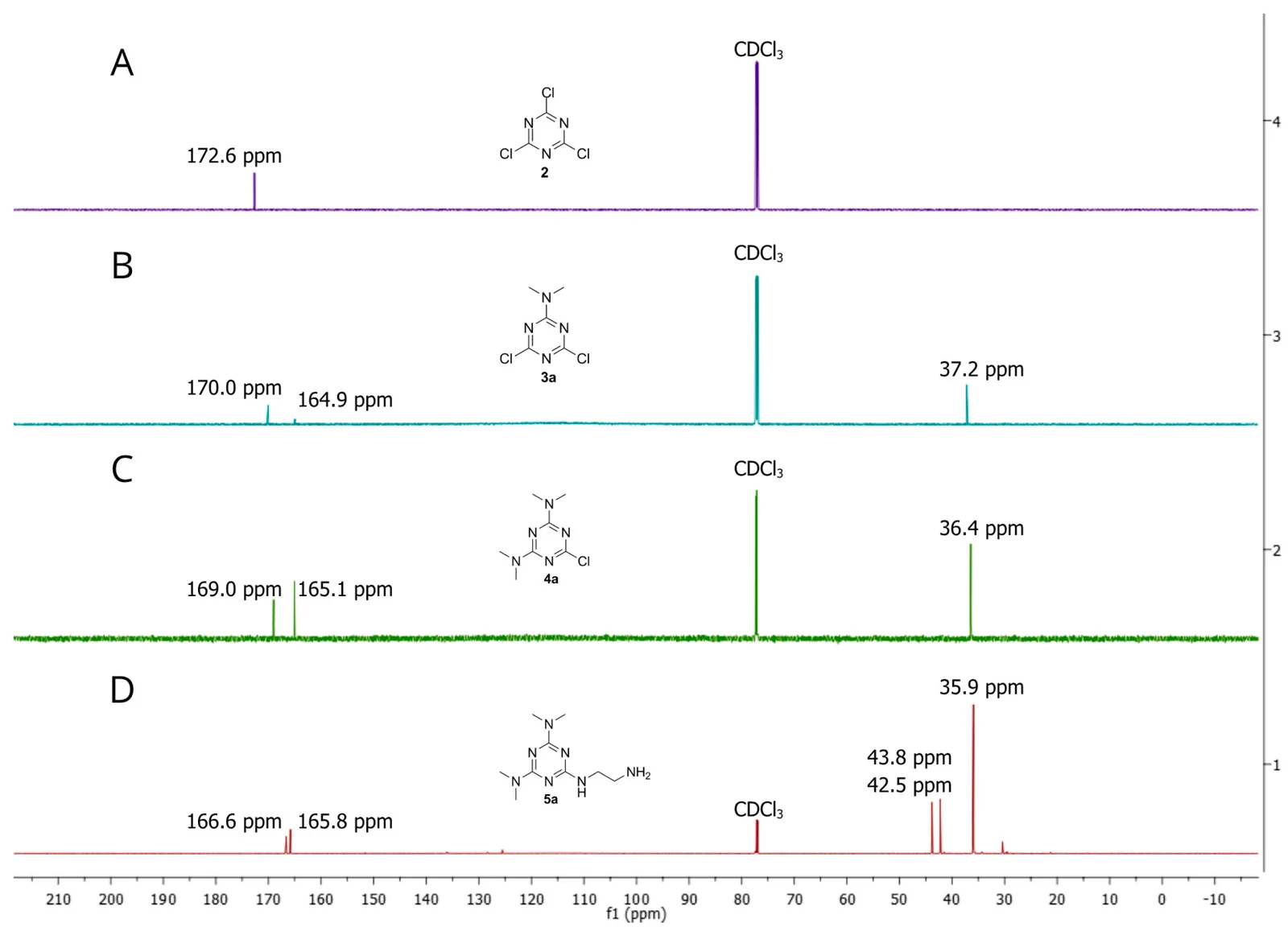
Differentiation of symmetrical mono- and disubstituted 1,3,5-triazines using ^13^C NMR spectroscopy. (**A**): compound **2**; (**B**): compound **3a**; (**C**): compound **4a**; and (**D**): compound **5a**.

While asymmetric mono- and disubstituted triazines could be distinguished based on their ^1^H NMR spectra, symmetric mono- and disubstituted 1,3,5-triazines **3a**–**f** and **4a**–**f** were difficult to differentiate due to overlapping signals. For this reason, ^13^C NMR spectroscopy was used to analyze the obtained products. The analysis presented in Figure 3 was performed for model compounds **3a**, **4a**, and **5a**. The diagnostic signals were peaks originating from 4-membered carbon atoms located in the aromatic ring. For cyanuric chloride (**2**) (Figure 3A), only one singlet was observed at 172.6 in the spectrum, which results from the symmetry of this compound. For the monosubstituted compound **3a** (Figure 3B), there were already two singlets in the aromatic region with a ratio of 2:1. The first was located at 170.0 from two aromatic carbon atoms coupled with chlorine and the second was located at 164.9 from an aromatic carbon atom coupled with a dimethylamine group. In addition, there was a singlet at 37.2 from two methyl groups. Compound **4a** (Figure 3C), which has two substituents, was also characterized by two singlets in the aromatic region with chemical shifts similar to those for compound **3a**, but with opposite intensities (1:2). In this case, the signal with lower intensity at 169.0 was from one aromatic carbon atom coupled with chlorine, and the signal with higher intensity at 165.1 was from two aromatic carbon atoms coupled with the –N(CH_3_)_2_ group. For this compound, we also observed a singlet originating from four dimethyl groups with a shift of 36.4. For compound **5a** (Figure 3D), two singlets in the aromatic region with an intensity ratio of 1:2 were observed, one originating from a carbon atom conjugated with a linker (166.6) and the other from two carbon atoms conjugated with dimethylamine groups (165.8). Additionally, in the aliphatic region, two singlets from the CH_2_ groups of methylenediamine (43.8 and 42.5) were observed, together with a singlet with a chemical shift of 35.9 from four CH_3_ groups.

**Figure 4:**
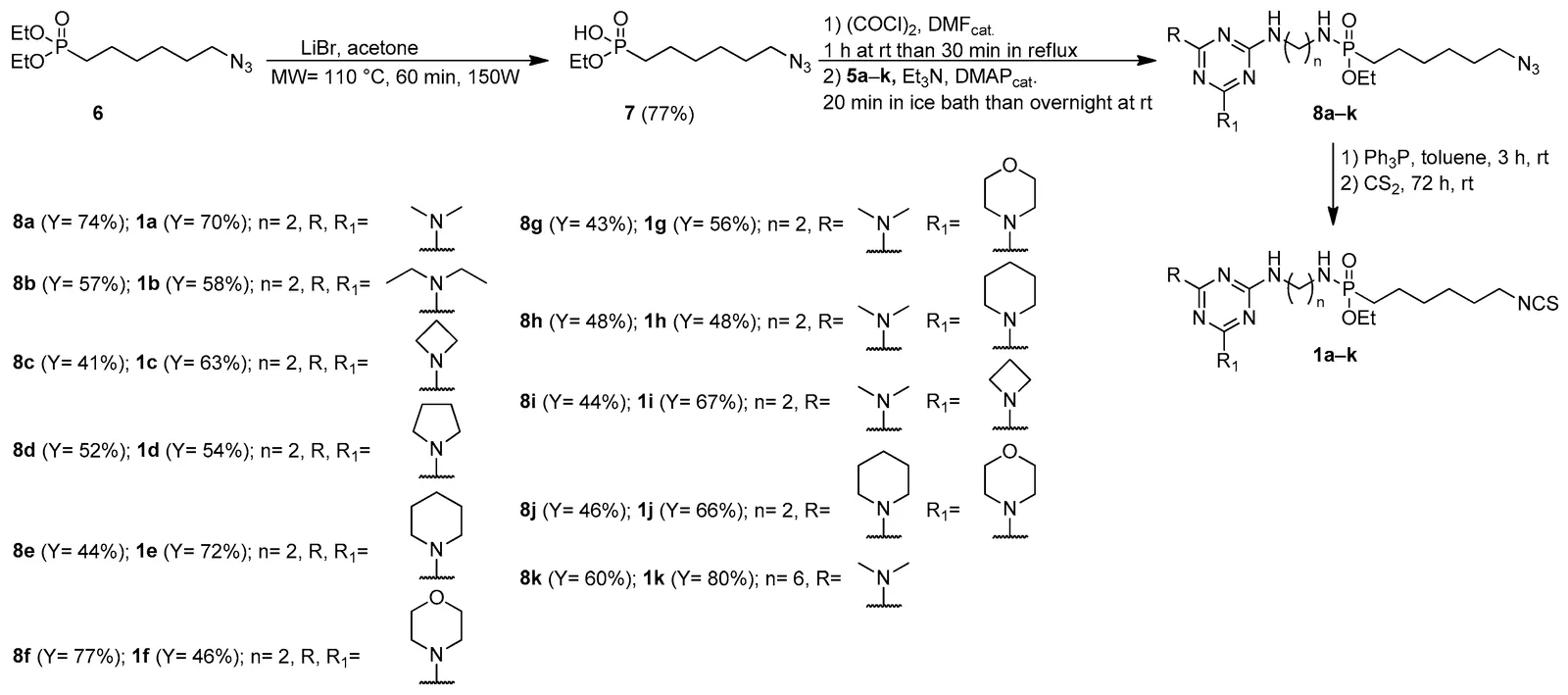
Synthesis of final isothiocyanate–triazine conjugates **1a**–**k**.

Following synthesis of the 1,3,5-triazine fragment, preparation of the phosphorus-containing fragment was initiated. Diethyl 6-(azidohexyl)phosphonate was obtained according to the procedure described by Gajda et al. [63] and subsequently converted into its monoester via a microwave-assisted reaction (110 °C, 60 min, 150 W) using lithium bromide in acetone. Once the reaction was complete, the mixture was extracted with Et_2_O to remove the unreacted substrate **6**. The aqueous layer was then acidified with concentrated HCl and extracted with chloroform. The target compound **7** was isolated after evaporation of the solvent under reduced pressure, with a yield of 77% (Figure 4). After all substrates **5a**–**k** and **7** had been obtained in high purity, the coupling process was initiated. Ethyl monoester **7** was converted to the corresponding acid monochloride via a reaction with oxalyl chloride and a catalytic amount of DMF at room temperature for 1 h, followed by 30 min at 40 °C. After evaporation of the solvent, the residue was dissolved in dichloromethane, cooled in an ice bath, and a solution of compounds **5a**–**k** in DCM was added dropwise in the presence of Et_3_N and a catalytic amount of DMAP. The reaction mixture was stirred for 20 min in an ice bath and then for 24 h at room temperature. The pure products **8a**–**k** were isolated in yields of 41–77%.

The final stage of the research involved the conversion of compounds **8a**–**k** into the target isothiocyanate–triazine conjugates **1a**–**k** via a tandem Staudinger/aza-Wittig reaction. Compounds **8a**–**k** were dissolved in toluene, cooled in an ice bath, and then triphenylphosphine was added. The reaction mixture was stirred at room temperature for 3 h. Subsequently, carbon disulfide was added dropwise, and the reaction was stirred for a further 72 h at room temperature. After evaporation of the solvent under reduced pressure, the products were isolated by column chromatography with yields of 46–80% (Figure 4).

### 3.2. ADME of Isothiocyanate–Triazine Conjugates ***1a**–**k***

In silico ADMET profiling of target derivatives **1a**–**k** was performed using the ADMET-AI platform to assess their physicochemical properties, drug-likeness parameters, topological polar surface area (TPSA), and oral bioavailability (Table 1; full dataset of 41 predicted properties is provided in the Supplementary Materials).

Based on the data presented in Table 1, four compounds (**1a**, **1c**, **1h**, and **1i**) fully complied with Lipinski’s rule of five (RO5), exhibiting a molar mass below 500 Da, logP < 5, fewer than 5 hydrogen bond donors, and no more than 10 hydrogen bond acceptors. The remaining compounds either had a molar mass above 500 Da (**1b**, **1d**–**g**, **1j**, and **1k**) or more than 10 hydrogen bond acceptors (**1f**, **1g**, and **1j**). The quantitative estimate of drug-likeness (QED) values for all compounds ranged from 0.11 to 0.17, while the calculated topological polar surface area (TPSA) values exceeded 107 Å^2^, indicating moderate oral bioavailability. For example, compound **1a** calculated oral bioavailability score 0.69. In comparison, the reference drug donepezil complies with RO5 and exhibits higher drug-likeness and bioavailability metrics (QED = 0.75, TPSA = 38.7 Å^2^, oral bioavailability = 0.88).

### 3.3. Evaluation of Biological Activity

All synthesized conjugates **1a**–**k** were evaluated in vitro for their inhibitory activity against acetylcholinesterase (AChE, from *Electrophorus electricus*) using Ellman’s method and β-secretase (BACE1, from equine serum) using a Förster resonance energy transfer (FRET) assay. Both enzymes are implicated in AD. Donepezil and quercetin were used as reference standards for comparative analysis. Altretamine, as well as PEITC and diethyl 6-(isothiocyanatohexyl)phosphonate (P-ITC), were also tested. The evaluation of compounds with documented anticancer activity (altretamine, P-ITC and PEITC) as inhibitors of AChE and BACE1 was motivated by the fact that the synthesized conjugates **1a**–**k** contain a P-ITC moiety linked to an altretamine ring or one of its derivatives. Since these compounds had not previously been investigated for their inhibitory activity against either enzyme, we sought to determine, first, whether they exhibit inhibitory properties and, second, whether their activity is greater or lesser than that of the corresponding final conjugates **1a**–**k**. Furthermore, we aimed to assess whether the conjugates **1a**–**k** display a synergistic effect resulting from the simultaneous presence of both the triazine ring and the P-ITC moiety. PEITC was also evaluated to determine whether this naturally occurring isothiocyanate exhibits inhibitory activity toward AChE and BAC-1 and to compare its activity with that of the synthesized conjugates **1a**–**k**. The results are presented in Table 2.

Based on the results presented in Table 2, it can be concluded that all synthesized isothiocyanate–triazine conjugates **1a**–**k** inhibit the activity of both AChE and BACE1. For AChE, the IC_50_ values ranged from 0.182 µM to 0.875 µM. The most active compound was **1i** (IC_50_ = 0.182 ± 0.02 µM), with a dimethylamine substituent and an azetidine ring. The presence of two dimethylamine groups in 1,3,5-triazine **1a** resulted in activity at the level of IC_50_ = 0.646 ± 0.02 µM, while replacing them with diethylamine groups increased the activity almost threefold (IC_50_ = 0.211 ± 0.02 µM). The presence of two secondary cyclic amines (**1c**–**f**) in the 1,3,5-triazine ring resulted in average activity against AChE (IC_50_ = 0.652–0.875 µM), while replacing one cyclic amine with a dimethylamine group (**1g**–**i**) resulted in more than a twofold improvement in activity (IC_50_ = 0.182–0.350 µM). Conjugate **1j**, containing two different cyclic amines, morpholine and piperidine, also exhibited high activity (IC_50_ = 0.223 ± 0.02 µM). The use of hexamethylenediamine in the synthesis of compound **1k** improved the activity twofold (IC_50_ = 0.341 ± 0.03 µM) compared to compound **1a** with ethylenediamine. Donepezil, an AChE inhibitor, showed activity at the level of IC_50_ = 0.046 ± 0.013 µM. Altretamine, a well-established 1,3,5-triazine drug, had the weakest activity (IC_50_ = 1.384 ± 0.04 µM). However, the presence of P-ITC significantly improves this activity, as can be seen for compounds **1a** and **1k**. P-ITC itself also has high AChE inhibitory activity (IC_50_ = 0.357 ± 0.03 µM). Natural PEITC has an IC_50_ value comparable to that of P-ITC (IC_50_ = 0.207 ± 0.01 µM).

As for the second enzyme, all synthesized conjugates **1a**–**k** exhibited BACE1 inhibition, but their activity against BACE1 was significantly weaker than that against AChE. Compound **1b** (IC_50_ = 8.48 ± 2.18 µM) was the most active among the entire pool of conjugates. Replacing diethylamine groups with dimethylamine groups resulted in lower inhibition (**1a**, IC_50_ = 10.52 ± 1.88 µM). The presence of two cyclic amine rings in compounds **1c** (IC_50_ = 10.06 ± 1.98 µM), **1e** (IC_50_ = 13.52 ± 1.05 µM), and **1f** (IC_50_ = 10.07 ± 2.09 µM) also reduced the inhibitory activity of the conjugates against BACE1; however, compound **1d**, bearing two pyrrolidine rings, showed activity similar to that of compound **1b** (IC_50_ = 9.01 ± 2.09 µM). In the case of mixed substituents in the 1,3,5-triazine core, compound **1h**, in which the core was substituted with dimethylamine and piperidine, exhibited relatively high activity (IC_50_ = 8.76 ± 2.53 µM). The other compounds, **1g** and **1i**, showed activity above 10 µM. Inhibition below 10 µM was also observed for conjugate **1j**, containing morpholine and piperidine rings (IC_50_ = 9.83 ± 1.41 µM), and for compound **1k**, containing two dimethylamine groups and hexamethylenediamine as a linker (IC_50_ = 9.06 ± 1.28 µM). The reference compounds—altretamine, PEITC, and P-ITC—exhibited activity of approximately 9 µM (IC_50_ = 8.89, 9.05, and 9.24 µM, respectively). Quercetin exhibited the highest activity (IC_50_ = 4.89 ± 2.31 µM), which was almost twice as strong as that of the most active conjugate, **1b**.

### 3.4. In Silico Molecular Docking and Binding Mode Profiling

The general structure of compounds **1a**–**k** incorporates a peripheral triazine ring and a terminal isothiocyanate group at opposite ends of the molecule, connected via a central phosphonamide moiety. This multifunctional architecture combines structural elements with distinct pharmacophoric features within a single scaffold, allowing their spatial separation for potential independent interactions with different regions of the active site. Simultaneously, varying the substituents at two of the three exogenous triazine nitrogen positions created a consistent series to analyze structure–binding relationships centered around the main triazine core.

**Figure 5:**
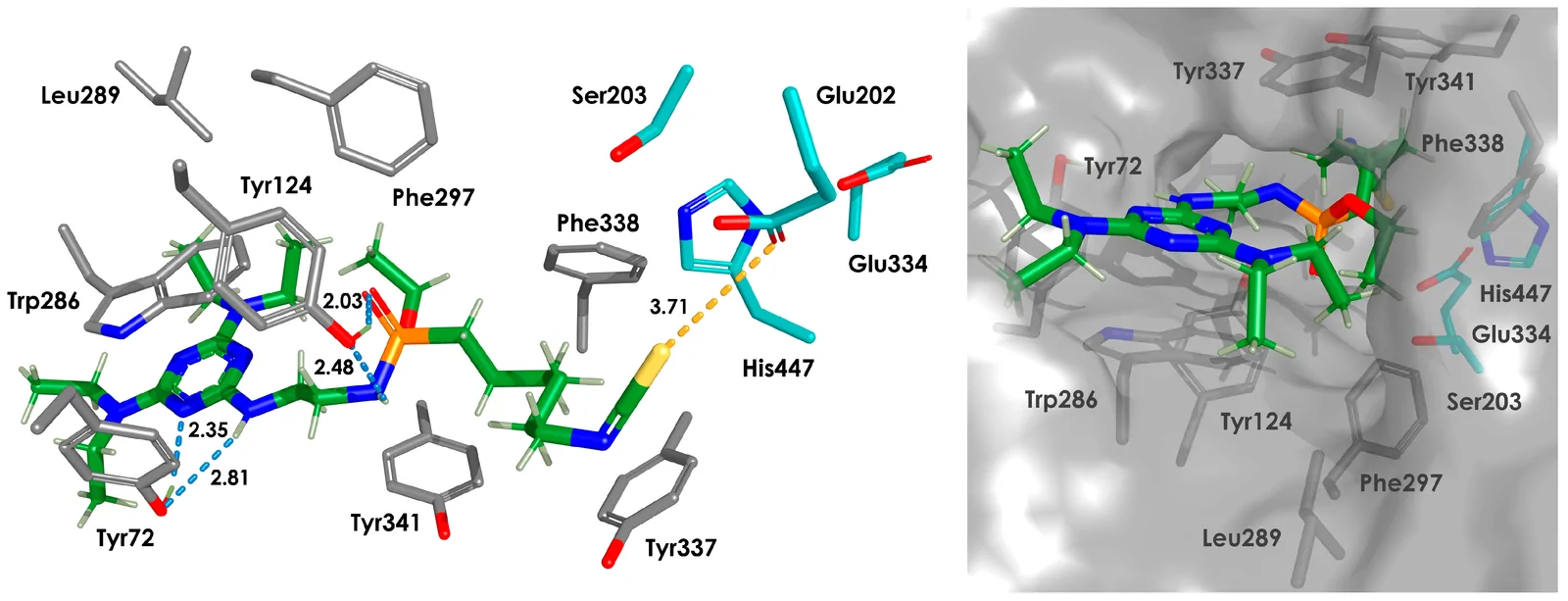
Predicted binding pose and selected noncovalent interactions of derivative **1b** within the hAChE (4EY7.pdb) binding pocket based on molecular docking simulations. The ligand molecule is shown as green sticks. Key amino acid residues forming the catalytic triad and CAS floor are shown as cyan sticks, while residues involved in noncovalent interactions (PAS and mid-gorge regions) are shown as gray sticks. Hydrogen bonds are indicated by blue dashed lines and the −N=C=S···O contact by a yellow dashed line. Distances for the indicated interactions are given in Å. Residue numbering follows the reference crystal structure.

To gain insight into potential interaction profiles and to suggest tentative binding modes for the series, molecular docking simulations and the protein–ligand interaction analysis were conducted to formulate plausible interaction hypotheses. Given the structural similarity across the series, a conserved binding orientation was assumed as a rational baseline for all derivatives (compound **1k** was evaluated separately due to extended linker length). To fulfill this assumption, pose selection relied not only on the docking score, but also on a consistent spatial conformational profile across the series. A visualization of **1b** is presented in Figure 5. All docking poses and interaction tables are available in the Supplementary Materials.

For the elongated active-site gorge of hAChE, the orientation selected as a potential binding mode for the analyzed series places the triazine moiety at the peripheral anionic site (PAS) and the isothiocyanate group facing the gorge bottom, where the catalytic active site (CAS) is located. This proposed orientation, spanning the mid-gorge region, facilitates multisite binding through the enzyme–ligand interactions observed in the docking models. The rigid triazine moiety located at the entrance of the gorge is predicted to form π-π-stacking interactions with Trp286, which are observed for all derivatives except **1f**, **1g**, and **1h**. For these compounds, the planar triazine system is tilted out of the optimal axis, disabling the stacking interaction. Further analysis of the proposed ligand poses indicates that the steric volume of the substituents, as well as the presence of heteroatoms and the geometry/symmetry of the triazine system, may influence not only its planar orientation but also how deeply it can penetrate the PAS region. In addition to aromatic stacking, the triazine core is anchored at the PAS region through a hydrogen bond between a triazine ring nitrogen and Tyr72, observed for almost all selected docking models.

Computational models indicate that the binding potency of the tested inhibitors may be strongly dependent on both hydrophobic contacts and hydrogen bonds within the mid-gorge region. The hydrophobic interactions involving Tyr337, Phe338 and, to a lesser extent, Phe297 and Tyr341 are predicted to create a dense localized network with the aliphatic regions of the derivatives, maintaining short contact distances (typically 3.3–3.9 Å). Moreover, Tyr124, located on the opposite wall of the gorge, forms strong hydrogen bonds (acting as a hydrogen bond acceptor and, in selected poses, additionally as a donor) with the phosphonamide nitrogen and, occasionally, the oxygen atoms of the phosphonamidate core, with distances ranging between 2.45 Å and 3.22 Å. In this proposed binding mode, the hydrophobic arc, together with the strong H-bond anchor at Tyr124, could establish a bottleneck that may play an important role in gorge desolvation.

In the proposed model, the flexible, five-carbon aliphatic chain provides sufficient length to locate the terminal isothiocyanate moiety in close proximity to the hAChE active site. Additionally, the conformational mobility of the aliphatic chain allows the terminal group to explore and penetrate the gorge bottom. In the generated docking poses, the isothiocyanate sulfur atom is positioned in spatial proximity to the catalytic triad environment. In the most consistent orientations, it interacts with the carboxylate group of Glu202, with -N=C=S···O distances reaching as close as 3.4–3.7 Å. Although this highly flexible moiety does not form rigid, directional hydrogen bonds, its presence near the gorge floor allows for localized dipole–dipole interactions, favorable van der Waals contacts, and desolvation of the CAS region.

Analysis of the binding poses and interaction profiles for the most potent inhibitors, particularly **1i** and **1b** (Figure 5), can provide a rational basis for the crucial role of hydrogen bonding with Tyr124 for binding affinity. Notably, the phosphonamide moiety of derivative **1i** exhibits the shortest and strongest hydrogen bond with Tyr124 (2.45 Å), while **1b** is anchored by a dual H-bond network of short contacts (2.90 Å and 3.05 Å). This key interaction is further stabilized by extensive hydrophobic contacts including π–π stacking interactions within the mid-gorge region and at the PAS entrance, involving Tyr337, Phe338, Tyr341, Phe297, Leu289, and Trp286. Additionally, the docking models show that the isothiocyanate sulfur atom reaches into the CAS region, maintaining close contact with the carboxylate oxygen of Glu202 (3.53 Å for **1i** and 3.71 Å for **1b**).

In general, the docking results indicate that the binding architecture within the hAChE gorge is governed by a consensus set of key residues: Tyr124, Tyr337, and Phe338 (which show interactions across all derivatives), alongside Trp286, Tyr341, and Glu202. Based on these observations, we suggest a general binding model in which the overall size and substitution pattern of the triazine core dictate the depth of penetration into the gorge and influence the orientation necessary for stacking interactions with Trp286. A triazine system of appropriate dimensions, combined with an H-bond donor/acceptor group located at an optimal distance, allows for the correct positioning of the phosphonamide in the mid-gorge region, successfully catching the essential Tyr124 H-bond anchor. Strong hydrogen bonding together with hydrophobic and aromatic π–π contacts, as well as the potential interaction of the isothiocyanate group with Glu202 enabled by the flexible aliphatic tail extending towards the CAS floor, supports a robust multisite binding mode. Furthermore, tailoring the size and nature of the triazine substituents to ensure optimal positioning at the PAS entrance, combined with hydrophobic contacts strengthened by the H-bond anchor at the mid-gorge bottleneck and the terminal isothiocyanate group, may synergistically promote the favorable desolvation of the hAChE gorge.

**Figure 6:**
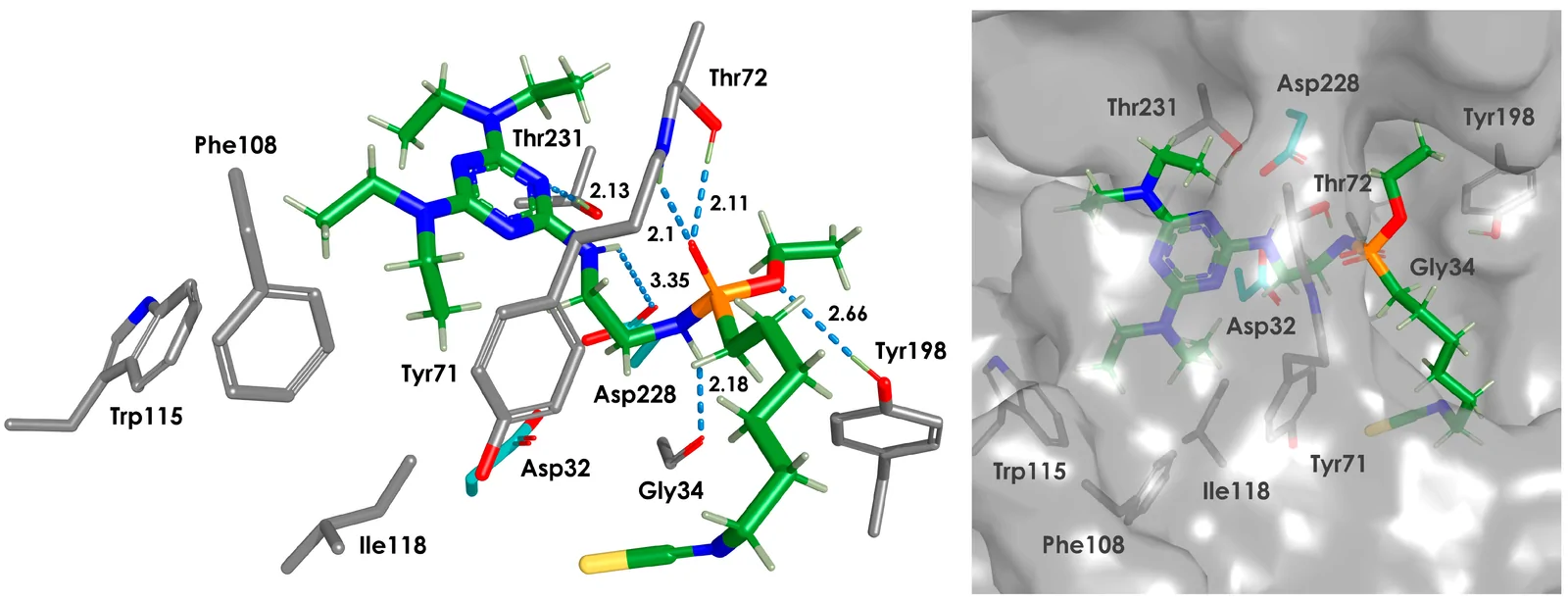
Predicted binding pose and selected noncovalent interactions of derivative **1b** within the hBACE1 (3UQW.pdb) binding pocket based on molecular docking simulations. The ligand molecule is shown as green sticks. Key amino acid residues forming the catalytic dyad are shown as cyan sticks. Residues involved in noncovalent interactions are shown as gray sticks. Hydrogen bonds are indicated by blue dashed lines. Distances for the indicated hydrogen bonds are given in Å. Residue numbering follows the reference crystal structure.

Due to the relatively open architecture of the BACE1 binding groove and the structural flexibility of the analyzed compounds, molecular docking yielded a diverse range of conformational poses. Therefore, a representative general binding orientation was established based on cluster population and the consistent spatial positioning of the triazine core across the entire series. For the selected docking poses, the triazine system is located below the flap loop, occupying the S1 and S2 pockets. The aliphatic chain extends towards the S’ sites, with the ethyl ester moiety located mainly in the S1′ pocket, while the isothiocyanate-terminated aliphatic tail is localized in the S3′–S4′ region. A visualization of **1b** is presented in Figure 6. All docking poses and interaction tables are available in the Supplementary Materials.

The induced-fit mechanism of BACE1 is primarily associated with the flap loop, and interactions with this dynamic structure are frequently observed for compounds binding near the active site [64]. Upon induced fit, the central region of the flap (Tyr71–Thr72–Gln73) constrains the size of the S1 pocket from above and partially from the sides. For the predicted poses, alongside these spatial limitations, the positioning of the triazine moiety in the S1–S2 region is supported by hydrophobic interactions, mainly with Tyr71 of the flap as well as Phe108 and Trp115 forming the pocket wall. In the proposed orientation, the triazine moiety spans the S1 and S2 regions. While its position in S1 is restricted by the flap loop, its extension into the S2 site is bounded by the Gly230–Thr232 segment. In most cases, the side-chain hydroxyl group of Thr231 forms strong hydrogen bonds with the aromatic nitrogens of the triazine ring (ranging from 2.53 Å to 3.08 Å).

Interestingly, a key anchoring point for the common molecular scaffold appears to be hydrogen bonds formed by its phosphonamide oxygen with Thr72 (located in the flap loop), involving both the side-chain hydroxyl group and the backbone amide nitrogen. These interactions do not exceed 3.1 Å. Moreover, based on the proposed binding mode, the phosphonamide moiety can be additionally stabilized by a hydrogen bond between the hydroxyl group of Tyr198 and the ester oxygen (ranging from 2.43 Å to 3.62 Å), as well as by a less frequently observed H-bond between the carbonyl oxygen of Gly34 and the phosphonamide nitrogen. Such extensive stabilization of the central phosphonamide core directly above catalytic Asp32 and Asp228 would be able to block access to the enzyme active site without direct interaction with the catalytic dyad. However, for half of the docking poses, a relatively weak hydrogen bond is observed between Asp228 and the nitrogen atom connecting the triazine ring to the rest of the molecule (ranging from 2.87 Å to 4.01 Å).

According to the proposed binding mode, Tyr198 and Ile226 contribute to the stabilization of the ethyl ester group in the S1′ pocket via hydrophobic interactions. On the other hand, the high flexibility of the aliphatic isothiocyanate chain and the relatively spacious nature of the S3′–S4′ region does not allow for an unambiguous determination of specific interactions involving the isothiocyanate group, nor for the definitive selection of an optimal binding pose.

Detailed inspection of individual docking poses for compounds **1b** and **1h** revealed a specific profile of interactions. The binding mode of compound **1b** forms an extended network of hydrogen bonds within the active site (Figure 6). In addition to the interactions with Thr72 (3.04 Å) and Tyr198 (3.62 Å), it also forms an H-bond with the carbonyl oxygen of Gly34 (3.11 Å). Furthermore, the binding mode of **1b** shows stabilization in the S2 region through a hydrogen bond with Thr231 (3.08 Å) and a weak contact with Asp228 (4.10 Å), supported by hydrophobic interactions with Tyr71, Phe108, Trp115, and Ile118.

A distinct interaction pattern was also observed for derivative **1h**. In the binding mode of **1h**, a short hydrogen bond formed between the hydroxyl group of Tyr198 and the ligand (2.64 Å), alongside a hydrogen bond with Thr231 (2.74 Å). Additionally, **1h** formed hydrogen bonds with Asp228 (3.06 Å) and Arg128 (3.01 Å).

In summary, the proposed docking model for the hBACE1 complex can be conceptualized around three main structural features: (1) the orientation of the triazine core within the S1–S2 region, supported by hydrophobic contacts and spatial limitations imposed by the flap loop and the groove walls; (2) an extensive hydrogen-bonding network centered around the phosphonamide moiety directly above the catalytic dyad (Asp32/Asp228), providing a key anchoring point; (3) the extension of the aliphatic tail towards the S1′ and S3′–S4′ binding sites.

## 4. Discussion

Analysis of the synthesis of the target conjugates **1a**–**k** revealed that both the synthesis of the intermediates containing the 1,3,5-triazine scaffold (**3a**–**f**, **4a**–**j**, and **5a**–**k**) and the synthesis of phosphonate monoester **7** proceeded without significant difficulties. To determine the overall reaction yield, the first step was the coupling of the trisubstituted triazines **5a**–**k** with the acid chloride generated in situ from monoester **7** and oxalyl chloride. This transformation required low reaction temperatures and the slow dropwise addition of a solution of triazine in dichloromethane to the acid chloride solution. Elevated temperatures or excessively rapid addition promoted the formation of the major side product, pyrophosphate. Another yield-determining step was the conversion of the azide group into the corresponding isothiocyanate via the Staudinger/aza-Wittig reaction. In addition to the target compounds **1a**–**k**, the reaction also generated triphenylphosphine oxide and triphenylphosphine sulfide as by-products. Their removal by column chromatography reduced the isolated yields of the target compounds.

Analysis of the biological evaluation demonstrated that the synthesized isothiocyanate–triazine conjugates **1a**–**k** exhibited inhibitory activity toward AChE, as summarized in Table 2. However, analysis of the structure–activity relationship (SAR) of compounds **1a**–**k** did not reveal a single, clear trend governing their inhibitory potency. The obtained results indicate that aliphatic amino substituents attached to the 1,3,5-triazine core (dimethylamino and diethylamino groups) generally confer stronger AChE inhibition than cyclic amino substituents, such as azetidine, pyrrolidine, piperidine, or morpholine. Comparison of compounds **1a** and **1b** shows that conjugate **1b**, bearing two diethylamino groups, is a more potent inhibitor than its dimethylamino analog (**1a**). This effect may be attributed to the greater steric bulk and lipophilicity of the diethylamino substituents. In contrast, no straightforward relationship between the size of the cyclic substituent and inhibitory activity was observed.

Among the cyclic analogs, compound **1e**, containing two piperidine rings, displayed the weakest inhibitory activity, followed by **1c** (two azetidine rings) and **1d** (two pyrrolidine rings) (**1e** < **1c** < **1d** < **1f**). The most potent inhibitor within this subgroup was **1f**, bearing two morpholine rings. These findings suggest that the presence of the oxygen atom in the morpholine ring may enhance biological activity relative to the corresponding piperidine analogue. The mixed-substituent conjugates **1g**–**j** also exhibited an interesting activity profile. Compounds containing both a dimethylamino group and a cyclic amino substituent showed substantially stronger AChE inhibition than compound **1a**, bearing two dimethylamino groups, as well as analogs containing two identical cyclic amino substituents (**1c**, **1e**, and **1f**). Particularly noteworthy is compound **1j**, which incorporates both morpholine and piperidine rings. Despite the relatively high steric demand of these substituents, **1j** exhibited approximately threefold and fourfold greater inhibitory potency than compounds **1f** and **1e**, respectively. Three conjugates (**1b**, **1i**, and **1j**) displayed IC_50_ values below 0.3 μM. Compound **1i** was identified as the most potent AChE inhibitor in this series. However, it remained approximately fourfold less potent than the reference inhibitor donepezil (IC_50_ = 0.046 ± 0.01 μM).

Numerous AChE inhibitors have been reported in the literature. In addition to cyanopyridine–triazine hybrids [65], triazine–triazolopyrimidine hybrids [66], coumarin–1,3,5-triazine derivatives [67], and peptide–triazine conjugates [44], which exhibit high inhibitory activity toward AChE, several other classes of triazine-based inhibitors have also been described, including 1,3,5-triazine–benzimidazole hybrids [68], genistein-O-1,3,5-triazine derivatives [69], 1,3,5-triazine–quinoline derivatives [70], and 2-phenylthiazole–1,3,5-triazine hybrids [43]. These compounds incorporate the same amino substituents on the 1,3,5-triazine core as those employed in the present study, including aliphatic amines (e.g., diethylamine) and cyclic amines (e.g., pyrrolidine and morpholine). Despite sharing these structural features, the reported compounds exhibit markedly weaker AChE inhibitory activity than the isothiocyanate–triazine conjugates described in the present study. This may be attributed to the presence of only a single secondary amine (either aliphatic or cyclic) instead of two substituents, or to the incorporation of a bulkier substituent, such as a benzimidazole or quinoline moiety, which may fit less favorably into the active-site pocket of AChE.

As with AChE inhibition, it is difficult to establish an unambiguous structure–activity relationship (SAR) for BACE1 inhibition. The biological results indicate that substituents derived from aliphatic amines (diethylamine (**1b**) > dimethylamine (**1a**)) are more favorable than those derived from cyclic amines (pyrrolidine (**1d**) > azetidine (**1c**) ≈ morpholine (**1f**) > piperidine (**1e**)), which may result from greater steric hindrance. Among compounds containing mixed substituents, the SAR is also difficult to define. On one hand, compound **1h**, containing a combination of dimethylamine and piperidine moieties, exhibited high inhibitory activity. On the other hand, similarly high activity was observed for compound **1j**, bearing morpholine and piperidine rings (also high steric hindrance). These results may indicate that the piperidine ring plays an important role in inhibitory activity. However, the presence of two piperidine rings resulted in the weakest BACE1 inhibition, as observed for compound **1e**.

Only four of the synthesized conjugates (**1b**, **1d**, **1h**, and **1k**) exhibited satisfactory inhibitory activity against BACE1. However, only compounds **1b** and **1h** showed IC_50_ values below 9 µM (see Table 2), with inhibitory potency almost two-fold lower than that of Quercetin, which itself is considered only a moderate inhibitor (IC_50_ = 4.89 ± 2.31 µM). Several classes of BACE1 inhibitors have been reported in the literature, including peptide conjugates [44] and benzimidazole/1,3,5-triazine-2,4-diamine hybrids, which demonstrated relatively weak inhibitory activity [71]. Significantly more potent BACE1 inhibitors have been developed based on a 1,3,5-triazine scaffold substituted with two pyrrolidine, piperidine, or morpholine rings combined with a benzothiazole fragment, showing submicromolar inhibitory activity [72,73]. Interestingly, the conjugates synthesized in the present study also contain two pyrrolidine, piperidine, or morpholine rings (**1d**, **1e**, and **1f**). However, their inhibitory activity was considerably lower. These findings indicate that the nature of the third substituent has a critical influence on BACE1 inhibition. In this context, the benzothiazole fragment appears to be more favorable than the NCS fragment, as it enables the formation of a hydrogen bond with Asp32, an interaction considered important for inhibition of BACE1 activity.

In recent years, clinical trials of five BACE1 inhibitors, initially regarded as promising therapeutic candidates due to their nanomolar potency (IC_50_ or Ki values), have been discontinued. Their administration was associated with adverse effects, including weight loss, hair loss, and skin rashes [74]. Therefore, the rational design of BACE1 inhibitors, together with the identification of an appropriate therapeutic dose that balances efficacy and safety, remains a critical challenge for the development of future anti-Alzheimer’s disease strategies. In this context, the multi-target-directed ligand approach has gained increasing attention. Combining balanced activity with dual-target selectivity towards AChE and BACE1 is considered a promising strategy that may offer synergistic therapeutic efficacy while potentially minimizing off-target toxicity [75].

Comparison of the biological activity of conjugates **1a** and **1k**, which differ only in linker length (n = 2 and n = 6, respectively), against both AChE and BACE1 indicates that the longer linker is more favorable for enzyme inhibition. This effect may be attributed to the greater flexibility of the longer spacer, allowing the molecule to adopt a more favorable conformation and achieve a better fit within the enzyme’s active-site pocket.

Analysis of the reference compounds (altretamine, P-ITC, and PEITC) revealed that only altretamine exhibited higher inhibitory activity toward BACE1 than toward AChE. In contrast, both ITCs inhibited AChE more effectively than BACE1. Against both enzymes, PEITC displayed higher inhibitory potency than P-ITC.

Comparison of the biological activity of the synthesized compounds with that of the parent compounds (altretamine and P-ITC) indicates that only selected conjugates exhibit dual-target activity. With respect to AChE inhibition, compounds **1b**, **1g**, **1i**, and **1j** demonstrated enhanced activity, whereas for BACE1 inhibition a synergistic effect was observed exclusively for compound **1b**. The remaining conjugates did not exhibit synergism, as their biological activity was comparable to or lower than that of the parent compounds. Since the synthesized conjugates **1a**–**k** were designed as dual-target ligands directed against enzymes involved in neurodegenerative diseases, their pharmacokinetic properties, blood–brain barrier (BBB) penetration, and predicted clinical toxicity are crucial parameters for evaluating their drug-likeness. However, not all of the synthesized compounds exhibited equally favorable profiles.

The predicted BBB permeability values of the conjugates ranged from 0.72 to 0.92 (see Supplementary Materials), with compound **1h** displaying the highest value (0.92), exceeding the 0.90 threshold generally considered desirable for CNS-active molecules. These results indicate good, although not optimal, BBB penetration for most of the synthesized conjugates. The predicted raw clinical toxicity values ranged from 0.36 to 0.56. Since higher raw ClinTox values indicate a higher predicted clinical toxicity signal, the lower values within this range represent a more favorable predicted toxicity profile. Accordingly, compounds **1a** and **1k**, bearing two dimethylamino groups in the 1,3,5-triazine ring, exhibited the most favorable raw clinical toxicity scores (0.36 and 0.37, respectively) among all the synthesized derivatives.

The in silico physicochemical and ADMET properties were predicted, and the analysis further revealed that only four conjugates (**1a**, **1c**, **1h**, and **1i**) complied with Lipinski’s Rule of Five and exhibited acceptable predicted oral bioavailability (0.58–0.69). Among these compounds, conjugates **1h** and **1i** demonstrated high inhibitory activity against AChE, while compound **1h** also showed good inhibitory activity toward BACE1 (see Table 2).

Considering both the predicted pharmacokinetic properties and the biological activity, compound **1h** appears to be the most promising lead structure for the future design of dual AChE and BACE1 inhibitors. This compound complies with Lipinski’s Rule of Five, exhibits high predicted probability of predicted BBB penetration (0.92) and acceptable predicted oral bioavailability (0.58), but has the least favorable predicted clinical toxicity profile within the synthesized series (0.56). It also exhibits high inhibitory activity toward both AChE and BACE1. Therefore, compound **1h** represents a promising starting point for further optimization, whereas the remaining compounds failed to simultaneously meet both the pharmacokinetic and biological criteria required to be considered candidates for further development as an anti-Alzheimer’s disease agents. Nevertheless, these findings are based solely on in silico predictions and preliminary biological data and therefore require confirmation through further in vitro and in vivo studies.

In order to propose the hypothesis that the synthesized conjugates bind to the target enzymes through a multifunctional binding mechanism, molecular docking simulations were conducted. To rationalize potential binding models and propose a functional group interaction profile based on the individual contribution of each pharmacophoric group, a binding mode exploration approach was applied to obtain a plausible orientation of the scaffold structure within the binding centers of both enzymes. Due to the narrow range of experimental IC_50_ values observed across the series and the inherent resolution limits of scoring functions used in docking calculations, the analysis focused primarily on establishing a plausible, general multisite binding hypothesis via structure-binding relationship (SBR) analysis, rather than rationalizing minor activity differences among individual compounds.

The resulting hypothesis for the conjugate–hAChE binding model features a distinct spatial distribution: the triazine ring occupies the PAS entrance, the phosphonamide moiety interacts within the mid-gorge, and the flexible isothiocyanate tail targets the CAS region. This architecture aligns well with the experimentally determined binding mode of donepezil (utilized for re-docking and method validation; see Supplementary Materials). In its crystal structure (PDB ID: 4EY7), donepezil positions its larger aromatic framework at the PAS and engages in π–π stacking with Trp286, confirming that accommodating the bulky aromatic core at the gorge entrance represents a plausible and energetically favorable binding mode. Interestingly, for the proposed orientation where the triazine moiety blocks the gorge entrance, the length of the conjugates allows for interactions between the ligand’s isothiocyanate group and residues in close proximity to the catalytic triad, with Glu202 being a suggested candidate for such contact. Additionally, based on the presented model, the phosphorus-containing region may act as an anchor point via hydrogen bonding interactions.

Analysis of the proposed conjugate–hBACE1 binding model suggests that a multi-site mode of interaction is the primary driving force for enzyme binding. The presence of a strong central anchoring focused on the phosphonamide moiety could potentially compensate for any non-optimal fit of individual side chains or substituents within the outer sub-pockets. This assumption offers a plausible framework to rationalize the experimental data, explaining both the micromolar inhibitory potency against BACE1 and the overall low diversity of IC_50_ values within the series. Further structural modifications within the proposed conjugate scaffold aimed at achieving better structural complementarity—particularly in the case of BACE1—could potentially lead not only to higher inhibitory potency but also to improved selectivity toward this protease. Overall, the presented findings provide a solid foundation for future structural optimization, while the obtained results support the hypothesis that this class of multifunctional isothiocyanate–triazine conjugates represents an attractive scaffold for the future design of dual-target AChE/BACE1 inhibitors.

## 5. Conclusions

In this study, we synthesized 11 previously undescribed isothiocyanate–triazine conjugates **1a**–**k** in satisfactory to good yields and in high purity. All synthesized compounds were evaluated as inhibitors of AChE and BACE1, which are key enzymes involved in AD, and all conjugates exhibited significant biological activity. However, high biological activity alone is not sufficient to qualify a compound as a promising drug candidate. Other factors, including pharmacokinetic properties, bioavailability, cytotoxicity, and the ability to penetrate the blood–brain barrier (BBB), are equally important. In the case of conjugates **1a**–**k**, these parameters are not yet optimal, and therefore none of the synthesized compounds can currently be considered a viable candidate for the treatment of AD. Nevertheless, the synthesized isothiocyanate–triazine conjugates, obtained by combining two biologically active fragments—a 1,3,5-triazine core substituted with secondary aliphatic or cyclic amines and a phosphorus-containing isothiocyanate derivative—represent promising lead-like molecular scaffolds. Following further structural optimization, these compounds may meet the requirements for effective inhibitors of enzymes involved in the pathogenesis of AD.

Moreover, the synthesized conjugates provide valuable insights into the structure–activity relationships of both 1,3,5-triazine and isothiocyanate derivatives. They also establish a foundation for the development of a new class of isothiocyanate–triazine conjugates, which will be investigated in future studies.

## Figures and Tables

**Table 1:** ADMET-Based Pharmacokinetic Evaluation of Isothiocyanate–Triazine Conjugates **1a**–**k**.

No	Comp.	Molecular Weight [Da]	LogP	Hydrogen Bond Acceptors	Hydrogen Bond Donors	Lipinski Rule of 5	QED	Tpsa	Oral Bioavailability
1	**1a**	458.57	2.90	10	2	4	0.17	107.87	0.69
2	**1b**	514.68	4.46	10	2	3	0.11	107.87	0.58
3	**1c**	482.60	3.19	10	2	4	0.16	107.87	0.67
4	**1d**	510.65	3.97	10	2	3	0.15	107.87	0.62
5	**1e**	538.70	4.75	10	2	3	0.13	107.87	0.55
6	**1f**	542.65	2.44	12	2	2	0.14	126.33	0.65
7	**1g**	500.61	2.67	11	2	2	0.15	117.10	0.66
8	**1h**	498.64	3.82	10	2	4	0.15	107.87	0.58
9	**1i**	470.58	3.04	10	2	4	0.16	107.87	0.68
10	**1j**	540.68	3.59	11	2	2	0.14	117.10	0.59
11	**1k**	514.68	4.46	10	2	3	0.11	107.87	0.62
12	Donepezil	379.50	4.36	4	0	4	0.75	38.77	0.88

**Table 2:** In vitro inhibition of AChE and BACE1 by the target compounds **1a**–**k**.

No	Compound	*ee*AChE IC_50_ (µM) ^a^	*h*BACE1 IC_50_ (µM) ^b^
1	**1a**	0.646 ± 0.02	10.52 ± 1.88
2	**1b**	0.211 ± 0.02	8.48 ± 2.18
3	**1c**	0.851 ± 0.04	10.06 ± 1.98
4	**1d**	0.766 ± 0.01	9.01 ± 2.09
5	**1e**	0.875 ± 0.03	13.52 ± 1.05
6	**1f**	0.652 ± 0.04	10.07 ± 2.09
7	**1g**	0.321 ± 0.02	17.05 ± 2.17
8	**1h**	0.350 ± 0.01	8.76 ± 2.53
9	**1i**	0.182 ± 0.02	12.58 ± 2.14
10	**1j**	0.223 ± 0.02	9.83 ± 1.41
11	**1k**	0.341 ± 0.03	9.06 ± 1.28
12	Altretamine	1.384 ± 0.04	8.89 ± 2.03
13	PEITC	0.207 ± 0.01	9.05 ± 2.09
14	P-ITC	0.357 ±0.03	9.24 ± 1.74
15	Donepezil [44]	0.046 ± 0.01	*–*
16	Quercetin [44]	*–*	4.89 ± 2.31

^a^ AChE from Electrophorus electricus (electric eel); IC_50_, inhibitor concentration (mean ± SD of three independent experiments) resulting in 50% inhibition of AChE. ^b^ BACE1 human; IC_50_, inhibitor concentration (mean ± SD of three independent experiments) resulting in 50% inhibition of BACE1.

## Data Availability

The original contributions presented in this study are included in the article. Further inquiries can be directed to the corresponding author.

## Supplementary Materials

The following supporting information can be downloaded at https://www.mdpi.com/article/10.3390/biom16091280/s1, The analytical data for compounds **3a**–**f** (S1. The analytical data for compounds **3a**–**f**) [76,77,78,79,80], **4a**–**j** (S2. The analytical data for compounds **4a–j**) [81,82,83,84], **5a**–**j** (S3. The analytical data for compounds **5a–j**) [85,86], **6** (S4. The analytical data for compound **6**) [63], **7** (S5. The analytical data for compound **7**), **8a**–**k** (S6. The analytical data for compounds **8a**–**k**) and **1a**–**k** (S7. The analytical data for compounds **1a**–**k**); Figures S1–S3: ^1^H, ^13^C and ^31^P NMR **7**; Figures S4–S108: ^1^H and ^13^C NMR, UV-Vis Chromatogram and Mass Spectrum **3a**–**f**, **4a**–**j** and **5a**–**k**; Figures S109–S155: ^1^H, ^13^C and ^31^P NMR, UV-Vis Chromatogram, Mass Spectrum **8a**–**k**; Figures S156–S217: ^1^H, ^13^C and ^31^P NMR, UV-Vis Chromatogram, Mass Spectrum and IR **1a**–**k**; Figures S218–S228: Dose–response curves on AChE for compounds **1a**–**k**; Figures S229–S239: Dose–response curves on BACE1 for compounds **1a**–**k**; Figure S240. Re-docking poses of AChE and BACE1 ligands aligned with their respective crystal structures; Figure S241. Docking pose of quercetin in the BACE1 binding cavity; Figure S242. Representative docking poses and protein–ligand interaction patterns for compounds **1a**–**k** in the active site of acetylcholinesterase (AChE; PDB ID: 4EY7); Figure S243. Representative docking poses and protein–ligand interaction patterns for compounds **1a**–**1k** in the active site of human beta-secretase 1 (BACE1; PDB ID: 3UQW). Theoretical ADMET properties were calculated using ADMET-AI and are given in the ADMET file.

## Abbreviations

The following abbreviations are used in this manuscript:

6-MSITC	6-(Methylsulfinyl)hexyl isothiocyanate
Aβ	Amyloid beta
AChE	Acetylcholinesterase
AD	Alzheimer’s disease
ADMET	Absorption, distribution, metabolism, excretion, and toxicity properties of molecules
AITC	Allyl isothiocyanate
APP	Amyloid precursor protein
ARE	Antioxidant response element
BACE1	β site APP cleaving enzyme 1, β secretase
BBB	Blood–Brain Barrier
BChE	Butyrylcholinesterase
BITC	Benzyl isothiocyanate
CDK5	Cyclin-dependent kinase 5
DCM	Dichloromethane
DIPEA	N,N-diisopropylethylamine
DMAP	4-Dimethylaminopyridine
DMF	Dimethylformamide
ER	Erucin
FRET	Fluorescence resonance energy transfer
ITCs	Isothiocyanates
LC-MS	Liquid chromatography coupled with mass spectrometry
MAPT	Microtubule-associated protein tau
MTDLs	Multi-target-directed ligands
MW	Microwave
NF-κB	Nuclear factor kappa-light-chain-enhancer of activated B cells
NFTs	Neurofibrillary tangles
Nrf2	Nuclear factor erythroid 2–related factor 2
P-ITC	Phosphorus analog of isothiocyanate
PEITC	Phenylethyl isothiocyanate
QED	Quantitative estimate of drug-likeness
SFN	Sulforaphane
TPSA	Topological polar surface area
TLC	Thin-layer chromatography

## References

[1] Alzheimer’s Disease International (2024). World Alzheimer Report 2024: Global Changes in Attitudes to Dementia.

[2] Better M.A. (2023). 2023 Alzheimer’s disease facts and figures. Alzheimer’s Dement.

[3] Rusowicz J., Pezdek K., Szczepańska-Gieracha J. (2021). Needs of Alzheimer’s Charges’ Caregivers in Poland in the Covid-19 Pandemic—An Observational Study. Int. J. Environ. Res. Public Health.

[4] De Ture M.A., Dickson D.W. (2019). The Neuropathological Diagnosis of Alzheimer’s Disease. Mol. Neurodegener..

[5] Gallardo G., Holtzman D.M. (2019). Amyloid-β and Tau at the Crossroads of Alzheimer’s Disease. Adv. Exp. Med. Biol..

[6] Bugaj A.M., Jermakow N. (2016). Mechanisms Underlying Alzheimer’s Disease. Neuropsychiatry Neuropsychol..

[7] Zhou S., Huang G. (2021). Synthesis and Activities of Acetylcholinesterase Inhibitors. Chem. Biol. Drug Des..

[8] Muscarà C., Gugliandolo A., Mazzon E., Cali G. (2025). Glucosinolate Metabolites and Brain Health: An Updated Review on Their Potential Benefits in Neurodegenerative, Neurodevelopmental, and Psychiatric Disorders. Antioxidants.

[9] Otoo R.A., Allen A.R. (2023). Sulforaphane’s Multifaceted Potential: From Neuroprotection to Anticancer Action. Molecules.

[10] Khan W.U., Salman M., Ali M., Majid H., Yar M.S., Akhtar M., Parvez S., Najmi A.K. (2024). Neuroprotective Effects of Sulforaphane in a rat model of Alzheimer’s Disease induced by Aβ (1-42) peptides. Neurochem. Int..

[11] Fahey J.W., Liu H., Batt H., Panjwani A.A., Tsuji P. (2025). Sulforaphane and Brain Health: From Pathways of Action to Effects on Specific Disorders. Nutrients.

[12] Alves I., Araujo E.M.Q., Dalgaard L.T., Singh S., Borsheim E., Carvalho E. (2025). Protective Effects of Sulforaphane Preventing Inflammation and Oxidative Stress to Enhance Metabolic Health: A Narrative Review. Nutrients.

[13] Wu N., Luo Z., Deng R., Zhang Z., Zhang J., Liu S., Luo Z., Qi Q. (2025). Sulforaphane: An Emerging Star in Neuroprotection and Neurological Disease Prevention. Biochem. Pharmacol..

[14] Olayanju J.B., Bozic D., Naidoo U., Sadik O.A. (2024). A Comparative Review of Key Isothiocyanates and Their Health Benefits. Nutrients.

[15] Ho E., Wong C.P., Bouranis J.A., Shannon J., Zhang Z. (2025). Cruciferous Vegetables, Bioactive Metabolites, and Microbiome for Breast Cancer Prevention. Annu. Rev. Nutr..

[16] Kattel S., Antonious G.F. (2025). Glucosinolates in Cruciferous Vegetables: Genetic and Environmental Regulation, Metabolic Pathways, and Cancer-Preventive Mechanisms. Int. J. Plant Biol..

[17] Labudda M., Rybarczyk-Płońska A., Sobieszek K.A., Niedziński T., Wurlitzer W.B., Muszyńska E., Prabucka B., Florczak S., Tomczykowa M., Makowski W. (2026). The Chemopreventive and Anticancer Potential of Glucosinolates and Their Hydrolysis Products from Cruciferous Vegetables. Nutrients.

[18] Quintero-Rincón P., Caballero-Gallardo K., Olivero-Verbel J. (2025). Natural Anticancer Agents: Prospection of Medicinal and Aromatic Plants in Modern Chemoprevention and Chemotherapy. Nat. Prod. Bioprospect..

[19] Gromek P., Kruczkowska W., Górecki K., Gałęziewska J., Płuciennik E., Janczewski Ł., Kciuk M., Zhao L.Y., Pasieka Z., Kałuzińska–kołat Ż. (2026). Structures, Characteristics, and Anticancer Mechanisms of Taxanes, Beta-glucans, and Isothiocyanates. Mol. Biol. Rep..

[20] Bertova A., Kontar S., Ksinanova M., Vergara A.Y., Sulova Z., Breier A., Imrichova D. (2024). Sulforaphane and Benzyl Isothiocyanate Suppress Cell Proliferation and Trigger Cell Cycle Arrest, Autophagy, and Apoptosis in Human AML Cell Line. Int. J. Mol. Sci..

[21] Hoch C.C., Shoykhet M., Weiser T., Griesbaum L., Petry J., Hachani K., Multhoff G., Bashiri Dezfouli A., Wollenberg B. (2024). Isothiocyanates in Medicine: A Comprehensive Review on Phenylethyl-, Allyl-, and Benzyl-isothiocyanates. Pharmacol. Res..

[22] Coscueta E.R., Sousa A.S., Reis C.A., Pintado M.M. (2022). Phenylethyl Isothiocyanate: A Bioactive Agent for Gastrointestinal Health. Molecules.

[23] Kaiser A.E., Baniasadi M., Giansiracusa D., Giansiracusa M., Garcia M., Fryda Z., Wong T.L., Bishayee A. (2021). Sulforaphane: A Broccoli Bioactive Phytocompound with Cancer Preventive Potential. Cancers.

[24] Zhang J., Li X., Ge P., Zhang B., Wen L., Gu C., Zhou X. (2022). Sulforaphene: Formation, Stability, Separation, Purification, Determination and Biological Activities. Sep. Purif. Rev..

[25] Janczewski Ł. (2022). Sulforaphane and Its Bifunctional Analogs: Synthesis and Biological Activity. Molecules.

[26] Bartkowiak-Wieczorek J., Malesza M., Malesza I., Hadada T., Winkler-Galicki J., Grzelak T., Mądry E. (2024). Methylsulfinyl Hexyl Isothiocyanate (6-MSITC) from Wasabi Is a Promising Candidate for the Treatment of Cancer, Alzheimer’s Disease, and Obesity. Nutrients.

[27] Kamal M.M., Akter S., Lin C.N., Nazzal S. (2020). Sulforaphane as an Anticancer Molecule: Mechanisms of Action, Synergistic Effects, Enhancement of Drug Safety, and Delivery Systems. Arch. Pharm. Res..

[28] Na G., He C., Zhang S., Tian S., Bao Y., Shan Y. (2023). Dietary Isothiocyanates: Novel Insights into the Potential for Cancer Prevention and Therapy. Int. J. Mol. Sci..

[29] Dufour V., Stahl M., Baysse C. (2015). The Antibacterial Properties of Isothiocyanates. Microbiology.

[30] Romeo L., Iori R., Rollin P., Bramanti P., Mazzon E. (2018). Isothiocyanates: An Overview of Their Antimicrobial Activity Against Human Infections. Molecules.

[31] de Figueiredo S.M., Filho S.A., Nogueira-Machado J.A., Caligiorne R.B. (2013). The Anti-oxidant Properties of Isothiocyanates: A Review. Recent Pat. Endocr. Metab. Immune. Drug Discov..

[32] Habtemariam S. (2024). Anti-Inflammatory Therapeutic Mechanisms of Isothiocyanates: Insights from Sulforaphane. Biomedicines.

[33] Khan F., Joshi A., Devkota H.P., Subramaniyan V., Kumarasamy V., Arora J. (2023). Dietary Glucosinolates Derived Isothiocyanates: Chemical Properties, Metabolism and Their Potential in Prevention of Alzheimer’s disease. Front. Pharmacol..

[34] Kamal R.M., Abdull Razis A.F., Mohd Sukri N.S., Perimal E.K., Ahmad H., Rollin P., Djedaini-Pilard F., Mazzon E., Rigaud S. (2022). Beneficial Health Effects of Glucosinolates- Derived Isothiocyanates on Cardiovascular and Neurodegenerative Diseases. Molecules.

[35] Burčul F., Generalić Mekinić I., Radan M., Rollin P., Blažević I. (2018). Isothiocyanates: Cholinesterase Inhibiting, Antioxidant, and Anti-inflammatory Activity. J. Enzym. Inhib. Med. Chem..

[36] Silva C.F.M., Guerrinha A.P.D.d.M.S., Carvalho S., Pinto D.C.G.A., Silva A.M.S. (2025). 1,3,5-Triazine: A Promising Molecular Scaffold for Novel Agents for the Treatment of Alzheimer’s Disease. Int. J. Mol. Sci..

[37] Gharat R., Prabhu A., Khambete M.P. (2022). Potential of Triazines in Alzheimer’s Disease: A Versatile Privileged Scaffold. Arch. Pharm..

[38] Wróbel A., Kolesińska B., Frączyk J., Kamiński Z.J., Tankiewicz-Kwedlo A., Hermanowicz J., Czarnomysy R., Maliszewski D., Drozdowska D. (2020). Synthesis and Cellular Effects of Novel 1,3,5-Triazine Derivatives in DLD and Ht-29 Human Colon Cancer Cell Lines. Investig. New Drugs..

[39] Dai Q., Sun Q., Ouyang X., Liu J., Jin L., Liu A., He B., Fan T., Jiang Y. (2023). Antitumor Activity of s-Triazine Derivatives: A Systematic Review. Molecules.

[40] Rathod B., Pawar S., Puri S., Diwan A., Kumar K. (2024). Recent Advancements and Developments in the Biological Importance of 1,3,5-Triazines. ChemistrySelect.

[41] Davila Ceron V., Illicachi L.A., Insuasty B. (2023). Triazine: An Important Building Block of Organic Materials for Solar Cell Application. Molecules.

[42] Maliszewski D., Drozdowska D. (2022). Recent Advances in the Biological Activity of s-Triazine Core Compounds. Pharmaceuticals.

[43] Zou J.P., Zhang X.Q., Guo Q.W., Xu X.H., Liu W.W., Zhang F., Shi D.H. (2024). Synthesis, Biological Activity, X-ray Crystallographic, DFT Calculations and Molecular Dynamics Simulation Studies of 2-phenylthiazole-1,3,5-triazine Derivatives as Potential Cholinesterase Inhibitors. J. Mol. Struct..

[44] Maliszewski D., Wróbel A., Kolesińska B., Frączyk J., Drozdowska D. (2021). 1,3,5-Triazine Nitrogen Mustards with Different Peptide Group as Innovative Candidates for AChE and BACE1 Inhibitors. Molecules.

[45] Kułaga D., Drabczyk A.K., Zaręba P., Jaśkowska J., Satała G., Zaręba P., Więckowska A., de Candia M., Purgatorio R., Boguszewska-Czubara A. (2025). Discovery of New Dual Butyrylcholinesterase (Buche) Inhibitors and 5-HT_7_ Receptor Antagonists as Compounds Used to Treat Alzheimer’s Disease Symptoms. Biomed. Pharmacother..

[46] Psurski M., Janczewski Ł., Świtalska M., Gajda A., Goszczyński T.M., Oleksyszyn J., Wietrzyk J., Gajda T. (2017). Novel Phosphonate Analogs of Sulforaphane: Synthesis, In Vitro and In Vivo Activity. Eur. J. Med. Chem..

[47] Rudzinska-Radecka M., Janczewski Ł., Gajda A., Godlewska M., Chmielewska–krzesinska M., Waskowicz K., Podlasz P. (2021). The Anti-Tumoral Potential of Phosphonate Analog of Sulforaphane in Zebrafish Xenograft Model. Cells.

[48] Xu Y., Li M.J., Greenblatt H., Chen W., Paz A., Dym O., Peleg Y., Chen T., Shen X., He J. (2012). Flexibility of the Flap in the Active Site of BACE1 as Revealed by Crystal Structures and Molecular Dynamics Simulations. Acta Crystallogr. D Biol. Crystallogr..

[49] Cheung J., Rudolph M.J., Burshteyn F., Cassidy M.S., Gary E.N., Love J., Franklin M.C., Height J.J. (2012). Structures of Human Acetylcholinesterase in Complex with Pharmacologically Important Ligands. J. Med. Chem..

[50] Landrum G. RDKit: Open-Source Cheminformatics. http://www.rdkit.org.

[51] Koes D. RDConf: Low-Energy Ligand Conformer Search. https://github.com/dkoes/rdkit-scripts.

[52] (2026). The Galaxy Community, Galaxy for accessible, reproducible, and collaborative data analyses: 2026 update. Nucleic Acids Res..

[53] Riniker S., Landrum G.A. (2015). Better Informed Distance Geometry: Using What We Know to Improve Conformation Generation. J. Chem. Inf. Model..

[54] Brink T., Exner T.E. (2010). pKa based protonation states and microspecies from protein-ligand docking. J. Comput. Aided. Mol. Des..

[55] Korb O., Stützle T., Exner T.E. (2009). Empirical Scoring Functions for Advanced Protein−Ligand Docking with PLANTS. J. Chem. Inf. Model..

[56] Korb O., Stützle T., Exner T.E., Dorigo M., Gambardella L.M., Birattari M., Martinoli A., Poli R., Stützle T. (2006). PLANTS: Application of Ant Colony Optimization to Structure-Based Drug Design. Ant Colony Optimization and Swarm Intelligence.

[57] Korb O., Stützle T., Exner T.E. (2007). An Ant Colony Optimization Approach to Flexible Protein–Ligand Docking. Swarm Intell..

[58] Schake P., Bolz S.N., Linnemann K., Schroeder M. (2025). PLIP 2025: Introducing protein-protein interactions to the protein-ligand interaction profiler. Nucleic Acids Res..

[59] ADMET-AI. https://admet.ai.greenstonebio.com/.

[60] Swanson K., Walther P., Leitz J., Mukherjee S., Wu J.C., Shivnaraine R.V., Zou J. (2024). ADMET-AI: A Machine learning ADMET Platform for Evaluation of Large-Scale Chemical Libraries. Bioinformatics.

[61] Kolesińska B., Kamiński Z.J. (2009). The Umpolung of Substituent Effect in Nucleophilic Aromatic Substitution. A New Approach to the Synthesis of N,N-disubstituted Melamines (triazine triskelions) Under Mild Reaction Conditions. Tetrahedron.

[62] Kolesińska B., Kamiński Z.J. (2008). Transformation of Tertiary Amines into Alkylating Reagents by Treatment with 2-chloro-4,6- dimethoxy-1,3,5-triazine. A Synthetic Application of Side-reaction Accompanying Coupling by Means of 4-(4,6-dimethoxy-[1,3,5] triazin-2-yl)-4-methyl-morpholin-4-ium Chloride (DMTMM). Pol. J. Chem..

[63] Janczewski Ł., Gajda A., Braszczyńska J., Gajda T. (2016). Microwave-assisted Synthesis of Dialkyl ω-azidoalkylphosphonates. Synth. Commun..

[64] Hu H., Chen Z., Xu X., Xu Y. (2019). Structure-Based Survey of the Binding Modes of BACE1 Inhibitors. ACS Chem. Neurosci..

[65] Maqbool M., Manral A., Jameel E., Kumar J., Saini V., Shandilya A., Tiwari M., Hoda N., Jayaram B. (2016). Development of cyanopyridine-triazine hybrids as lead multitarget anti-Alzheimer agents. Bioorg. Med. Chem..

[66] Jameel E., Meena P., Maqbool M., Kumar J., Ahmed W., Mumtazuddin S., Tiwari M., Hoda N., Jayaram B. (2017). Rational design, synthesis and biological screening of triazine-triazolopyrimidine hybrids as multitarget anti-Alzheimer agents. Eur. J. Med. Chem..

[67] Zhang X., Wang J., Zou J., Cao Y., Xu X., Ding B., Liu W., Ma S., Shi D. (2024). Design, Synthesis and Anticholinesterase Activity of Coumarin-1,3,5-triazine Derivatives. ChemistrySelect.

[68] Wu W.-L., Wen Z.-Y., Qian J.-J., Zou J.-P., Liu S.-M., Yang S., Qin T., Yang Q., Liu Y.-H., Liu W.-W. (2022). Design, Synthesis, Characterization and Evaluation of 1,3,5-Triazine-Benzimidazole Hybrids as Multifunctional Acetylcholinesterases Inhibitors. J. Mol. Struct..

[69] Zou J., Qian J., Liu S., Li R., Zhang X., Yang S., Liu Y., Liu W., Ma S., Shi D. (2022). Design, Synthesis, Biological Evaluation and Molecular Dynamics Simulations Study of Genistein-O-1,3,5-Triazine Derivatives as Multifunctional Anti-Alzheimer Agents. ChemistrySelect.

[70] Su J., Wu W., Dong C.-E., Yang S., Feng Y., Qin T., Chen K., Qian J., Zou J., Liu Y.-H. (2023). Synthesis, Characterization, Crystal Structure and Biological Evaluation of 1,3,5-Triazine-Quinoline Derivatives as Butyrylcholinesterase Inhibitors. J. Mol. Struct..

[71] Karimian S., Shekouhy M., Pirhadi S., Iraji A., Attarroshan M., Edraki N., Khoshneviszadeh M. (2022). Synthesis and Biological Evaluation of Benzimidazoles/1,3,5-Triazine-2,4-Diamine Hybrid Compounds: A New Class of Multifunctional Alzheimer Targeting Agents. New J. Chem..

[72] Xu W., Chen G., Liew O.W., Zuo Z., Jiang H., Zhu W. (2009). Novel Non-Peptide β-Secretase Inhibitors Derived from Structure-Based Virtual Screening and Bioassay. Bioorg. Med. Chem. Lett..

[73] Xu W., Chen G., Zhu W., Zuo Z. (2010). Molecular Docking and Structure-Activity Relationship Studies on Benzothiazole Based Non-Peptidic BACE-1 Inhibitors. Bioorg. Med. Chem. Lett..

[74] Hrabinova M., Pejchal J., Kucera T., Jun D., Schmidt M., Soukup O. (2021). Is It the Twilight of BACE1 Inhibitors?. Curr. Neuropharmacol..

[75] Prati F., Bottegoni G., Bolognesi M.L., Cavalli A. (2018). BACE-1 Inhibitors: From Recent Single-Target Molecules to Multitarget Compounds for Alzheimer’s Disease. J. Med. Chem..

[76] Patel D., Kumari P., Patel N. (2012). Synthesis and Biological Evaluation of Some Thiazolidinones as Antimicrobial Agents. Eur. J. Med. Chem..

[77] Sole R., Agostinis L., Conca S., Gatto V., Bardella N., Morandini A., Buranello C., Beghetto V. (2021). Synthesis of Amidation Agents and Their Reactivity in Condensation Reactions. Synthesis.

[78] Bakharev V.V., Gidaspov A.A., Parfenov V.E., Ul’yankina I.V., Zavodskaya A.V., Selezneva E.V., Suponitskii K.Y., Sheremetev A.B. (2012). Synthesis of 4-amino-6-chloro-1,3,5-triazin-2(1H)-ones. Russ. Chem. Bull..

[79] Kumari P., Kaur S., Kaur J., Bhatti R., Singh P. (2020). Modification of the Lead Molecule: Tryptophan and Piperidine Appended Triazines Reversing Inflammation and Hyperalgesia in Rats. Bioorg. Med. Chem..

[80] Khalil A.F., El-Moselhy T.F., El-Bastawissy E.A., Abdelhady R., Younis N.S., El-Hamamsy M.H. (2023). Discovery of Novel Enasidenib Analogues Targeting Inhibition of Mutant Isocitrate Dehydrogenase 2 as Antileukaemic Agents. J. Enzym. Inhib. Med. Chem..

[81] d’Atri G., Gomarasca A., Resnati G., Tronconi G., Scolastico C., Sirtori C.R. (1984). Novel Pyrimidine and 1,3,5-Triazine Hypolipidemic Agents. J. Med. Chem..

[82] Matsuno T., Kato M., Tsuchida Y., Takahashi M., Yaguchi S., Terada S. (1997). Synthesis and Aromatase-Inhibitory Activity of Imidazoly-1,3,5-Triazine Derivatives. Chem. Pharm. Bull..

[83] Lemos B.C., Westphal R., Filho E.V., Fiorot R.G., Carneiro J.W.M., Gomes A.C.C., Guimarães C.J., de Oliveira F.C.E., Costa P.M.S., Pessoa C. (2021). Synthetic Enamine Naphthoquinone Derived from Lawsone as Cytotoxic Agents Assessed by In vitro and In silico Evaluations. Bioorg. Med. Chem. Lett..

[84] Sharma A., Jad Y., Siddiqui M.R.H., de la Torre B.G., Albericio F., El-Faham A. (2017). Synthesis, Characterization, and Tautomerism of 1,3-Dimethyl Pyrimidine-2,4,6-trione S-triazinyl Hydrazine/Hydrazone Derivatives. J. Chem..

[85] Dovlatyan V.V., Éliazyan K.A., Pivazyan V.A., Akopyan A.M. (1993). Reactions of Derivatives of Amino- and Mercapto-sym-triazines with Ethyleneimine and Ethylenediamine. Chem. Heterocycl. Compd..

[86] Cloete T.T., de Kock C., Smith P.J., N’Da D.D. (2014). Synthesis, In vitro Antiplasmodial Activity and Cytotoxicity of a Series of Artemisinin–triazine Hybrids and Hybrid-dimers. Eur. J. Med. Chem..

