# Supplementary material for: Design, Synthesis, and Biological Activity of Isothiocyanate-Triazine Conjugates as AChE and BACE1 Inhibitors
Source: Biomolecules. 2026 Sep 3;16(9):1280. doi: 10.3390/biom16091280 (PMC13604150; doi:10.3390/biom16091280)
Supplement: Supplementary file 1 [file biomolecules-16-01280-s001.zip › ADMET/Compound 1a.pdf]

Molecule 1: O=P(CCCCCCN=C=S)(NCCNC1=NC(N(C)C)=NC(N(C…

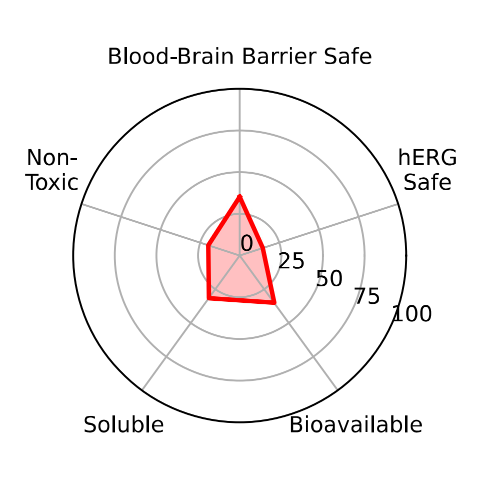

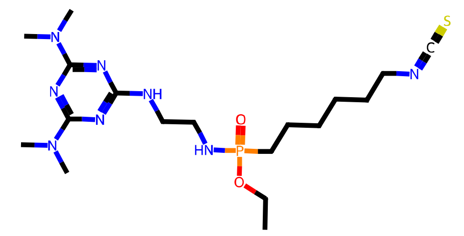

Physicochemical

Property	Value	DrugBank Percentile	Units
**Molecular Weight (https://www.rdkit.org/docs/source/rdkit.Chem.Descriptors.html#rdkit.Chem.Descriptors.MolWt)**	458.57	76.81%	Dalton
**LogP (https://www.rdkit.org/docs/source/rdkit.Chem.Crippen.html#rdkit.Chem.Crippen.MolLogP)**	2.90	60.41%	log-ratio
**Hydrogen Bond Acceptors (https://www.rdkit.org/docs/source/rdkit.Chem.rdMolDescriptors.html#rdkit.Chem.rdMolDescriptors.CalcNumHBA)**	10.00	88.91%	#
**Hydrogen Bond Donors (https://www.rdkit.org/docs/source/rdkit.Chem.rdMolDescriptors.html#rdkit.Chem.rdMolDescriptors.CalcNumHBD)**	2.00	60.86%	#
**Lipinski Rule of 5 (https://en.wikipedia.org/wiki/Lipinski%27s_rule_of_five#Components_of_the_rule)**	4.00	63.80%	# of 4
**Quantitative Estimate of Druglikeness (QED) (https://www.rdkit.org/docs/source/rdkit.Chem.QED.html#rdkit.Chem.QED.qed)**	0.17	9.85%	-
**Stereo Centers (https://www.rdkit.org/docs/source/rdkit.Chem.rdMolDescriptors.html#rdkit.Chem.rdMolDescriptors.CalcNumAtomStereoCenters)**	1.00	54.81%	#
**Topological Polar Surface Area (TPSA) (https://www.rdkit.org/docs/source/rdkit.Chem.rdMolDescriptors.html#rdkit.Chem.rdMolDescriptors.CalcTPSA)**	107.87	72.20%	$Å^2$

Absorption

Property	Prediction	DrugBank Percentile	Units
**Human Intestinal Absorption (https://tdcommons.ai/single_pred_tasks/adme/#hia-human-intestinal-absorption-hou-et-al)**	0.99	45.60%	-
**Oral Bioavailability (https://tdcommons.ai/single_pred_tasks/adme/#bioavailability-ma-et-al)**	0.69	35.09%	-
**Aqueous Solubility (https://tdcommons.ai/single_pred_tasks/adme/#solubility-aqsoldb)**	-4.01	31.56%	log(mol/L)
**Lipophilicity (https://tdcommons.ai/single_pred_tasks/adme/#lipophilicity-astrazeneca)**	2.13	62.82%	log-ratio
**Hydration Free Energy (https://tdcommons.ai/single_pred_tasks/adme/#hydration-free-energy-freesolv)**	-7.32	72.74%	kcal/mol

Property	Prediction	DrugBank Percentile	Units
Cell Effective Permeability (https://tdcommons.ai/single_pred_tasks/adme/#caco-2-cell-effective-permeability-wang-et-al)	-5.31	31.29%	log(10^{-6} cm/s)
PAMPA Permeability (https://tdcommons.ai/single_pred_tasks/adme/#pampa-permeability-ncats)	0.97	87.48%	-
P-glycoprotein Inhibition (https://tdcommons.ai/single_pred_tasks/adme/#pgp-p-glycoprotein-inhibition-broccatelli-et-al)	0.45	73.01%	-

Distribution

Property	Prediction	DrugBank Percentile	Units
Blood-Brain Barrier Penetration (https://tdcommons.ai/single_pred_tasks/adme/#bbb-blood-brain-barrier-martins-et-al)	0.88	64.87%	-
Plasma Protein Binding Rate (https://tdcommons.ai/single_pred_tasks/adme/#ppbr-plasma-protein-binding-rate-astrazeneca)	77.56	53.12%	%
Volume of Distribution at Steady State (https://tdcommons.ai/single_pred_tasks/adme/#vdss-volumn-of-distribution-at-steady-state-lombardo-et-al)	8.24	87.67%	L/kg

Metabolism

Property	Prediction	DrugBank Percentile	Units
CYP1A2 Inhibition (https://tdcommons.ai/single_pred_tasks/adme/#cyp-p450-1a2-inhibition-veith-et-al)	0.16	67.12%	-
CYP2C19 Inhibition (https://tdcommons.ai/single_pred_tasks/adme/#cyp-p450-2c19-inhibition-veith-et-al)	0.80	94.42%	-
CYP2C9 Inhibition (https://tdcommons.ai/single_pred_tasks/adme/#cyp-p450-2c9-inhibition-veith-et-al)	0.77	95.93%	-
CYP2D6 Inhibition (https://tdcommons.ai/single_pred_tasks/adme/#cyp-p450-2d6-inhibition-veith-et-al)	0.29	78.05%	-
CYP3A4 Inhibition (https://tdcommons.ai/single_pred_tasks/adme/#cyp-p450-3a4-inhibition-veith-et-al)	0.85	92.75%	-
CYP2C9 Substrate (https://tdcommons.ai/single_pred_tasks/adme/#cyp2c9-substrate-carbon-mangels-et-al)	0.27	73.90%	-
CYP2D6 Substrate (https://tdcommons.ai/single_pred_tasks/adme/#cyp2d6-substrate-carbon-mangels-et-al)	0.38	79.26%	-
CYP3A4 Substrate (https://tdcommons.ai/single_pred_tasks/adme/#cyp3a4-substrate-carbon-mangels-et-al)	0.81	89.84%	-

Excretion

Property	Prediction	DrugBank Percentile	Units
Half Life (https://tdcommons.ai/single_pred_tasks/adme/#half-life-obach-et-al)	55.19	88.56%	hr
Drug Clearance (Hepatocyte) (https://tdcommons.ai/single_pred_tasks/adme/#clearance-astrazeneca)	78.86	81.89%	uL/min/10^6 cells
Drug Clearance (Microsome) (https://tdcommons.ai/single_pred_tasks/adme/#clearance-astrazeneca)	76.27	89.49%	uL/min/mg

Toxicity

Property	Prediction	DrugBank Percentile	Units
hERG Blocking (https://tdcommons.ai/single_pred_tasks/tox/#herg-blockers)	0.89	85.50%	-
Clinical Toxicity (https://tdcommons.ai/single_pred_tasks/tox/#clintox)	0.36	80.19%	-
Mutagenicity (https://tdcommons.ai/single_pred_tasks/tox/#ames-mutagenicity)	0.23	56.38%	-
Drug Induced Liver Injury (https://tdcommons.ai/single_pred_tasks/tox/#dili-drug-induced-liver-injury)	0.73	66.69%	-
Carcinogenicity (https://tdcommons.ai/single_pred_tasks/tox/#carcinogens)	0.32	74.87%	-
Acute Toxicity LD50 (https://tdcommons.ai/single_pred_tasks/tox/#acute-toxicity-ld50)	2.83	69.52%	log(1/(mol/kg))
Skin Reaction (https://tdcommons.ai/single_pred_tasks/tox/#skin-reaction)	0.62	68.09%	-
Androgen Receptor (Full Length) (https://tdcommons.ai/single_pred_tasks/tox/#tox21)	0.01	20.94%	-
Androgen Receptor (Ligand Binding Domain) (https://tdcommons.ai/single_pred_tasks/tox/#tox21)	0.01	33.58%	-
Aryl Hydrocarbon Receptor (https://tdcommons.ai/single_pred_tasks/tox/#tox21)	0.26	85.34%	-
Aromatase (https://tdcommons.ai/single_pred_tasks/tox/#tox21)	0.17	82.94%	-
Estrogen Receptor (Full Length) (https://tdcommons.ai/single_pred_tasks/tox/#tox21)	0.04	16.48%	-
Estrogen Receptor (Ligand Binding Domain) (https://tdcommons.ai/single_pred_tasks/tox/#tox21)	0.03	67.16%	-
Peroxisome Proliferator-Activated Receptor Gamma (https://tdcommons.ai/single_pred_tasks/tox/#tox21)	0.02	71.93%	-
Nuclear Factor (Erythroid-Derived 2)-Like 2/Antioxidant Responsive Element (https://tdcommons.ai/single_pred_tasks/tox/#tox21)	0.53	85.23%	-
ATPase Family AAA Domain-Containing Protein 5 (ATAD5) (https://tdcommons.ai/single_pred_tasks/tox/#tox21)	0.03	72.39%	-
Heat Shock Factor Response Element (https://tdcommons.ai/single_pred_tasks/tox/#tox21)	0.17	90.46%	-
Mitochondrial Membrane Potential (https://tdcommons.ai/single_pred_tasks/tox/#tox21)	0.44	83.33%	-
Tumor Protein p53 (https://tdcommons.ai/single_pred_tasks/tox/#tox21)	0.14	79.91%	-
