# Supplementary material for: Design, Synthesis, and Biological Activity of Isothiocyanate-Triazine Conjugates as AChE and BACE1 Inhibitors
Source: Biomolecules. 2026 Sep 3;16(9):1280. doi: 10.3390/biom16091280 (PMC13604150; doi:10.3390/biom16091280)
Supplement: Supplementary file 1 [file biomolecules-16-01280-s001.zip › ADMET/Compound 1d.pdf]

Molecule 1: O=P(CCCCCCN=C=S)(NCCNC1=NC(N2CCCC2)=NC(…

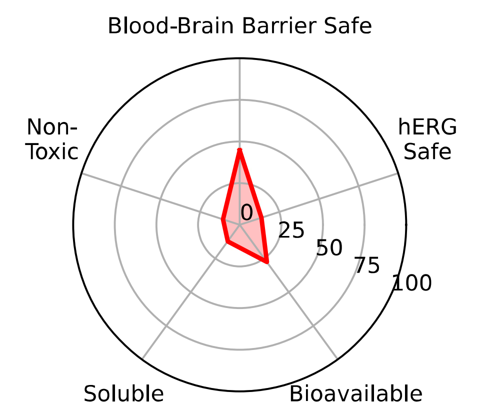

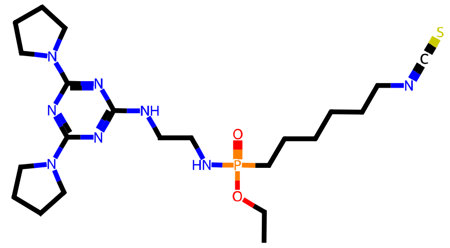

Physicochemical

Property	Value	DrugBank Percentile	Units
**Molecular Weight (https://www.rdkit.org/docs/source/rdkit.Chem.Descriptors.html#rdkit.Chem.Descriptors.MolWt)**	510.65	82.75%	Dalton
**LogP (https://www.rdkit.org/docs/source/rdkit.Chem.Crippen.html#rdkit.Chem.Crippen.MolLogP)**	3.97	77.16%	log-ratio
**Hydrogen Bond Acceptors (https://www.rdkit.org/docs/source/rdkit.Chem.rdMolDescriptors.html#rdkit.Chem.rdMolDescriptors.CalcNumHBA)**	10.00	88.91%	#
**Hydrogen Bond Donors (https://www.rdkit.org/docs/source/rdkit.Chem.rdMolDescriptors.html#rdkit.Chem.rdMolDescriptors.CalcNumHBD)**	2.00	60.86%	#
**Lipinski Rule of 5 (https://en.wikipedia.org/wiki/Lipinski%27s_rule_of_five#Components_of_the_rule)**	3.00	20.92%	# of 4
**Quantitative Estimate of Druglikeness (QED) (https://www.rdkit.org/docs/source/rdkit.Chem.QED.html#rdkit.Chem.QED.qed)**	0.15	8.61%	-
**Stereo Centers (https://www.rdkit.org/docs/source/rdkit.Chem.rdMolDescriptors.html#rdkit.Chem.rdMolDescriptors.CalcNumAtomStereoCenters)**	1.00	54.81%	#
**Topological Polar Surface Area (TPSA) (https://www.rdkit.org/docs/source/rdkit.Chem.rdMolDescriptors.html#rdkit.Chem.rdMolDescriptors.CalcTPSA)**	107.87	72.20%	$Å^2$

Absorption

Property	Prediction	DrugBank Percentile	Units
**Human Intestinal Absorption (https://tdcommons.ai/single_pred_tasks/adme/#hia-human-intestinal-absorption-hou-et-al)** ?	1.00	57.15%	-
**Oral Bioavailability (https://tdcommons.ai/single_pred_tasks/adme/#bioavailability-ma-et-al)** ?	0.62	27.69%	-
**Aqueous Solubility (https://tdcommons.ai/single_pred_tasks/adme/#solubility-aqsoldb)** ?	-5.15	12.33%	log(mol/L)
**Lipophilicity (https://tdcommons.ai/single_pred_tasks/adme/#lipophilicity-astrazeneca)** ?	2.91	78.40%	log-ratio
**Hydration Free Energy (https://tdcommons.ai/single_pred_tasks/adme/#hydration-free-energy-freesolv)** ?	-7.21	73.56%	kcal/mol

Property	Prediction	DrugBank Percentile	Units
Cell Effective Permeability (https://tdcommons.ai/single_pred_tasks/adme/#caco-2-cell-effective-permeability-wang-et-al)	-5.24	34.39%	log(10^{-6} cm/s)
PAMPA Permeability (https://tdcommons.ai/single_pred_tasks/adme/#pampa-permeability-ncats)	0.95	81.47%	-
P-glycoprotein Inhibition (https://tdcommons.ai/single_pred_tasks/adme/#pgp-p-glycoprotein-inhibition-broccatelli-et-al)	0.66	82.47%	-

Distribution

Property	Prediction	DrugBank Percentile	Units
Blood-Brain Barrier Penetration (https://tdcommons.ai/single_pred_tasks/adme/#bbb-blood-brain-barrier-martins-et-al)	0.80	55.41%	-
Plasma Protein Binding Rate (https://tdcommons.ai/single_pred_tasks/adme/#ppbr-plasma-protein-binding-rate-astrazeneca)	91.84	76.97%	%
Volume of Distribution at Steady State (https://tdcommons.ai/single_pred_tasks/adme/#vdss-volumn-of-distribution-at-steady-state-lombardo-et-al)	7.72	86.12%	L/kg

Metabolism

Property	Prediction	DrugBank Percentile	Units
CYP1A2 Inhibition (https://tdcommons.ai/single_pred_tasks/adme/#cyp-p450-1a2-inhibition-veith-et-al)	0.22	71.73%	-
CYP2C19 Inhibition (https://tdcommons.ai/single_pred_tasks/adme/#cyp-p450-2c19-inhibition-veith-et-al)	0.84	95.54%	-
CYP2C9 Inhibition (https://tdcommons.ai/single_pred_tasks/adme/#cyp-p450-2c9-inhibition-veith-et-al)	0.82	96.94%	-
CYP2D6 Inhibition (https://tdcommons.ai/single_pred_tasks/adme/#cyp-p450-2d6-inhibition-veith-et-al)	0.42	83.44%	-
CYP3A4 Inhibition (https://tdcommons.ai/single_pred_tasks/adme/#cyp-p450-3a4-inhibition-veith-et-al)	0.89	94.57%	-
CYP2C9 Substrate (https://tdcommons.ai/single_pred_tasks/adme/#cyp2c9-substrate-carbon-mangels-et-al)	0.16	51.84%	-
CYP2D6 Substrate (https://tdcommons.ai/single_pred_tasks/adme/#cyp2d6-substrate-carbon-mangels-et-al)	0.36	78.13%	-
CYP3A4 Substrate (https://tdcommons.ai/single_pred_tasks/adme/#cyp3a4-substrate-carbon-mangels-et-al)	0.79	87.05%	-

Excretion

Property	Prediction	DrugBank Percentile	Units
Half Life (https://tdcommons.ai/single_pred_tasks/adme/#half-life-obach-et-al)	35.09	79.41%	hr
Drug Clearance (Hepatocyte) (https://tdcommons.ai/single_pred_tasks/adme/#clearance-astrazeneca)	65.39	73.75%	uL/min/10^6 cells
Drug Clearance (Microsome) (https://tdcommons.ai/single_pred_tasks/adme/#clearance-astrazeneca)	79.09	90.35%	uL/min/mg

Toxicity

Property	Prediction	DrugBank Percentile	Units
hERG Blocking (https://tdcommons.ai/single_pred_tasks/tox/#herg-blockers)	0.89	86.35%	-
Clinical Toxicity (https://tdcommons.ai/single_pred_tasks/tox/#clintox)	0.51	89.45%	-
Mutagenicity (https://tdcommons.ai/single_pred_tasks/tox/#ames-mutagenicity)	0.35	71.73%	-
Drug Induced Liver Injury (https://tdcommons.ai/single_pred_tasks/tox/#dili-drug-induced-liver-injury)	0.82	73.40%	-
Carcinogenicity (https://tdcommons.ai/single_pred_tasks/tox/#carcinogens)	0.20	58.32%	-
Acute Toxicity LD50 (https://tdcommons.ai/single_pred_tasks/tox/#acute-toxicity-ld50)	2.81	68.48%	log(1/(mol/kg))
Skin Reaction (https://tdcommons.ai/single_pred_tasks/tox/#skin-reaction)	0.68	73.87%	-
Androgen Receptor (Full Length) (https://tdcommons.ai/single_pred_tasks/tox/#tox21)	0.01	18.11%	-
Androgen Receptor (Ligand Binding Domain) (https://tdcommons.ai/single_pred_tasks/tox/#tox21)	4.41e-03	29.43%	-
Aryl Hydrocarbon Receptor (https://tdcommons.ai/single_pred_tasks/tox/#tox21)	0.27	85.77%	-
Aromatase (https://tdcommons.ai/single_pred_tasks/tox/#tox21)	0.24	87.63%	-
Estrogen Receptor (Full Length) (https://tdcommons.ai/single_pred_tasks/tox/#tox21)	0.07	36.56%	-
Estrogen Receptor (Ligand Binding Domain) (https://tdcommons.ai/single_pred_tasks/tox/#tox21)	0.04	70.69%	-
Peroxisome Proliferator-Activated Receptor Gamma (https://tdcommons.ai/single_pred_tasks/tox/#tox21)	0.04	81.12%	-
Nuclear Factor (Erythroid-Derived 2)-Like 2/Antioxidant Responsive Element (https://tdcommons.ai/single_pred_tasks/tox/#tox21)	0.66	91.12%	-
ATPase Family AAA Domain-Containing Protein 5 (ATAD5) (https://tdcommons.ai/single_pred_tasks/tox/#tox21)	0.07	84.84%	-
Heat Shock Factor Response Element (https://tdcommons.ai/single_pred_tasks/tox/#tox21)	0.28	94.38%	-
Mitochondrial Membrane Potential (https://tdcommons.ai/single_pred_tasks/tox/#tox21)	0.63	90.23%	-
Tumor Protein p53 (https://tdcommons.ai/single_pred_tasks/tox/#tox21)	0.33	91.70%	-
