# Supplementary material for: Design, Synthesis, and Biological Activity of Isothiocyanate-Triazine Conjugates as AChE and BACE1 Inhibitors
Source: Biomolecules. 2026 Sep 3;16(9):1280. doi: 10.3390/biom16091280 (PMC13604150; doi:10.3390/biom16091280)
Supplement: Supplementary file 1 [file biomolecules-16-01280-s001.zip › ADMET/Compound 1e.pdf]

Molecule 1: O=P(CCCCCCN=C=S)(NCCNC1=NC(N2CCCCC2)=N…

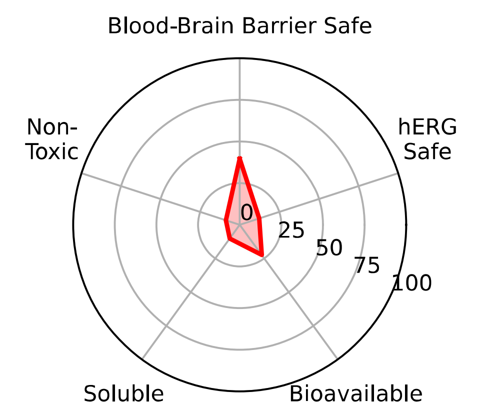

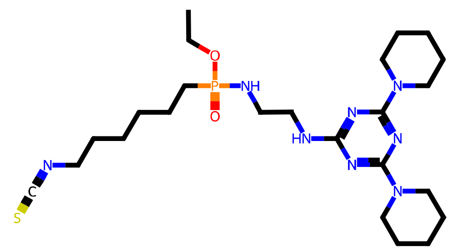

Physicochemical

Property	Value	DrugBank Percentile	Units
Molecular Weight (https://www.rdkit.org/docs/source/rdkit.Chem.Descriptors.html#rdkit.Chem.Descriptors.MolWt)	538.70	85.07%	Dalton
LogP (https://www.rdkit.org/docs/source/rdkit.Chem.Crippen.html#rdkit.Chem.Crippen.MolLogP)	4.75	86.62%	log-ratio
Hydrogen Bond Acceptors (https://www.rdkit.org/docs/source/rdkit.Chem.rdMolDescriptors.html#rdkit.Chem.rdMolDescriptors.CalcNumHBA)	10.00	88.91%	#
Hydrogen Bond Donors (https://www.rdkit.org/docs/source/rdkit.Chem.rdMolDescriptors.html#rdkit.Chem.rdMolDescriptors.CalcNumHBD)	2.00	60.86%	#
Lipinski Rule of 5 (https://en.wikipedia.org/wiki/Lipinski%27s_rule_of_five#Components_of_the_rule)	3.00	20.92%	# of 4
Quantitative Estimate of Druglikeness (QED) (https://www.rdkit.org/docs/source/rdkit.Chem.QED.html#rdkit.Chem.QED.qed)	0.13	7.37%	-
Stereo Centers (https://www.rdkit.org/docs/source/rdkit.Chem.rdMolDescriptors.html#rdkit.Chem.rdMolDescriptors.CalcNumAtomStereoCenters)	1.00	54.81%	#
Topological Polar Surface Area (TPSA) (https://www.rdkit.org/docs/source/rdkit.Chem.rdMolDescriptors.html#rdkit.Chem.rdMolDescriptors.CalcTPSA)	107.87	72.20%	$Å^2$

Absorption

Property	Prediction	DrugBank Percentile	Units
Human Intestinal Absorption (https://tdcommons.ai/single_pred_tasks/adme/#hia-human-intestinal-absorption-hou-et-al) ?	1.00	61.81%	-
Oral Bioavailability (https://tdcommons.ai/single_pred_tasks/adme/#bioavailability-ma-et-al) ?	0.55	22.45%	-
Aqueous Solubility (https://tdcommons.ai/single_pred_tasks/adme/#solubility-aqsoldb) ?	-5.36	10.20%	log(mol/L)
Lipophilicity (https://tdcommons.ai/single_pred_tasks/adme/#lipophilicity-astrazeneca) ?	3.23	85.50%	log-ratio
Hydration Free Energy (https://tdcommons.ai/single_pred_tasks/adme/#hydration-free-energy-freesolv) ?	-6.42	80.11%	kcal/mol

Property	Prediction	DrugBank Percentile	Units
Cell Effective Permeability (https://tdcommons.ai/single_pred_tasks/adme/#caco-2-cell-effective-permeability-wang-et-al)	-5.17	38.35%	log(10^{-6} cm/s)
PAMPA Permeability (https://tdcommons.ai/single_pred_tasks/adme/#pampa-permeability-ncats)	0.96	83.56%	-
P-glycoprotein Inhibition (https://tdcommons.ai/single_pred_tasks/adme/#pgp-p-glycoprotein-inhibition-broccatelli-et-al)	0.71	83.99%	-

Distribution

Property	Prediction	DrugBank Percentile	Units
Blood-Brain Barrier Penetration (https://tdcommons.ai/single_pred_tasks/adme/#bbb-blood-brain-barrier-martins-et-al)	0.84	60.45%	-
Plasma Protein Binding Rate (https://tdcommons.ai/single_pred_tasks/adme/#ppbr-plasma-protein-binding-rate-astrazeneca)	97.47	87.05%	%
Volume of Distribution at Steady State (https://tdcommons.ai/single_pred_tasks/adme/#vdss-volumn-of-distribution-at-steady-state-lombardo-et-al)	7.46	85.34%	L/kg

Metabolism

Property	Prediction	DrugBank Percentile	Units
CYP1A2 Inhibition (https://tdcommons.ai/single_pred_tasks/adme/#cyp-p450-1a2-inhibition-veith-et-al)	0.22	71.27%	-
CYP2C19 Inhibition (https://tdcommons.ai/single_pred_tasks/adme/#cyp-p450-2c19-inhibition-veith-et-al)	0.88	96.32%	-
CYP2C9 Inhibition (https://tdcommons.ai/single_pred_tasks/adme/#cyp-p450-2c9-inhibition-veith-et-al)	0.79	96.39%	-
CYP2D6 Inhibition (https://tdcommons.ai/single_pred_tasks/adme/#cyp-p450-2d6-inhibition-veith-et-al)	0.55	87.05%	-
CYP3A4 Inhibition (https://tdcommons.ai/single_pred_tasks/adme/#cyp-p450-3a4-inhibition-veith-et-al)	0.89	94.69%	-
CYP2C9 Substrate (https://tdcommons.ai/single_pred_tasks/adme/#cyp2c9-substrate-carbon-mangels-et-al)	0.11	38.89%	-
CYP2D6 Substrate (https://tdcommons.ai/single_pred_tasks/adme/#cyp2d6-substrate-carbon-mangels-et-al)	0.35	77.67%	-
CYP3A4 Substrate (https://tdcommons.ai/single_pred_tasks/adme/#cyp3a4-substrate-carbon-mangels-et-al)	0.80	87.17%	-

Excretion

Property	Prediction	DrugBank Percentile	Units
**Half Life (https://tdcommons.ai/single_pred_tasks/adme/#half-life-obach-et-al)**	30.76	76.11%	hr
**Drug Clearance (Hepatocyte) (https://tdcommons.ai/single_pred_tasks/adme/#clearance-astrazeneca)**	56.11	67.43%	uL/min/10^6 cells
**Drug Clearance (Microsome) (https://tdcommons.ai/single_pred_tasks/adme/#clearance-astrazeneca)**	78.44	90.07%	uL/min/mg

Toxicity

Property	Prediction	DrugBank Percentile	Units
**hERG Blocking (https://tdcommons.ai/single_pred_tasks/tox/#herg-blockers)**	0.91	87.82%	-
**Clinical Toxicity (https://tdcommons.ai/single_pred_tasks/tox/#clintox)**	0.54	91.31%	-
**Mutagenicity (https://tdcommons.ai/single_pred_tasks/tox/#ames-mutagenicity)**	0.28	64.56%	-
**Drug Induced Liver Injury (https://tdcommons.ai/single_pred_tasks/tox/#dili-drug-induced-liver-injury)**	0.76	68.32%	-
**Carcinogenicity (https://tdcommons.ai/single_pred_tasks/tox/#carcinogens)**	0.18	54.09%	-
**Acute Toxicity LD50 (https://tdcommons.ai/single_pred_tasks/tox/#acute-toxicity-ld50)**	2.65	57.85%	log(1/(mol/kg))
**Skin Reaction (https://tdcommons.ai/single_pred_tasks/tox/#skin-reaction)**	0.73	78.67%	-
**Androgen Receptor (Full Length) (https://tdcommons.ai/single_pred_tasks/tox/#tox21)**	4.71e-03	12.76%	-
**Androgen Receptor (Ligand Binding Domain) (https://tdcommons.ai/single_pred_tasks/tox/#tox21)**	2.92e-03	22.33%	-
**Aryl Hydrocarbon Receptor (https://tdcommons.ai/single_pred_tasks/tox/#tox21)**	0.32	87.51%	-
**Aromatase (https://tdcommons.ai/single_pred_tasks/tox/#tox21)**	0.27	89.45%	-
**Estrogen Receptor (Full Length) (https://tdcommons.ai/single_pred_tasks/tox/#tox21)**	0.07	37.92%	-
**Estrogen Receptor (Ligand Binding Domain) (https://tdcommons.ai/single_pred_tasks/tox/#tox21)**	0.03	67.89%	-
**Peroxisome Proliferator-Activated Receptor Gamma (https://tdcommons.ai/single_pred_tasks/tox/#tox21)**	0.03	75.88%	-
**Nuclear Factor (Erythroid-Derived 2)-Like 2/Antioxidant Responsive Element (https://tdcommons.ai/single_pred_tasks/tox/#tox21)**	0.69	91.93%	-
**ATPase Family AAA Domain-Containing Protein 5 (ATAD5) (https://tdcommons.ai/single_pred_tasks/tox/#tox21)**	0.05	81.12%	-
**Heat Shock Factor Response Element (https://tdcommons.ai/single_pred_tasks/tox/#tox21)**	0.35	95.39%	-
**Mitochondrial Membrane Potential (https://tdcommons.ai/single_pred_tasks/tox/#tox21)**	0.64	90.58%	-
**Tumor Protein p53 (https://tdcommons.ai/single_pred_tasks/tox/#tox21)**	0.25	88.41%	-
