# Supplementary material for: Design, Synthesis, and Biological Activity of Isothiocyanate-Triazine Conjugates as AChE and BACE1 Inhibitors
Source: Biomolecules. 2026 Sep 3;16(9):1280. doi: 10.3390/biom16091280 (PMC13604150; doi:10.3390/biom16091280)
Supplement: Supplementary file 1 [file biomolecules-16-01280-s001.zip › ADMET/Compound 1j.pdf]

Molecule 1: O=P(CCCCCCN=C=S)(NCCNC1=NC(N2CCOCC2)=N…

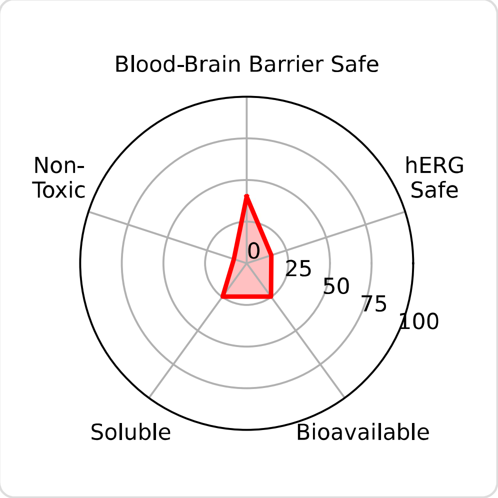

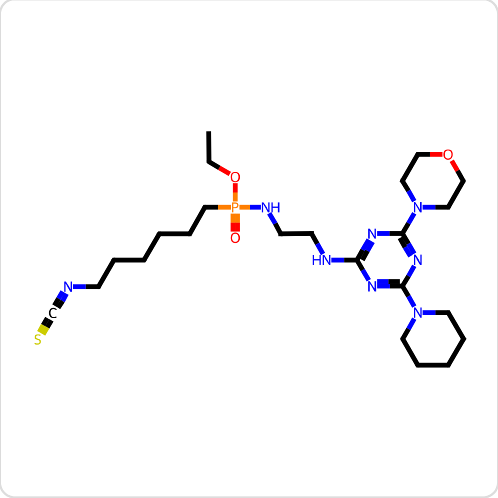

Physicochemical

Property	Value	DrugBank Percentile	Units
**Molecular Weight (https://www.rdkit.org/docs/source/rdkit.Chem.Descriptors.html#rdkit.Chem.Descriptors.MolWt)**	540.68	85.23%	Dalton
**LogP (https://www.rdkit.org/docs/source/rdkit.Chem.Crippen.html#rdkit.Chem.Crippen.MolLogP)**	3.59	71.23%	log-ratio
**Hydrogen Bond Acceptors (https://www.rdkit.org/docs/source/rdkit.Chem.rdMolDescriptors.html#rdkit.Chem.rdMolDescriptors.CalcNumHBA)**	11.00	90.73%	#
**Hydrogen Bond Donors (https://www.rdkit.org/docs/source/rdkit.Chem.rdMolDescriptors.html#rdkit.Chem.rdMolDescriptors.CalcNumHBD)**	2.00	60.86%	#
**Lipinski Rule of 5 (https://en.wikipedia.org/wiki/Lipinski%27s_rule_of_five#Components_of_the_rule)**	2.00	9.85%	# of 4
**Quantitative Estimate of Druglikeness (QED) (https://www.rdkit.org/docs/source/rdkit.Chem.QED.html#rdkit.Chem.QED.qed)**	0.14	7.87%	-
**Stereo Centers (https://www.rdkit.org/docs/source/rdkit.Chem.rdMolDescriptors.html#rdkit.Chem.rdMolDescriptors.CalcNumAtomStereoCenters)**	1.00	54.81%	#
**Topological Polar Surface Area (TPSA) (https://www.rdkit.org/docs/source/rdkit.Chem.rdMolDescriptors.html#rdkit.Chem.rdMolDescriptors.CalcTPSA)**	117.10	76.39%	$Å^2$

Absorption

Property	Prediction	DrugBank Percentile	Units
**Human Intestinal Absorption (https://tdcommons.ai/single_pred_tasks/adme/#hia-human-intestinal-absorption-hou-et-al)**	1.00	52.62%	-
**Oral Bioavailability (https://tdcommons.ai/single_pred_tasks/adme/#bioavailability-ma-et-al)**	0.59	24.78%	-
**Aqueous Solubility (https://tdcommons.ai/single_pred_tasks/adme/#solubility-aqsoldb)**	-4.36	24.70%	log(mol/L)
**Lipophilicity (https://tdcommons.ai/single_pred_tasks/adme/#lipophilicity-astrazeneca)**	2.79	75.84%	log-ratio
**Hydration Free Energy (https://tdcommons.ai/single_pred_tasks/adme/#hydration-free-energy-freesolv)**	-7.07	74.45%	kcal/mol

Property	Prediction	DrugBank Percentile	Units
**Cell Effective Permeability (https://tdcommons.ai/single_pred_tasks/adme/#caco-2-cell-effective-permeability-wang-et-al)**	-5.08	43.85%	log(10^{-6} cm/s)
**PAMPA Permeability (https://tdcommons.ai/single_pred_tasks/adme/#pampa-permeability-ncats)**	0.95	80.38%	-
**P-glycoprotein Inhibition (https://tdcommons.ai/single_pred_tasks/adme/#pgp-p-glycoprotein-inhibition-broccatelli-et-al)**	0.61	80.34%	-

Distribution

Property	Prediction	DrugBank Percentile	Units
**Blood-Brain Barrier Penetration (https://tdcommons.ai/single_pred_tasks/adme/#bbb-blood-brain-barrier-martins-et-al)**	0.84	59.98%	-
**Plasma Protein Binding Rate (https://tdcommons.ai/single_pred_tasks/adme/#ppbr-plasma-protein-binding-rate-astrazeneca)**	87.96	69.76%	%
**Volume of Distribution at Steady State (https://tdcommons.ai/single_pred_tasks/adme/#vdss-volumn-of-distribution-at-steady-state-lombardo-et-al)**	5.95	79.80%	L/kg

Metabolism

Property	Prediction	DrugBank Percentile	Units
**CYP1A2 Inhibition (https://tdcommons.ai/single_pred_tasks/adme/#cyp-p450-1a2-inhibition-veith-et-al)**	0.11	61.92%	-
**CYP2C19 Inhibition (https://tdcommons.ai/single_pred_tasks/adme/#cyp-p450-2c19-inhibition-veith-et-al)**	0.87	96.16%	-
**CYP2C9 Inhibition (https://tdcommons.ai/single_pred_tasks/adme/#cyp-p450-2c9-inhibition-veith-et-al)**	0.82	96.94%	-
**CYP2D6 Inhibition (https://tdcommons.ai/single_pred_tasks/adme/#cyp-p450-2d6-inhibition-veith-et-al)**	0.36	81.81%	-
**CYP3A4 Inhibition (https://tdcommons.ai/single_pred_tasks/adme/#cyp-p450-3a4-inhibition-veith-et-al)**	0.86	93.33%	-
**CYP2C9 Substrate (https://tdcommons.ai/single_pred_tasks/adme/#cyp2c9-substrate-carbon-mangels-et-al)**	0.09	31.60%	-
**CYP2D6 Substrate (https://tdcommons.ai/single_pred_tasks/adme/#cyp2d6-substrate-carbon-mangels-et-al)**	0.28	73.32%	-
**CYP3A4 Substrate (https://tdcommons.ai/single_pred_tasks/adme/#cyp3a4-substrate-carbon-mangels-et-al)**	0.79	86.04%	-

Excretion

Property	Prediction	DrugBank Percentile	Units
Half Life (https://tdcommons.ai/single_pred_tasks/adme/#half-life-obach-et-al)	5.03	46.53%	hr
Drug Clearance (Hepatocyte) (https://tdcommons.ai/single_pred_tasks/adme/#clearance-astrazeneca)	58.22	69.25%	uL/min/10^6 cells
Drug Clearance (Microsome) (https://tdcommons.ai/single_pred_tasks/adme/#clearance-astrazeneca)	66.74	85.92%	uL/min/mg

Toxicity

Property	Prediction	DrugBank Percentile	Units
hERG Blocking (https://tdcommons.ai/single_pred_tasks/tox/#herg-blockers)	0.88	84.49%	-
Clinical Toxicity (https://tdcommons.ai/single_pred_tasks/tox/#clintox)	0.56	91.78%	-
Mutagenicity (https://tdcommons.ai/single_pred_tasks/tox/#ames-mutagenicity)	0.32	68.40%	-
Drug Induced Liver Injury (https://tdcommons.ai/single_pred_tasks/tox/#dili-drug-induced-liver-injury)	0.76	68.90%	-
Carcinogenicity (https://tdcommons.ai/single_pred_tasks/tox/#carcinogens)	0.13	44.67%	-
Acute Toxicity LD50 (https://tdcommons.ai/single_pred_tasks/tox/#acute-toxicity-ld50)	2.71	62.00%	log(1/(mol/kg))
Skin Reaction (https://tdcommons.ai/single_pred_tasks/tox/#skin-reaction)	0.62	68.36%	-
Androgen Receptor (Full Length) (https://tdcommons.ai/single_pred_tasks/tox/#tox21)	0.01	15.24%	-
Androgen Receptor (Ligand Binding Domain) (https://tdcommons.ai/single_pred_tasks/tox/#tox21)	3.38e-03	24.74%	-
Aryl Hydrocarbon Receptor (https://tdcommons.ai/single_pred_tasks/tox/#tox21)	0.33	88.37%	-
Aromatase (https://tdcommons.ai/single_pred_tasks/tox/#tox21)	0.24	87.86%	-
Estrogen Receptor (Full Length) (https://tdcommons.ai/single_pred_tasks/tox/#tox21)	0.07	36.37%	-
Estrogen Receptor (Ligand Binding Domain) (https://tdcommons.ai/single_pred_tasks/tox/#tox21)	0.04	71.81%	-
Peroxisome Proliferator-Activated Receptor Gamma (https://tdcommons.ai/single_pred_tasks/tox/#tox21)	0.03	76.54%	-
Nuclear Factor (Erythroid-Derived 2)-Like 2/Antioxidant Responsive Element (https://tdcommons.ai/single_pred_tasks/tox/#tox21)	0.66	90.97%	-
ATPase Family AAA Domain-Containing Protein 5 (ATAD5) (https://tdcommons.ai/single_pred_tasks/tox/#tox21)	0.09	86.47%	-
Heat Shock Factor Response Element (https://tdcommons.ai/single_pred_tasks/tox/#tox21)	0.30	94.84%	-
Mitochondrial Membrane Potential (https://tdcommons.ai/single_pred_tasks/tox/#tox21)	0.59	88.87%	-
Tumor Protein p53 (https://tdcommons.ai/single_pred_tasks/tox/#tox21)	0.35	92.13%	-
