# Supplementary material for: Design, Synthesis, and Biological Activity of Isothiocyanate-Triazine Conjugates as AChE and BACE1 Inhibitors
Source: Biomolecules. 2026 Sep 3;16(9):1280. doi: 10.3390/biom16091280 (PMC13604150; doi:10.3390/biom16091280)
Supplement: Supplementary file 1 [file biomolecules-16-01280-s001.zip › ADMET/Compound 1k.pdf]

Molecule 1: O=P(CCCCCCN=C=S)(NCCCCCCNC1=NC(N(C)C)=N…

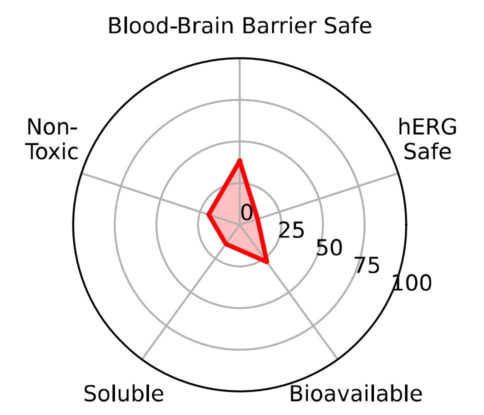

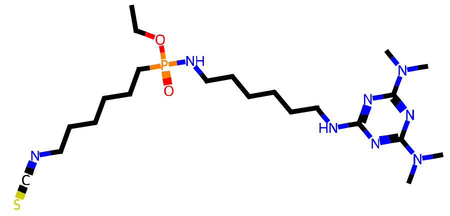

Physicochemical

Property	Value	DrugBank Percentile	Units
**Molecular Weight (https://www.rdkit.org/docs/source/rdkit.Chem.Descriptors.html#rdkit.Chem.Descriptors.MolWt)**	514.68	83.02%	Dalton
**LogP (https://www.rdkit.org/docs/source/rdkit.Chem.Crippen.html#rdkit.Chem.Crippen.MolLogP)**	4.46	83.56%	log-ratio
**Hydrogen Bond Acceptors (https://www.rdkit.org/docs/source/rdkit.Chem.rdMolDescriptors.html#rdkit.Chem.rdMolDescriptors.CalcNumHBA)**	10.00	88.91%	#
**Hydrogen Bond Donors (https://www.rdkit.org/docs/source/rdkit.Chem.rdMolDescriptors.html#rdkit.Chem.rdMolDescriptors.CalcNumHBD)**	2.00	60.86%	#
**Lipinski Rule of 5 (https://en.wikipedia.org/wiki/Lipinski%27s_rule_of_five#Components_of_the_rule)**	3.00	20.92%	# of 4
**Quantitative Estimate of Druglikeness (QED) (https://www.rdkit.org/docs/source/rdkit.Chem.QED.html#rdkit.Chem.QED.qed)**	0.11	6.24%	-
**Stereo Centers (https://www.rdkit.org/docs/source/rdkit.Chem.rdMolDescriptors.html#rdkit.Chem.rdMolDescriptors.CalcNumAtomStereoCenters)**	1.00	54.81%	#
**Topological Polar Surface Area (TPSA) (https://www.rdkit.org/docs/source/rdkit.Chem.rdMolDescriptors.html#rdkit.Chem.rdMolDescriptors.CalcTPSA)**	107.87	72.20%	$Å^2$

Absorption

Property	Prediction	DrugBank Percentile	Units
**Human Intestinal Absorption (https://tdcommons.ai/single_pred_tasks/adme/#hia-human-intestinal-absorption-hou-et-al)**	0.99	48.27%	-
**Oral Bioavailability (https://tdcommons.ai/single_pred_tasks/adme/#bioavailability-ma-et-al)**	0.62	27.69%	-
**Aqueous Solubility (https://tdcommons.ai/single_pred_tasks/adme/#solubility-aqsoldb)**	-5.00	14.31%	log(mol/L)
**Lipophilicity (https://tdcommons.ai/single_pred_tasks/adme/#lipophilicity-astrazeneca)**	2.74	74.80%	log-ratio
**Hydration Free Energy (https://tdcommons.ai/single_pred_tasks/adme/#hydration-free-energy-freesolv)**	-6.31	80.77%	kcal/mol

Property	Prediction	DrugBank Percentile	Units
**Cell Effective Permeability (https://tdcommons.ai/single_pred_tasks/adme/#caco-2-cell-effective-permeability-wang-et-al)**	-5.23	35.28%	$\log(10^{-6}$ cm/s)
**PAMPA Permeability (https://tdcommons.ai/single_pred_tasks/adme/#pampa-permeability-ncats)**	0.96	83.21%	-
**P-glycoprotein Inhibition (https://tdcommons.ai/single_pred_tasks/adme/#pgp-p-glycoprotein-inhibition-broccatelli-et-al)**	0.71	84.22%	-

Distribution

Property	Prediction	DrugBank Percentile	Units
**Blood-Brain Barrier Penetration (https://tdcommons.ai/single_pred_tasks/adme/#bbb-blood-brain-barrier-martins-et-al)**	0.85	61.81%	-
**Plasma Protein Binding Rate (https://tdcommons.ai/single_pred_tasks/adme/#ppbr-plasma-protein-binding-rate-astrazeneca)**	87.98	69.76%	%
**Volume of Distribution at Steady State (https://tdcommons.ai/single_pred_tasks/adme/#vdss-volumn-of-distribution-at-steady-state-lombardo-et-al)**	12.13	94.03%	L/kg

Metabolism

Property	Prediction	DrugBank Percentile	Units
**CYP1A2 Inhibition (https://tdcommons.ai/single_pred_tasks/adme/#cyp-p450-1a2-inhibition-veith-et-al)**	0.13	64.06%	-
**CYP2C19 Inhibition (https://tdcommons.ai/single_pred_tasks/adme/#cyp-p450-2c19-inhibition-veith-et-al)**	0.81	94.46%	-
**CYP2C9 Inhibition (https://tdcommons.ai/single_pred_tasks/adme/#cyp-p450-2c9-inhibition-veith-et-al)**	0.76	95.85%	-
**CYP2D6 Inhibition (https://tdcommons.ai/single_pred_tasks/adme/#cyp-p450-2d6-inhibition-veith-et-al)**	0.35	81.04%	-
**CYP3A4 Inhibition (https://tdcommons.ai/single_pred_tasks/adme/#cyp-p450-3a4-inhibition-veith-et-al)**	0.86	93.29%	-
**CYP2C9 Substrate (https://tdcommons.ai/single_pred_tasks/adme/#cyp2c9-substrate-carbon-mangels-et-al)**	0.26	72.04%	-
**CYP2D6 Substrate (https://tdcommons.ai/single_pred_tasks/adme/#cyp2d6-substrate-carbon-mangels-et-al)**	0.35	77.47%	-
**CYP3A4 Substrate (https://tdcommons.ai/single_pred_tasks/adme/#cyp3a4-substrate-carbon-mangels-et-al)**	0.84	93.99%	-

Excretion

Property	Prediction	DrugBank Percentile	Units
**Half Life (https://tdcommons.ai/single_pred_tasks/adme/#half-life-obach-et-al)**	81.79	93.80%	hr
**Drug Clearance (Hepatocyte) (https://tdcommons.ai/single_pred_tasks/adme/#clearance-astrazeneca)**	73.76	79.22%	uL/min/10^6 cells
**Drug Clearance (Microsome) (https://tdcommons.ai/single_pred_tasks/adme/#clearance-astrazeneca)**	83.12	91.86%	uL/min/mg

Toxicity

Property	Prediction	DrugBank Percentile	Units
**hERG Blocking (https://tdcommons.ai/single_pred_tasks/tox/#herg-blockers)**	0.92	88.72%	-
**Clinical Toxicity (https://tdcommons.ai/single_pred_tasks/tox/#clintox)**	0.37	80.42%	-
**Mutagenicity (https://tdcommons.ai/single_pred_tasks/tox/#ames-mutagenicity)**	0.19	50.64%	-
**Drug Induced Liver Injury (https://tdcommons.ai/single_pred_tasks/tox/#dili-drug-induced-liver-injury)**	0.69	64.29%	-
**Carcinogenicity (https://tdcommons.ai/single_pred_tasks/tox/#carcinogens)**	0.35	78.40%	-
**Acute Toxicity LD50 (https://tdcommons.ai/single_pred_tasks/tox/#acute-toxicity-ld50)**	2.74	63.71%	log(1/(mol/kg))
**Skin Reaction (https://tdcommons.ai/single_pred_tasks/tox/#skin-reaction)**	0.66	72.24%	-
**Androgen Receptor (Full Length) (https://tdcommons.ai/single_pred_tasks/tox/#tox21)**	0.01	18.22%	-
**Androgen Receptor (Ligand Binding Domain) (https://tdcommons.ai/single_pred_tasks/tox/#tox21)**	4.34e-03	29.24%	-
**Aryl Hydrocarbon Receptor (https://tdcommons.ai/single_pred_tasks/tox/#tox21)**	0.22	83.06%	-
**Aromatase (https://tdcommons.ai/single_pred_tasks/tox/#tox21)**	0.18	83.64%	-
**Estrogen Receptor (Full Length) (https://tdcommons.ai/single_pred_tasks/tox/#tox21)**	0.04	15.47%	-
**Estrogen Receptor (Ligand Binding Domain) (https://tdcommons.ai/single_pred_tasks/tox/#tox21)**	0.03	63.75%	-
**Peroxisome Proliferator-Activated Receptor Gamma (https://tdcommons.ai/single_pred_tasks/tox/#tox21)**	0.02	72.94%	-
**Nuclear Factor (Erythroid-Derived 2)-Like 2/Antioxidant Responsive Element (https://tdcommons.ai/single_pred_tasks/tox/#tox21)**	0.54	85.96%	-
**ATPase Family AAA Domain-Containing Protein 5 (ATAD5) (https://tdcommons.ai/single_pred_tasks/tox/#tox21)**	0.02	70.53%	-
**Heat Shock Factor Response Element (https://tdcommons.ai/single_pred_tasks/tox/#tox21)**	0.19	91.78%	-
**Mitochondrial Membrane Potential (https://tdcommons.ai/single_pred_tasks/tox/#tox21)**	0.55	87.51%	-
**Tumor Protein p53 (https://tdcommons.ai/single_pred_tasks/tox/#tox21)**	0.15	81.16%	-
