# Supplementary material for: Design, Synthesis, and Biological Activity of Isothiocyanate-Triazine Conjugates as AChE and BACE1 Inhibitors
Source: Biomolecules. 2026 Sep 3;16(9):1280. doi: 10.3390/biom16091280 (PMC13604150; doi:10.3390/biom16091280)
Supplement: Supplementary file 1 [file biomolecules-16-01280-s001.zip › biomolecules-4386773-supplementary.pdf]

Supporting Information

Design, Synthesis, and Biological Activity of Isothiocyanate–Triazine Conjugates as AChE and BACE1 Inhibitors

Kacper Górecki [1], Jakub Polakowski [1], Natalia Więckowska [1], Renata Grzywa [2], Justyna Frączyk [1], Beata Kolesińska [1], Agnieszka Wróbel-Tałałaj [3], Danuta Drozdowska [3], Łukasz Janczewski [1*]

1 Institute of Organic Chemistry, Faculty of Chemistry, Lodz University of Technology, 116 Żeromskiego Str., 90-924 Łódź, Poland
2 Department of Organic and Medicinal Chemistry, Faculty of Chemistry, Wrocław University of Science and Technology, Wybrzeże Wyspiańskiego 27, 50-370 Wrocław, Poland
3 Department of Organic Chemistry, Medical University, 2a Mickiewicza Str., 15-222 Białystok, Poland

**Corresponding author - e-mail: lukasz.janczewski@p.lodz.pl*

Table of contents

S1. The analytical data for compounds 3a–f 6

S1.1. 4,6-dichloro-N,N-dimethyl-1,3,5-triazin-2-amine (**3a**) 6

S1.2. 4,6-dichloro-N,N-diethyl-1,3,5-triazin-2-amine (**3b**) 6

S1.3. 2-(azetidin-1-yl)-4,6-dichloro-1,3,5-triazine (**3c**) 6

S1.4. 2,4-dichloro-6-(pyrrolidin-1-yl)-1,3,5-triazine (**3d**) 6

S1.5. 2,4-dichloro-6-(piperidin-1-yl)-1,3,5-triazine (**3e**) 6

S1.6. 4-(4,6-dichloro-1,3,5-triazin-2-yl)morpholine (**3f**) 6

S2. The analytical data for compounds 4a–j 7

S2.1. 6-chloro-N2,N2,N4,N4-tetramethyl-1,3,5-triazine-2,4-diamine (**4a**) 7

S2.2. 6-chloro-N2,N2,N4,N4-tetraethyl-1,3,5-triazine-2,4-diamine (**4b**) 7

S2.3. 2,4-di(azetidin-1-yl)-6-chloro-1,3,5-triazine (**4c**) 7

S2.4. 2- chloro-4,6-di(pyrrolidin-1-yl)-1,3,5-triazine (**4d**) 7

S2.5. 2-chloro-4,6-di(piperidin-1-yl)-1,3,5-triazine (**4e**) 7

S2.6. 4,4'-(6-chloro-1,3,5-triazine-2,4-diyl)dimorpholine (**4f**) 8

S2.7. 2-chloro-N,N-dimethyl-6-morpholino-1,3,5-triazin-2-amine (**4g**) 8

S2.8. 4-chloro-N,N-dimethyl-6-(piperidin-1-yl)-1,3,5-triazin-2-amine (**4h**) 8

S2.9. 4-(azetidin-1-yl)-6-chloro-N,N-dimethyl-1,3,5-triazin-2-amine (**4i**) 8

S2.10. 4-(4-chloro-6-(piperidin-1-yl)-1,3,5-triazin-2-yl)morpholine (**4j**) 8

S3. The analytical data for compounds 5a–j. 9

S3.1. Compound **5a** ... 9
S3.2. Compound **5b** ... 9
S3.3. Compound **5c** ... 9
S3.4. Compound **5d** ... 9
S3.5. Compound **5e** ... 9
S3.6. Compound **5f** ... 10
S3.7. Compound **5g** ... 10
S3.8. Compound **5h** ... 10
S3.9. Compound **5i** ... 10
S3.10. Compound **5j** ... 10
S3.11. Compound **5k** ... 11
S4. The analytical data for compound 6 ... 11
S5. The analytical data for compound 7 ... 11
S6. The analytical data for compounds 8a-k ... 11
S6.1. Compound **8a** ... 11
S6.2. Compound **8b** ... 12
S6.3. Compound **8c** ... 12
S6.4. Compound **8d** ... 12
S6.5. Compound **8e** ... 13
S6.6. Compound **8f** ... 13
S6.7. Compound **8g** ... 13
S6.8. Compound **8h** ... 14
S6.9. Compound **8i** ... 14
S6.10. Compound **8j** ... 14
S6.11. Compound **8k** ... 15
S7. The analytical data for compounds 1a-k ... 15
S7.1. Isothiocyanate-triazine conjugate **1a** ... 15
S7.2. Isothiocyanate-triazine conjugate **1b** ... 15
S7.3. Isothiocyanate-triazine conjugate **1c** ... 16
S7.4. Isothiocyanate-triazine conjugate **1d** ... 16
S7.5. Isothiocyanate-triazine conjugate **1e** ... 16
S7.6. Isothiocyanate-triazine conjugate **1f** ... 17

S7.7. Isothiocyanate-triazine conjugate **1g** 17
S7.8. Isothiocyanate-triazine conjugate **1h** 18
S7.9. Isothiocyanate-triazine conjugate **1i** 18
S7.10. Isothiocyanate-triazine conjugate **1j** 18
S7.11. Isothiocyanate-triazine conjugate **1k** 19
S8. References 19
S9. Analytical spectra of 7 21
S10. Analytical spectra of 3a 23
S11. Analytical spectra of 3b 25
S12. Analytical spectra of 3c 27
S13. Analytical spectra of 3d 29
S14. Analytical spectra of 3e 31
S15. Analytical spectra of 3f 33
S16. Analytical spectra of 4a 35
S17. Analytical spectra of 4b 37
S18. Analytical spectra of 4c 39
S19. Analytical spectra of 4d 41
S20. Analytical spectra of 4e 43
S21. Analytical spectra of 4f 45
S22. Analytical spectra of 4g 47
S23. Analytical spectra of 4h 49
S24. Analytical spectra of 4i 51
S25. Analytical spectra of 4j 53
S26. Analytical spectra of 5a 55
S27. Analytical spectra of 5b 57
S28. Analytical spectra of 5c 59
S29. Analytical spectra of 5d 61
S30. Analytical spectra of 5e 63
S31. Analytical spectra of 5f 65
S32. Analytical spectra of 5g 67
S33. Analytical spectra of 5h 69
S34. Analytical spectra of 5i 71

S35. Analytical spectra of 5j 72
S36. Analytical spectra of 5k 74
S37. Analytical spectra of 8a 76
S38. Analytical spectra of 8b 79
S39. Analytical spectra of 8c 81
S40. Analytical spectra of 8d 84
S41. Analytical spectra of 8e 86
S42. Analytical spectra of 8f 88
S43. Analytical spectra of 8g 91
S44. Analytical spectra of 8h 94
S45. Analytical spectra of 8i 97
S46. Analytical spectra of 8j 100
S47. Analytical spectra of 8k 103
S48. Analytical spectra of 1a 105
S49. Analytical spectra of 1b 108
S50. Analytical spectra of 1c 111
S51. Analytical spectra of 1d 115
S52. Analytical spectra of 1e 118
S53. Analytical spectra of 1f 121
S54. Analytical spectra of 1g 125
S55. Analytical spectra of 1h 128
S56. Analytical spectra of 1i 131
S57. Analytical spectra of 1j 134
S58. Analytical spectra of 1k 137
Fig. S218. Dose–response curves on AChE for compounds 1a 140
Fig. S219. Dose–response curves on AChE for compounds 1b 141
Fig. S220. Dose–response curves on AChE for compounds 1c 142
Fig. S221. Dose–response curves on AChE for compounds 1d 143
Fig. S222. Dose–response curves on AChE for compounds 1e 144
Fig. S223. Dose–response curves on AChE for compounds 1f 145
Fig. S224. Dose–response curves on AChE for compounds 1g 146
Fig. S225. Dose–response curves on AChE for compounds 1h 147

Fig. S226. Dose–response curves on AChE for compounds 1i 148

Fig. S227. Dose–response curves on AChE for compounds 1j 149

Fig. S228. Dose–response curves on AChE for compounds 1k 150

Fig. S229. Dose–response curves on BACE-1 for compounds 1a 151

Fig. S230. Dose–response curves on BACE-1 for compounds 1b 152

Fig. S231. Dose–response curves on BACE-1 for compounds 1c 153

Fig. S232. Dose–response curves on BACE-1 for compounds 1d 154

Fig. S233. Dose–response curves on BACE-1 for compounds 1e 155

Fig. S234. Dose–response curves on BACE-1 for compounds 1f 156

Fig. S235. Dose–response curves on BACE-1 for compounds 1g 157

Fig. S236. Dose–response curves on BACE-1 for compounds 1h 158

Fig. S237. Dose–response curves on BACE-1 for compounds 1i 159

Fig. S238. Dose–response curves on BACE-1 for compounds 1j 160

Fig. S239. Dose–response curves on BACE-1 for compounds 1k 161

Fig. S240. Re-docking poses of AChE and BACE1 ligands aligned with their respective crystal structures. 163

Fig. S241. Docking pose of quercetin in the BACE1 binding cavity. 164

Fig. S242. Representative docking poses and protein–ligand interaction patterns for compounds 1a–1k in the active site of acetylcholinesterase (AChE; PDB ID: 4EY7). 168

Fig. S243. Representative docking poses and protein–ligand interaction patterns for compounds 1A–1K in the active site of human beta-secretase 1 (BACE1; PDB ID: 3UQW) 172

S1. The analytical data for compounds 3a–f

S1.1. 4,6-dichloro-N,N-dimethyl-1,3,5-triazin-2-amine (**3a**)

Scale of **2** 10 mmol. White solid, mp. 124–126 °C (lit. [1] 126–128 °C). Yield 85% (1.641 g). Purity > 99% (t_R = 5.6 min). **^{1}H NMR** ($CDCl_3$, 400 MHz) δ: 3.22 (s, 6 H, N(C*H*$_3$)$_2$). **^{13}C NMR** ($CDCl_3$, 176 MHz) δ: 169.9 (s, 2 C, 2 × C_{Ar}Cl), 164.8 (s, 1 C, C_{Ar}N(CH_3)$_2$), 37.0 (s, 2 C, 2 × *C*H$_3$). **HRMS**: *m/z* [M+H]$^+$ $C_5H_7Cl_2N_4^+$ calcd 193.0042, found 193.0058.

S1.2. 4,6-dichloro-N,N-diethyl-1,3,5-triazin-2-amine (**3b**)

Scale of **2** 5 mmol. White solid, mp. 77–79 °C (lit. [2] 77 °C). Yield 86% (1.017 g). Purity 92% (t_R = 4.4 min). **^{1}H NMR** ($CDCl_3$, 700 MHz) δ: 3.61 (q, J_{HH} = 7.1 Hz, 4 H, 2 × C*H*$_2$), 1.20 (t, J_{HH} = 7.1 Hz, 6 H, 2 × C*H*$_3$). **^{13}C NMR** ($CDCl_3$, 176 MHz) δ: 170.0 (s, 2 C, 2 × C_{Ar}Cl), 163.9 (s, 1 C, C_{Ar}N), 42.6 (s, 2 C, 2 × *C*H$_2$), 12.7 (s, 2 C, 2 × *C*H$_3$). **HRMS**: *m/z* [M+H]$^+$ $C_7H_{11}Cl_2N_4^+$ calcd 221.0355, found 221.0360.

S1.3. 2-(azetidin-1-yl)-4,6-dichloro-1,3,5-triazine (**3c**)

Scale of **2** 5 mmol. Yellowish solid, mp. 115–117 °C. Yield 62% (0.624 g). Purity ≥ 99% (t_R = 5.2 min). **^{1}H NMR** ($CDCl_3$, 700 MHz) δ: 4.25 (t, J_{HH} = 7.7 Hz, 4 H, 2 × C*H*$_2$N), 2.45 (qn, J_{HH} = 7.8 Hz, 2 H, C*H*$_2$). **^{13}C NMR** ($CDCl_3$, 176 MHz) δ: 170.1 (s, 2 C, 2 × C_{Ar}Cl), 163.5 (s, 1 C, C_{Ar}N), 50.1 (s, 2 C, 2 × *C*H$_2$N), 16.2 (s, 1 C, *C*H$_2$). **HRMS**: *m/z* [M+H]$^+$ $C_6H_7Cl_2N_4^+$ calcd 205.0042, found 205.0050

S1.4. 2,4-dichloro-6-(pyrrolidin-1-yl)-1,3,5-triazine (**3d**)

Scale of **2** 5 mmol. White solid, mp. 105–106 °C (lit. [3] 93–95 °C). Yield 62% (0.695 g). Purity 96% (t_R = 6.1 min). **^{1}H NMR** ($CDCl_3$, 700 MHz) δ: 3.61 (t, J_{HH} = 6.9 Hz, 4 H, 2 × C*H*$_2$), 2.00 (t, J_{HH} = 6.9 Hz, 4 H, 2 × C*H*$_2$). **^{13}C NMR** ($CDCl_3$, 176 MHz) δ: 169.7 (s, 2 C, 2 × C_{Ar}Cl), 162.5 (s, 1 C, C_{Ar}N), 47.3 (s, 2 C, 2 × *C*H$_2$), 25.1 (s, 2 C, 2 × *C*H$_2$). **HRMS**: *m/z* [M+H]$^+$ $C_7H_9Cl_2N_4^+$ calcd 219.0199, found 219.0232.

S1.5. 2,4-dichloro-6-(piperidin-1-yl)-1,3,5-triazine (**3e**)

Scale of **2** 5 mmol. White solid, mp. 84–86 °C (lit. [4] 86 °C). Yield 66% (0.788 g). Purity 95% (t_R = 6.9 min). **^{1}H NMR** ($CDCl_3$, 700 MHz) δ: 3.80 (t, J_{HH} = 5.4 Hz, 4 H, 2 × C*H*$_2$), 1.73–1.66 (m, 2 H, C*H*$_2$), 1.65–1.59 (m, 4 H, 2 × C*H*$_2$). **^{13}C NMR** ($CDCl_3$, 176 MHz) δ: 170.2 (s, 2 C, 2 × C_{Ar}Cl), 163.6 (s, 1 C, C_{Ar}N), 45.4 (s, 2 C, 2 × *C*H$_2$), 25.7 (s, 2 C, 2 × *C*H$_2$), 24.3 (s, 1 C, *C*H$_2$). **HRMS**: *m/z* [M+H]$^+$ $C_8H_{11}Cl_2N_4^+$ calcd 233.0355, found 233.0381.

S1.6. 4-(4,6-dichloro-1,3,5-triazin-2-yl)morpholine (**3f**)

Scale of **2** 40 mmol. White solid, mp. 158–160 °C (lit. [5] 159–160 °C). Yield 56% (5.237 g). Purity 90% (t_R = 5.6 min). **^{1}H NMR** ($CDCl_3$, 700 MHz) δ: 3.87 (t, J_{HH} = 5.0 Hz, 4 H, 2 × OC*H*$_2$), 3.73 (t, J_{HH} = 5.0 Hz, 4 H, 2 × NC*H*$_2$). **^{13}C NMR** ($CDCl_3$, 176 MHz) δ: 170.4 (s, 2 C, 2 × C_{Ar}Cl), 164.1 (s, 1 C, C_{Ar}N), 66.4 (s, 2 C, 2 × O*C*H$_2$), 44.5 (s, 2 C, 2 × N*C*H$_2$). **HRMS**: *m/z* [M+H]$^+$ $C_7H_9Cl_2N_4O^+$ calcd 235.0148, found 235.0085.

S2. The analytical data for compounds 4a–j

S2.1. 6-chloro-N2,N2,N4,N4-tetramethyl-1,3,5-triazine-2,4-diamine (**4a**)

Scale of **3a** 8 mmol. White solid, mp. 66–68 °C (lit. [6] 68–69 °C). Column chromatography DCM 100%. Yield 94% (1.519 g). Purity 95% (t_R = 6.1 min). **^{1}H NMR** ($CDCl_3$, 700 MHz) δ: 3.13 (s, 6 H, $N(CH_3)_2$), 3.11 (s, 6 H, $N(CH_3)_2$). **^{13}C NMR** ($CDCl_3$, 176 MHz) δ: 169.0 (s, 1 C, $C_{Ar}Cl$), 165.0 (s, 2 C, 2 × $C_{Ar}N(CH_3)_2$), 36.3 (s, 4 C, 4 × CH_3). **HRMS**: *m/z* $[M+H]^+$ $C_7H_{13}ClN_5^+$ calcd 202.0854, found 202.0887.

S2.2. 6-chloro-N2,N2,N4,N4-tetraethyl-1,3,5-triazine-2,4-diamine (**4b**)

Scale of **3b** 4.6 mmol. Yellowish oil. Column chromatography DCM 100%. Yield 85% (1.519 g). Purity 95% (t_R = 8.1 min). **^{1}H NMR** ($CDCl_3$, 700 MHz) δ: 3.52 (sx, J_{HH} = 7.5 Hz, 8 H, 4 × CH_2), 1.14 (t, J_{HH} = 6.80 Hz, 12 H, 4 × CH_3). **^{13}C NMR** ($CDCl_3$, 176 MHz) δ: 169.1 (s, 1 C, $C_{Ar}Cl$), 164.1 (s, 2 C, 2 × $C_{Ar}N$), 41.7 (s, 2 C, 2 × CH_2), 41.5 (s, 2 C, 2 × CH_2), 13.5 (s, 2 C, 2 × CH_3), 12,9 (s, 2 C, 2 × CH_3). **HRMS**: *m/z* $[M+H]^+$ $C_{11}H_{21}ClN_5^+$ calcd 258.1480, found 258.1542.

S2.3. 2,4-di(azetidin-1-yl)-6-chloro-1,3,5-triazine (**4c**)

Scale of **3c** 2.2 mmol. White solid, mp. 132–135 °C. Column chromatography DCM : acetone 3:1 (v:v). Yield 66% (0.33 g). Purity >99% (t_R = 4.8 min). **^{1}H NMR** ($CDCl_3$, 700 MHz) δ: 4.13–4.09 (m, 8 H, 4 × CH_2N), 2.32 (qn, J_{HH} = 7.5 Hz, 4 H, 2 × CH_2). **^{13}C NMR** ($CDCl_3$, 176 MHz) δ: 168.9 (s, 1 C, $C_{Ar}Cl$), 164.4 (s, 2 C, 2 × $C_{Ar}N$), 49.5 (s, 4 C, 4 × CH_2N), 16.2 (s, 2 C, 2 × CH_2). **HRMS**: *m/z* $[M+H]^+$ $C_9H_{13}ClN_5^+$ calcd 226.0854, found 226.0868.

S2.4. 2- chloro-4,6-di(pyrrolidin-1-yl)-1,3,5-triazine (**4d**)

Scale of **3d** 3.2 mmol. White solid, mp. 103–104 °C (lit. [7] 112–115 °C). Column chromatography DCM : acetone 10:1 (v:v). Yield 43% (0.350 g). Purity 95% (t_R = 7.0 min). **^{1}H NMR** ($CDCl_3$, 700 MHz) δ: 3.56–3.49 (m, 8 H, 4 × CH_2), 1.93–1.83 (m, 8 H, 4 × CH_2). **^{13}C NMR** ($CDCl_3$, 176 MHz) δ: 168.4 (s, 1 C, $C_{Ar}Cl$), 162.3 (s, 2 C, 2 × $C_{Ar}N$), 46.4 (s, 2 C, 2 × CH_2), 46.3 (s, 2 C, 2 × CH_2), 25.3 (s, 2 C, 2 × CH_2), 25.1 (s, 2 C, 2 × CH_2). **HRMS**: *m/z* $[M+H]^+$ $C_{11}H_{17}ClN_5^+$ calcd 254.1167, found 254.1195.

S2.5. 2-chloro-4,6-di(piperidin-1-yl)-1,3,5-triazine (**4e**)

Scale of **3e** 3.3 mmol. White solid, mp. 116–118 °C (lit. [8] 114–117 °C). Column chromatography DCM 100%. Yield 41% (0.377 g). Purity 94% (t_R = 8.2 min). **^{1}H NMR** ($CDCl_3$, 700 MHz) δ: 3.71 (t, J_{HH} = 5.7 Hz, 8 H, 4 × CH_2), 1.66–1.61 (m, 4 H, 2 × CH_2), 1.58–1.52 (m, 8 H, 5 × CH_2). **^{13}C NMR** ($CDCl_3$, 176 MHz) δ: 169.6 (s, 1 C, $C_{Ar}Cl$), 164.3 (s, 2 C, 2 × $C_{Ar}N$), 44.5 (s, 4 C, 4 × CH_2), 25.8 (s, 4 C, 4 × CH_2), 24.7 (s, 2 C, 2 × CH_2). **HRMS**: *m/z* $[M+H]^+$ $C_{13}H_{21}ClN_5^+$ calcd 282.1480, found 282.1471.

S2.6. 4,4'-(6-chloro-1,3,5-triazine-2,4-diyl)dimorpholine (**4f**)

Scale of **3f** 10.3 mmol. White solid, mp. 180–182 °C (lit. [8] 171–172 °C). Column chromatography DCM : acetone 10:1 (v:v). Yield 84% (2.478 g). Purity 95% (t_R = 5.5 min). **^{1}H NMR** ($CDCl_3$, 700 MHz) δ: 3.72 (t, J_{HH} = 4.9 Hz, 8 H, 4 × OCH_2), 3.67 (t, J_{HH} = 4.6 Hz, 8 H, 4 × NCH_2). **^{13}C NMR** ($CDCl_3$, 176 MHz) δ: 169.8 (s, 1 C, C_{Ar}Cl), 164.6 (s, 2 C, 2 × C_{Ar}N), 66.6 (s, 4 C, 4 × OCH_2), 43.9 (s, 4 C, 4 × NCH_2). **HRMS** : *m/z* $[M+H]^+$ $C_{11}H_{17}ClN_5O_2^+$ calcd 286.1065, found 286.1087.

S2.7. 2-chloro-N,N-dimethyl-6-morpholino-1,3,5-triazin-2-amine (**4g**)

Scale of **3g** 5 mmol. White solid, mp. 88–90 °C (lit. [7] 96–98 °C). Column chromatography DCM : acetone 10:1 (v:v). Yield 89% (1.092g). Purity 99% (t_R = 5.9 min). **^{1}H NMR** ($CDCl_3$, 700 MHz) δ: 3.82 (t, J_{HH} = 5.2 Hz, 4 H, 2 × OCH_2), 3.73 (t, J_{HH} = 5.1 Hz, 4 H, 2 × NCH_2), 3.18 (s, 3 H, NCH_3), 3.13 (s, 3 H, NCH_3). **^{13}C NMR** ($CDCl_3$, 176 MHz) δ: 169.3 (s, 1 C, C_{Ar}Cl), 165.1 (s, 1 C, $C_{Ar}N(CH_3)_2$), 164.4 (s, 1 C, C_{Ar}N), 66.6 (s, 2 C, 2 × OCH_2), 43.8 (s, 2 C, 2 × NCH_2), 36.4 (s, 2 C, 2 × CH_3). **HRMS**: *m/z* $[M+H]^+$ $C_9H_{15}ClN_5O^+$ calcd 244.0960, found 244.0950.

S2.8. 4-chloro-N,N-dimethyl-6-(piperidin-1-yl)-1,3,5-triazin-2-amine (**4h**)

Scale of **3h** 2.5 mmol. White solid, mp. 83–85 °C (lit. [7] 68–72 °C). Column chromatography DCM 100%. Yield 59% (0.356 g). Purity 95% (t_R = 7.5 min). **^{1}H NMR** ($CDCl_3$, 700 MHz) δ: 3.72 (t, J_{HH} = 5.7 Hz, 4 H, 2 × NCH_2), 3.11 (s, 3 H, NCH_3), 3.09 (s, 3 H, NCH_3), 1.66–1.61 (m, 2 H, CH_2), 1.58–1.52 (m, 4 H, 2 × CH_2). **^{13}C NMR** ($CDCl_3$, 176 MHz) δ: 169.3 (s, 1 C, C_{Ar}Cl), 165.2 (s, 1 C, $C_{Ar}N(CH_3)_2$), 164.0 (s, 1 C, C_{Ar}N), 44.5 (s, 2 C, 2 × NCH_2), 36.9 (s, 2 C, 2 × CH_3), 25.8 (s, 2 C, 2 × CH_2), 24.7 (s, 1 C, CH_2). **HRMS**: *m/z* $[M+H]^+$ $C_{10}H_{17}ClN_5O^+$ calcd 242.1167, found 242.1197.

S2.9. 4-(azetidin-1-yl)-6-chloro-N,N-dimethyl-1,3,5-triazin-2-amine (**4i**)

Scale of **3i** 1 mmol. Colorless oil. Column chromatography DCM : acetone 9:1 (v:v). Yield 86% (0.183 g). Purity >99% (t_R = 5.6 min). **^{1}H NMR** ($CDCl_3$, 700 MHz) δ: 4.13–4.11 (m, 4 H, 2 × CH_2N), 3.11 (s, 3 H, NCH_3), 3.09 (s, 3 H, NCH_3), 2.33–2.31 (m, 2 H, CH_2). **^{13}C NMR** ($CDCl_3$, 176 MHz) δ: 168.9 (s, 1 C, C_{Ar}Cl), 164.8 (s, 1 C, C_{Ar}N), 164.6 (s, 1 C, C_{Ar}N), 49.4 (s, 2 C, 2 × CH_2), 35.0 (s, 2 C, 2 × CH_3N), 16.2 (s, 1 C, CH_2). **HRMS**: *m/z* $[M+H]^+$ $C_8H_{11}ClN_5^+$ calcd 214.0854, found 214.0867.

S2.10. 4-(4-chloro-6-(piperidin-1-yl)-1,3,5-triazin-2-yl)morpholine (**4j**)

Scale of **3j** 5 mmol. White solid, mp. 138–140 ˚C (lit. [9] 125–126 °C). Column chromatography DCM 100%. Yield 51% (0.733 g). Purity 97% (t_R = 7.2 min). **^{1}H NMR** ($CDCl_3$, 700 MHz) δ: 3.76 (t, J_{HH} = 4.7 Hz, 4 H, 2 × OCH_2), 3.68 (t, J_{HH} = 4.9 Hz, 8 H, 4 × NCH_2), 1.67–1.61 (m, 2 H, CH_2), 1.58–1.53 (m, 4 H, 2 × CH_2). **^{13}C NMR** ($CDCl_3$, 176 MHz) δ: 169.6 (s, 1 C, C_{Ar}Cl), 164.7 (s, 1 C, C_{Ar}N), 164.1 (s, 1 C, C_{Ar}N), 66.7 (s, 2 C, 2 × OCH_2), 44.6 (s, 2 C, 2 × NCH_2), 43.8 (s, 2 C, 2 × NCH_2), 25.7 (s, 2 C, 2 × CH_2), 24.6 (s, 1 C, CH_2). **HRMS**: *m/z* $[M+H]^+$ $C_{12}H_{19}ClN_5O^+$ calcd 284.1273, found 284.1256.

S3. The analytical data for compounds 5a–j.

S3.1. Compound **5a**

Scale of **4a** 0.7 mmol. White solid, mp. 82–84 °C. Yield 67% (0.108 g). Purity 95% (t_R = 1.4 min). **^{1}H NMR** ($CDCl_3$, 700 MHz) δ: 4.96 (bs, 1 H, N*H*), 3.44 (q, J_{HH} = 6.8 Hz, 2 H, C*H*$_2$), 3.08 (s, 12 H, 2 × N(C*H*$_3$)$_2$), 2.86 (t, J_{HH} = 2.7 Hz, 2 H, C*H*$_2$), 1.33 (s, 2 H, N*H*$_2$). **^{13}C NMR** ($CDCl_3$, 176 MHz) δ: 166.5 (s, 1 C, C_{Ar}NH), 165.7 (s, 2 C, 2 × C_{Ar}N(CH_3)$_2$), 43.8 (s, 1 C, *C*H$_2$), 42.2 (s, 1 C, *C*H$_2$), 35.9 (s, 4 C, 4 × *C*H$_3$). **HRMS**: *m/z* [M+H]$^+$ $C_9H_{20}N_7^+$ calcd 226.1775, found 226.1814.

S3.2. Compound **5b**

Scale of **4b** 3.9 mmol. White solid, mp. 48–50 ˚C (lit. [10] 58–60 °C). Yield 82% (0.905 g). Purity 95% (t_R = 3.9 min). **^{1}H NMR** ($CDCl_3$, 700 MHz) δ: 4.89 (t, J_{HH} = 6.4 Hz, 1 H, N*H*), 3.51 (q, J_{HH} = 7.1 Hz, 8 H, 4 × C*H*$_2$), 3.41 (q, J_{HH} = 6.8 Hz, 2 H, C*H*$_2$), 2.85 (t, J_{HH} = 6.0 Hz, 2 H, C*H*$_2$), 1.55 (bs, 2 H, N*H*$_2$), 1.13 (t, J_{HH} = 7.1 Hz, 12 H, 4 × C*H*$_3$). **^{13}C NMR** ($CDCl_3$, 176 MHz) δ: 166.7 (s, 1 C, C_{Ar}NH), 164.6 (s, 2 C, 2 × C_{Ar}N), 43.9 (s, 1 C, *C*H$_2$), 42.3 (s, 1 C, *C*H$_2$), 40.9 (s, 4 C, 4 × *C*H$_2$), 13.5 (s, 4 C, 4 × *C*H$_3$). **HRMS**: *m/z* [M+H]$^+$ $C_{13}H_{28}N_7^+$ calcd 282.2401, found 282.2446.

S3.3. Compound **5c**

Scale of **4c** 0.8 mmol. White solid, mp. 157–160 °C. Yield ≥99% (0.214 g). Purity ≥99% (t_R = 1.2 min). **^{1}H NMR** ($CDCl_3$, 700 MHz) δ: 5.00 (s, 1 H, N*H*), 4.03 (t, J_{HH} = 7.8 Hz, 8 H, 4 × C*H*$_2$N), 3.40 (q, J_{HH} = 6.0 Hz, 2 H, C*H*$_2$NH), 2.82 (t, J_{HH} = 6.0 Hz, 2 H, C*H*$_2$NH$_2$), 2.25 (qn, J_{HH} = 7.7 Hz, 4 H, 2 × C*H*$_2$), 1.49 (s, 2 H, N*H*$_2$). **^{13}C NMR** ($CDCl_3$, 176 MHz) δ: 166.2 (s, 1 C, C_{Ar}N), 166.1 (s, 2 C, 2×C_{Ar}N(CH_2)$_2$), 49.3 (s, 4 C, 4 × *C*H$_2$N), 43.6 (s, 1 C, *C*H$_2$), 42.1 (s, 1 C, *C*H$_2$), 16.3 (s, 2 C, 2 × *C*H$_2$). **HRMS**: *m/z* [M+H]$^+$ $C_{11}H_{20}N_7^+$ calcd 250.1775, found 250.1785.

S3.4. Compound **5d**

Scale of **4d** 1.3 mmol. Yellowish oil. Yield 95% (0.343 g). Purity 95% (t_R = 3.1 min). **^{1}H NMR** ($CDCl_3$, 700 MHz) δ: 5.10 (t, J_{HH} = 6.07 Hz, 1 H, N*H*), 3.51 (bs, 8 H, 4 × C*H*$_2$), 3.45 (q, J_{HH} = 6.0 Hz, 2 H, C*H*$_2$), 2.86 (t, J_{HH} = 6.0 Hz, 2 H, C*H*$_2$), 1.88 (t, J_{HH} = 6.8 Hz, 8 H, 4 × C*H*$_2$), 1.63 (bs, 2 H, N*H*$_2$). **^{13}C NMR** ($CDCl_3$, 176 MHz) δ: 166.3 (s, 1 C, C_{Ar}NH), 163.7 (s, 2 C, 2 × C_{Ar}N), 45.8 (s, 4 C, 4 × *C*H$_2$), 43.8 (s, 1 C, *C*H$_2$), 42.4 (s, 1 C, *C*H$_2$), 25.3 (s, 4 C, 4 × *C*H$_2$). **HRMS**: *m/z* [M+H]$^+$ $C_{13}H_{24}N_7^+$ calcd 278.2088, found 278.2066.

S3.5. Compound **5e**

Scale of **4e** 1.3 mmol. White solid, mp. 83–85 ˚C (lit. [11] 123 °C). Yield 99% (0.405 g). Purity 94% (t_R = 4.0 min). **^{1}H NMR** ($CDCl_3$, 700 MHz) δ: 4.88 (t, J_{HH} = 6.3 Hz, 1 H, N*H*), 3.68 (t, J_{HH} = 6.0 Hz, 8 H, 4 × C*H*$_2$), 3.41 (q, J_{HH} = 5.4 Hz, 2 H, C*H*$_2$), 2.84 (t, J_{HH} = 6.0 Hz, 2 H, C*H*$_2$), 1.64–1.58 (m, 4 H, 2 × C*H*$_2$), 1.55–1.49 (m, 8 H, 4 × C*H*$_2$), 1.22 (bs, 2 H, N*H*$_2$). **^{13}C NMR** ($CDCl_3$, 176 MHz) δ: 166.9 (s, 1 C, C_{Ar}NH), 165.2 (s, 2 C, 2 × C_{Ar}N), 44.1 (s, 4 C, 4 × *C*H$_2$),

43.9 (s, 1 C, CH_2), 42.3 (s, 1 C, CH_2), 25.9 (s, 4 C, 4 × CH_2), 25.1 (s, 2 C, 2 × CH_2). **HRMS**: *m/z* $[M+H]^+$ $C_{15}H_{28}N_7^+$ calcd 306.2401, found 306.2320.

S3.6. Compound **5f**

Scale of **4f** 4 mmol. White solid, mp. 132–134 ˚C (lit. [11] 133 °C). Yield 85% (1.051 g). Purity 95% (t_R = 1.3 min). **^{1}H NMR** ($CDCl_3$, 700 MHz) δ: 5.04 (t, J_{HH} = 6.0 Hz, 1 H, N*H*), 3.71–3.67 (m, 8 H, 4 × OC*H₂*), 3.67–3.66 (m, 8 H, 4 × NC*H₂*), 3.41 (q, J_{HH} = 6.0 Hz, 2 H, C*H₂*), 2.84 (t, J_{HH} = 6.0 Hz, 2 H, C*H₂*), 1.28 (s, 2 H, NH_2). **^{13}C NMR** ($CDCl_3$, 176 MHz) δ: 166.7 (s, 1 C, C_{Ar}NH), 165.4 (s, 2 C, 2 × C_{Ar}N), 66.9 (s, 4 C, 4 × OCH_2), 43.7 (s, 4 C, 4 × NCH_2), 43.6 (s, 1 C, CH_2), 42,0 (s, 1 C, CH_2). **HRMS**: *m/z* $[M+H]^+$ $C_{13}H_{24}N_7O_2^+$ calcd 310.1986, found 310.2028.

S3.7. Compound **5g**

Scale of **4g** 2.4 mmol. Yellowish oil. Yield 76% (0.491 g). Purity 99% (t_R= 1.3 min). **^{1}H NMR** ($CDCl_3$, 700 MHz) δ: 4.99 (t, J_{HH} = 5.7 Hz, 1 H, N*H*), 3.71 (t, J_{HH} = 4.5 Hz, 4 H, 2 × OCH_2), 3.67 (t, J_{HH} = 4.5 Hz, 4 H, NCH_2), 3.42 (q, J_{HH} = 6.0 Hz, 2 H, CH_2), 3.05 (s, 6 H, $N(CH_3)_2$), 2.84 (t, J_{HH} = 6.0 Hz, 2 H, CH_2), 1.27 (bs, 2 H, NH_2). **^{13}C NMR** ($CDCl_3$, 176 MHz) δ: 166.7 (s, 1 C, C_{Ar}N), 165.9 (s, 1 C, C_{Ar}N), 165.4 (s, 1 C, C_{Ar}N), 66.9 (s, 2 C, 2 × OCH_2), 43.8 (s, 2 C, 2 × NCH_2), 43.6 (s, 1 C, CH_2), 42.2 (s, 1 C, CH_2), 36.0 (s, 2 C, 2 × CH_3). **HRMS**: *m/z* $[M+H]^+$ $C_{11}H_{22}N_7O^+$ calcd 268.1880, found 268.1835.

S3.8. Compound **5h**

Scale of **4h** 1.5 mmol. Yellowish oil. Yield 90% (0.352 g). Purity ≥99% (t_R = 2.4 min). **^{1}H NMR** ($CDCl_3$, 700 MHz) δ: 4.90 (t, J_{HH} = 6.2 Hz, 1 H, N*H*), 3.69 (t, J_{HH} = 5.7 Hz, 4 H, 2 × CH_2), 3.42 (q, J_{HH} = 6.0 Hz, 2 H, CH_2), 3.06 (s, 6 H, $N(CH_3)_2$), 2.84 (t, J_{HH} = 6.0 Hz, 2 H, CH_2), 1.64–1.58 (m, 2 H, CH_2), 1.55–1.49 (m, 4 H, 2 × CH_2), 1.21 (bs, 2 H, NH_2). **^{13}C NMR** ($CDCl_3$, 176 MHz) δ: 166.8 (s, 1 C, C_{Ar}NH), 166.0 (s, 1 C, C_{Ar}N), 165.0 (s, 1 C, C_{Ar}N), 44.1 (s, 2 C, 2 × CH_2), 43.8 (s, 1 C, CH_2), 42.3 (s, 1 C, CH_2), 35.9 (s, 2 C, 2 × CH_3), 25.9 (s, 2 C, 2 × CH_2), 25.0 (s, 1 C, CH_2). **HRMS**: *m/z* $[M+H]^+$ $C_{12}H_{24}N_7^+$ calcd 266.2088, found 266.2070.

S3.9. Compound **5i**

Scale of **4i** 2.17 mmol. Colorless oil. Yield 76% (0.391 g). **^{1}H NMR** ($CDCl_3$, 700 MHz) δ: 5.0 (bs, 1 H, N*H*), 4.03 (t, J_{HH} = 7.2 Hz, 4 H, 2 × CH_2N), 3.42 (q, J_{HH} = 5.6 Hz, 2 H, CH_2NH), 3.06 (s, 6 H, 2 × CH_3), 2.84 (t, J_{HH} = 5.9 Hz, 2 H, CH_2NH_2), 2.25 (qn, J_{HH} = 7.2 Hz, 2 H, CH_2), 1.69 (s, 2 H, NH_2). **^{13}C NMR** ($CDCl_3$, 176 MHz) δ: 166.3 (s, 1 C, C_{Ar}NH), 165.5 (s, 2 C, 2 × C_{Ar}N), 49.2 (s, 2 C, 2 × CH_2N), 43.7 (s, 1 C, CH_2), 42.1 (s, 1 C, CH_2), 35.8 (s, 2 C, 2 × CH_3N); 16.3 (s, 1 C, CH_2).

S3.10. Compound **5j**

Scale of **4j** 2.6 mmol. Yellowish oil. Yield 88% (0.698 g). Purity 96% (t_R = 2.6 min). **^{1}H NMR** ($CDCl_3$, 700 MHz) δ: 4.95 (t, J_{HH} = 6.3 Hz, 1 H, N*H*), 3.72 (d, J_{HH} = 4.51 Hz, 4 H, 2 × OCH_2), 3.69-3.66 (m, 8 H, NCH_2), 3.42 (q, J_{HH} = 6.0 Hz, 2 H, CH_2), 2.85 (t, J_{HH} = 6.0 Hz, 2 H, CH_2),

1.63–1.59 (m, 2 H, CH_2), 1.58–1.55 (m, 4 H, 2 × CH_2), 1.38 (bs, 2 H, NH_2). **^{13}C NMR** ($CDCl_3$, 176 MHz) δ: 166.8 (s, 1 C, C_{Ar}N), 165.5 (s, 1 C, C_{Ar}N), 165.0 (s, 1 C, C_{Ar}N), 67.0 (s, 2 C, 2 × OCH_2), 44.1 (s, 2 C, 2 × NCH_2), 43.8 (s, 2 C, 2 × NCH_2), 43.6 (s, 1 C, CH_2), 42.1 (s, 1 C, CH_2), 25.8 (s, 2 C, 2 × CH_2), 25.0 (s, 1 C, CH_2). **HRMS**: *m/z* $[M+H]^+$ $C_{14}H_{26}N_7O^+$ calcd 308.2193, found 308.2193.

S3.11. Compound **5k**

^{1}H NMR ($CDCl_3$, 700 MHz) δ: 4.67 (bs, 1H, NH), 3.38–3.34 (m, 2 H, CH_2), 3.06 (s, 12 H, 4 × CH_3), 2.68–2.63 (m, 2 H, CH_2), 1.57–1.50 (m, 2 H, CH_2), 1.43–1.40 (m, 2 H, CH_2), 1.39–1.32 (m, 4 H, 2 × CH_2), 1.31–1.29 (bs, 2 H, NH_2). **^{13}C NMR** ($CDCl_3$, 176 MHz) δ: 166.3 (s, 1 C, C_{Ar}N), 165.8 (s, 2 C, 2 × C_{Ar}N), 42.3 (s, 1 C, CH_2), 40.6 (s, 1 C, CH_2) 36.4 (s, 1 C, CH_2), 35.9 (s, 4 C, 4 × CH_3), 33.8 (s, 1 C, CH_2), 30.0 (s, 1 C, CH_2), 26.7 (s, 1 C, CH_2). **HRMS**: *m/z* $[M+H]^+$ $C_{13}H_{28}N_7^+$ calcd 282.2401, found 282.2372.

S4. The analytical data for compound 6

^{1}H NMR ($CDCl_3$, 700 MHz) δ: 4.11–4.01 (m, 4 H, 2 × CH_2O), 3.23 (t, J_{HH} = 6.9 Hz, 2 H, CH_2N_3), 1.74–1.65 (m, 2 H, CH_2), 1.63–1.53 (m, 4 H, 2 × CH_2), 1.42–1.34 (m, 4 H, 2 × CH_2), 1.29 (t, J_{HH}= 7.1 Hz, 6 H, 2 × CH_3). **^{31}P NMR** ($CDCl_3$, 283 MHz) δ: 32.82.

The analytical data are consistent with those described in the literature.[12]

S5. The analytical data for compound 7

^{1}H NMR ($CDCl_3$, 400 MHz) δ: 11.43 (bs, 1 H, OH), 4.10–4.06 (m, 2 H, CH_2O), 3.25 (t, J_{HH} = 6.9 Hz, 2 H, CH_2N_3), 1.75–1.71 (m, 2 H, CH_2), 1.65–1.57 (m, 4 H, 2 × CH_2), 1.44–1.36 (m, 4 H, 2 × CH_2), 1.32 (t, J_{HH} = 7.1 Hz, 3 H, CH_3). **^{31}P NMR** ($CDCl_3$, 161.8 MHz) δ: 35.18. **^{13}C NMR** ($CDCl_3$, 176 MHz) δ: 61.8 (d, J_{CP} = 6.8 Hz, 1 C, CH_2O), 51.4 (s, 1 C, CH_2N_3), 30.1 (d, J_{CP} = 16.8 Hz, 1 C, CH_2), 28.7 (s, 1 C, CH_2), 26.3 (s, 1 C, CH_2), 25.8 (d, J_{CP} = 143.5 Hz, 1 C, CH_2P), 22.2 (d, J_{CP} = 5.0 Hz, 1 C, CH_2), 16.4 (d, J_{CP} = 6.4 Hz, CH_3). **HRMS**: *m/z* $[M+Na]^+$ $C_8H_{18}N_3NaO_3P^+$ calcd 258.0978, found 258.0995.

S6. The analytical data for compounds 8a-k

S6.1. Compound **8a**

Scale of **7** 0.85 mmol. Flash chromatography acetone : hexane 10:1 (v:v). Yellow oil. Yield 74% (0.279 g). Purity 95% (t_R = 4.9 min). **^{31}P NMR** ($CDCl_3$, 161.8 MHz) δ: 35.73. **^{1}H NMR** ($CDCl_3$, 400 MHz) δ: 4.98 (bs, 1 H, NH), 4.09–4.00 (m, 1 H, H_α from CH_2O), 3.97–3.88 (m, 1 H, H_β from CH_2O), 3.50–3.45 (m, 2 H, CH_2), 3.35–3.29 (m, 1 H, NH), 3.23 (t, J_{HH} = 6.9 Hz, 2 H, CH_2N_3), 3.11–3.07 (m, 14 H, CH_2 and 2 × N(CH_3)$_2$), 1.72–1.60 (m, 2 H, CH_2), 1.59–1.52 (m, 4 H, 2 × CH_2), 1.36–1.33 (m, 4 H, 2 × CH_2), 1.25 (t, J_{HH} = 7.1 Hz, 3 H, CH_3). **^{13}C NMR** ($CDCl_3$, 176 MHz) δ: 166.7 (s, 1 C, C_{Ar}NH), 165.7 (s, 2 C, 2 × C_{Ar}N), 59.4 (d, J_{CP} = 6.6 Hz, 1 C, CH_2O), 51.5 (s, 1 C, $C_{(6)}H_2N_3$), 42.3 (d, J_{CP} = 6.1 Hz, 1 C, CH_2NH), 42.0 (s, 1 C, CH_2NH), 36.0 (s, 4 C, 2 × N(CH_3)$_2$), 30.3 (d, J_{CP} = 16.4 Hz, 1 C, CH_2), 28,7 (s, 1 C, CH_2), 27.1 (d, J_{CP} = 129.9

Hz, 1 C, P$C_{(1)}H_2$), 26.4 (d, J_{CP} = 1.6 Hz 1 C, CH_2), 22.5 (d, J_{CP} = 4.4 Hz, 1 C, CH_2), 16.5 (d, J_{CP} = 6.6 Hz, CH_3). **HRMS**: *m/z* $[M+H]^+$ $C_{17}H_{36}N_{10}O_2P^+$ calcd 443.2755, found 443.2709.

S6.2. Compound **8b**

Scale of **7** 0.6 mmol. Flash chromatography acetone : hexane 1:1 (v:v). Yellow oil. Yield 57% (0.169 g). **^{31}P NMR** ($CDCl_3$, 161.8 MHz) δ: 35.76 **^{1}H NMR** ($CDCl_3$, 400 MHz) δ: 4.90 (bs, 1 H, N*H*), 4.07–4.02 (m, 1 H, H_α from CH_2O), 3.95–3.90 (m, 1 H, H_β from CH_2O), 3.50 (q, J_{HH} = 6.9 Hz, 8 H, 4 × CH_2), 3.45 (q, J_{HH} = 4.4 Hz, 2 H, CH_2), 3.23 (t, J_{HH} = 6.9 Hz, 2 H, CH_2N_3), 3.11–3.07 (m, 3 H, N*H* and CH_2), 1.71–1.62 (m, 2 H, CH_2), 1.59–1.53 (m, 4 H, 2 × CH_2), 1.39–1.33 (m, 4 H, 2 × CH_2), 1.25 (t, J_{HH} = 7.1 Hz, 3 H, CH_3), 1.12 (t, J_{HH} = 7.0 Hz, 12 H 4 × CH_3). **^{13}C NMR** ($CDCl_3$, 176 MHz) δ: 166.7 (s, 1 C, C_{Ar}NH), 164.6 (s, 2 C, 2 × C_{Ar}N), 59.4 (d, J_{CP} = 6.5 Hz, 1 C, CH_2O), 51.5 (s, 1 C, $C_{(6)}H_2N_3$), 42.3 (d, J_{CP} = 6.1 Hz, 1 C, CH_2NH), 41.7 (s, 1 C, CH_2NH), 41.0 (s, 4 C, 4 × CH_2), 30.3 (d, J_{CP} = 16.7 Hz, 1 C, CH_2), 28,7 (s, 1 C, CH_2), 27.2 (d, J_{CP} = 130.1 Hz, 1 C, P$C_{(1)}H_2$), 26.4 (s, 1 C, CH_2), 22.5 (d, J_{CP} = 4.4 Hz, 1 C, CH_2), 16.5 (d, J_{CP} = 6.6 Hz, CH_3), 13.5 (s, 4 C, 4 × CH_3).

S6.3. Compound **8c**

Scale of **7** 0.54 mmol. Flash chromatography acetone 100% → acetone : methanol 5%. Yellow oil. Yield 41% (0.104 g). Purity ≥99% (t_R = 5.2 min). **^{31}P NMR** ($CDCl_3$, 283 MHz) δ: 35.05. **^{1}H NMR** ($CDCl_3$, 700 MHz) δ: 5.23 (s, 1H, N*H*), 4.04 (t, J_{HH} = 7.5 Hz, 9 H, 4 × CH_2N; Hα from OCH_2), 3.95–3.90 (m, 1 H, H_β from OCH_2), 3.49–3.46 (m, 1H, N*H*), 3.44 (q, J_{HH} = 7.5 Hz, 2 H, CH_2NH); 3.24 (t, J_{HH} = 6.6 Hz, 2 H, CH_2N_3), 3.07–3.03 (m, 2 H, CH_2NH), 2.29–2.25 (m, 4 H, 2 × CH_2), 1.71–1.61 (m, 2 H, CH_2), 1.59–1.53 (m, 4 H, 2 × CH_2), 1.40–1.32 (m, 4 H, 2 × CH_2), 1.26 (t, J_{HH} = 7.0 Hz, 3 H, CH_3CH_2O). **^{13}C NMR** ($CDCl_3$, 176 MHz) δ: 165.9 (s, 1 C, C_{Ar}NH), 165.6 (s, 2 C, 2 × C_{Ar}N), 59.4 (d, J_{CP} = 6.6 Hz, 1 C, CH_2O), 51.5 (s, 1 C, $C_{(6)}H_2N_3$), 45.9 (s, 4 C, 4 × CH_2), 42.3 (d, J_{CP} = 6.3 Hz, 1 C, CH_2NH), 42.1 (s, 1 C, CH_2NH), 30.3 (d, J_{CP} = 16.6 Hz, 1 C, CH_2), 28.7 (s, 1 C, CH_2), 27.1 (d, J_{CP} = 129.8 Hz, 1 C, P$C_{(1)}H_2$), 26.4 (s, 1 C, CH_2), 22.4 (d, J_{CP} = 4.4 Hz, 1 C, CH_2), 16.5 (d, J_{CP} = 6.6 Hz, 1 C, CH_3), 16.3 (s, 2 C, 2 × CH_2). **HRMS**: *m/z* $[M+H]^+$ for $C_{19}H_{36}N_{10}O_2P^+$ calcd 467.2755, found 467.2773.

S6.4. Compound **8d**

Scale of **7** 0.65 mmol. Flash chromatography acetone : methanol 100:1 (v:v). Yellow oil. Yield 52% (0.166 g). **^{31}P NMR** ($CDCl_3$, 161.8 MHz) δ: 35.64. **^{1}H NMR** ($CDCl_3$, 700 MHz) δ: 5.02 (bs, 1 H, N*H*), 4.07–4.02 (m, 1 H, H_α from CH_2O), 3.96–3.90 (m, 1 H, H_β from CH_2O), 3.54–3.46 (m, 11 H, 4 × CH_2, CH_2NH, N*H*), 3.23 (t, J_{HH} = 6.9 Hz, 2 H, CH_2N_3), 3.10–3.06 (m, 2 H, CH_2NH), 1.89–1.86 (m, 8 H, 4 × CH_2), 1.71–1.61 (m, 2 H, CH_2), 1.58–1.54 (m, 4 H, 2 × CH_2), 1.39–1.32 (m, 4 H, 2 × CH_2), 1.26 (t, J_{HH} = 7.1 Hz, 3 H, CH_3). **^{13}C NMR** ($CDCl_3$, 176 MHz) δ: 166.3 (s, 1 C, C_{Ar}NH), 163.4 (s, 2 C, 2 × C_{Ar}N), 59.4 (d, J_{CP} = 6.5 Hz, 1 C, CH_2O), 51.5 (s, 1 C, $C_{(6)}H_2N_3$), 45.9 (s, 4 C, 4 × CH_2), 42.3 (s, 1 C, CH_2NH), 42.3 (d, J_{CP} = 5.0 Hz, 1 C, CH_2NH), 30.3 (d, J_{CP} = 16.6 Hz, 1 C, CH_2), 28.7 (s, 1 C, CH_2), 27.1 (d, J_{CP} = 129.8 Hz, 1 C, P$C_{(1)}H_2$), 26.4 (s, 1 C, CH_2), 25.4 (s, 4 C, 4 × CH_2), 22.4 (d, J_{CP} = 4.4 Hz, 1 C, CH_2), 16.5 (d, J_{CP} = 6.6 Hz, CH_3).

S6.5. Compound **8e**

Scale of **7** 0.66 mmol. Flash chromatography acetone : hexane 1:1 (v:v). Yellow oil. Yield 44% (0.137 g). **^{31}P NMR** ($CDCl_3$, 161.8 MHz) δ: 35.08 **^{1}H NMR** ($CDCl_3$, 400 MHz) δ: 4.90 (bs, 1 H, N*H*), 4.08–4.03 (m, 1 H, H_α from C*H*$_2$O), 3.97–3.91 (m, 1 H, H_β from C*H*$_2$O), 3.69 (bs, 8 H, 4 × C*H*$_2$), 3.50–3.43 (m, 2 H, C*H*$_2$), 3.24 (t, J_{HH} = 6.9 Hz, 2 H, C*H*$_2$N$_3$), 3.13–3.08 (m, 3 H, C*H*$_2$ and N*H*), 1.72–1.61 (m, 6 H, 3 × C*H*$_2$), 1.60–1.52 (m, 12 H, 6 × C*H*$_2$), 1.40–1.33 (m, 4 H, 2 × C*H*$_2$), 1.27 (t, J_{HH} = 7.1 Hz, 3 H, C*H*$_3$). **^{13}C NMR** ($CDCl_3$, 176 MHz) δ: 167.1 (s, 1 C, C_{Ar}NH), 165.1 (s, 2 C, 2 × C_{Ar}N), 59.2 (d, J_{CP} = 6.6 Hz, 1 C, *C*H$_2$O), 51.5 (s, 1 C, $C_{(6)}$H$_2$N$_3$), 44.2 (s, 4 C, 4 × *C*H$_2$), 42.3 (d, J_{CP} = 6.1 Hz, 1 C, *C*H$_2$NH), 41.9 (s, 1 C, *C*H$_2$NH), 30.3 (d, J_{CP} = 16.4 Hz, 1 C, *C*H$_2$), 28.7 (s, 1 C, *C*H$_2$), 27.3 (d, J_{CP} = 129.8 Hz, 1 C, P$C_{(1)}$H$_2$), 26.4 (s, 1 C, *C*H$_2$), 25.9 (s, 4 C, 4 × *C*H$_2$), 25.1 (s, 2 C, 2 × *C*H$_2$), 22.5 (d, J_{CP} = 4.4 Hz, 1 C, *C*H$_2$), 16.6 (d, J_{CP} = 6.6 Hz, *C*H$_3$).

S6.6. Compound **8f**

Scale of **7** 0.96 mmol. Flash chromatography acetone : hexane 1:1 (v:v) → 10:1 (v:v). Yellow oil. Yield 77 % (0.389 g). Purity >95% (t_R = 4.9 min). **^{31}P NMR** ($CDCl_3$, 700 MHz) δ: 35.17. **^{1}H NMR** ($CDCl_3$, 700 MHz) δ: 5.16 (bs, 1H, N*H*), 4.09–4.03 (m, 1 H, H_α from C*H*$_2$O), 3.97–3.91 (m, 1H, H_β from C*H*$_2$O), 3.72 (bs, 8 H, 4 × C*H*$_2$), 3.68 (bs, 8 H, 4 × C*H*$_2$), 3.49–3.44 (m, 2 H, C*H*$_2$NH), 3.24 (t, J_{HH} = 6.9 Hz, 2H, C*H*$_2$N$_3$), 3.11–3.07 (m, 2 H, C*H*$_2$NH), 3.03–2.99 (m, 1 H, N*H*), 1.73–1.62 (m, 2 H, C*H*$_2$), 1.61–1.54 (m, 4 H, 2 × C*H*$_2$), 1.41–1.33 (m, 4 H, 2 × C*H*$_2$), 1,27 (t, J_{HH} = 7.1 Hz, 3 H, C*H*$_3$). **^{13}C NMR** ($CDCl_3$, 176 MHz) δ: 166.5 (s, 1 C, C_{Ar}NH), 165.2 (s, 2 C, 2 × C_{Ar}N), 66.9 (s, 4 C, 4 × *C*H$_2$O), 59.6 (d, J_{CP} = 6.8 Hz, 1 C, *C*H$_2$O), 51.4 (s, 1 C, *C*H$_2$N$_3$), 43.7 (s, 4 C, 4 × *C*H$_2$N), 42.8 (d, J_{CP} = 5.5 Hz, 1 C, *C*H$_2$NH), 41.4 (s, 1 C, *C*H$_2$NH$_2$), 30.3 (d, J_{CP} = 16.6 Hz, 1 C, *C*H$_2$), 28.7 (s, 1 C, *C*H$_2$), 27.2 (d, J_{CP} = 129.8 Hz, 1 C, P*C*H$_2$), 26.4 (s, 1 C, *C*H$_2$), 22.5 (d, J_{CP} = 4.4 Hz, 1 C, *C*H$_2$), 16.6 (d, J_{CP} = 6.5 Hz, 1 C, *C*H$_3$). **HRMS**: *m/z* $[M+H]^+$ for $C_{21}H_{40}N_{10}O_4P^+$ calcd 527.2966, found 527.2963.

S6.7. Compound **8g**

Scale of **7** 0.6 mmol. Flash chromatography acetone : hexane 3:1 (v:v). Yellow oil. Yield 43% (0.124 g). Purity ≥99% (t_R = 5.1 min). **^{31}P NMR** ($CDCl_3$, 283 MHz) δ: 35.09. **^{1}H NMR** ($CDCl_3$, 700 MHz) δ: 5.07 (bs, 1 H, N*H*), 4.09–4.03 (m, 1 H, H_α from C*H*$_2$O), 3.97–3.91 (m, 1H, H_β from C*H*$_2$O), 3.74 (bs, 4 H, 2 × C*H*$_2$), 3.69 (bs, 4 H, 2 × C*H*$_2$), 3.51–3.45 (m, 2 H, C*H*$_2$NH), 3.24 (t, J_{HH} = 6,9 Hz, 2H, C*H*$_2$N$_3$), 3.11–3.08 (m, 9 H, C*H*$_2$N*H*, 2 × CH$_3$), 1.73–1.63 (m, 2 H, C*H*$_2$), 1.60–1.54 (m, 4 H, 2 × C*H*$_2$), 1.40–1.33 (m, 4 H, 2 × C*H*$_2$), 1.27 (t, J_{HH} = 7.1 Hz, 3 H, C*H*$_3$). **^{13}C NMR** ($CDCl_3$, 176 MHz) δ: 166.4 (s, 1 C, C_{Ar}), 165.6 (s, 1 C, C_{Ar}), 165.0 (s, 1 C, C_{Ar}), 67.0 (s, 2 C, 2 × *C*H$_2$O), 59.6 (d, J_{CP} = 6.7 Hz, 1 C, *C*H$_2$O), 51.5 (s, 1 C, *C*H$_2$N$_3$), 43.7 (s, 2 C, 2 × *C*H$_2$N), 42.4 (d, J_{CP} = 5.8 Hz, 1 C, CH$_2$NH), 41.7 (s, 1 C, *C*H$_2$NH$_2$), 36.2 (s, 2 C, 2 × *C*H$_3$), 30.3 (d, J_{CP} = 16.8 Hz, 1 C, *C*H$_2$), 28.7 (s, 1 C, *C*H$_2$), 27.2 (d, J_{CP} = 129.8 Hz, 1 C, P*C*H$_2$), 26.4 (s, 1 C, *C*H$_2$), 22.5 (d, J_{CP} = 4.4 Hz, 1 C, *C*H$_2$), 16.6 (d, J_{CP} = 6.4 Hz, 1 C, *C*H$_3$). HRMS: *m/z* $[M+H]^+$ for $C_{19}H_{36}N_{10}O_3P^+$ calcd 485.2860, found 485.2858.

S6.8. Compound **8h**

Scale of **7** 1.9 mmol. Flash chromatography acetone : hexane 2:1 (v:v). Yellow oil. Yield 48% (0.41 g). Purity >97% (t_R = 5.1 min). ^{31}P NMR ($CDCl_3$, 283 MHz) δ: 35.19. **^{1}H NMR** ($CDCl_3$, 700 MHz) δ: 5.01 (bs, 1 H, N*H*), 4.08–4.02 (m, 1 H, H_α from C*H*$_2$O), 3.96–3.91 (m, 1H, H_β from C*H*$_2$O), 3.69 (bs, 4 H, 2 × C*H*$_2$), 3.51–3.45 (m, 2 H, C*H*$_2$NH), 3.24 (t, J_{HH} = 6,9 Hz, 2H, C*H*$_2$N$_3$), 3.11–3.08 (m, 2 H, C*H*$_2$NH), 3.07 (s, 6 H, 2 × C*H*$_3$), 1.70–1.61 (m, 4 H, 2 × C*H*$_2$), 1.59–1.52 (m, 8 H, 4 × C*H*$_2$), 1.39–1.33 (m, 4 H, 2 × C*H*$_2$), 1.26 (t, J_{HH} = 7.1 Hz, 3 H, C*H*$_3$). **^{13}C NMR** ($CDCl_3$, 176 MHz) δ: 166.7 (s, 1 C, C_{Ar}), 165.8 (s, 1 C, C_{Ar}), 164.8 (s, 1 C, C_{Ar}), 59.5 (d, J_{CP} = 6.7 Hz, 1 C, *C*H$_2$O), 51.5 (s, 1 C, *C*H$_2$N$_3$), 44.2 (s, 2 C, 2 × *C*H$_2$), 42.3 (d, J_{CP} = 6.0 Hz, 1 C, *C*H$_2$NH), 41.9 (s, 1 C, *C*H$_2$NH), 36.1 (s, 2 C, 2 × *C*H$_3$), 30.3 (d, J_{CP} = 16.7 Hz, 1 C, *C*H$_2$), 28.7 (s, 1 C, *C*H$_2$), 27.2 (d, J_{CP} = 129.9 Hz, 1 C, P*C*H$_2$), 26.4 (s, 1 C, *C*H$_2$), 25.9 (s, 2 C, 2 × *C*H$_2$), 25.1 (s, 1 C, *C*H$_2$), 22.4 (d, J_{CP} = 4.4 Hz, 1 C, *C*H$_2$), 16.6 (d, J_{CP} = 6.4 Hz, 1 C, *C*H$_3$). **HRMS**: *m/z* $[M+H]^+$ for $C_{20}H_{40}N_{10}O_2P^+$ calcd 483.3068, found 483.3091.

S6.9. Compound **8i**

Scale of **7** 0.6 mmol. Flash chromatography acetone 100%. Yellow oil. Yield 44% (0.12 g). Purity >97% (t_R = 5.3 min). **^{31}P NMR** ($CDCl_3$, 283 MHz) δ: 35.08. **^{1}H NMR** ($CDCl_3$, 700 MHz) δ: 5.11 (s, 1H, N*H*), 4.07–4.02 (m, 5 H, 2 × C*H*$_2$N; Hα from OC*H*$_2$), 3.96–3.90 (m, 1 H, H_β from OC*H*$_2$), 3.47–3.44 (m, 2H, C*H*$_2$NH), 3.24 (t, J_{HH} = 6.9 Hz, 2 H, C*H*$_2$N$_3$), 3.09–3.06 (m, 8 H, 2×C*H*$_3$, C*H*$_2$N*H*), 2.29–2.25 (m, 2H, C*H*$_2$), 1.71–1.61 (m, 2H, CH$_2$), 1.60–1.53 (m, 4 H, 2 × C*H*$_2$), 1.39–1.33 (m, 4 H, 2 × C*H*$_2$), 1.26 (t, J_{HH} = 7 Hz, 3 H, C*H*$_3$CH$_2$O). **^{13}C NMR** ($CDCl_3$, 176 MHz) δ: 166.2 (s, 1 C, C_{Ar}N), 165.9 (s, 1 C, C_{Ar}N), 165.4 (s, 1 C, C_{Ar}N), 59.4 (d, J_{CP} = 6.6 Hz, 1 C, *C*H$_2$O), 51.5 (s, 1 C, $C_{(6)}H_2N_3$), 49.4 (s, 2 C, 2 × *C*H$_2$), 42.3 (d, J_{CP} = 6.3 Hz, 1 C, *C*H$_2$NH), 41.9 (s, 1 C, *C*H$_2$NH), 36.0 (s, 2 C, 2 × *C*H$_3$), 30.3 (d, J_{CP} = 16.6 Hz, 1 C, *C*H$_2$), 28,7 (s, 1 C, *C*H$_2$), 27.1 (d, J_{CP} = 129.8 Hz, 1 C, P$C_{(1)}H_2$), 26.4 (s, 1 C, *C*H$_2$), 22.4 (d, J_{CP} = 4.4 Hz, 1 C, *C*H$_2$), 16.5 (d, J_{CP} = 6.5 Hz, 1 C, *C*H$_3$), 16.4 (s, 1 C, *C*H$_2$). **HRMS**: *m/z* $[M+H]^+$ for $C_{18}H_{36}N_{10}O_2P^+$ calcd 455.2755, found 455.2767.

S6.10. Compound **8j**

Scale of **7** 1.08 mmol. Flash chromatography acetone : hexane 2:1 (v:v)– 8:3 (v:v). Yellow oil. Yield 46% (0.26 g). Purity ≥99% (t_R = 5.4 min). **^{31}P NMR** ($CDCl_3$, 283 MHz) δ: 35.22. **^{1}H NMR** ($CDCl_3$, 700 MHz) δ: 5.07 (bs, 1 H, N*H*), 4.08–4.03 (m, 1 H, Hα from OC*H*$_2$), 3.97–3.91 (m, 1 H, H_β from OC*H*$_2$), 3.72–3.69 (m, 12 H, 6 × C*H*$_2$), 3.50–3.44 (m, 2 H, C*H*$_2$NH), 3.24 (t, J_{HH} = 6.9 Hz, 2 H, C*H*$_2$N$_3$), 3.11–3.08 (m, 2 H, C*H*$_2$NH), 3.07–3.03 (m, 1 H, N*H*), 1.72–1.62 (m, 4 H, 2 × C*H*$_2$), 1.60–1.52 (m, 8 H, 4 × C*H*$_2$), 1.40–1.33 (m, 4 H, 2 × C*H*$_2$), 1.27 (t, J_{HH} = 7.1 Hz, 3 H, C*H*$_3$). **^{13}C NMR** ($CDCl_3$, 176 MHz) δ: 166.7 (s, 1 C, C_{Ar}), 165.3 (s, 1 C, C_{Ar}), 164.8 (s, 1 C, C_{Ar}), 67.1 (s, 2 C, 2 × *C*H$_2$O), 59.6 (d, J_{CP} = 6.5 Hz, 1 C, *C*H$_2$O), 51.5 (s, 1 C, *C*H$_2$N$_3$), 44.2 (s, 2 C, 2 × *C*H$_2$), 43.7 (s, 2 C, 2 × *C*H$_2$), 42.4 (d, J_{CP} = 6.0 Hz, 1 C, *C*H$_2$NH), 41.6 (s, 1 C, *C*H$_2$NH), 30.3 (d, J_{CP} = 16.6 Hz, 1 C, *C*H$_2$), 28.7 (s, 1 C, *C*H$_2$), 27.2 (d, J_{CP} = 129.9 Hz, 1 C, P*C*H$_2$), 26.4 (s, 1 C, *C*H$_2$), 25.9 (s, 2 C, 2 × *C*H$_2$), 25.0 (s, 1 C, *C*H$_2$), 22.4 (d, J_{CP} = 4.4 Hz, 1 C,

CH_2), 16.6 (d, J_{CP} = 6.5 Hz, 1 C, CH_3). **HRMS**: *m/z* $[M+H]^+$ for $C_{22}H_{42}N_{10}O_3P^+$ calcd 525.3173, found 525.3233.

S6.11. Compound **8k**

Scale of **7** 0.73 mmol. Flash chromatography acetone : hexane 1:1 (v:v). Yellow oil. Yield 60% (0.22 g). **^{31}P NMR** ($CDCl_3$, 283 MHz) δ: 35.63. **^{1}H NMR** ($CDCl_3$, 700 MHz) δ: 4.72 (bs, 1 H, N*H*), 4.08–4.02 (m, 1 H, Hα from OCH_2), 3.95–3.89 (m, 1 H, H_β from OCH_2), 3.35 (dd, J_{HH} = 13.1 Hz, J_{HH} = 6.8 Hz, 2 H, CH_2), 3.26 (t, J_{HH} = 6.9 Hz, 2 H, CH_2N_3), 3.07 (s, 12 H, 4 × CH_3), 2.86–2.82 (m, 2 H, CH_2), 2.41–2.37 (m, 1 H, N*H*), 1.72–1.63 (m, 2 H, CH_2), 1.62–1.55 (m, 4 H, 2 × CH_2), 1.54–1.51 (m, 2 H, CH_2), 1.47–1.43 (m, 2 H, CH_2), 1.42–1.30 (m, 8 H, 4 × CH_2), 1.27 (t, J_{HH} = 7.1 Hz, 3 H, CH_3). **^{13}C NMR** ($CDCl_3$, 176 MHz) δ: 166.3 (s, 1 C, C_{Ar}NH), 165.8 (s, 2 C, 2 × C_{Ar}N), 59.3 (d, J_{CP} = 6.6 Hz, 1 C, CH_2O), 51.4 (s, 1 C, CH_2N_3), 41.0 (s, 1 C, CH_2), 40.5 (s, 1 C, CH_2), 35.9 (s, 4 C, 4 × CH_3), 32.2 (d, J_{CP} = 5.5 Hz, 1 C, CH_2), 30.3 (d, J_{CP} = 16.6 Hz, 1 C, CH_2), 29.9 (s, 1 C, CH_2), 28.7 (s, 1 C, CH_2), 26.9 (d, J_{CP} = 128.9 Hz, 1 C, CH_2P), 26.7 (s, 1 C, CH_2), 26.8 (s, 1 C, CH_2), 26.4 (s, 1 C, CH_2), 22.4 (d, J_{CP} = 4.4 Hz, 1 C, CH_2), 16.5 (d, J_{CP} = 6.5 Hz, 1 C, CH_3).

S7. The analytical data for compounds 1a-k

S7.1. Isothiocyanate-triazine conjugate **1a**

Scale of **8a** 0.63 mmol. Flash chromatography acetone : hexane 3:1 (v:v)→ acetone 100%. Yellow oil. Yield 70% (0.2 g). Purity ≥99% (t_R = 10 min). **^{31}P NMR** ($CDCl_3$, 161.8 MHz) δ: 35.52. **^{1}H NMR** ($CDCl_3$, 400 MHz) δ: 5.00 (bs, 1 H, N*H*), 4.08–4.02 (m, 1 H, H_α from CH_2O), 3.97–3.91 (m, 1 H, H_β from CH_2O), 3.49 (t, J_{HH} = 6.6 Hz, 4 H, CH_2 and CH_2NCS), 3.31–3.28 (m, 1 H, N*H*), 3.12–3.08 (m, 14 H, CH_2 and 2 × N(CH_3)$_2$), 1.72–1.63 (m, 4 H, 2 × CH_2), 1.58–1.54 (m, 2 H, CH_2), 1.42–1.35 (m, 4 H, 2 × CH_2), 1.27 (t, J_{HH} = 7.1 Hz, CH_3). **^{13}C NMR** ($CDCl_3$, 176 MHz) δ: 166.7 (s, 1 C, C_{Ar}NH), 165.7 (s, 2 C, 2 × C_{Ar}N), 129.9 (s, 1 C, N*C*S), 59.4 (d, J_{CP} = 6.7 Hz, 1 C, CH_2O), 45.1 (s, 1 C, $C_{(6)}H_2$NCS), 42.3 (d, J_{CP} = 6.2 Hz, 1 C, CH_2NH), 42.0 (s, 1 C, CH_2NH), 36.1 (s, 4 C, 2 × N(CH_3)$_2$), 29.9 (d, J_{CP} = 16.4 Hz,1 C, CH_2), 29.8 (s, 1 C, CH_2), 27.1 (d, J_{CP} = 129.9 Hz, 1 C, P$C_{(1)}H_2$), 26.3 (s, 1 C, CH_2), 22.4 (d, J_{CP} = 4.5 Hz, 1 C, CH_2), 16.5 (d, J_{CP} = 6.5 Hz, 1 C, CH_3). **HRMS**: *m/z* $[M+H]^+$ calcd for $C_{18}H_{36}N_8O_2PS^+$ 459.2414, found 459.2413. **IR** (ATR) ν: 3260 (N–H), 2927 (C–H_{Arom}), 2860 (C–H_{Arom}), 2178 (NCS), 2090 (NCS), 1521, 1389, 1293 (P=O), 1037 (P–O–C), 950 cm^{-1}.

S7.2. Isothiocyanate-triazine conjugate **1b**

Scale of **8b** 0.34 mmol. Flash chromatography acetone : hexane 1:1 (v:v). Yellow oil. Yield 58% (0.1 g). Purity ≥99% (t_R = 12.5 min). **^{31}P NMR** ($CDCl_3$, 161.8 MHz) δ: 35.63. **^{1}H NMR** ($CDCl_3$, 700 MHz) δ: 4.89 (bs, 1 H, N*H*), 4.08–4.02 (m, 1 H, H_α from CH_2O), 3.96–3.91 (m, 1 H, H_β from CH_2O), 3.51 (q, J_{HH} = 6.9 Hz, 8 H, 4 × CH_2), 3.48 (t, J_{HH} = 6.6 Hz, 2 H, CH_2NCS), 3.46–3.44 (m, 2 H, CH_2), 3.13–3.04 (m, 3 H, N*H* and CH_2), 1.71–1.63 (m, 4 H, 2 × CH_2), 1.61–1.56 (m, 2 H, CH_2), 1.40–1.37 (m, 4 H, 2 × CH_2), 1.26 (t, J_{HH} = 7.1 Hz, 3 H, CH_3), 1.13 (t, J_{HH} = 7.0 Hz, 12 H 4 × CH_3). **^{13}C NMR** ($CDCl_3$, 176 MHz) δ: 166.7 (s, 1 C, C_{Ar}NH), 164.6 (s, 2 C, 2

× C_{Ar}N), 129.9 (s, 1 C, N*C*S), 59.4 (d, J_{CP} = 6.5 Hz, 1 C, *C*H_2O), 45.1 (s, 1 C, *C*H_2NCS), 42.3 (d, J_{CP} = 6,1 Hz, 1 C, *C*H_2NH), 41.7 (s, 1 C, *C*H_2NH), 41.0 (s, 4 C, 4 × *C*H_2), 29.9 (d, J_{CP} = 16.7 Hz, 1 C, *C*H_2), 29.8 (s, 1 C, *C*H_2), 27.1 (d, J_{CP} = 130.1 Hz, 1 C, P$C_{(1)}H_2$), 26.2 (s, 1 C, *C*H_2), 22.4 (d, J_{CP} = 4.4 Hz, 1 C, *C*H_2), 16.5 (d, J_{CP} = 6.6 Hz, 1 C, *C*H_3), 13.6 (s, 4 C, 4 × *C*H_3). **HRMS**: *m/z* $[M+H]^+$ calcd for $C_{22}H_{44}N_8O_2PS^+$ 515.3040, found 515.3044. **IR** (ATR) ν: 3253 (N–H), 2970 (C–H_{Arom}), 2927 (C–H_{Arom}), 2866 (C–H_{Arom}), 2188 (NCS), 2093 (NCS), 1527, 1371, 1300 (P=O), 1036 (P–O–C), 950 cm^{-1}.

S7.3. Isothiocyanate-triazine conjugate **1c**

Scale of **8c** 0.19 mmol. Flash chromatography acetone → acetone : metanol 2% → acetone : metanol 5%. Yellow oil. Yield 63% (0.057 g). Purity ≥99% (t_R = 5.4 min). **^{31}P NMR** ($CDCl_3$, 283 MHz) δ: 34.95. **^{1}H NMR** ($CDCl_3$, 700 MHz) δ: 5.38 (s, 1H, N*H*), 4.06 (t, J_{HH} = 7.5 Hz, 9 H, 4 × CH_2N; Hα from OCH_2), 3.97–3.93 (m, 1 H, H_β from OCH_2), 3.50 (t, J_{HH} = 6.6 Hz, 3 H, CH_2NCS and N*H*), 3.45 (q, J_{HH} = 7.5 Hz, 2 H, CH_2NH); 3.09–3.06 (m, 2 H, CH_2NH), 2.31–2.27 (m, 4 H, 2 × CH_2), 1.72–1.63 (m, 4 H, 2 × CH_2), 1.61–1.55 (m, 2 H, CH_2), 1.42–1.37 (m, 4 H, 2 × CH_2), 1.28 (t, J_{HH} = 7.5 Hz, 3 H, CH_3CH_2O). **^{13}C NMR** ($CDCl_3$, 176 MHz) δ: 165.8 (s, 1 C, C_{Ar}NH), 165.3 (s, 2 C, 2 × C_{Ar}N), 129,9 (s, 1C, N*C*S), 59.5 (d, J_{CP} = 6.7 Hz, 1 C, *C*H_2O), 49.5 (s, 4 C, 4 × *C*H_2), 45.1 (s, 1 C, $C_{(6)}H_2$NCS), 42.4 (d, J_{CP} = 6.3 Hz, 1 C, *C*H_2NH), 42.1 (s, 1 C, *C*H_2NH), 29.9 (d, J_{CP} = 16.4 Hz, 1 C, *C*H_2), 29.8 (s, 1 C, *C*H_2), 27.1 (d, J_{CP} = 130.1 Hz, 1 C, P$C_{(1)}H_2$), 26.3 (s, 1 C, *C*H_2), 22.3 (d, J_{CP} = 4.4 Hz, 1 C, *C*H_2), 16.6 (d, J_{CP} = 6.6 Hz, 1 C, *C*H_3), 16.4 (s, 2 C, *C*H_3). **HRMS**: *m/z* $[M+H]^+$ calcd for $C_{20}H_{36}N_8O_2PS^+$ 483.2414, found 483.2411. **IR** (ATR) ν: 3297 (N–H), 2937 (C–H_{Arom}), 2877 (C–H_{Arom}), 2182 (NCS), 2102 (NCS), 1525, 1357, 1297 (P=O), 1032 (P–O–C), 947 cm^{-1}.

S7.4. Isothiocyanate-triazine conjugate **1d**

Scale of **8d** 0.36 mmol. Flash chromatography acetone → acetone : metanol 4%. Yellow oil. Yield 54% (0.1 g). Purity >96% (t_R = 11.5 min). **^{31}P NMR** ($CDCl_3$, 161,8 MHz) δ: 35.51 **^{1}H NMR** ($CDCl_3$, 700 MHz) δ: 4.97 (bs, 1 H, N*H*), 4.09–4.03 (m, 1 H, H_α from CH_2O), 3.97–3.92 (m, 1 H, H_β from CH_2O), 3.57–3.45 (m, 13 H, 4 × CH_2, CH_2NH, N*H*, CH_2NCS), 3.12–3.08 (m, 2 H, CH_2NH), 1.91–1.87 (m, 8 H, 4 × CH_2), 1.72–1.63 (m, 4 H, 2 × CH_2), 1.61–1.54 (m, 2 H, CH_2), 1.42–1.37 (m, 4 H, 2 × CH_2), 1.27 (t, J_{HH} = 7.1 Hz, 3 H, CH_3). **^{13}C NMR** ($CDCl_3$, 176 MHz) δ: 166.2 (s, 1 C, C_{Ar}NH), 163.4 (s, 2 C, 2 × C_{Ar}N), 129.9 (s, 1 C, N*C*S), 59.4 (d, J_{CP} = 6.5 Hz, 1 C, *C*H_2O), 46.0 (s, 4 C, 4 × *C*H_2), 45.1 (s, 1 C, $C_{(6)}H_2$NCS), 42.3 (s, 1 C, *C*H_2NH), 42.3 (d, J_{CP} = 5.0 Hz, 1 C, *C*H_2NH), 29.9 (d, J_{CP} = 16.6 Hz, 1 C, *C*H_2), 29.8 (s, 1 C, *C*H_2), 27.1 (d, J_{CP} = 129.8 Hz, 1 C, P$C_{(1)}H_2$), 26.3 (s, 1 C, *C*H_2), 25.4 (s, 4 C, 4 × *C*H_2), 22.4 (d, J_{CP} = 4.4 Hz, 1 C, *C*H_2), 16.5 (d, J_{CP} = 6.6 Hz, *C*H_3). **HRMS**: *m/z* $[M+H]^+$ calcd for $C_{22}H_{40}N_8O_2PS^+$ 511.2727, found 511.2725. **IR** (ATR) ν: 3242 (N–H), 2934 (C–H_{Arom}), 2867 (C–H_{Arom}), 2169 (NCS), 2078 (NCS), 1526, 1369, 1232 (P=O), 1035 (P–O–C), 954 cm^{-1}.

S7.5. Isothiocyanate-triazine conjugate **1e**

Scale of **8e** 0.29 mmol. Flash chromatography acetone : hexane 2:1 (v:v). Yellow oil. Yield 72% (0.115 g). Purity ≥99% (t_R = 12.8 min). **^{31}P NMR** ($CDCl_3$, 161.8 MHz) δ: 35.55. **^{1}H NMR**

($CDCl_3$, 400 MHz) δ: 4.96 (bs, 1 H, N*H*), 4.07–4.02 (m, 1 H, H_α from CH_2O), 3.96–3.90 (m, 1 H, H_β from CH_2O), 3.68 (bs, 8 H, 4 × CH_2), 3.49–3.43 (m, 2 H, CH_2), 3.48 (t, J_{HH} = 6.6 Hz, 2 H, CH_2NCS), 3.20–3.17 (m, 1 H, N*H*), 3.11–3.05 (m, 2 H, CH_2), 1.72–1.62 (m, 8 H, 4 × CH_2), 1.58–1.53 (m, 10 H, 5 × CH_2), 1.41–1.35 (m, 4 H, 2 × CH_2), 1.26 (t, J_{HH} = 7.1 Hz, 3 H, CH_3). **^{13}C NMR** ($CDCl_3$, 176 MHz) δ: 166.9 (s, 1 C, $C_{Ar}NH$), 165.1 (s, 2 C, 2 × $C_{Ar}N$), 130.0 (s, 1 C, NCS), 59.5 (d, J_{CP} = 6.5 Hz, 1 C, CH_2O), 45.1 (s, 1 C, CH_2NCS), 44.2 (s, 4 C, 4 × CH_2), 42.3 (d, J_{CP} = 6,1 Hz, 1 C, CH_2NH), 41.9 (s, 1 C, CH_2NH), 29.9 (d, J_{CP} = 16,4 Hz, 1 C, CH_2), 29.8 (s, 1 C, CH_2), 27.2 (d, J_{CP} = 129,9 Hz, 1 C, $PC_{(1)}H_2$), 26.3 (s, 1 C, CH_2), 25.9 (s, 4 C, 4 × CH_2), 25.1 (s, 2 C, 2 × CH_2), 22.4 (d, J_{CP} = 4,4 Hz, 1 C, CH_2), 16.6 (d, J_{CP} = 6,6 Hz, CH_3). **HRMS**: *m/z* $[M+H]^+$ calcd for $C_{24}H_{44}N_8O_2PS^+$ 539.3040, found 539.3039. **IR** (ATR) ν: 3282 (N–H), 2928 (C–H_{Arom}), 2852 (C–H_{Arom}), 2173 (NCS), 2095 (NCS), 1525, 1347, 1239 (P=O), 1027 (P–O–C), 950 cm^{-1}.

S7.6. Isothiocyanate-triazine conjugate **1f**

Scale of **8f** 0.74 mmol. Flash chromatography acetone : hexane 7:1 (v:v). Yellow oil. Yield 46% (0.186 g). Purity ≥99% (t_R = 12.8 min). **^{31}P NMR** ($CDCl_3$, 700 MHz) δ: 34.95. **^{1}H NMR** ($CDCl_3$, 283 MHz) δ: 5.17 (bs, 1 H, N*H*), 4.09–4.02 (m, 1 H, H_α from CH_2O), 3.97–3.91 (m, 1 H, H_β from CH_2O), 3.72 (bs, 8 H, 4 × CH_2), 3.68 (bs, 8 H, 4 × CH_2), 3.49 (t, J_{HH} = 6,6 Hz, 2 H, CH_2NCS), 3.49–3.44 (m, 2 H, CH_2NH), 3.12–3.08 (m, 2 H, CH_2NH), 3.05–3.02 (m, 1 H, N*H*), 1.72–1.63 (m, 4 H, 2 × CH_2), 1.61–1.55 (m, 2 H, CH_2), 1.43–1.37 (m, 4 H, 2 × CH_2), 1.27 (t, J_{HH} = 7.1 Hz, 3 H, CH_3). **^{13}C NMR** ($CDCl_3$, 176 MHz) δ: 166.2 (s, 1 C, $C_{Ar}NH$), 165.1 (s, 2 C, 2 × $C_{Ar}N$), 130.1 (s, 1 C, NCS), 66.9 (s, 4 C, 4 × CH_2O), 59.6 (d, J_{CP} = 6.5 Hz, 1 C, CH_2O), 45.1 (s, 1 C, CH_2NCS), 43.8 (s, 4 C, 4 × CH_2N), 42.5 (d, J_{CP} = 5.4 Hz, 1 C, CH_2NH), 41.3 (s, 1 C, CH_2NH_2), 29.9 (d, J_{CP} = 16.4 Hz, 1 C, CH_2), 29.8 (s, 1 C, CH_2), 27.3 (d, J_{CP} = 129.9 Hz, 1 C, PCH_2), 26.3 (s, 1 C, CH_2), 22.4 (d, J_{CP} = 4.4 Hz, 1 C, CH_2), 16.6 (d, J_{CP} = 6.5 Hz, 1 C, CH_3). HRMS: *m/z* $[M+H]^+$ for $C_{22}H_{40}N_8O_4PS^+$ calcd 543.2625, found 543.2620. **IR** (ATR) ν: 3310 (N–H), 2925 (C–H_{Arom}), 2853 (C–H_{Arom}), 2175 (NCS), 2098 (NCS), 1531, 1358, 1287 (P=O), 1034 (P–O–C), 949 cm^{-1}.

S7.7. Isothiocyanate-triazine conjugate **1g**

Scale of **8g** 0.26 mmol. Flash chromatography acetone : hexane 7:1 (v:v)→acetone 100%→acetone : methanol 9:1 (v:v). Yellow oil. Yield 56% (0.146 g). Purity >97% (t_R = 5.3 min). **^{31}P NMR** ($CDCl_3$, 283 MHz) = δ: 35.09. **^{1}H NMR** ($CDCl_3$, 700 MHz) δ: 5.09 (bs, 1 H, N*H*), 4.09–4.02 (m, 1 H, H_α from CH_2O), 3.97–3.91 (m, 1 H, H_β from CH_2O), 3.74 (bs, 4 H, 2 × CH_2), 3.69 (bs, 4 H, 2 × CH_2), 3.49 (t, J_{HH} = 6.6 Hz, 2 H, CH_2NCS), 3.50–3.48 (m, 2 H, CH_2NH), 3.11–3.08 (m, 9 H, 2×CH_3, CH_2NH), 1.72–1.63 (m, 4 H, 2 × CH_2), 1.61–1.55 (m, 2 H, CH_2), 1.43–1.37 (m, 4 H, 2 × CH_2), 1.27 (t, J_{HH} = 7.1 Hz, 3 H, CH_3). **^{13}C NMR** ($CDCl_3$, 700 MHz) δ: 166.5 (s, 1 C, C_{Ar}), 165.6 (s, 1 C, C_{Ar}), 165.1 (s, 1 C, C_{Ar}), 130.0 (s, 1 C, NCS), 67.0 (s, 2 C, 2 × CH_2O), 59.6 (d, J_{CP} = 6.6 Hz, 1 C, CH_2O), 45.1 (s, 1 C, CH_2NCS), 43.7 (s, 2 C, 2 × CH_2N), 42.4 (d, J_{CP} = 5.8 Hz, 1 C, CH_2NH), 41.7 (s, 1 C, CH_2NH_2), 36.2 (s, 2 C, 2 × CH_3), 29.9 (d, J_{CP} = 16.5 Hz, 1 C, CH_2), 29.8 (s, 1 C, CH_2), 27.3 (d, J_{CP} = 130.1 Hz, 1 C, PCH_2), 26.3 (s, 1

C, CH_2), 22.4 (d, J_{CP} = 4.2 Hz, 1 C, CH_2), 16.6 (d, J_{CP} = 6.4 Hz, 1 C, CH_3). **HRMS**: *m/z* $[M+H]^+$ $C_{20}H_{36}N_8O_3PS^+$ calcd 501.2520, found 501.2521. **IR** (ATR) ν: 3272 (N–H), 2928 (C–H_{Arom}), 2855 (C–H_{Arom}), 2180 (NCS), 2098 (NCS), 1527, 1397, 1288 (P=O), 1023 (P–O–C), 949 cm^{-1}.

S7.8. Isothiocyanate-triazine conjugate **1h**

Scale of **8h** 0.85 mmol. Flash chromatography acetone : hexane 2:1 (v:v)→ 1:1 (v:v). Yellow oil. Yield 48% (0.203 g). Purity > 97% (t_R = 5.6 min). ^{31}**P NMR** ($CDCl_3$, 283 MHz) δ: 35.04. ^{1}**H NMR** ($CDCl_3$, 700 MHz) δ: 4.98 (bs, 1 H, N*H*), 4.08–4.03 (m, 1 H, H_α from CH_2O), 3.97–3.91 (m, 1H, H_β from CH_2O), 3.70 (bs, 4 H, 2 × CH_2), 3.51–3.45 (m, 4 H, CH_2NH, CH_2NCS), 3.12–3.09 (m, 2 H, CH_2NH), 3.07 (s, 6 H, 2 × CH_3), 1.71–1.62 (m, 6 H, 3 × CH_2), 1.59–1.52 (m, 6 H, 3 × CH_2), 1.41–1.36 (m, 4 H, 2 × CH_2), 1.27 (t, *J*= 7.1 Hz, 3 H, CH_3). ^{13}**C NMR** ($CDCl_3$, 176 MHz) δ: 166.6 (s, 1 C, C_{Ar}), 165.6 (s, 1 C, C_{Ar}), 164.7 (s, 1 C, C_{Ar}), 129.9 (s, 1 C, N*C*S), 59.5 (d, J_{CP} = 6.7 Hz, 1 C, *C*H_2O), 45.1 (s, 1 C, *C*H_2NCS), 44.2 (s, 2 C, 2 × *C*H_2), 42.3 (d, J_{CP} = 6.0 Hz, 1 C, *C*H_2NH), 41.9 (s, 1 C, *C*H_2NH), 36.1 (s, 2 C, 2 × *C*H_3), 29.9 (d, J_{CP} = 16.7 Hz, 1 C, *C*H_2), 29.8 (s, 1 C, *C*H_2), 27.2 (d, J_{CP} = 130.0 Hz, 1 C, P*C*H_2), 26.3 (s, 1 C, *C*H_2), 25.9 (s, 2 C, 2 × *C*H_2), 25.1 (s, 1 C, *C*H_2), 22.4 (d, J_{CP} = 4.4 Hz, 1 C, *C*H_2), 16.5 (d, J_{CP} = 6.5 Hz, 1 C, *C*H_3). **HRMS**: *m/z* $[M+H]^+$ for $C_{21}H_{40}N_8O_2PS^+$ calcd 499.2727, found 499.2737. **IR** (ATR) ν: 3282 (N–H), 2929 (C–H_{Arom}), 2853 (C–H_{Arom}), 2176 (NCS), 2096 (NCS), 1524, 1396, 1295 (P=O), 1033 (P–O–C), 950 cm^{-1}.

S7.9. Isothiocyanate-triazine conjugate **1i**

Scale of **8i** 0.26 mmol. Flash chromatography acetone 100% → acetone : methanol 2%. Yellow oil. Yield 67% (0.082 g). Purity ≥ 99% (t_R = 5.5 min). ^{31}**P NMR** ($CDCl_3$, 283 MHz) δ: 35.04. ^{1}**H NMR** ($CDCl_3$, 700 MHz) δ: 5.06 (s, 1 H, N*H*), 4.08–4.01 (m, 5 H, 2 × CH_2N; Hα from OCH_2), 3.97–3.91 (m, 1 H, H_β from OCH_2), 3.50 (t, J_{HH} = 6.6 Hz, 2 H, CH_2NCS), 3.48–3.46 (m, 2H, CH_2NH), 3.11–3.07 (m, 8 H, 2×CH_3, CH_2N*H*), 2.31–2.26 (m, 2 H, CH_2), 1.73–1.61 (m, 4 H, 2 × CH_2), 1.60–1.55 (m, 2 H, CH_2), 1.42–1.35 (m, 4 H, 2 × CH_2), 1.27 (t, J_{HH} = 7.1 Hz, 3 H, CH_3CH_2O). ^{13}**C NMR** ($CDCl_3$, 176 MHz) δ: 166.2 (s, 1 C, C_{Ar}N), 165.9 (s, 1 C, C_{Ar}N), 165.4 (s, 1 C, C_{Ar}N), 129.9 (s, 1 C, N*C*S), 59.5 (d, J_{CP} = 6.6 Hz, 1 C, *C*H_2O), 49.4 (s, 2 C, 2 × *C*H_2), 45.1 (s, 1 C, *C*H_2NCS), 42.3 (d, J_{CP} = 6.3 Hz, 1 C, *C*H_2NH), 41.9 (s, 1 C, *C*H_2NH), 36.1 (s, 2 C, 2 × *C*H_3), 29.9 (d, J_{CP} = 16.5 Hz, 1 C, *C*H_2), 29.8 (s, 1 C, *C*H_2), 27.1 (d, J_{CP} = 129.8 Hz, 1 C, P$C_{(1)}H_2$), 26.2 (s, 1 C, *C*H_2), 22.4 (d, J_{CP} = 4,4 Hz, 1 C, *C*H_2), 16.5 (d, J_{CP} = 6,5 Hz, 1 C, *C*H_3), 16.4 (s, 1 C, *C*H_2). **HRMS**: *m/z* $[M+H]^+$ for $C_{19}H_{36}N_8O_2PS^+$ calcd 471.2414, found 471.2405. **IR** (ATR) ν: 3283 (N–H), 2930 (C–H_{Arom}), 2876 (C–H_{Arom}), 2171 (NCS), 2102 (NCS), 1517, 1396, 1300 (P=O), 1032 (P–O–C), 948 cm^{-1}.

S7.10. Isothiocyanate-triazine conjugate **1j**

Scale of **8j** 0.85 mmol. Flash chromatography hexane : acetone 2:1(v:v) to 1:1 (v:v). Yellow oil. Yield 66% (0.165 g). Purity > 95% (t_R = 5.8 min). ^{31}**P NMR** ($CDCl_3$, 283 MHz) δ: 35.13. ^{1}**H NMR** ($CDCl_3$, 700 MHz) δ: 5.02 (bs, 1 H, N*H*), 4.09–4.04 (m, 1 H, Hα from OCH_2), 3.97–3.92 (m, 1 H, H_β from OCH_2), 3.73–3.69 (m, 12 H, 6 × CH_2), 3.50 (t, J_{HH} = 6.6 Hz, 2 H, CH_2NCS),

3.47–3.45 (m, 2 H, CH_2NH), 3.13–3.09 (m, 2 H, CH_2NH), 3.06 (bs, 1 H, N*H*), 1.72–1.62 (m, 6 H, 3 × CH_2), 1.61–1.54 (m, 2 H, CH_2), 1.56–1.54 (m, 4 H, 2 × CH_2), 1.42–1.38 (m, 4 H, 2 × CH_2), 1.28 (t, J_{HH} = 7.1 Hz, 3 H, CH_3). **^{13}C NMR** ($CDCl_3$, 176 MHz) δ: 166.7 (s, 1 C, C_{Ar}), 165.3 (s, 1 C, C_{Ar}), 164.8 (s, 1 C, C_{Ar}), 130.1 (s, 1 C, N*C*S), 67.0 (s, 2 C, 2 × CH_2O), 59.6 (d, J_{CP} = 6.6 Hz, 1 C, CH_2O), 45.1 (s, 1 C, CH_2NCS), 44.2 (s, 2 C, 2 × CH_2), 43.7 (s, 2 C, 2 × CH_2), 42.3 (d, J_{CP} = 5.7 Hz, 1 C, CH_2NH), 41.6 (s, 1 C, CH_2NH), 29.9 (d, J_{CP} = 16.5 Hz, 1 C, CH_2), 29.8 (s, 1 C, CH_2), 27.1 (d, J_{CP} = 130.0 Hz, 1 C, PCH_2), 26.2 (s, 1 C, CH_2), 25.9 (s, 2 C, 2 × CH_2), 25.0 (s, 1 C, CH_2), 22.4 (d, J_{CP} = 4.4 Hz, 1 C, CH_2), 16.6 (d, J_{CP} = 6.5 Hz, 1 C, CH_3). **HRMS**: *m/z* $[M+H]^+$ for $C_{23}H_{42}N_8O_3PS^+$ calcd 541.2843, found 541.2873. **IR** (ATR) ν: 3289 (N–H), 2929 (C–H_{Arom}), 2852 (C–H_{Arom}), 2175 (NCS), 2095 (NCS), 1528, 1359, 1294 (P=O), 1023 (P–O–C), 949 cm^{-1}.

S7.11. Isothiocyanate-triazine conjugate **1k**

Scale of **8k** 0.24 mmol. Flash chromatography hexane : acetone 2:1 (v:v). Yellow oil. Yield 80% (0.1 g). Purity ≥ 99% (t_R = 11.8 min). **^{31}P NMR** ($CDCl_3$, 283 MHz) δ: 35.49. **^{1}H NMR** ($CDCl_3$, 700 MHz) δ: 4.68 (bs, 1 H, N*H*), 4.10–4.03 (m, 1 H, Hα from OCH_2), 3.98–3.92 (m, 1 H, $H_β$ from OCH_2), 3.50 (t, J_{HH} = 6.6 Hz, 2 H, CH_2NCS), 3.39 (q, J_{HH} = 6.6 Hz, 2 H, CH_2NH), 3.08 (s, 12 H, 4 × CH_3), 2.89–2.84 (m, 2 H, CH_2), 2.34–2.31 (m, 1 H, N*H*), 1.73–1.63 (m, 6 H, 3 × CH_2), 1.59–1.54 (m, 2 H, CH_2), 1.50–1.45 (m, 2 H, CH_2), 1.44–1.40 (m, 4 H, 2 × CH_2), 1.39–1.33 (m, 4 H, 2 × CH_2), 1.30 (t, J_{HH} = 7.1 Hz, 3 H, CH_3). **^{13}C NMR** ($CDCl_3$, 176 MHz) δ: 166.3 (s, 1 C, C_{Ar}NH), 165.8 (s, 2 C, 2 × C_{Ar}N), 130.0 (s, 1 C, N*C*S), 59.4 (d, J_{CP} = 6.6 Hz, 1 C, CH_2O), 45.1 (s, 1 C, CH_2NCS), 41.0 (s, 1 C, CH_2), 40.6 (s, 1 C, CH_2), 36.0 (s, 4 C, 4 × CH_3), 32.2 (d, J_{CP} = 5.4 Hz, 1 C, CH_2), 30.0 (d, J_{CP} = 4.6 Hz, 1 C, CH_2), 29.9 (d, J_{CP} = 16.4 Hz, 1 C, CH_2), 29.8 (s, 1 C, CH_2), 26.9 (d, J_{CP} = 130.3 Hz, 1 C, CH_2P), 26.7 (s, 1 C, CH_2), 26.6 (s, 1 C, CH_2), 26.3 (s, 1 C, CH_2), 22.3 (d, J_{CP} = 4.4 Hz, 1 C, CH_2), 16.5 (d, J_{CP} = 6.6 Hz, 1 C, CH_3). **HRMS**: *m/z* $[M+H]^+$ for $C_{22}H_{44}N_8O_2PS^+$ calcd 515.3140, found. 515.3122. **IR** (ATR) ν: 3250 (N–H), 2926 (C–H_{Arom}), 2857 (C–H_{Arom}), 2180 (NCS), 2094 (NCS), 1521, 1390, 1190 (P=O), 1035 (P–O–C), 946 cm^{-1}.

S8. References

1. Patel, D.; Kumari, P.; Patel, N. Synthesis and Biological Evaluation of Some Thiazolidinones as Antimicrobial Agents. *Eur. J. Med. Chem.*, **2012**, *48*, 354–362.
2. Sole, R.; Agostinis, L.; Conca, S.; Gatto, V.; Bardella, N.; Morandini, A.; Buranello, C.; Beghetto, V. Synthesis of Amidation Agents and Their Reactivity in Condensation Reactions. *Synthesis*, **2021**, *53*, 1672–1682.
3. Bakharev, V.V.; Gidaspov, A.A.; Parfenov, V.E.; Ul'yankina, I.V.; Zavodskaya, A.V.; Selezneva, E.V.; Suponitskii, K. Yu.; Sheremetev, A.B. Synthesis of 4-amino-6-chloro-1,3,5-triazin-2(1H)-ones. *Russ. Chem. Bull.*, **2012**, *61*, 99–112.
4. Kumari, P.; Kaur, S.; Kaur, J.; Bhatti, R.; Singh, P. Modification of the Lead Molecule: Tryptophan and Piperidine Appended Triazines Reversing Inflammation and Hyperalgesia in Rats. *Bioorg. Med. Chem.*, **2020**, *28*, 115246.
5. Khalil, A.F.; El-Moselhy, T.F.; El-Bastawissy, E.A.; Abdelhady, R.; Younis, N.S.; El-Hamamsy, M.H. Discovery of Novel Enasidenib Analogues Targeting Inhibition of Mutant Isocitrate Dehydrogenase 2 as Antileukaemic Agents. *J. Enzyme Inhib. Med. Chem.*, **2023**, *38*, 2157411.

6. d'Atri, G.; Gomarasca, A.; Resnati, G.; Tronconi, G.; Scolastico, C.; Sirtori, C.R. Novel Pyrimidine and 1,3,5-Triazine Hypolipidemic Agents. *J. Med. Chem.*, **1984**, *27*, 1621–1629.
7. Matsuno, T.; Kato, M.; Tsuchida, Y.; Takahashi, M.; Yaguchi, S.; Terada, S. Synthesis and Aromatase-Inhibitory Activity of Imidazoly-1,3,5-Triazine Derivatives. *Chem. Pharm. Bull.*, **1997**, *45*, 291–296.
8. Lemos, B.C.; Westphal, R.; Filho, E.V.; Fiorot, R.G.; Carneiro, J.W.M.; Gomes, A.C.C, Guimarães, C.J.; de Oliveira, F.C.E.; Costa, P.M.S.; Pessoa, C.; Greco, S. Synthetic Enamine Naphthoquinone Derived from Lawsone as Cytotoxic Agents Assessed by In vitro and In silico Evaluations. *Bioorg. Med. Chem. Lett.*, **2021**, *53*, 128419.
9. Sharma, A.; Jad, Y.; Siddiqui, M.R.H.; de la Torre, B.G.; Albericio, F.; El-Faham, A. Synthesis, Characterization, and Tautomerism of 1,3-Dimethyl Pyrimidine-2,4,6-trione S-triazinyl Hydrazine/Hydrazone Derivatives. *J. Chem.*, **2017**, 5702962.
10. Dovlatyan, V.V.; Éliazyan, K.A.; Pivazyan, V.A.; Akopyan, A.M. Reactions of Derivatives of Amino- and Mercapto-sym-triazines with Ethyleneimine and Ethylenediamine. *Chem. Heterocycl. Compd.*, **1993**, *29*, 704–707.
11. Cloete, T.T.; de Kock, C.; Smith, P.J.; N'Da, D.D. Synthesis, In vitro Antiplasmodial Activity and Cytotoxicity of a Series of Artemisinin–triazine Hybrids and Hybrid-dimers. *Eur. J. Med. Chem.*, **2014**, *76*, 470–481.
12. Janczewski, Ł.; Gajda, A.; Braszczyńska, J.; Gajda, T. Microwave-assisted Synthesis of Dialkyl ω-azidoalkylphosphonates. *Synth. Commun.* **2016**, *46*, 1625–1633.

S9. Analytical spectra of 7

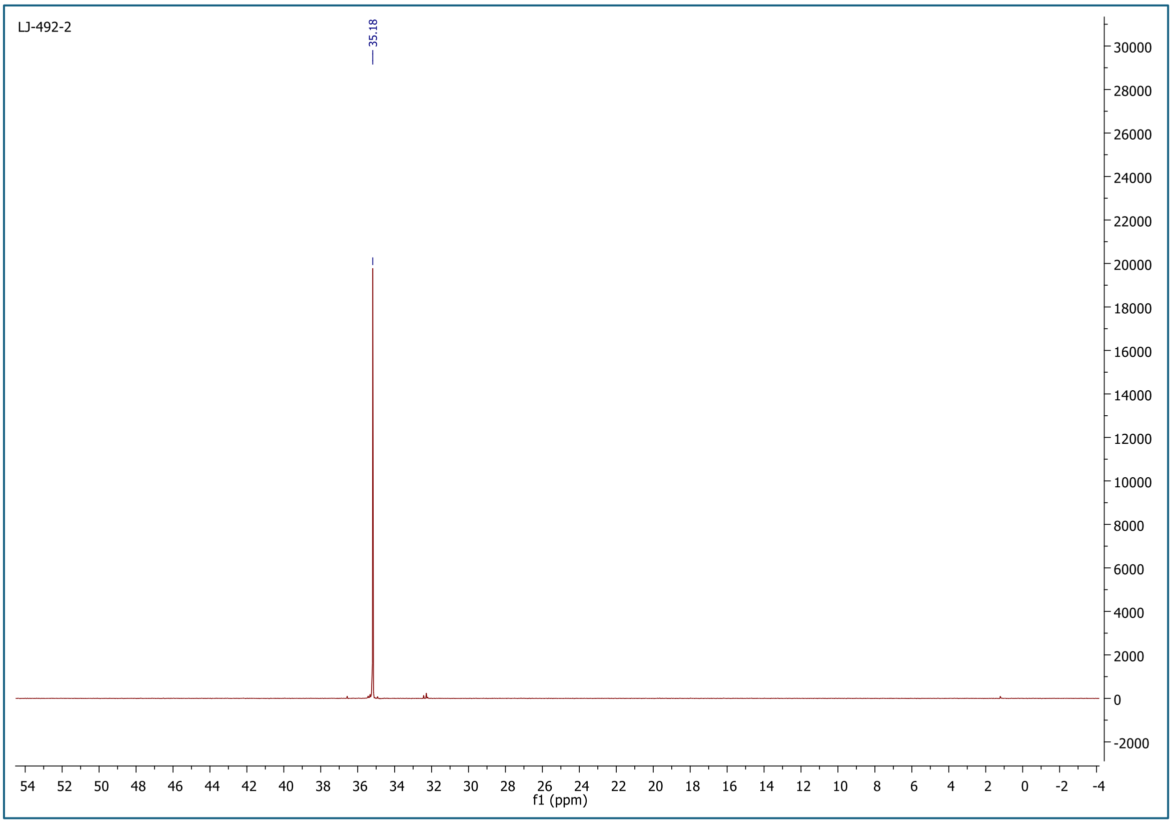

Fig. S1. ^{31}P NMR 7 ($CDCl_3$, 700 MHz).

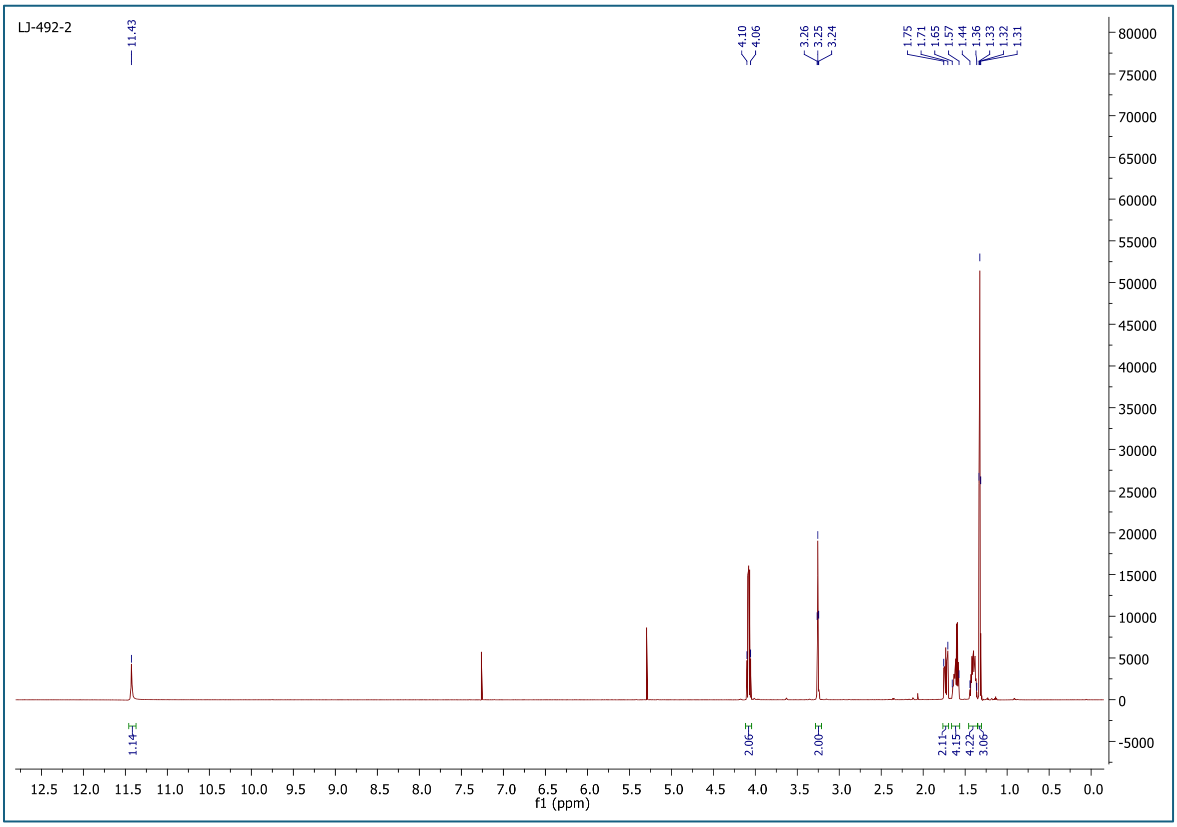

Fig. S2. ^{1}H NMR 7 ($CDCl_3$, 700 MHz).

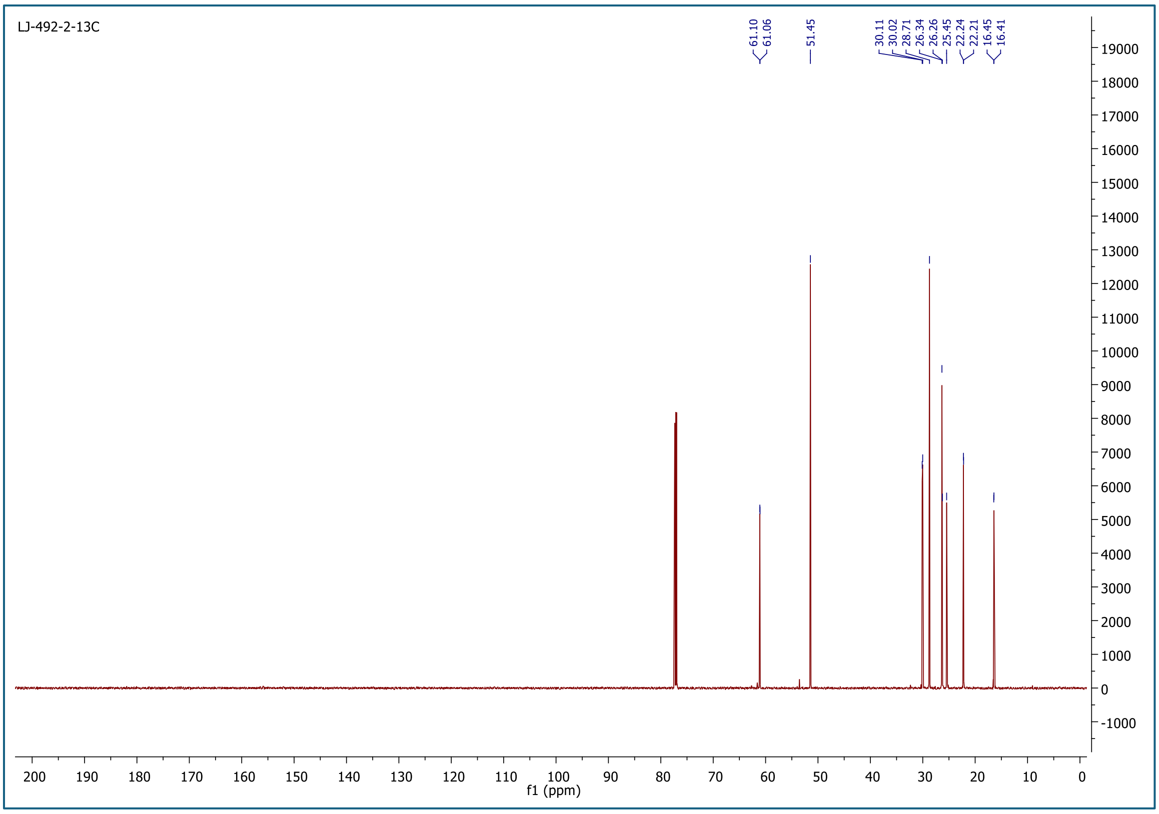

Fig. S3. ^{13}C NMR 7 ($CDCl_3$, 176 MHz).

S10. Analytical spectra of 3a

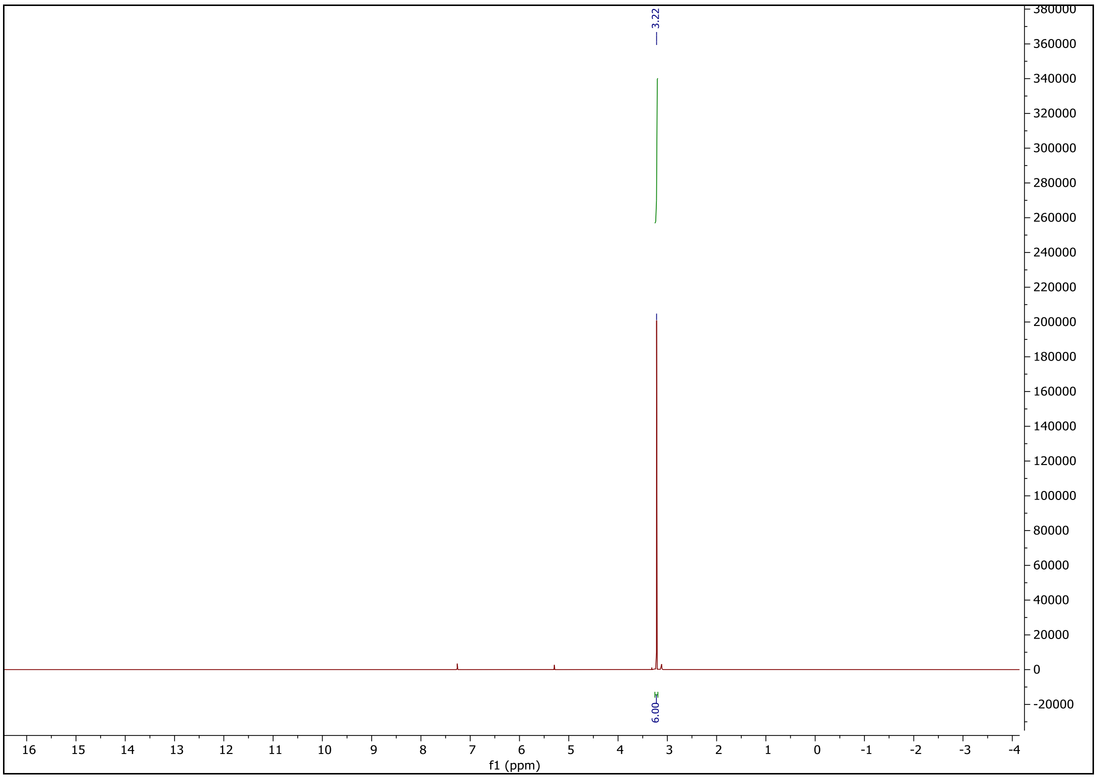

Fig. S4. ^{1}H NMR 3a ($CDCl_3$, 400 MHz).

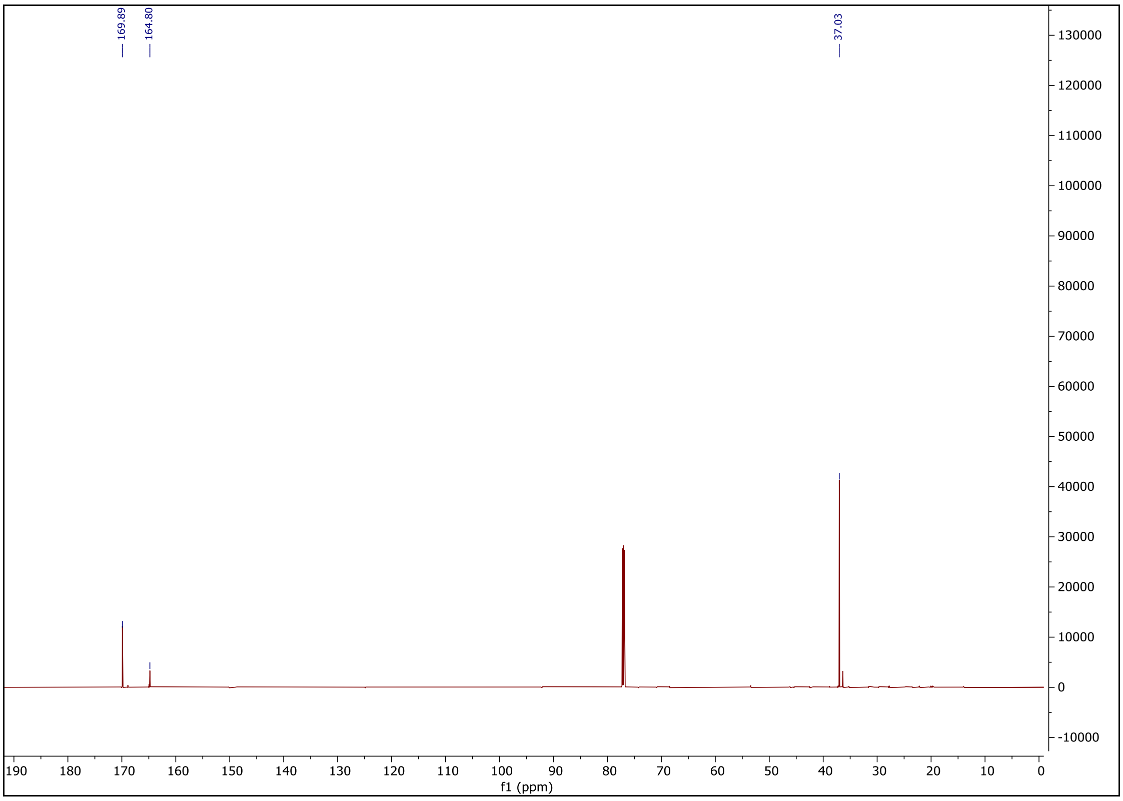

Fig. S5. ^{13}C NMR 3a ($CDCl_3$, 176 MHz).

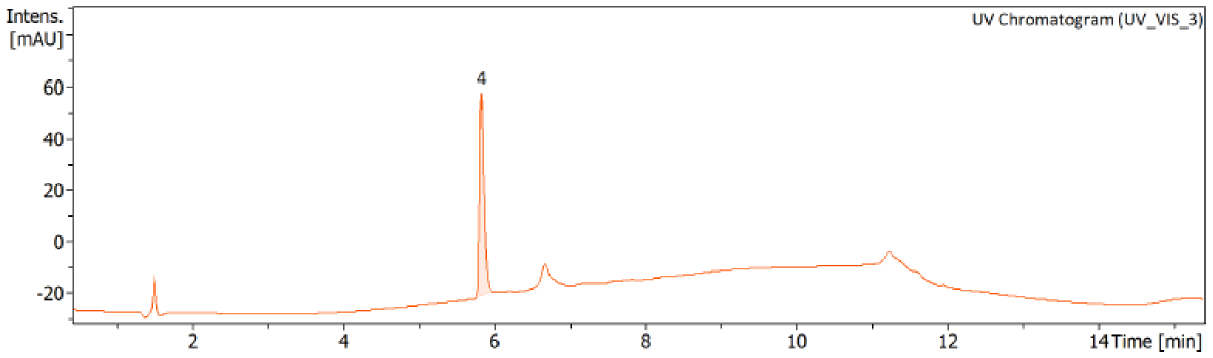

Fig. S6. UV-Vis Chromatogram o3a

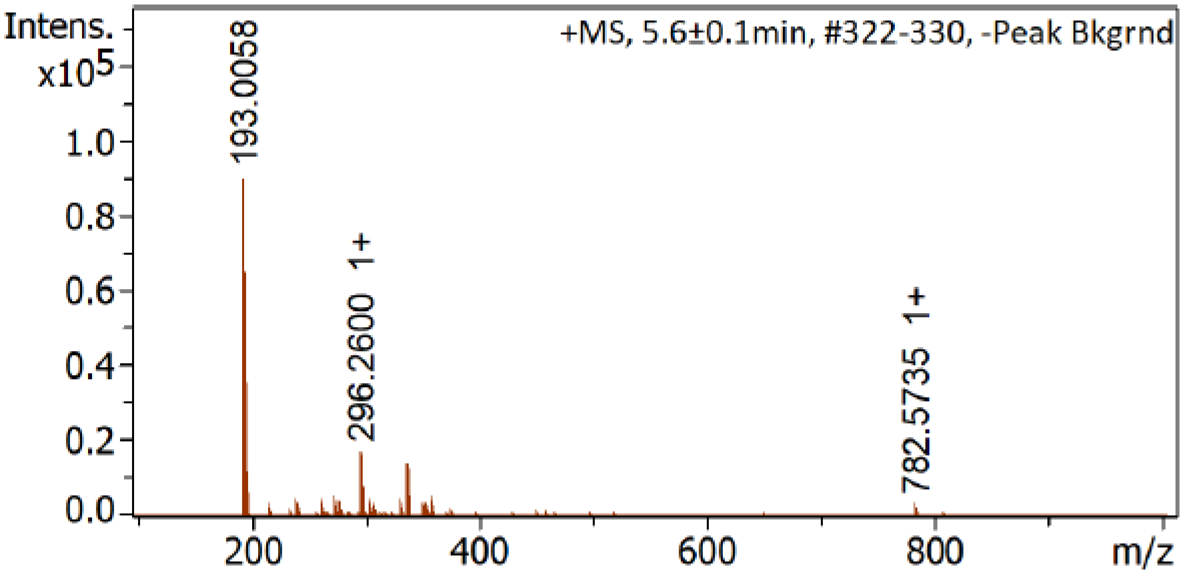

Fig. S7. Mass spectrum 3a

S11. Analytical spectra of 3b

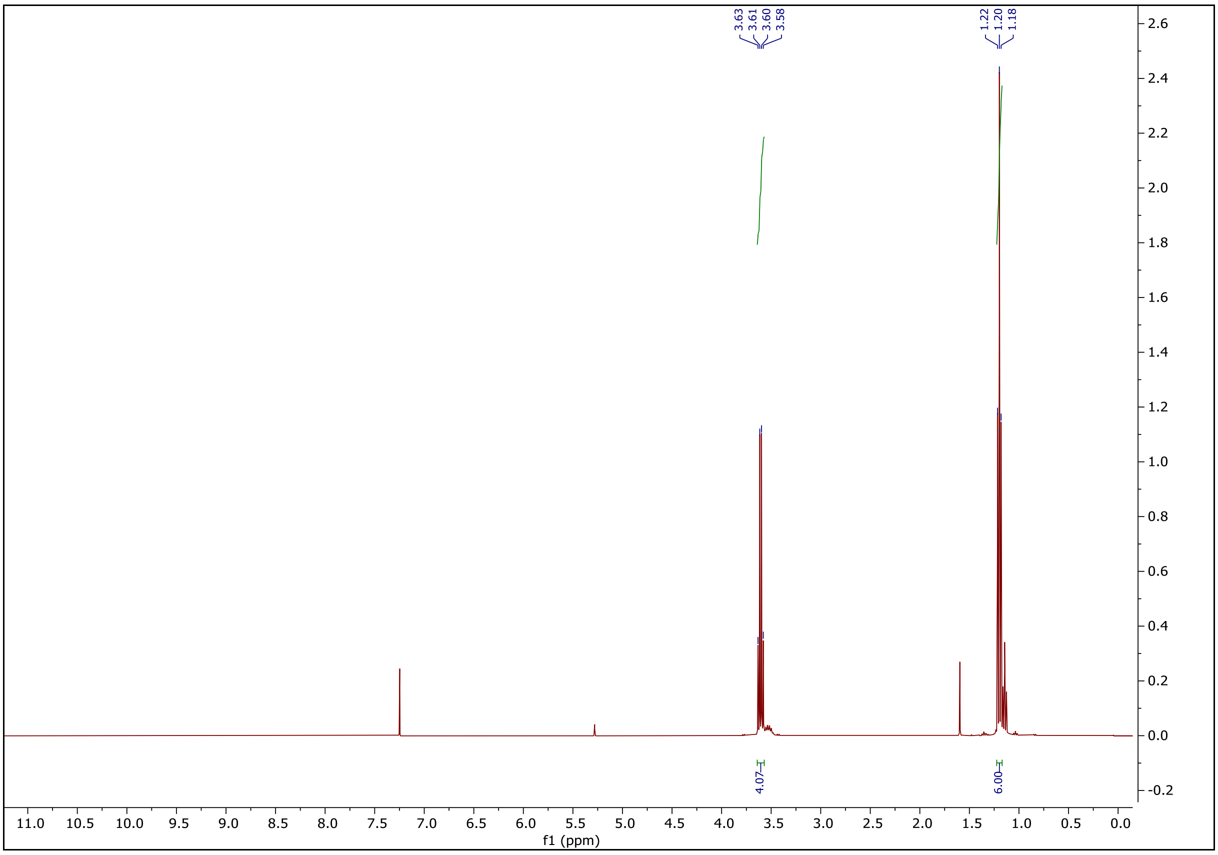

Fig. S8. ^{1}H NMR 3b ($CDCl_3$, 700 MHz)

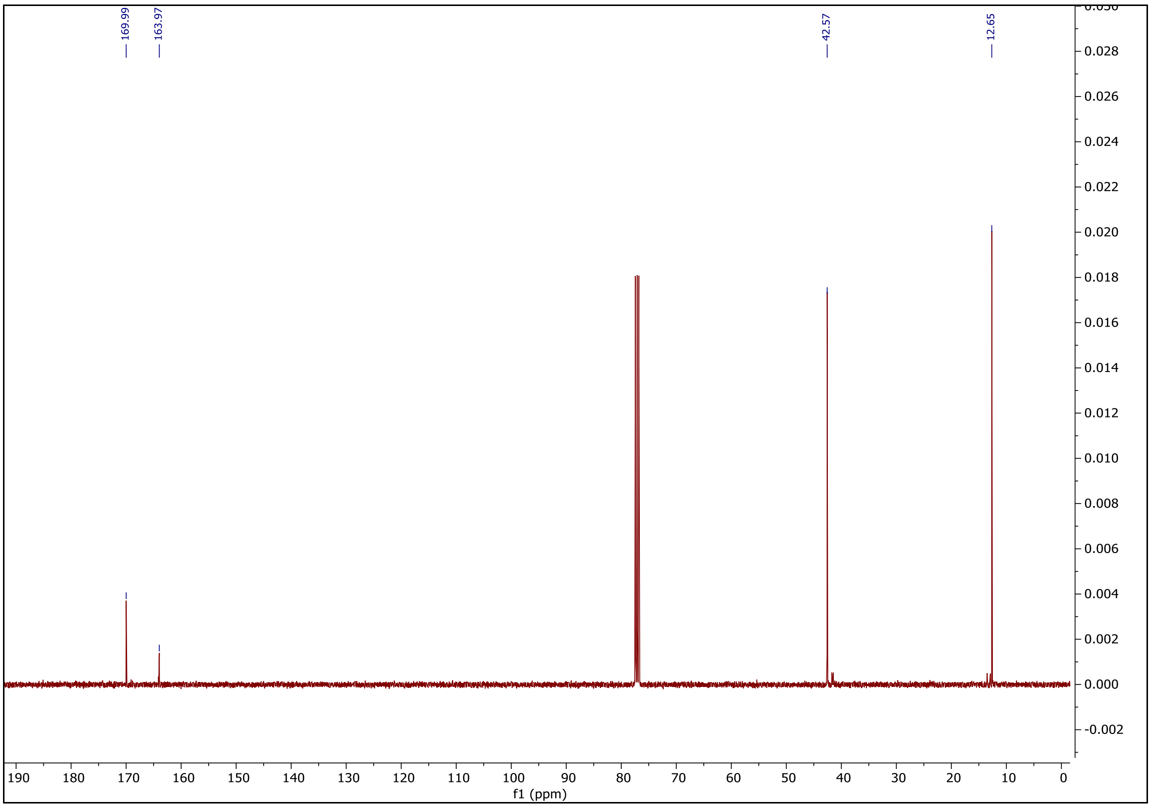

Fig. S9. ^{13}C NMR 3b ($CDCl_3$, 176 MHz)

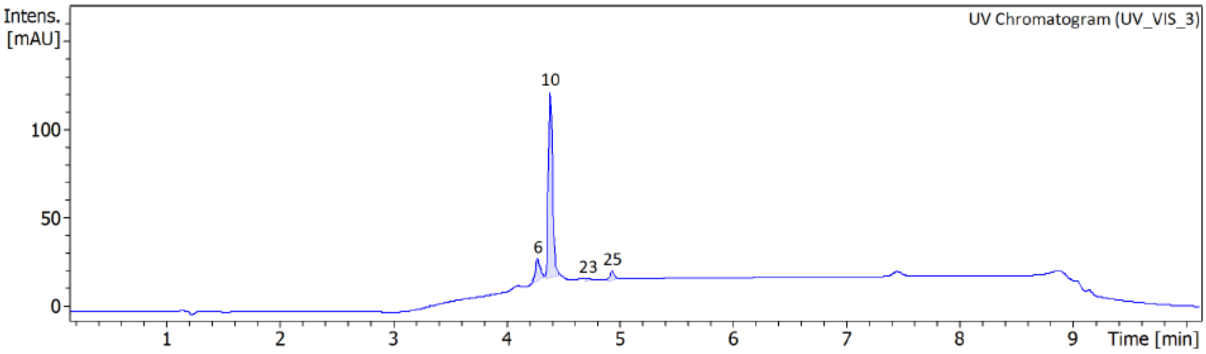

Fig. S10. UV-Vis Chromatogram 3b

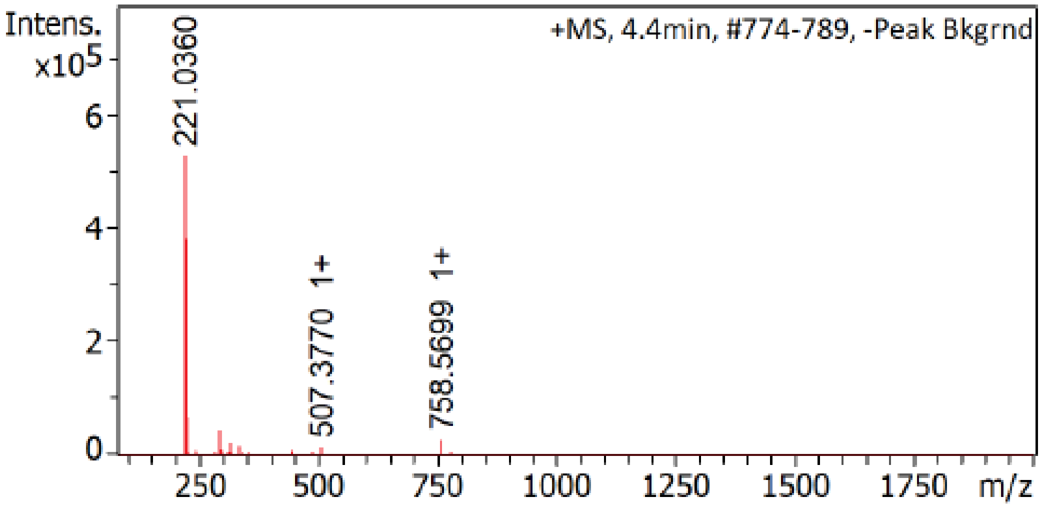

Fig. S11. Mass spectrum 3b

S12. Analytical spectra of 3c

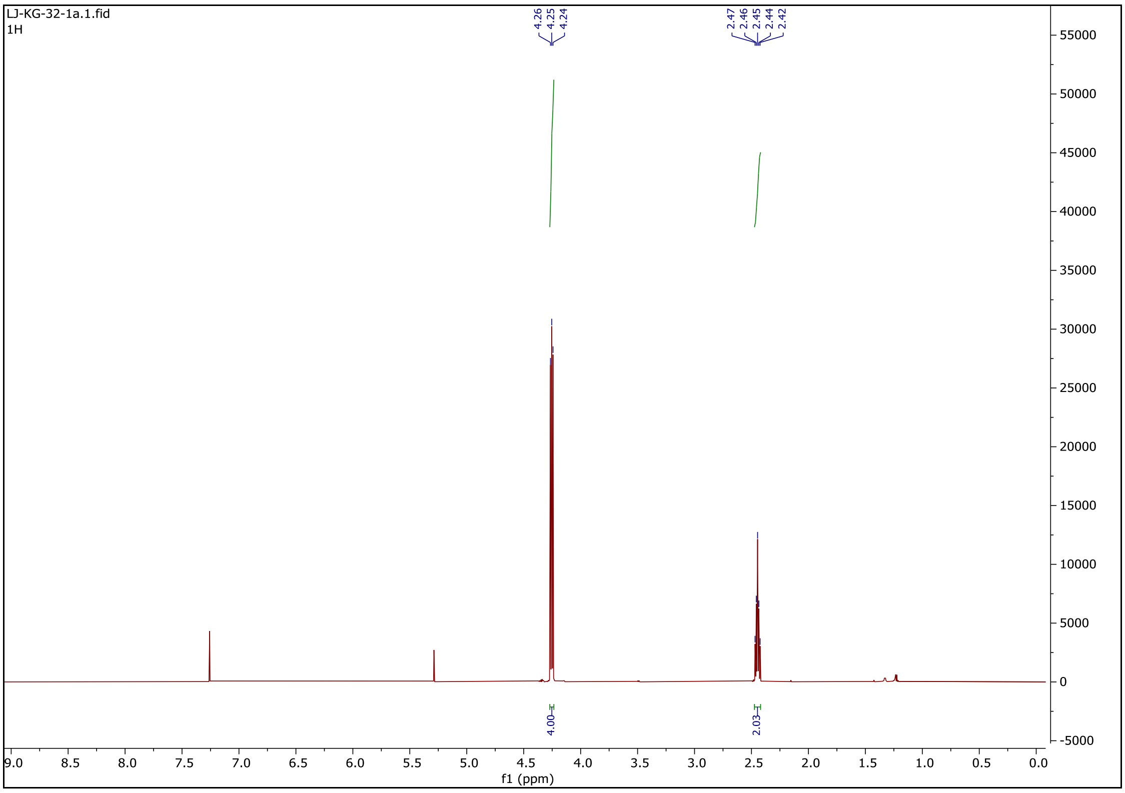

Fig. S12. ^{1}H NMR 3c ($CDCl_3$, 700 MHz)

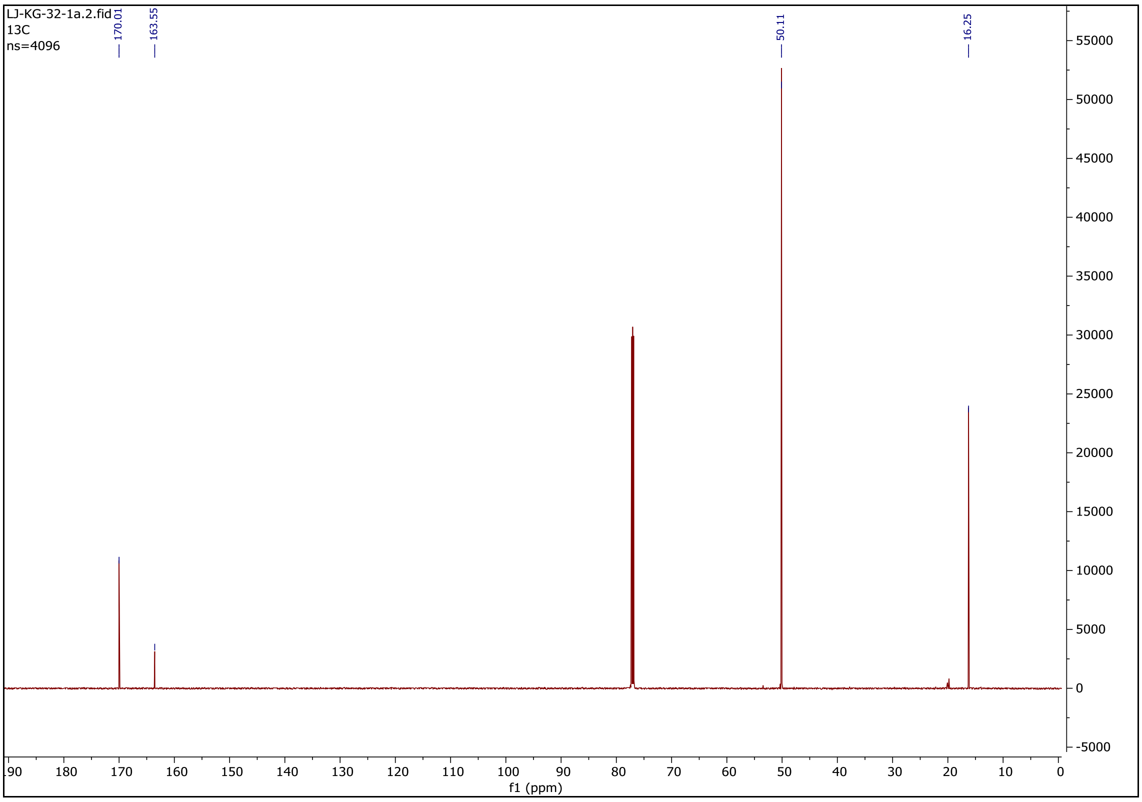

Fig. S13. ^{13}C NMR 3c ($CDCl_3$, 176 MHz)

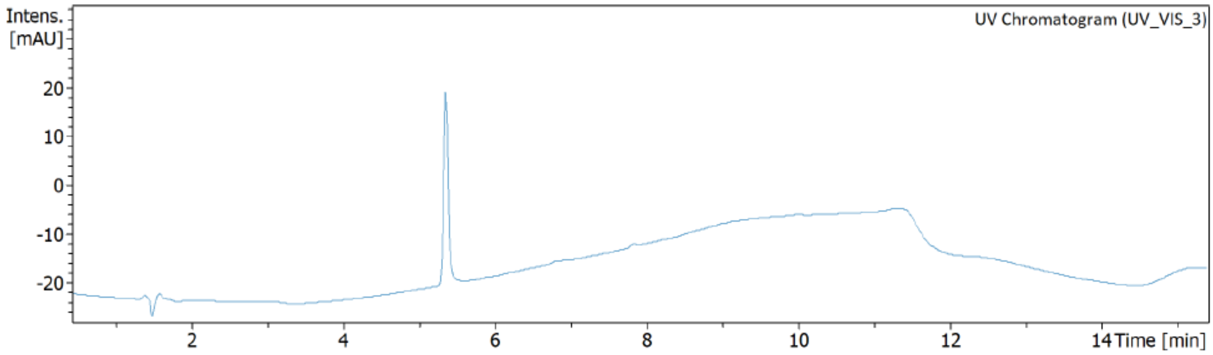

Fig. S14. UV-Vis Chromatogram 3c

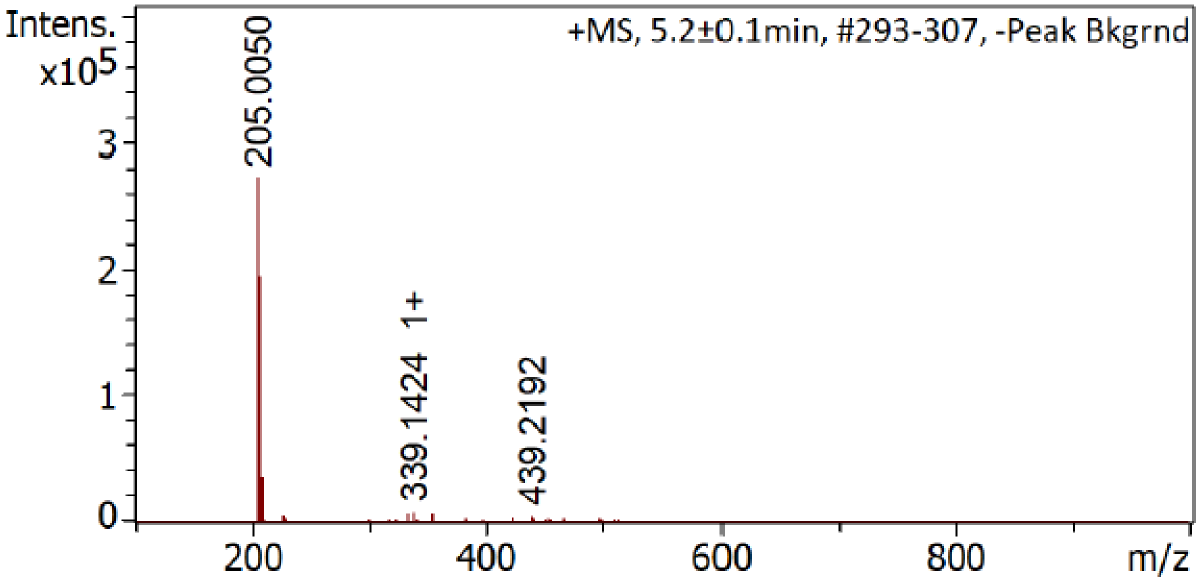

Fig. S15. Mass spectrum 3c

S13. Analytical spectra of 3d

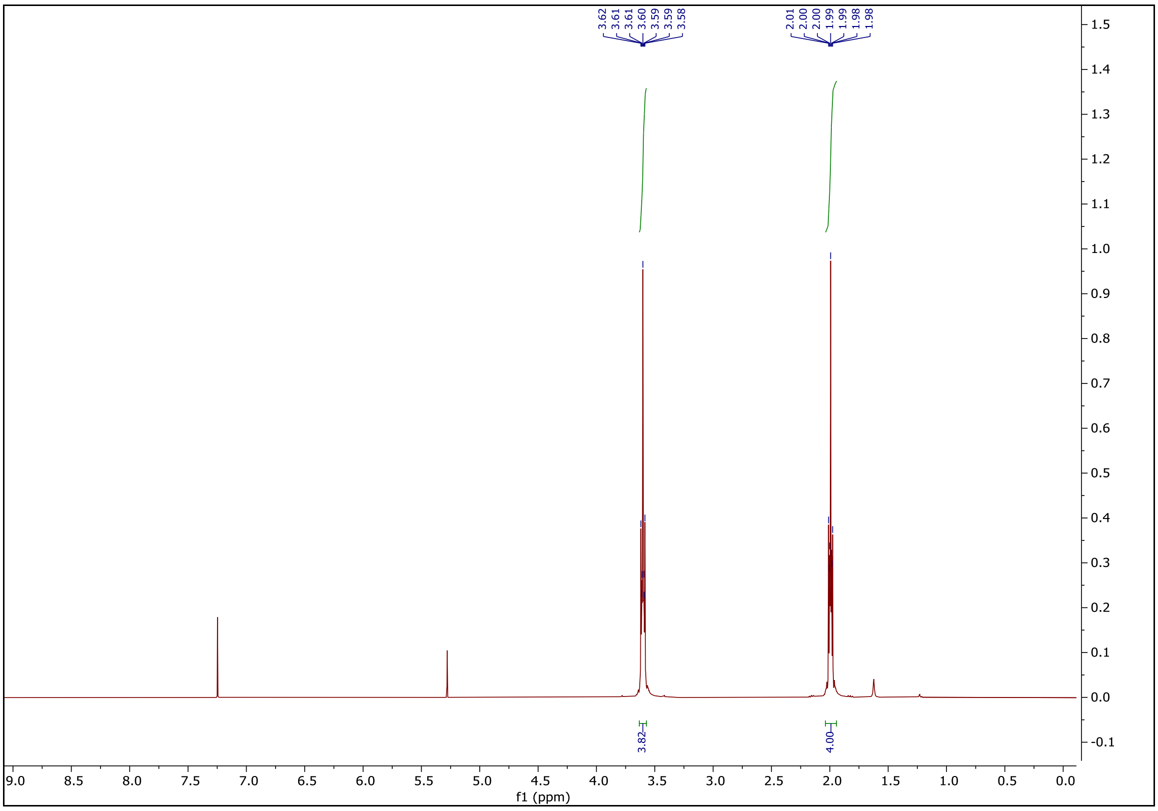

Fig. S16. ^{1}H NMR 3d ($CDCl_3$, 700 MHz).

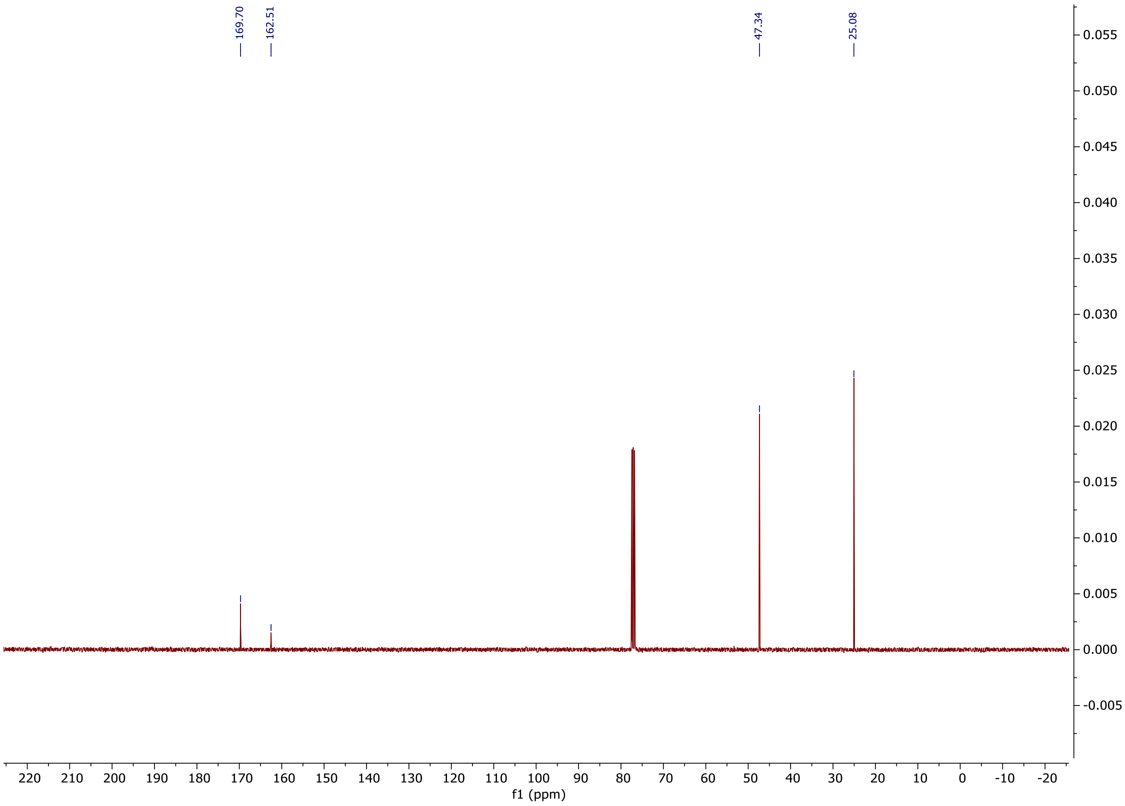

Fig. S17. ^{13}C NMR 3d ($CDCl_3$, 176 MHz).

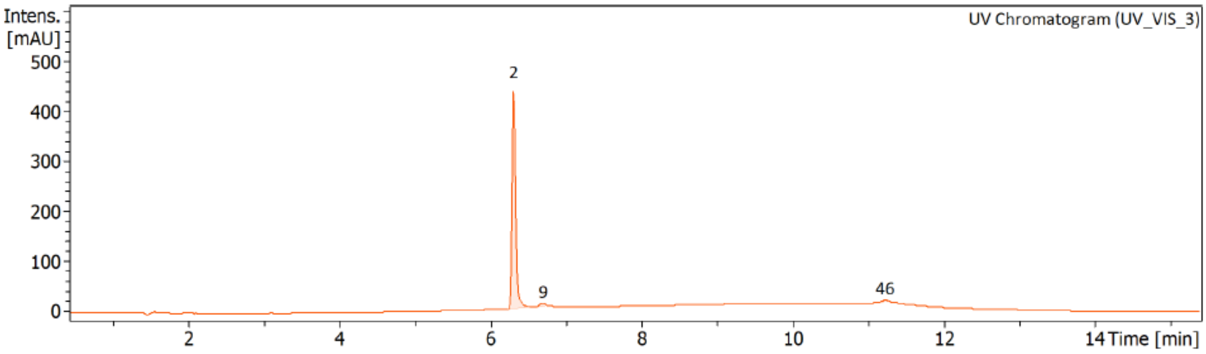

Fig. S18. UV-Vis Chromatogram 3d

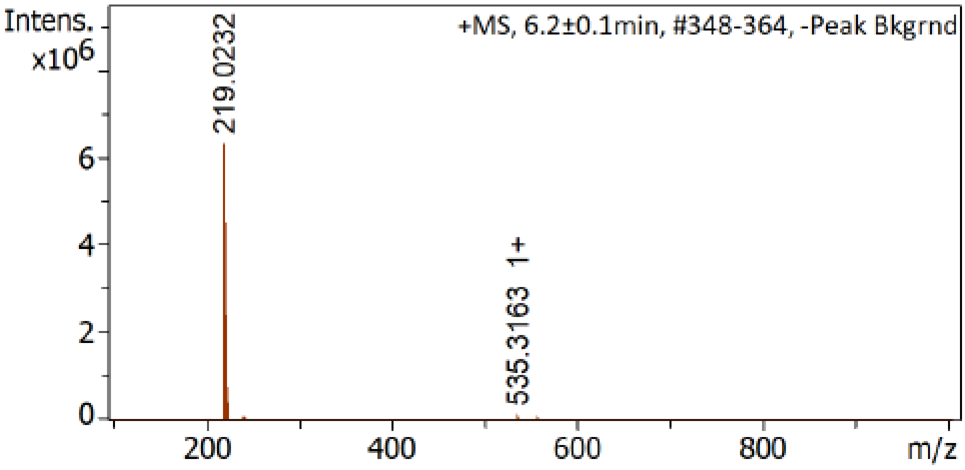

Fig. S19. Mass spectrum 3d

S14. Analytical spectra of 3e

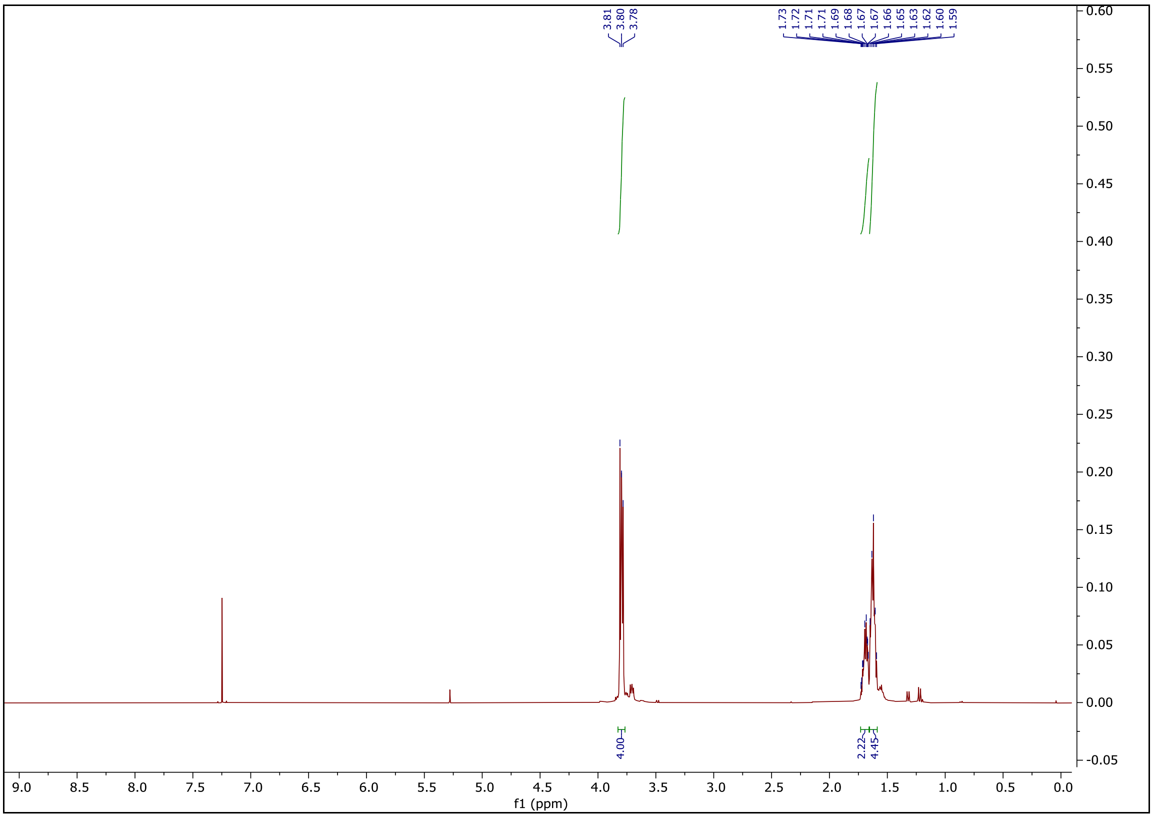

Fig. S20. ^{1}H NMR 3e ($CDCl_3$, 700 MHz).

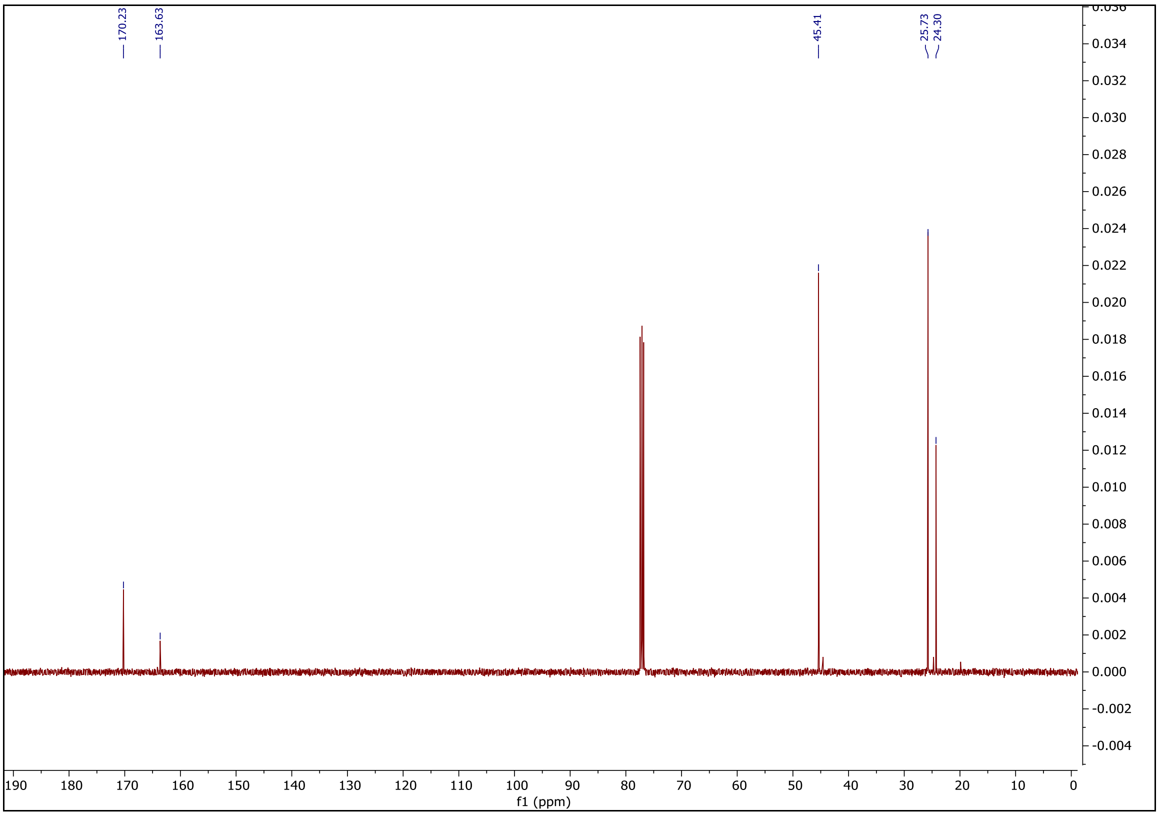

Fig. S21. ^{13}C NMR 3e ($CDCl_3$, 176 MHz).

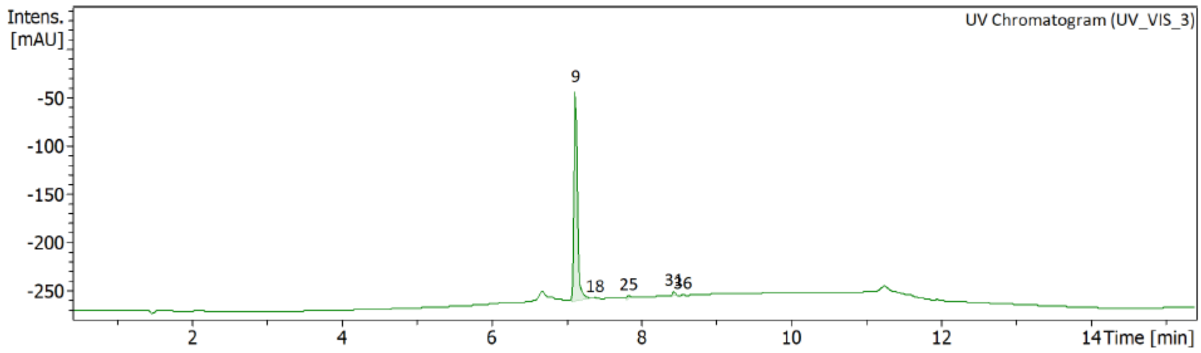

Fig. S22. UV-Vis Chromatogram 3e

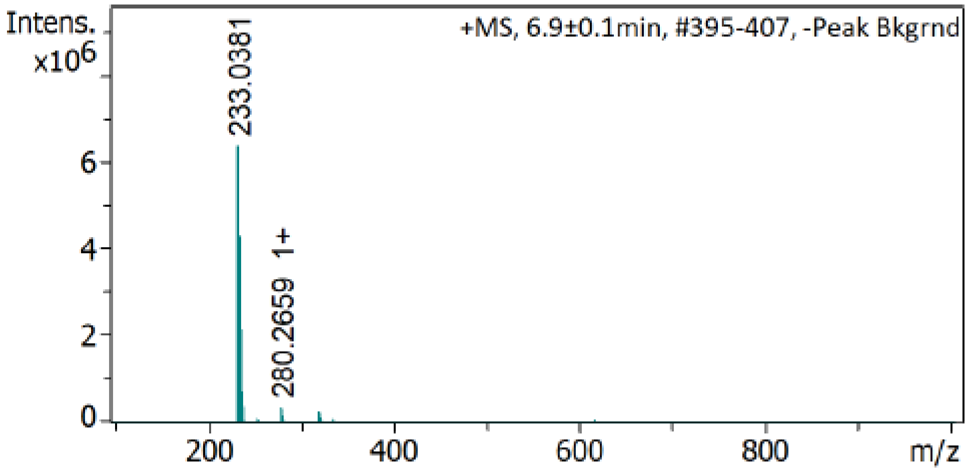

Fig. S23. Mass spectrum 3e

S15. Analytical spectra of 3f

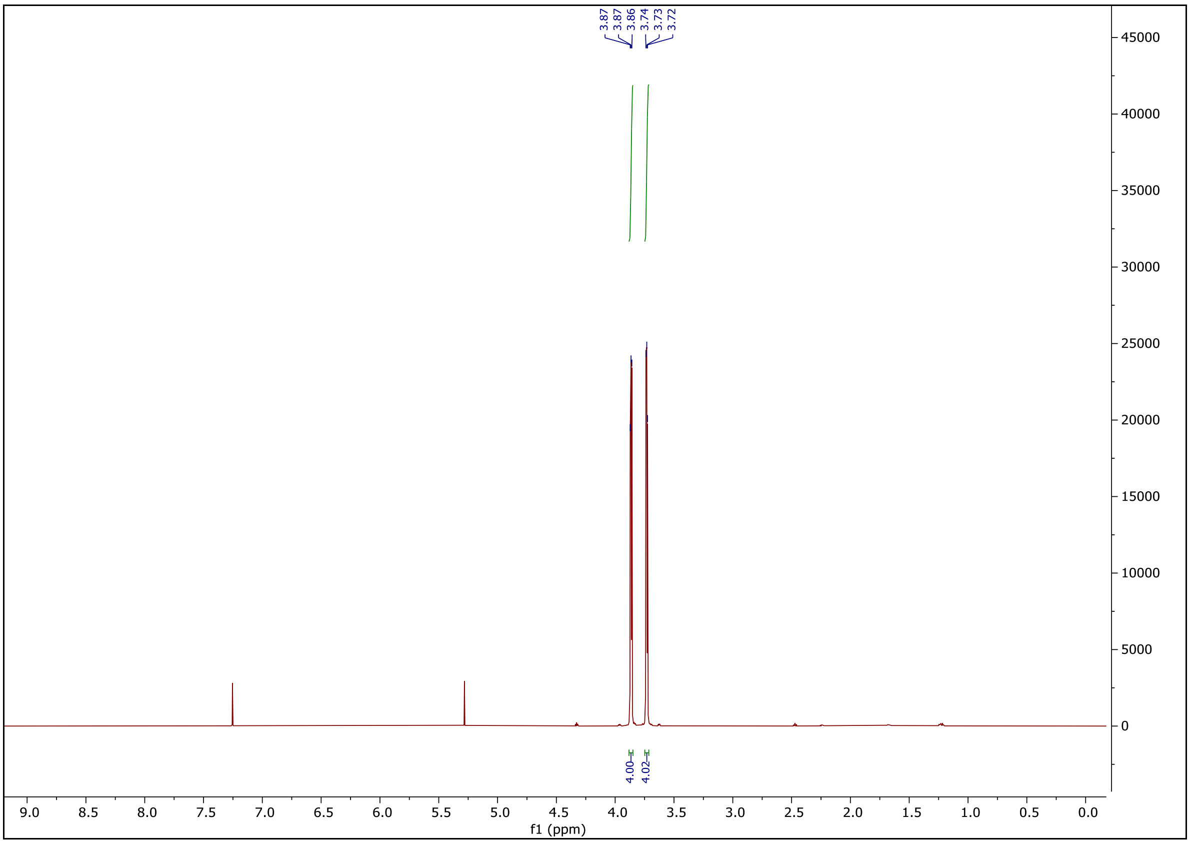

Fig. S24. ^{1}H NMR 3f ($CDCl_3$, 700 MHz).

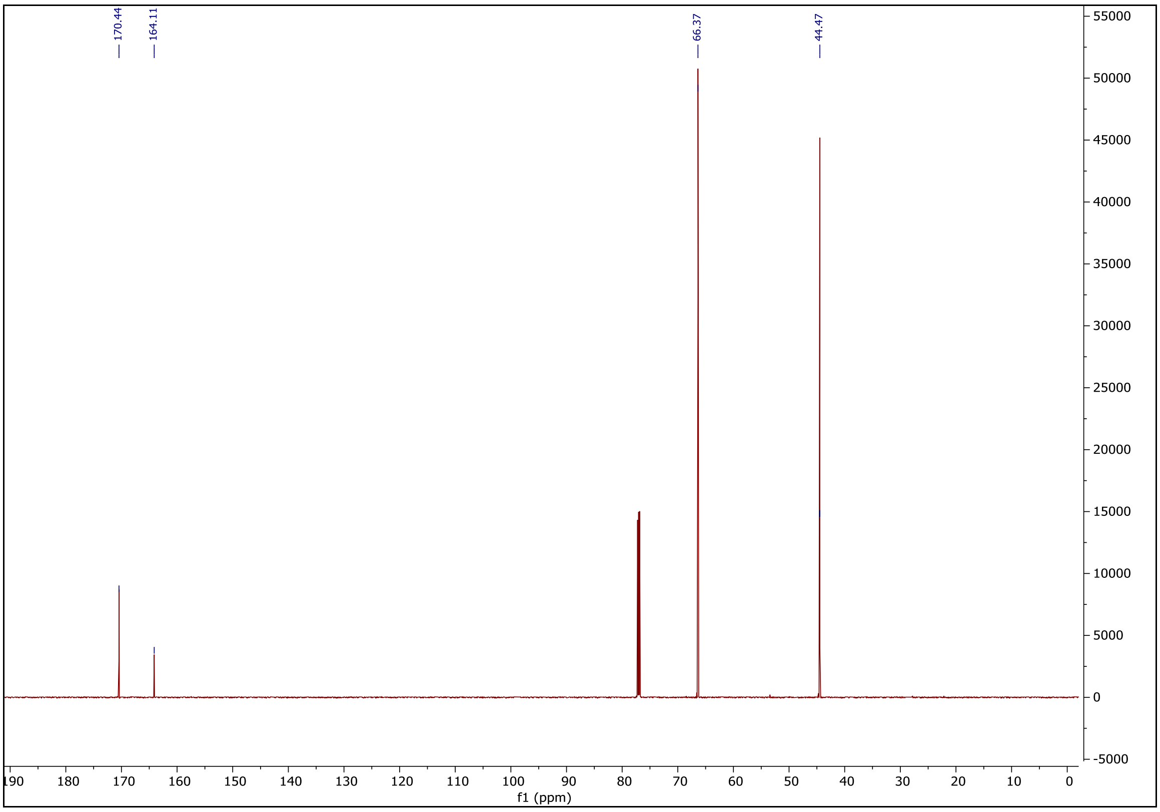

Fig. S25. ^{13}C NMR 3f ($CDCl_3$, 176 MHz).

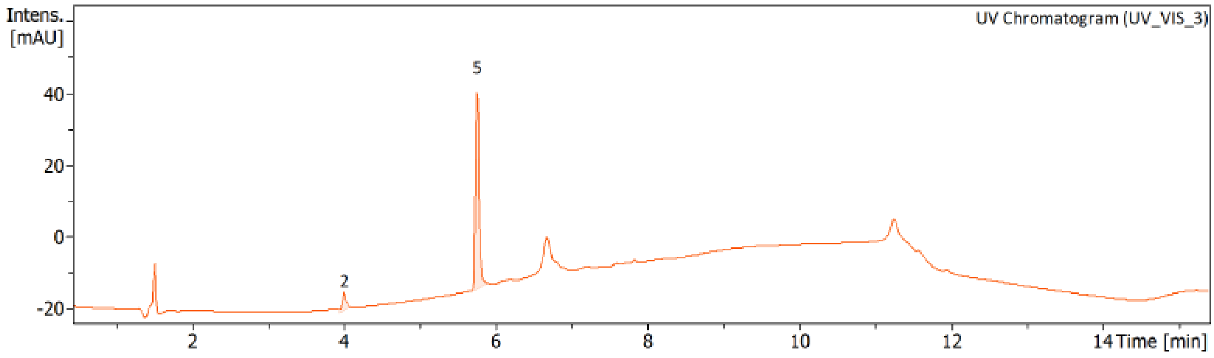

Fig. S26. UV-Vis Chromatogram 3f

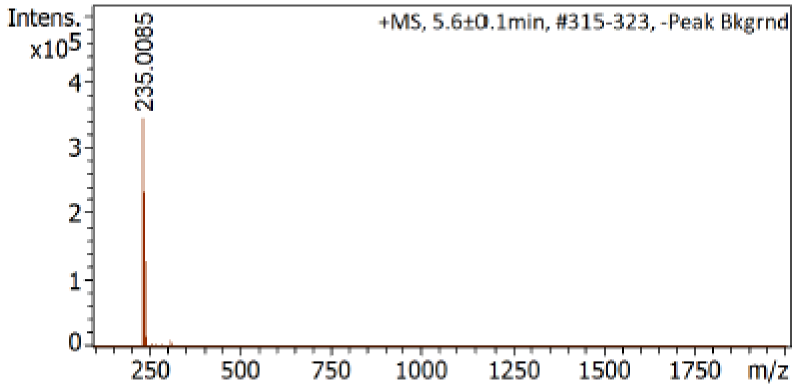

Fig. S27. Mass spectrum 3f.

S16. Analytical spectra of 4a

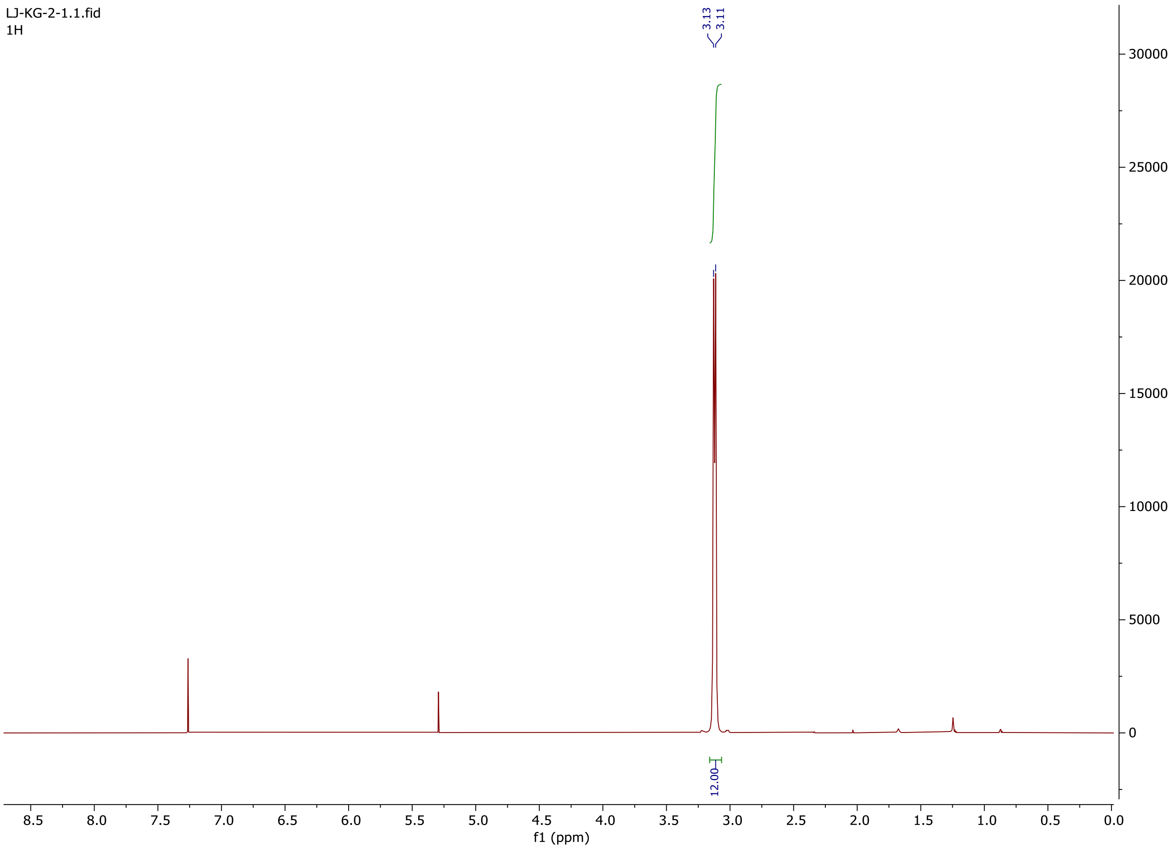

Fig. S28. ^{1}H NMR 4a ($CDCl_3$, 700 MHz).

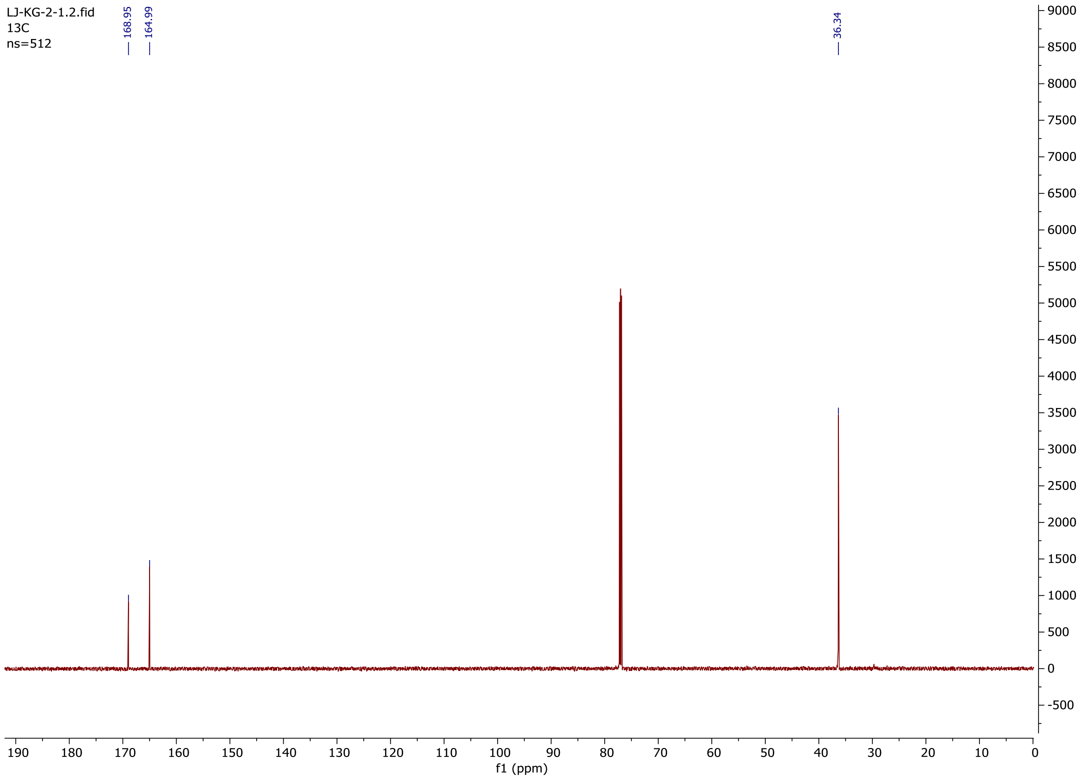

Fig. S29. ^{13}C NMR 4a ($CDCl_3$, 176 MHz).

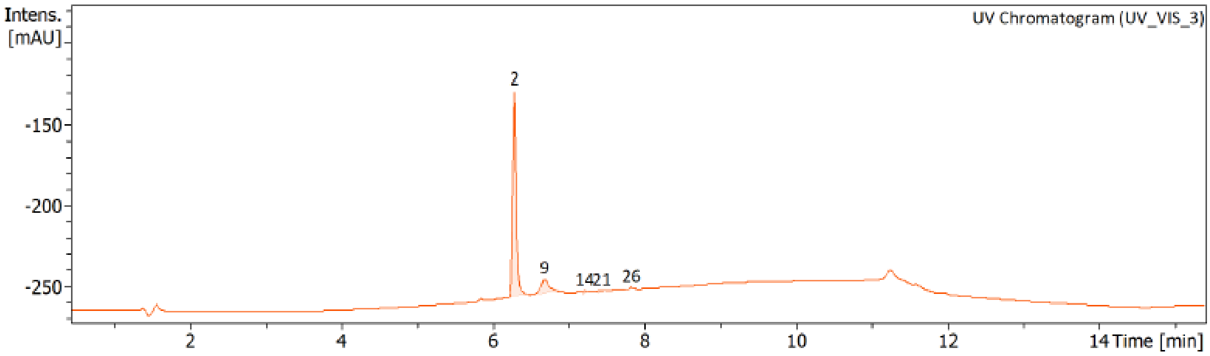

Fig. S30. UV-Vis Chromatogram 4a

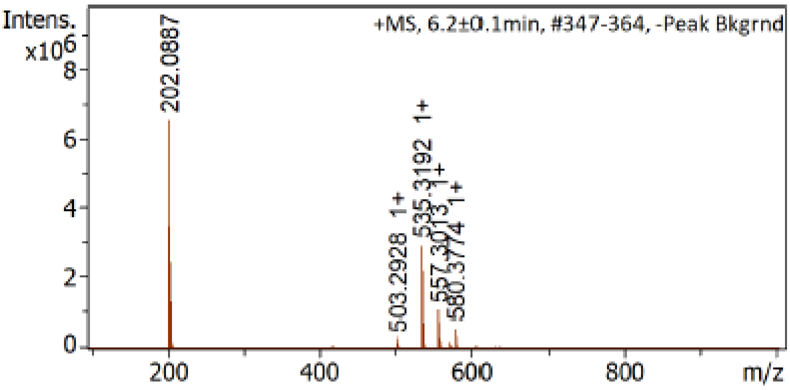

Fig. S31. Mass spectrum 4a

S17. Analytical spectra of 4b

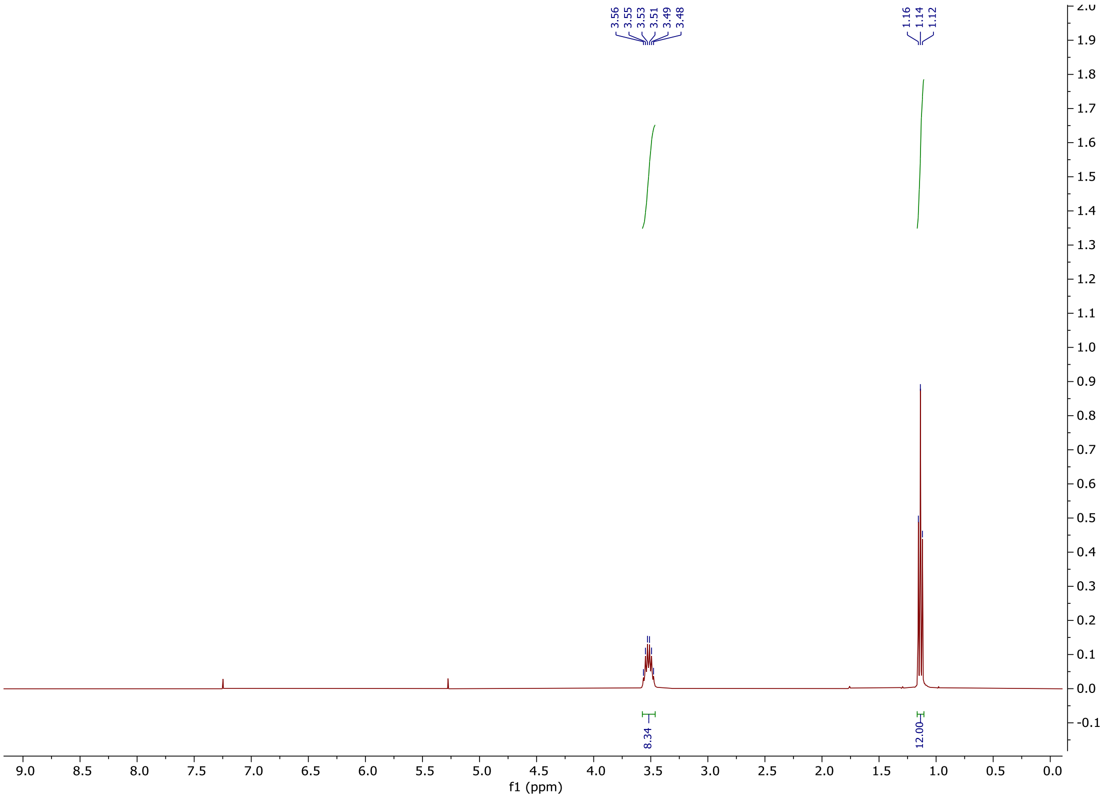

Fig. S32. ^{1}H NMR 4b ($CDCl_3$, 700 MHz).

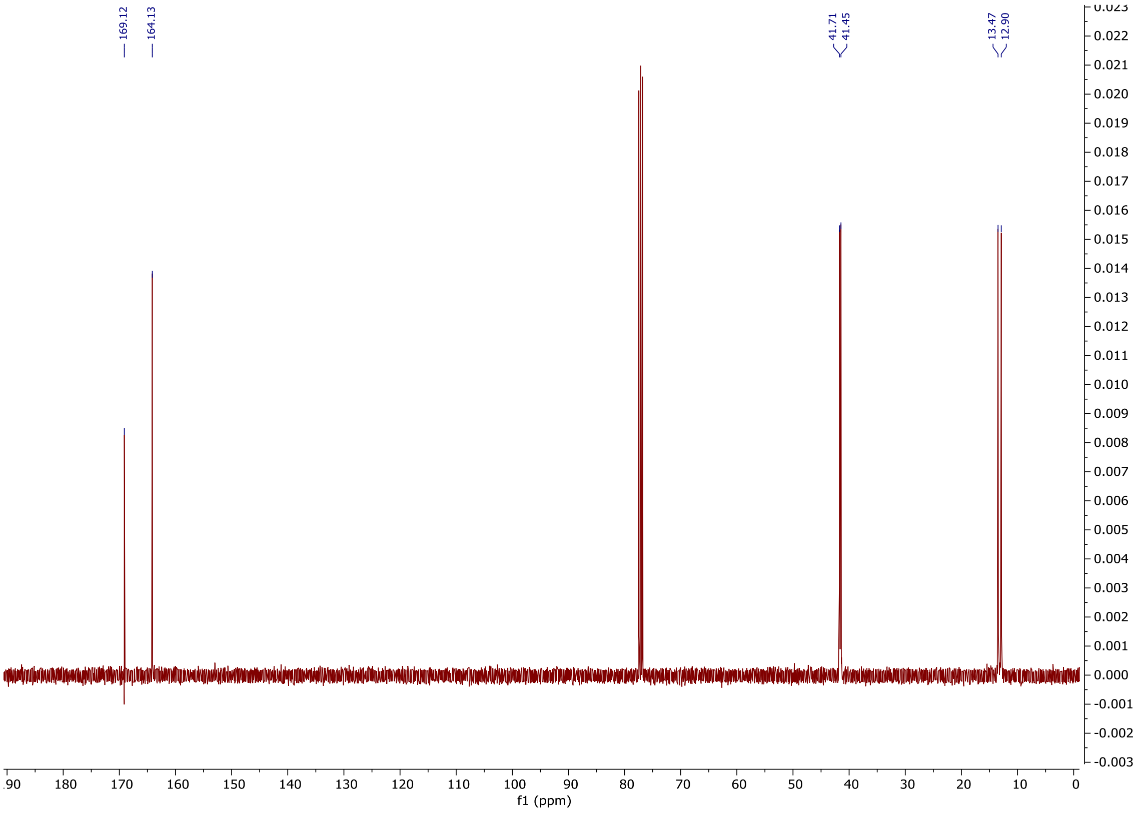

Fig. S33. ^{13}C NMR 4b ($CDCl_3$, 176 MHz).

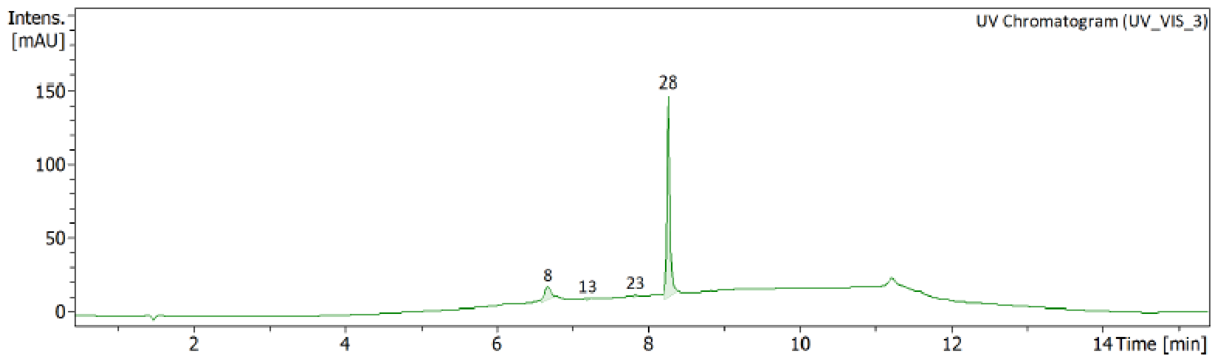

Fig. S34. UV-Vis Chromatogram 4b

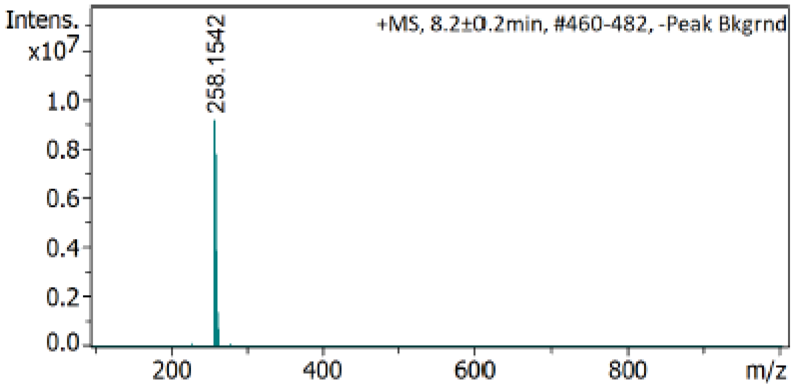

Fig. S35. Mass spectrum 4b

S18. Analytical spectra of 4c

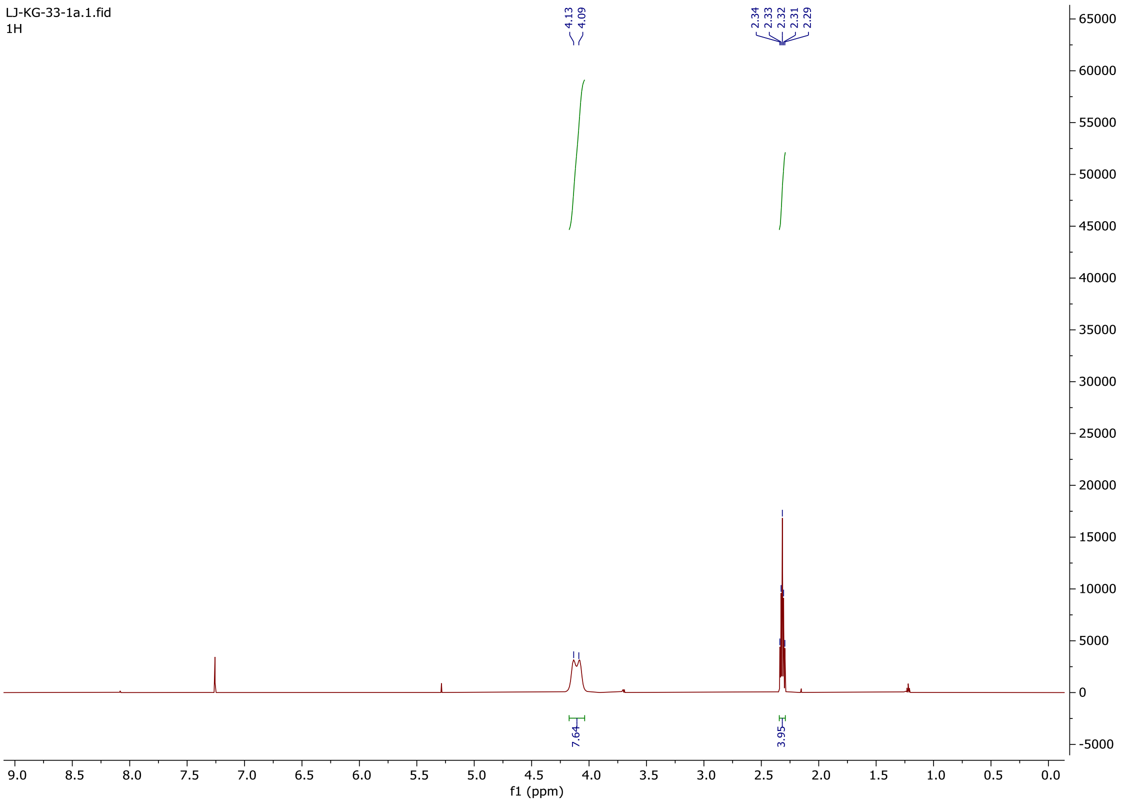

Fig. S36. ^{1}H NMR 4c ($CDCl_3$, 700 MHz).

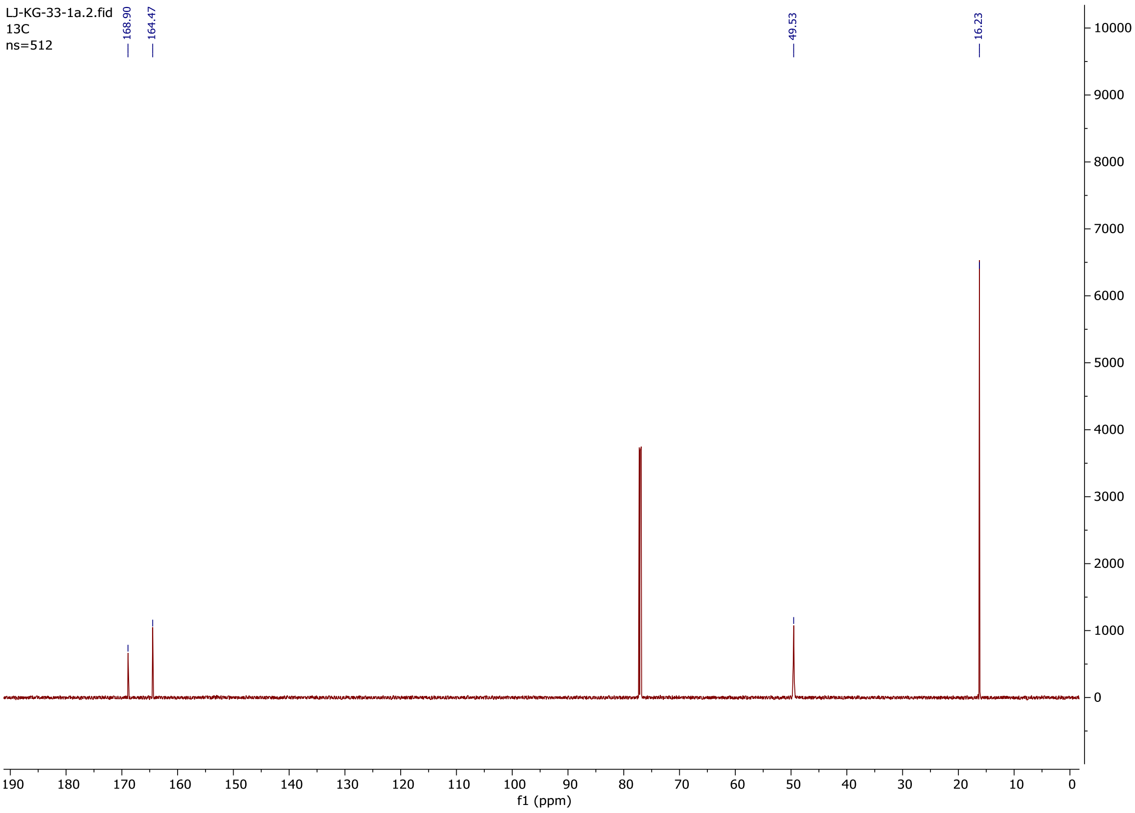

Fig. S37. ^{13}C NMR 4c ($CDCl_3$, 176 MHz).

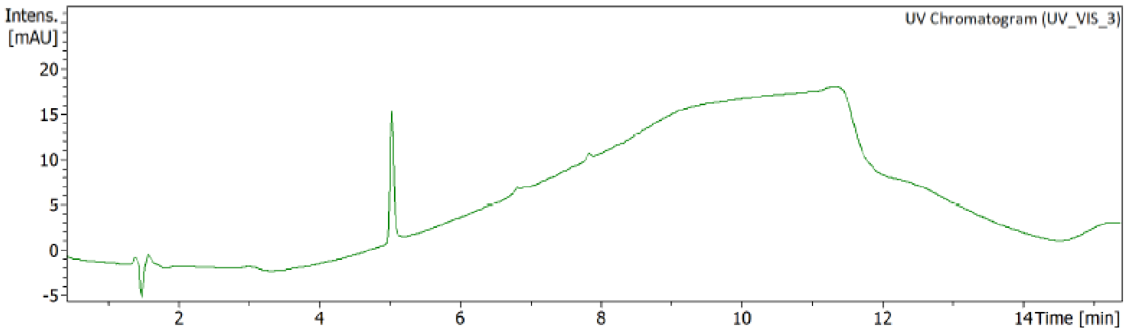

Fig. S38. UV-Vis Chromatograph 4c

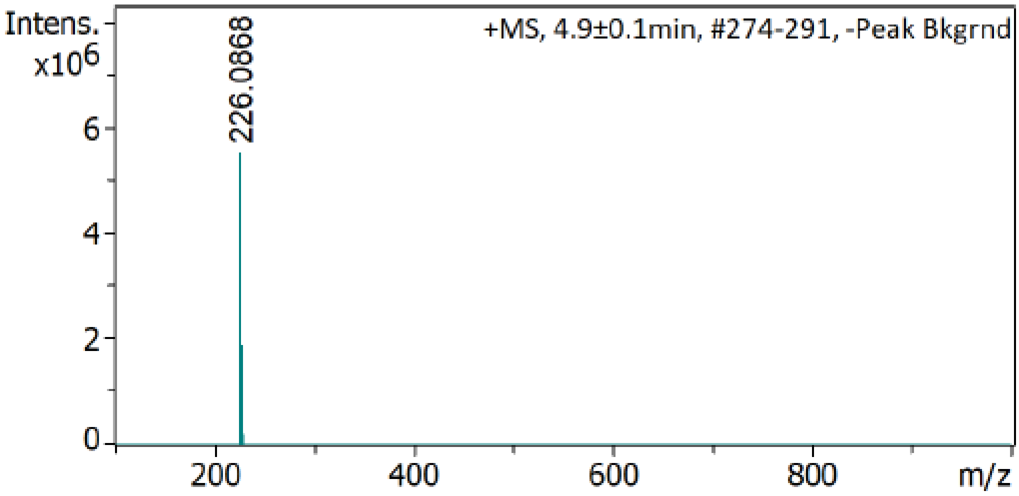

Fig. S39. Mass spectrum 4c

S19. **Analytical spectra of 4d**

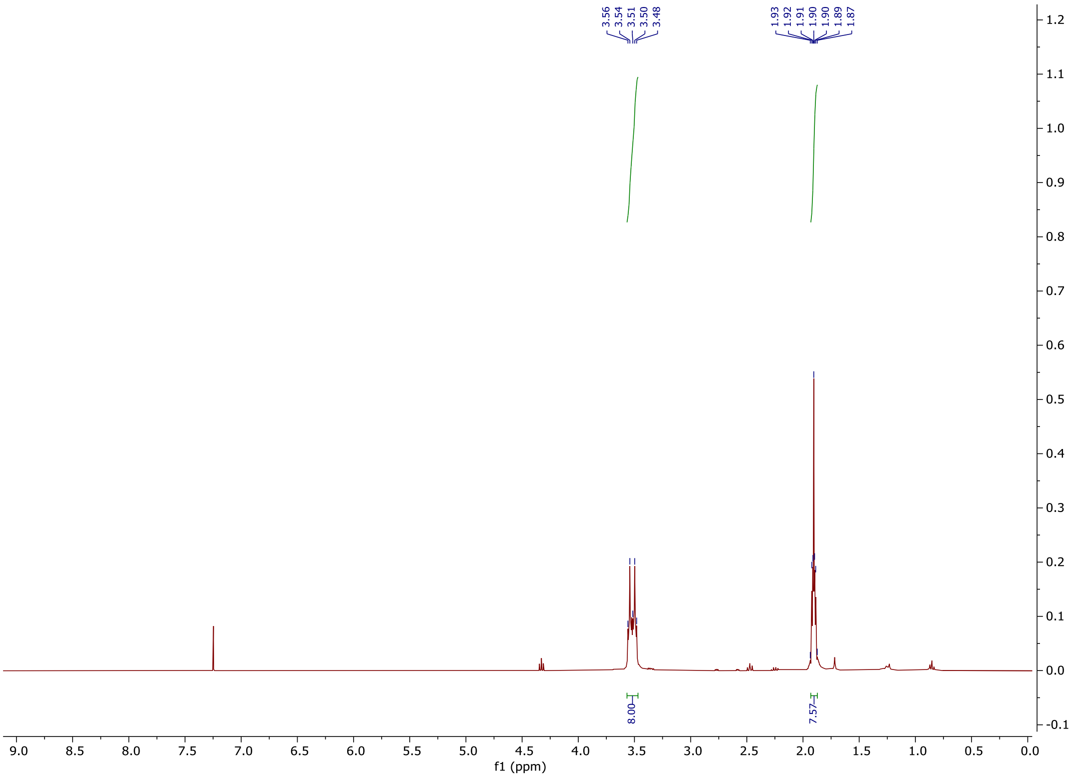

Fig. S40. ^{1}H NMR 4d ($CDCl_3$, 700 MHz).

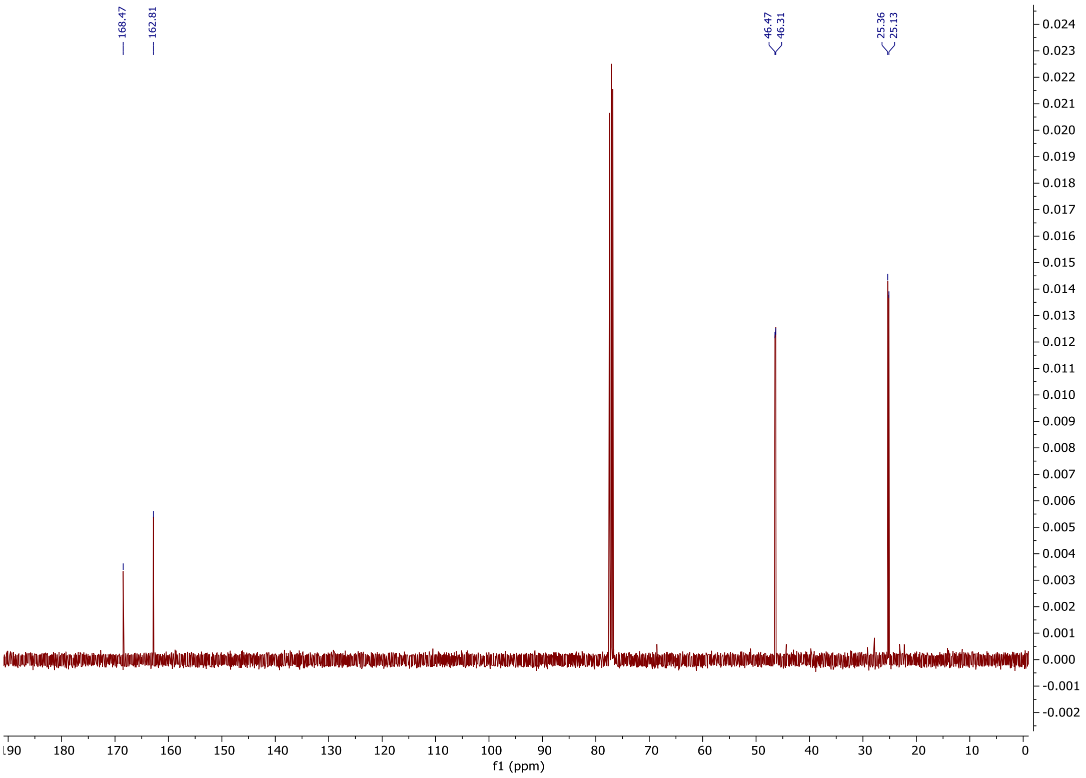

Fig. S41. ^{13}C NMR 4d ($CDCl_3$, 176 MHz).

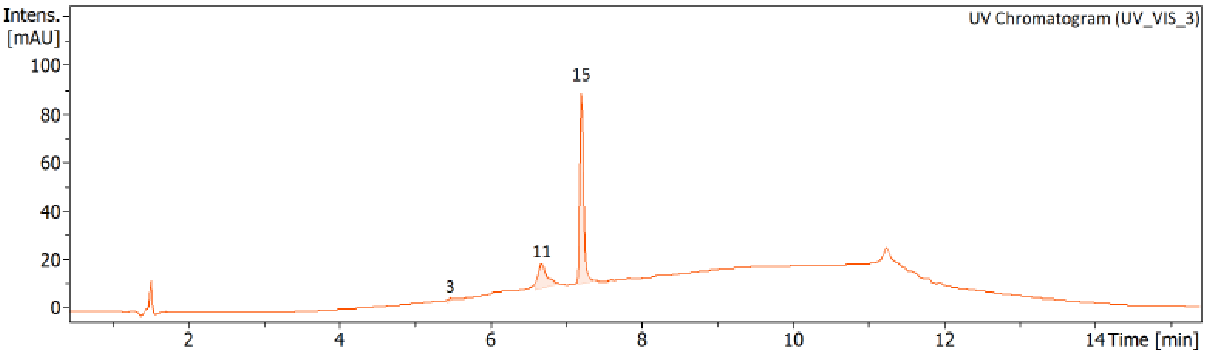

Fig. S42. UV-Vis Chromatogram 4d

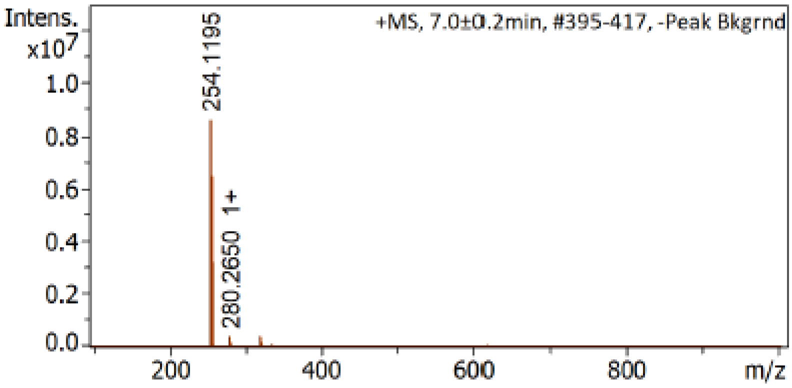

Fig. S43. Mass spectrum 4d

S20. Analytical spectra of 4e

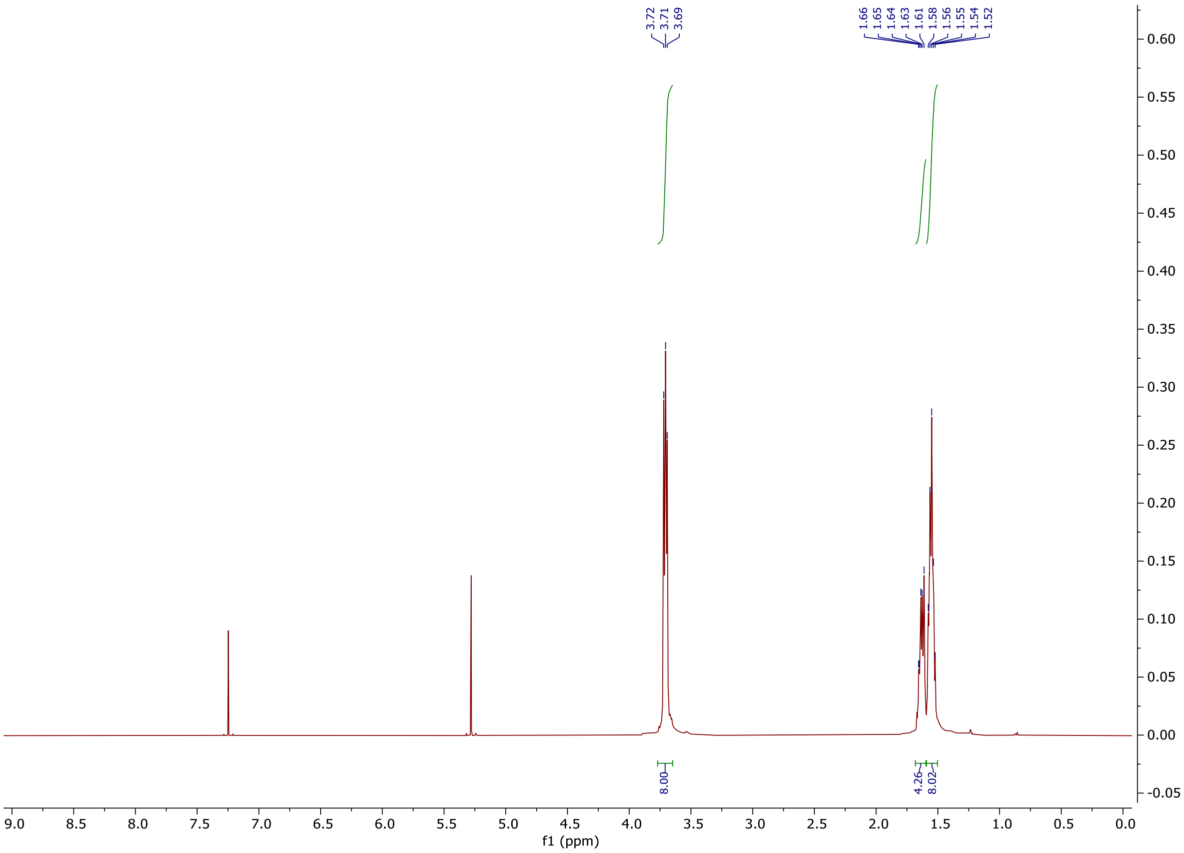

Fig. S44. ^{1}H NMR 4e ($CDCl_3$, 700 MHz).

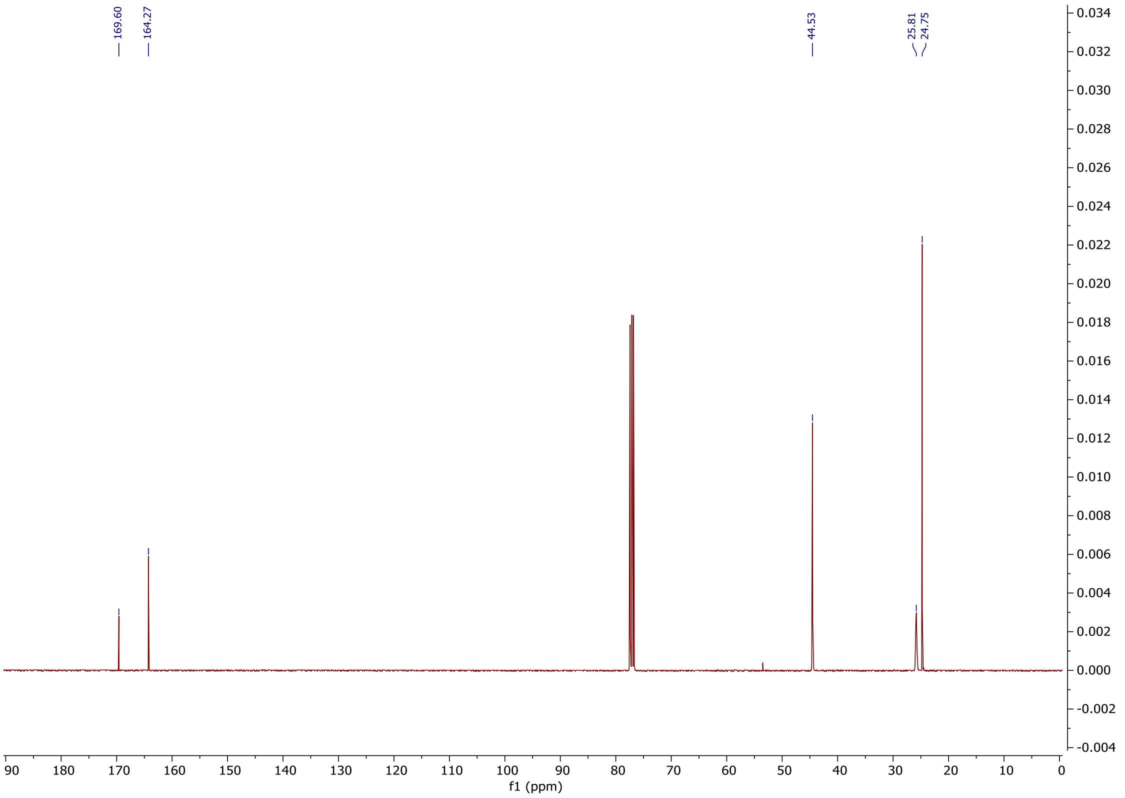

Fig. S45. ^{13}C NMR 4e ($CDCl_3$, 176 MHz).

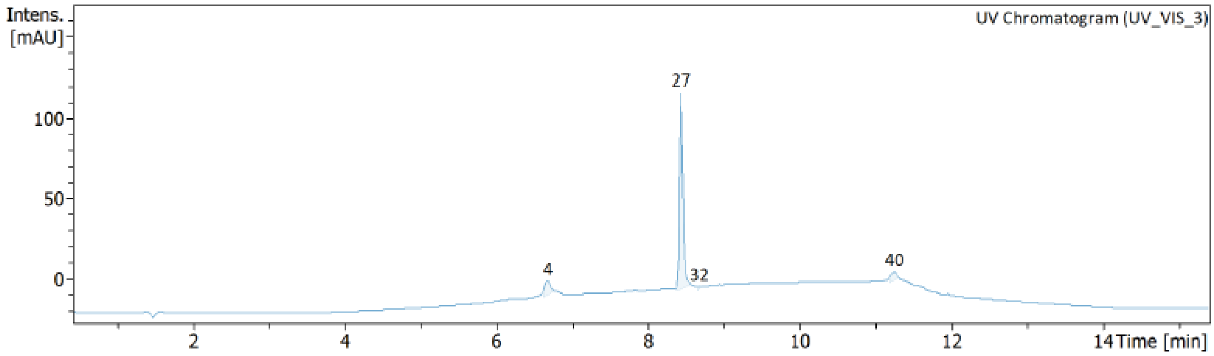

Fig. S46. UV-Vis Chromatogram 4e

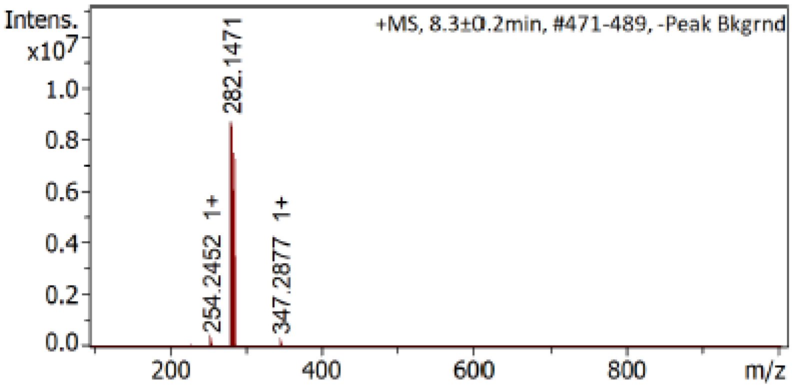

Fig. S47. Mass spectrum 4e

S21. **Analytical spectra of 4f**

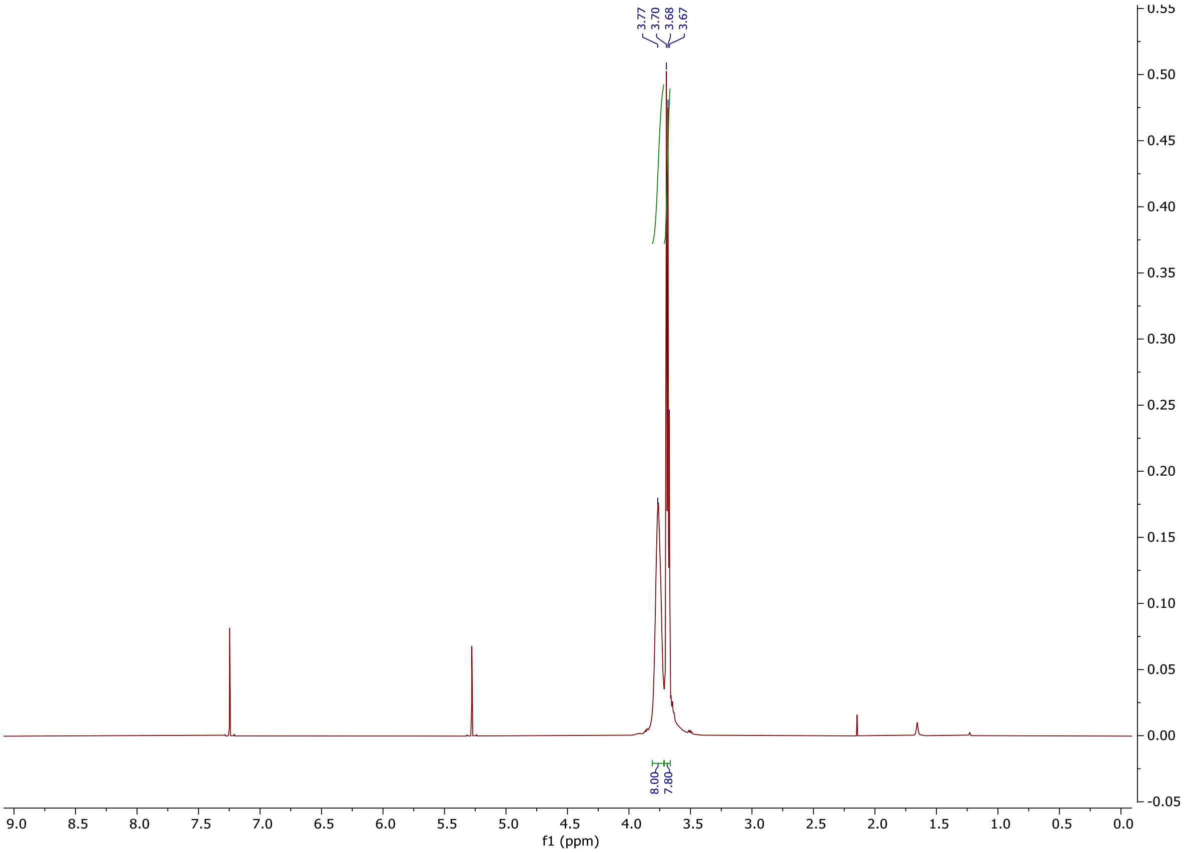

Fig. S48. ^{1}H NMR 4f ($CDCl_3$, 700 MHz).

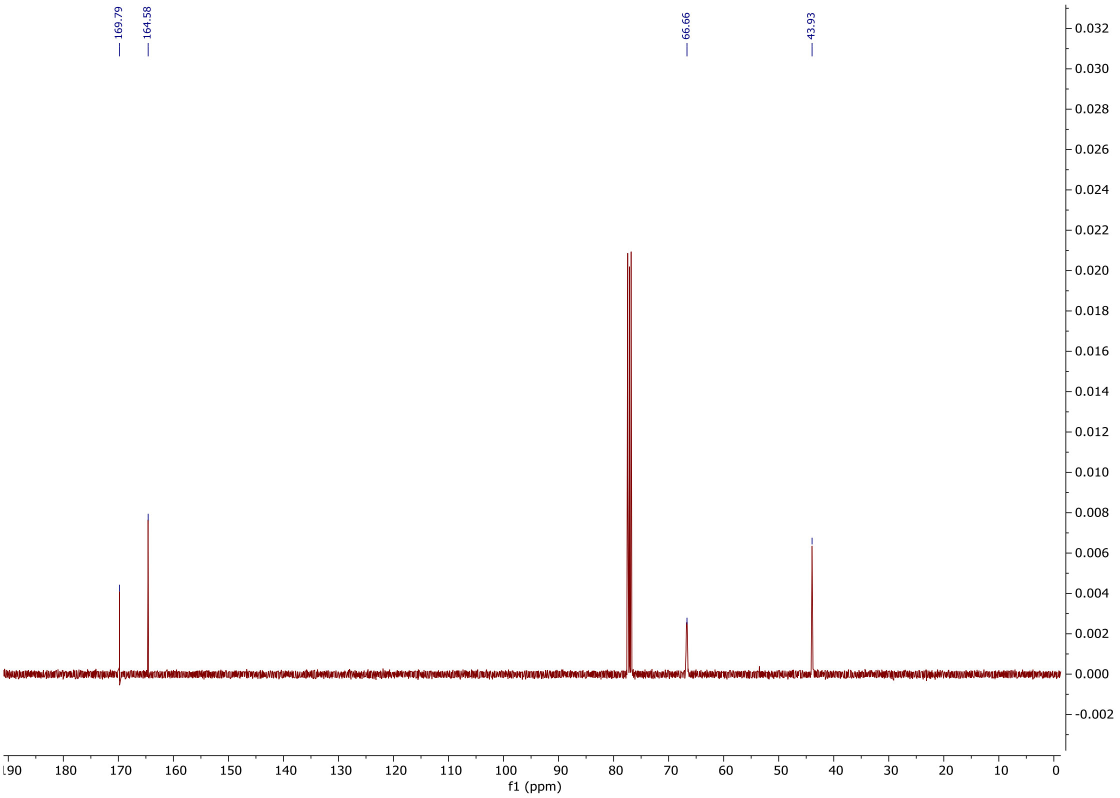

Fig. S49. ^{13}C NMR 4f ($CDCl_3$, 176 MHz).

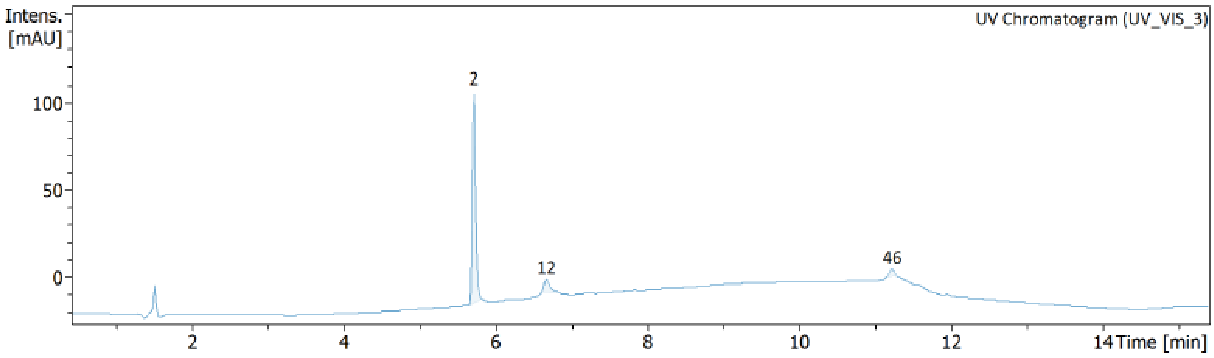

Fig. S50. UV-Vis Chromatogram 4f

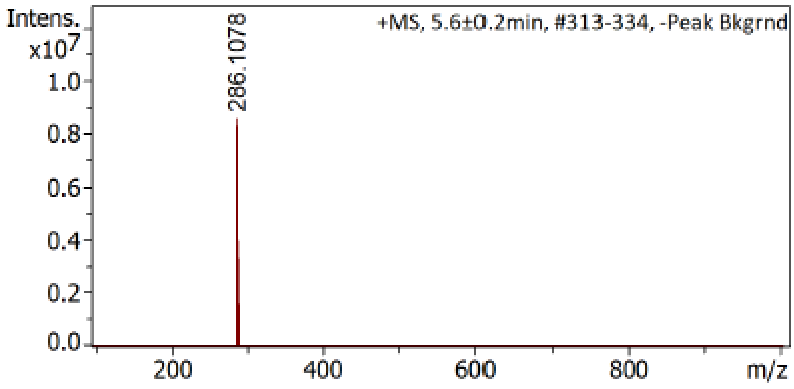

Fig. S51. Mass spectrum 4f

S22. Analytical spectra of 4g

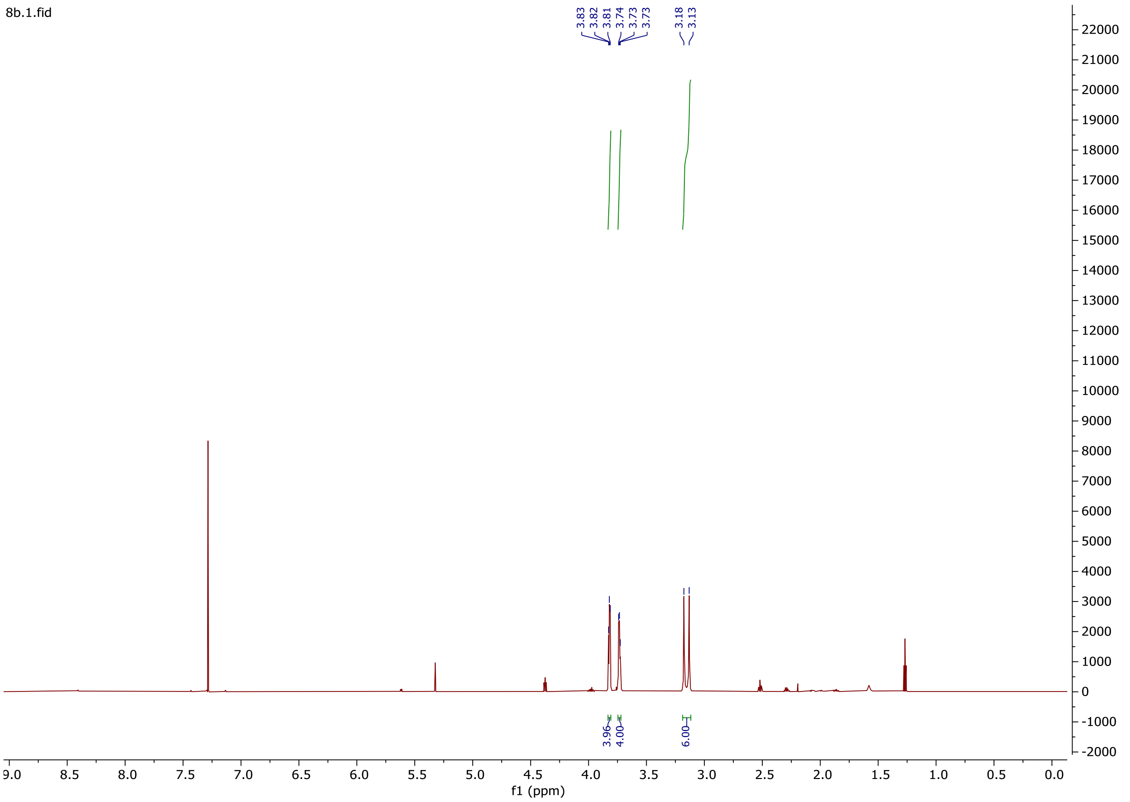

Fig. S52. ^{1}H NMR 4g ($CDCl_3$, 700 MHz).

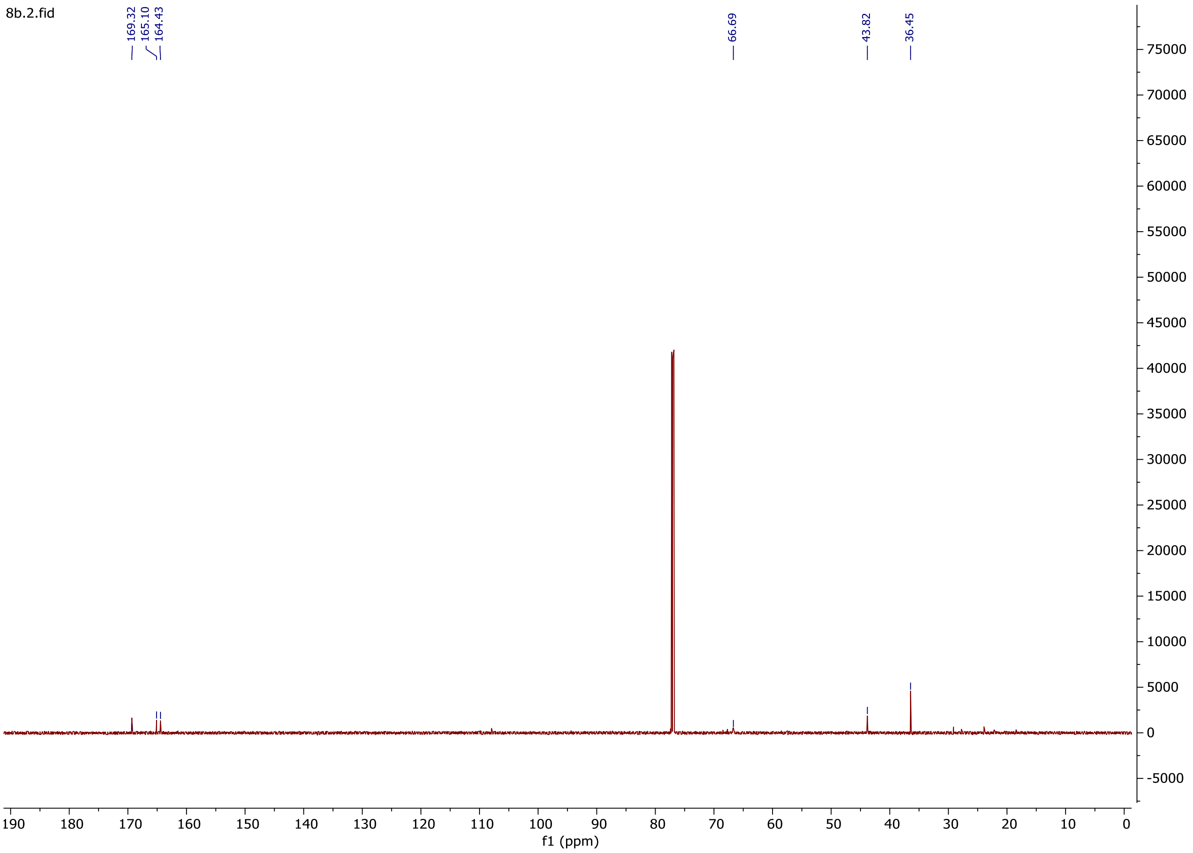

Fig. S53. ^{13}C NMR 4g ($CDCl_3$, 176 MHz).

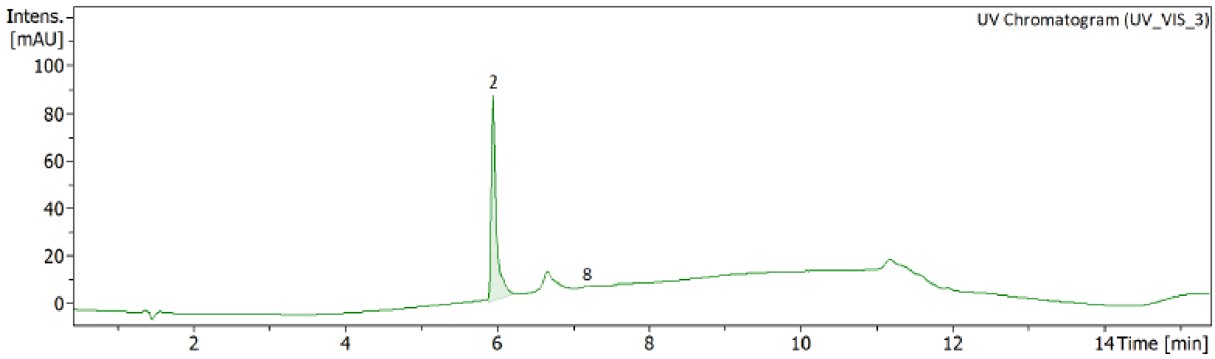

Fig. S54. UV-Vis Chromatogram 4g

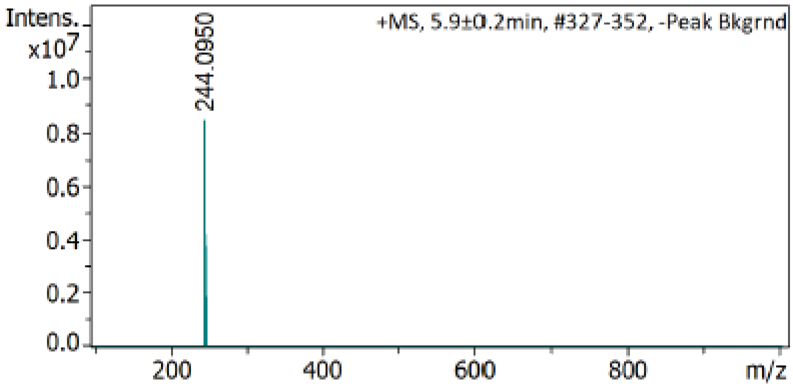

Fig. S55. Mass spectrum 4g

S23. Analytical spectra of **4h**

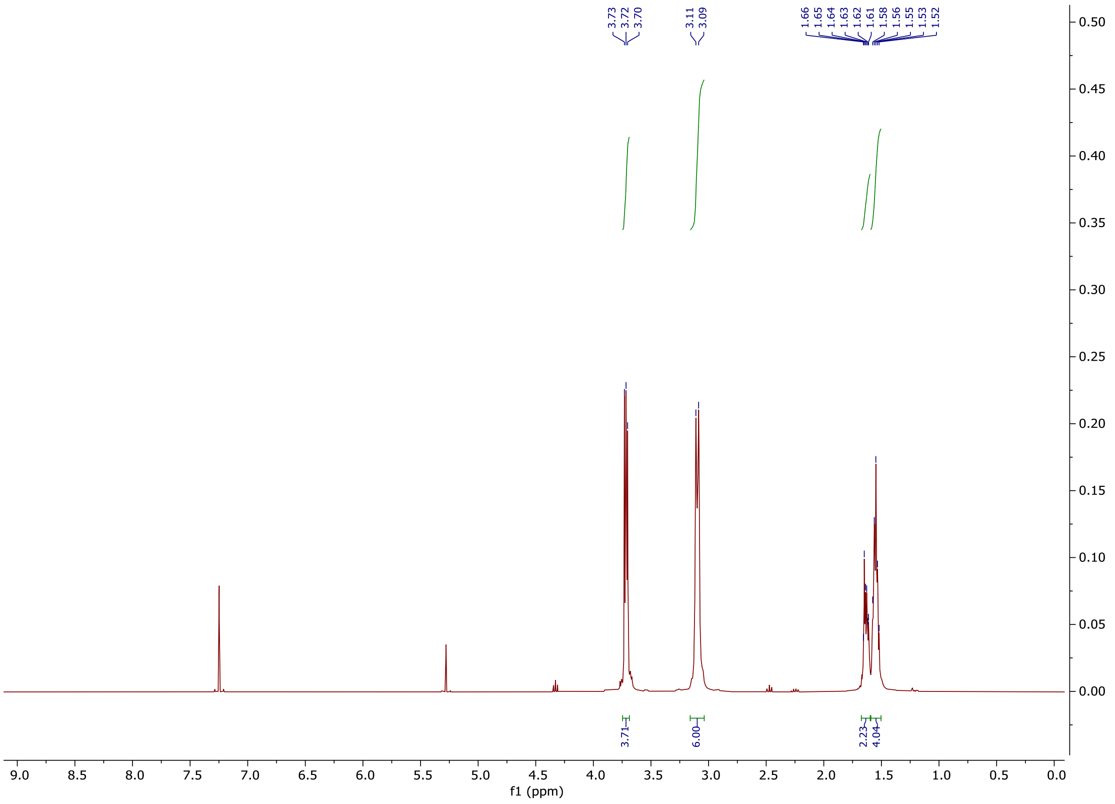

Fig. S56. ^{1}H NMR 4h ($CDCl_3$, 700 MHz).

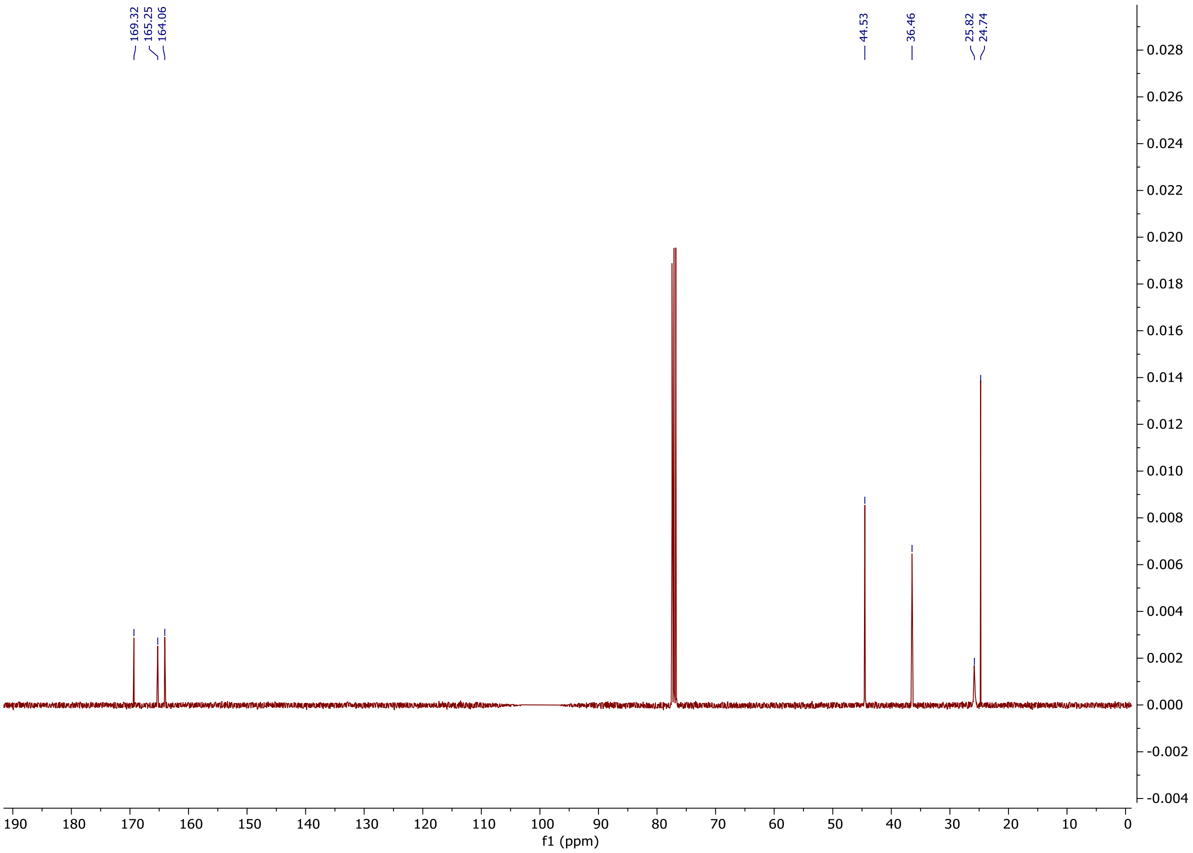

Fig. S57. ^{13}C NMR 4h ($CDCl_3$, 176 MHz).

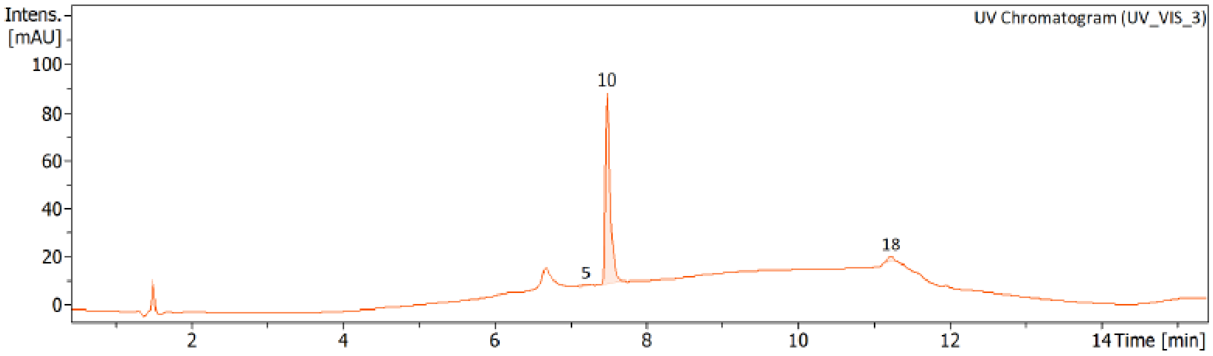

Fig. S58. UV-Vis Chromatogram 4h

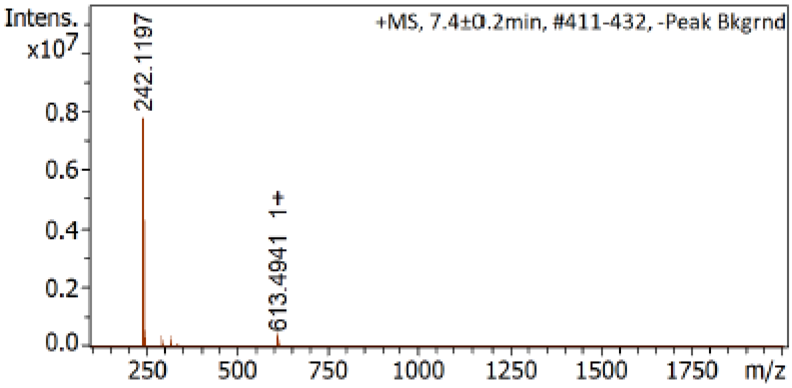

Fig. S59. Mass spectrum 4h

S24. Analytical spectra of 4i

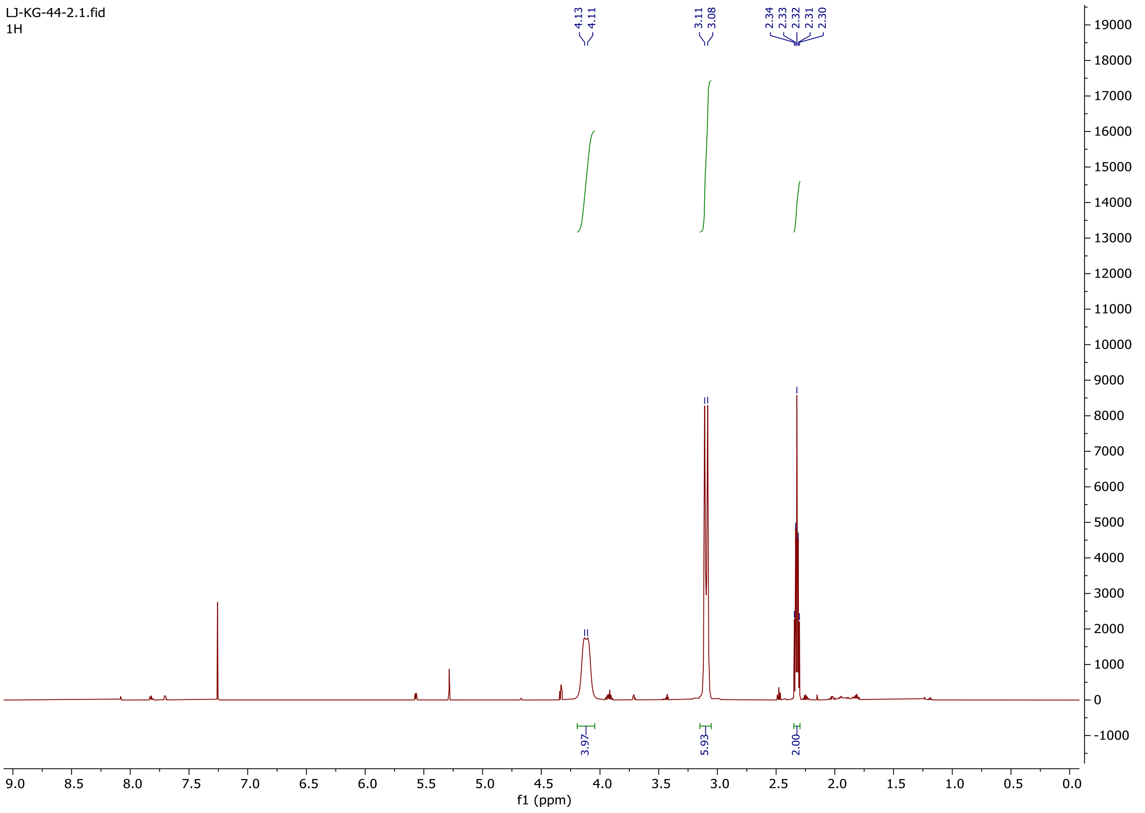

Fig. S60. ^{1}H NMR 4i ($CDCl_3$, 700 MHz).

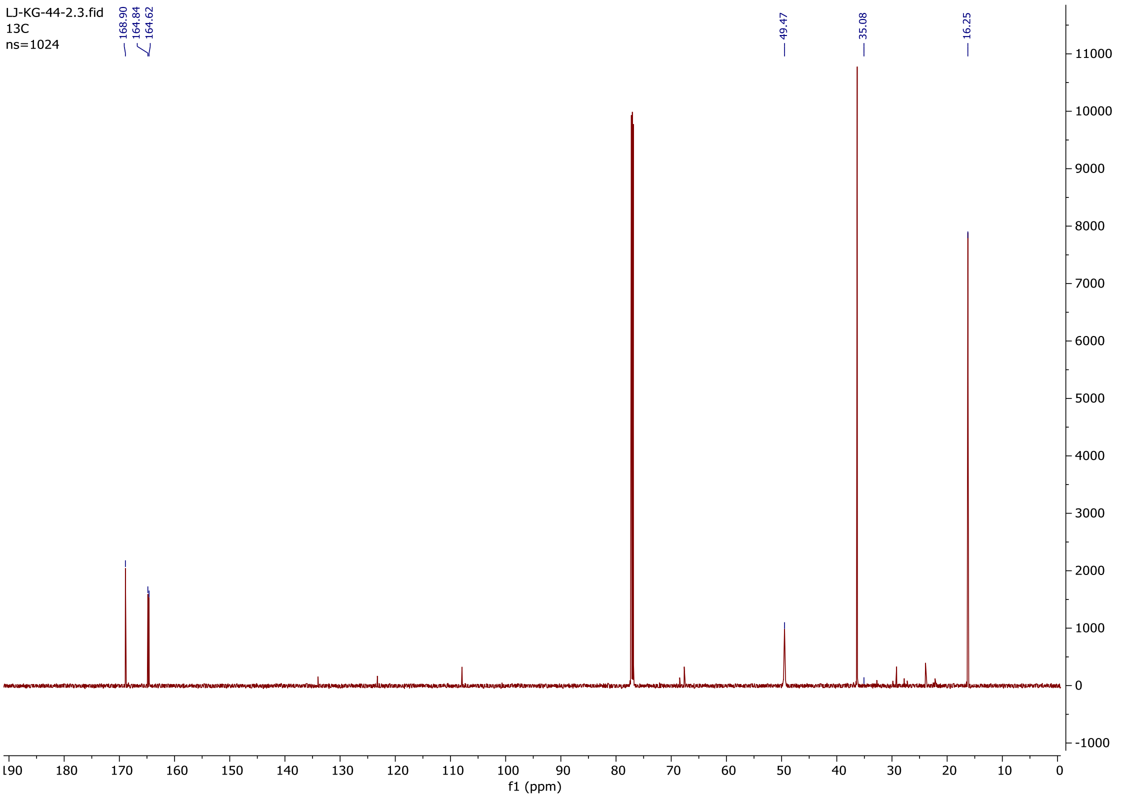

Fig. S61. ^{13}C NMR 4i ($CDCl_3$, 176 MHz).

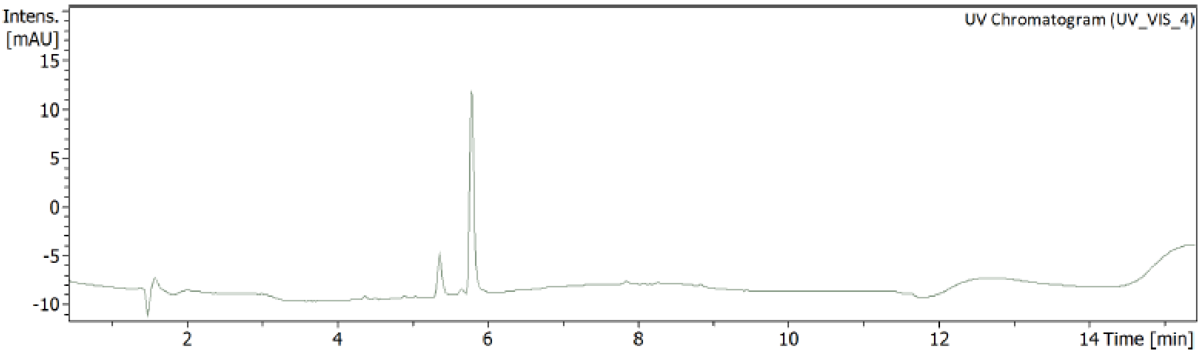

Fig. S62. UV-Vis Chromatograph 4i

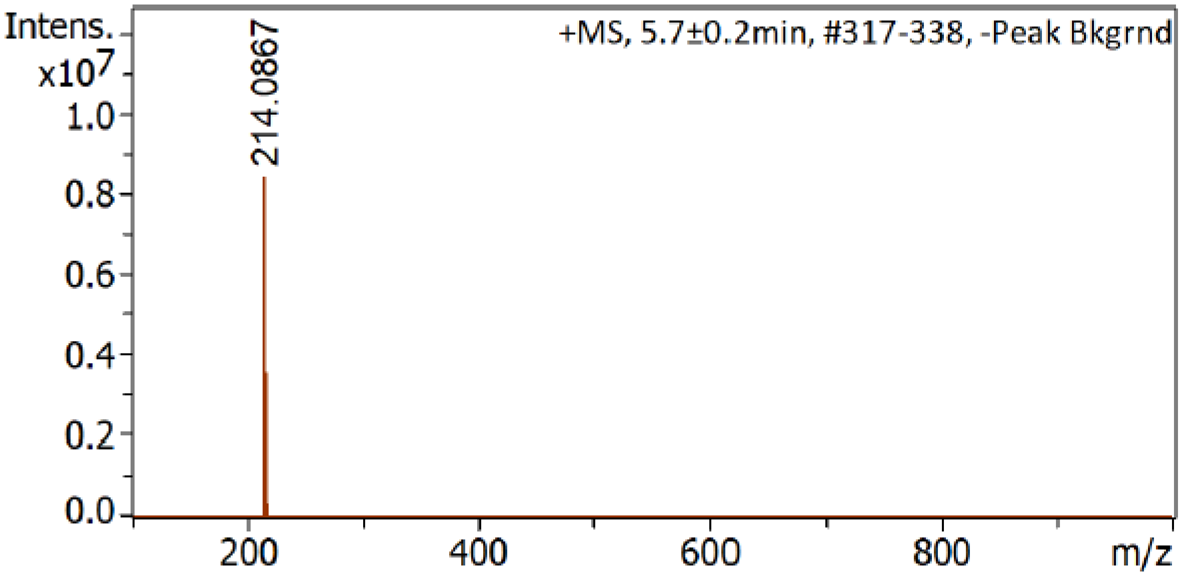

Fig. S63. Mass spectrum 4i

S25. Analytical spectra of 4j

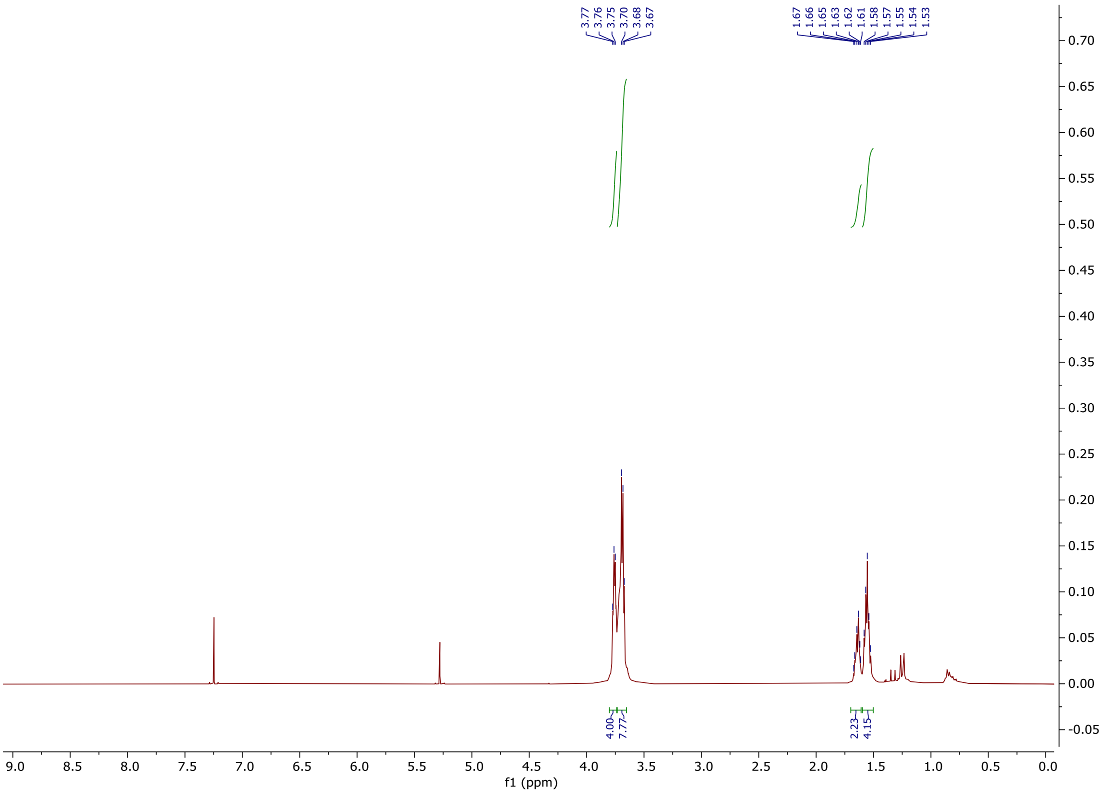

Fig. S64. ^{1}H NMR 4j ($CDCl_3$, 700 MHz).

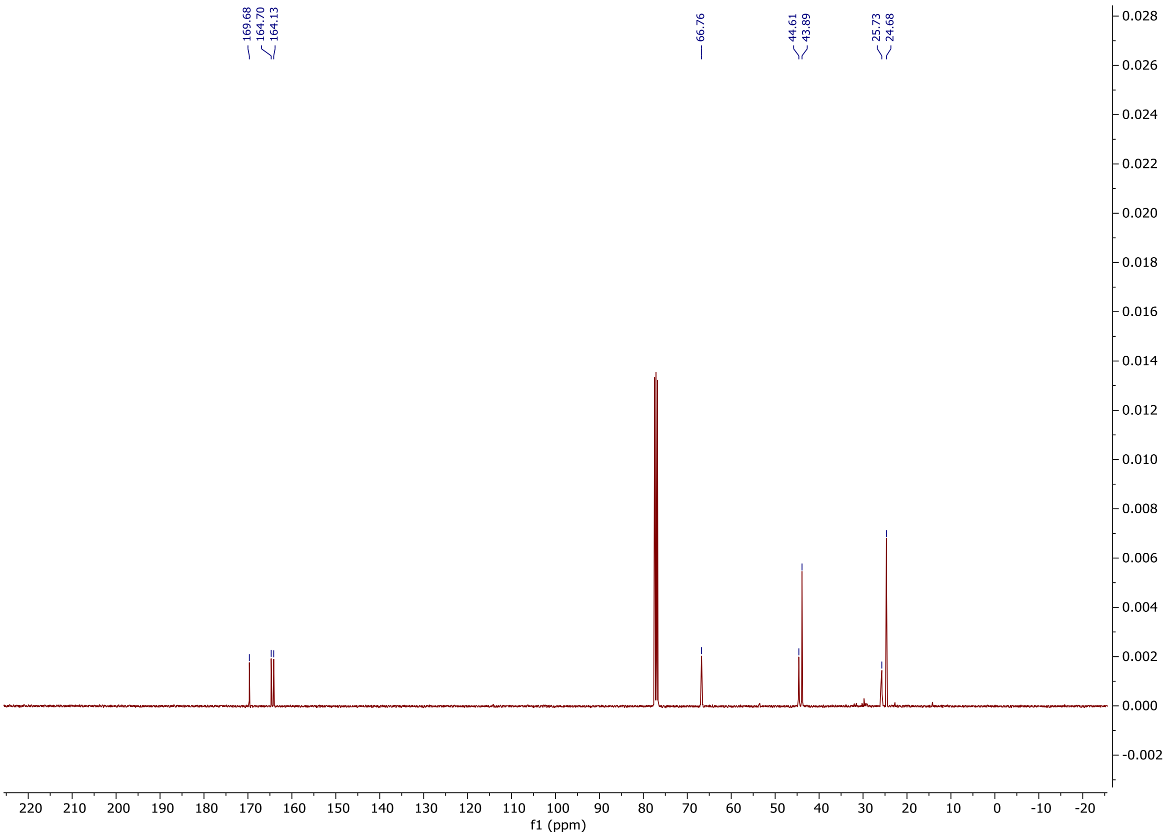

Fig. S65. ^{13}C NMR 4j ($CDCl_3$, 176 MHz).

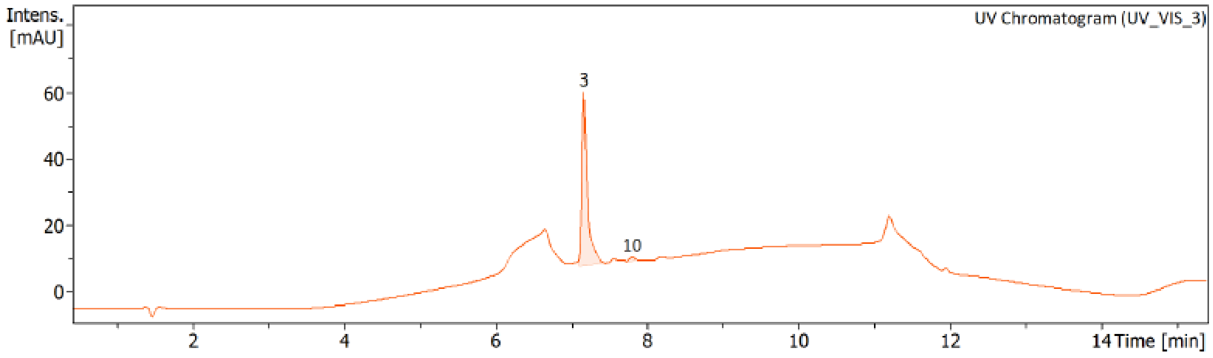

Fig. S66. UV-Vis Chromatogram 4j

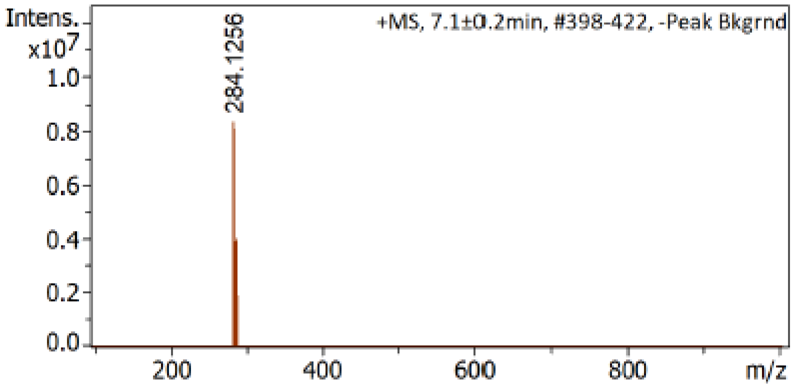

Fig. S67. Mass spectrum 4j

S26. Analytical spectra of 5a

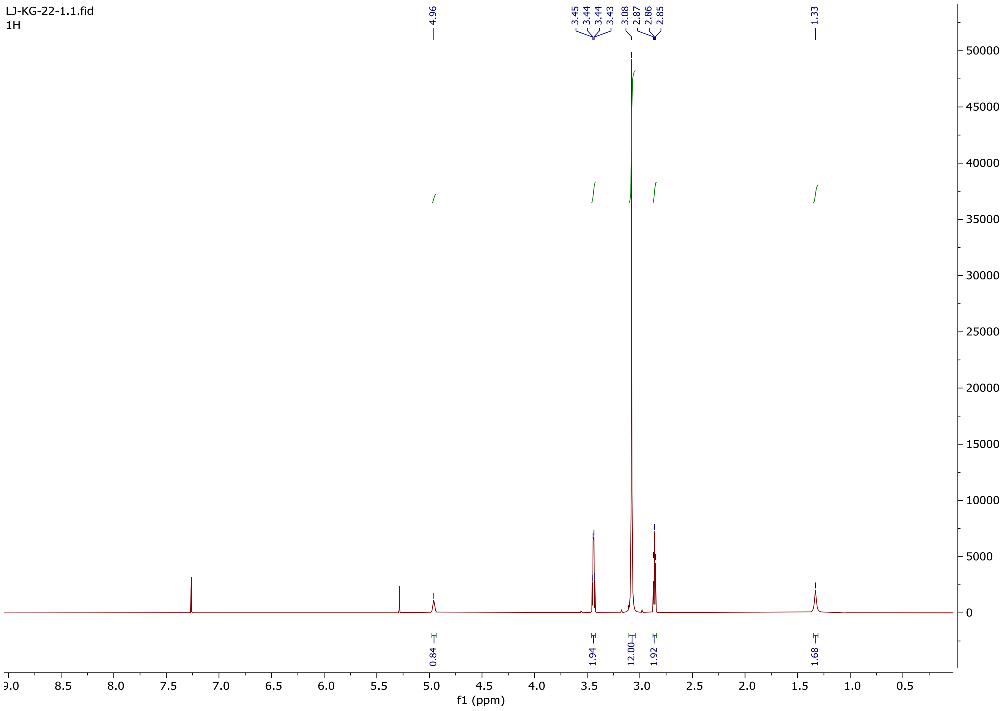

Fig. S68. ^{1}H NMR 5a ($CDCl_3$, 700 MHz).

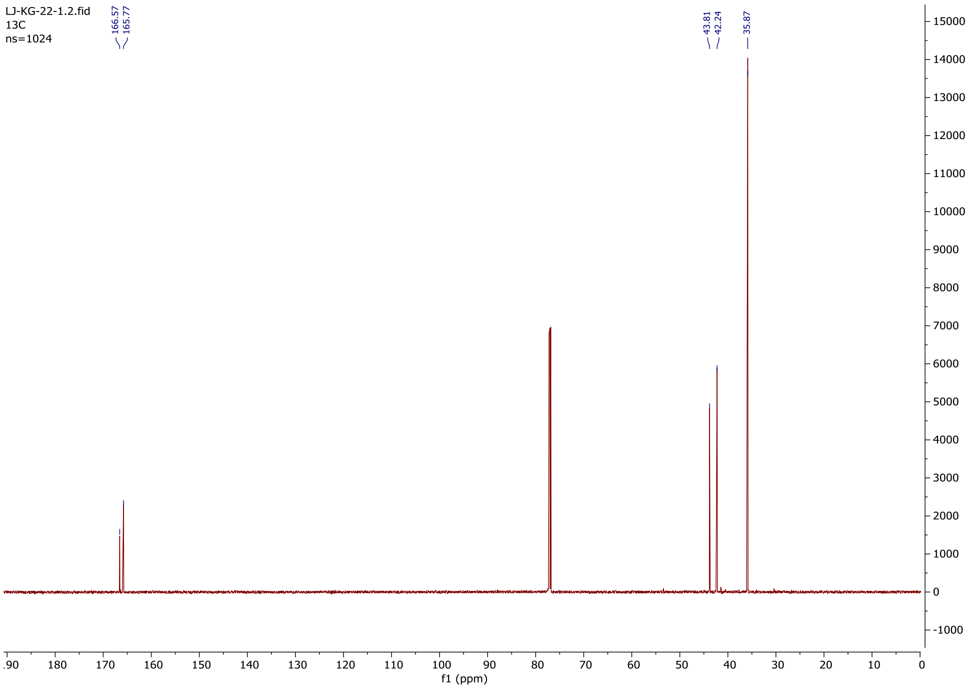

Fig. S69. ^{13}C NMR 5a ($CDCl_3$, 176 MHz).

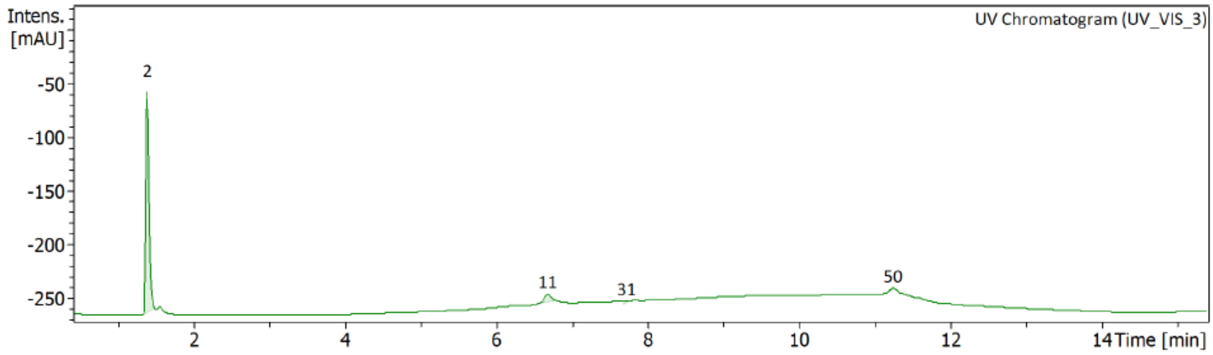

Fig. S70. UV-Vis Chromatogram 5a

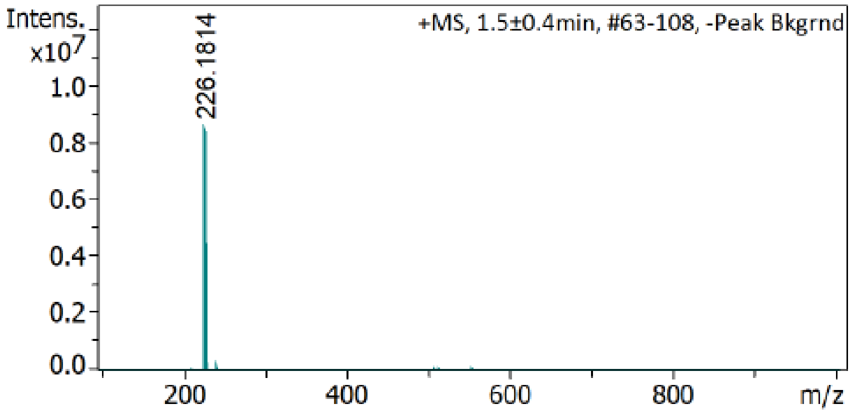

Fig. S71. Mass spectrum 5a

S27. Analytical spectra of 5b

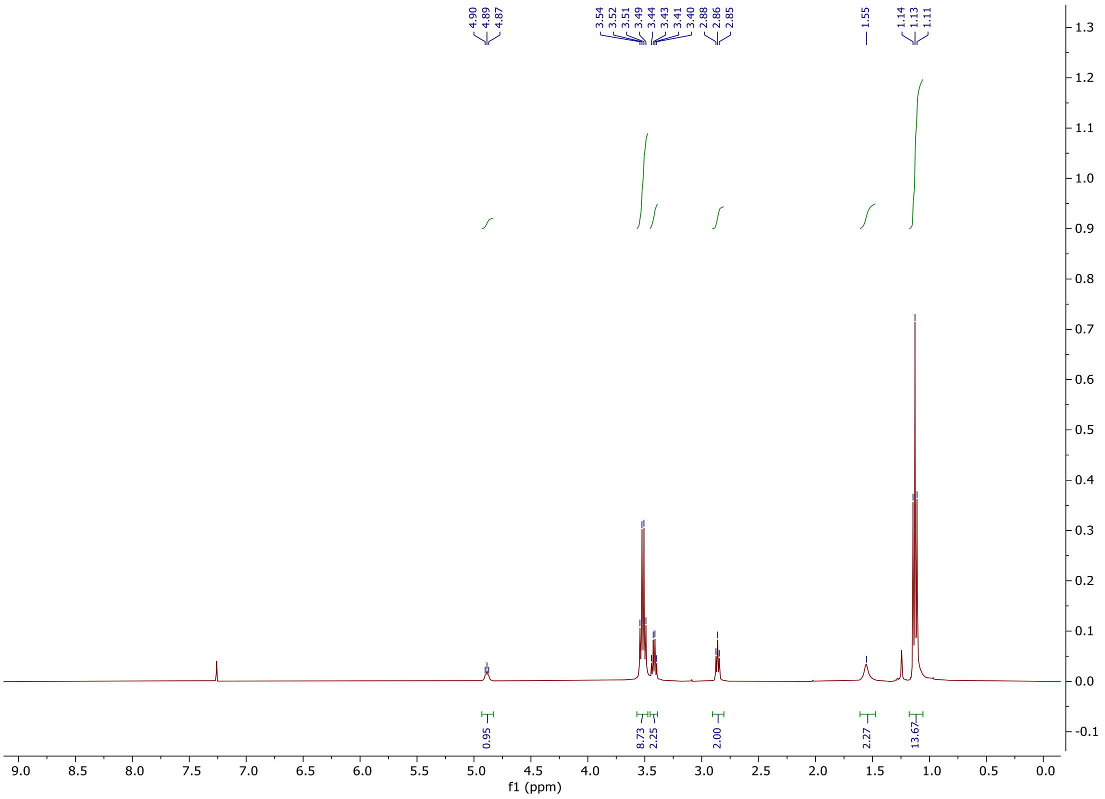

Fig. S72. ^{1}H NMR 5b ($CDCl_3$, 700 MHz).

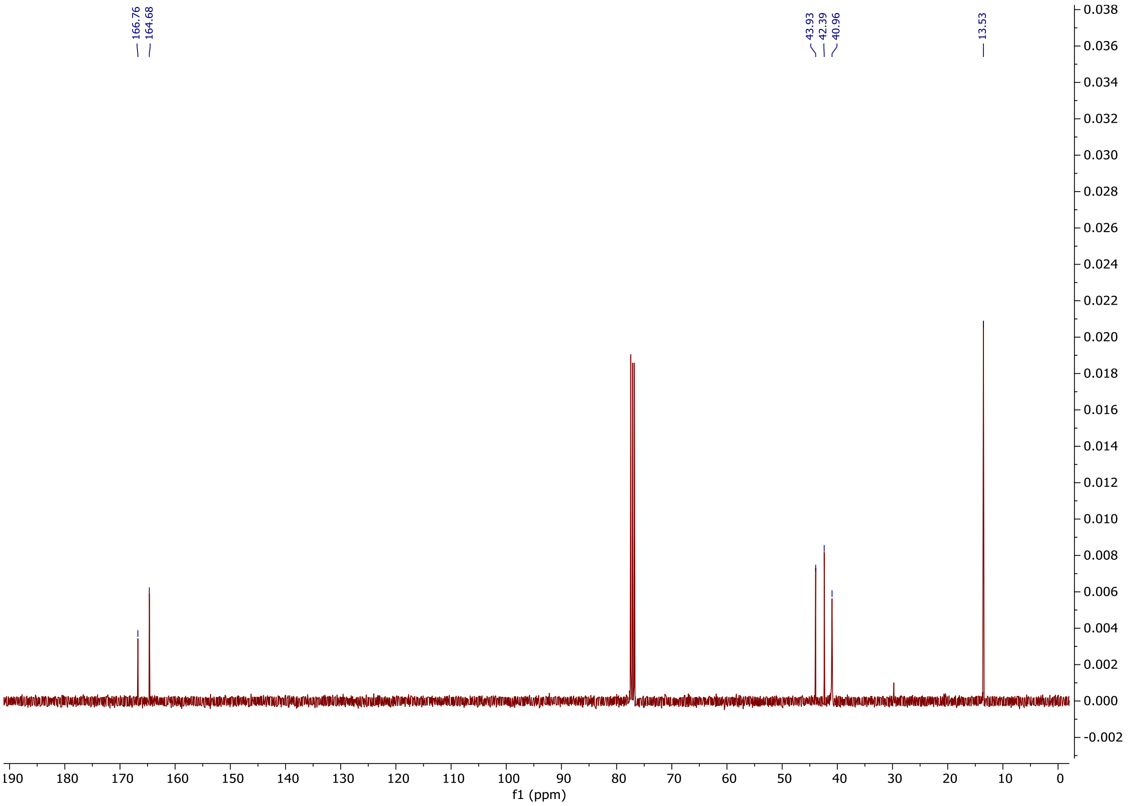

Fig. S73. ^{13}C NMR 5b ($CDCl_3$, 176 MHz).

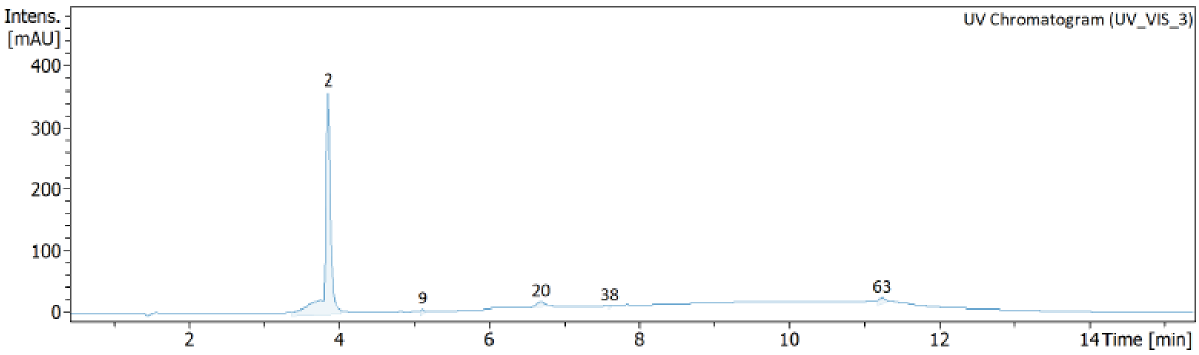

Fig. S74. UV-Vis Chromatogram 5b

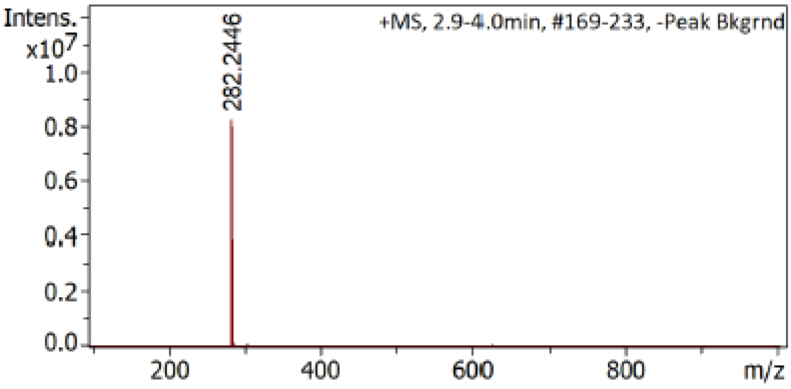

Fig. S75. Mass spectrum 5b

S28. Analytical spectra of 5c

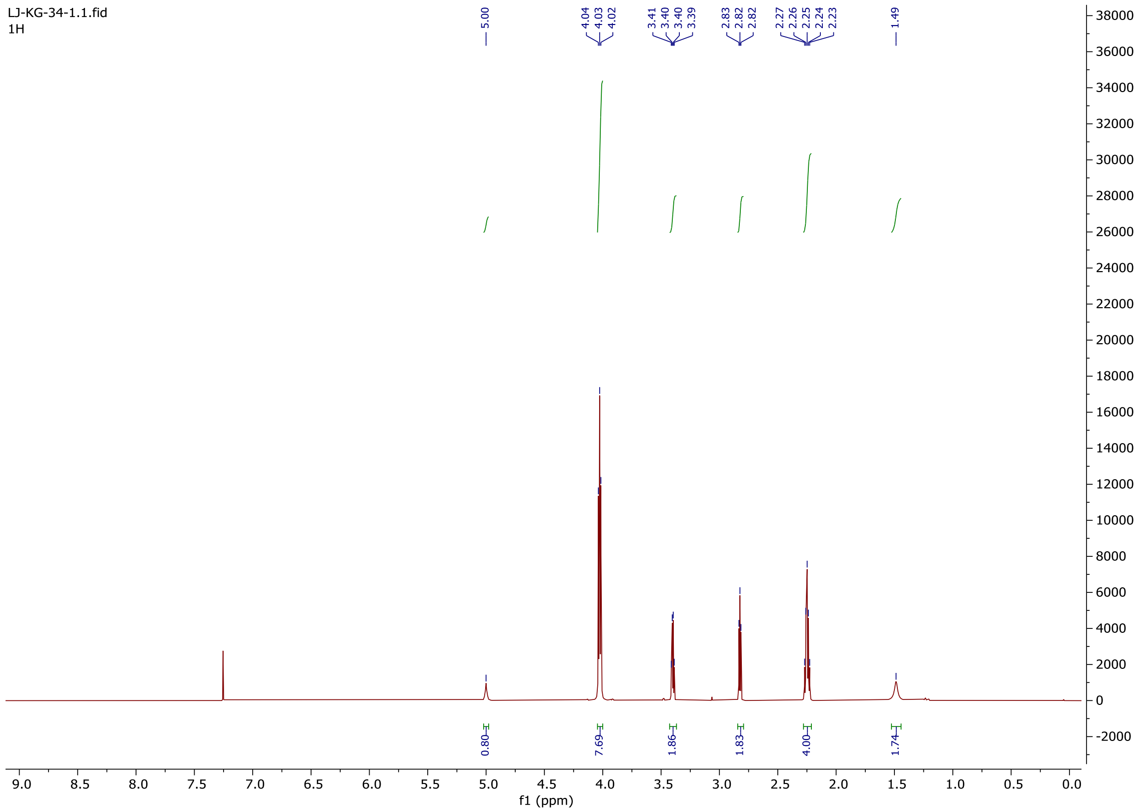

Fig. S76. ^{1}H NMR 5c ($CDCl_3$, 700 MHz).

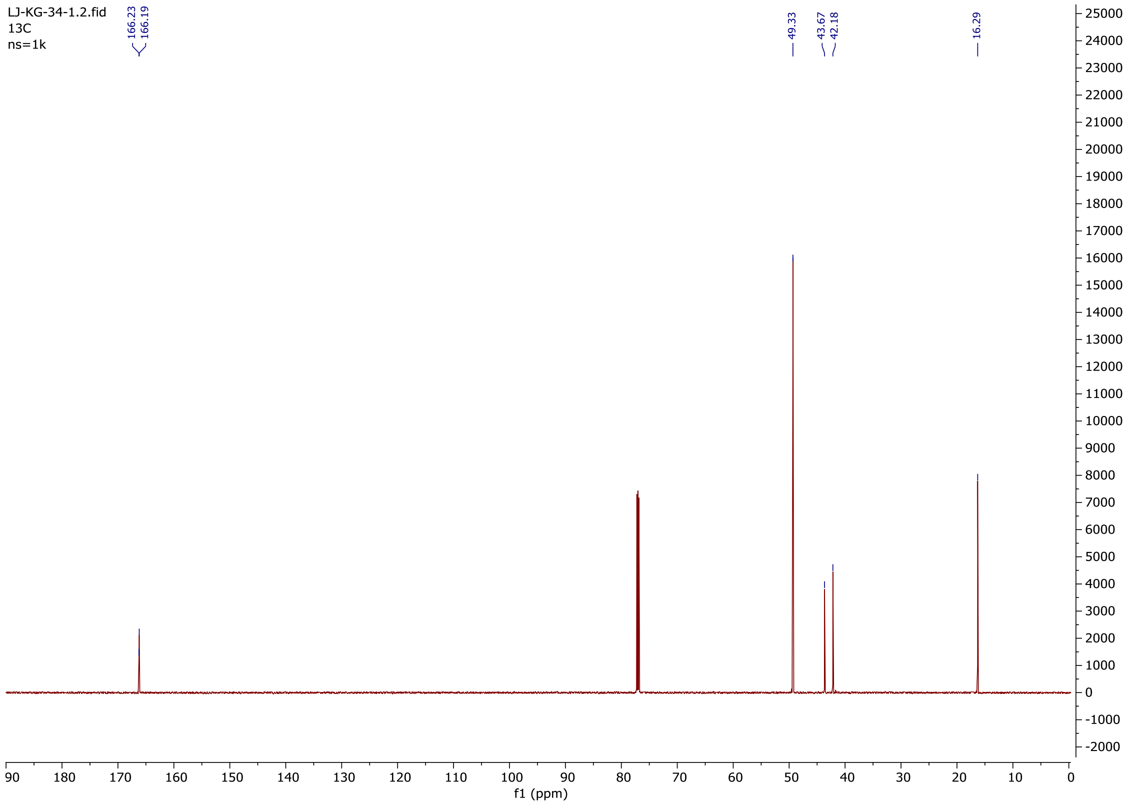

Fig. S77. ^{13}C NMR 5c ($CDCl_3$, 176 MHz).

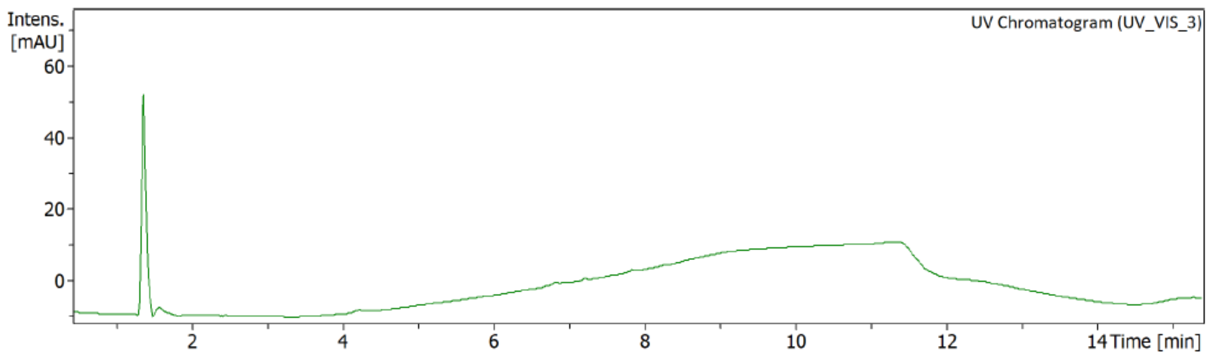

Fig. S78. UV-Vis Chromatograph 5c

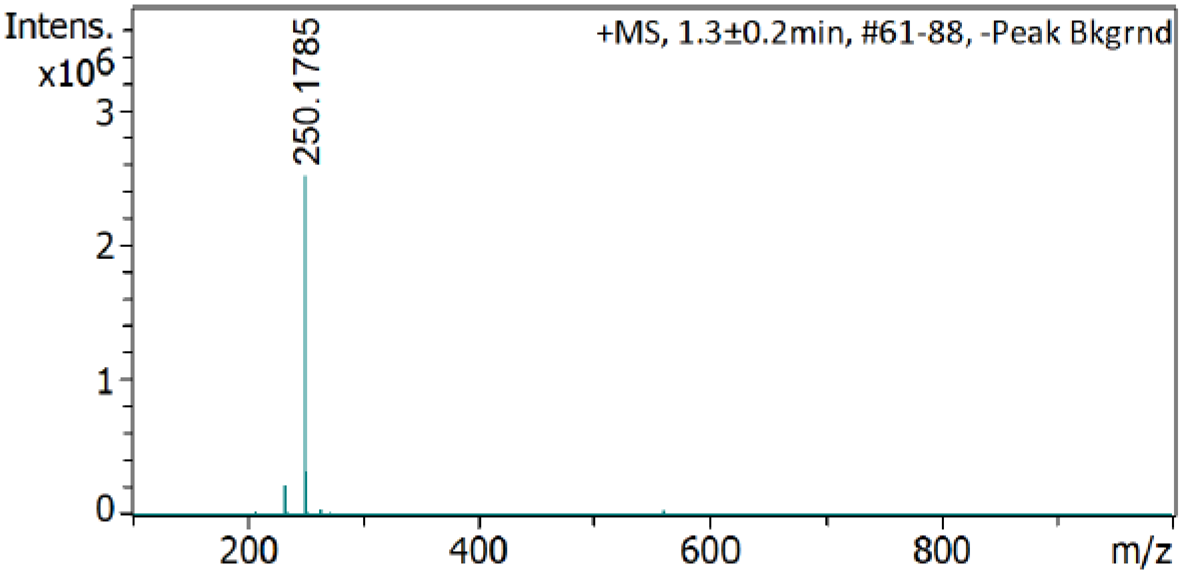

Fig. S79. Mass spectrum 5c

S29. Analytical spectra of 5d

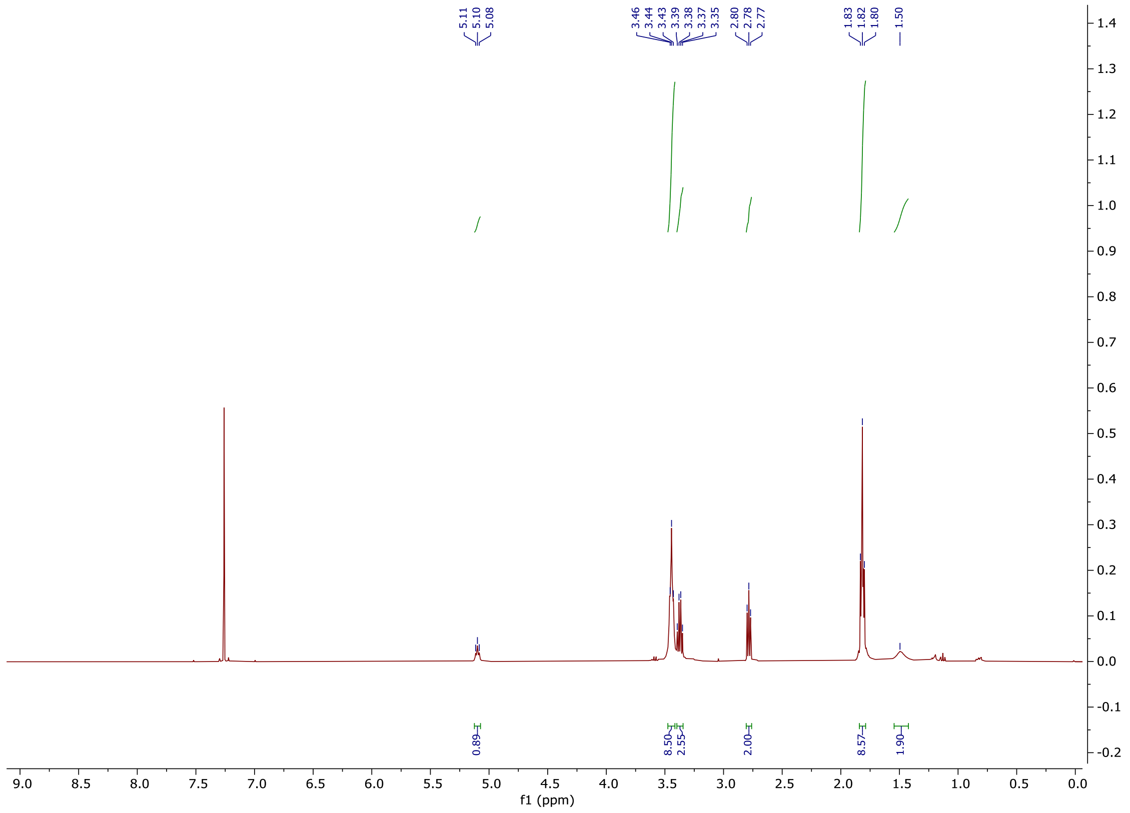

Fig. S80. ^{1}H NMR 5d ($CDCl_3$, 700 MHz).

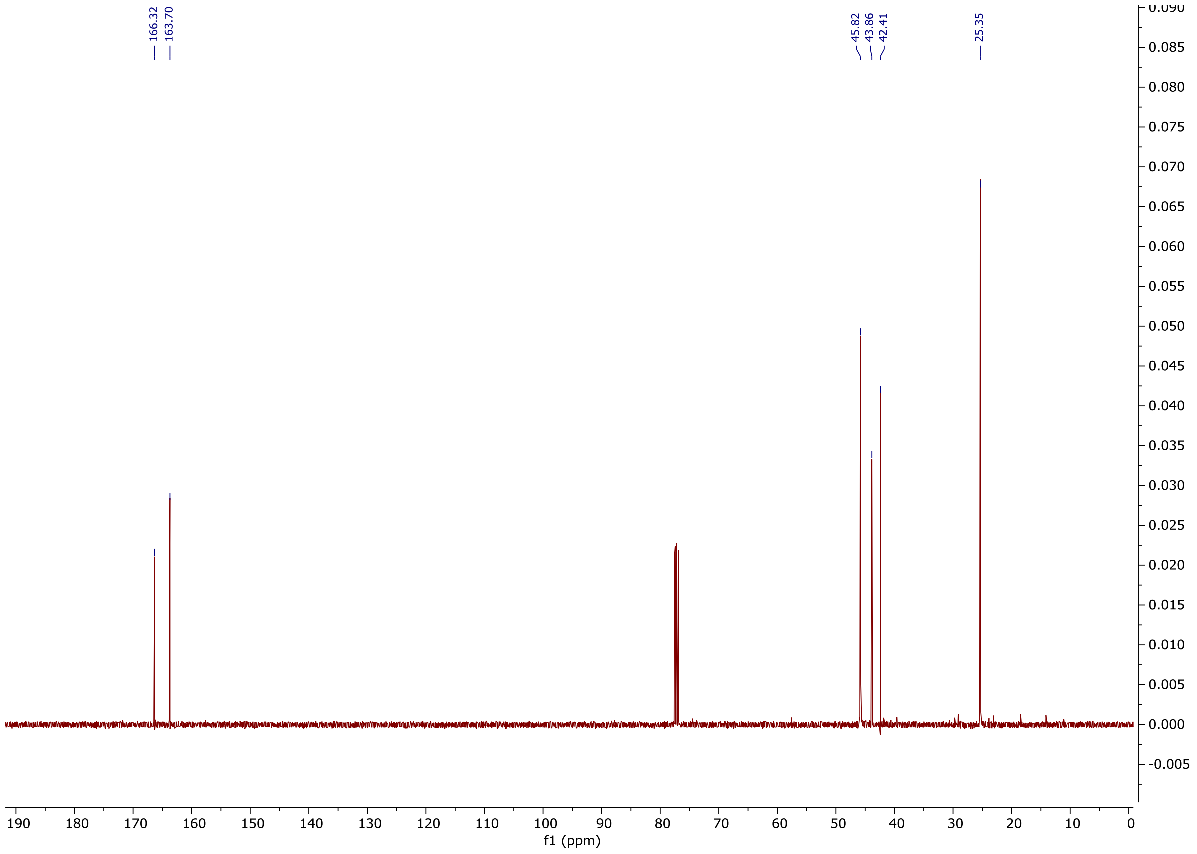

Fig. S81. ^{13}C NMR 5d ($CDCl_3$, 176 MHz).

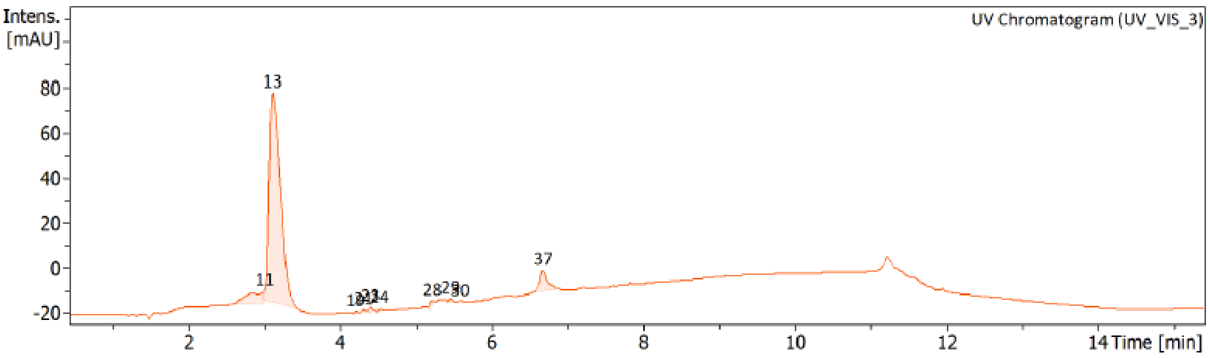

Fig. S82. UV-Vis Chromatogram 5d

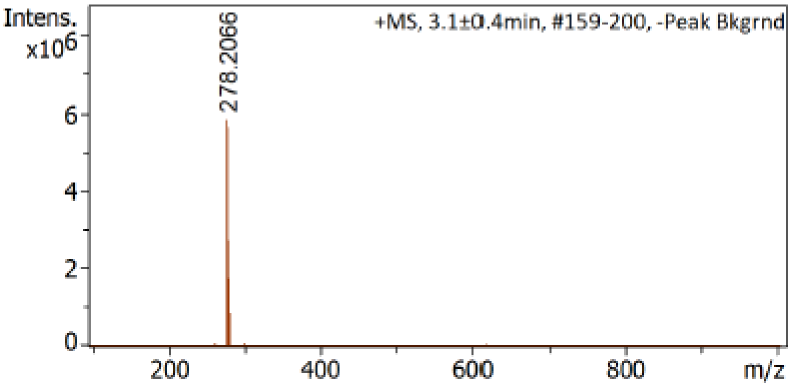

Fig. S83. Mass spectrum 5d

S30. Analytical spectra of 5e

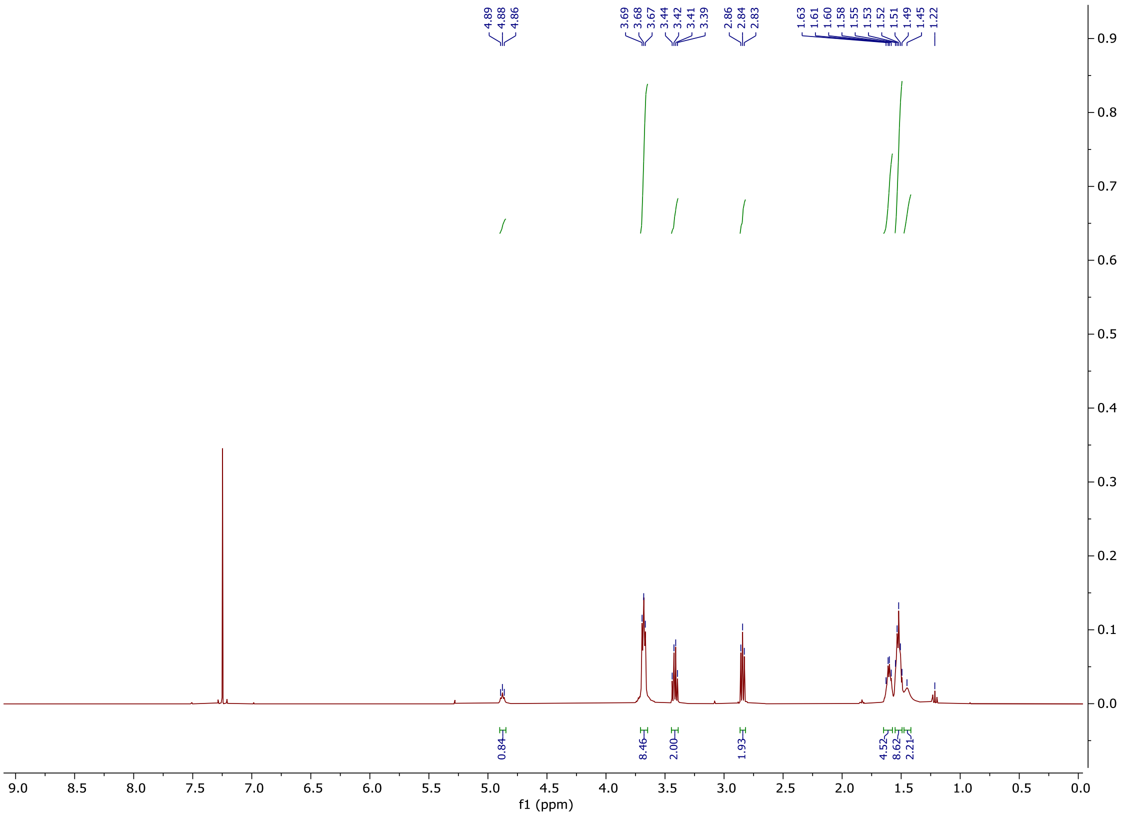

Fig. S84. ^{1}H NMR 5e ($CDCl_3$, 700 MHz).

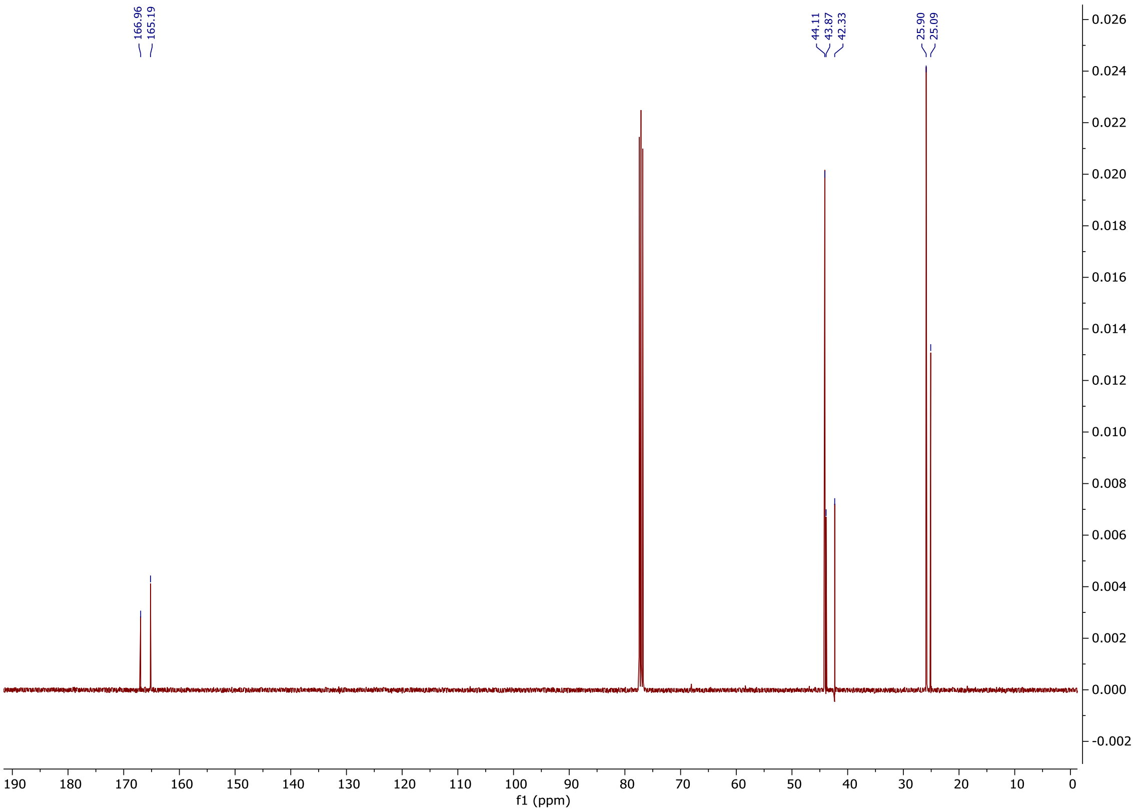

Fig. S85. ^{13}C NMR 5e ($CDCl_3$, 176 MHz).

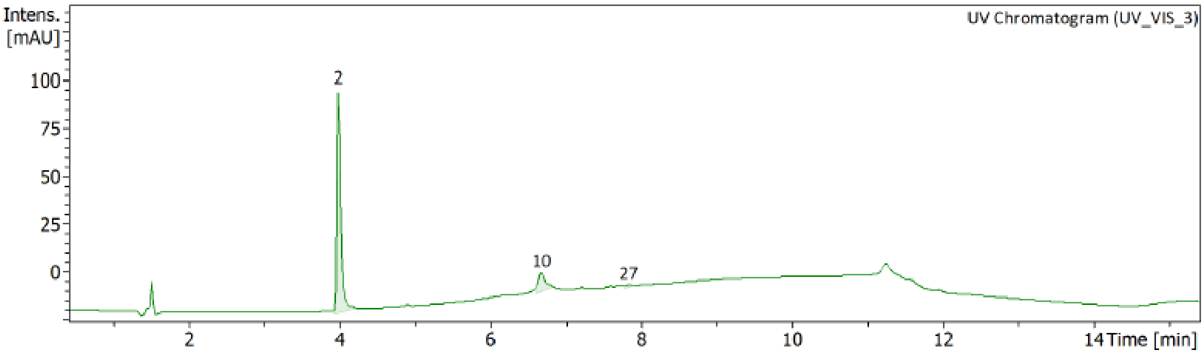

Fig. S86. UV-Vis Chromatogram 5e

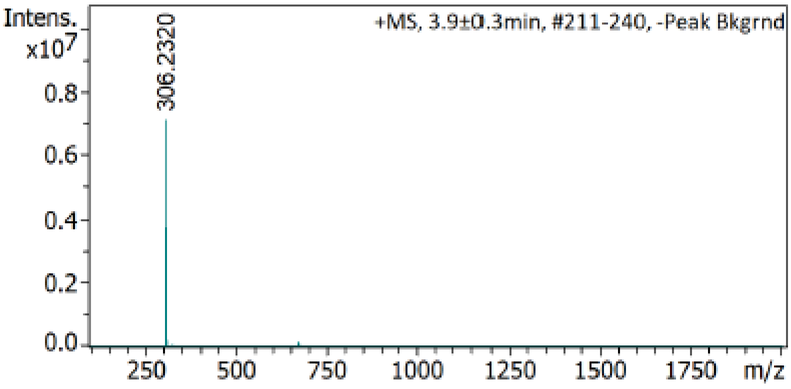

Fig. S87. Mass spectrum 5e

S31. Analytical spectra of 5f

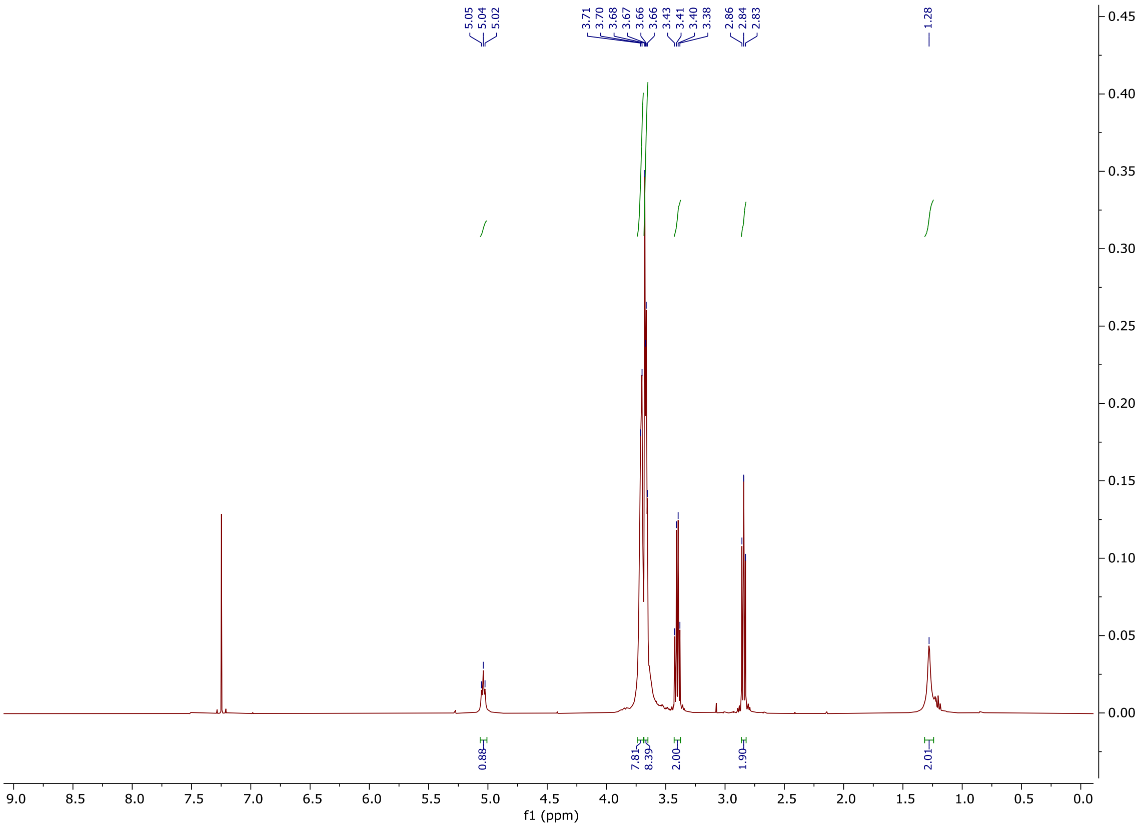

Fig. S88. ^{1}H NMR 5f ($CDCl_3$, 700 MHz).

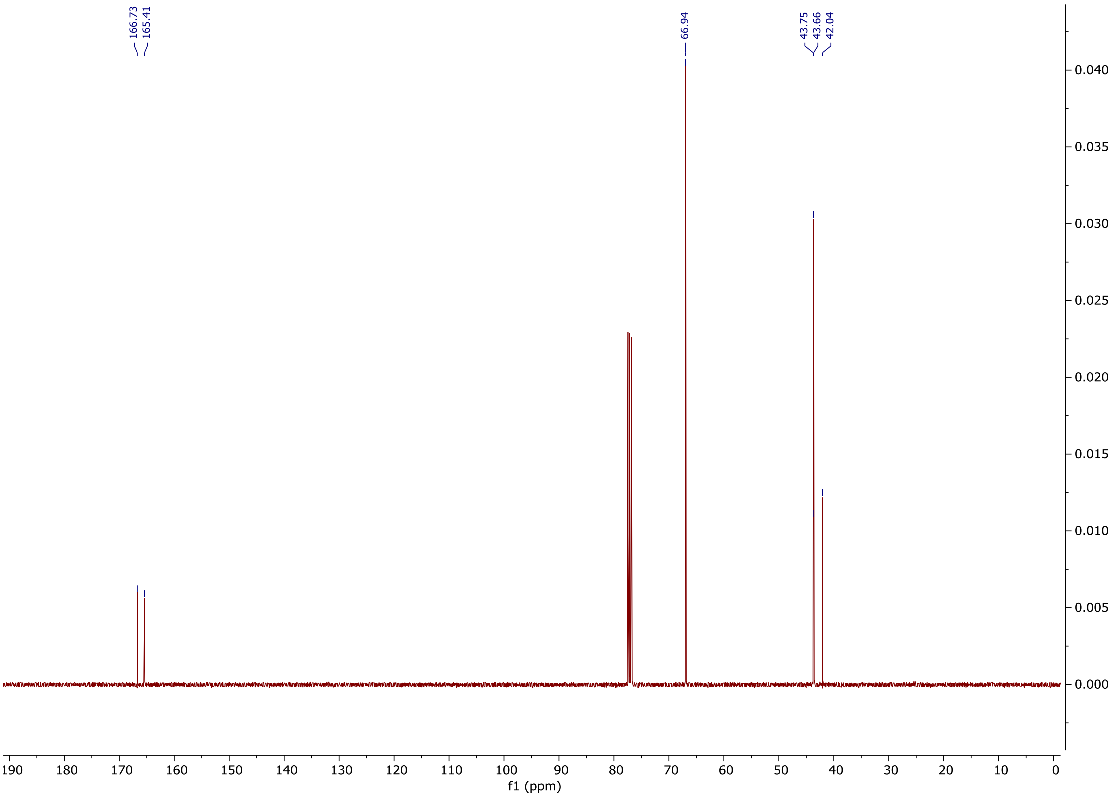

Fig. S89. ^{13}C NMR 5f ($CDCl_3$, 176 MHz).

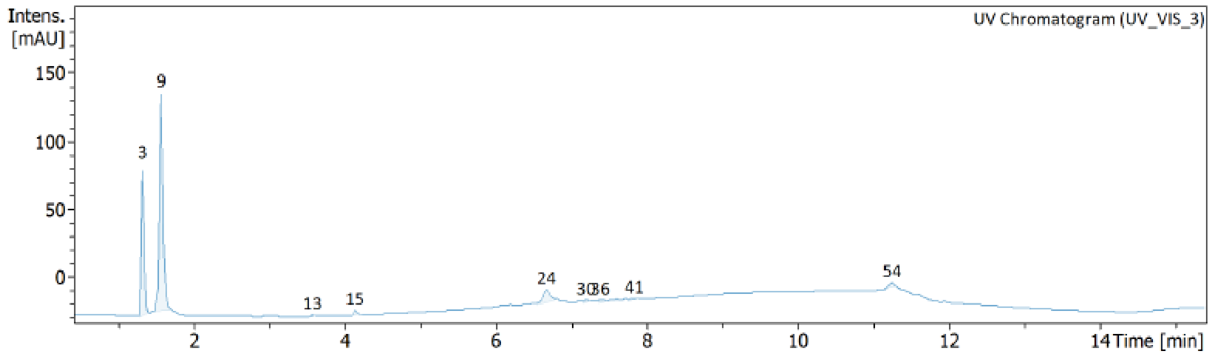

Fig. S90. UV-Vis Chromatogram 5f

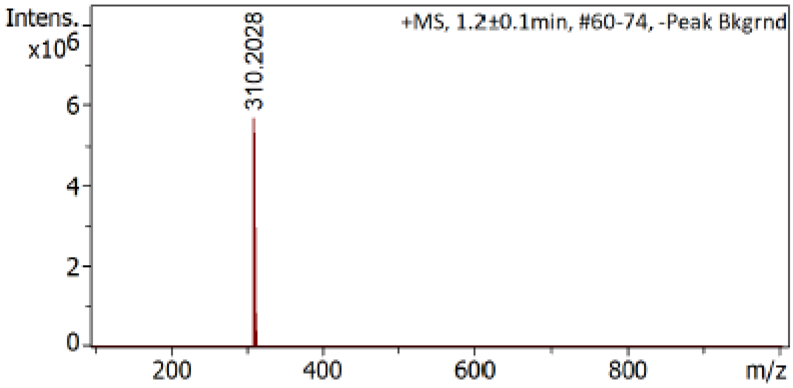

Fig. S91. Mass spectrum 5f

S32. Analytical spectra of 5g

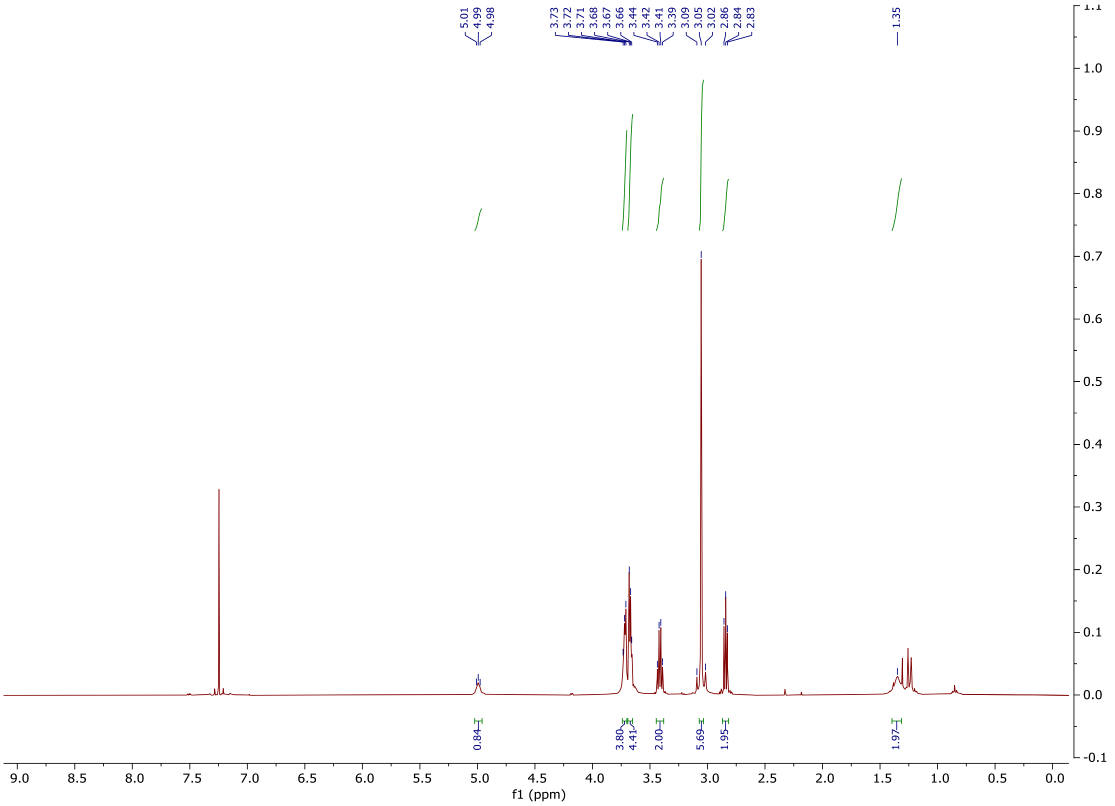

Fig. S92. ^{1}H NMR 5g ($CDCl_3$, 700 MHz).

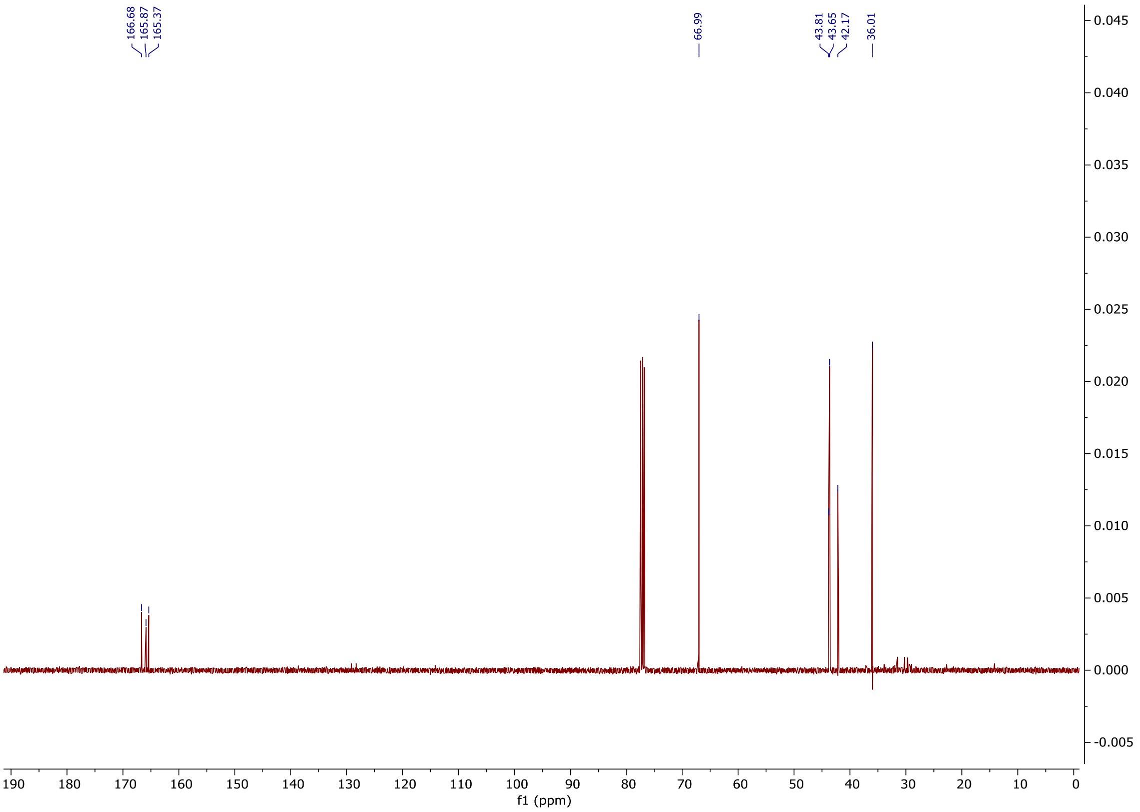

Fig. S93. ^{13}C NMR 5g ($CDCl_3$, 176 MHz).

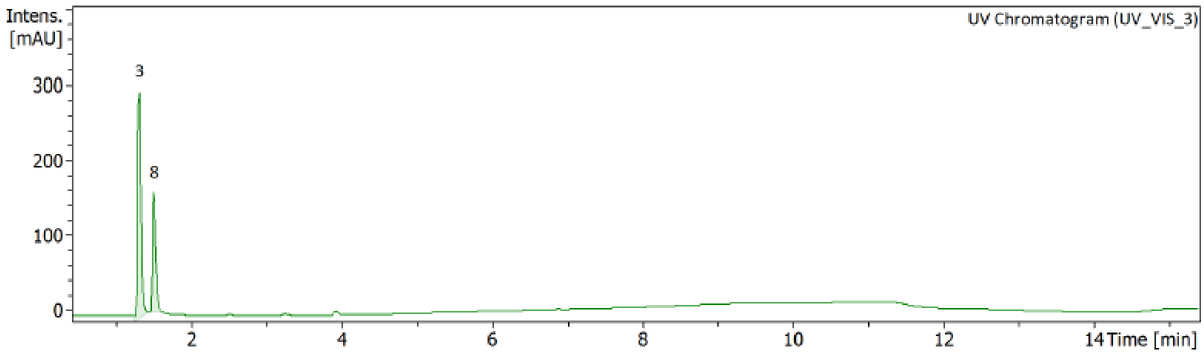

Fig. S94. UV-Vis Chromatogram 5g

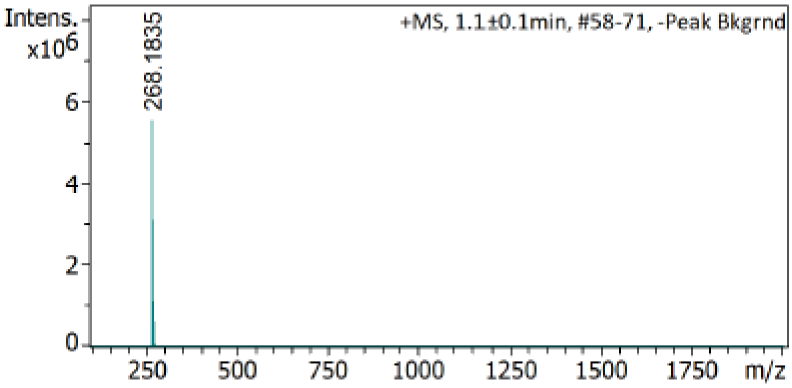

Fig. S95. Mass spectrum 5g

S33. Analytical spectra of 5h

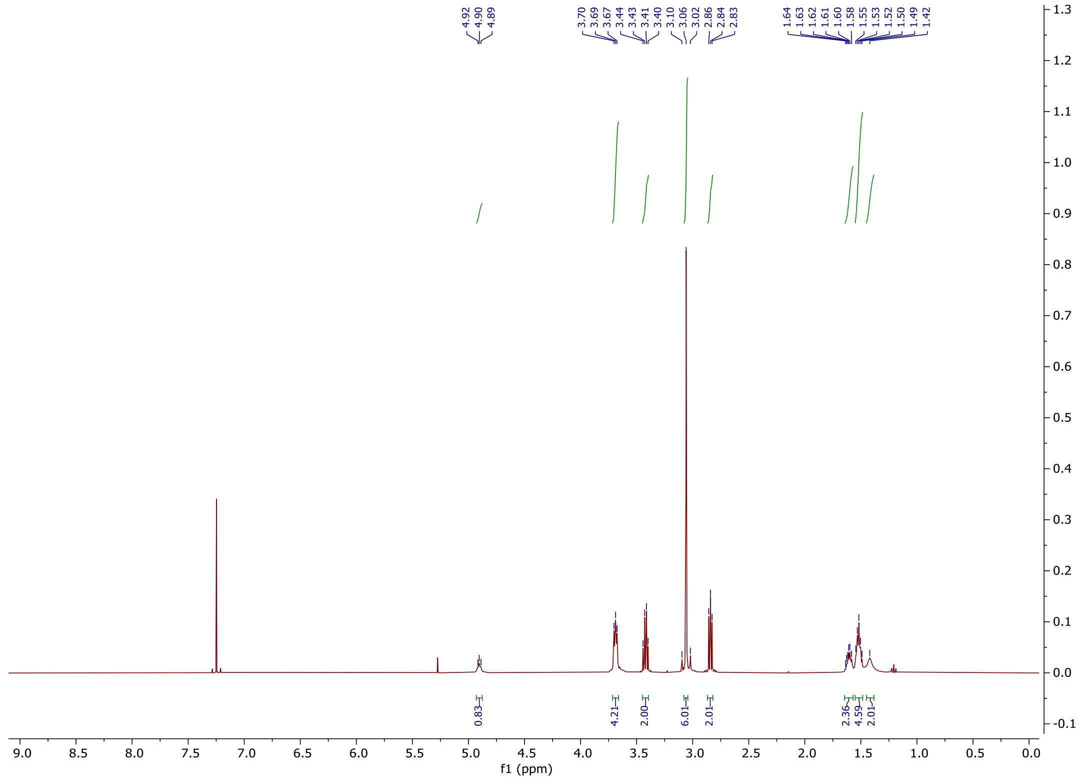

Fig. S96. ¹H NMR 5h ($CDCl_3$, 700 MHz).

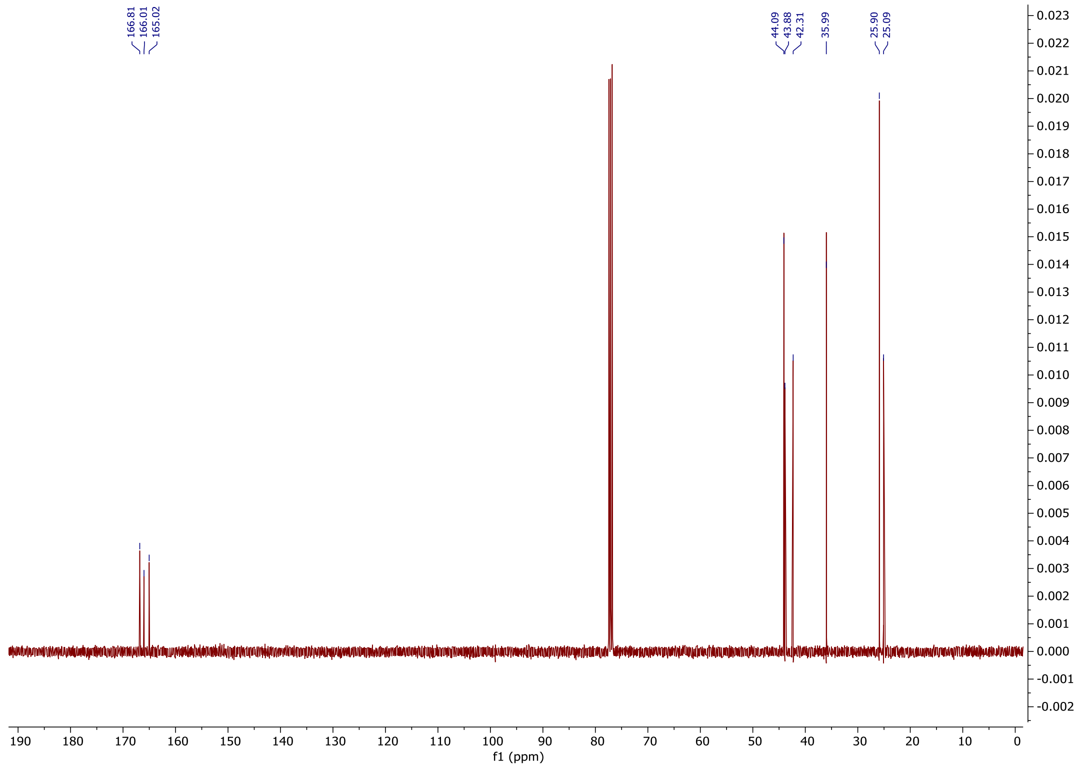

Fig. S97. ¹³C NMR 5h ($CDCl_3$, 176 MHz).

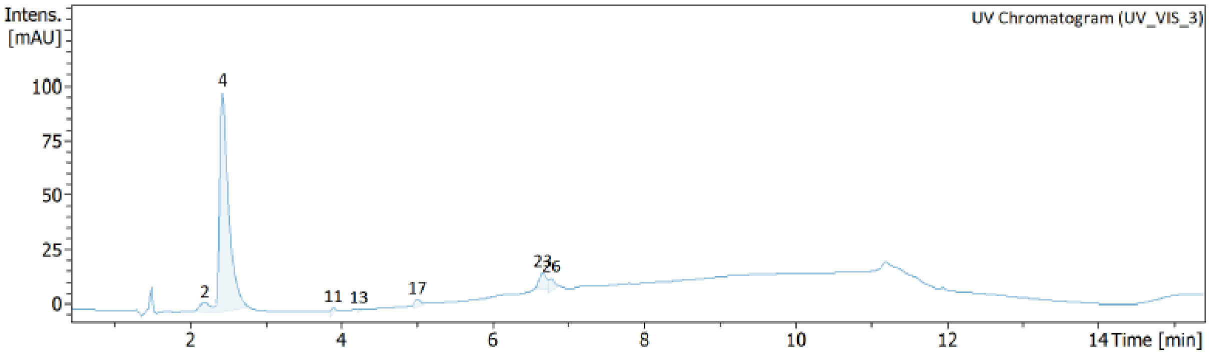

Fig. S98. UV-Vis Chromatogram 5h

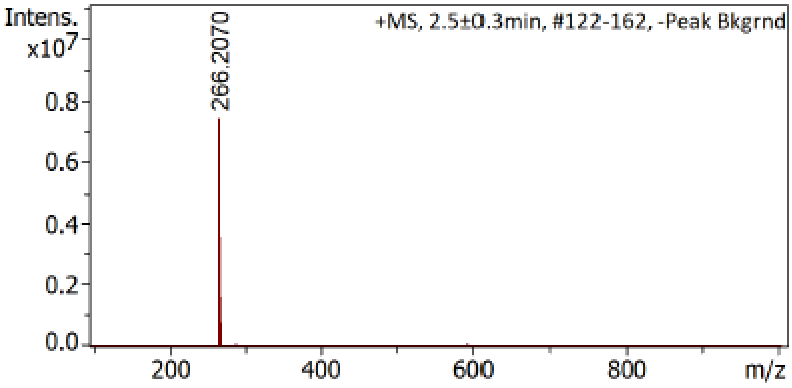

Fig. S99. Mass spectrum 5h

S34. Analytical spectra of 5i

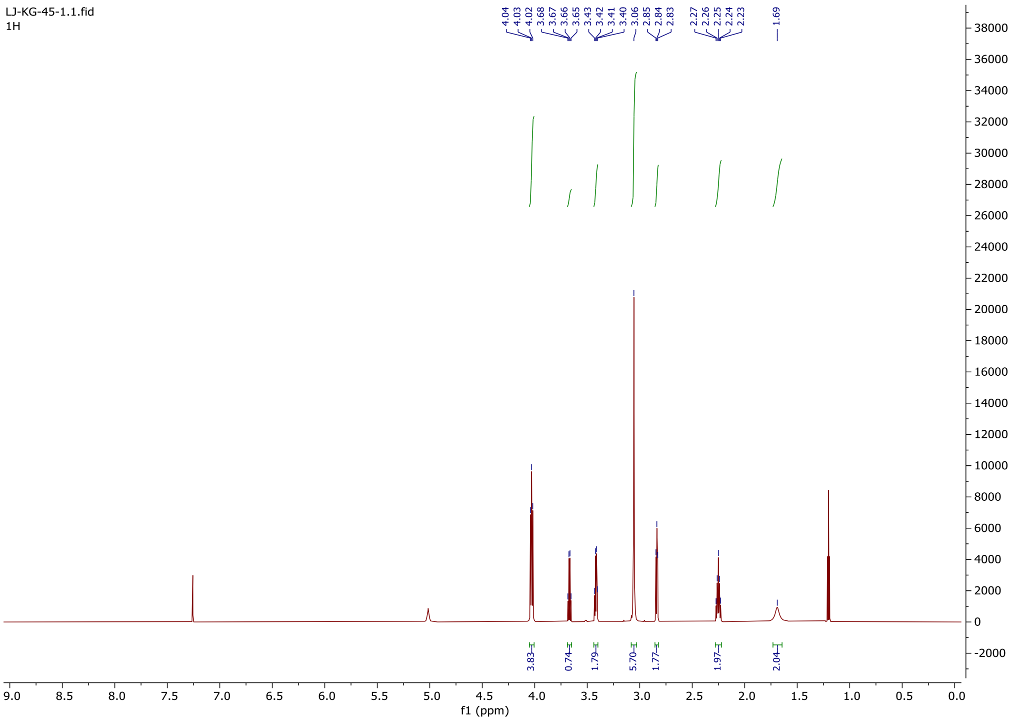

Fig. S100. ^{1}H NMR 5i ($CDCl_3$, 700 MHz).

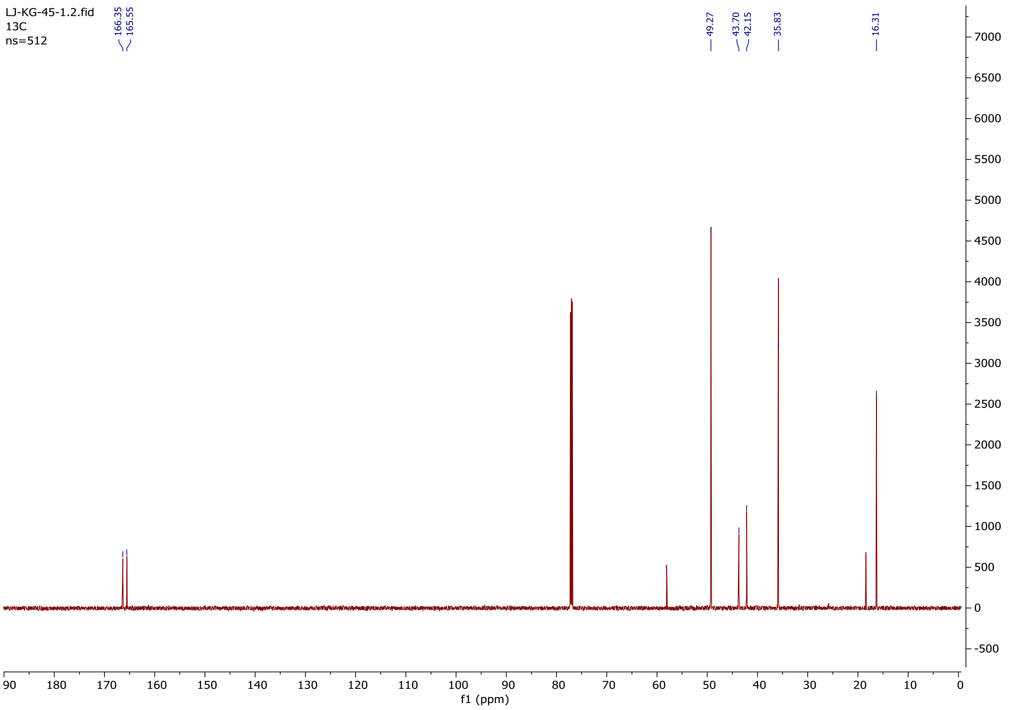

Fig. S101. ^{13}C NMR 5i ($CDCl_3$, 176 MHz).

S35. Analytical spectra of 5j

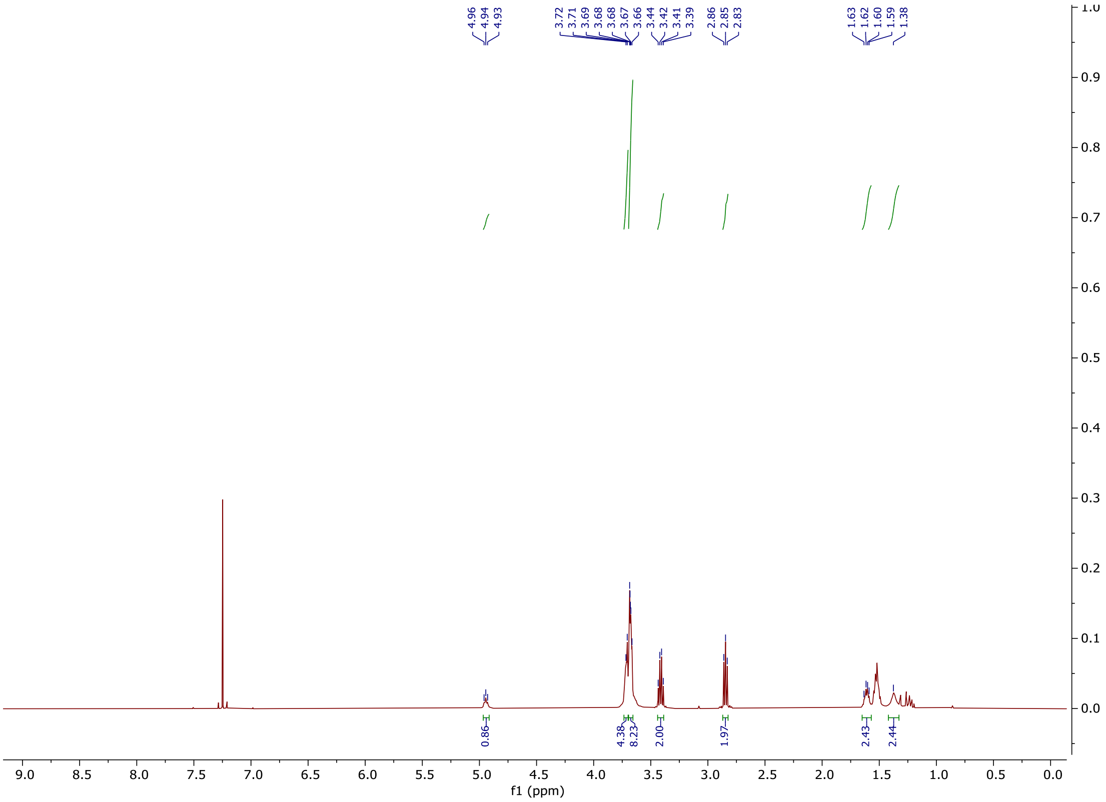

Fig. S102. ^{1}H NMR 5j ($CDCl_3$, 700 MHz).

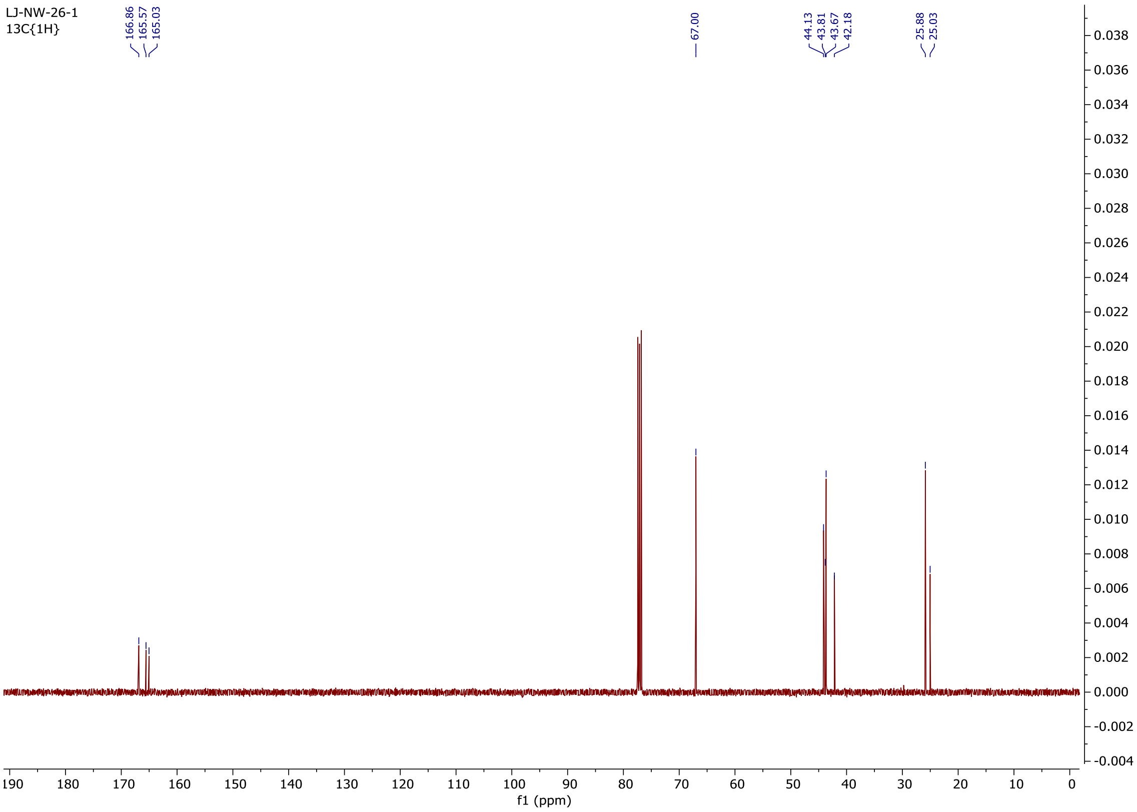

Fig. S103. ^{13}C NMR 5j ($CDCl_3$, 176 MHz).

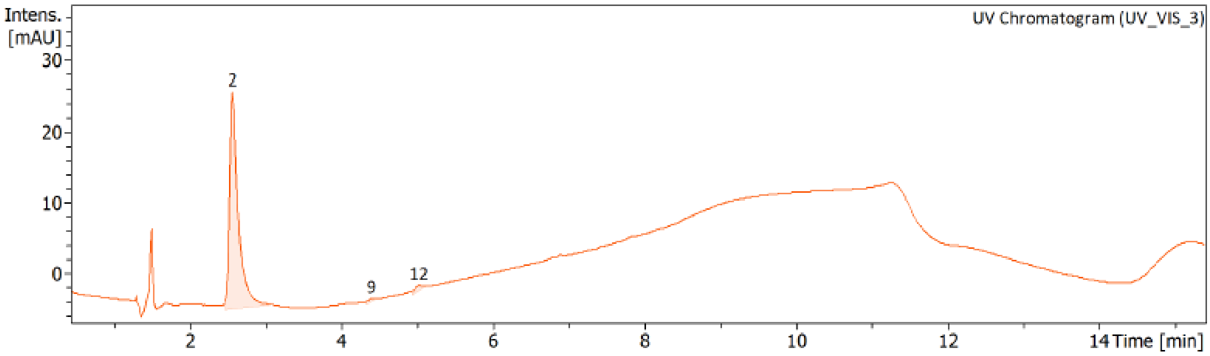

Fig. S104. UV-Vis Chromatogram 5j

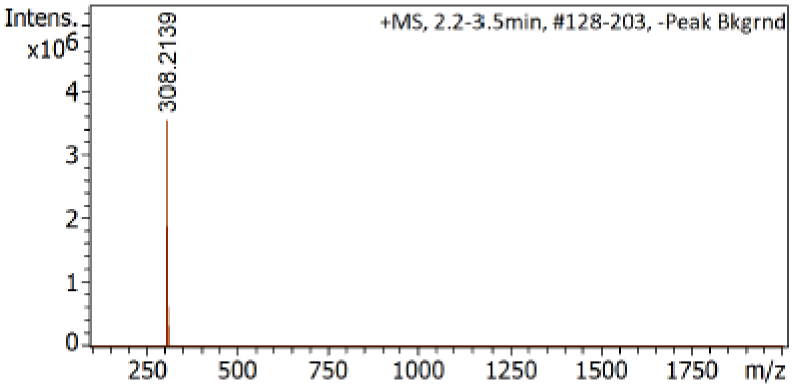

Fig. S105. Mass spectrum 5j

S36. Analytical spectra of 5k

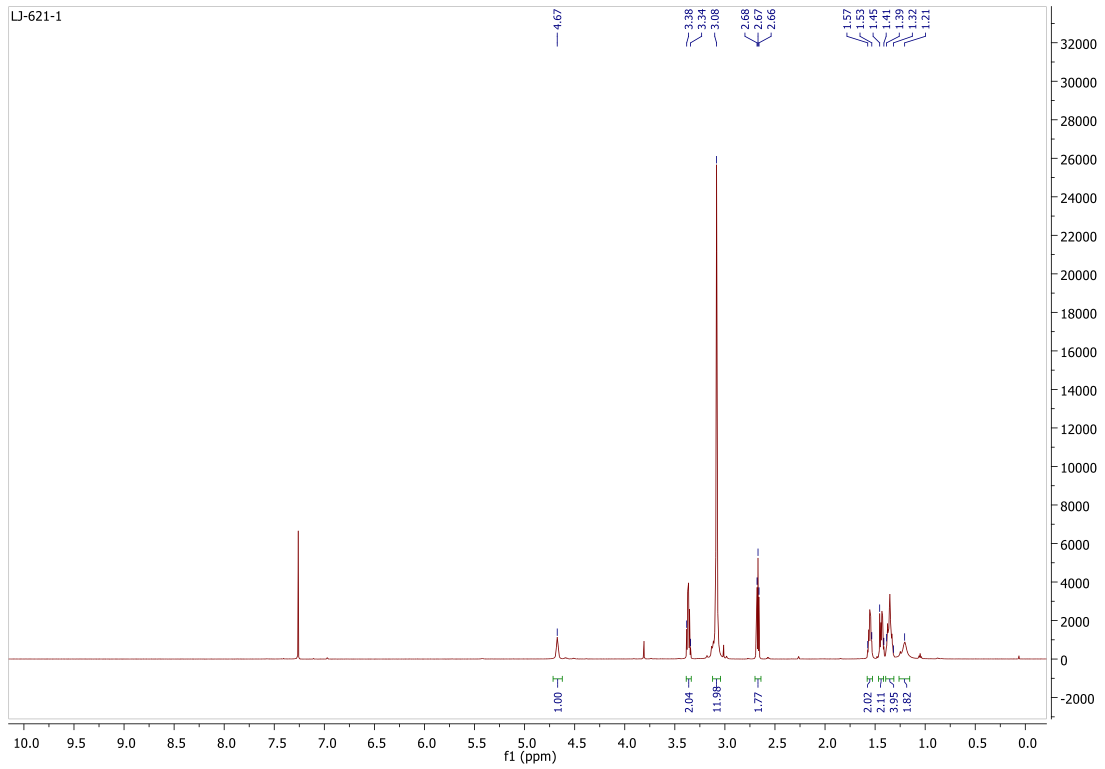

Fig. S106. ^{1}H NMR 5k ($CDCl_3$, 700 MHz).

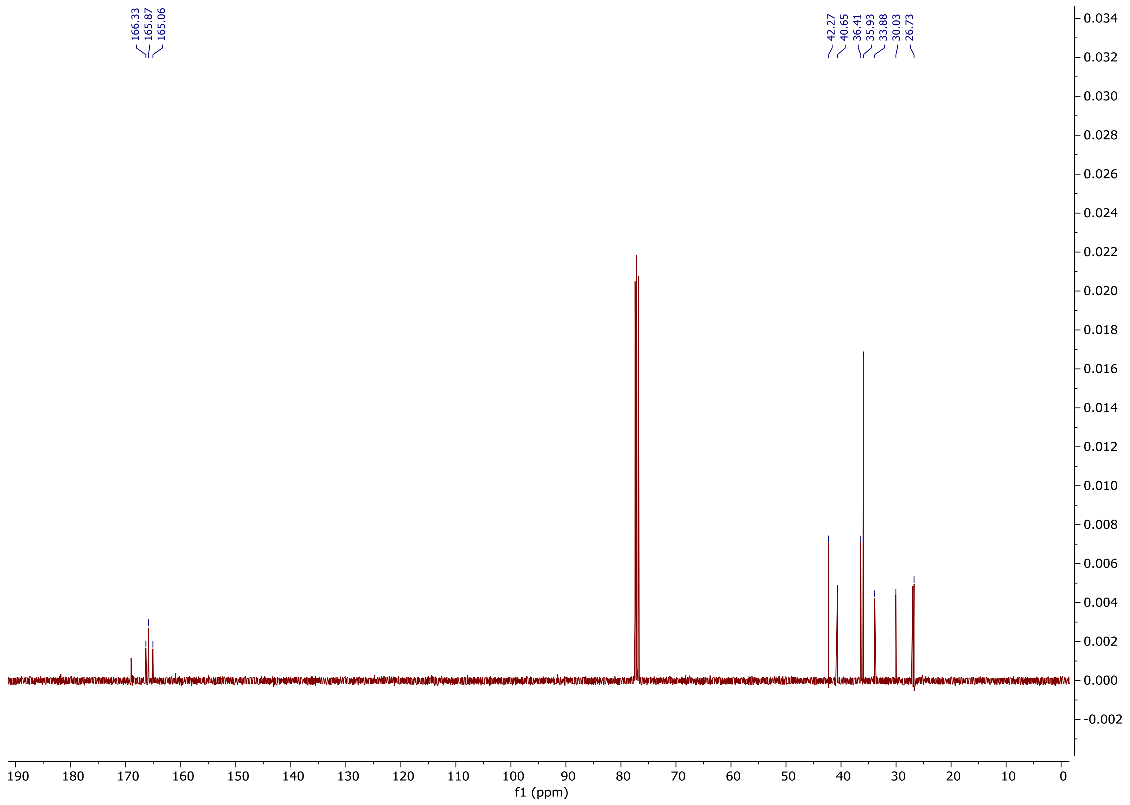

Fig. S107. ^{13}C NMR 5k ($CDCl_3$, 176 MHz).

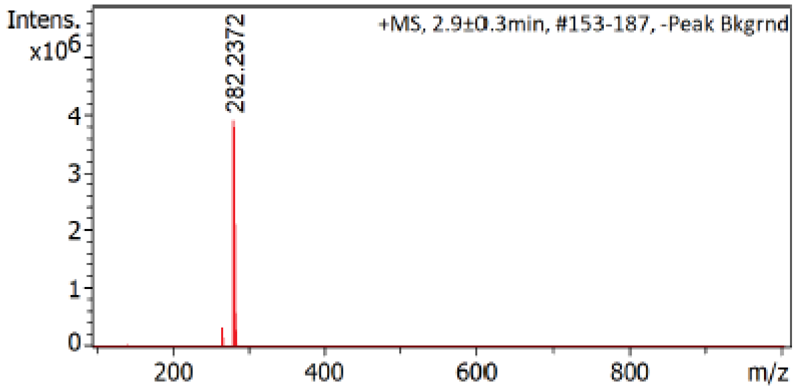

Fig. S108. Mass spectrum 5k

S37. Analytical spectra of 8a

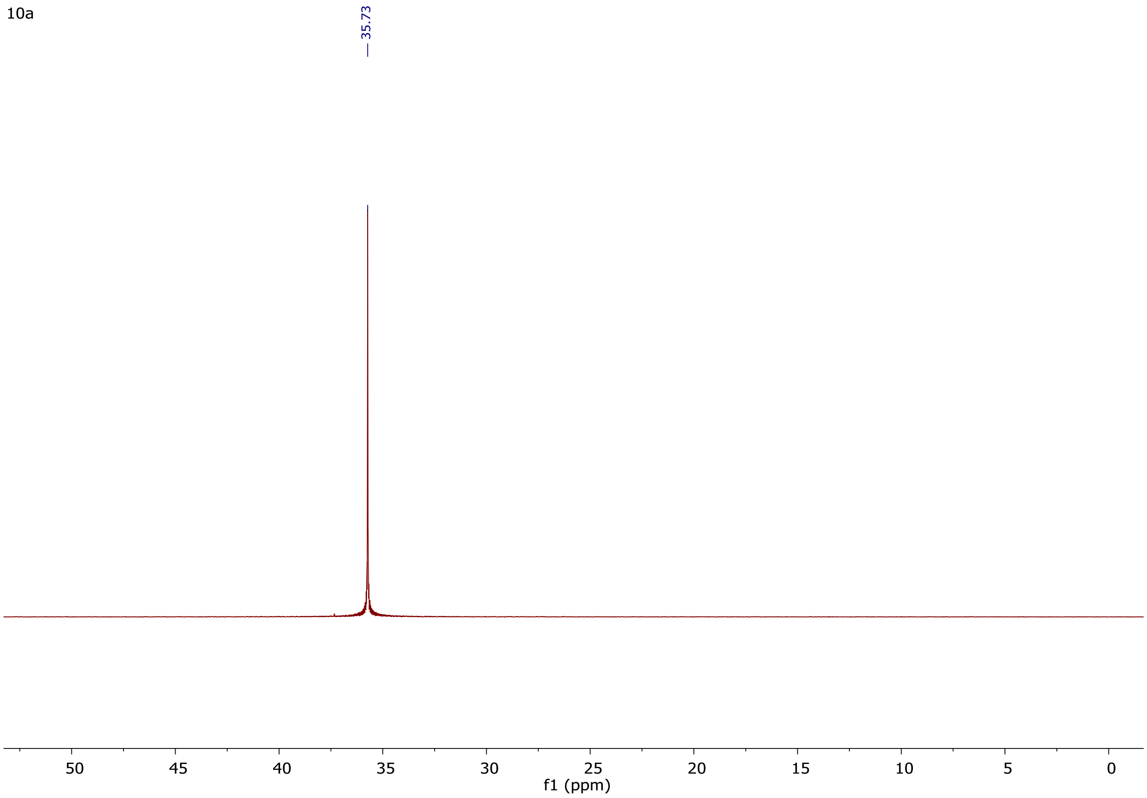

Fig. S109. ^{31}P NMR 8a ($CDCl_3$, 700 MHz).

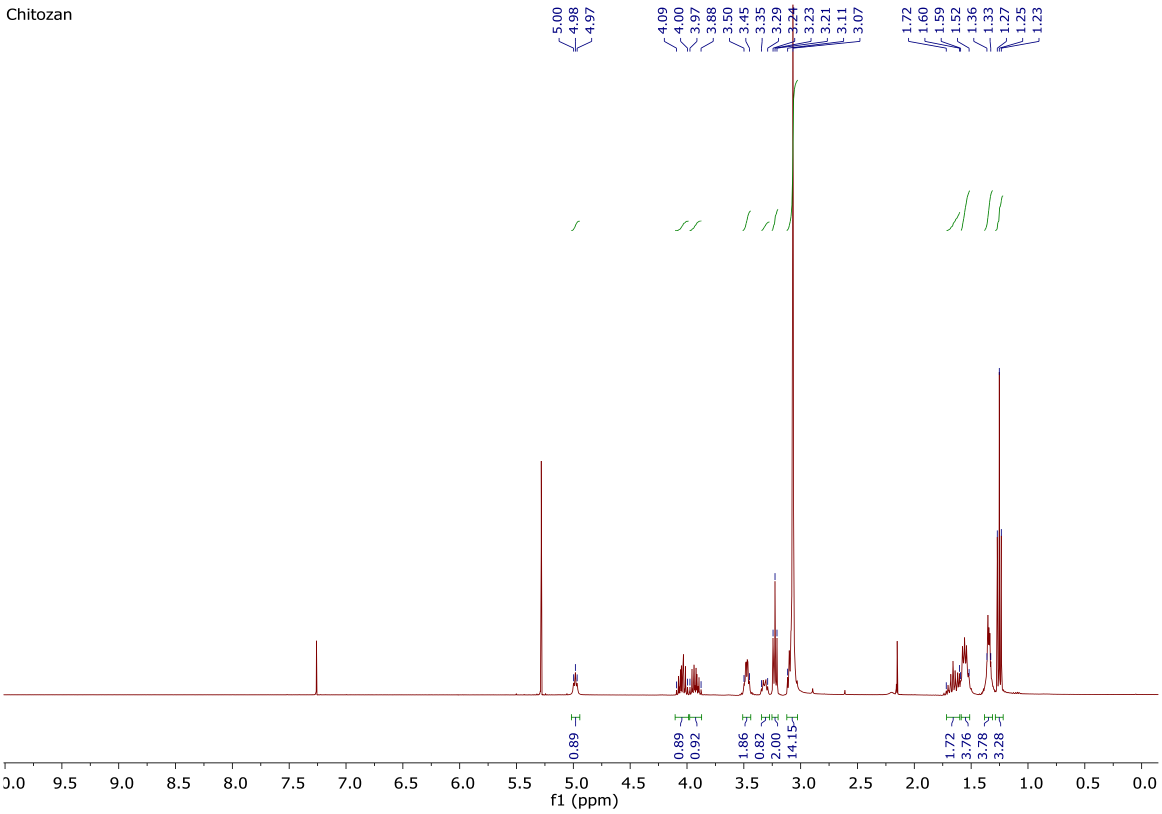

Fig. S110. ^{1}H NMR of 8a ($CDCl_3$, 700 MHz).

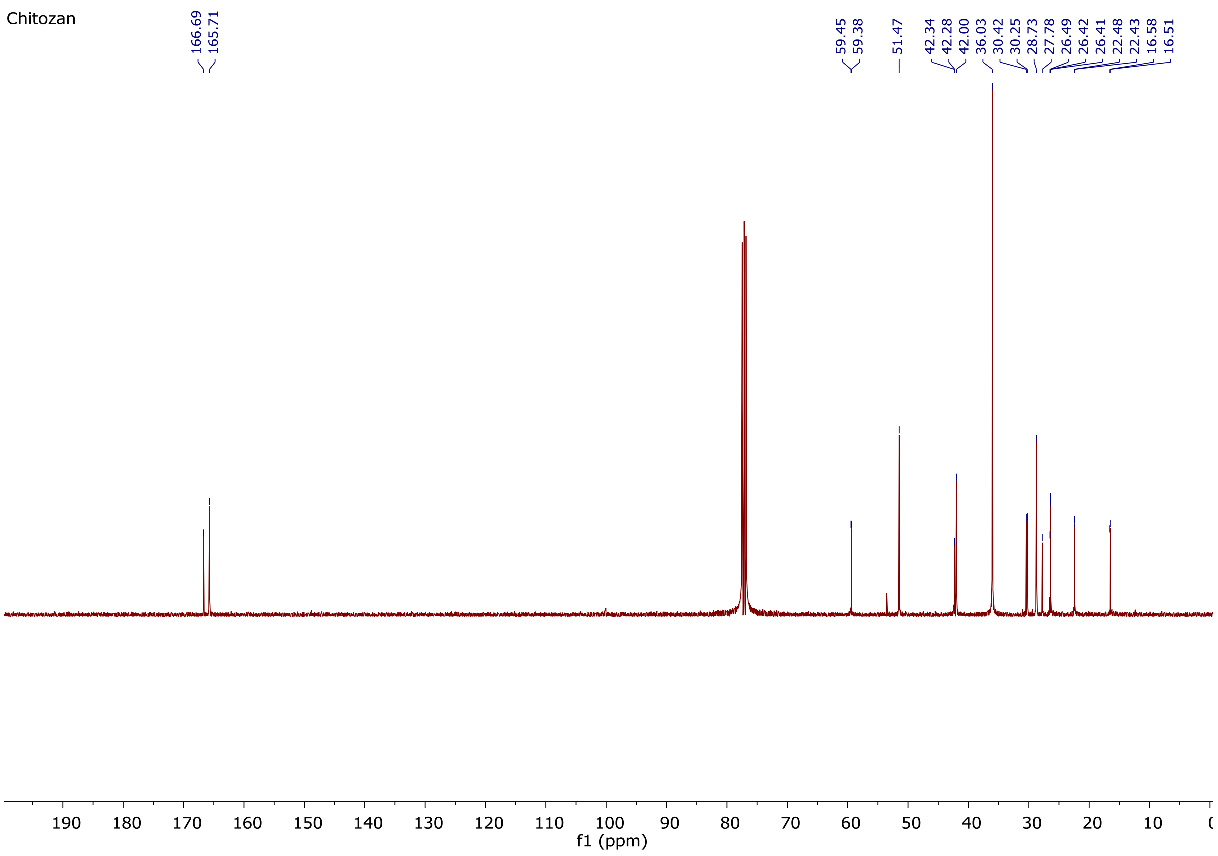

Fig. S111. ^{13}C NMR 8a ($CDCl_3$, 176 MHz).

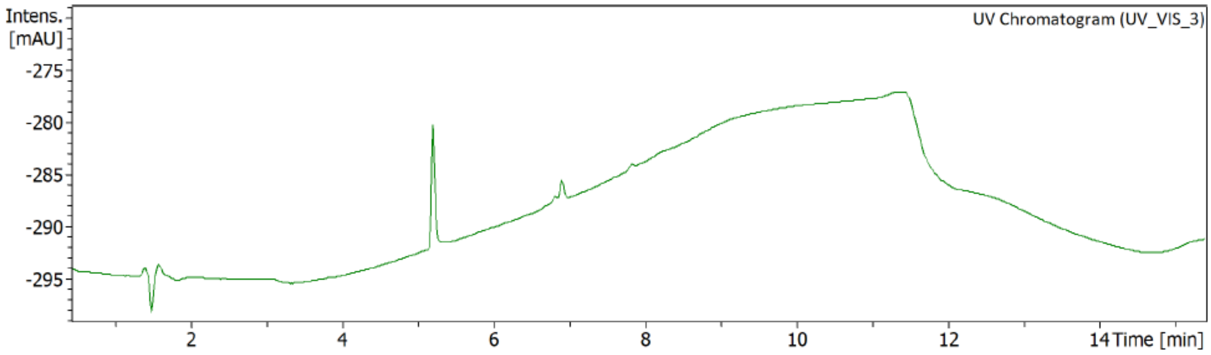

Fig. S112. UV-Vis Chromatogram 8a

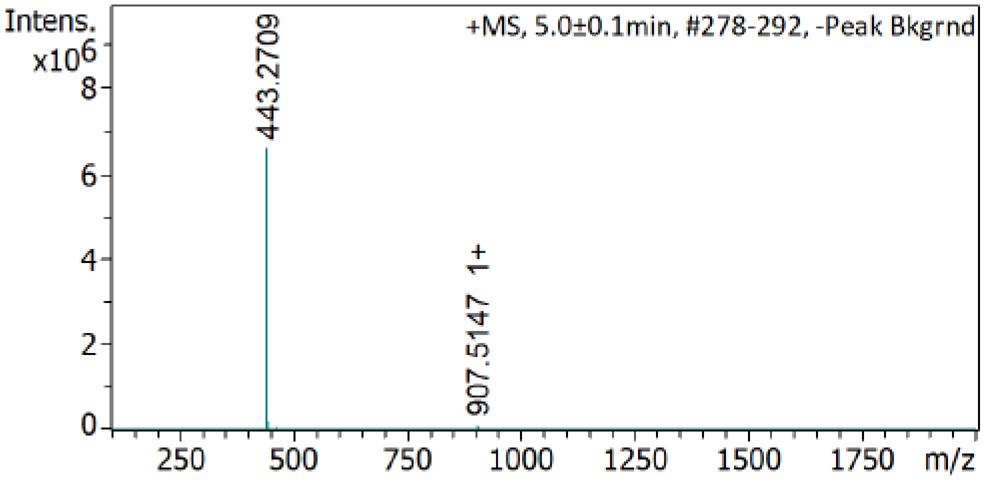

Fig. S113. Mass spectrum 8a

S38. Analytical spectra of 8b

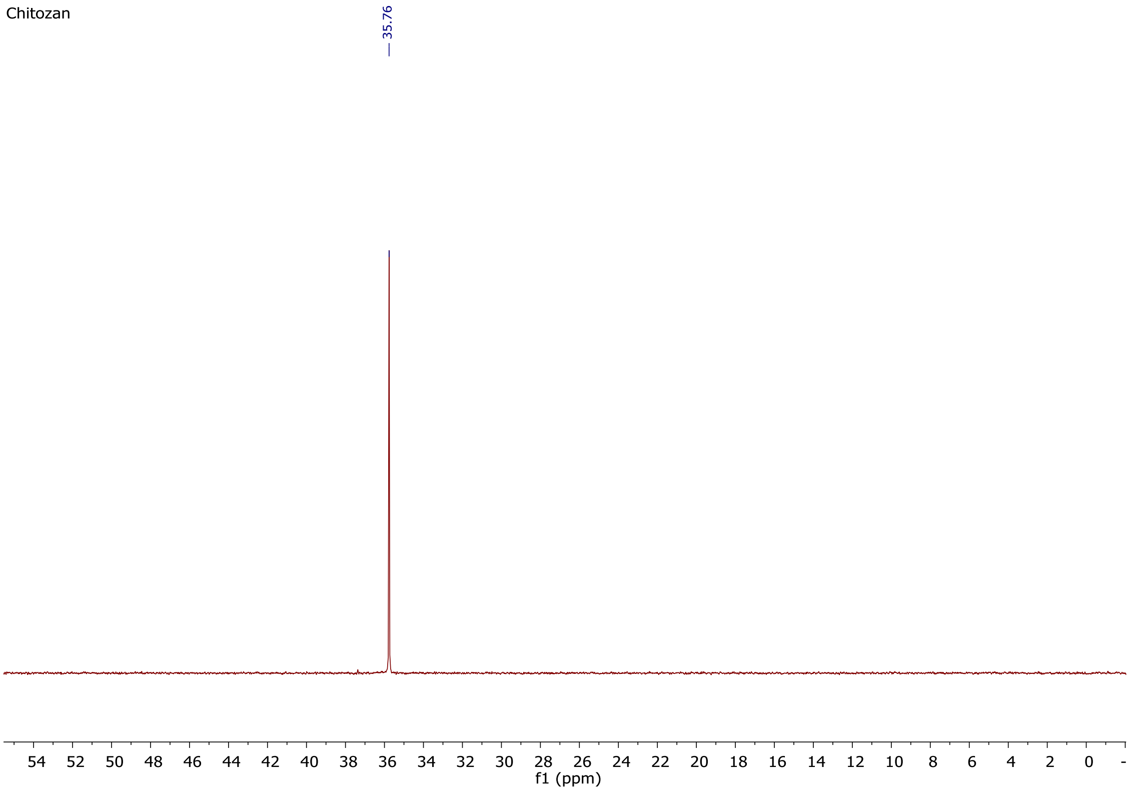

Fig. S114. ^{31}P NMR 8b ($CDCl_3$, 700 MHz).

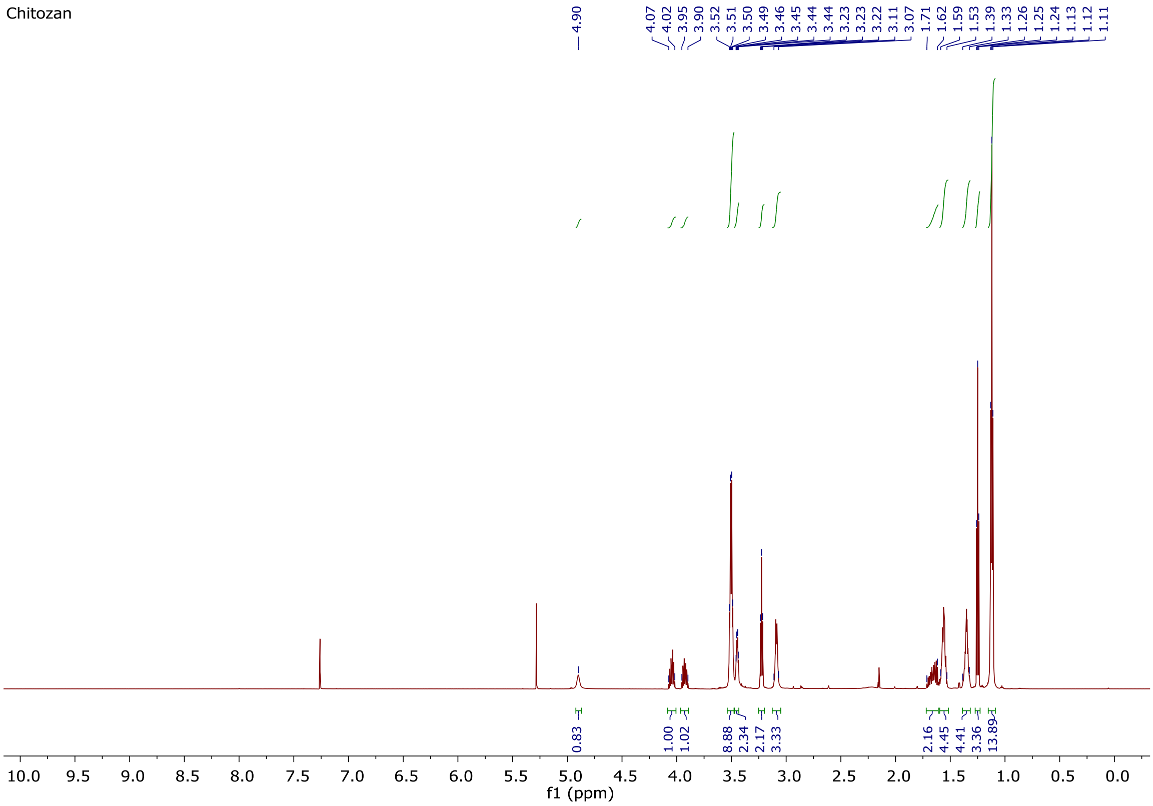

Fig. S115. ^{1}H NMR 8b ($CDCl_3$, 700 MHz).

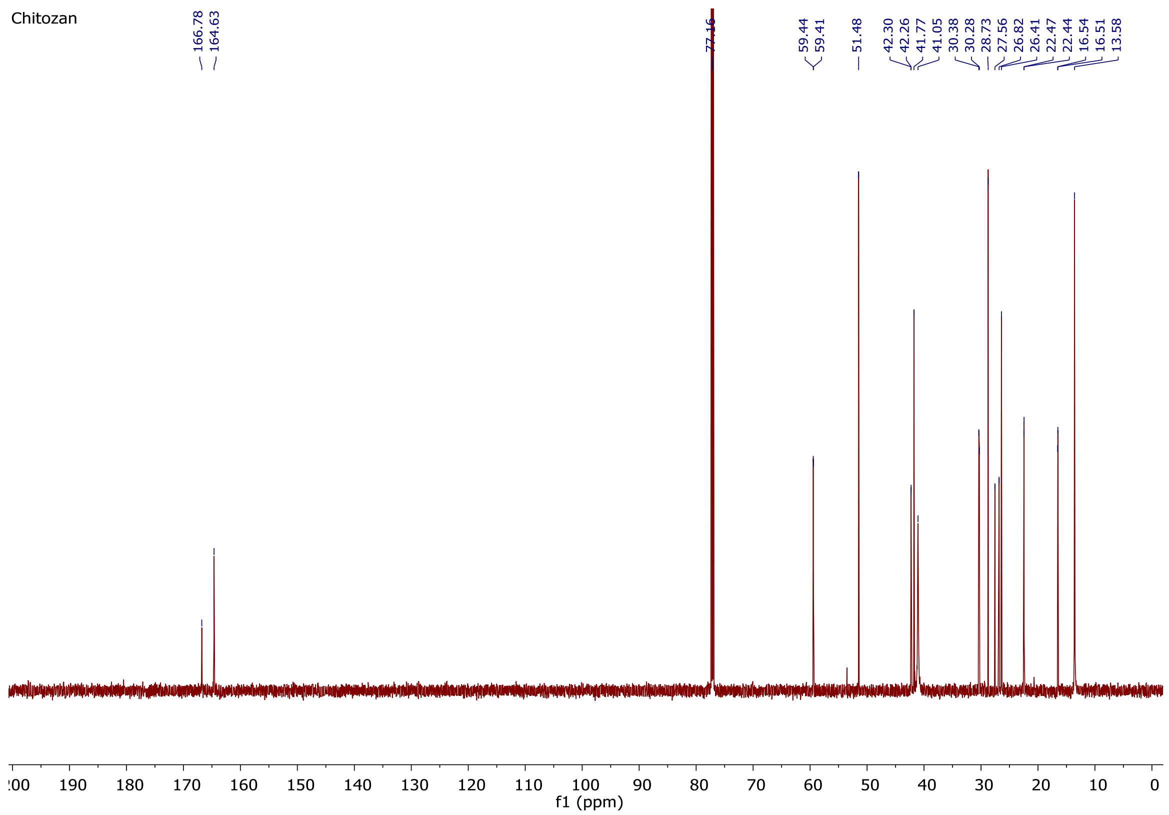

Fig. S116. ^{13}C NMR 8b ($CDCl_3$, 176 MHz).

S39. Analytical spectra of 8c

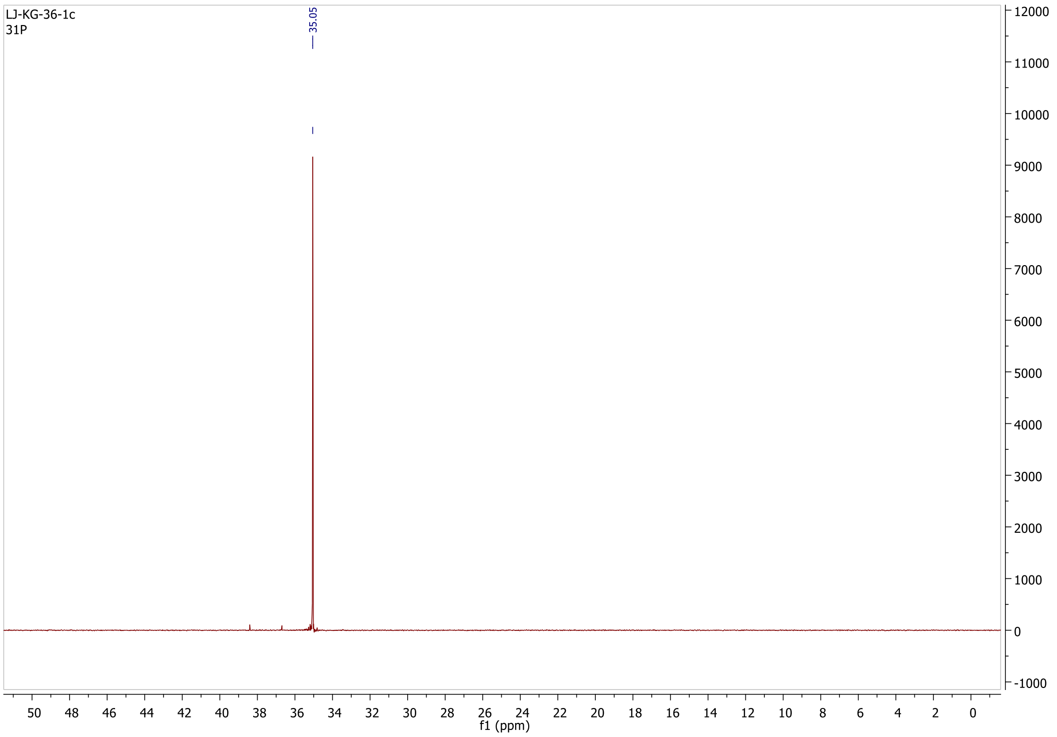

Fig. S117. ^{31}P NMR 8c ($CDCl_3$, 700 MHz).

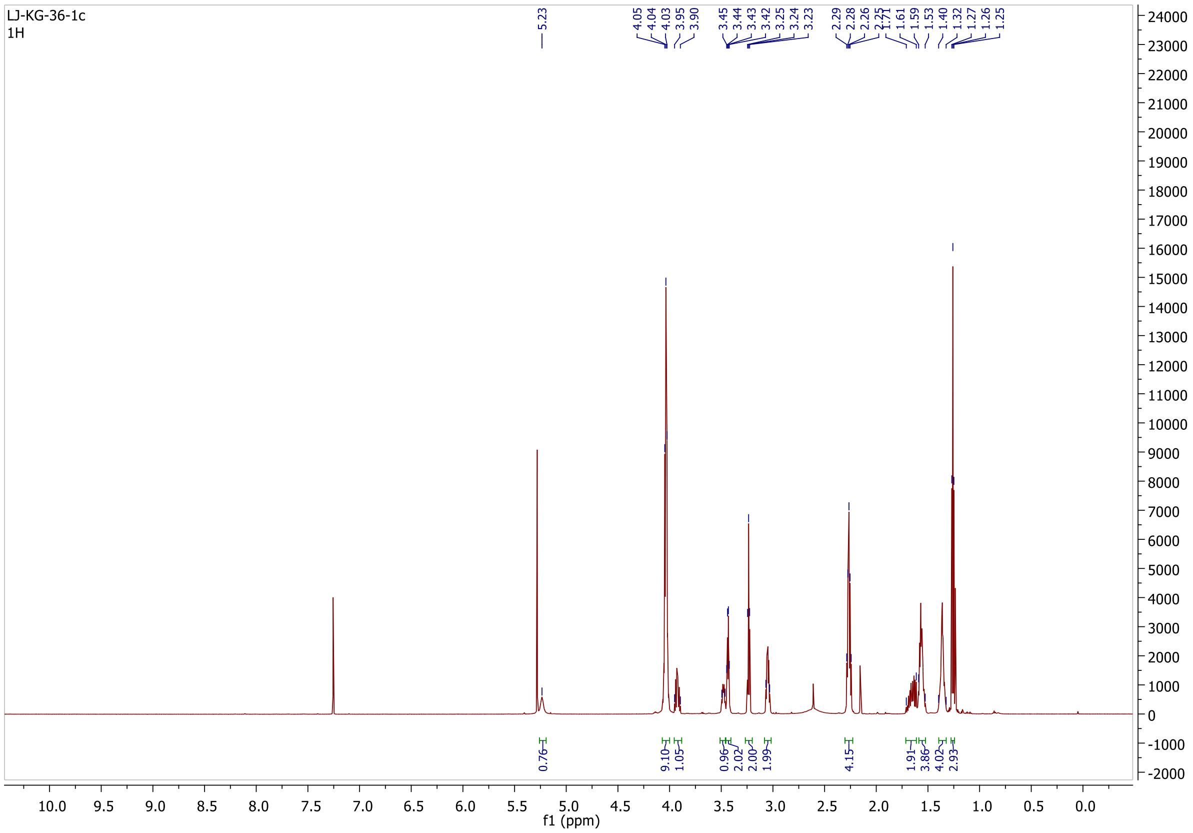

Fig. S118. ^{1}H NMR 8c ($CDCl_3$, 700 MHz).

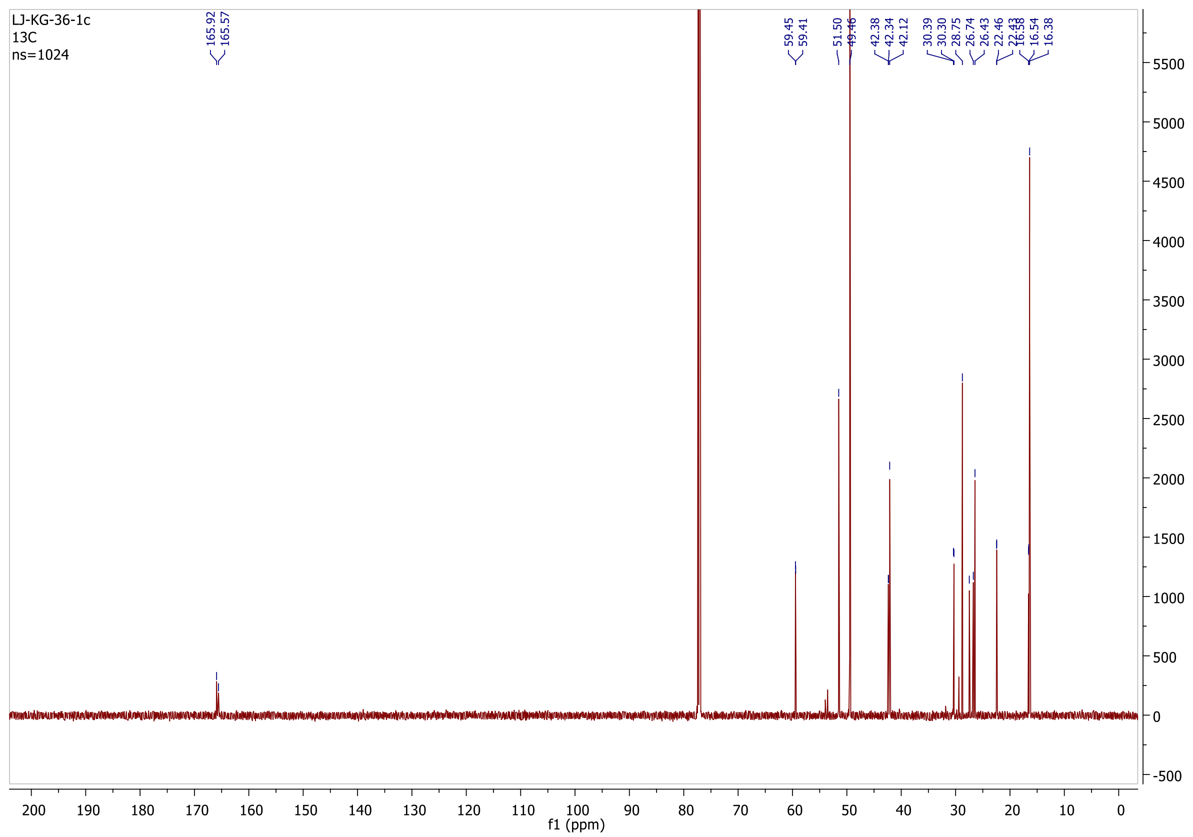

Fig. S119. ^{13}C NMR 8c ($CDCl_3$, 176 MHz).

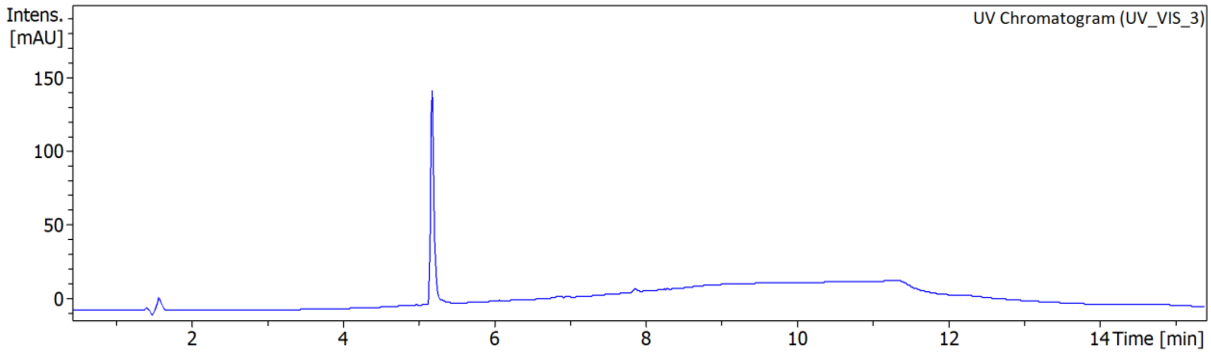

Fig. S120. UV-Vis Chromatogram 8c

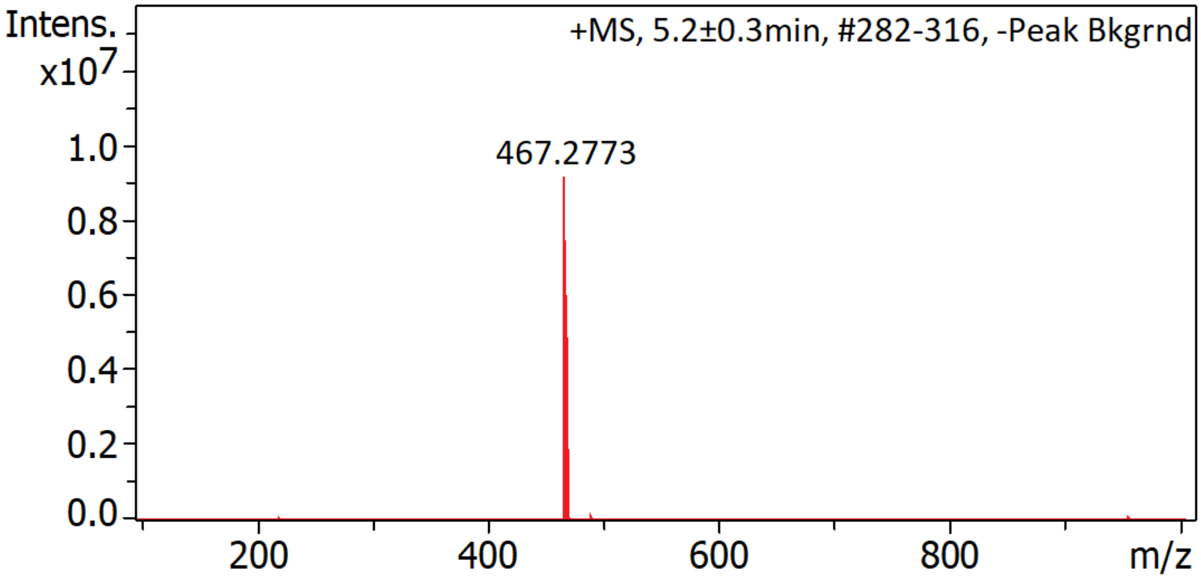

Fig. S121. Mass spectrum 8c

S40. **Analytical spectra of 8d**

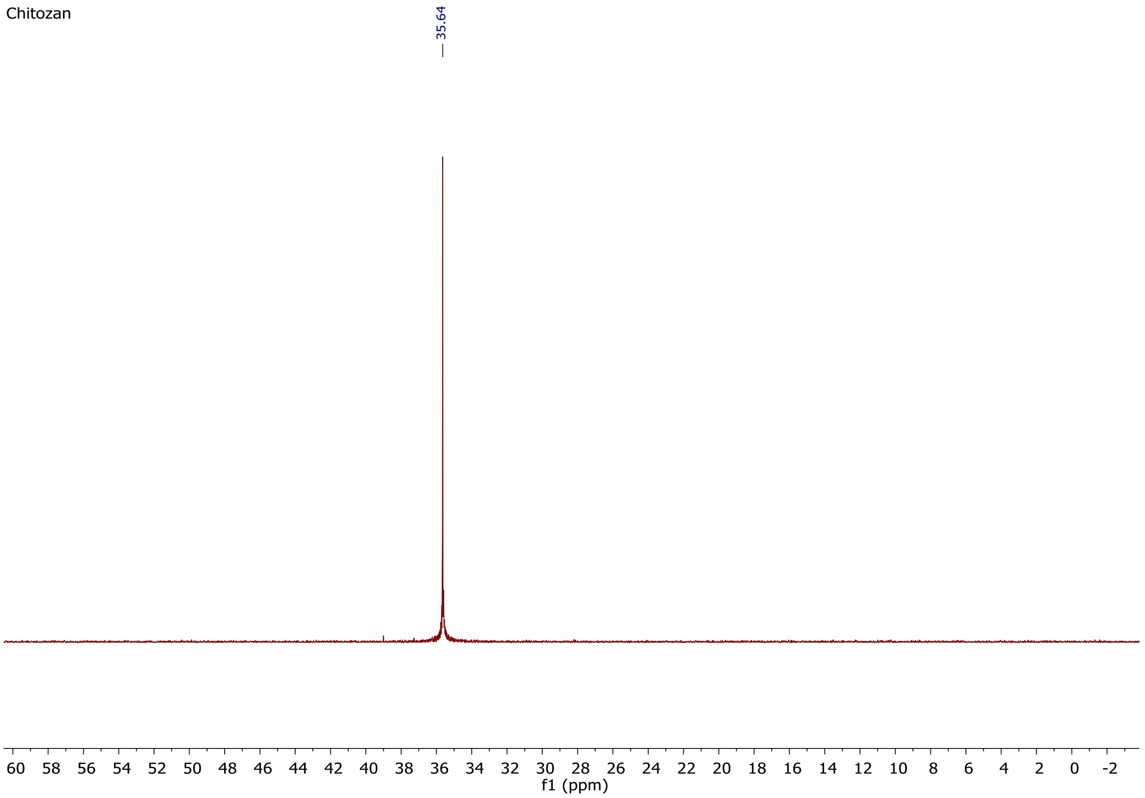

Fig. S122. ^{31}P NMR 8d ($CDCl_3$, 700 MHz).

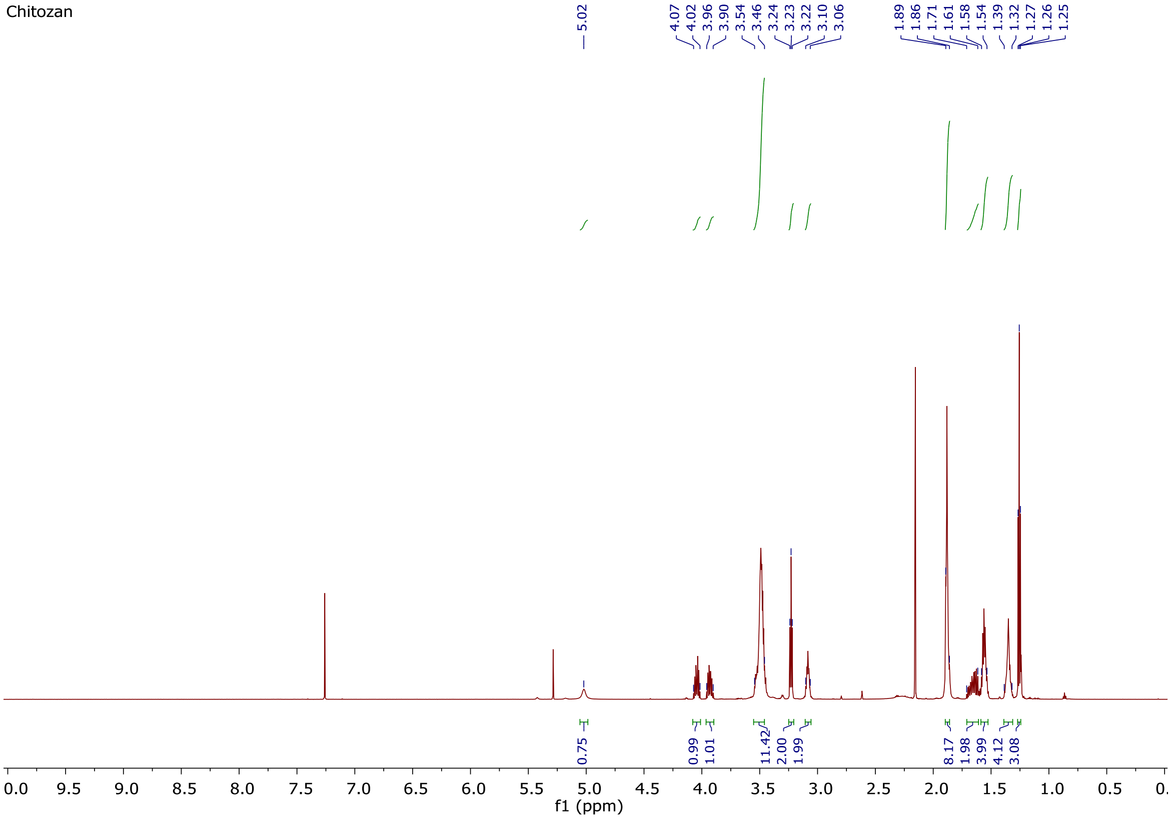

Fig. S123. ^{1}H NMR 8d ($CDCl_3$, 700 MHz).

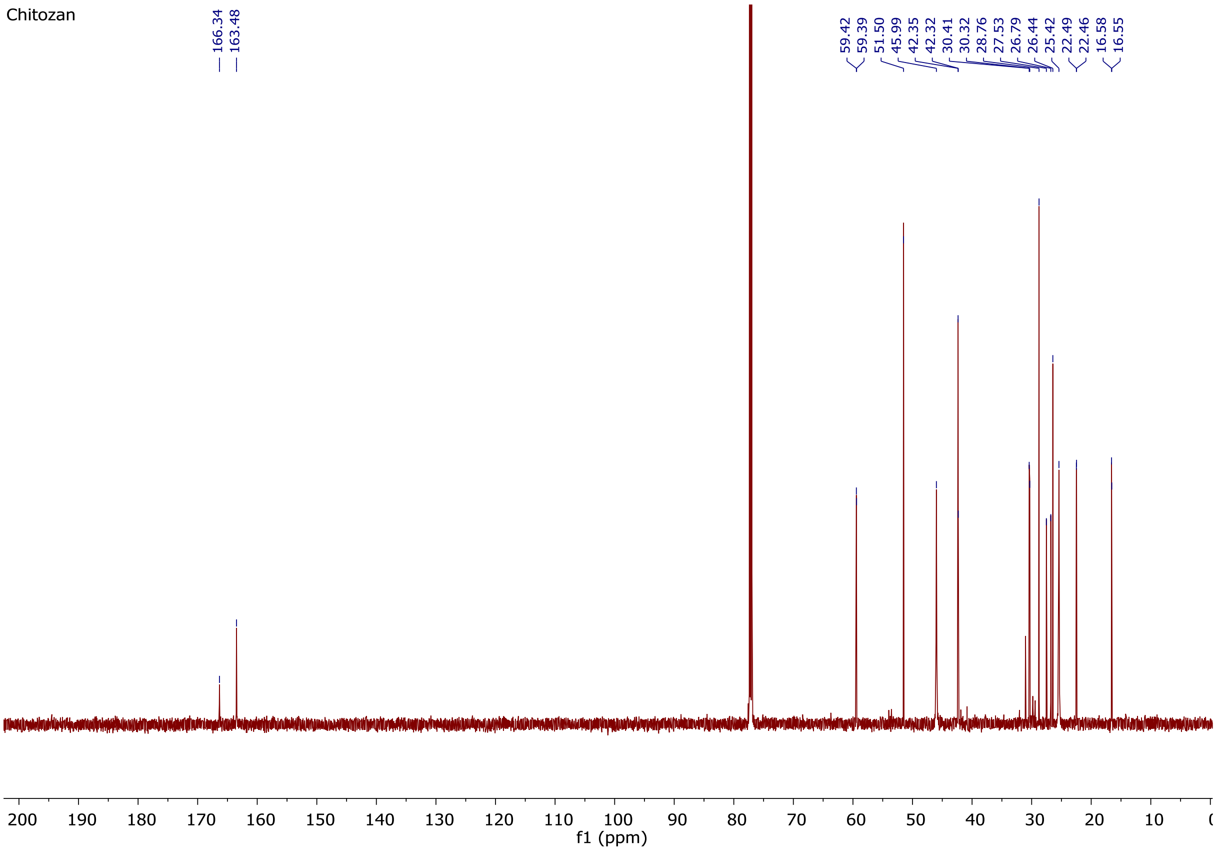

Fig. S124. ^{13}C NMR 8d ($CDCl_3$, 176 MHz).

S41. Analytical spectra of 8e

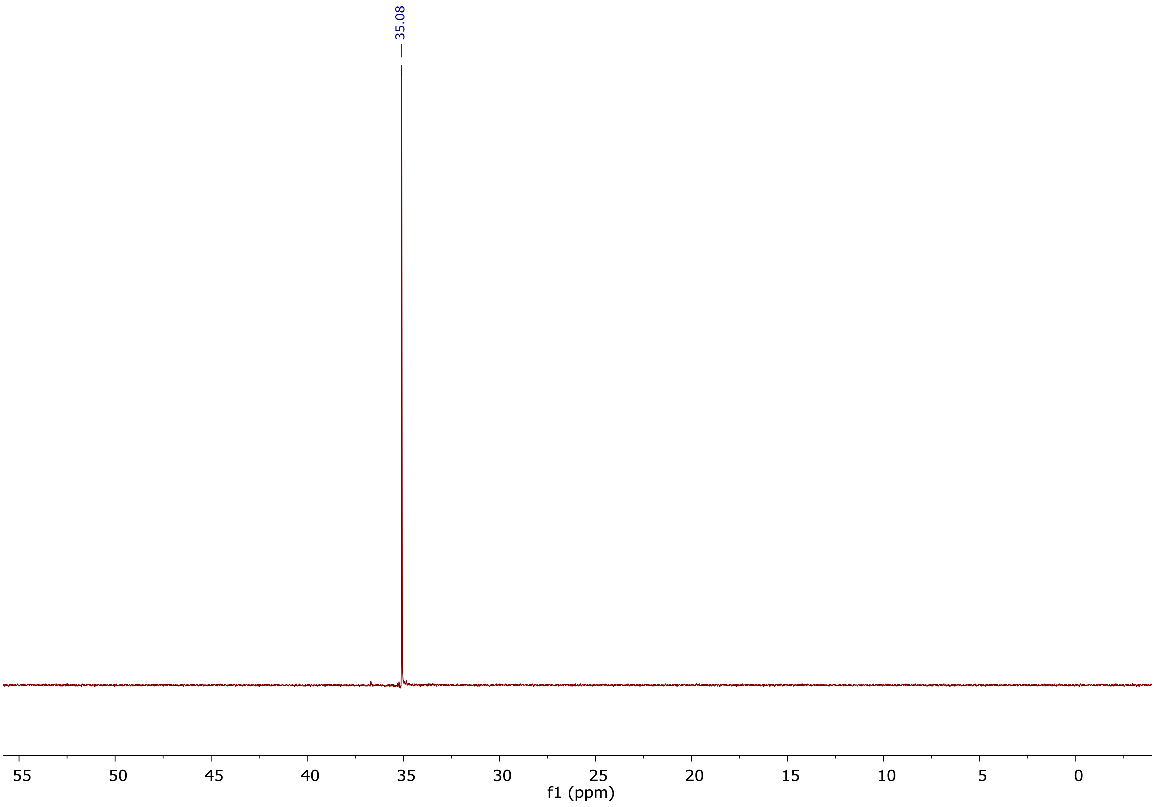

Fig. S125. ^{31}P NMR 8e ($CDCl_3$, 700 MHz).

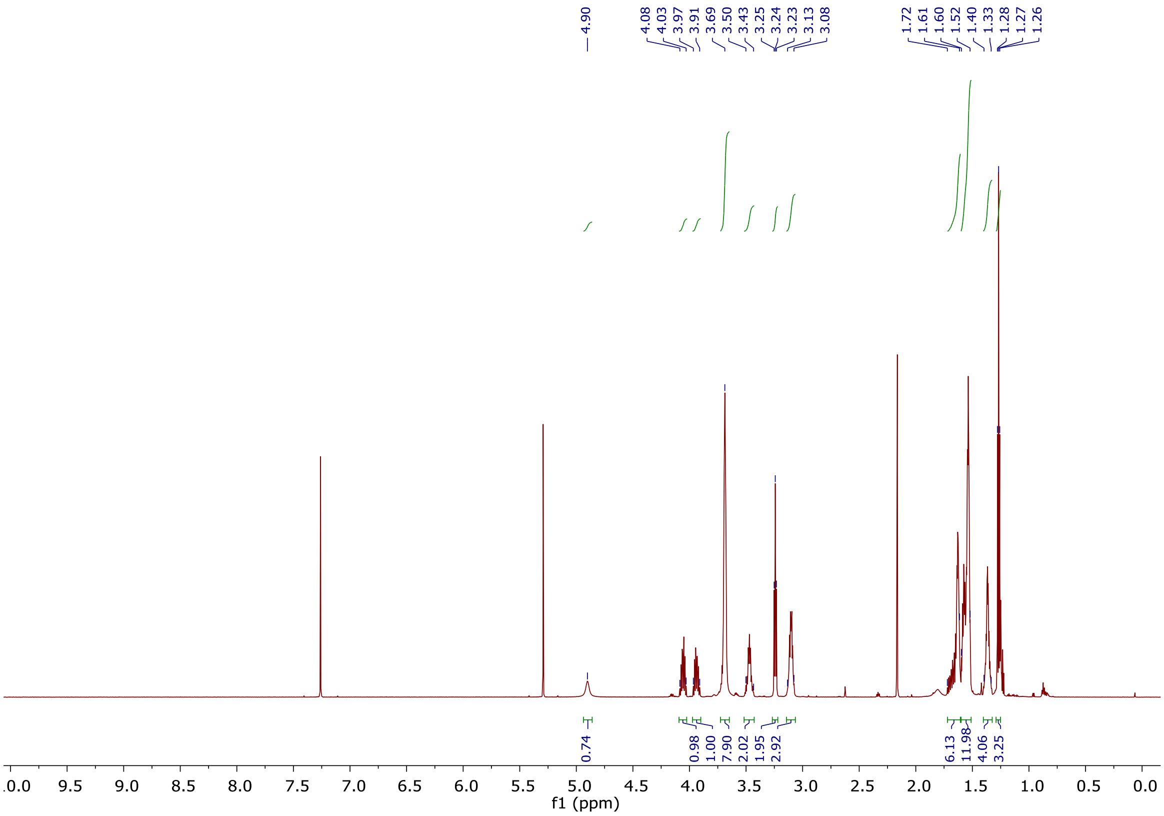

Fig. S126. ^{1}H NMR 8e ($CDCl_3$, 700 MHz).

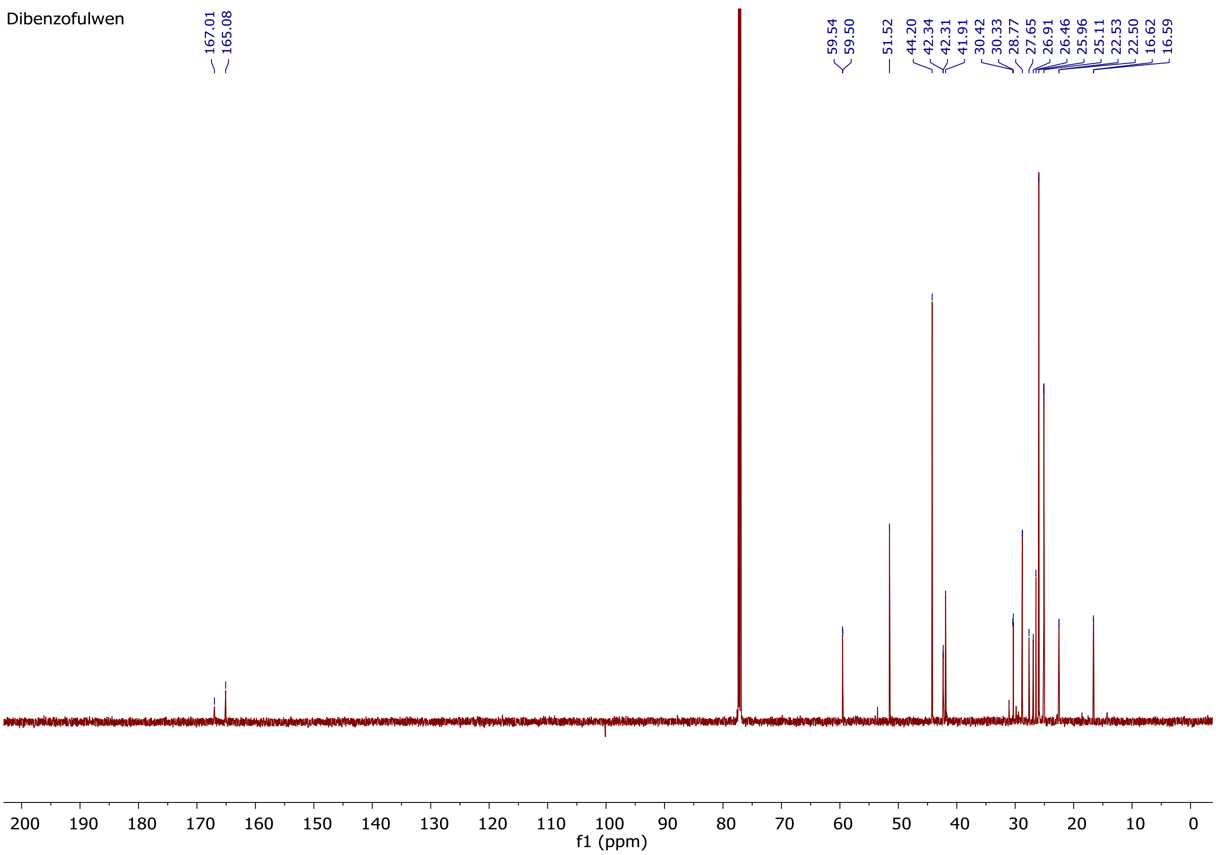

Fig. S127. ^{13}C NMR 8e ($CDCl_3$, 176 MHz).

S42. Analytical spectra of 8f

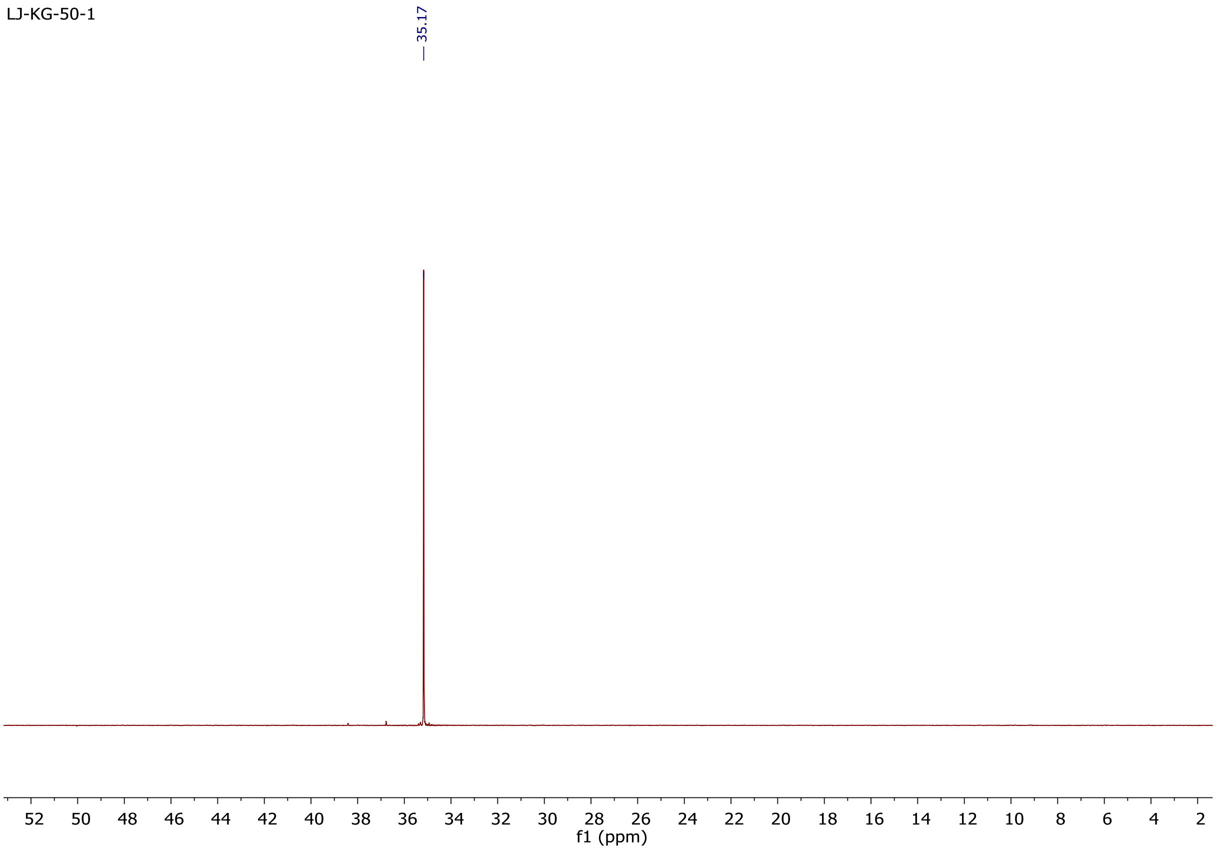

Fig. S128. ^{31}P NMR 8f ($CDCl_3$, 700 MHz).

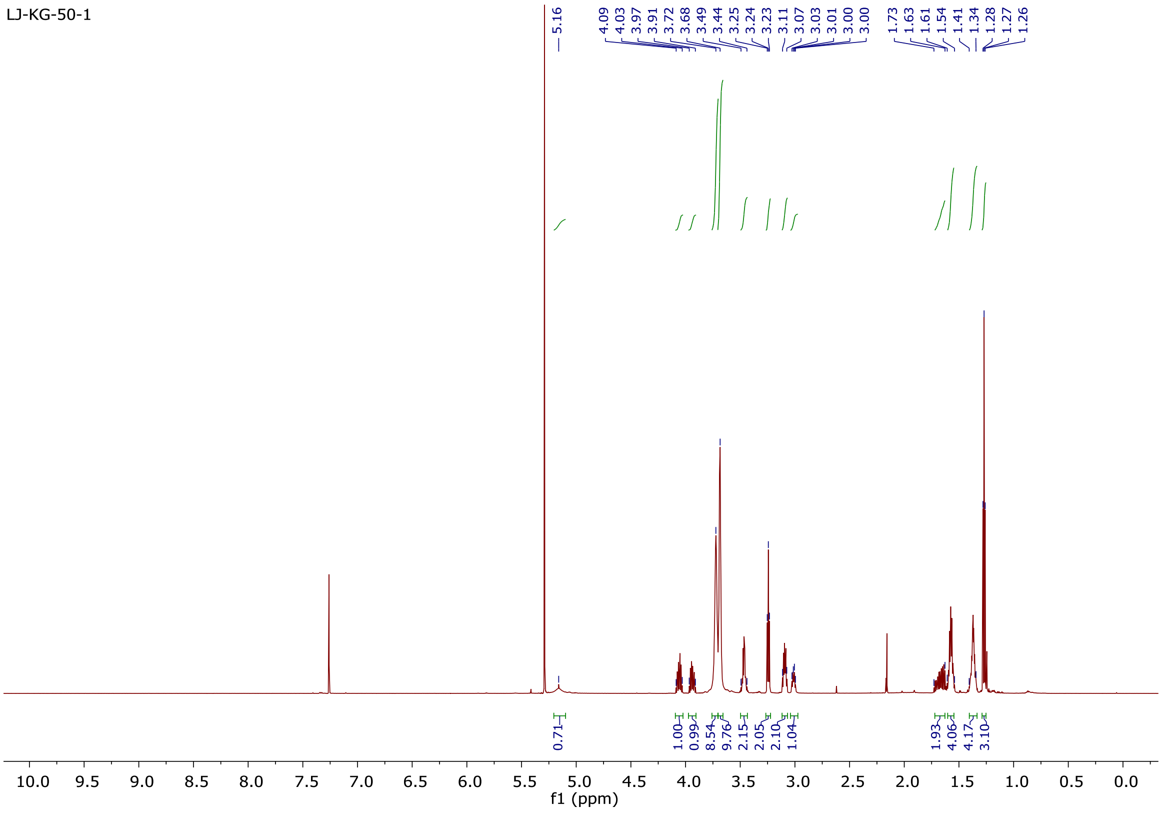

Fig. S129. ^{1}H NMR 8f ($CDCl_3$, 700 MHz).

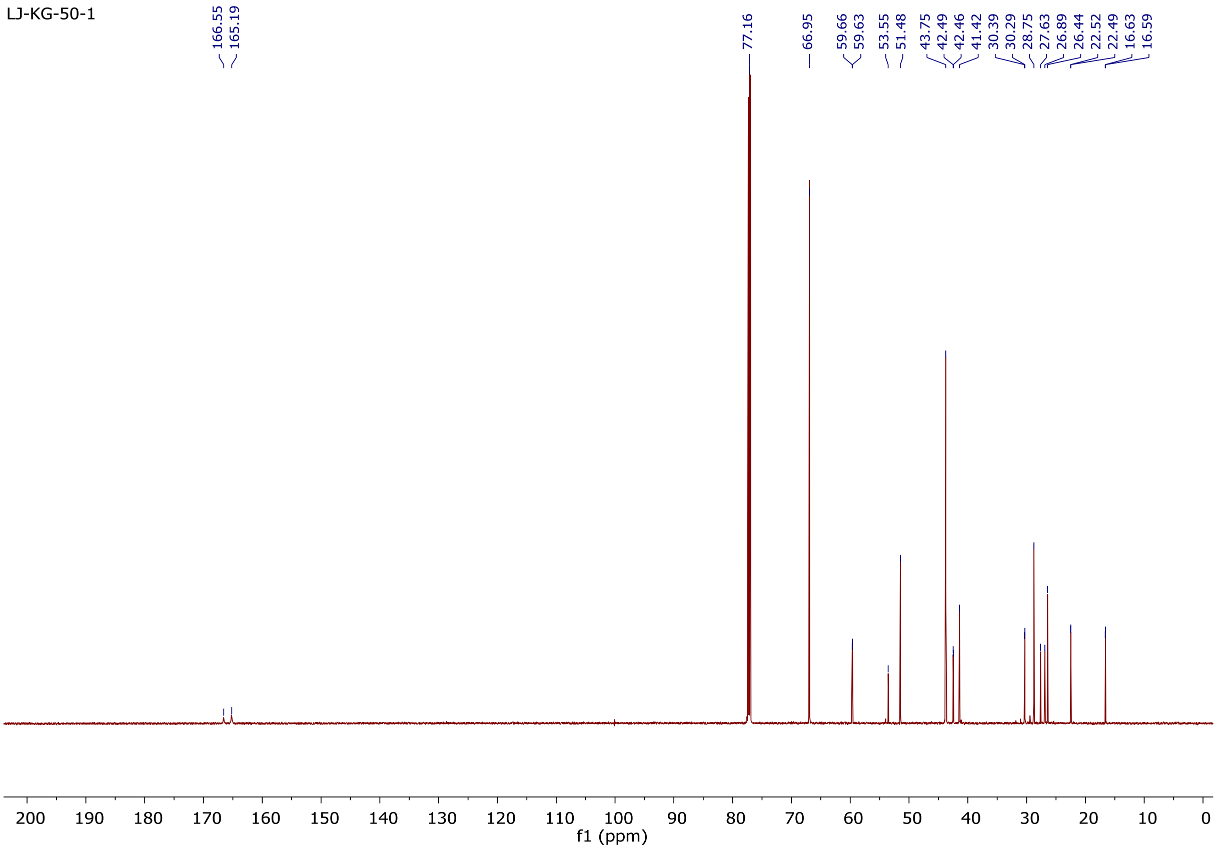

Fig. S130. ^{13}C NMR 8f ($CDCl_3$, 176 MHz).

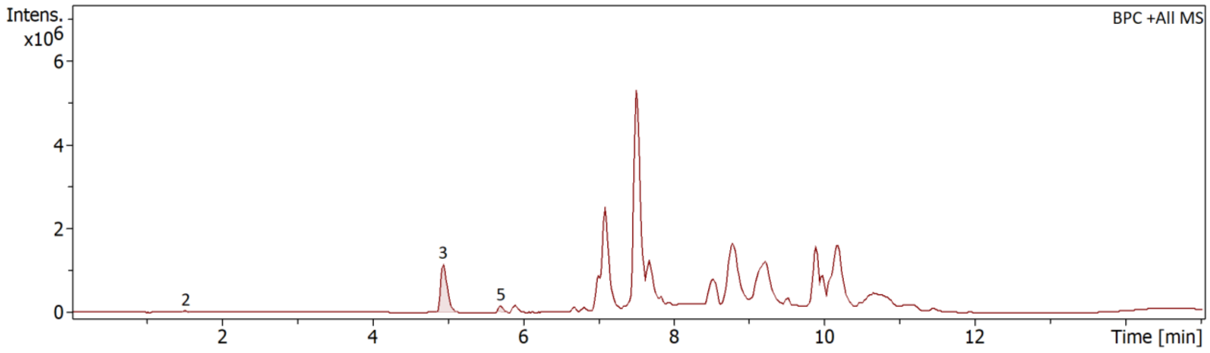

Fig. S131. LC-MS 8f

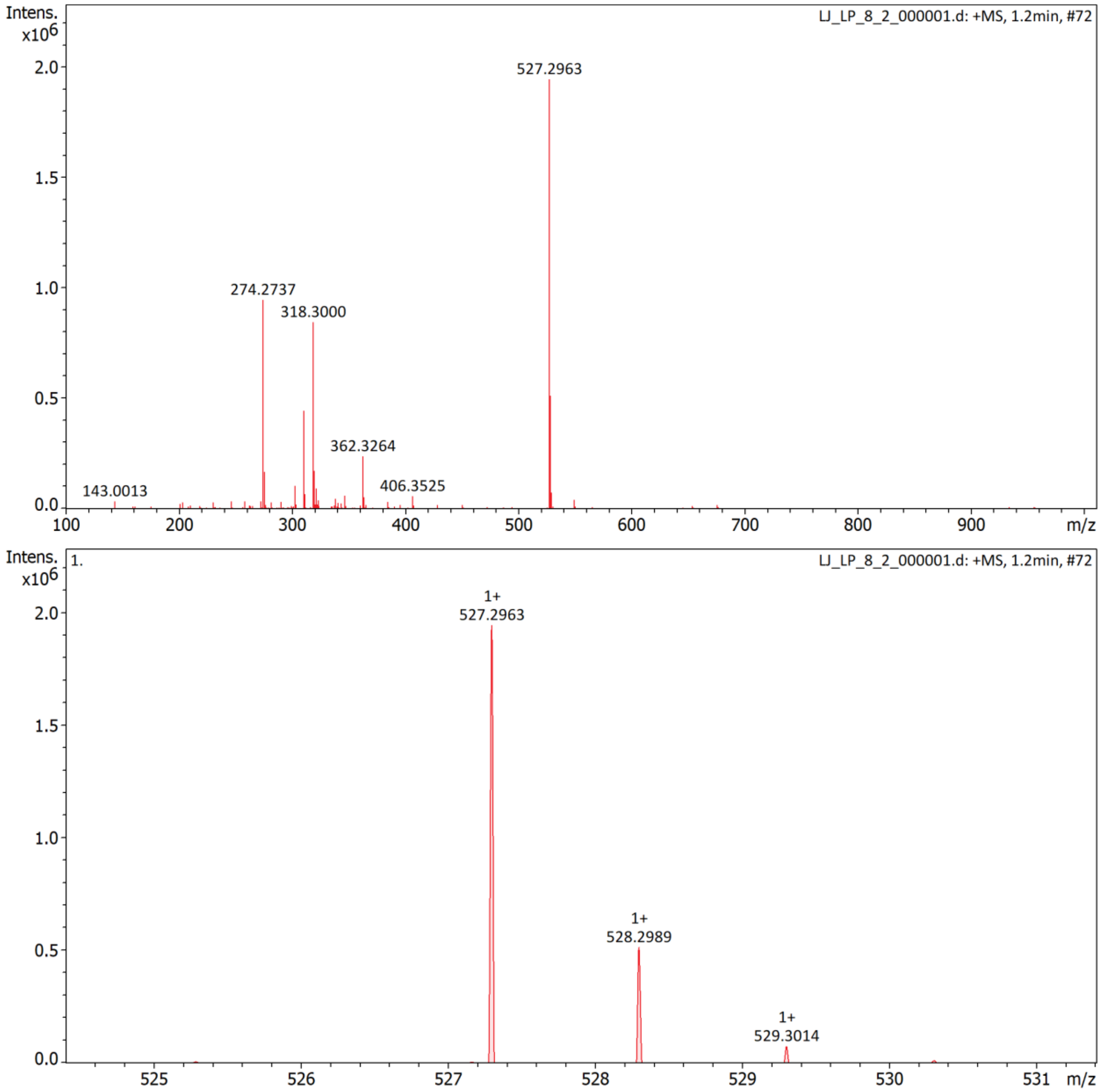

Fig. S132. Mass spectrum 8f

S43. Analytical spectra of 8g

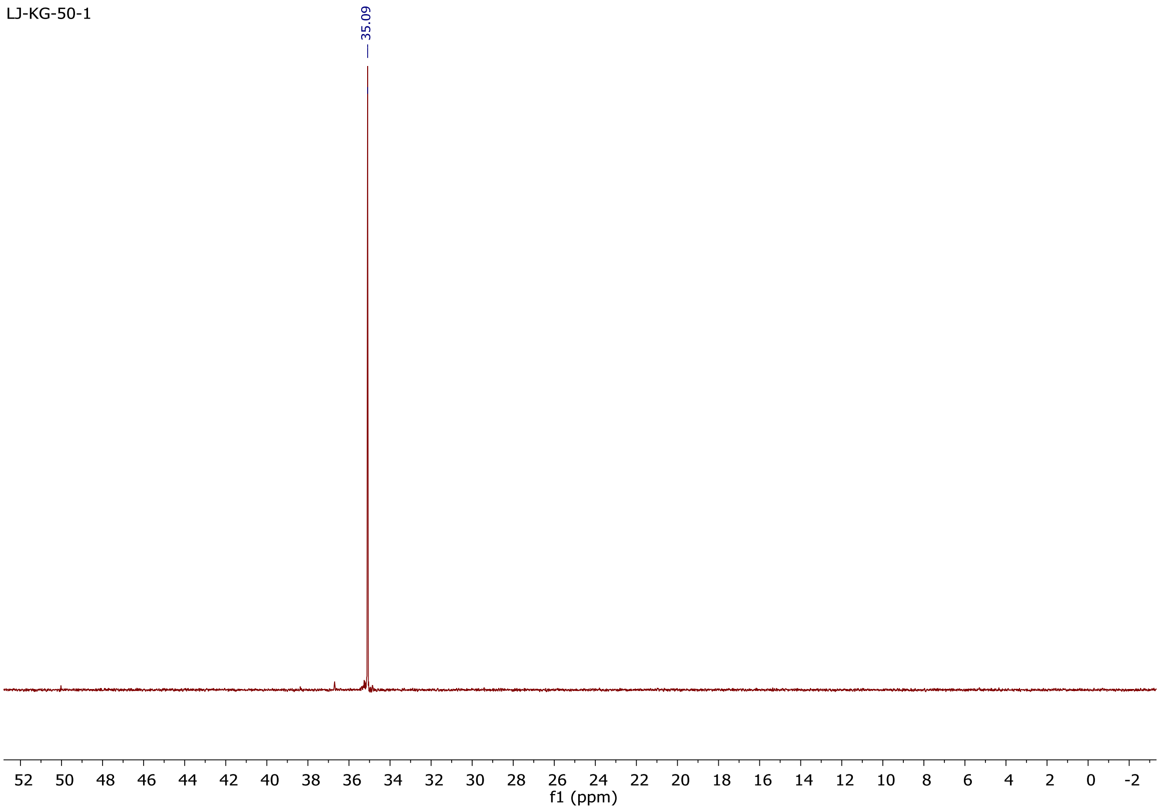

Fig. S133. ^{31}P NMR 8g ($CDCl_3$, 700 MHz).

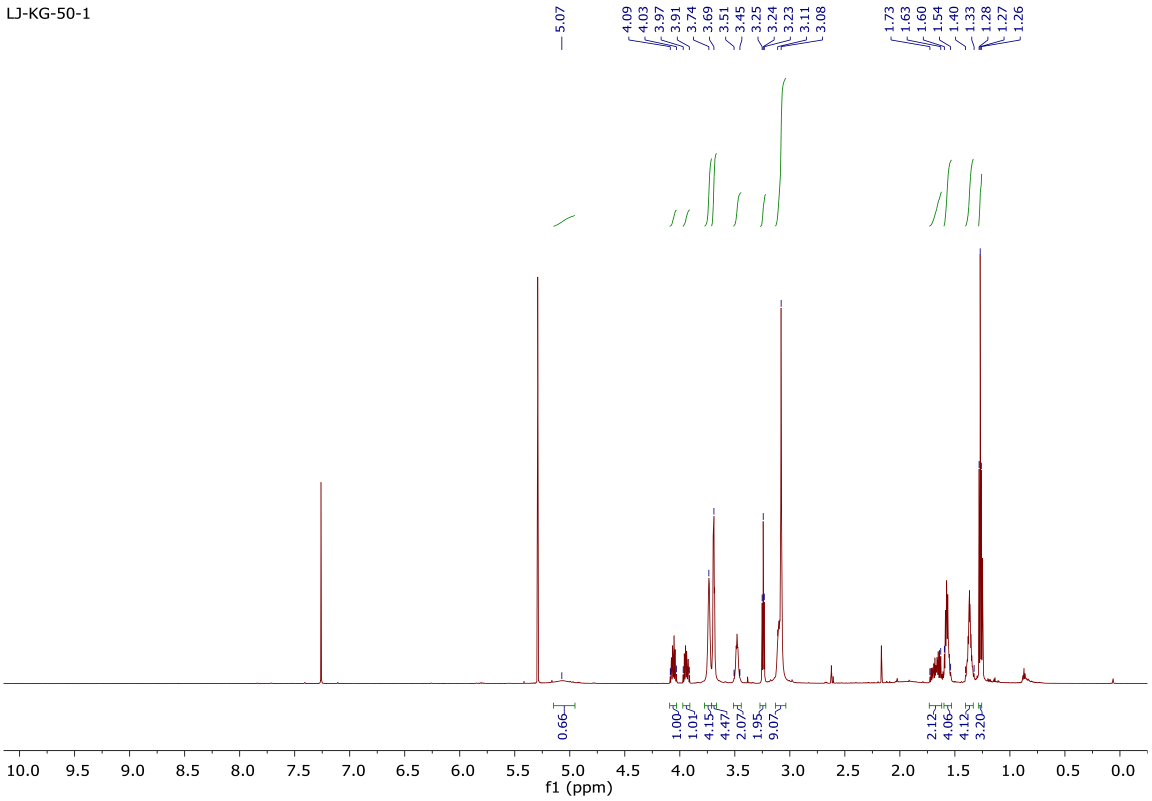

Fig. S134. ^{1}H NMR 8g ($CDCl_3$, 700 MHz).

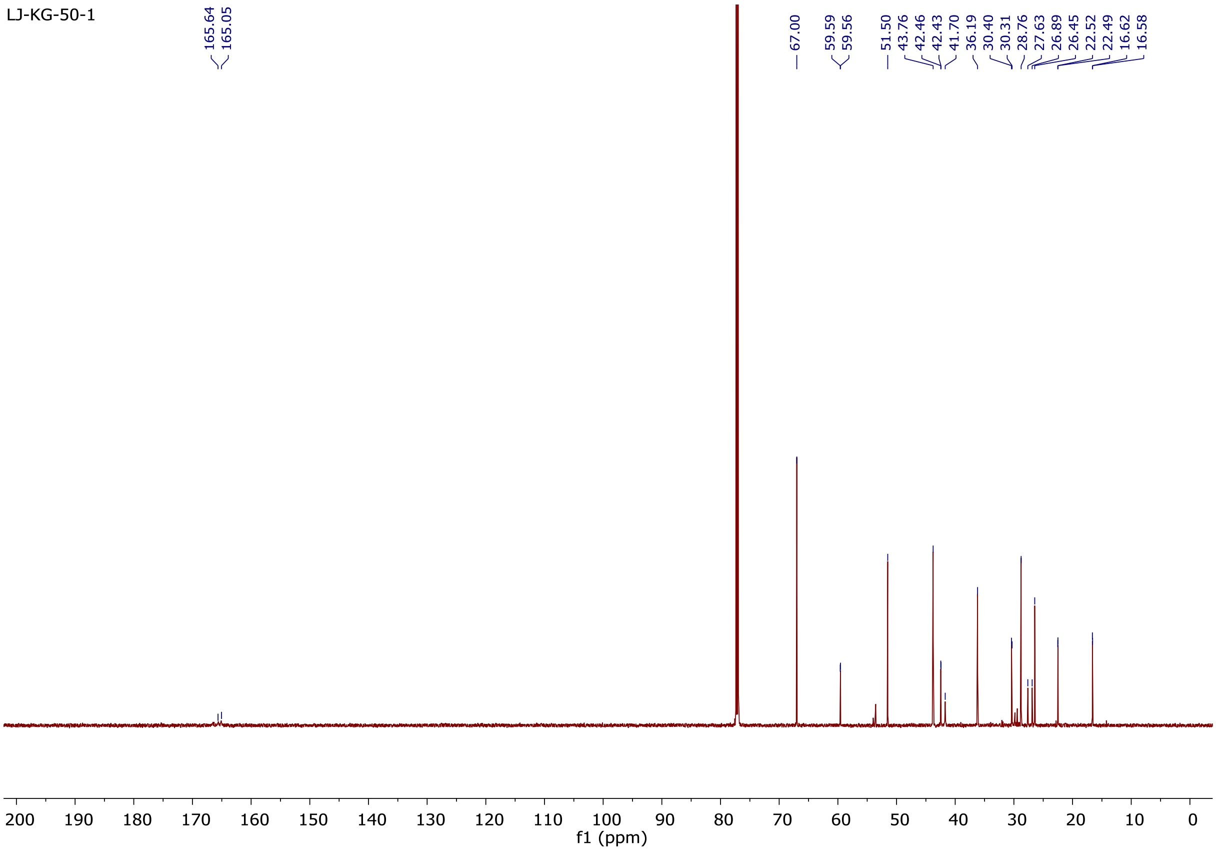

Fig. S135. ^{13}C NMR 8g ($CDCl_3$, 176 MHz).

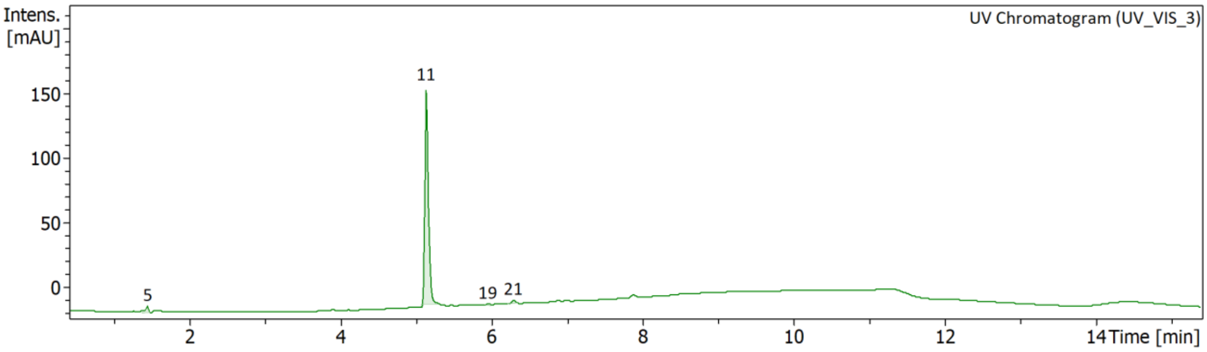

Fig. S136. UV-Vis Chromatogram 8g

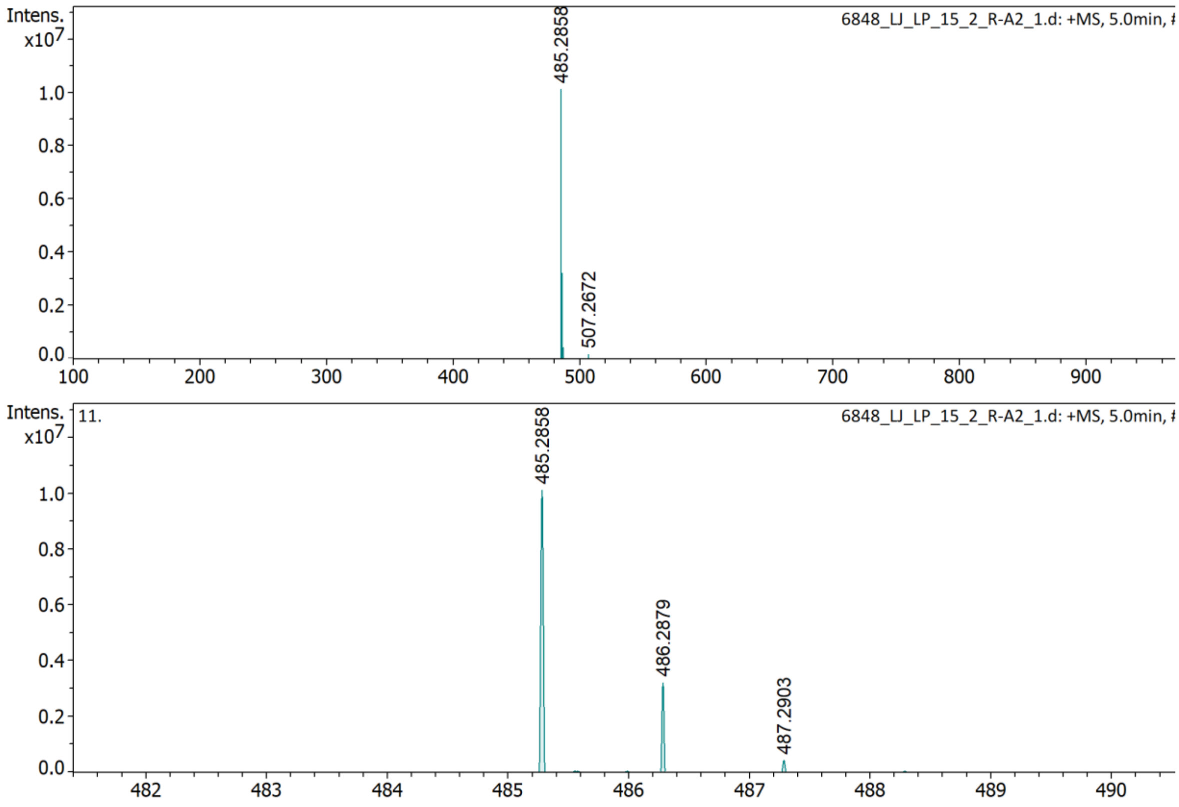

Fig. S137. Mass spectrum 8g

S44. Analytical spectra of **8h**

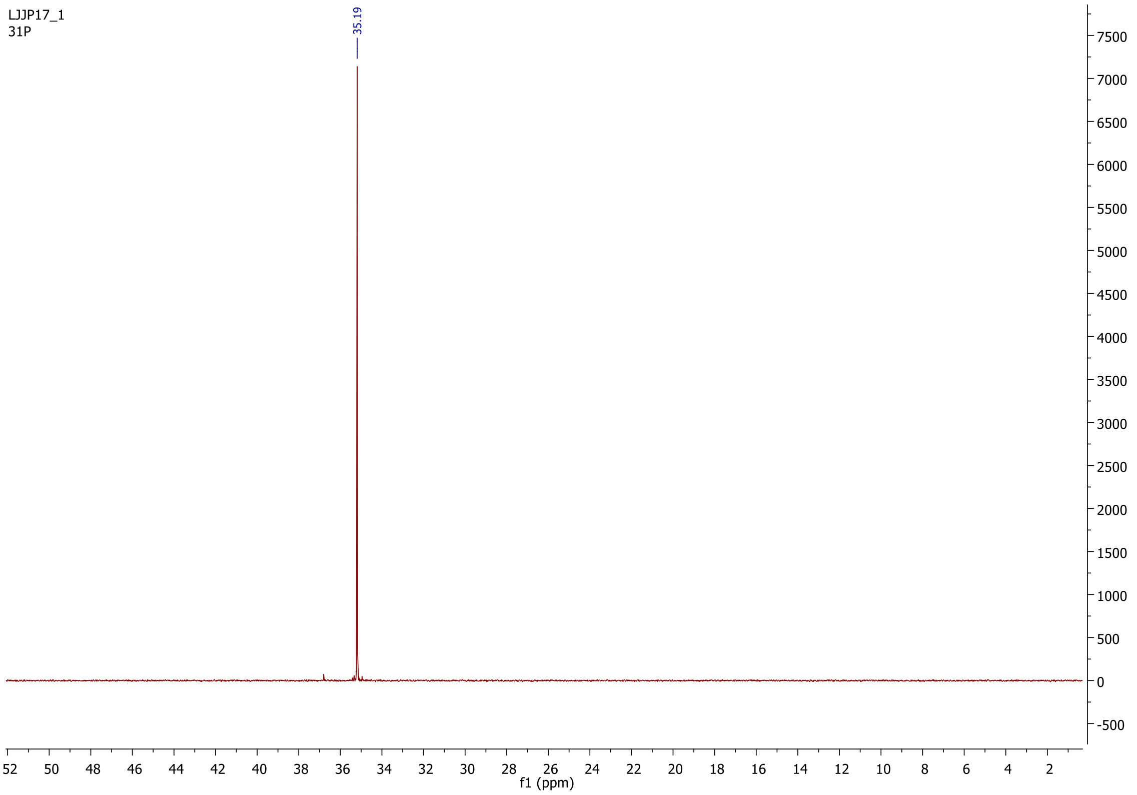

Fig. S138. ^{31}P NMR 8h ($CDCl_3$, 700 MHz).

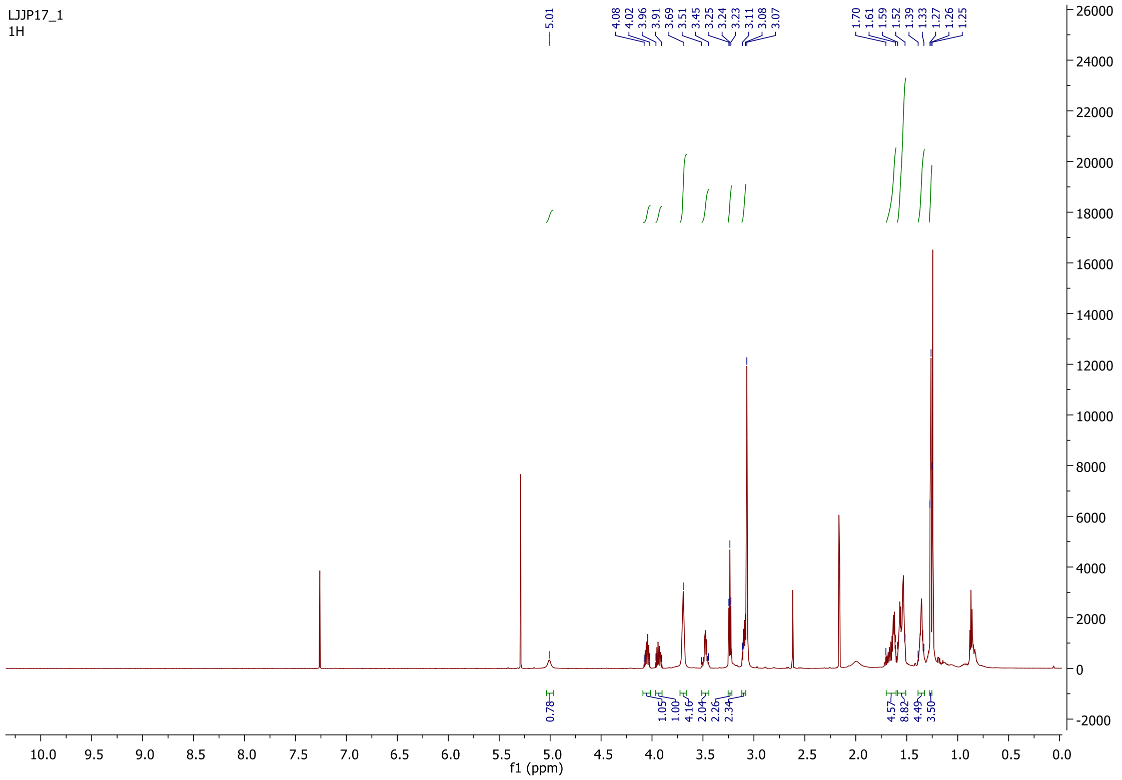

Fig. S139. ^{1}H NMR 8h ($CDCl_3$, 700 MHz).

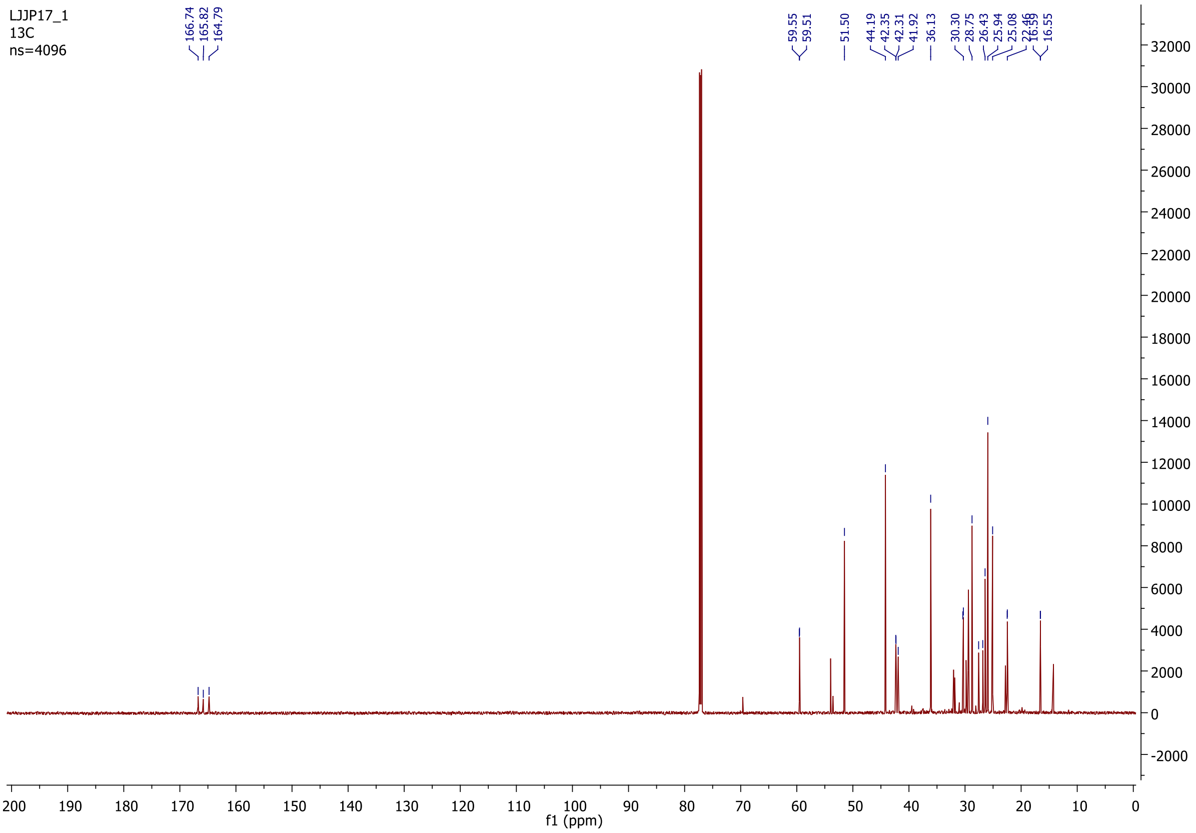

Fig. S140. ^{13}C NMR 8h ($CDCl_3$, 176 MHz).

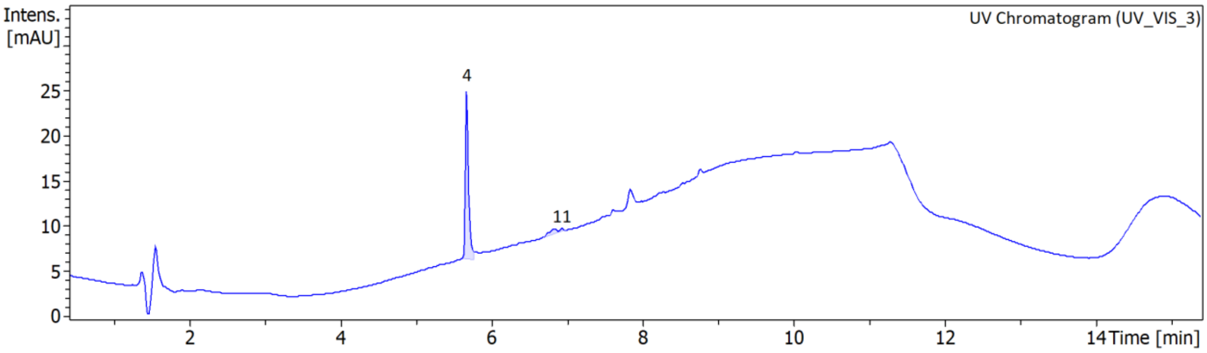

Fig. S141. UV-Vis Chromatogram 8h

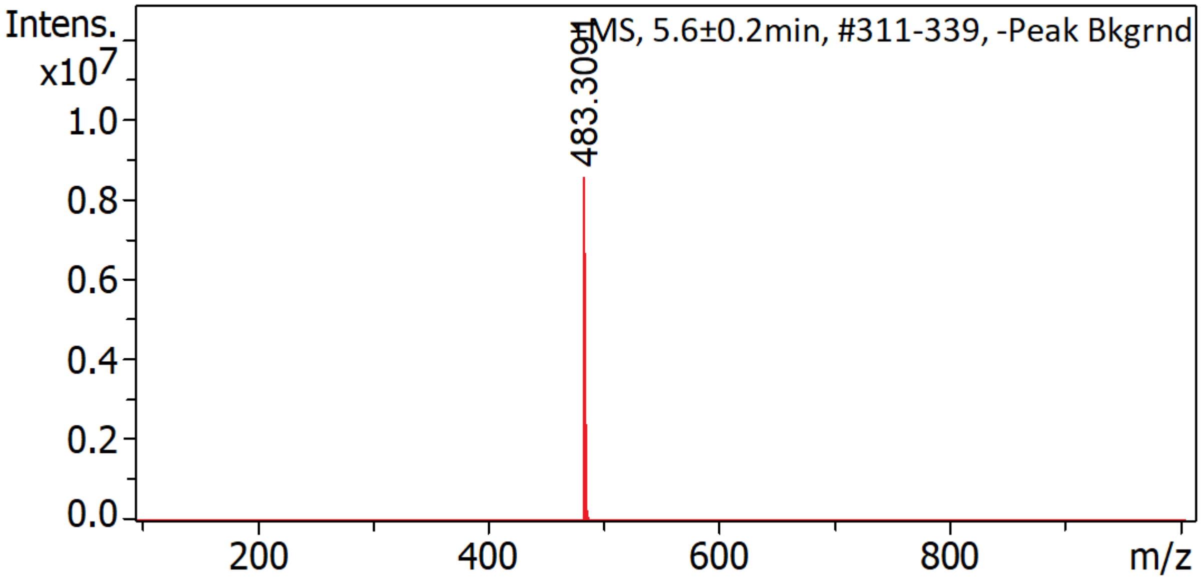

Fig. S142. Mass spectrum 8h

S45. Analytical spectra of **8i**

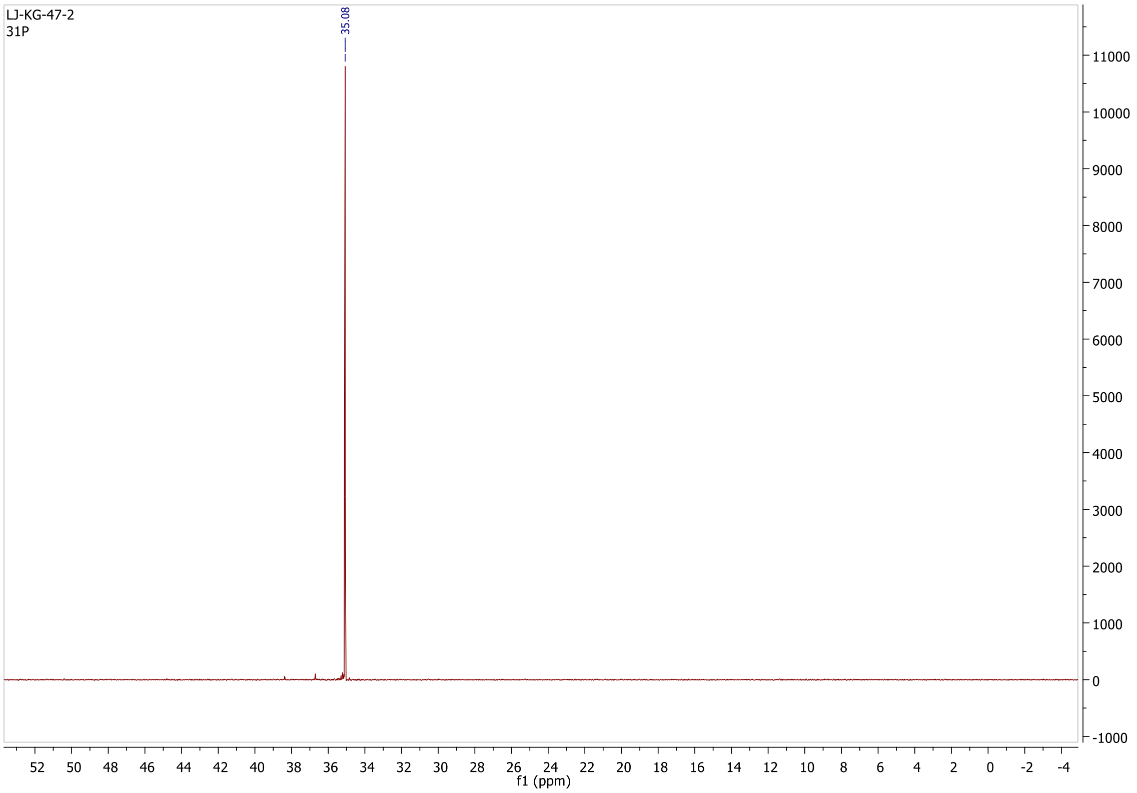

Fig. S143. ^{31}P NMR 8i ($CDCl_3$, 700 MHz).

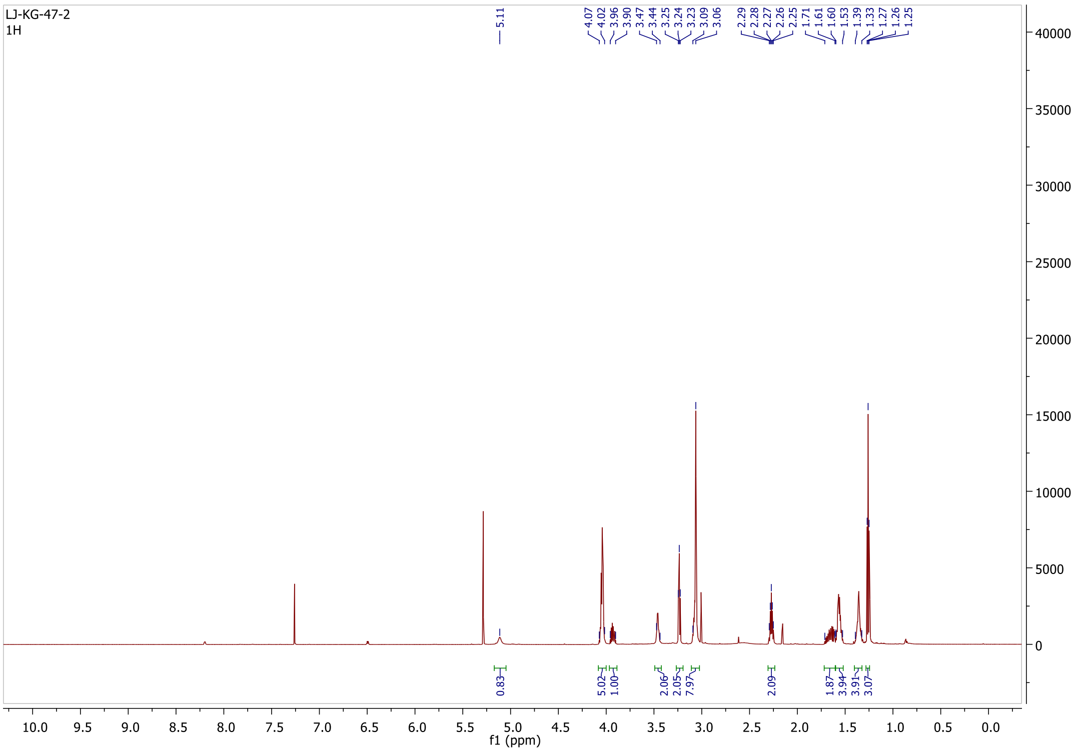

Fig. S144. ^{1}H NMR 8i ($CDCl_3$, 700 MHz).

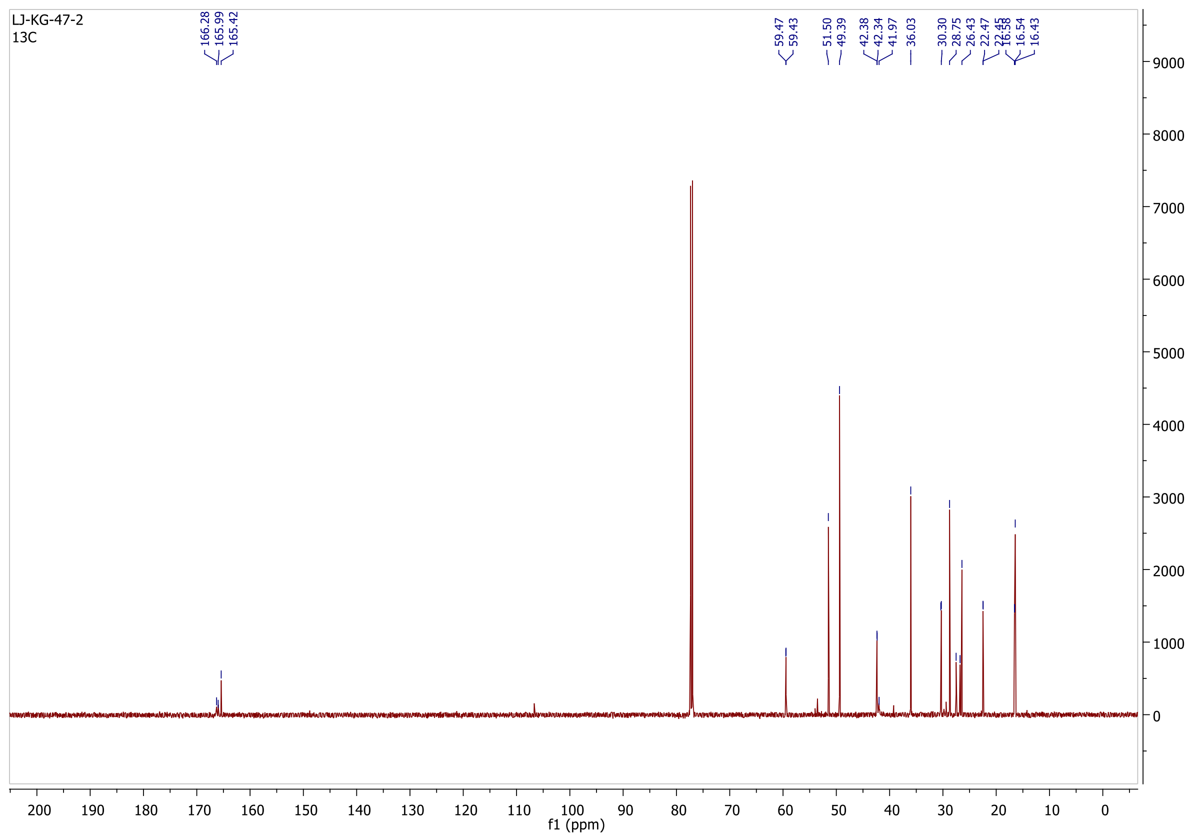

Fig. S145. ^{13}C NMR 8i ($CDCl_3$, 176 MHz).

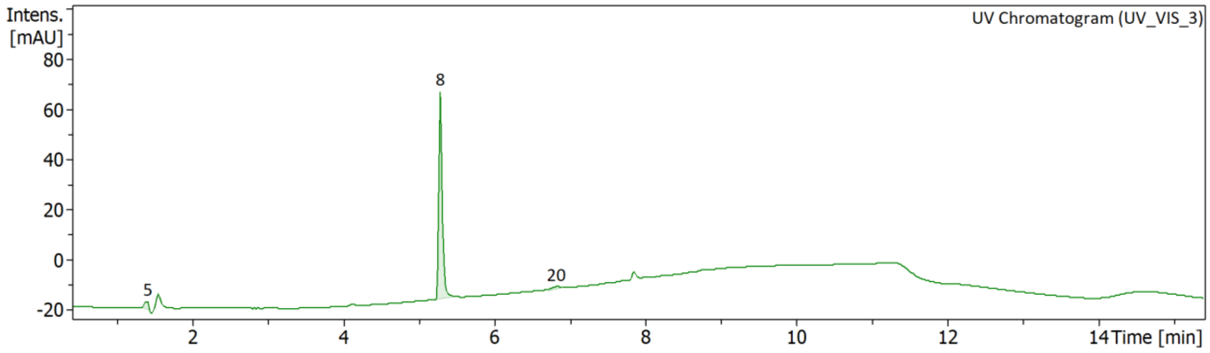

Fig. S146. UV-Vis Chromatogram 8i

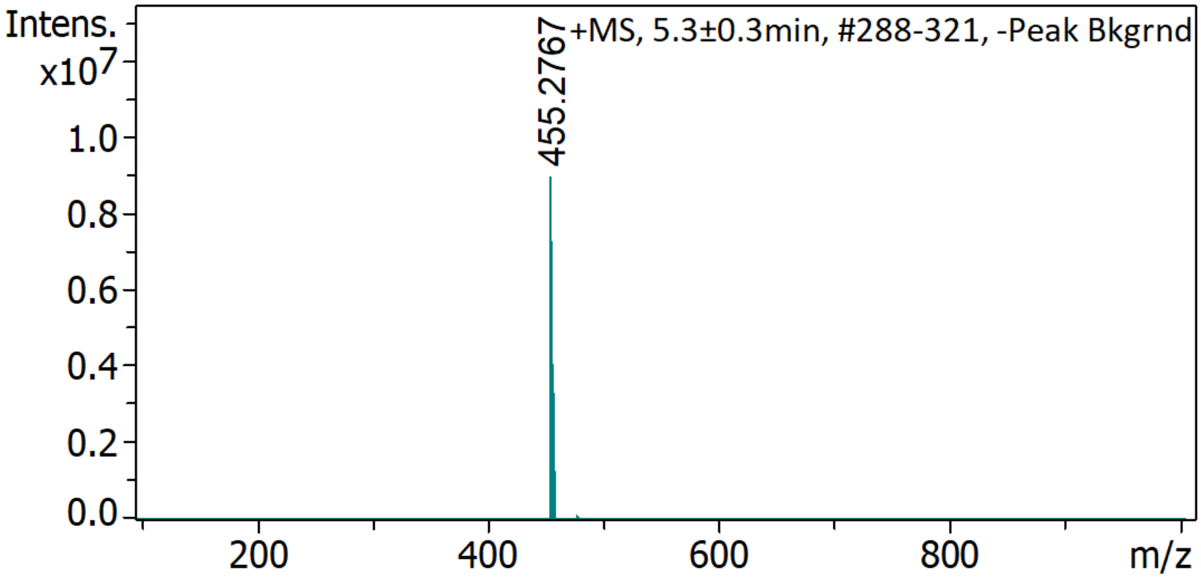

Fig. S147. Mass spectrum 8i

S46. Analytical spectra of 8j

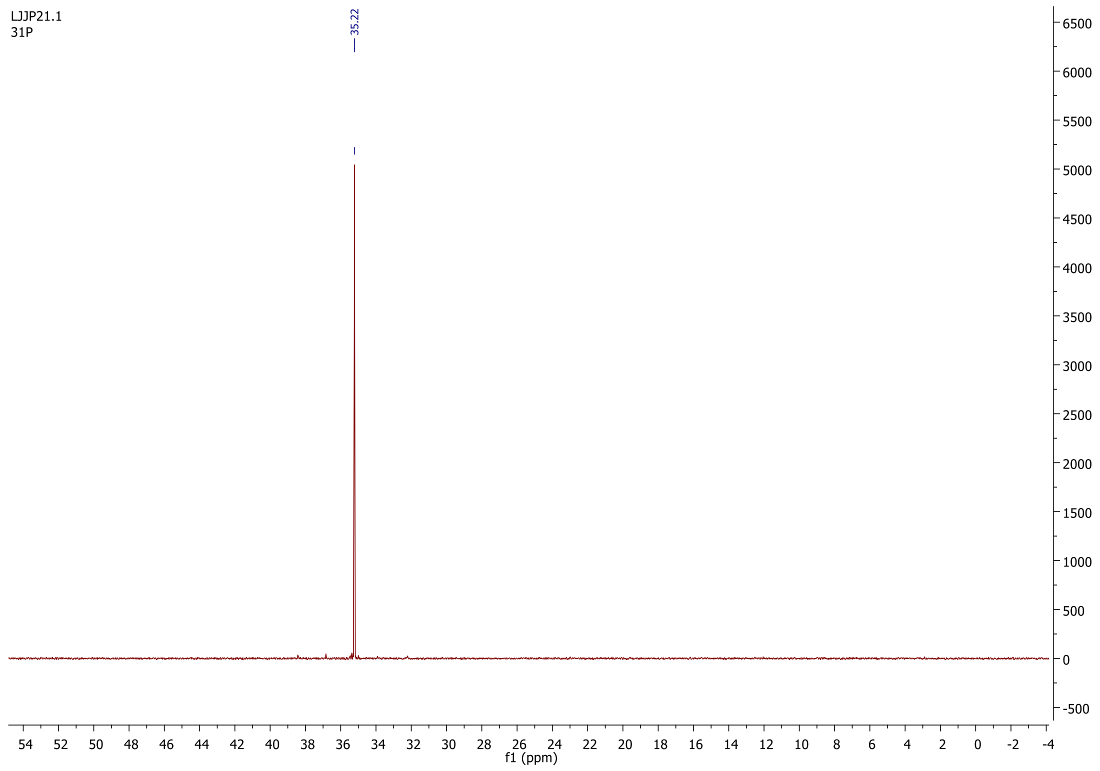

Fig. S148. ^{31}P NMR 8j ($CDCl_3$, 700 MHz).

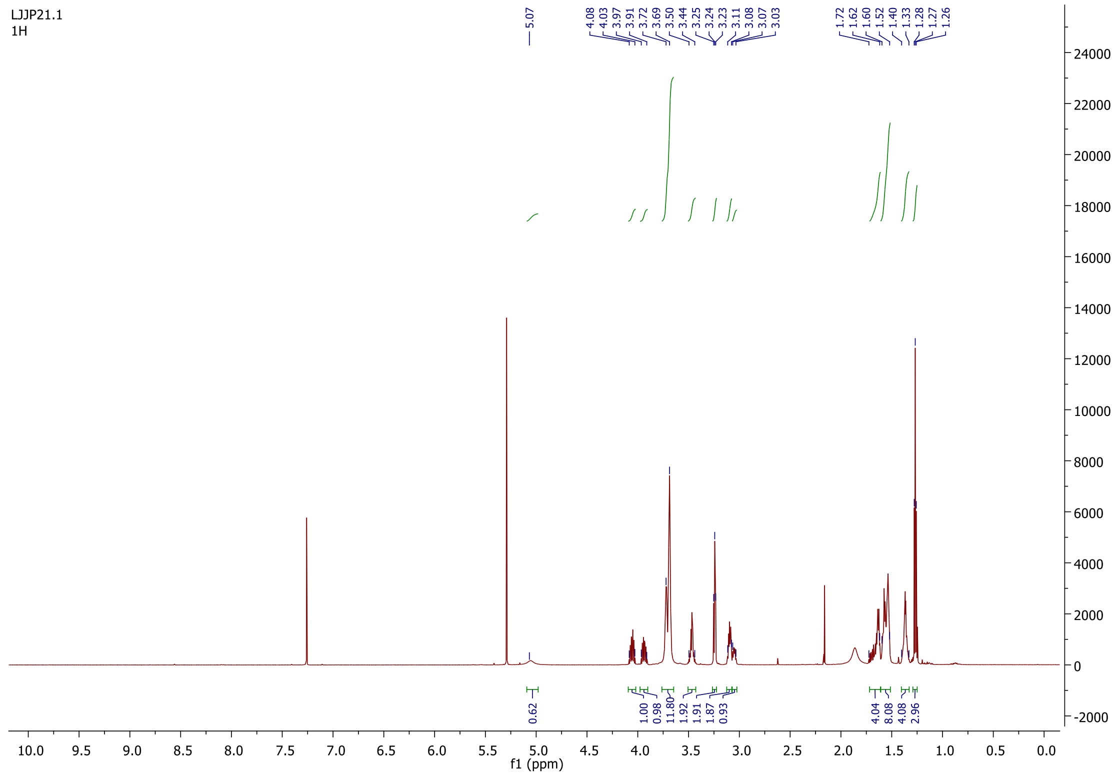

Fig. S149. ^{1}H NMR 8j ($CDCl_3$, 700 MHz).

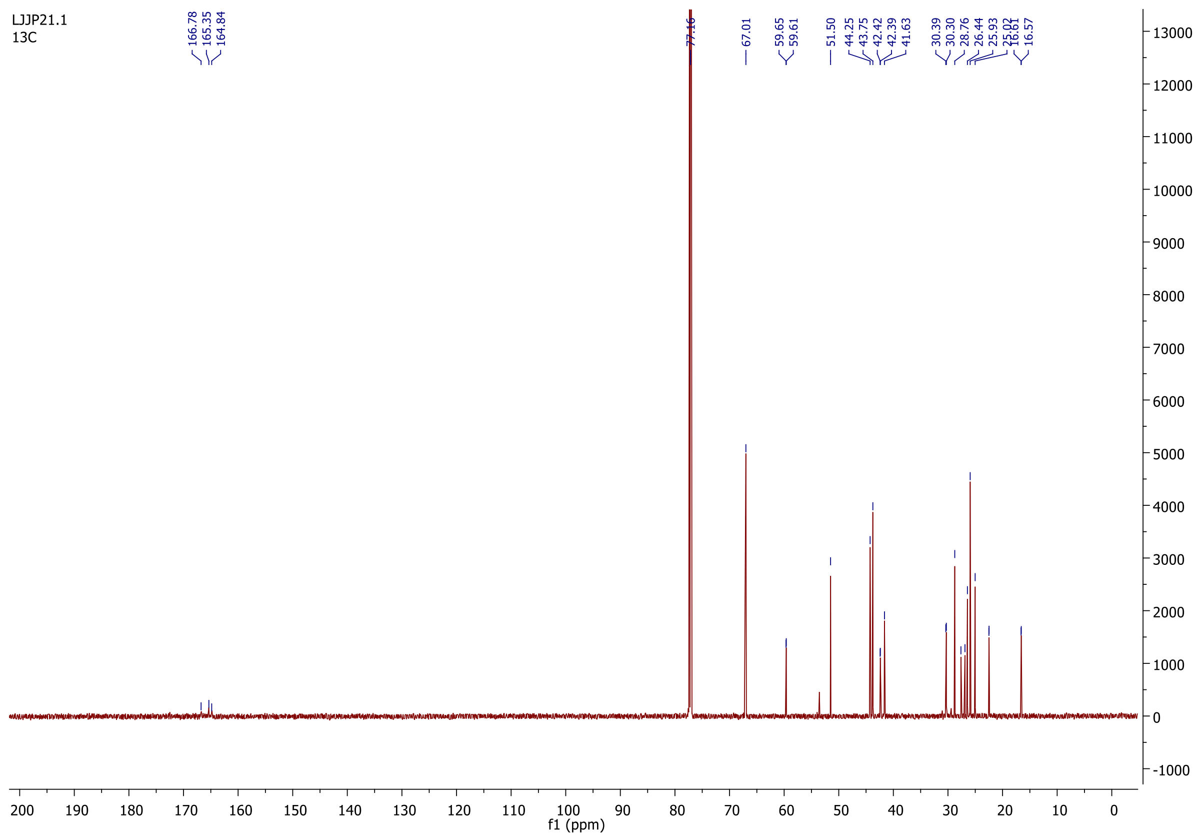

Fig. S150. ^{13}C NMR 8j ($CDCl_3$, 176 MHz).

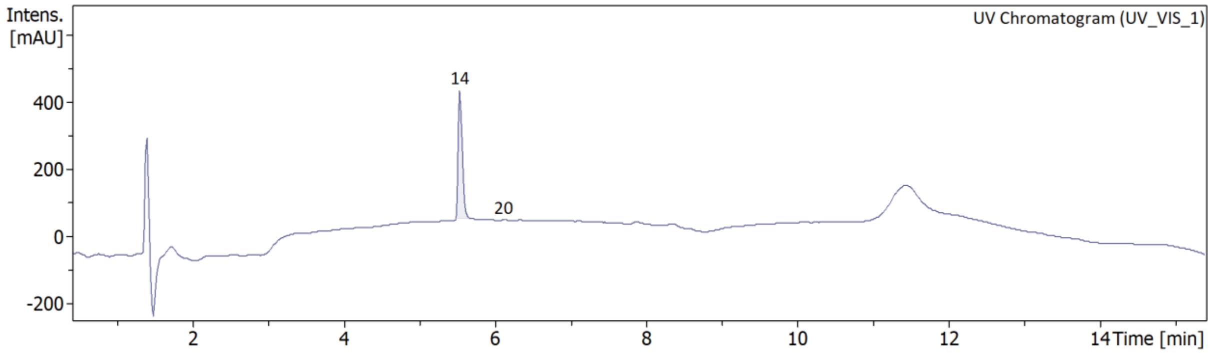

Fig. S151. UV-Vis Chromatogram 8j

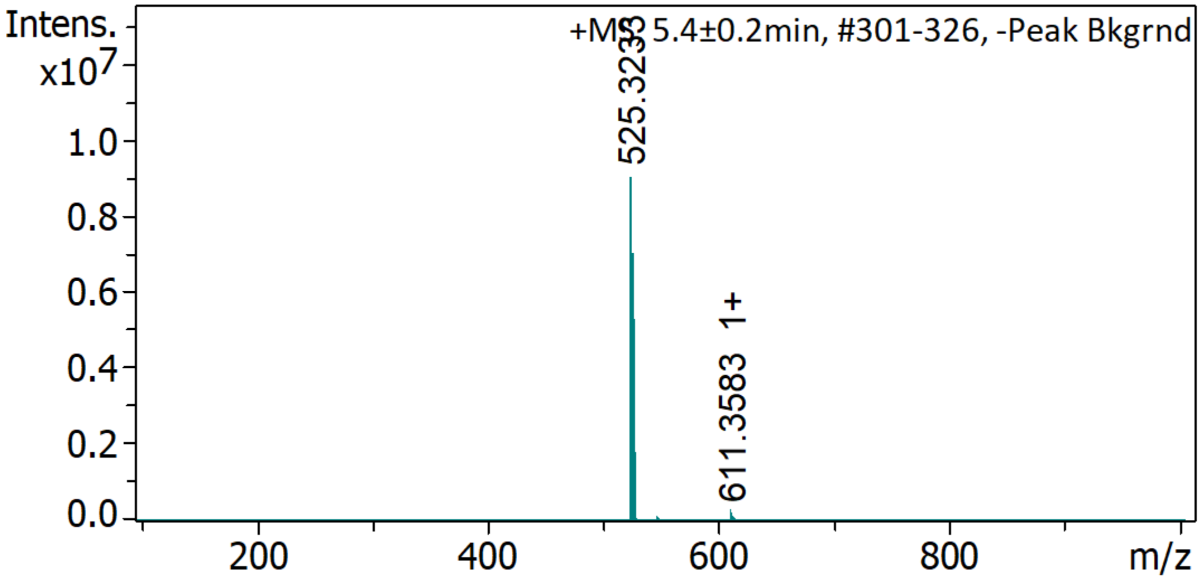

Fig. S152. Mass spectrum 8j

S47. Analytical spectra of 8k

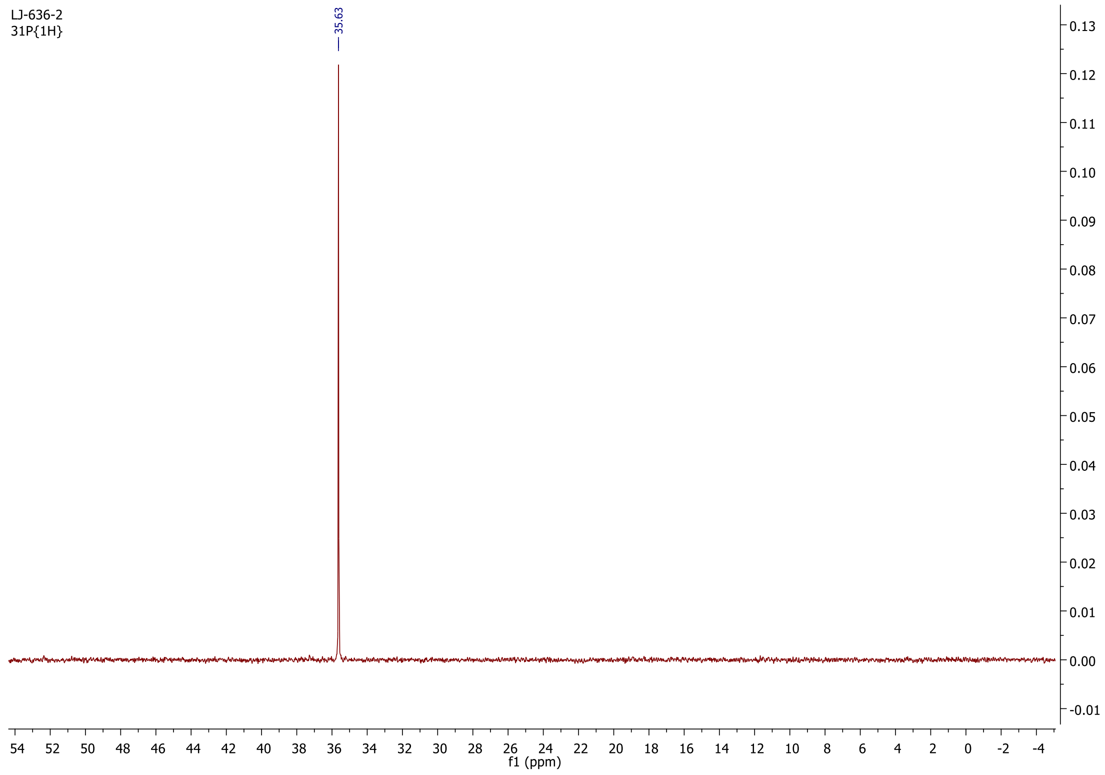

Fig. S153. ^{31}P NMR 8k ($CDCl_3$, 700 MHz).

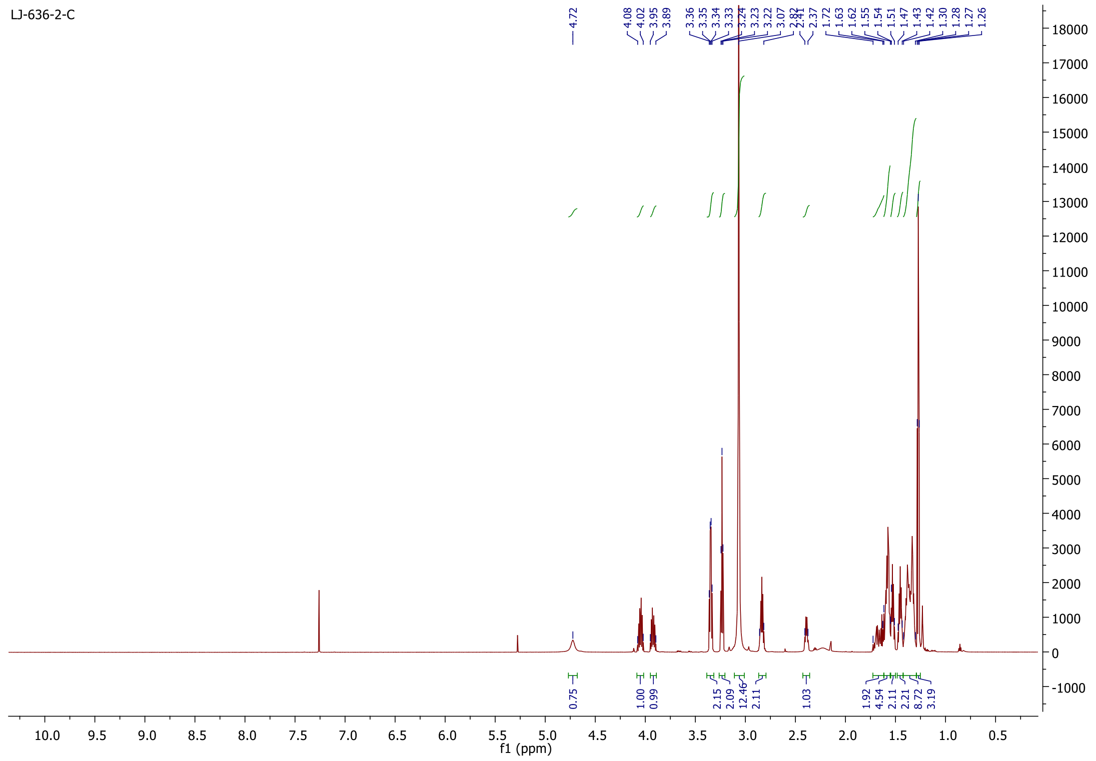

Fig. S154. ^{1}H NMR 8k ($CDCl_3$, 700 MHz).

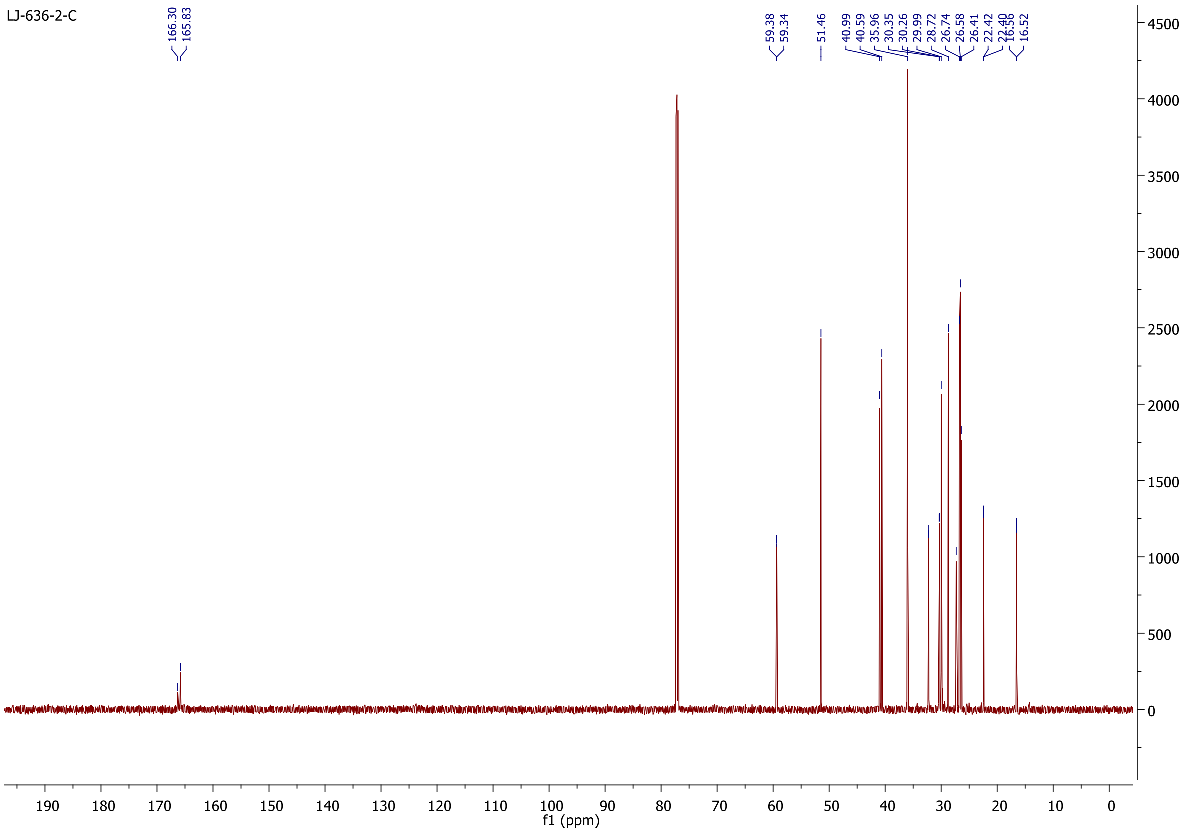

Fig. S155. ^{13}C NMR 8k ($CDCl_3$, 176 MHz).

S48. Analytical spectra of 1a

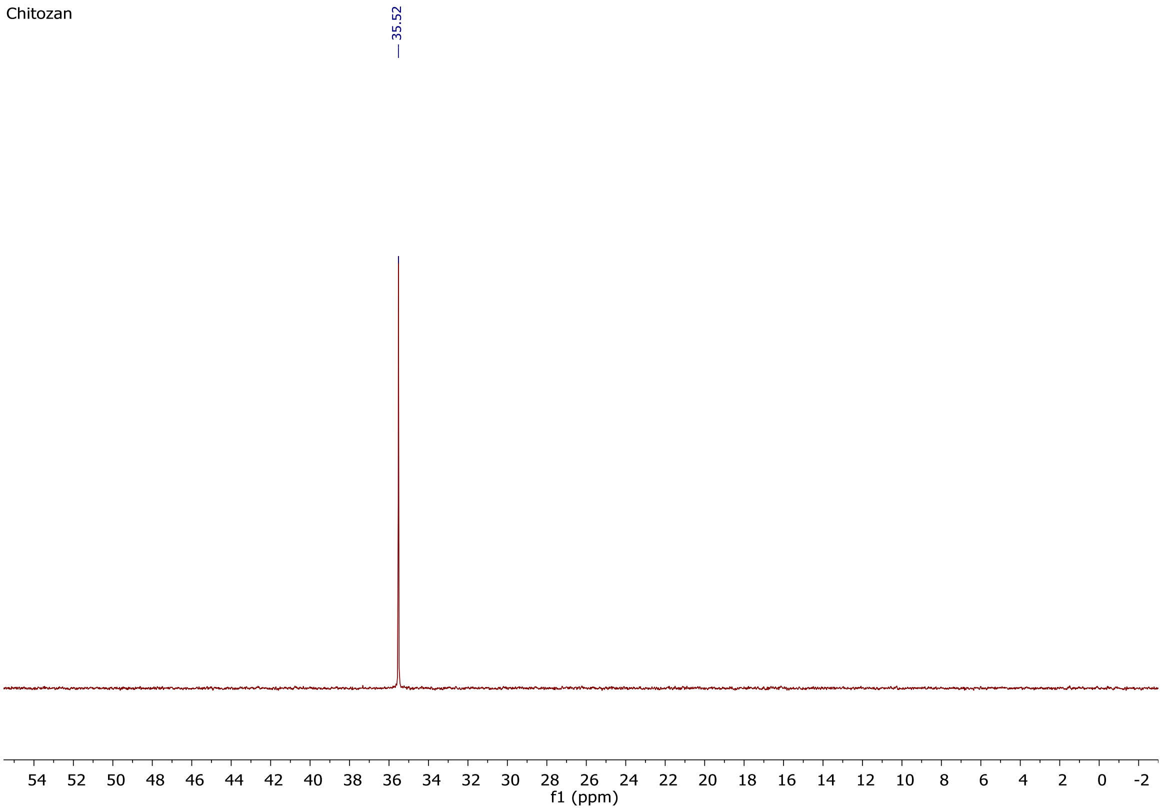

Fig. S156. ^{31}P NMR 1a ($CDCl_3$, 700 MHz).

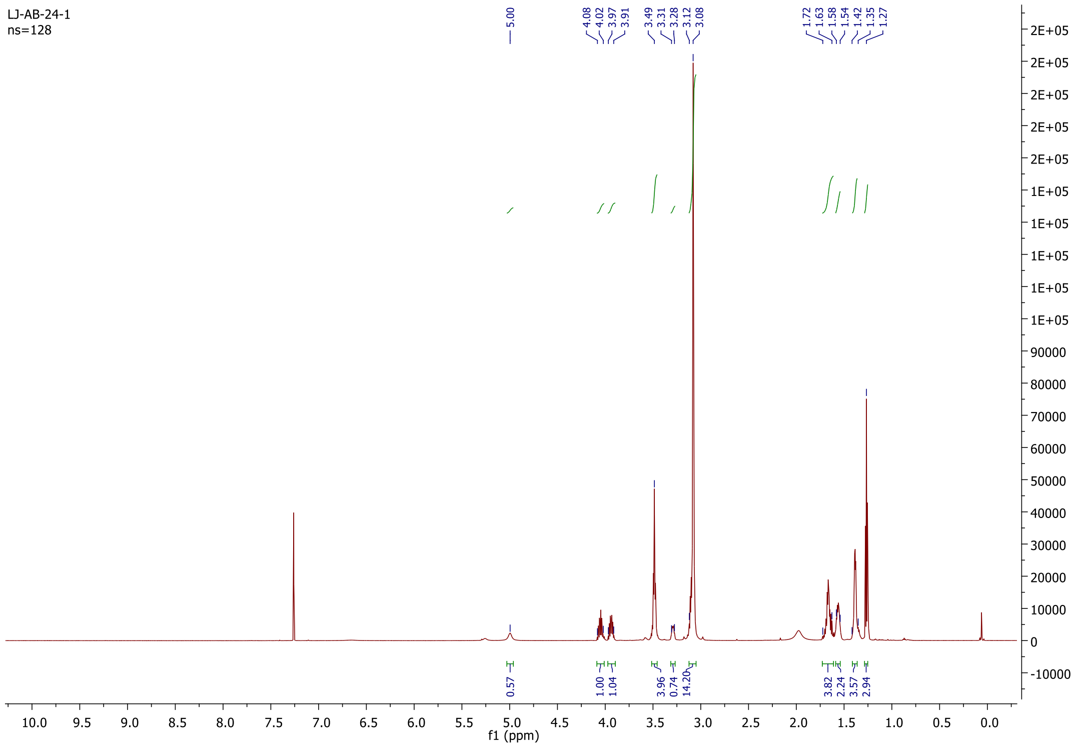

Fig. S157. ^{1}H NMR 1a ($CDCl_3$, 700 MHz).

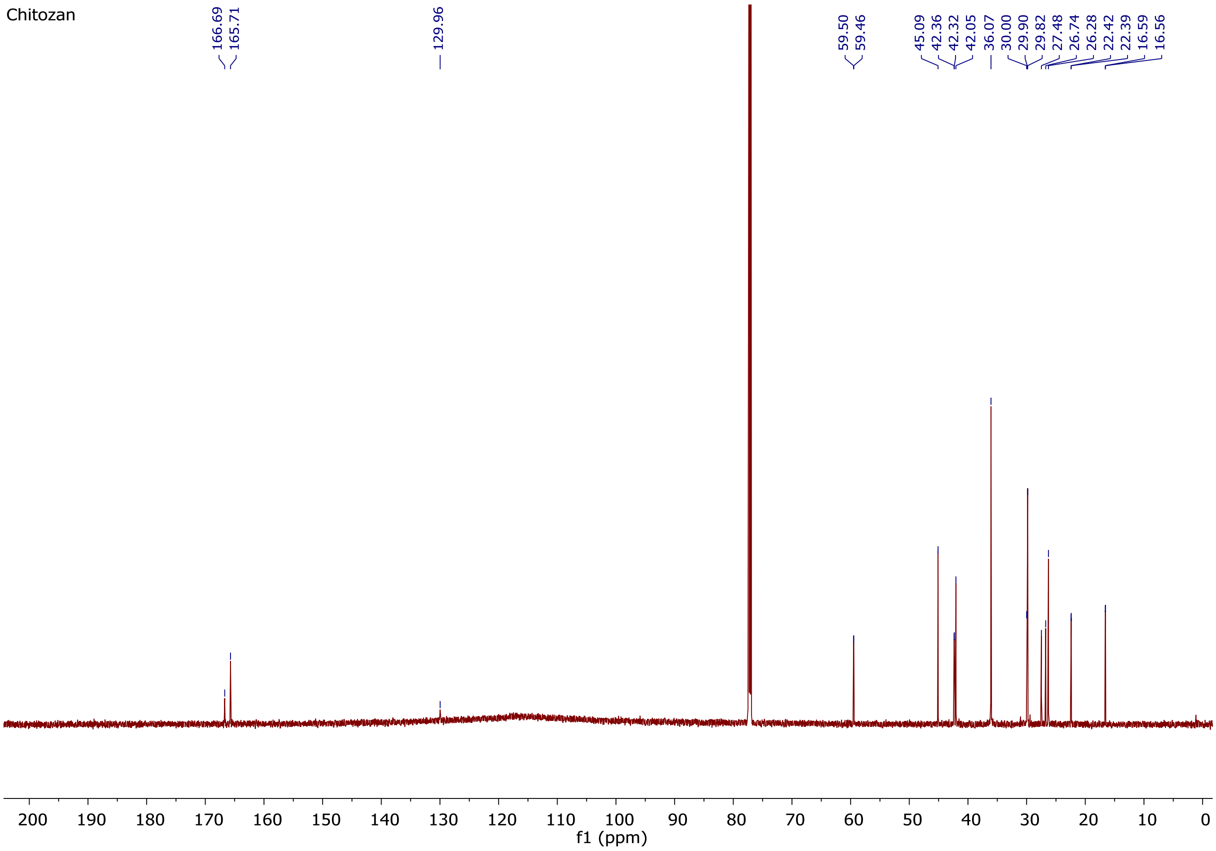

Fig. S158. ^{13}C NMR 1a ($CDCl_3$, 176 MHz).

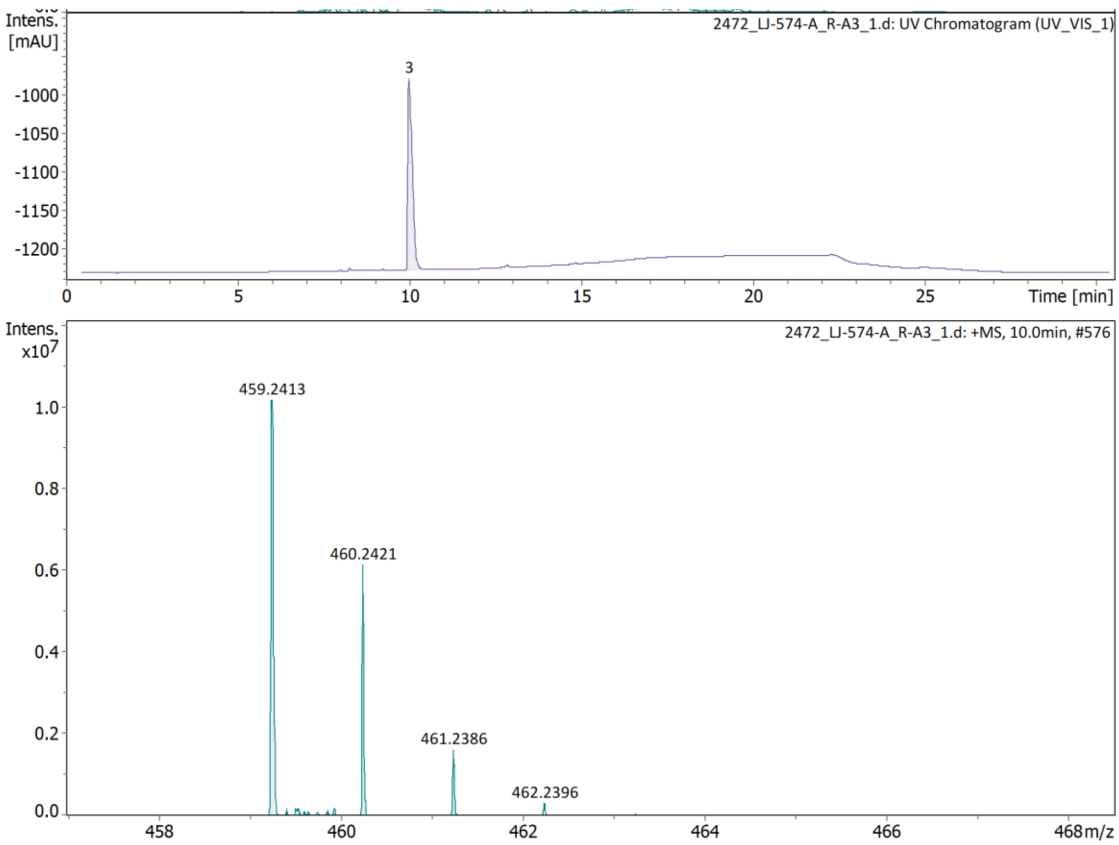

Fig. S159. UV-Vis Chromatogram and LC-MS 1a

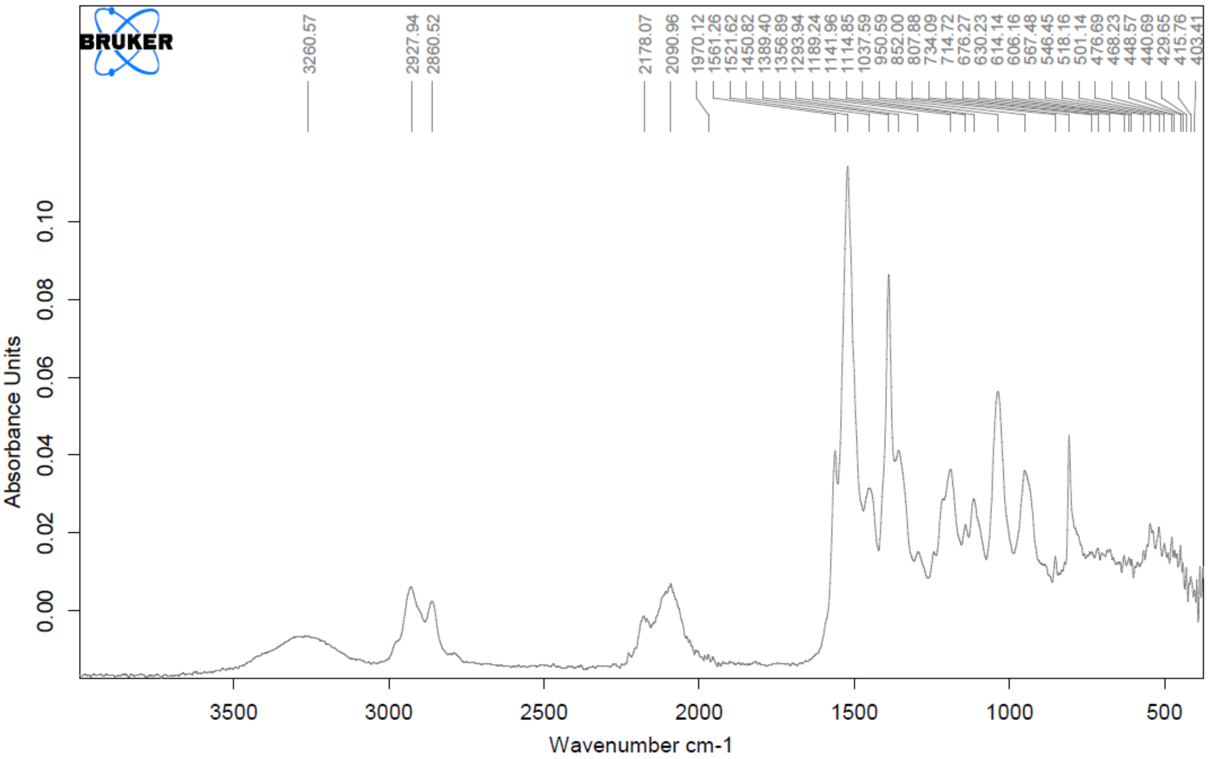

Fig. S160. IR spectra 1a

S49. Analytical spectra of 1b

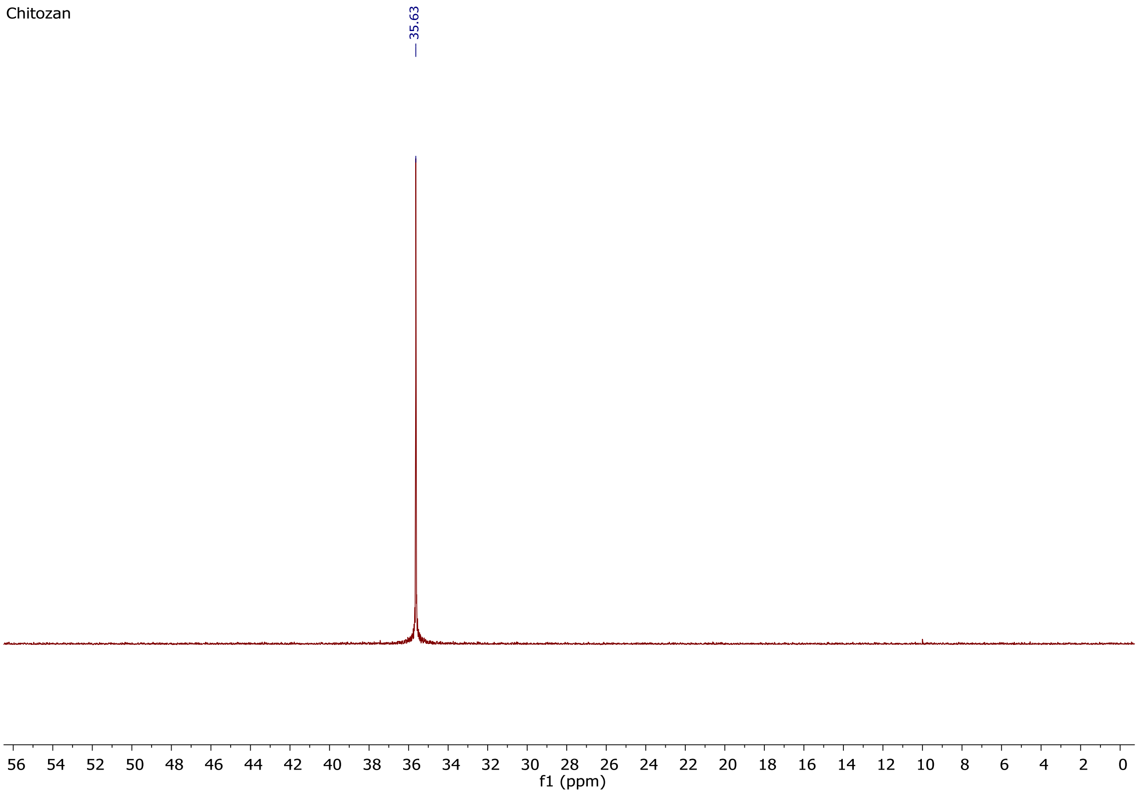

Fig. S161. ^{31}P NMR 1b ($CDCl_3$, 700 MHz).

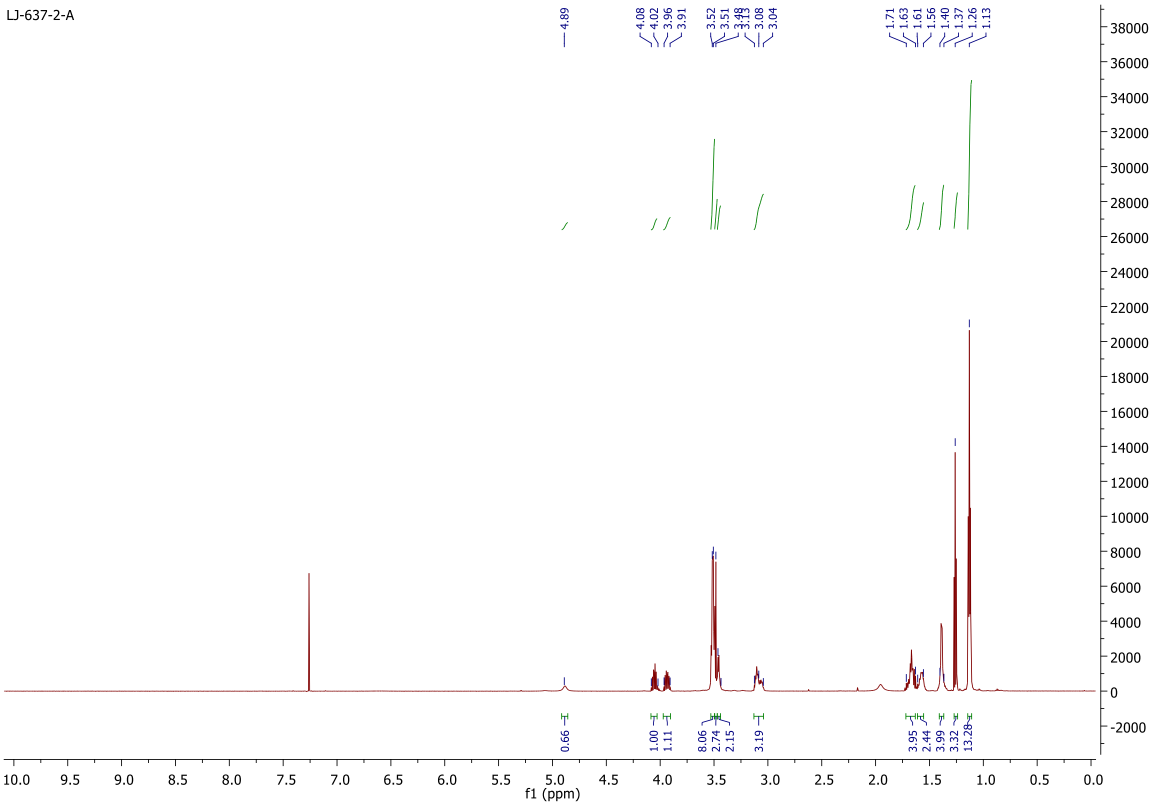

Fig. S162. ^{1}H NMR 1b ($CDCl_3$, 700 MHz).

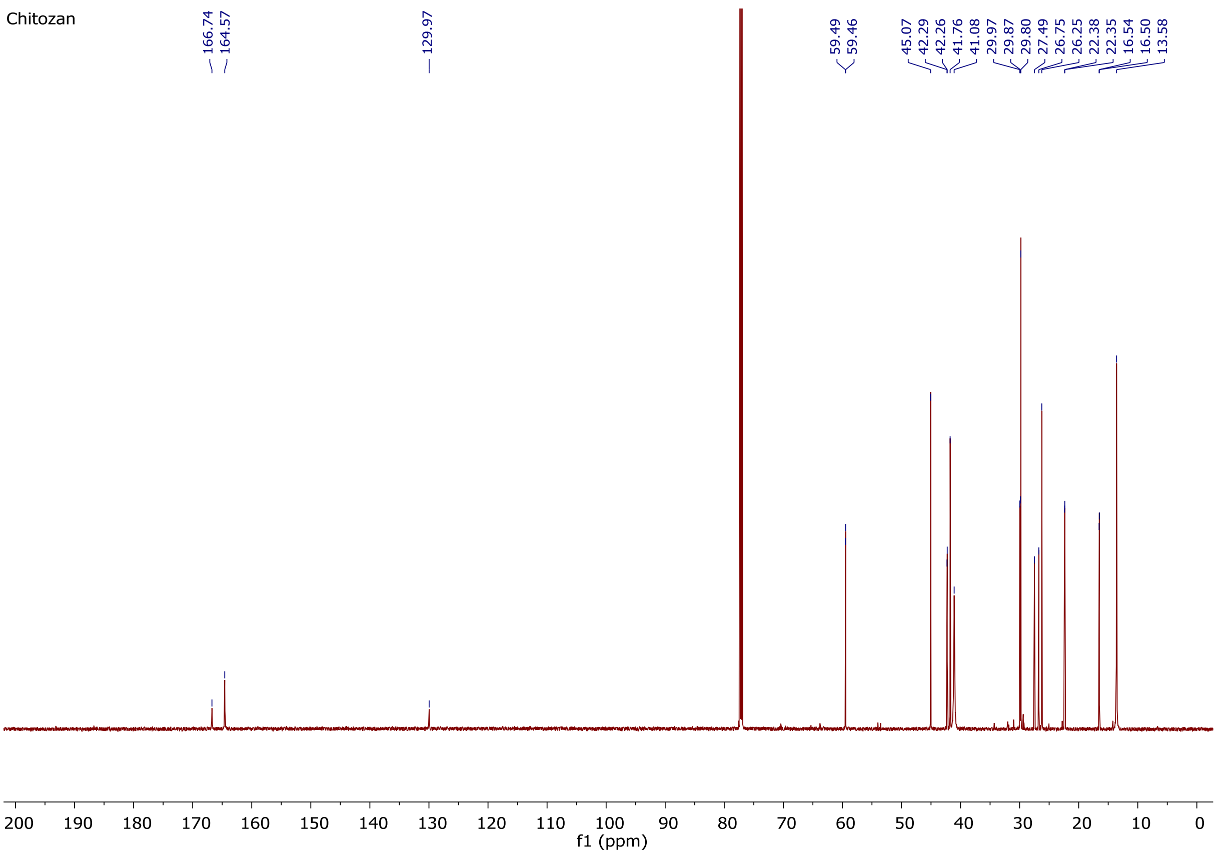

Fig. S163. ^{13}C NMR 1b ($CDCl_3$, 176 MHz).

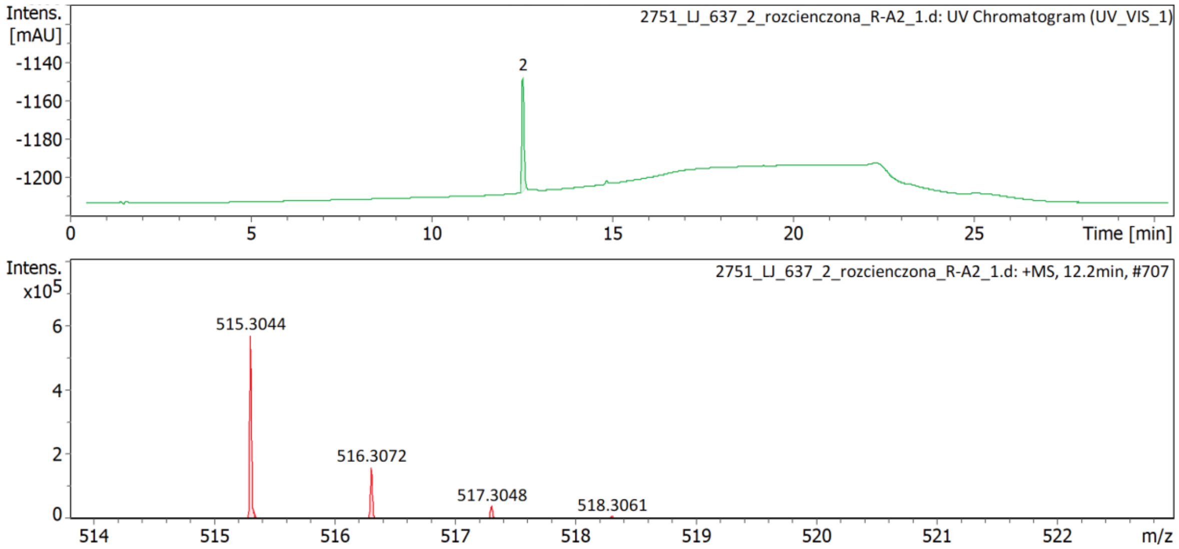

Fig. S164. UV-Vis Chromatogram and LC-MS 1b

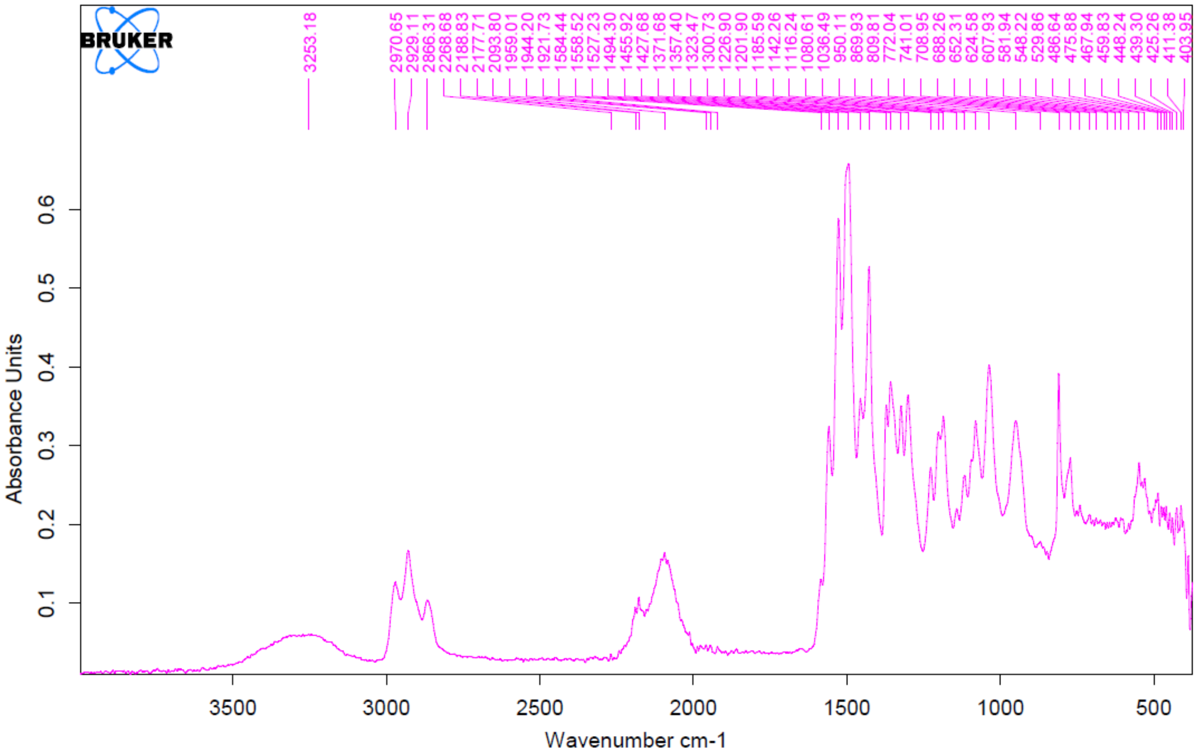

Fig. S165. IR spectra 1b

S50. Analytical spectra of 1c

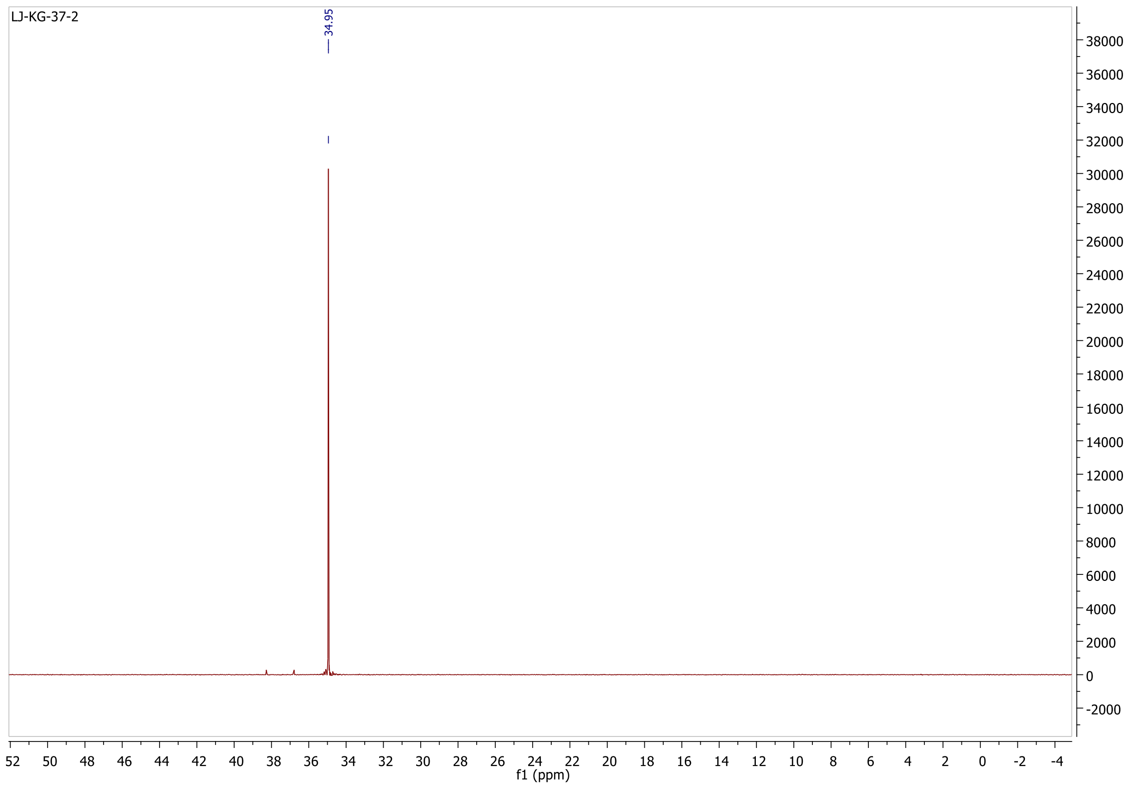

Fig. S166. ^{31}P NMR 1c ($CDCl_3$, 700 MHz).

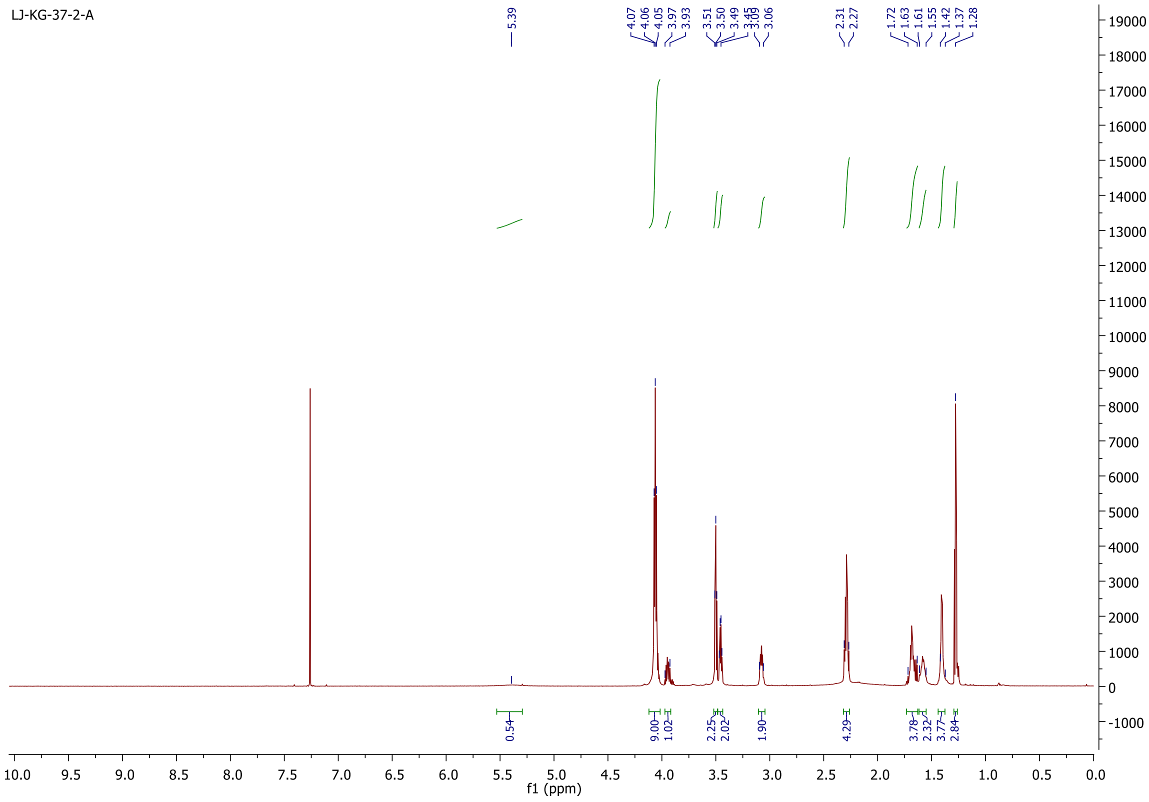

Fig. S167. ^{1}H NMR 1c ($CDCl_3$, 700 MHz).

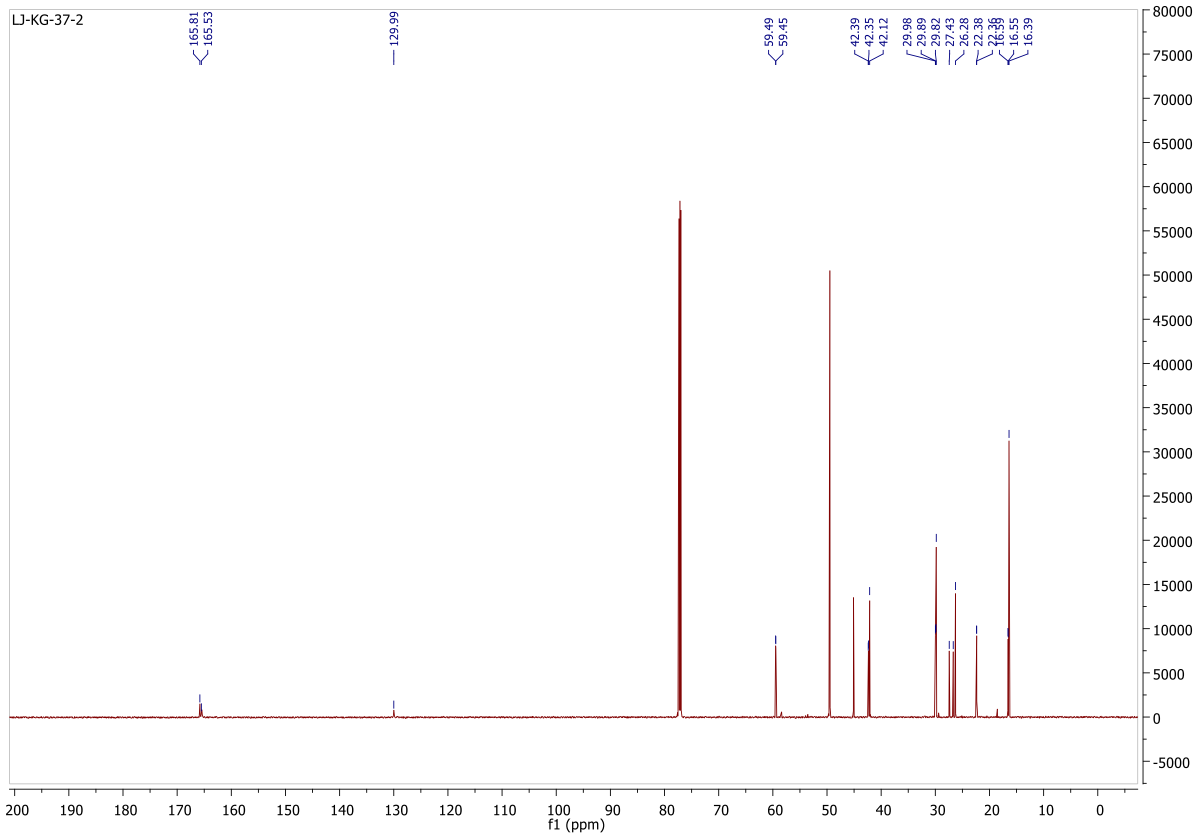

Fig. S168. ^{13}C NMR 1c ($CDCl_3$, 176 MHz).

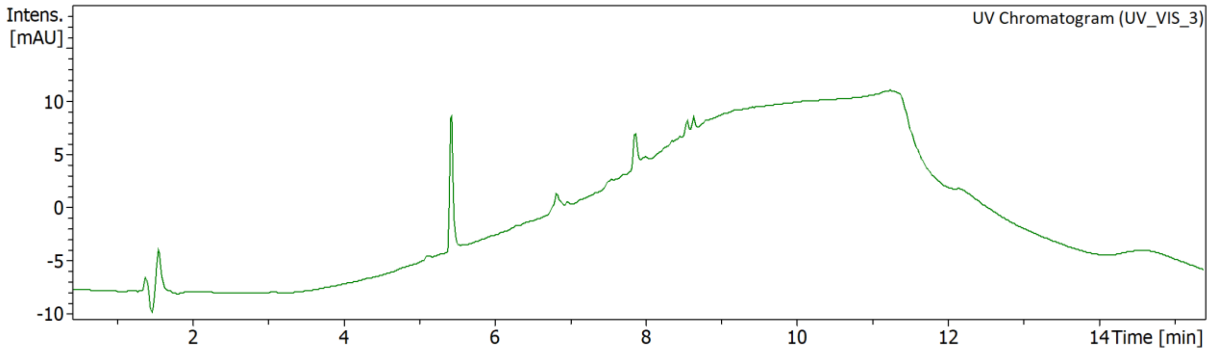

Fig. S169. UV-Vis Chromatogram 1c

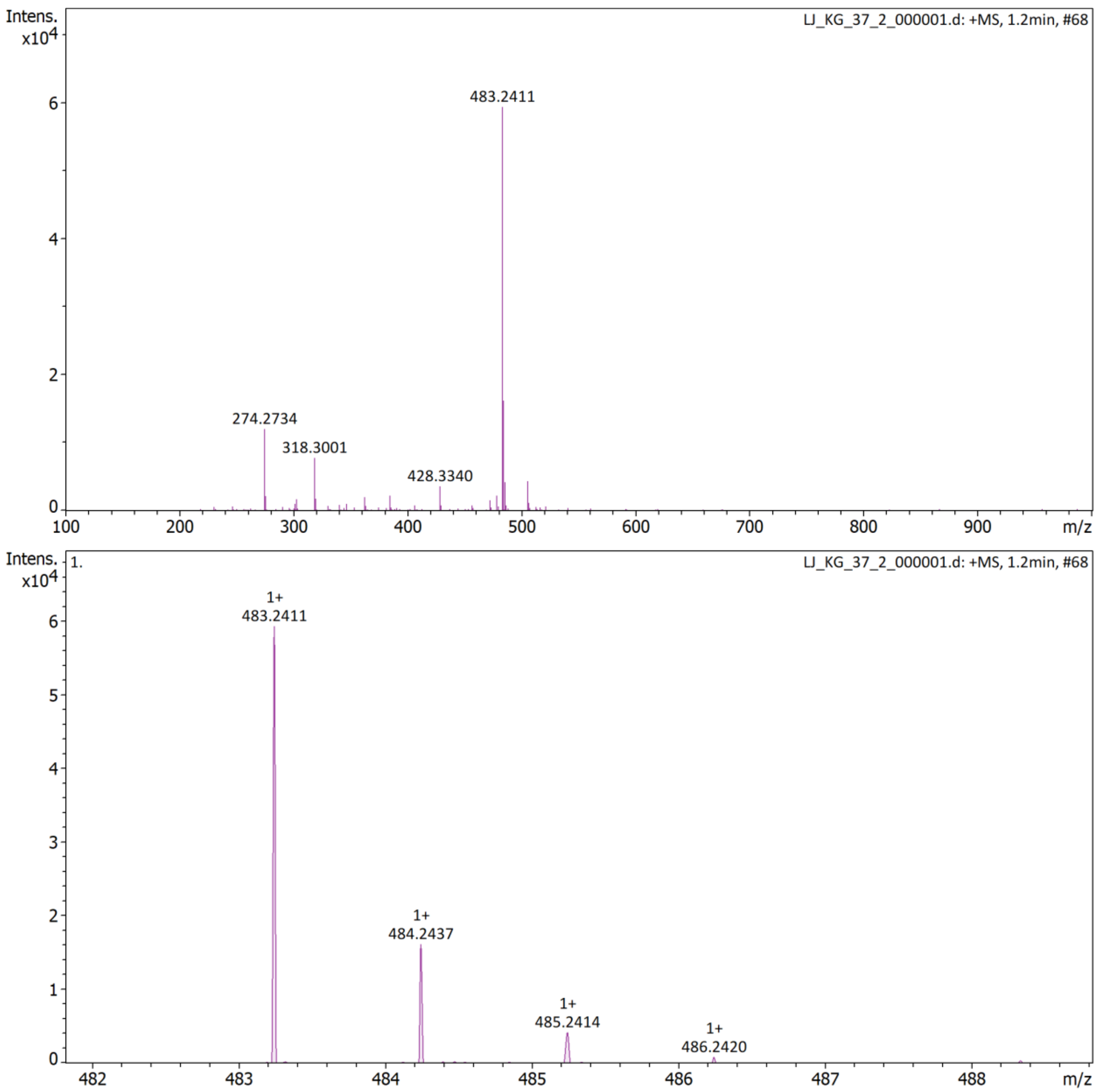

Fig. S170. Mass spectrum 1c

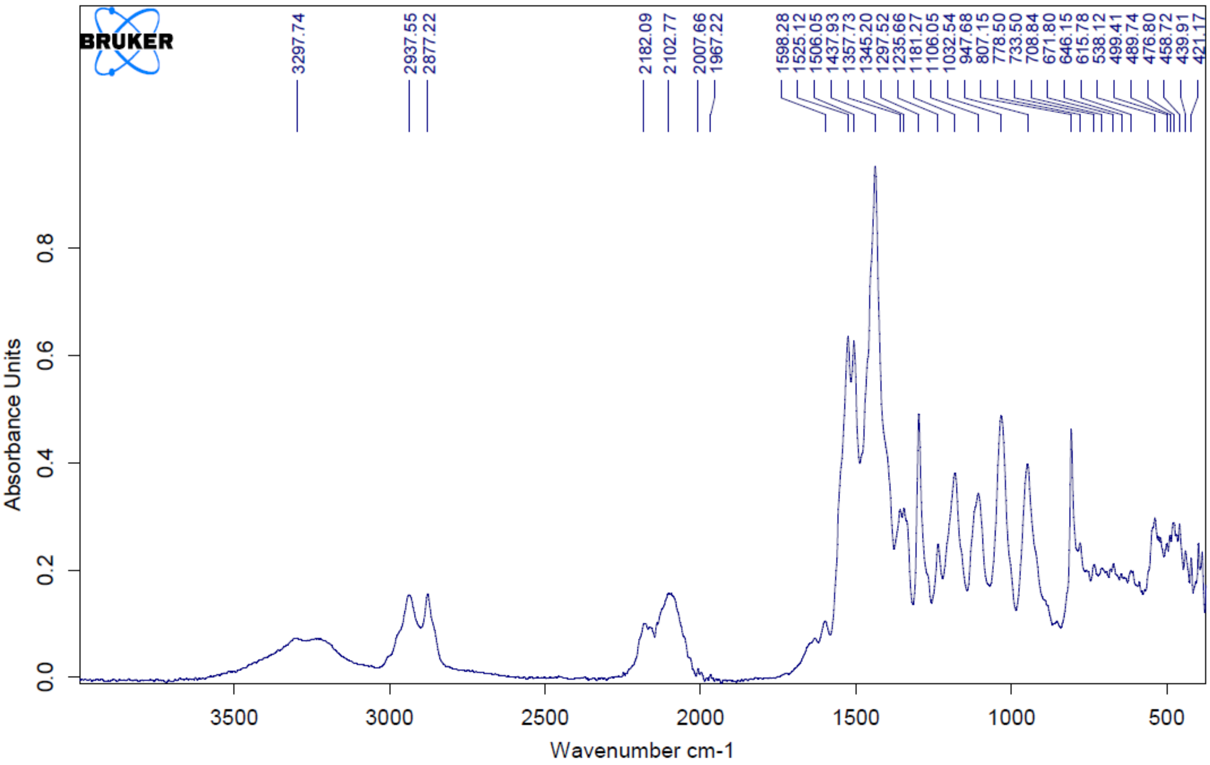

Fig. S171. IR spectra 1c

S51. Analytical spectra of 1d

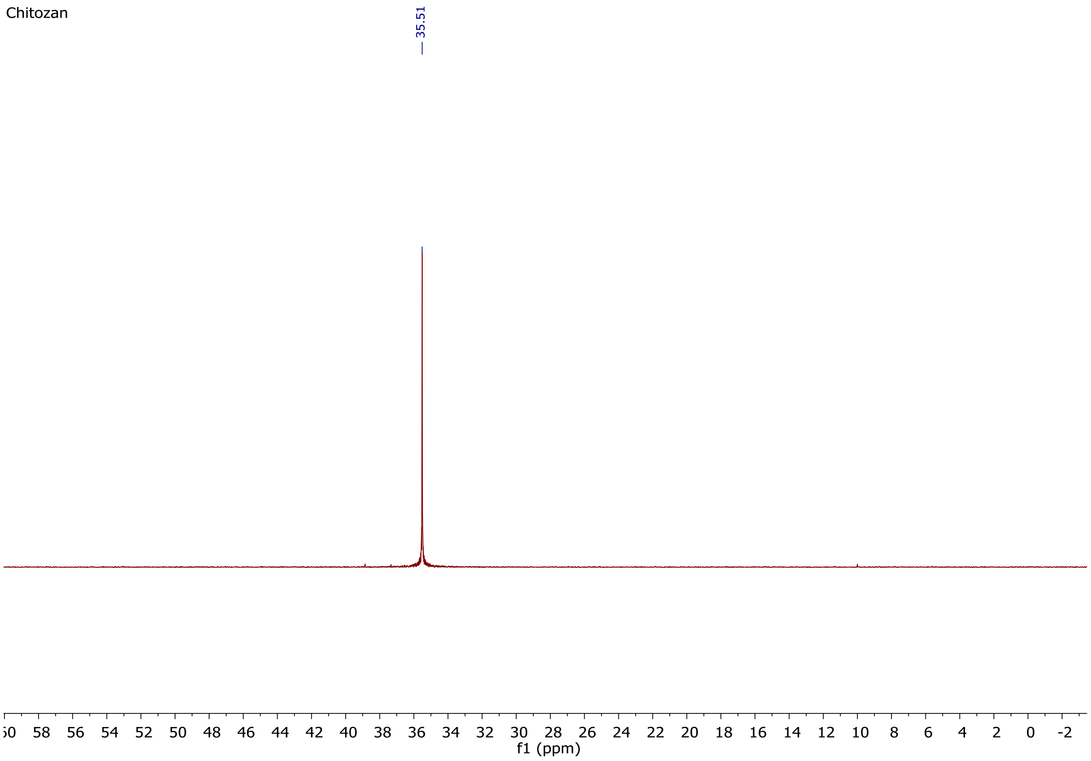

Fig. S172. ^{31}P NMR 1d ($CDCl_3$, 700 MHz).

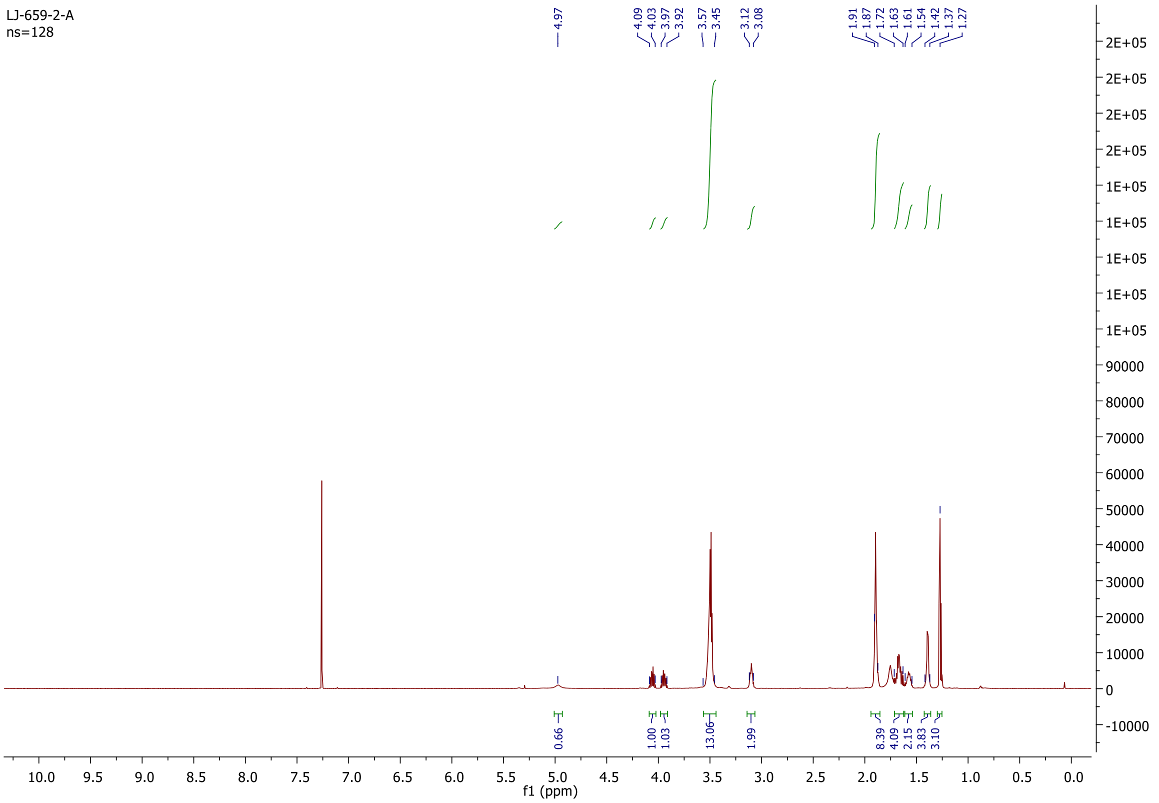

Fig. S173. ^{1}H NMR 1d ($CDCl_3$, 700 MHz).

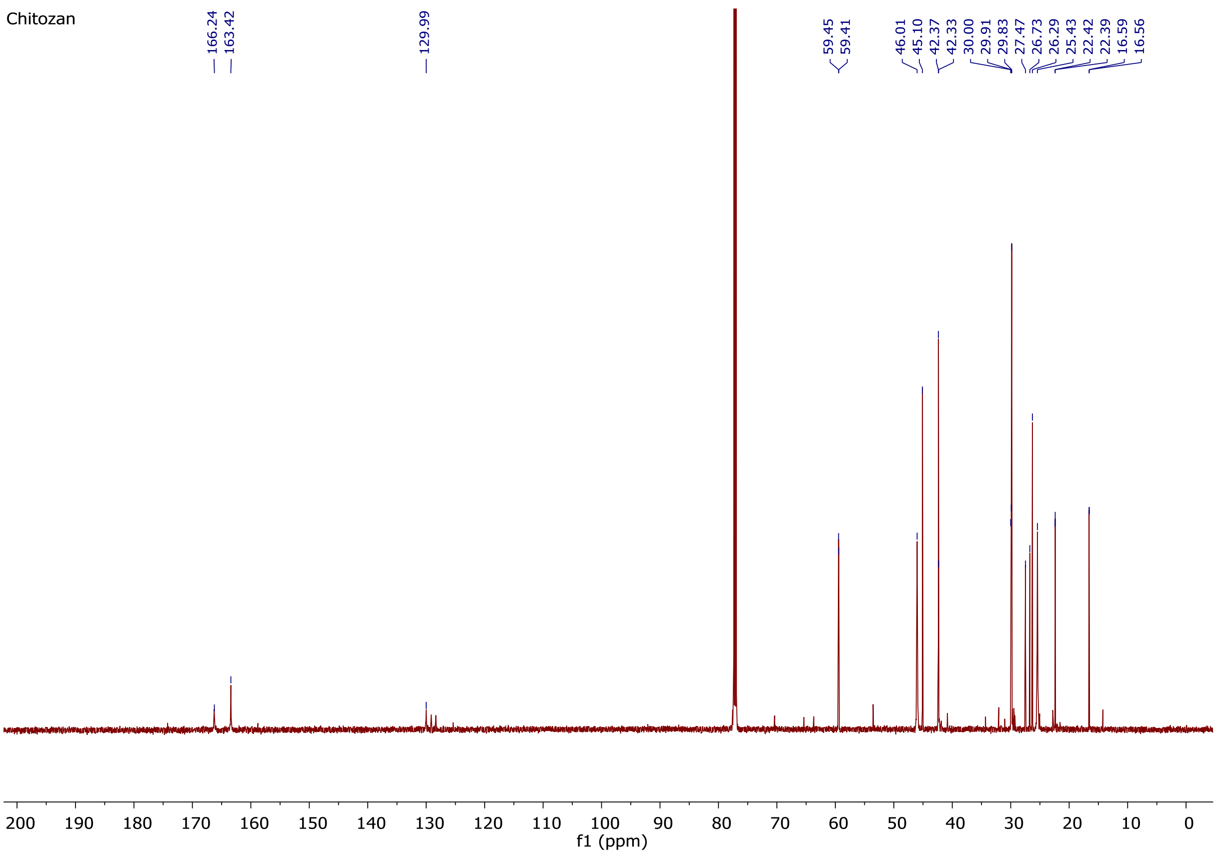

Fig. S174. ^{13}C NMR 1d ($CDCl_3$, 176 MHz).

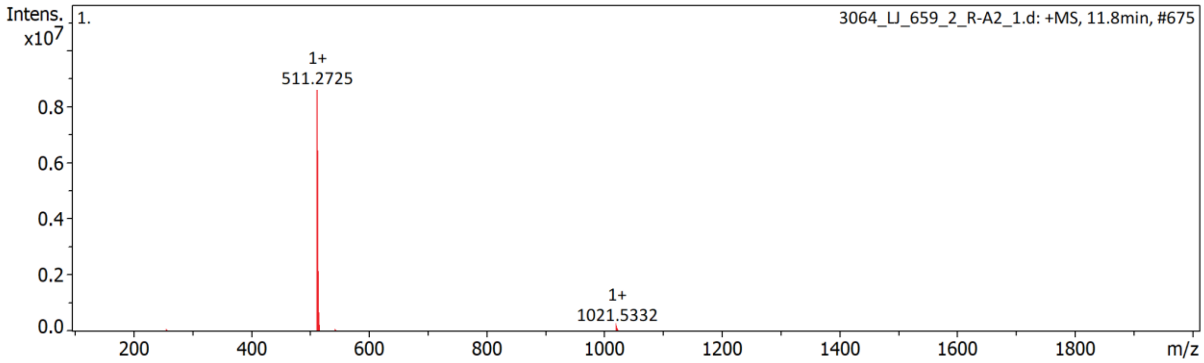

Fig. S175. LC-MS 1d

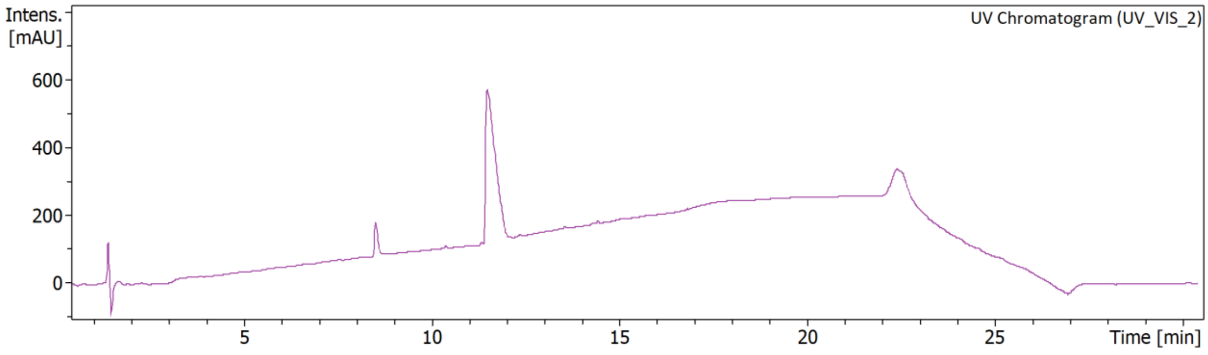

Fig. S176. UV-Vis Chromatogram 1d

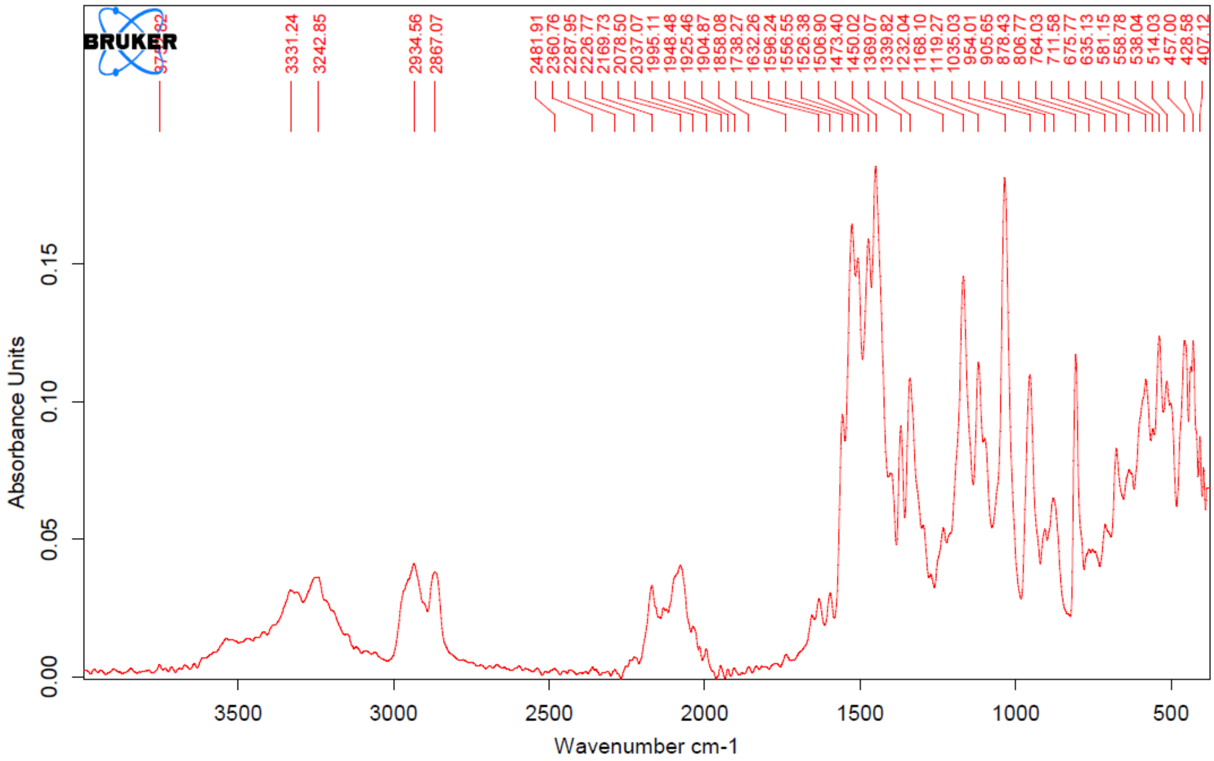

Fig. S177. IR spectra 1d

S52. Analytical spectra of 1e

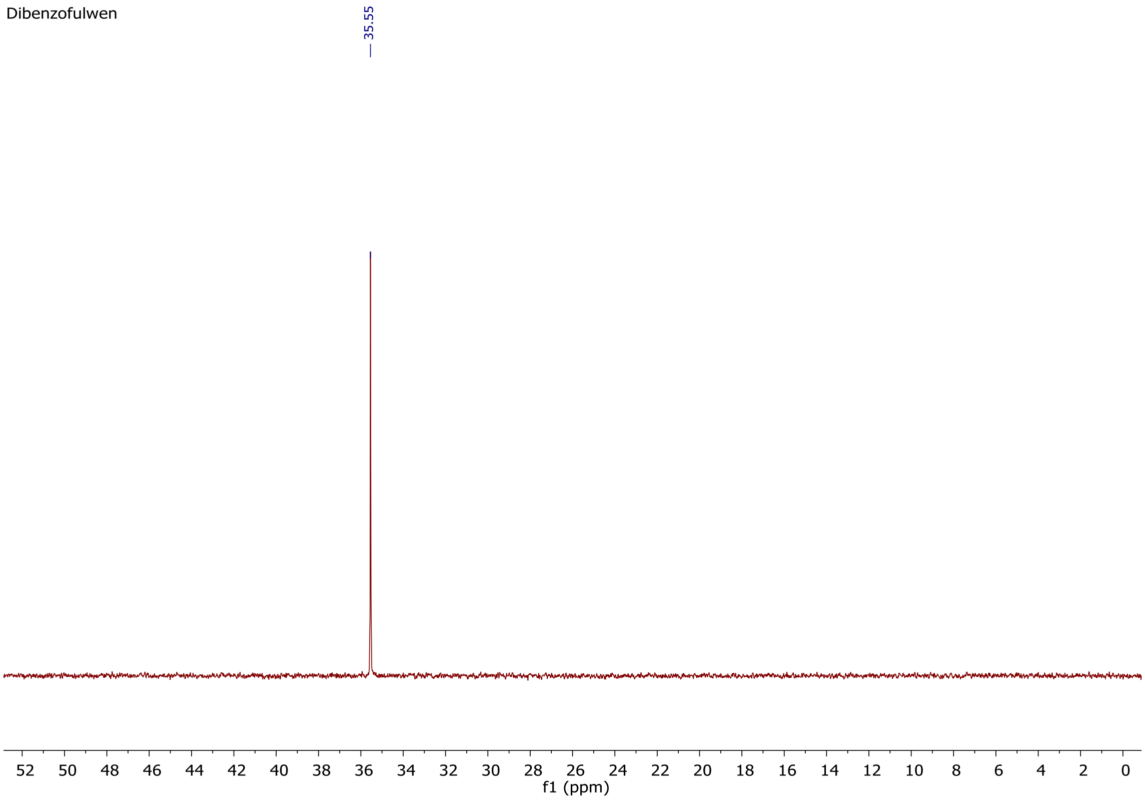

Fig. S178. ^{31}P NMR 1e ($CDCl_3$, 700 MHz).

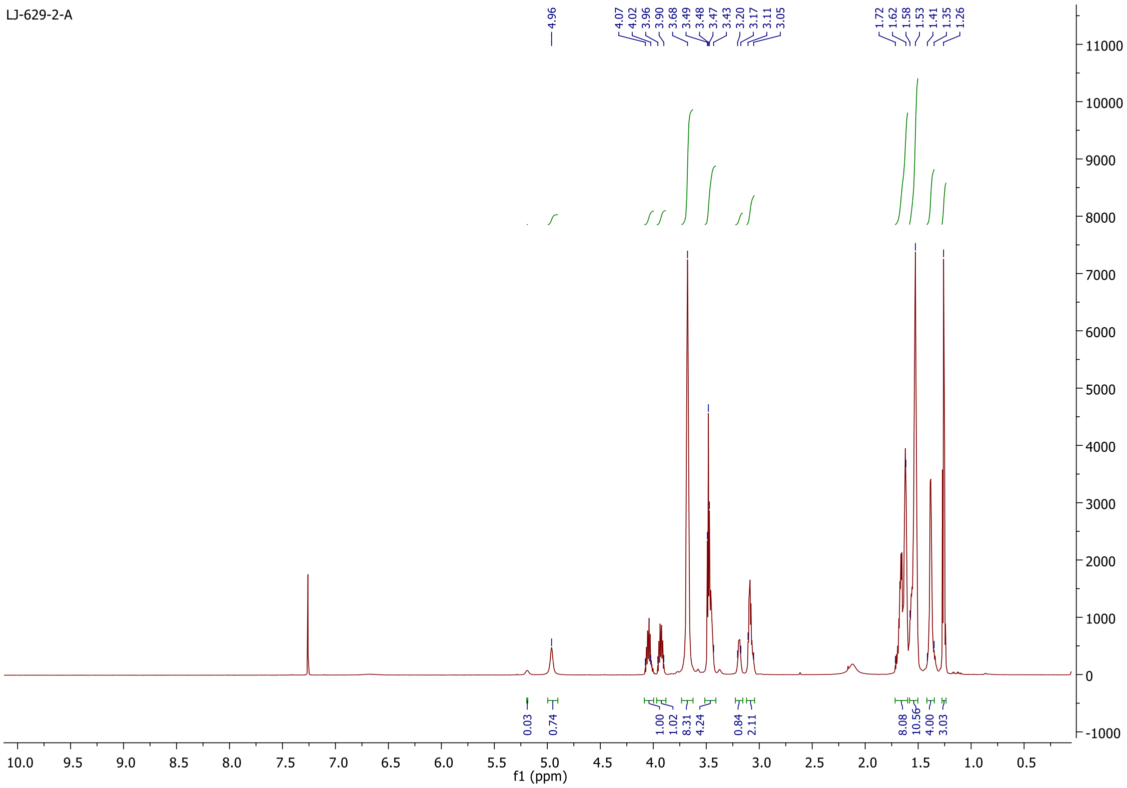

Fig. S179. ^{1}H NMR 1e ($CDCl_3$, 700 MHz).

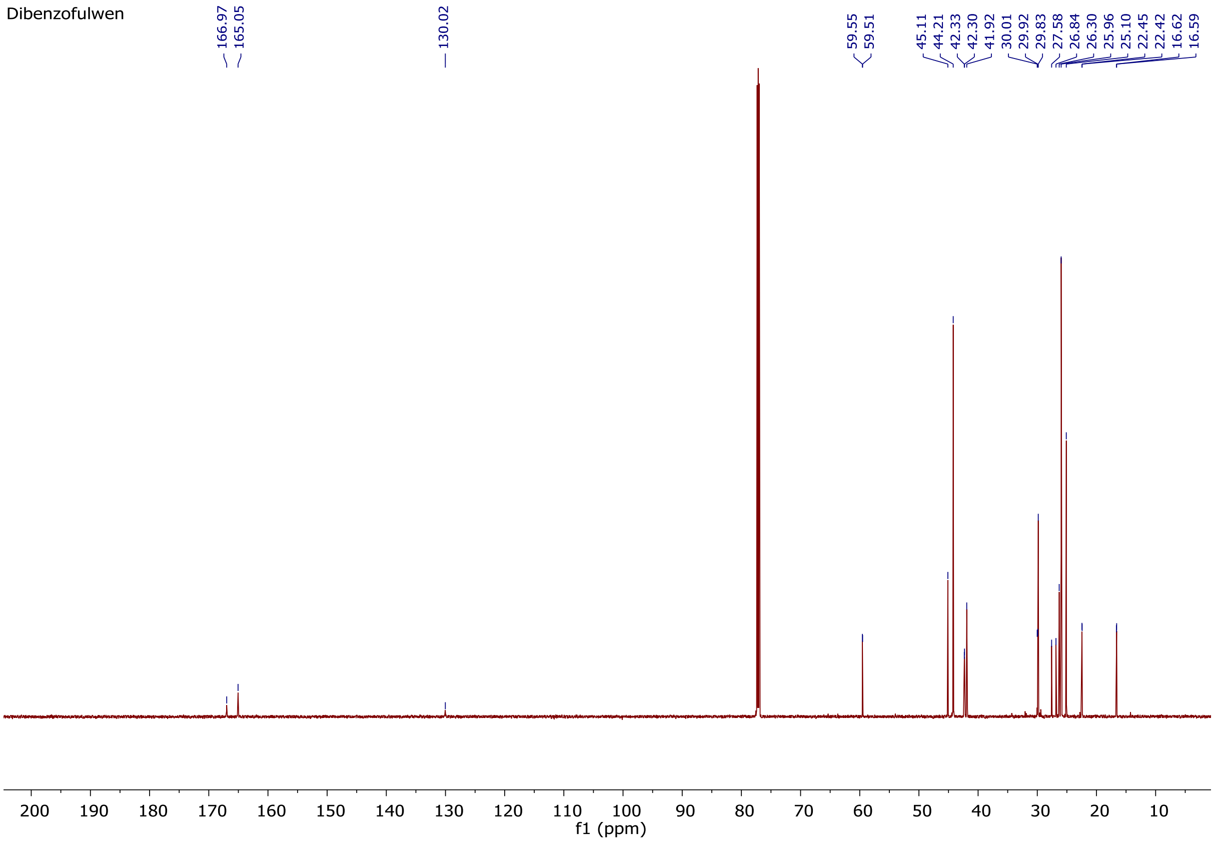

Fig. S180. ^{13}C NMR 1e ($CDCl_3$, 176 MHz).

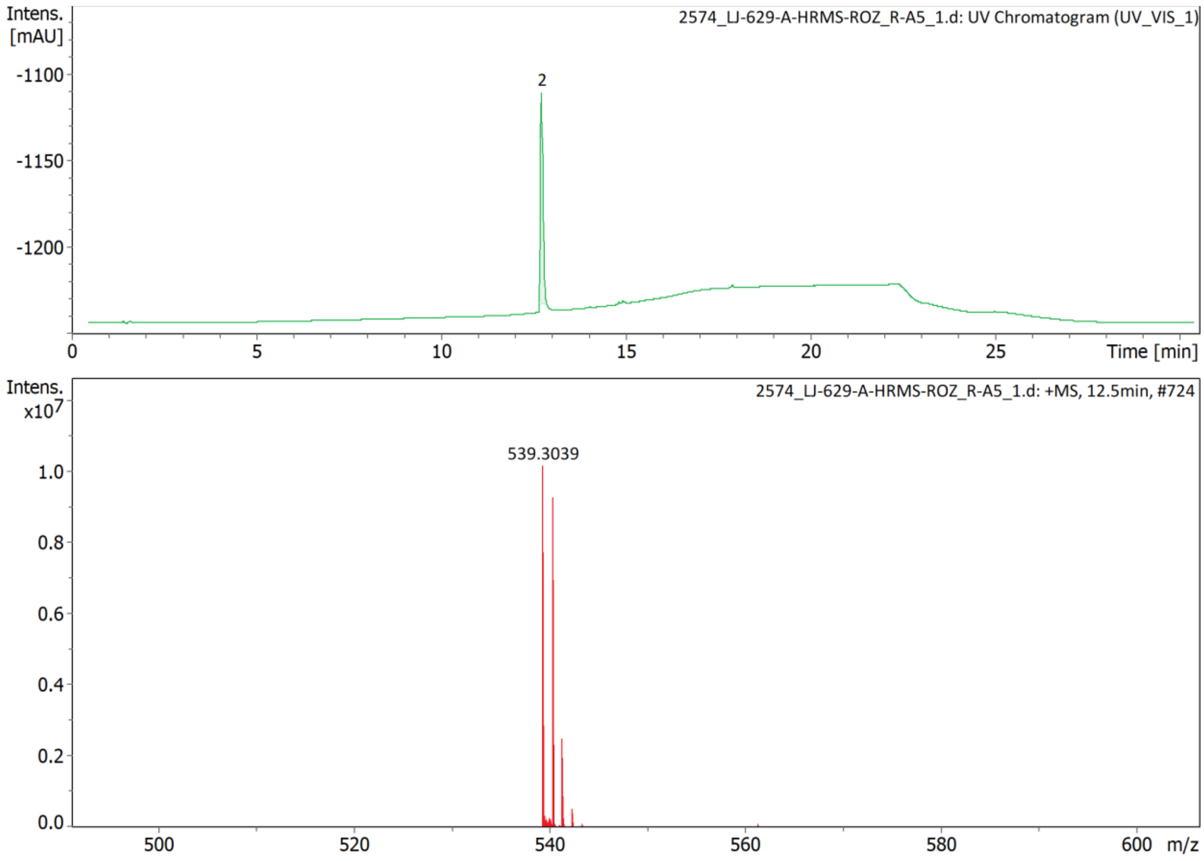

Fig. S181. UV-Vis Chromatogram and LC-MS 1e

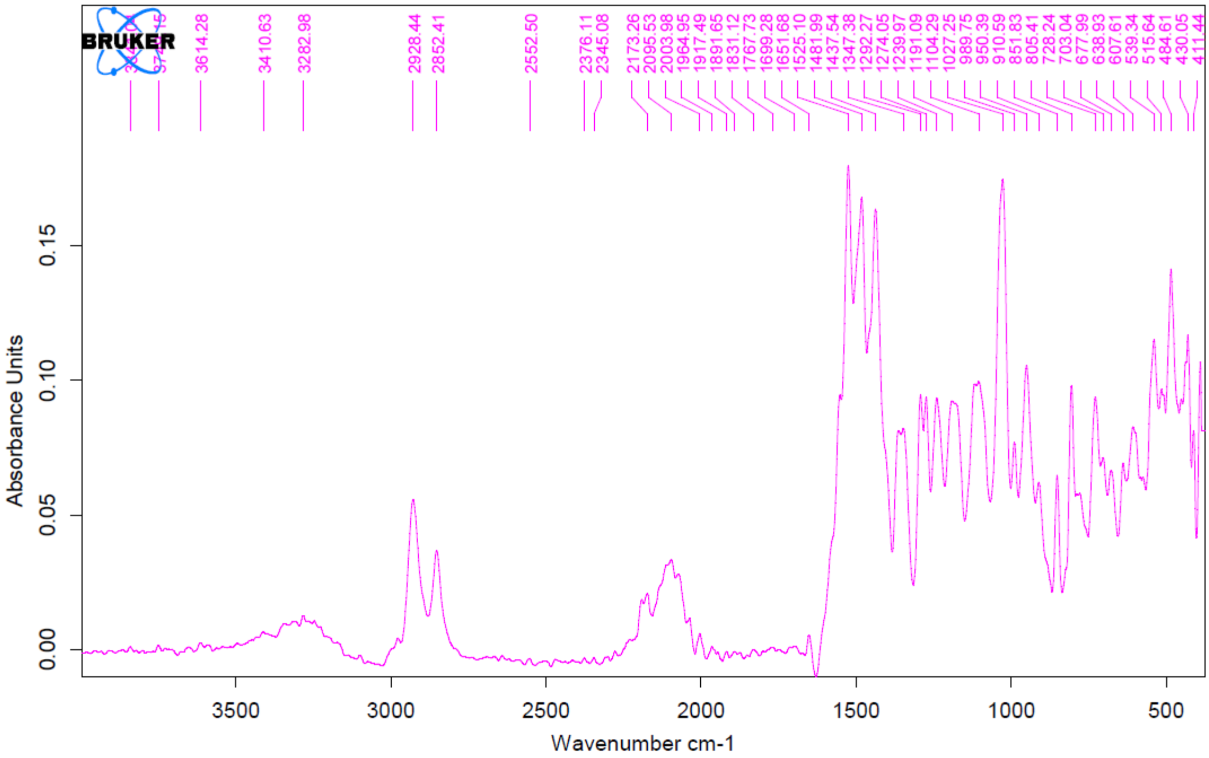

Fig. S182. IR spectra 1e

S53. Analytical spectra of 1f

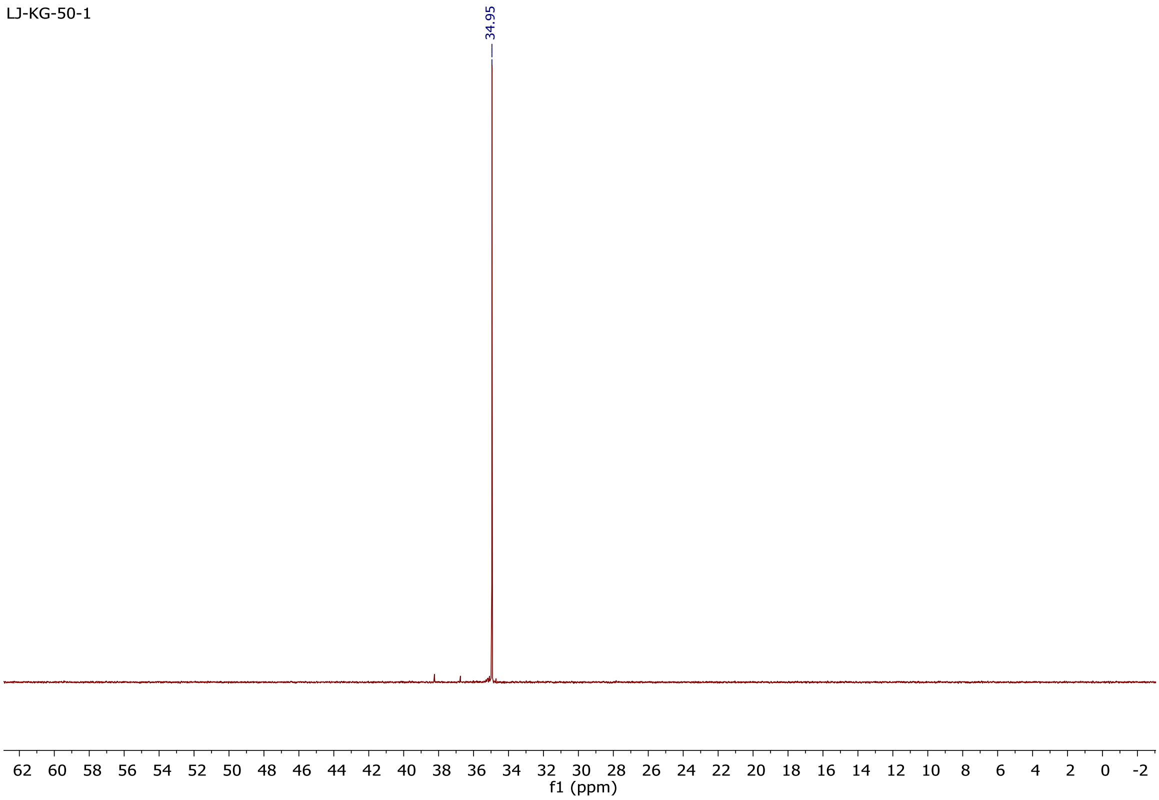

Fig. S183. ^{31}P NMR 1f (CDCl$_3$, 700 MHz).

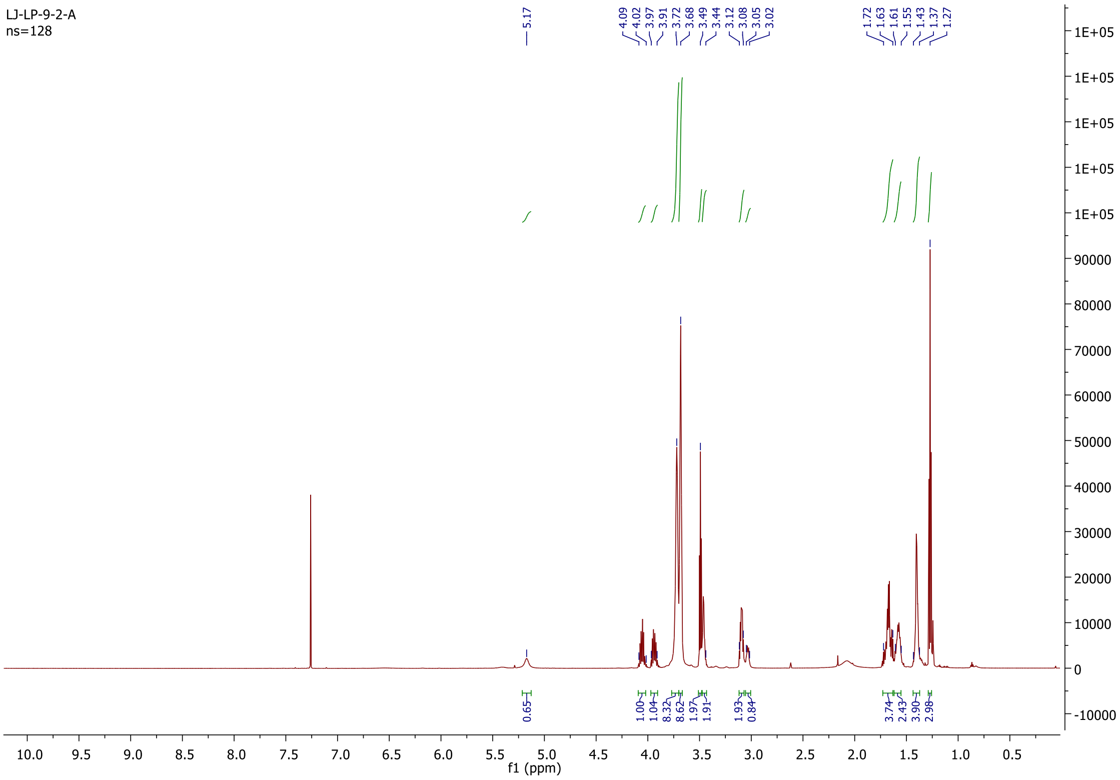

Fig. S184. ^{1}H NMR 1f (CDCl$_3$, 700 MHz).

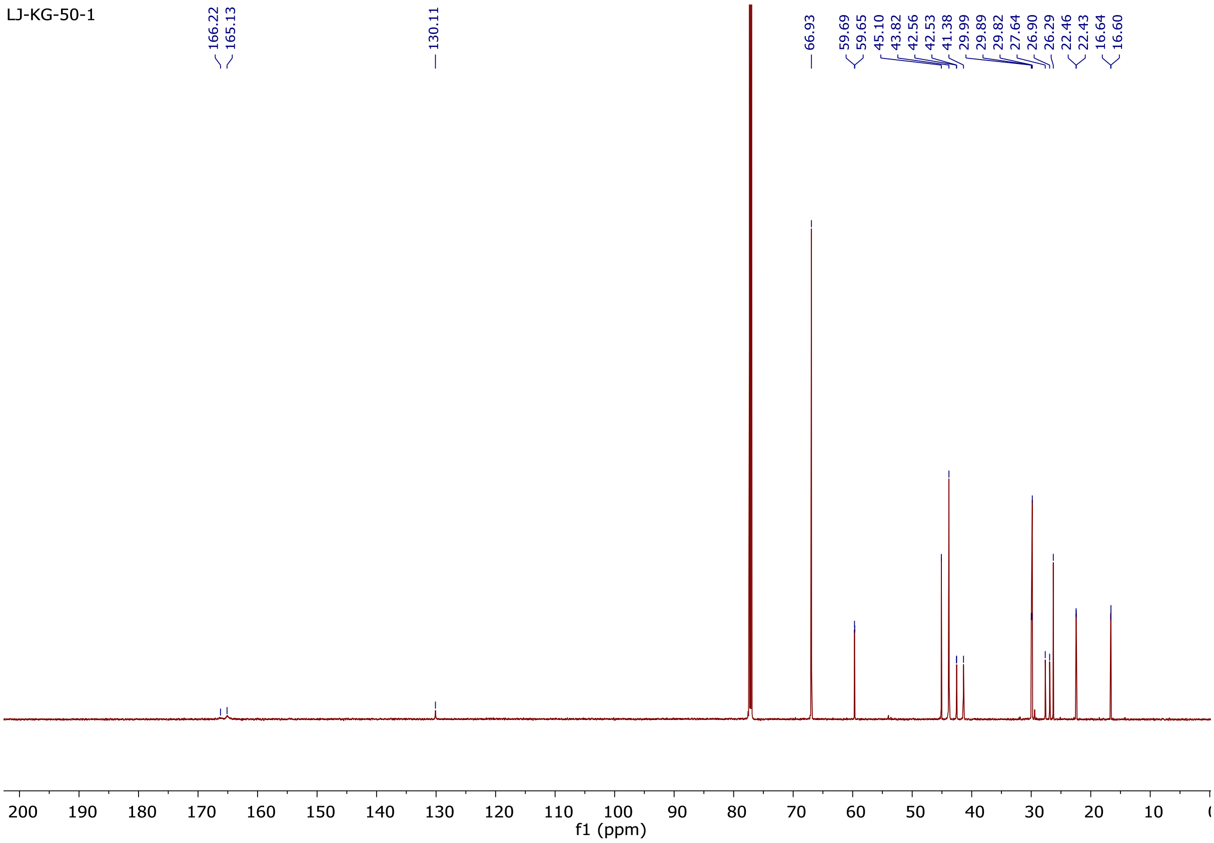

Fig. S185. ^{13}C NMR 1f ($CDCl_3$, 176 MHz).

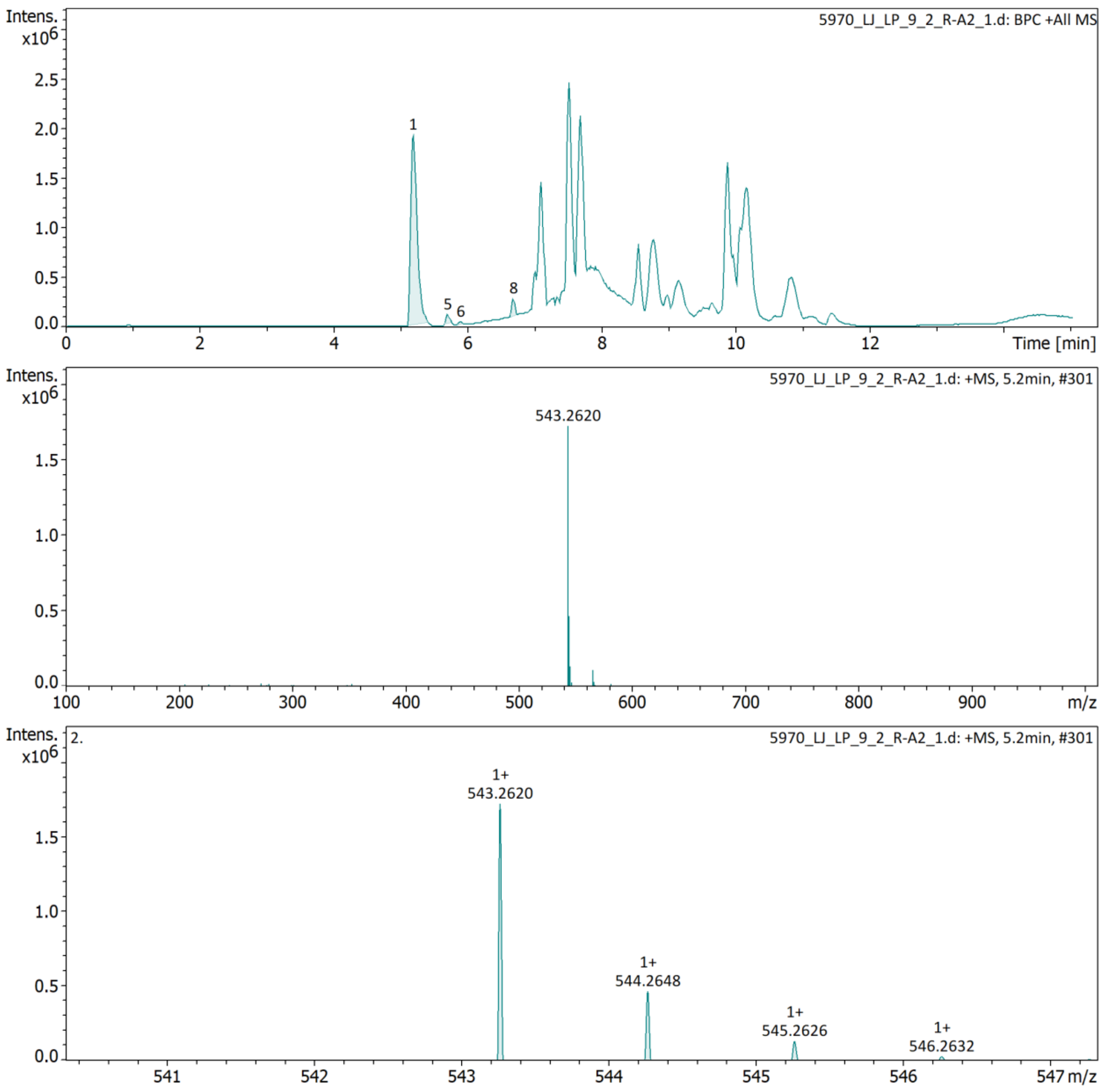

Fig. S186. LC-MS 1f

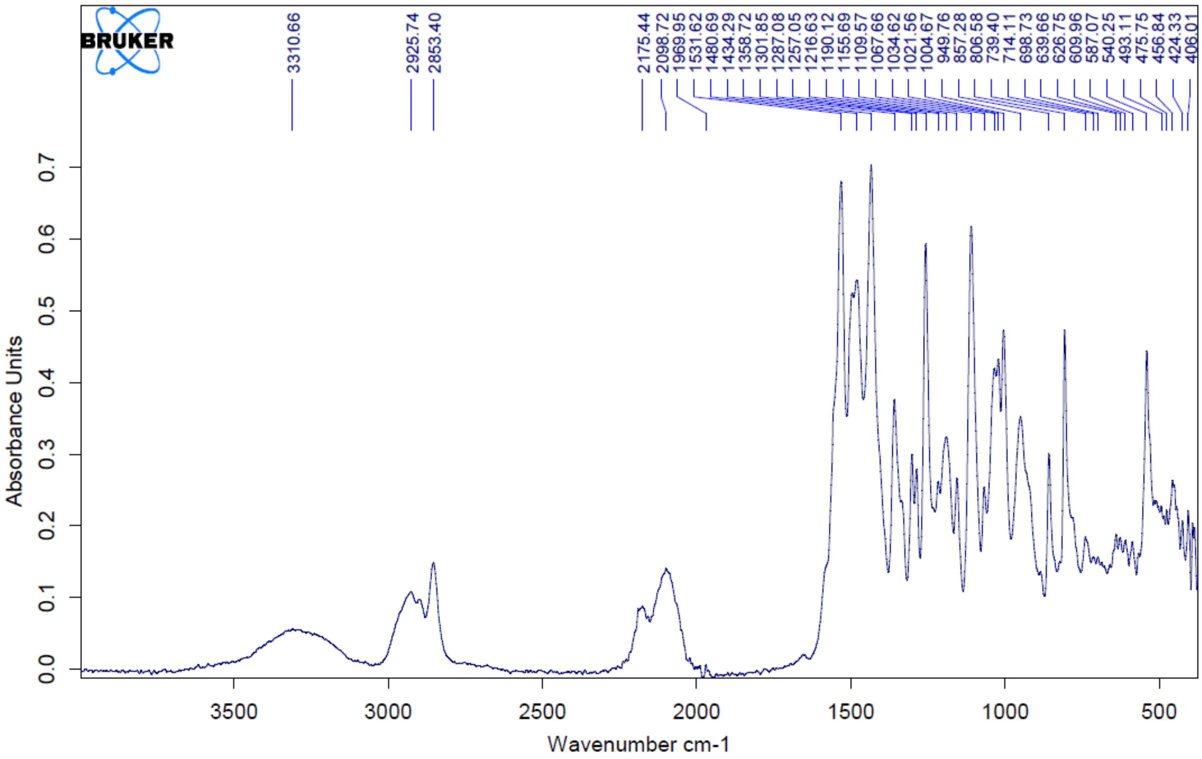

Fig. S187. IR spectra 1f

S54. Analytical spectra of 1g

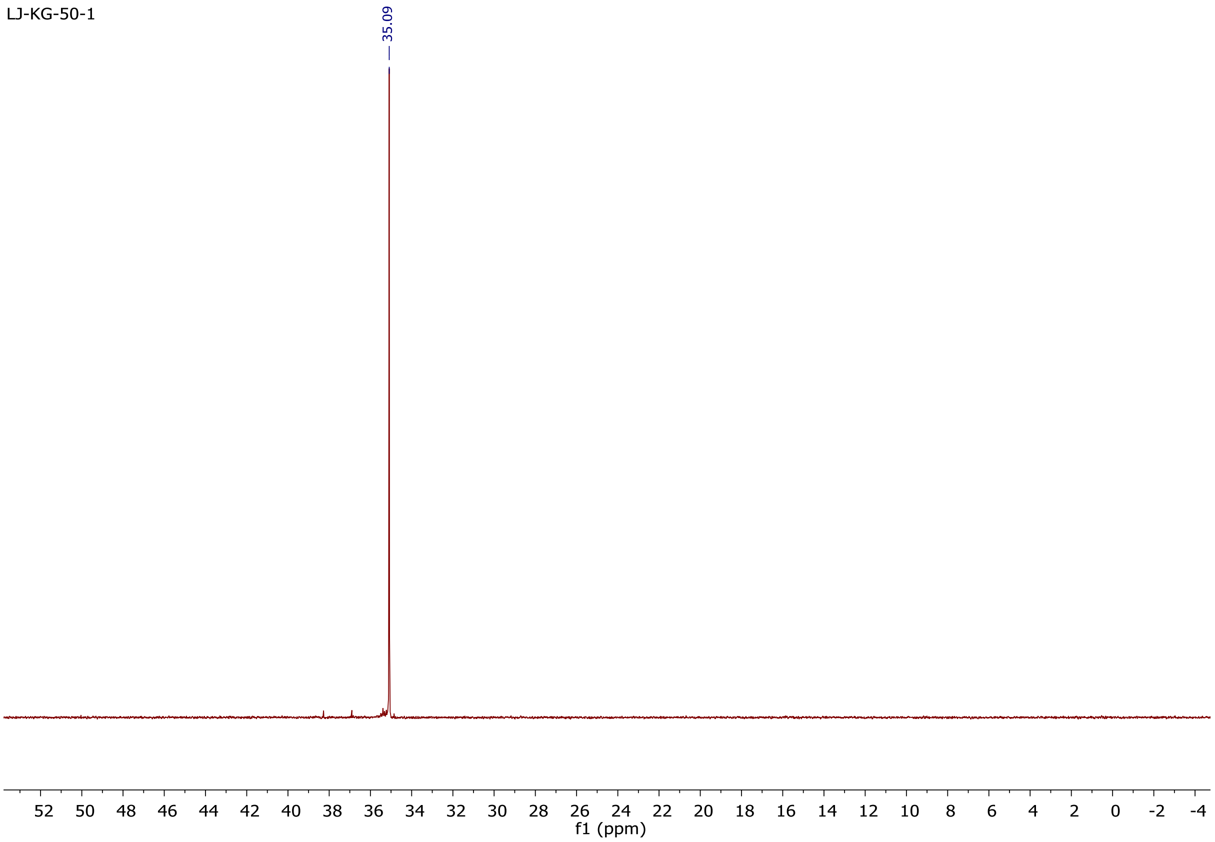

Fig. S188. ^{31}P NMR 1g ($CDCl_3$, 700 MHz).

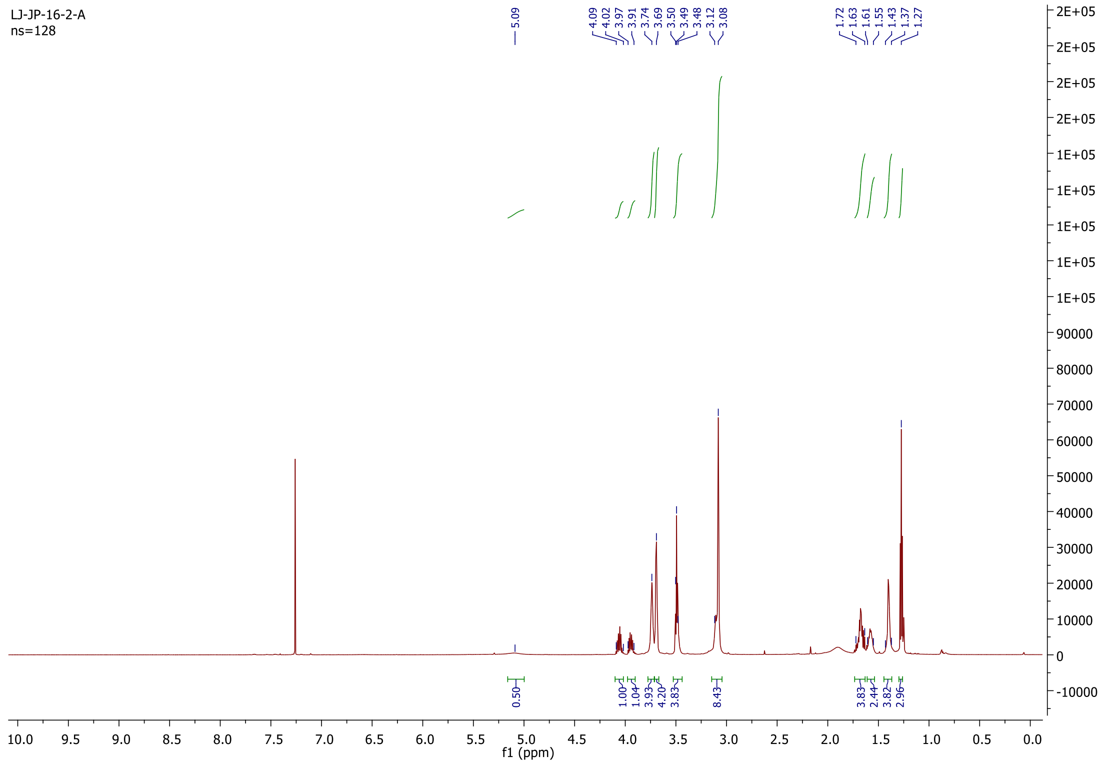

Fig. S189. ^{1}H NMR 1g ($CDCl_3$, 700 MHz).

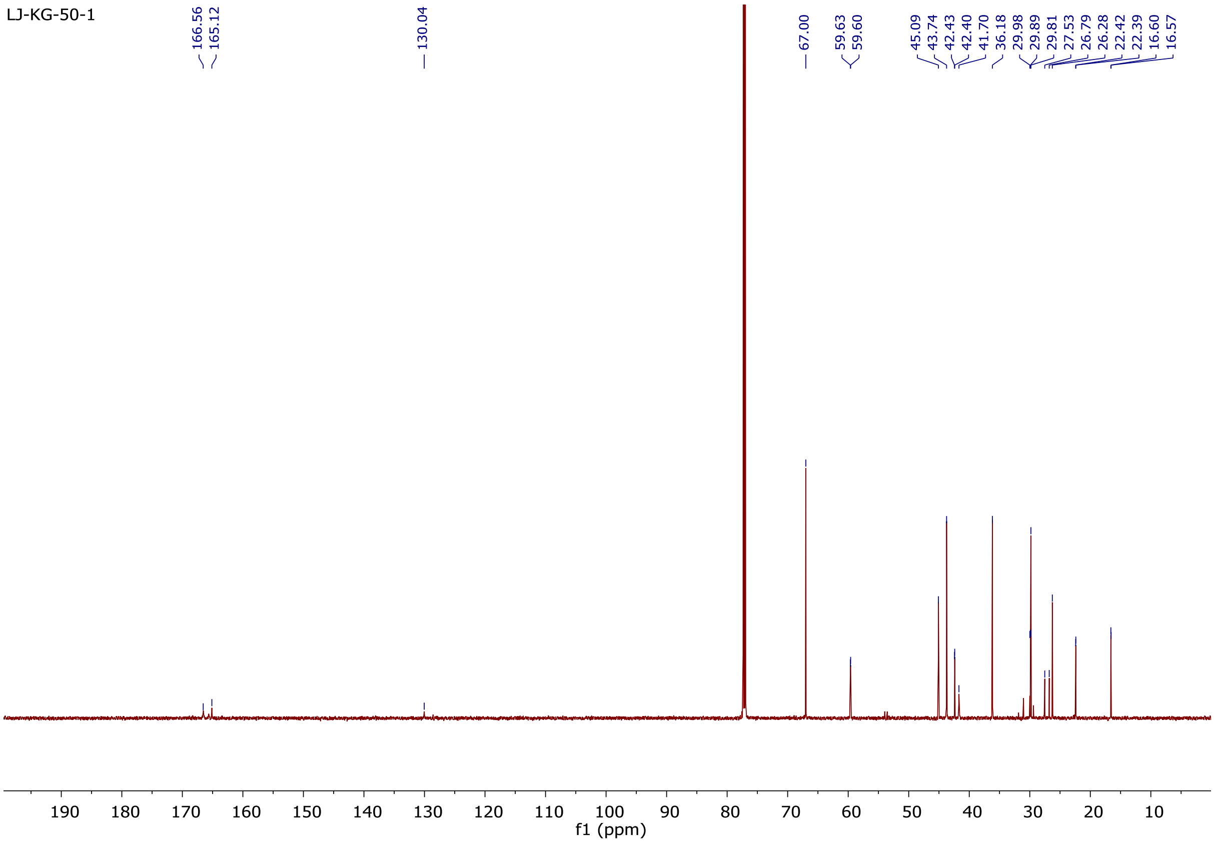

Fig. S190. ^{13}C NMR 1g ($CDCl_3$, 176 MHz).

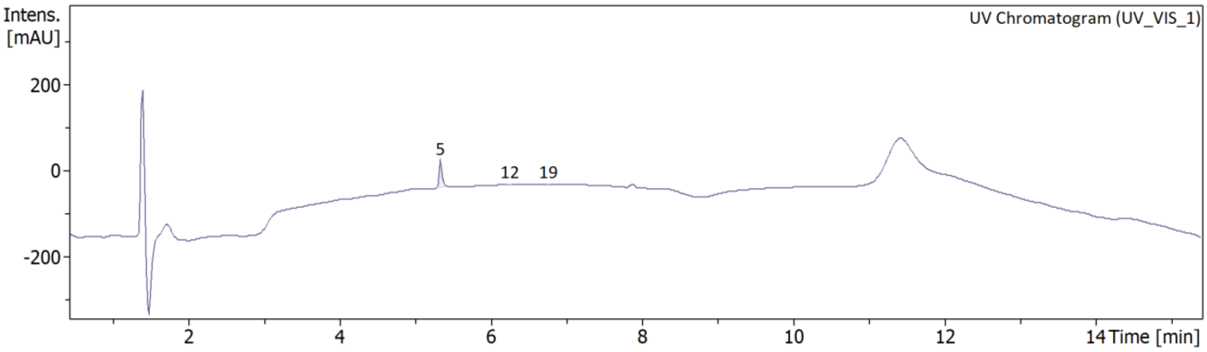

Fig. S191. UV-Vis Chromatogram 1g

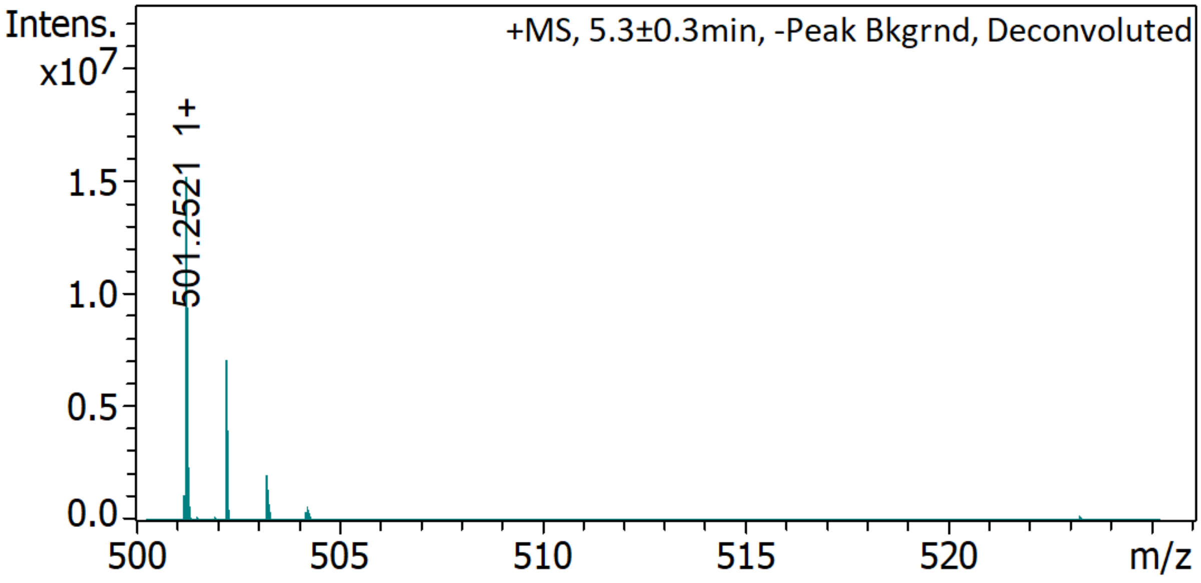

Fig. S192. Mass spectrum 1g

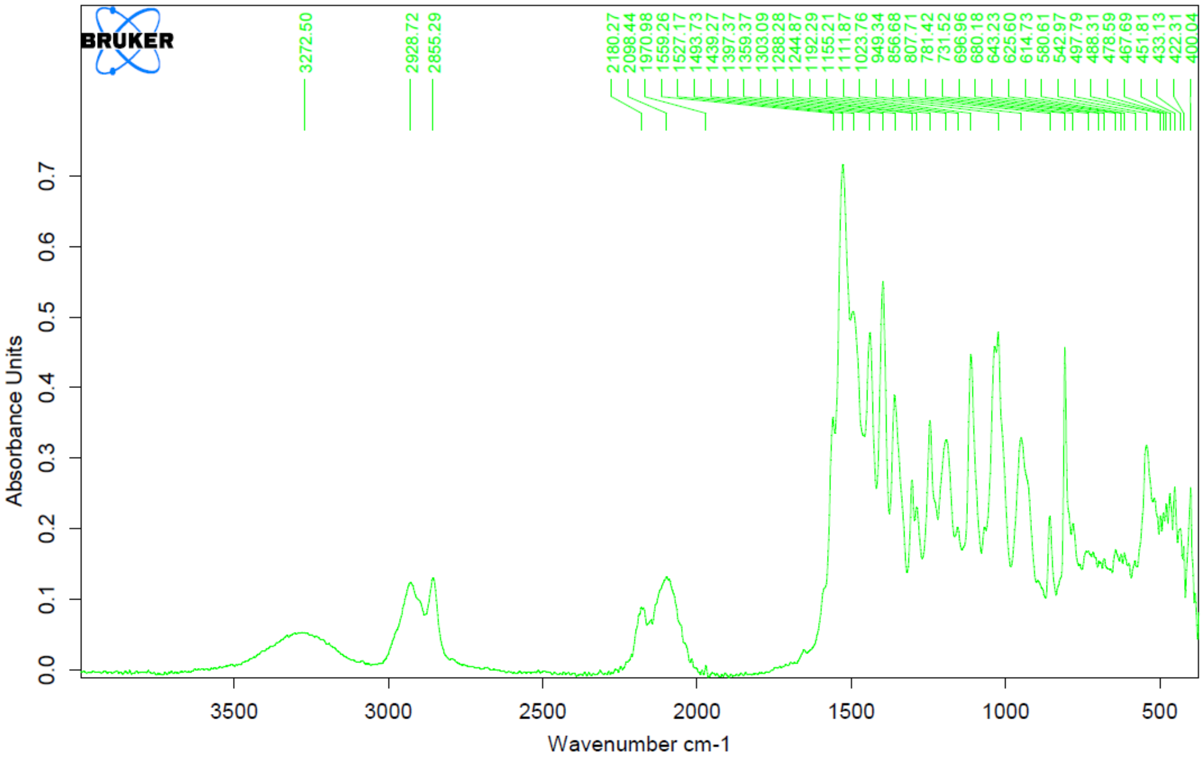

Fig. S193. IR spectra 1g

S55. Analytical spectra of 1h

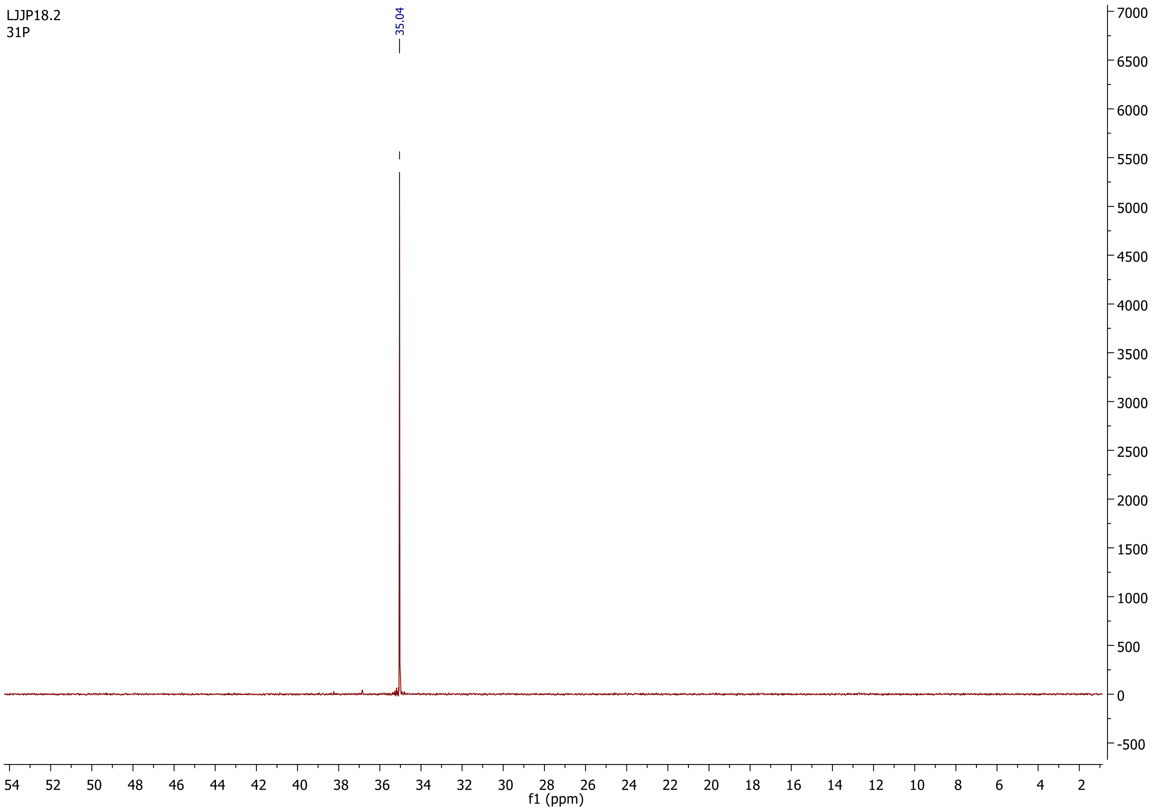

Fig. S194. ^{31}P NMR 1h ($CDCl_3$, 700 MHz).

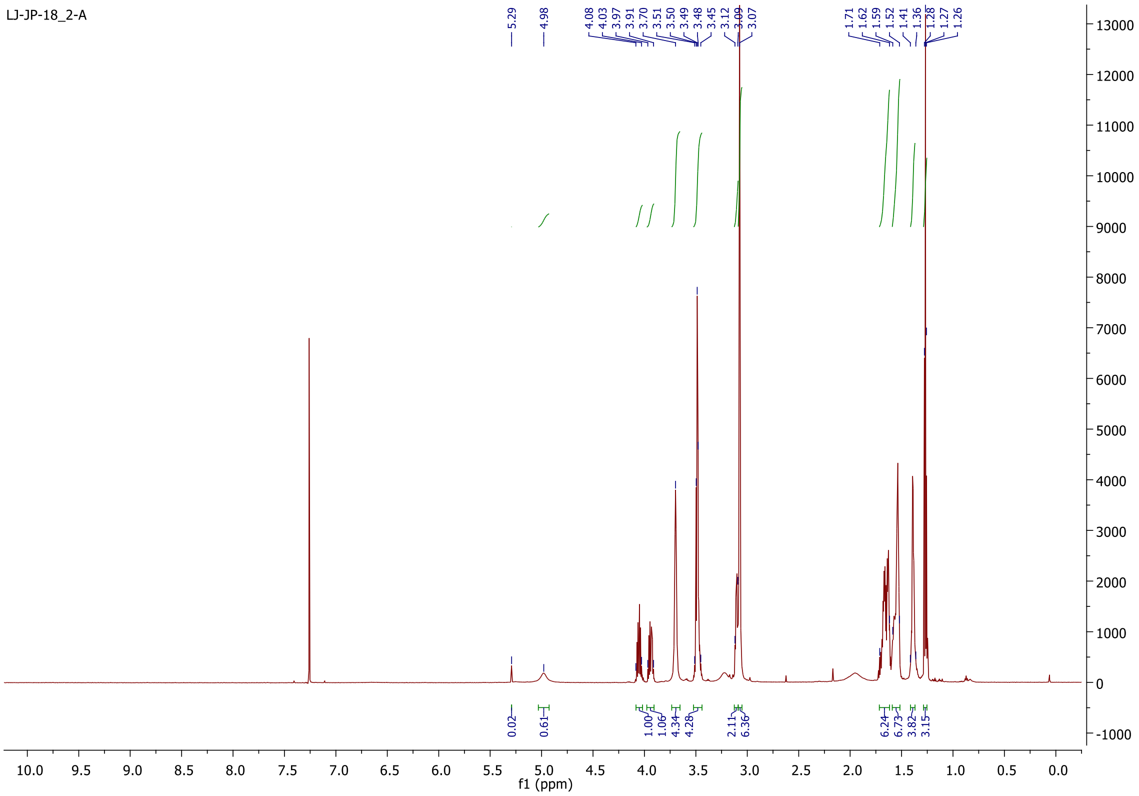

Fig. S195. ^{1}H NMR 1h ($CDCl_3$, 700 MHz).

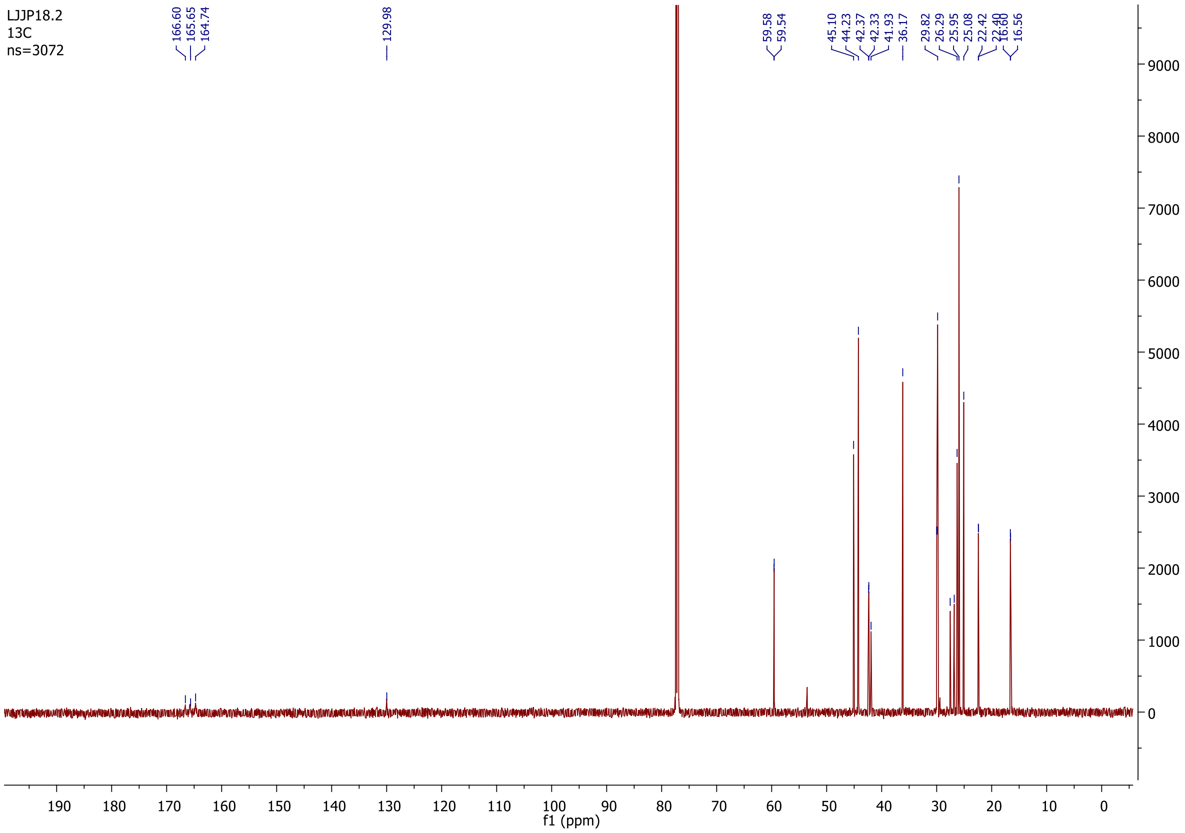

Fig. S196. ^{13}C NMR 1h ($CDCl_3$, 176 MHz).

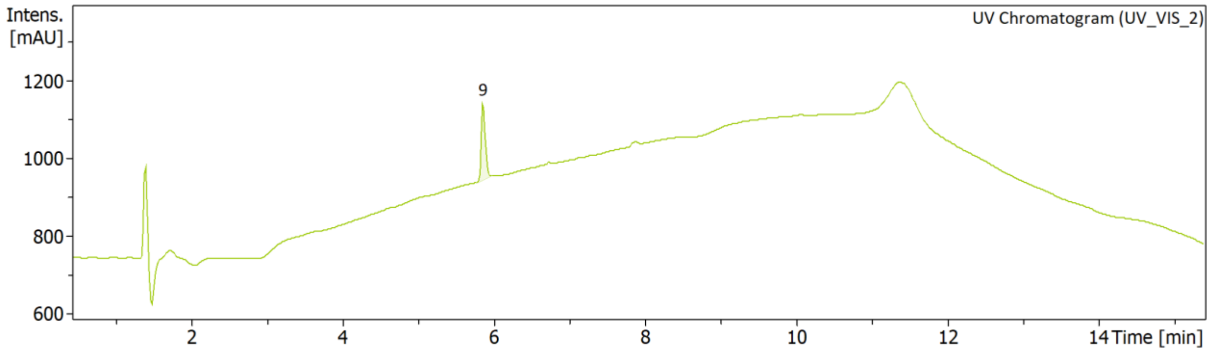

Fig. S197. UV-Vis Chromatogram 1h

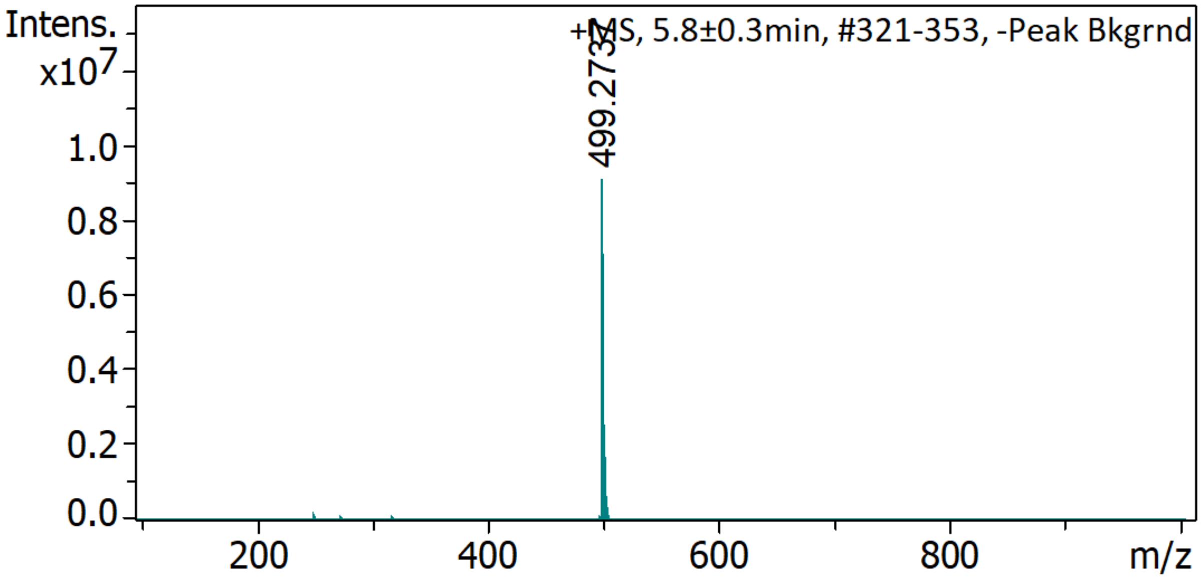

Fig. S198. Mass spectrum 1h

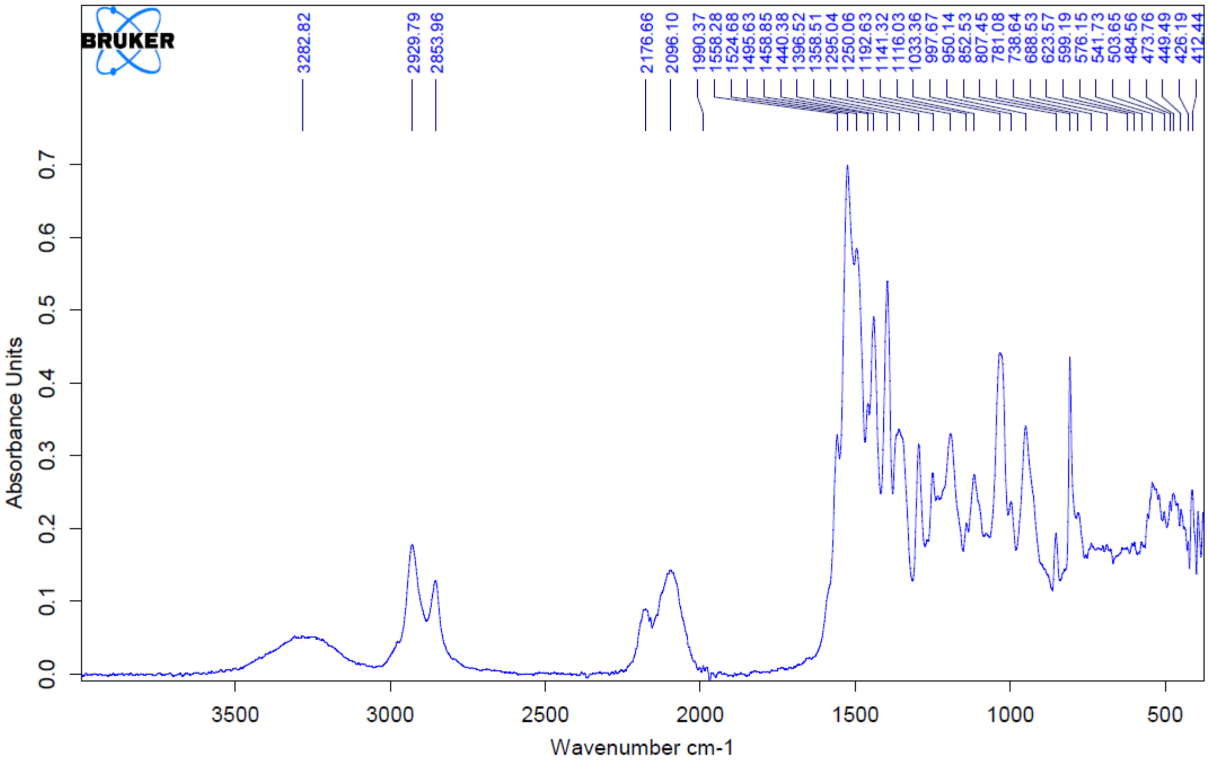

Fig. S199. IR spectra 1h

S56. Analytical spectra of 1i

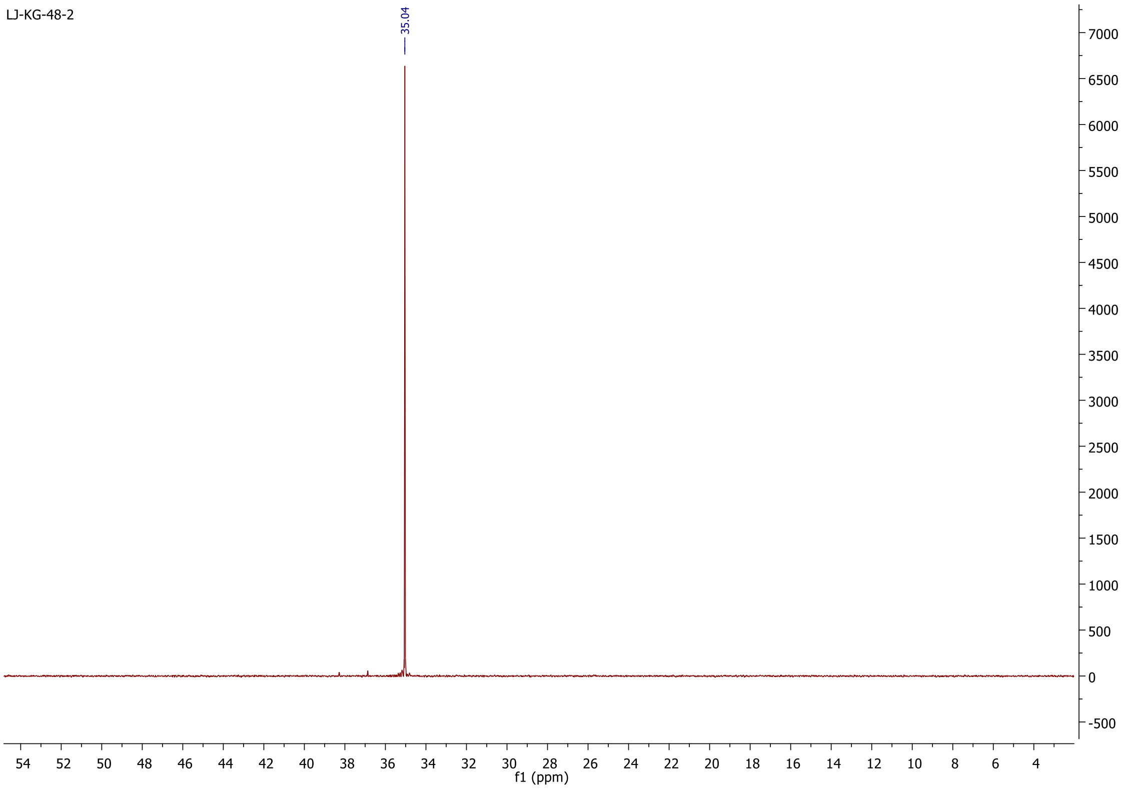

Fig. S200. ^{31}P NMR 1i ($CDCl_3$, 700 MHz).

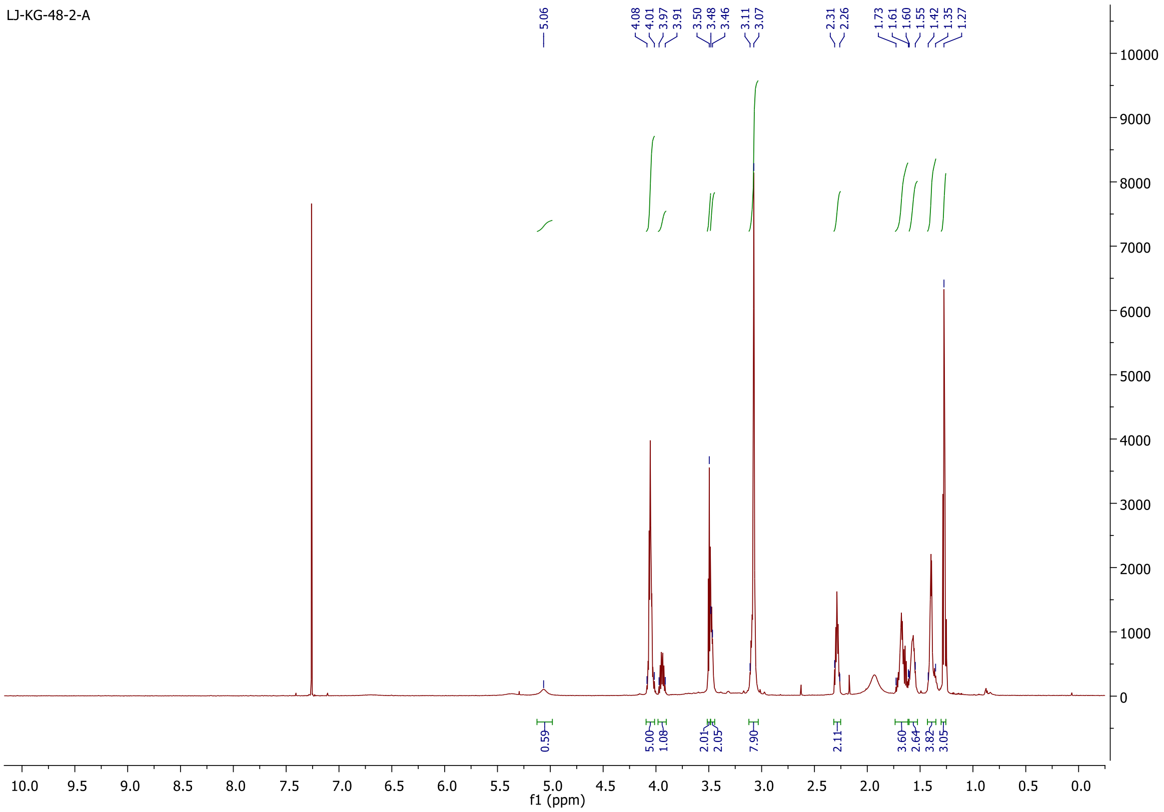

Fig. S201. ^{1}H NMR 1i ($CDCl_3$, 700 MHz).

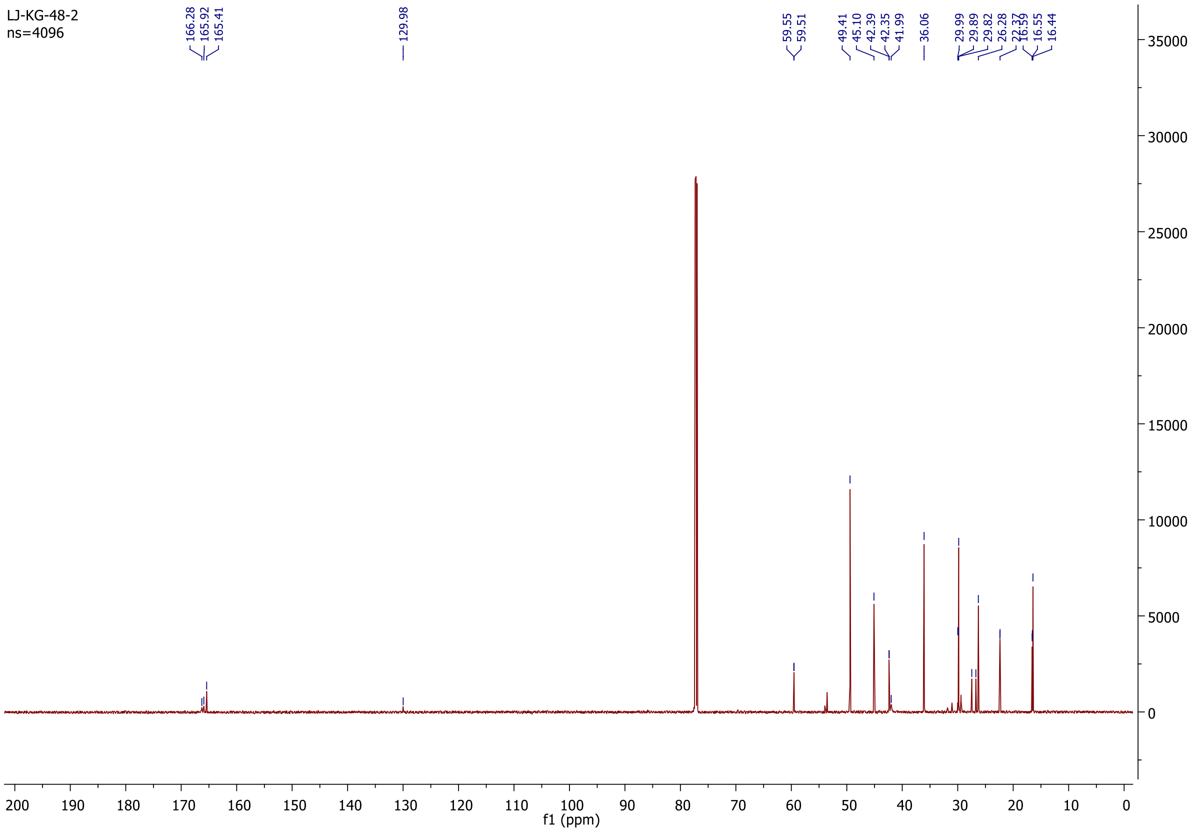

Fig. S202. ^{13}C NMR 1i ($CDCl_3$, 176 MHz).

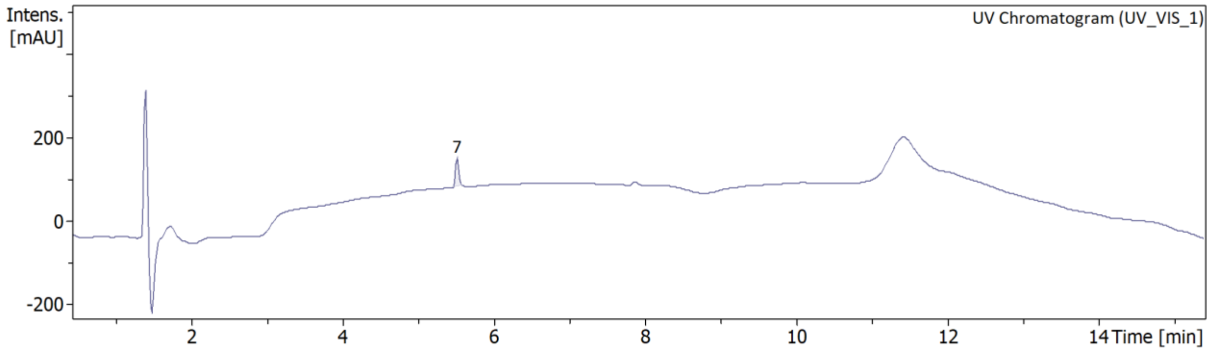

Fig. S203. UV-Vis Chromatogram 1i

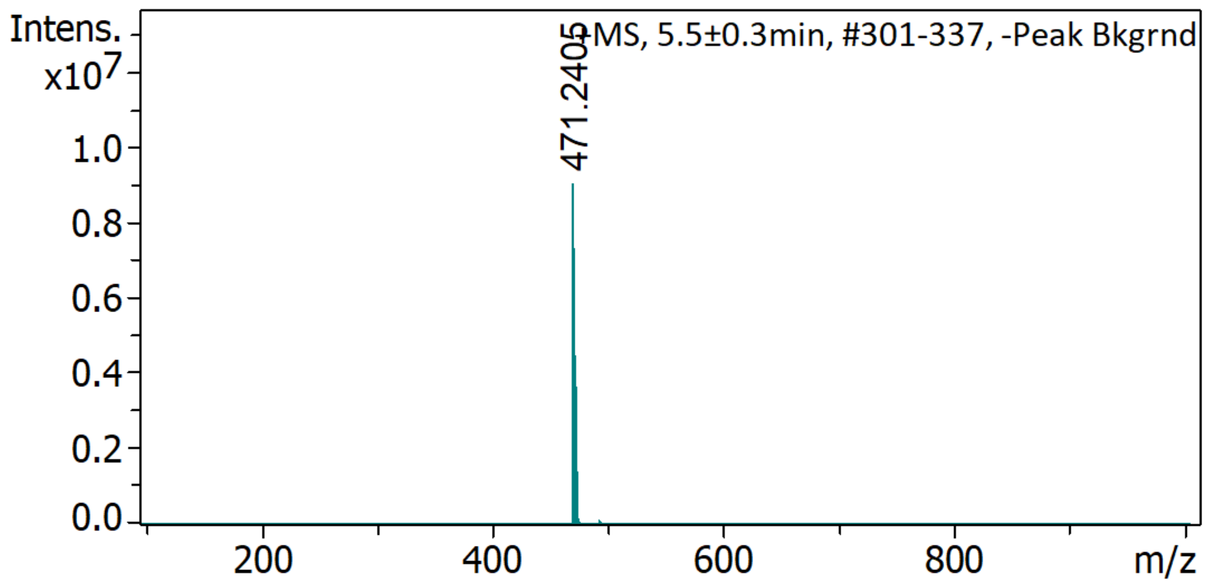

Fig. S204. Mass spectrum 1i

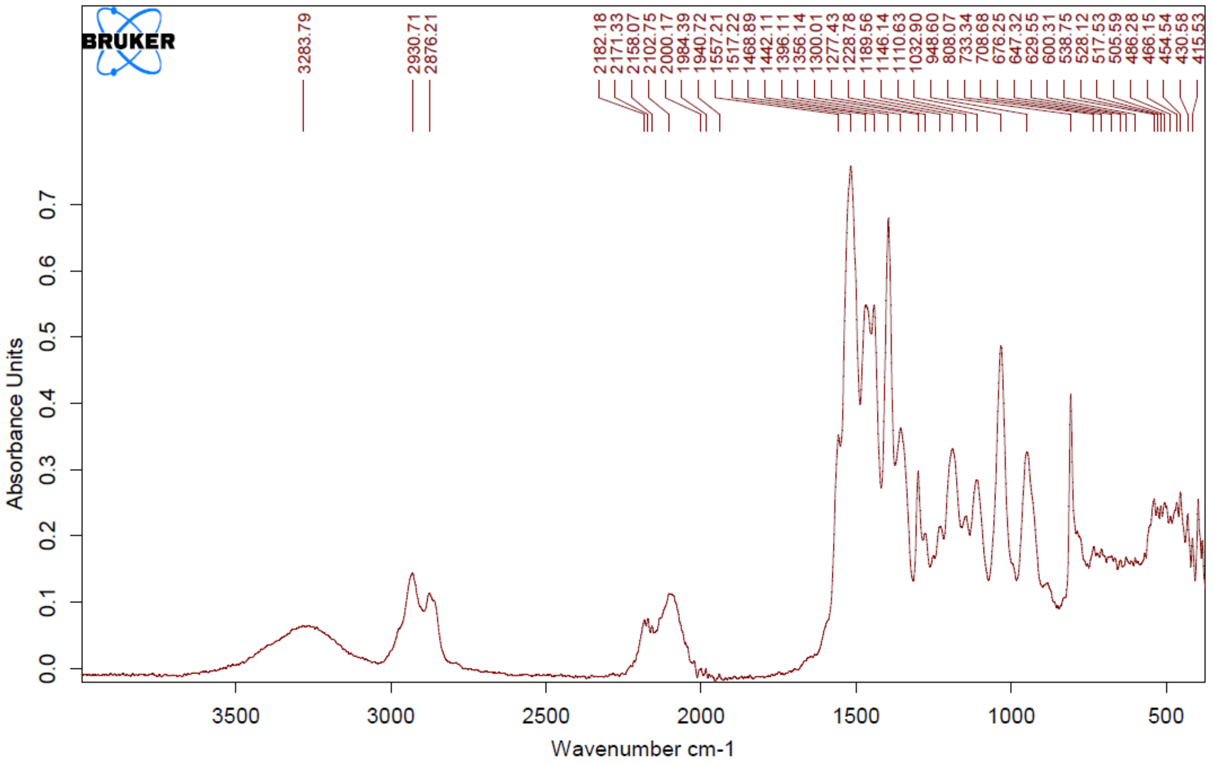

Fig. S205. IR spectra 1i

S57. Analytical spectra of 1j

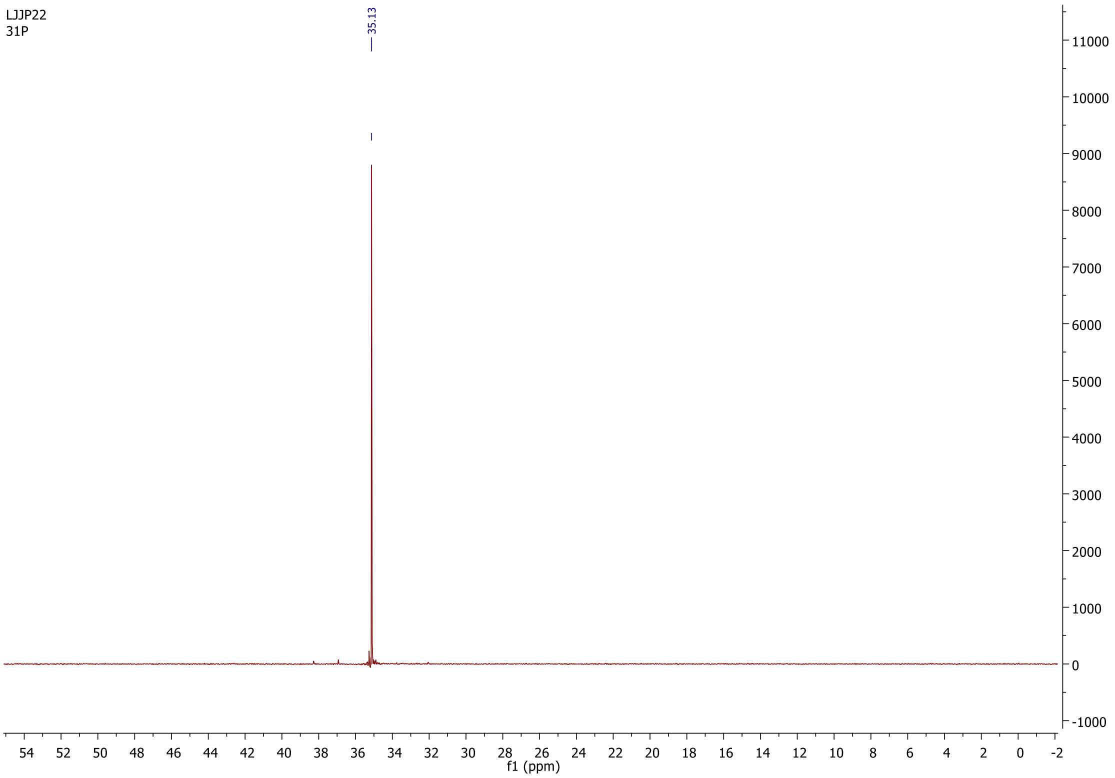

Fig. S206. ^{31}P NMR 1j ($CDCl_3$, 700 MHz).

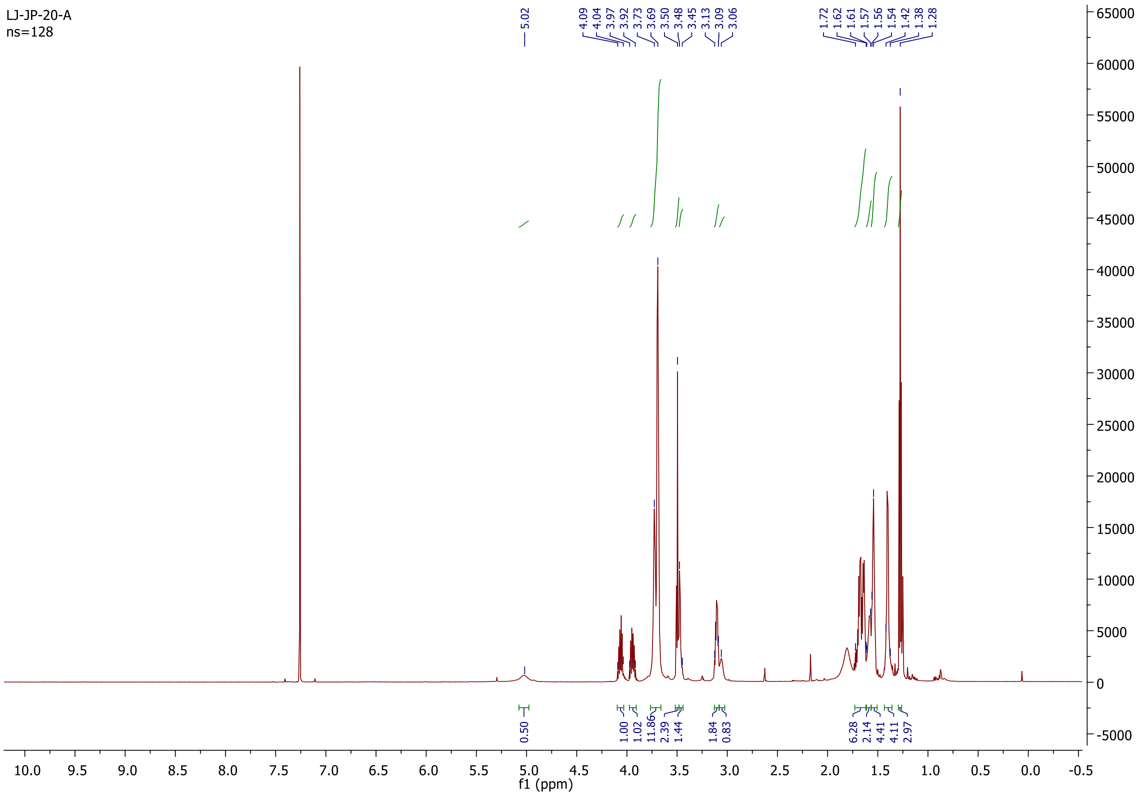

Fig. S207. ^{1}H NMR 1j ($CDCl_3$, 700 MHz).

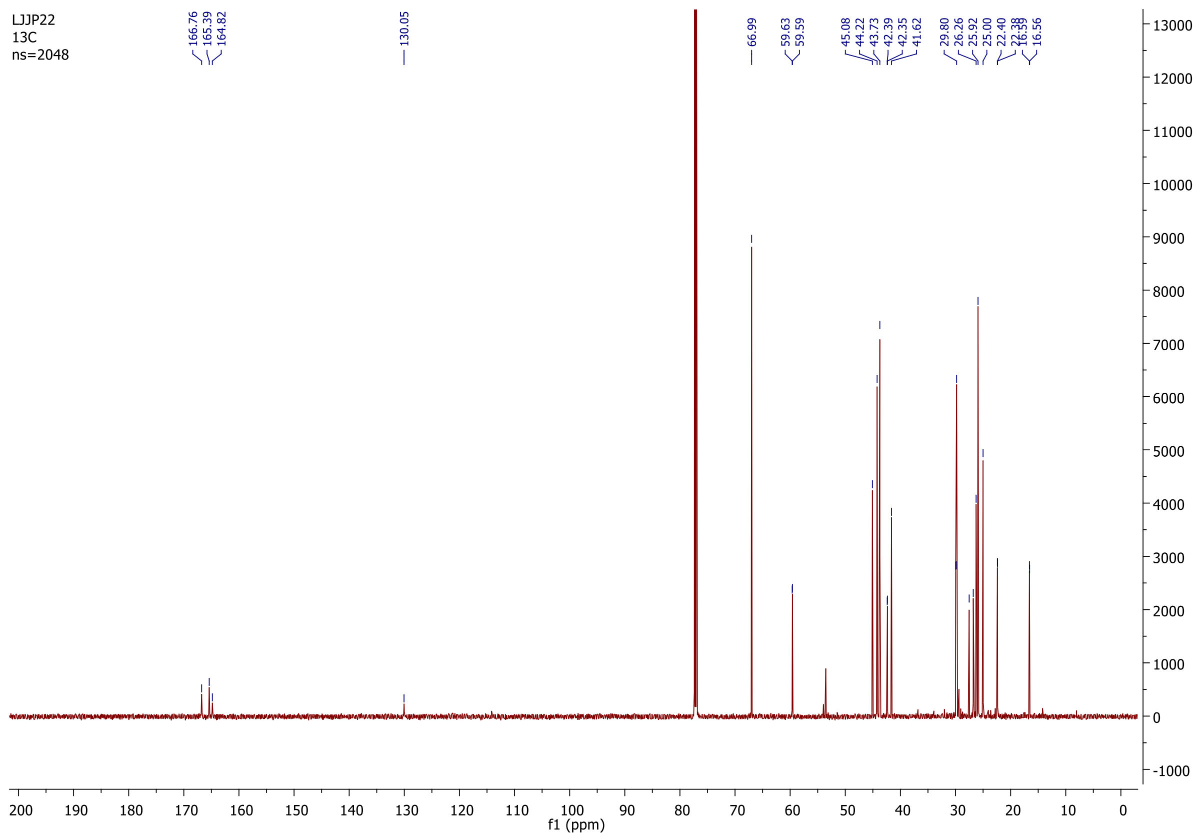

Fig. S208. ^{13}C NMR 1j ($CDCl_3$, 176 MHz).

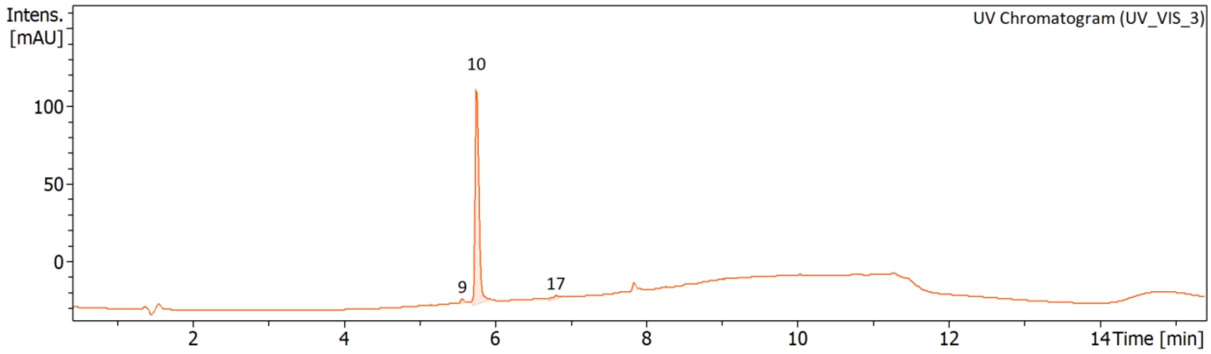

Fig. S209. UV-Vis Chromatogram 1j

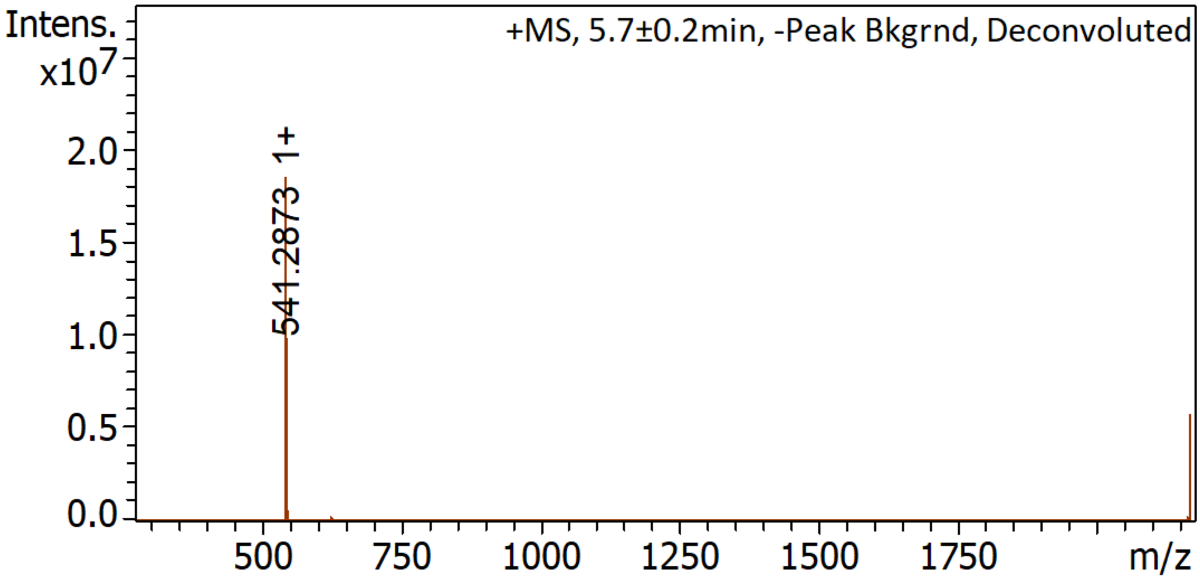

Fig. S210. Mass spectrum of 1j

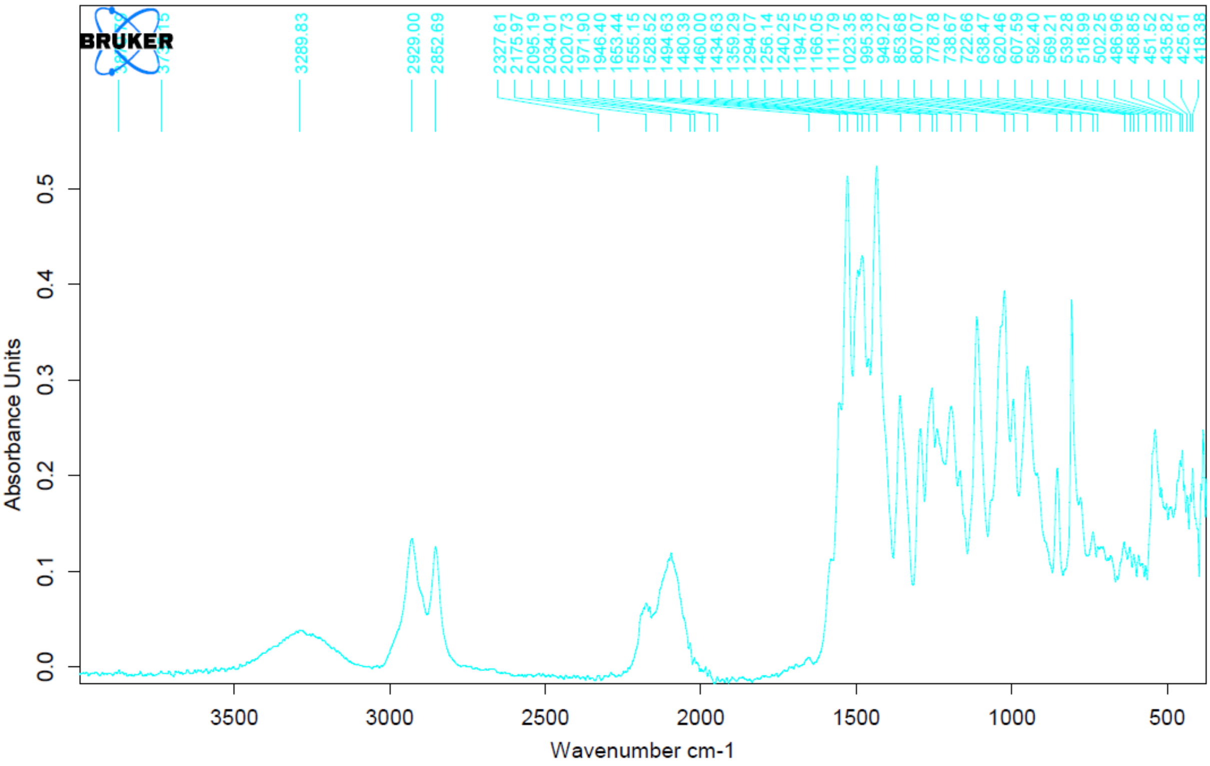

Fig. S211. IR spectra 1j

S58. Analytical spectra of 1k

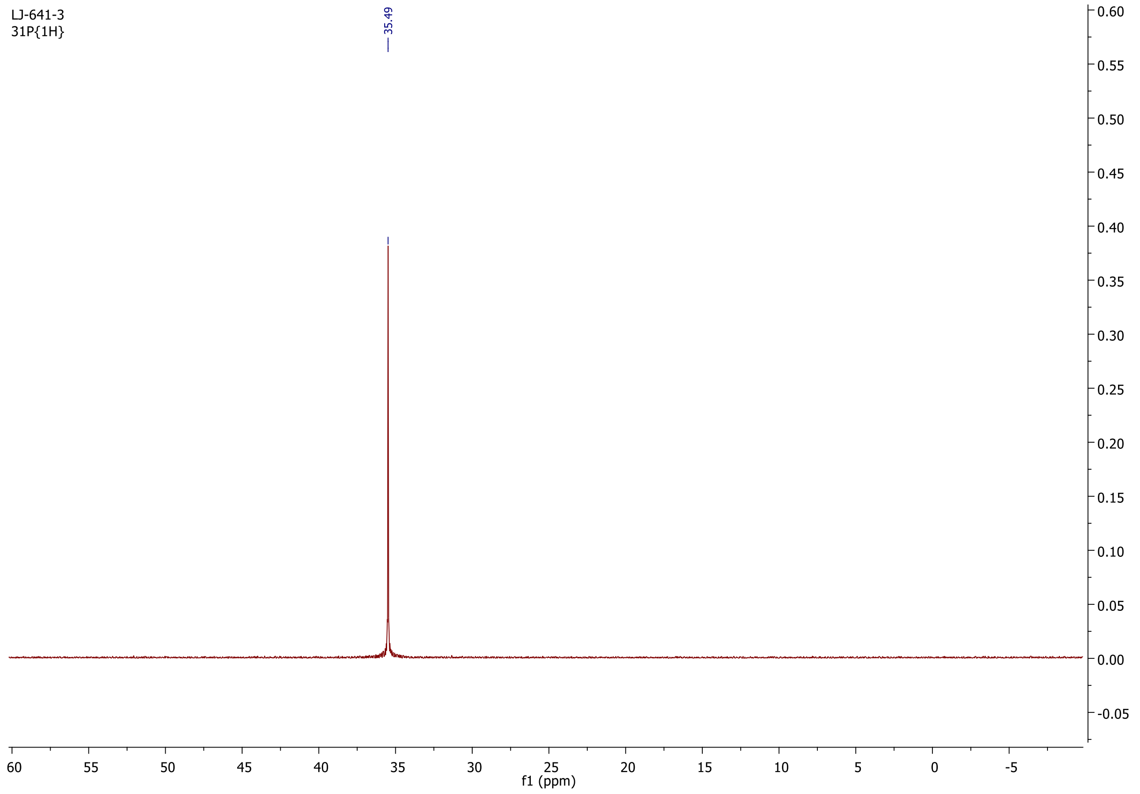

Fig. S212. ^{31}P NMR 1k ($CDCl_3$, 700 MHz).

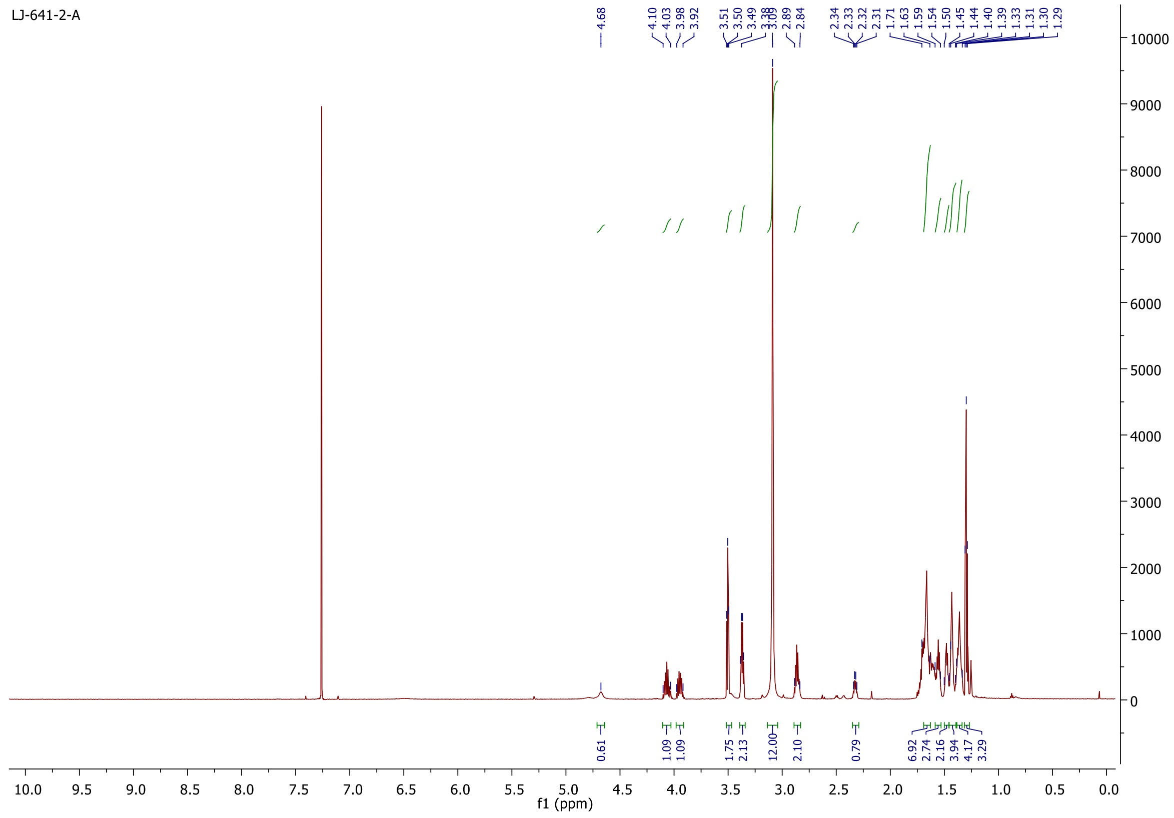

Fig. S213. ^{1}H NMR 1k ($CDCl_3$, 700 MHz).

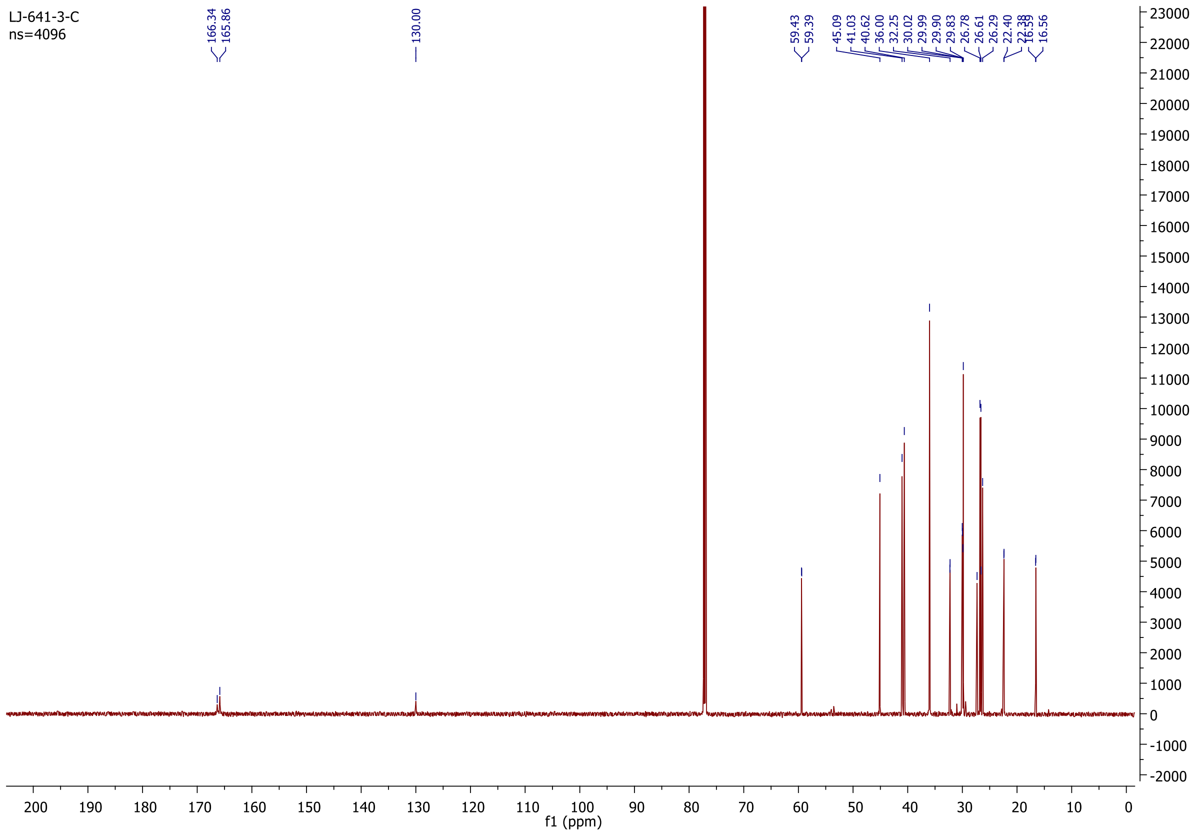

Fig. S214. ^{13}C NMR 1k ($CDCl_3$, 176 MHz).

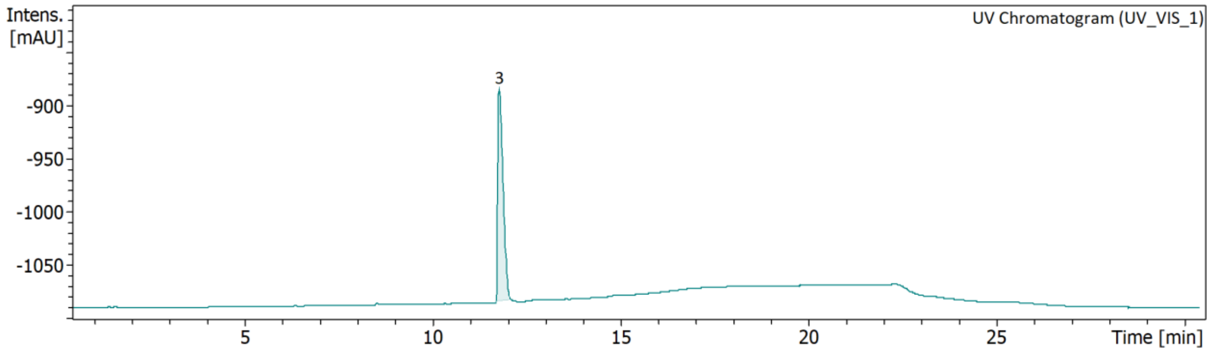

Fig. S215. UV-Vis Chromatogram 1k

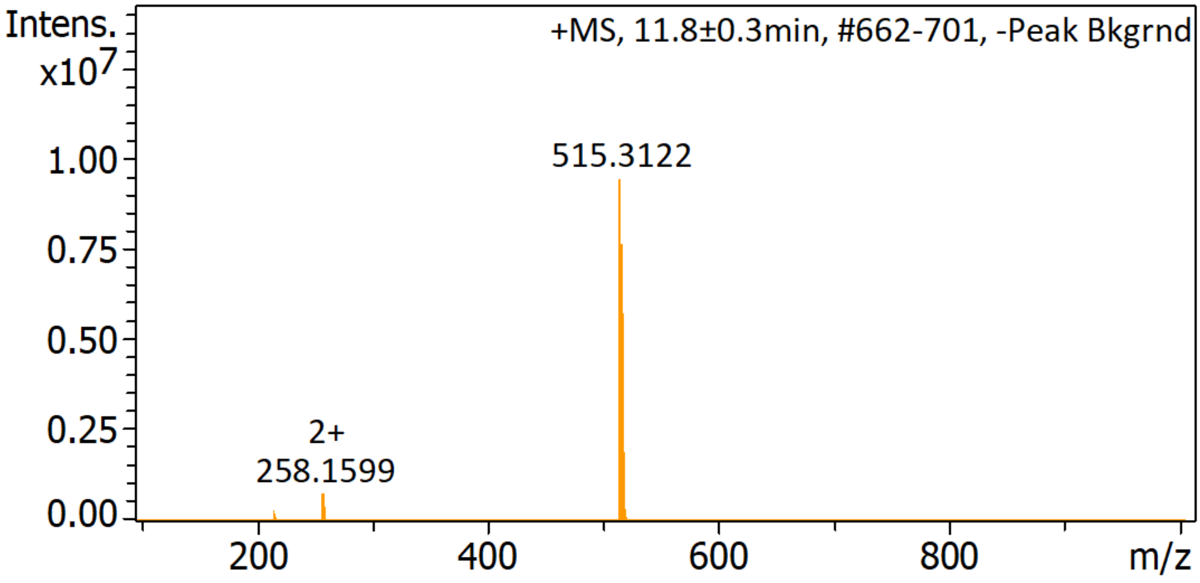

Fig. S216. Mass spectrum 1k

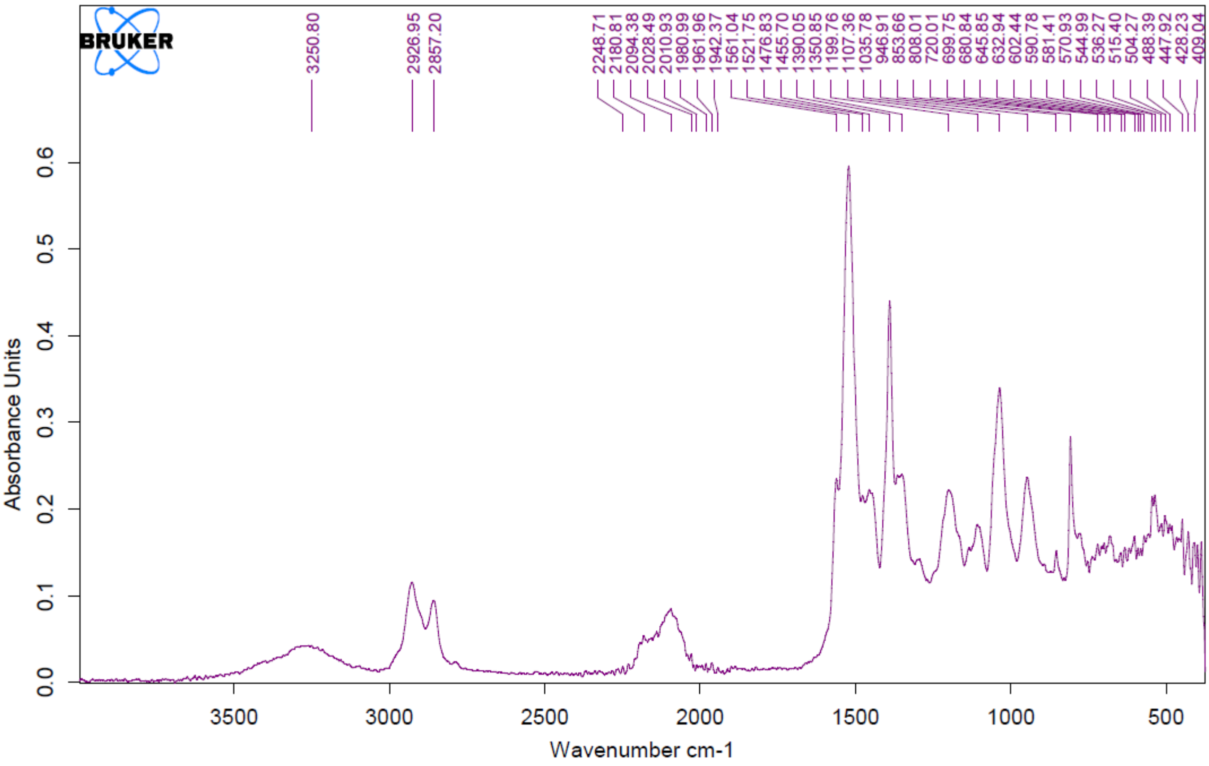

Fig. S217. IR spectra 1k

Dose–response curves on AChE for compounds 1a-k

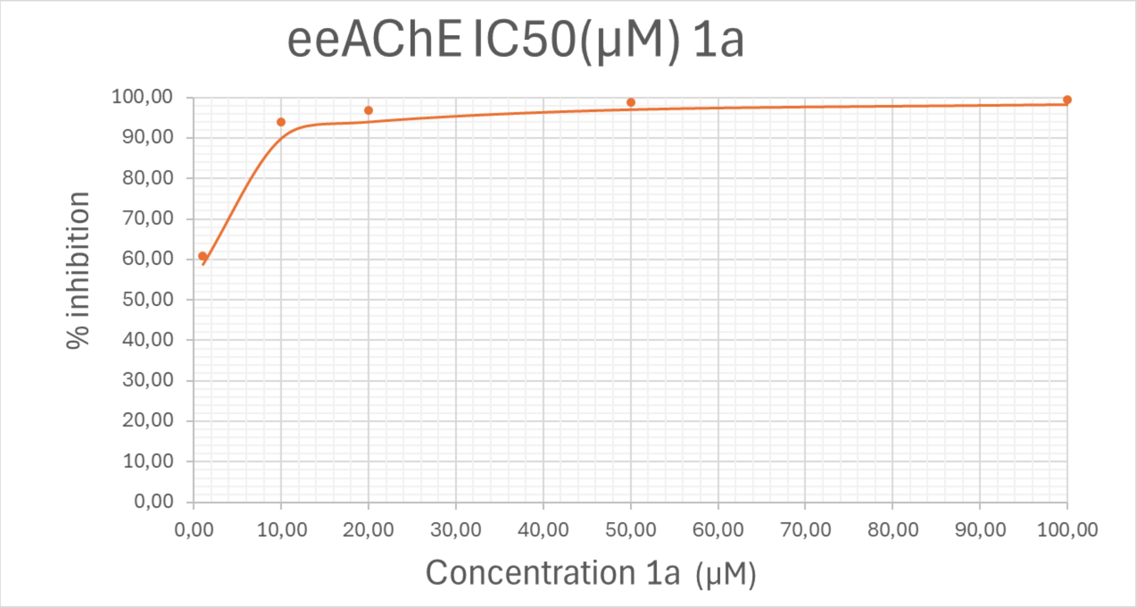

the regression formula: a0+(a1-a0)/(1+(x/a2)*a3)

	r2 Coef Det	DF Adj r2	Fit Std Err	F-Statistic	
	0.985921	0.943682	3.909532	23.341859	
	Value	Std Error	t-Value	95% Confidence Limits	
a0	96.479009	3.256634	29.625373	55.100211	137.857807
a1	689.576516	16044.876241	0.042978	-203176.62626	204555.779301
a2	-0.714715	3741393.24093	-1.9103e-07	-47538144.461	47538143.0320
a3	11.844784	62067049.0527	1.9084e-07	-788623933.78	788623957.471

Fig. S218. Dose–response curves on AChE for compounds **1a**

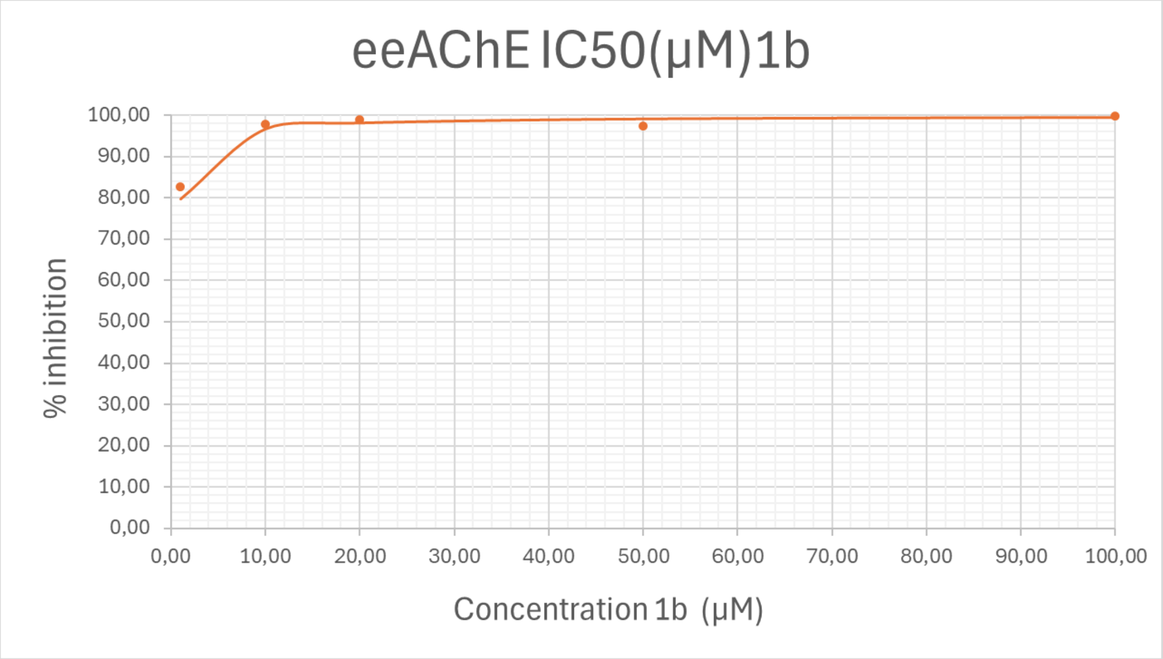

	r2 Coef Det	DF Adj r2	Fit Std Err	F-Statistic	
	0.997713	0.990851	0.806871	145.408074	
	Value	Std Error	t-Value	95% Confidence Limits	
a0	99.321497	0.673838	147.396694	90.75971	107.883283
a1	2202.502838	144877.027543	0.015203	-1838605.0566	1843010.06232
a2	-0.202627	710736.716024	-2.8509e-07	-9030621.1495	9030620.74434
a3	21.883031	76833913.0421	2.8481e-07	-976251702.43	976251746.203

Fig. S219. Dose–response curves on AChE for compounds **1b**

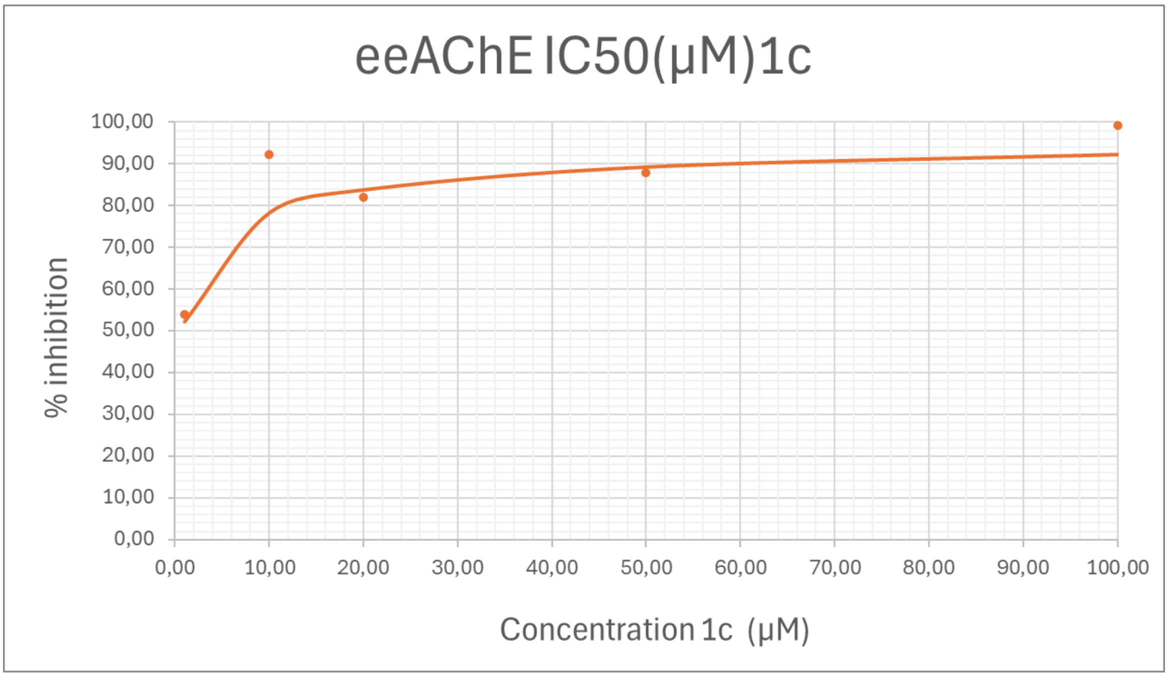

	r2 Coef Det	DF Adj r2	Fit Std Err	F-Statistic	
	0.933143	0.732574	8.288339	4.652462	
	Value	Std Error	t-Value	95% Confidence Limits	
a0	87.632975	6.918738	12.666034	-0.276516	175.542465
a1	2043.669021	301399.817528	0.006781	-3827542.5113	3831629.84941
a2	-4.324743	527376470.117	-8.2005e-09	-6700845606.6	6700845597.99
a3	238.620962	2.9127e+10	8.1923e-09	-3.7009e+11	3.7009e+11

Fig. S220. Dose–response curves on AChE for compounds **1c**

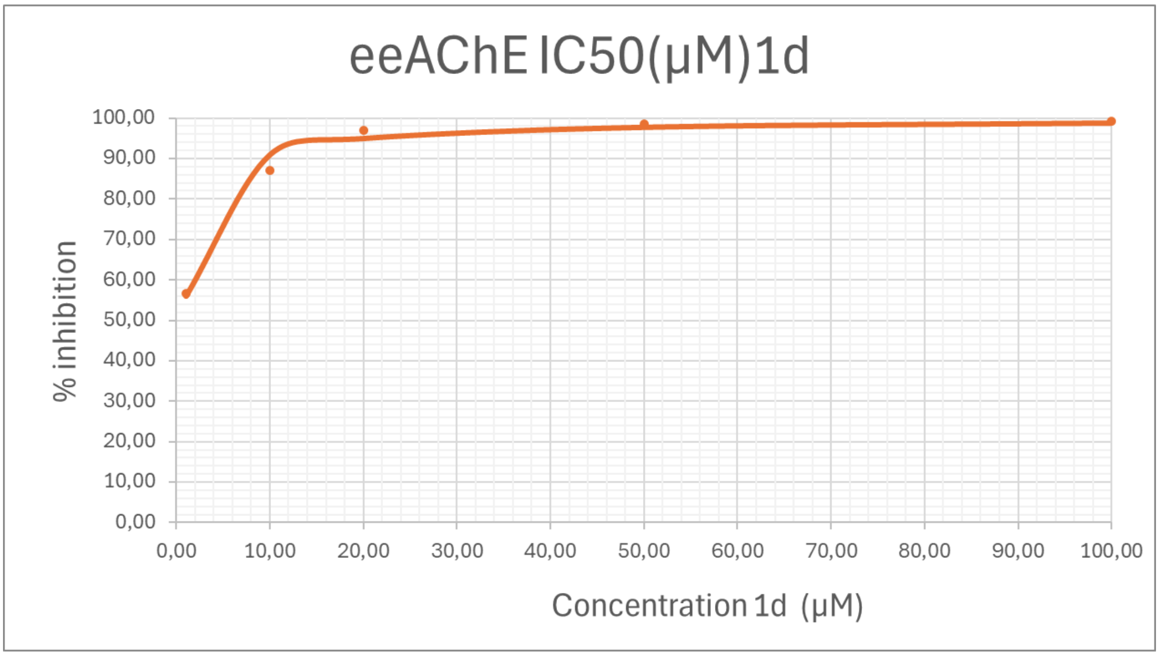

	r2 Coef Det	DF Adj r2	Fit Std Err	F-Statistic	
	0.992544	0.970176	3.10549	44.372806	
	Value	Std Error	t-Value	95% Confidence Limits	
a0	97.554147	2.59493	37.594128	64.58296	130.525334
a1	-20677.094071	163848642.900	-0.000126	-2081881587.5	2081840233.35
a2	0.00494	19465.261576	2.5380e-07	-247325.61502	247325.624905
a3	2.449759	9681419.96162	2.5304e-07	-123012122.75	123012127.658

Fig. S221. Dose–response curves on AChE for compounds **1d**

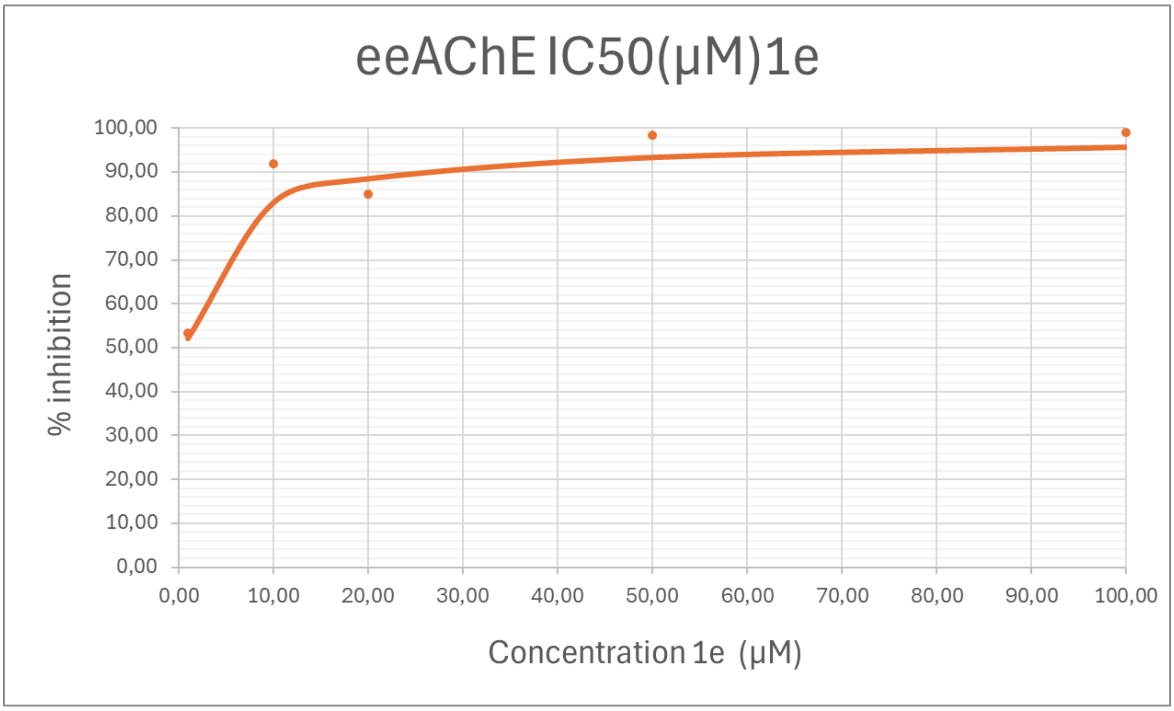

	r2 Coef Det	DF Adj r2	Fit Std Err	F-Statistic	
	0.960621	0.842484	6.995034	8.131423	
	Value	Std Error	t-Value	95% Confidence Limits	
a0	91.962045	5.837334	15.754118	17.792881	166.13121
a1	1706.080369	182708.746401	0.009338	-2319791.3113	2323203.47207
a2	-4.483207	66055142.3332	-6.7871e-08	-839296664.64	839296655.673
a3	184.159117	2716093605.57	6.7803e-08	-3.4511e+10	3.4511e+10

Fig. S222. Dose–response curves on AChE for compounds **1e**

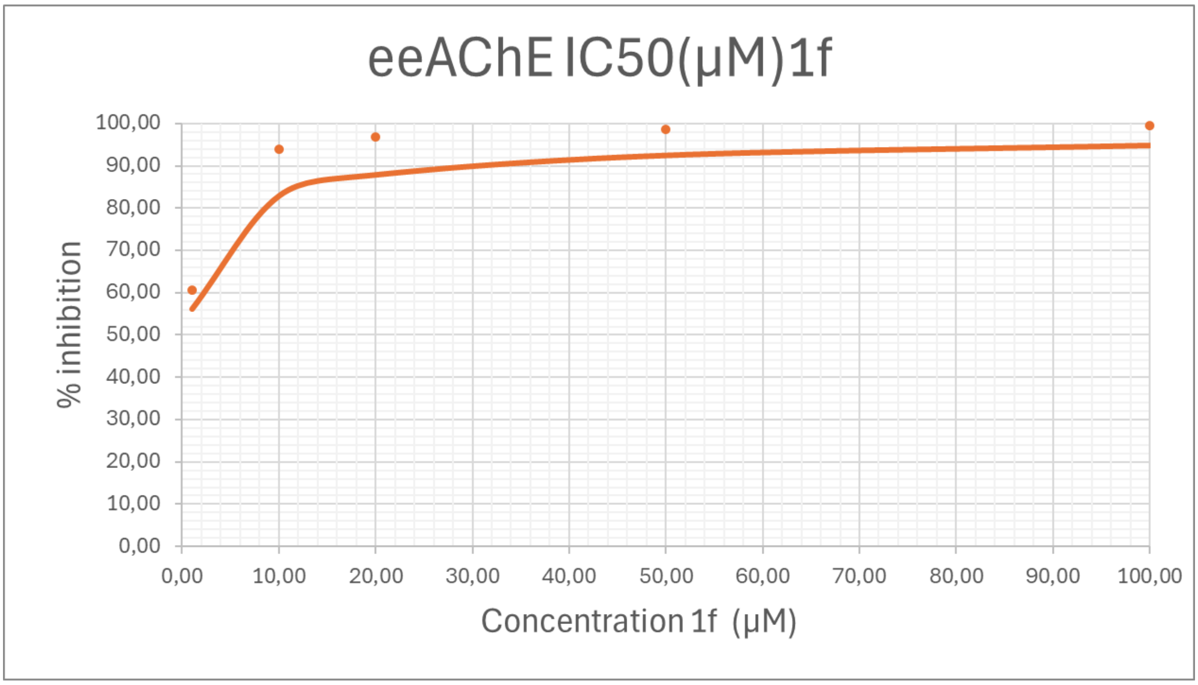

	r2 Coef Det	DF Adj r2	Fit Std Err	F-Statistic	
	0.953417	0.813668	6.741341	6.82237	
	Value	Std Error	t-Value	95% Confidence Limits	
a0	91.260705	5.626222	16.2206	19.773921	162.747488
a1	1655.573814	210724.348263	0.007857	-2675808.0643	2679119.21198
a2	-3.991596	57693825.4822	-6.9186e-08	-733057769.49	733057761.513
a3	179.606155	2598588810.70	6.9117e-08	-3.3018e+10	3.3018e+10

Fig. S223. Dose–response curves on AChE for compounds **1f**

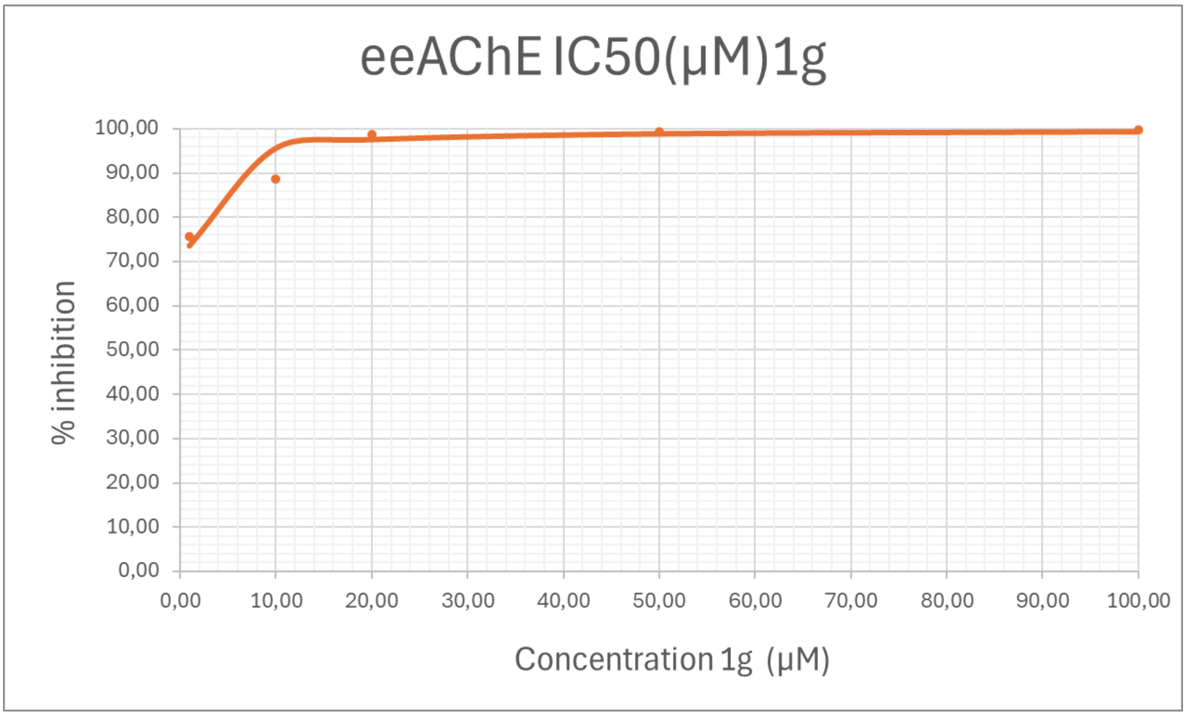

	r2 Coef Det	DF Adj r2	Fit Std Err	F-Statistic	
	0.996562	0.986249	1.289	96.632373	
	Value	Std Error	t-Value	95% Confidence Limits	
a0	98.983741	1.07176	92.356236	85.365955	112.601527
a1	336.108201	1917.021822	0.175328	-24021.571699	24693.7881
a2	-0.198195	589531.839673	-3.3619e-07	-7490591.9464	7490591.55009
a3	2.04023	6074725.07380	3.3586e-07	-77185456.740	77185460.8208

Fig. S224. Dose–response curves on AChE for compounds **1g**

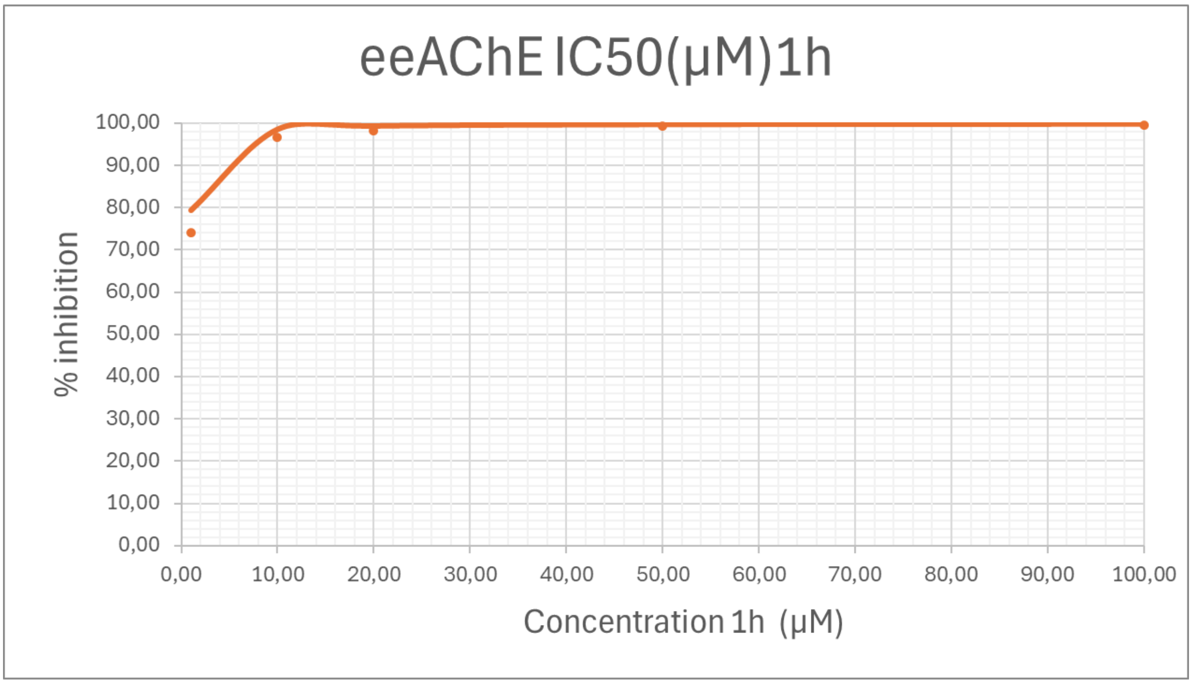

	r2 Coef Det	DF Adj r2	Fit Std Err	F-Statistic	
	0.999094	0.996377	0.538873	367.693306	
	Value	Std Error	t-Value	95% Confidence Limits	
a0	100.433378	0.453905	221.265068	94.666057	106.200698
a1	-730.540067	1771292.28648	-0.000412	-22506770.913	22505309.8331
a2	0.284029	311260.907565	9.1251e-07	-3954880.9096	3954881.47766
a3	11.007238	12098127.6061	9.0983e-07	-153718802.32	153718824.339

Fig. S225. Dose–response curves on AChE for compounds **1h**

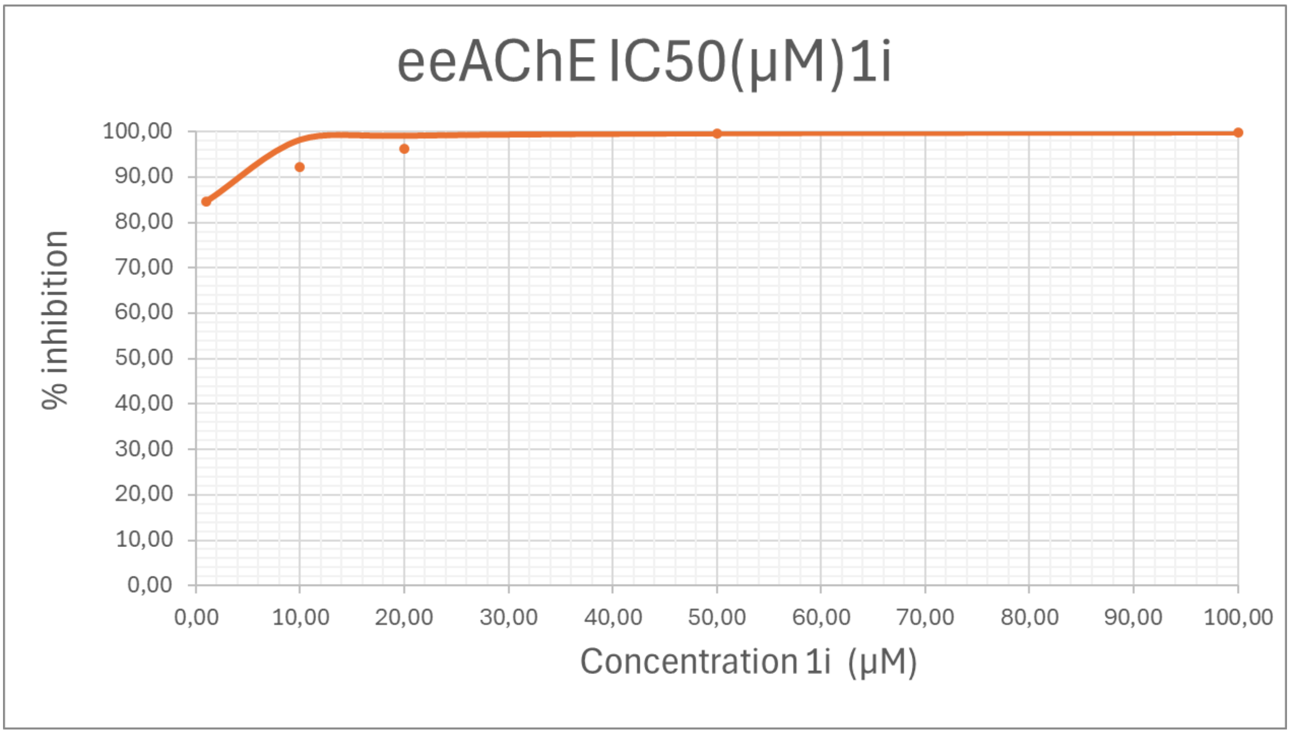

	r2 Coef Det	DF Adj r2	Fit Std Err	F-Statistic	
	1	1	0.001838	16956252.5278	
	Value	Std Error	t-Value	95% Confidence Limits	
a0	100.003339	0.001541	64910.593098	99.983764	100.022915
a1	1.521794	11.334205	0.134266	-142.490615	145.534204
a2	0.414633	6000.309484	0.000069	-76239.519637	76240.348903
a3	2.23633	32363.078743	0.000069	-411203.05279	411207.525454

Fig. S226. Dose–response curves on AChE for compounds **1i**

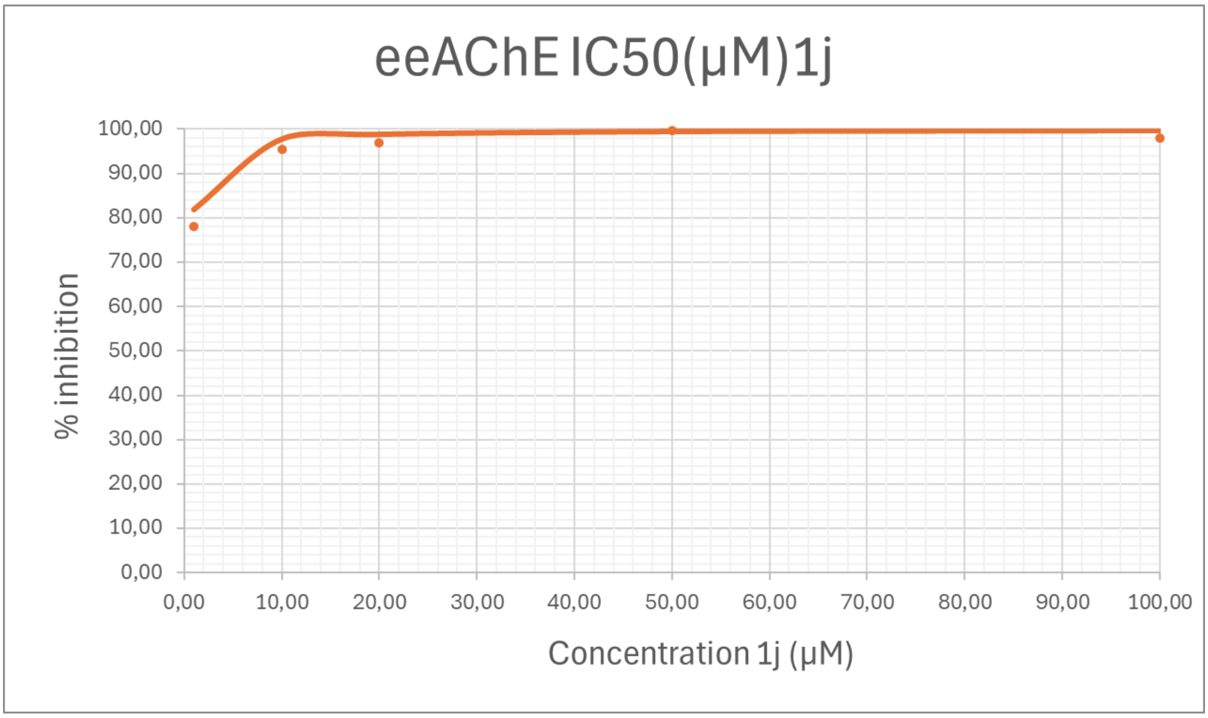

	r2 Coef Det	DF Adj r2	Fit Std Err	F-Statistic	
	1	1	0.001266	49950271.2600	
	Value	Std Error	t-Value	95% Confidence Limits	
a0	100.003609	0.00107	93495.454192	99.990018	100.017199
a1	0.486865	1.138862	0.427501	-13.98352	14.957251
a2	0.527754	2155.077403	0.000245	-27381.88644	27382.941947
a3	2.352659	9607.097707	0.000245	-122065.43395	122070.139273

Fig. S227. Dose–response curves on AChE for compounds **1j**

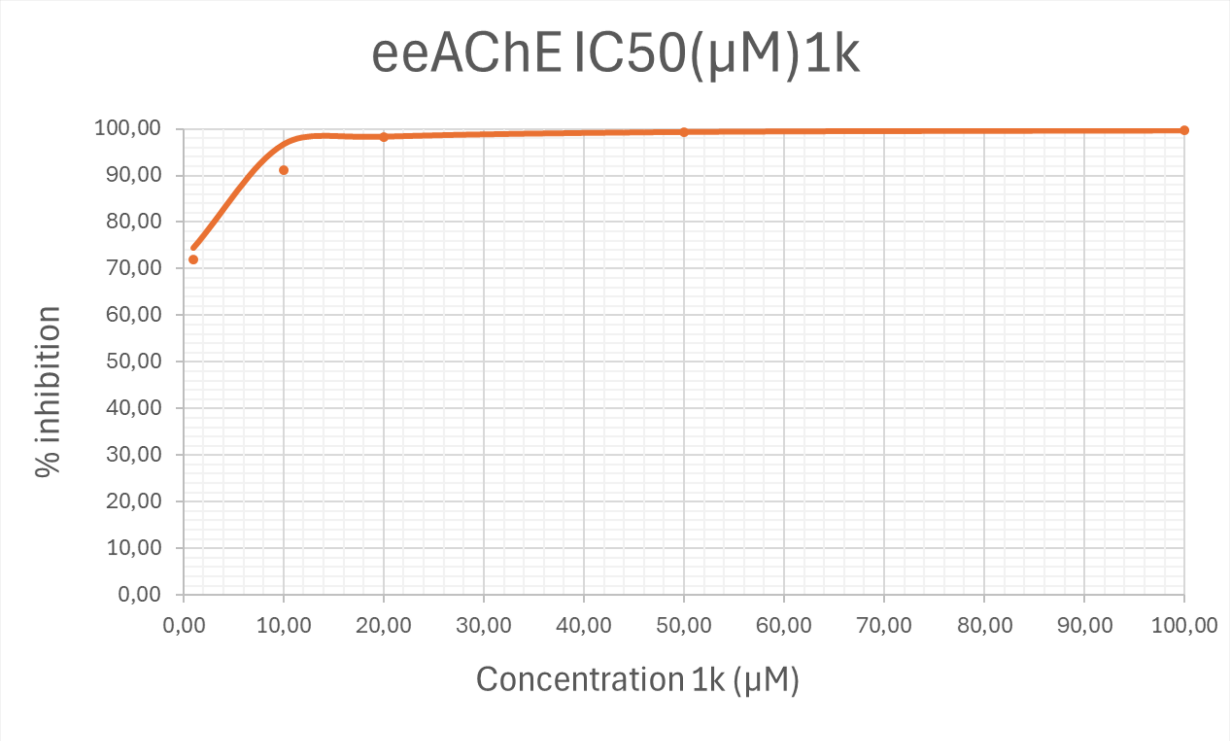

	r2 Coef Det	DF Adj r2	Fit Std Err	F-Statistic	
	0.999307	0.99723	0.566429	481.011509	
	Value	Std Error	t-Value	95% Confidence Limits	
a0	99.625373	0.472941	210.650633	93.616181	105.634564
a1	1945.944851	48350.008152	0.040247	-612389.27459	616281.164296
a2	-0.290914	359014.055497	-8.1031e-07	-4561632.9978	4561632.41601
a3	21.695581	26801016.9894	8.0951e-07	-340533708.96	340533752.355

Fig. S228. Dose–response curves on AChE for compounds **1k**

Dose–response curves on BACE-1 for compounds 1a-k

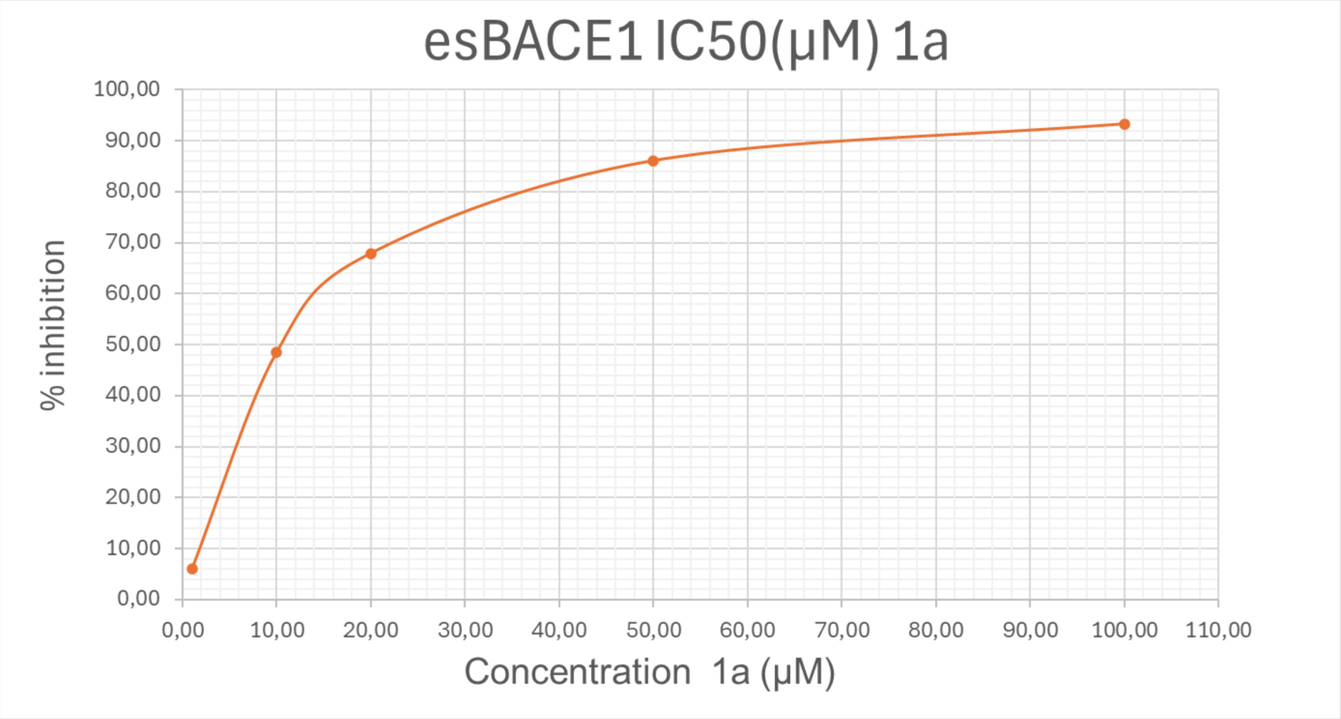

the regression formula: a0+(a1-a0)/(1+(x/a2)*a3)

r2 Coef Det	DF Adj r2	Fit Std Err	F-Statistic
0.999661	0.998646	1.288188	984.370692

	Value	Std Error	t-Value	95% Confidence Limits	
a0	103.999333	3.554317	29.260003	58.838177	149.16049
a1	-3.617003	3.43064	-1.054323	-47.20672	39.972714
a2	6.265937	1839.587768	0.003406	-23367.536844	23380.068717
a3	0.605339	177.731555	0.003406	-2257.651857	2258.862535

Fig. S229. Dose–response curves on BACE-1 for compounds **1a**

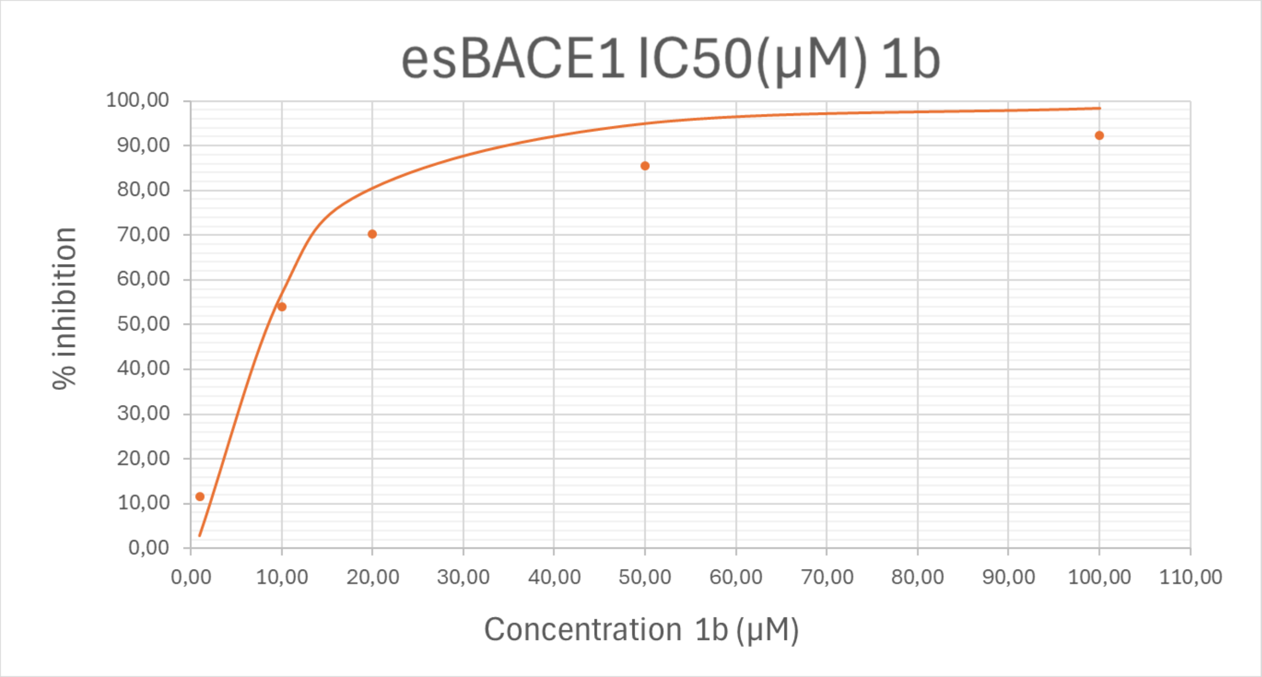

r2 Coef Det	DF Adj r2	Fit Std Err	F-Statistic
0.996584	0.986334	4.588023	97.234954

	Value	Std Error	t-Value	95% Confidence Limits	
a0	107.97474	10.072775	10.719463	-20.009941	235.959422
a1	-13.095175	25.42237	-0.515104	-336.111813	309.921462
a2	2.453141	3156.809347	0.000777	-40107.967453	40112.873734
a3	0.364664	469.446539	0.000777	-5964.423212	5965.152539

Fig. S230. Dose–response curves on BACE-1 for compounds **1b**

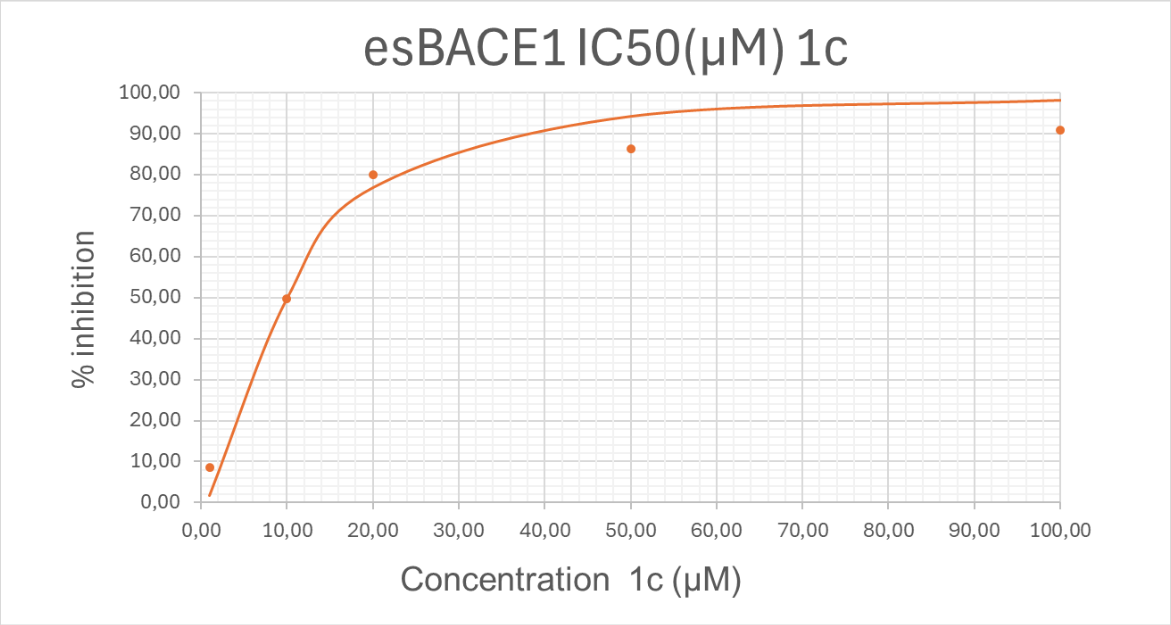

r2 Coef Det	DF Adj r2	Fit Std Err	F-Statistic
0.994103	0.976413	6.112846	56.195604

	Value	Std Error	t-Value	95% Confidence Limits	
a0	110.807914	15.449352	7.172334	-85.49156	307.107388
a1	-11.06213	20.281274	-0.545436	-268.756001	246.631741
a2	3.968829	5356.855308	0.000741	-68060.236474	68068.174133
a3	0.444559	600.14219	0.000741	-7624.962307	7625.851424

Fig. S231. Dose–response curves on BACE-1 for compounds **1c**

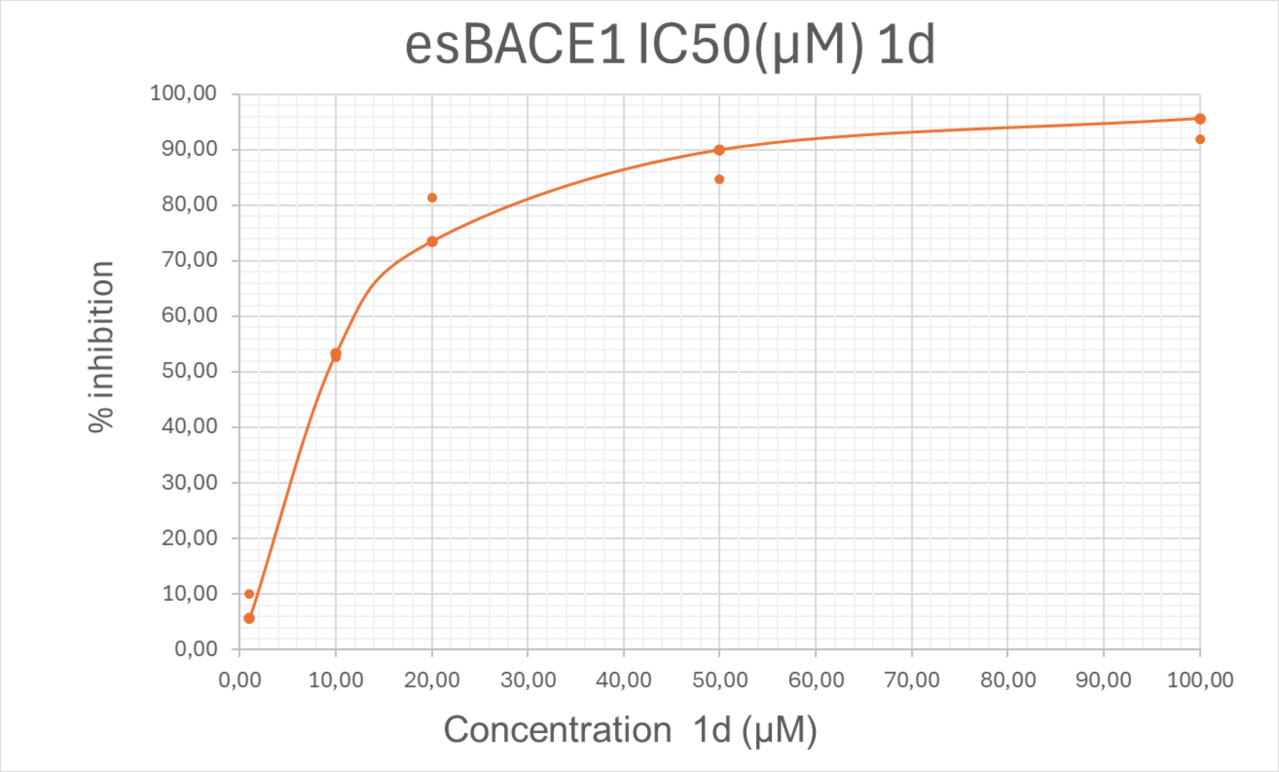

r2 Coef Det	DF Adj r2	Fit Std Err	F-Statistic
0.999222	0.996887	2.027318	427.948802

	Value	Std Error	t-Value	95% Confidence Limits	
a0	105.111282	4.928208	21.328499	42.493468	167.729097
a1	-6.482195	7.577119	-0.855496	-102.757077	89.792688
a2	2.615604	1347.000221	0.001942	-17112.36965	17117.600859
a3	0.314753	162.130516	0.001941	-2059.715639	2060.345144

Fig. S232. Dose–response curves on BACE-1 for compounds **1d**

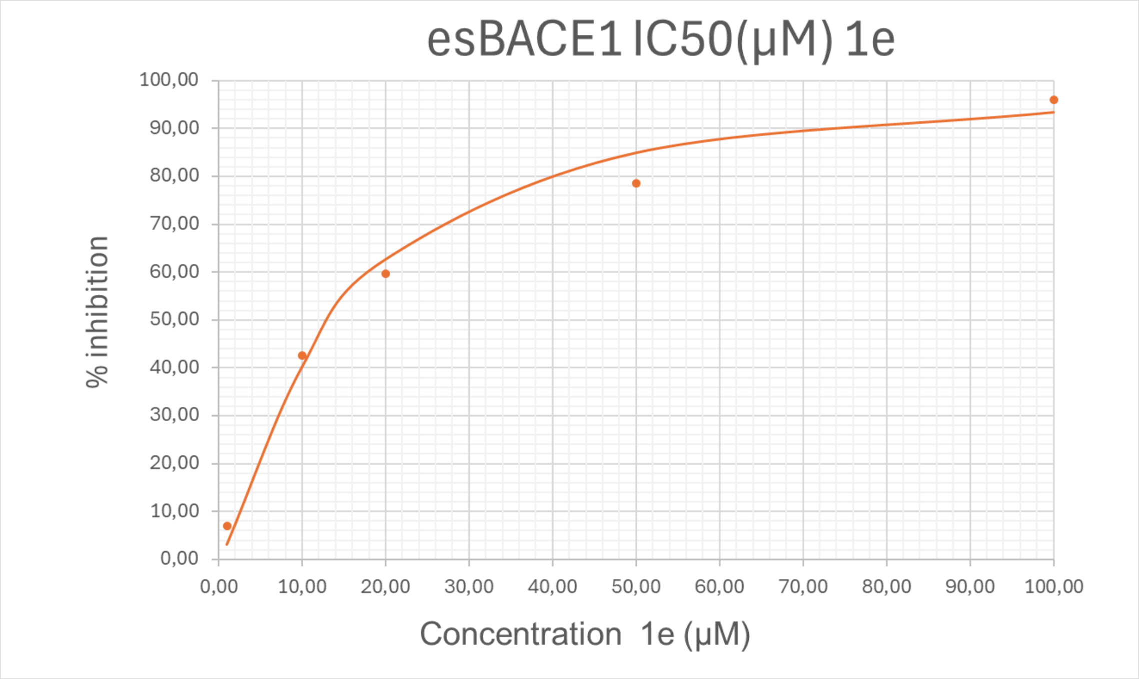

r2 Coef Det	DF Adj r2	Fit Std Err	F-Statistic
0.998465	0.99386	2.856993	216.836359

	Value	Std Error	t-Value	95% Confidence Limits	
a0	108.840992	9.934836	10.955489	-17.391038	235.073022
a1	-4.679188	5.323294	-0.879002	-72.31697	62.958593
a2	3.522346	1898.826403	0.001855	-24122.966556	24130.011247
a3	0.245393	132.265931	0.001855	-1680.325567	1680.816353

Fig. S233. Dose–response curves on BACE-1 for compounds **1e**

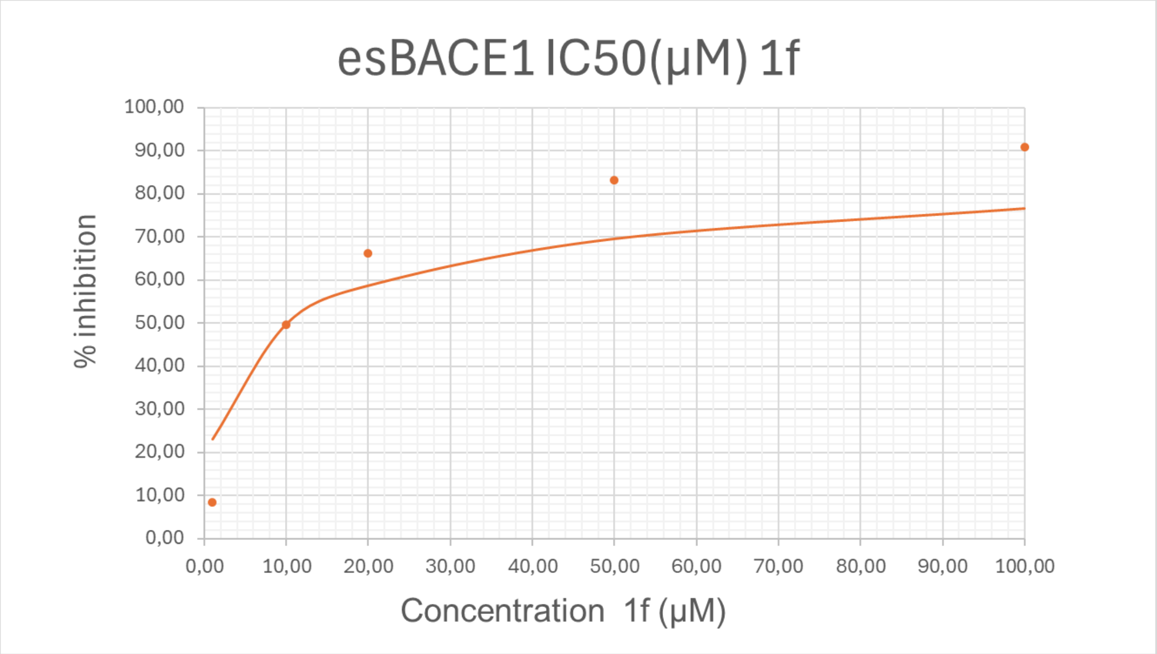

r2 Coef Det	DF Adj r2	Fit Std Err	F-Statistic
0.99787	0.991481	1.926535	156.18771

	Value	Std Error	t-Value	95% Confidence Limits	
a0	81.969975	5.418447	15.127947	13.123187	150.816763
a1	17.821774	4.927788	3.616587	-44.790708	80.434256
a2	111.349385	80678.32772	0.00138	-1024987.5090	1025210.20786
a3	10.437525	7562.904207	0.00138	-96083.825813	96104.700862

Fig. S234. Dose–response curves on BACE-1 for compounds **1f**

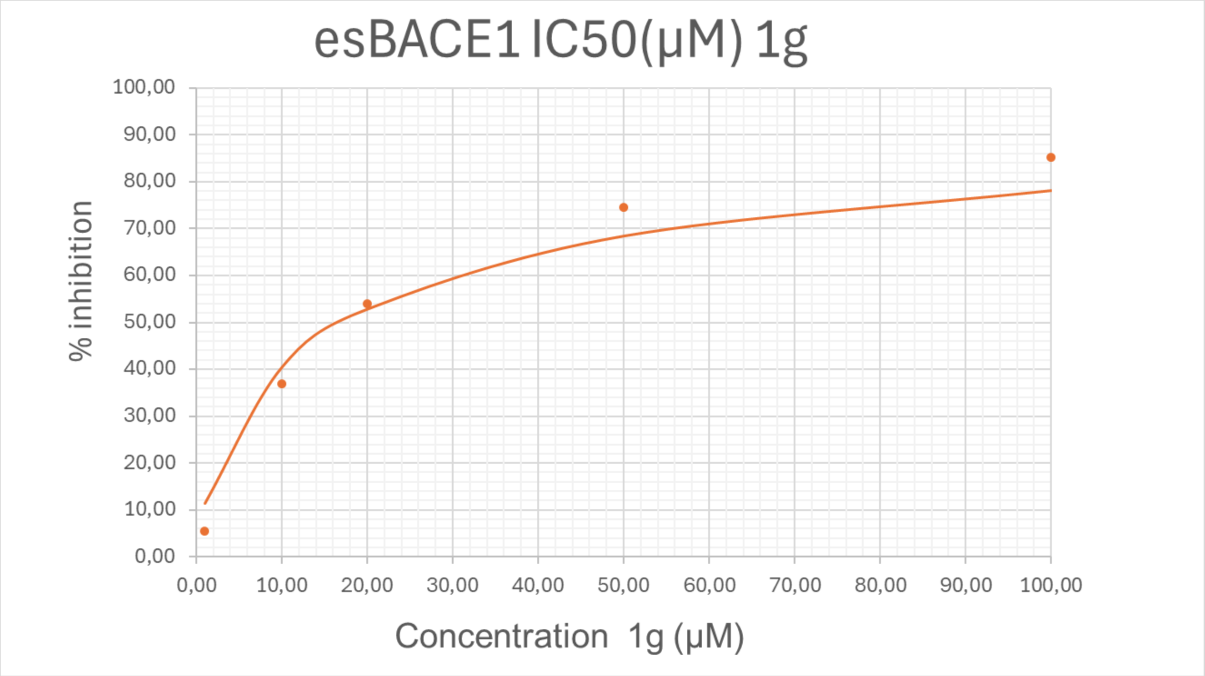

r2 Coef Det	DF Adj r2	Fit Std Err	F-Statistic
0.998971	0.995885	1.670649	323.694738

	Value	Std Error	t-Value	95% Confidence Limits	
a0	87.692803	5.880602	14.912214	12.973866	162.41174
a1	6.506431	3.073594	2.116881	-32.546652	45.559513
a2	15.219285	6681.161988	0.002278	-84875.627131	84906.065701

Fig. S235. Dose–response curves on BACE-1 for compounds **1g**

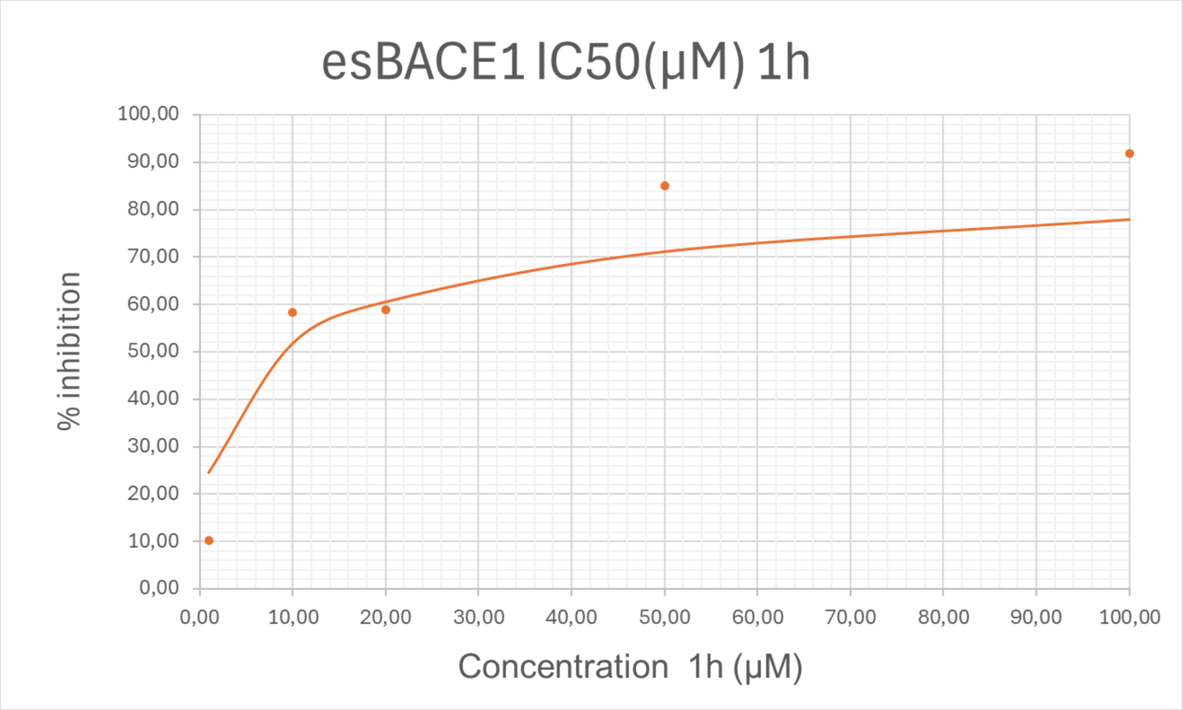

r2 Coef Det	DF Adj r2	Fit Std Err	F-Statistic
0.997953	0.991812	1.889416	162.504478

	Value	Std Error	t-Value	95% Confidence Limits	
a0	82.969383	5.145642	16.124203	17.58885	148.349916
a1	18.859087	5.179367	3.641195	-46.949951	84.668126
a2	727.932907	532477.840037	0.001367	-6764935.6772	6766391.54309
a3	71.81232	52534.781694	0.001367	-667435.14112	667578.765761

Fig. S236. Dose–response curves on BACE-1 for compounds **1h**

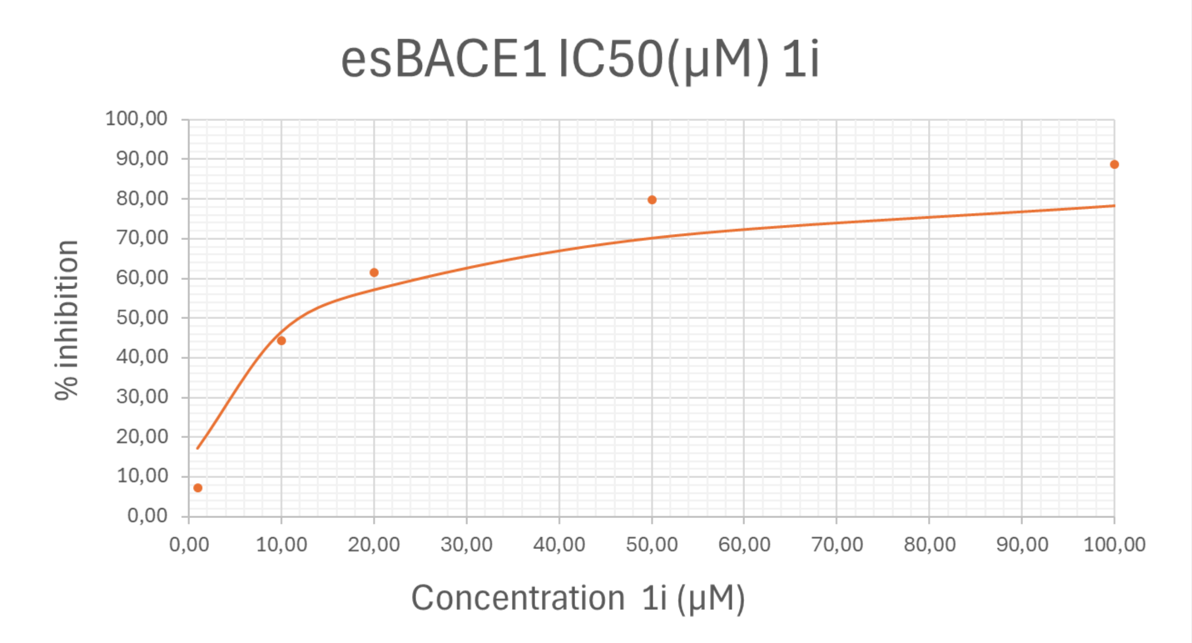

r2 Coef Det	DF Adj r2	Fit Std Err	F-Statistic
0.998464	0.993854	1.868496	216.623491

	Value	Std Error	t-Value	95% Confidence Limits	
a0	85.185519	5.62302	15.149424	13.73942	156.631619
a1	11.697649	4.220333	2.771736	-41.925899	65.321197
a2	10.934885	6384.158958	0.001713	-81106.190929	81128.060698
a3	0.926119	540.68862	0.001713	-6869.063665	6870.915903

Fig. S237. Dose–response curves on BACE-1 for compounds **1i**

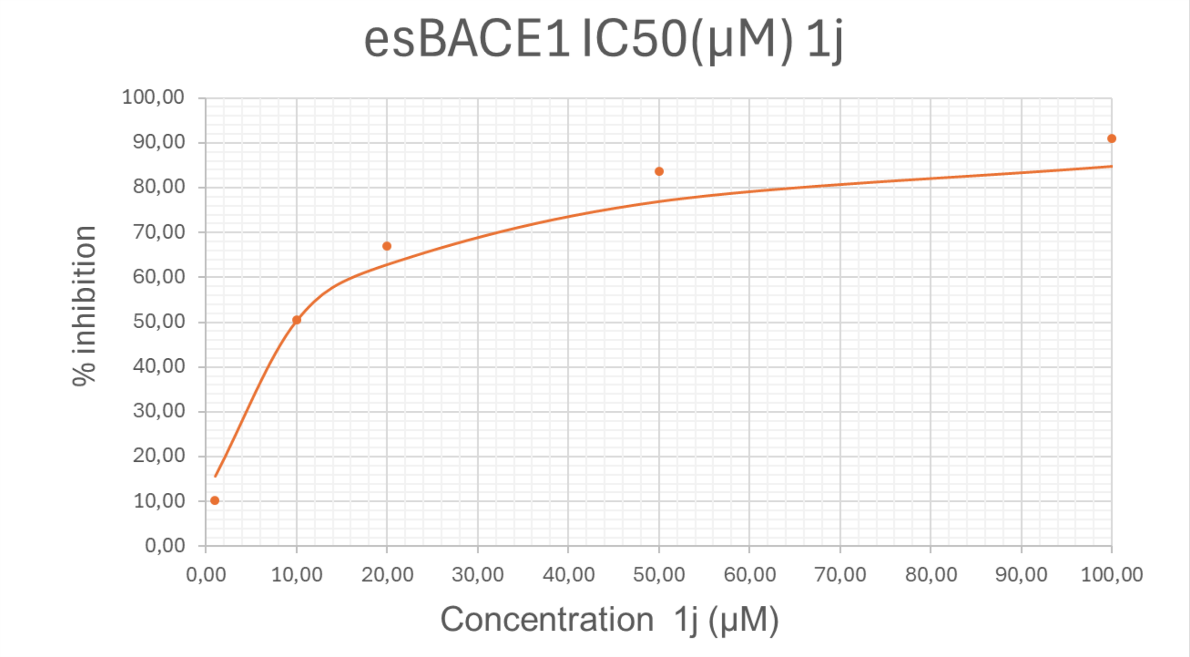

r2 Coef Det	DF Adj r2	Fit Std Err	F-Statistic
0.999302	0.997208	1.436835	477.30141

	Value	Std Error	t-Value	95% Confidence Limits	
a0	91.8451	3.942352	23.297035	41.75358	141.93662
a1	8.274275	3.873647	2.136042	-40.944291	57.492841
a2	51.792406	21950.26033	0.00236	-278848.22255	278951.807366
a3	5.048469	2139.77136	0.002359	-27182.887139	27192.984077

Fig. S238. Dose–response curves on BACE-1 for compounds **1j**

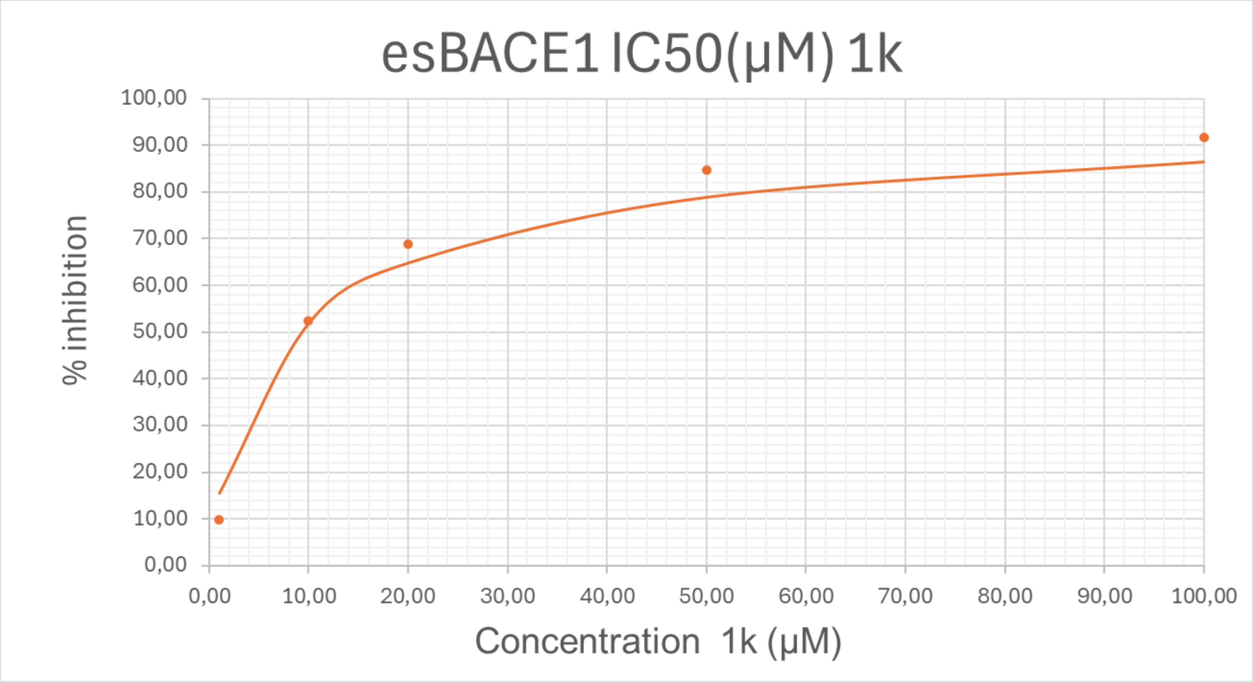

r2 Coef Det	DF Adj r2	Fit Std Err	F-Statistic
0.999473	0.997894	1.281727	632.777711

	Value	Std Error	t-Value	95% Confidence Limits	
a0	93.322153	3.388525	27.540643	50.267555	136.376751
a1	7.552373	3.77005	2.003255	-40.349889	55.454636
a2	11.32569	4326.283638	0.002618	-54958.435634	54981.087015
a3	1.173388	448.275535	0.002618	-5694.615703	5696.96248

Fig. S239. Dose–response curves on BACE-1 for compounds **1k**

Re-docking poses of AChE and BACE1 ligands aligned with their respective crystal structures

Re-docking poses for (A) human acetylcholinesterase (AChE; PDB ID: 4EY7) and (B) human beta-secretase 1 (BACE1; PDB ID: 3UQW) ligands. The poses correspond to the top-scoring ChemPLP conformations, with heavy-atom RMSD values of 0.974 Å (AChE) and 1.613 Å (BACE1) compared with the corresponding crystal structure ligands. Protein–ligand interactions for the docking poses were identified using PLIP. Hydrophobic interactions are shown as gray dashed lines, hydrogen bonds as blue dashed lines, and π–π stacking interactions as magenta dashed lines. The crystal structure ligand is shown as brown sticks, and the corresponding re-docking pose is shown in green sticks. Interacting amino acid residues identified by PLIP are presented as gray sticks, and the protein backbone as a semi-transparent gray cartoon representation. Active-site residues are shown in cyan. For better visibility, hydrogen atoms are shown only for the ligand and for protein residues when involved in PLIP-recognized interactions. Hydrogen atoms are shown in pale green. Backbone atoms are shown only when directly involved in a PLIP-recognized hydrogen bond. In stick representations, carbon atoms follow the colour scheme described above, whereas heteroatoms are shown using standard atom colours. Residue numbering follows the reference crystal structure. The ChemPLP score (obtained from PLANTS) corresponding to the pose shown in each panel, together with the individual interaction distances identified for that pose, are summarized in the accompanying table. Distances reported for hydrogen bonds correspond to H···A distances generated by PLIP.

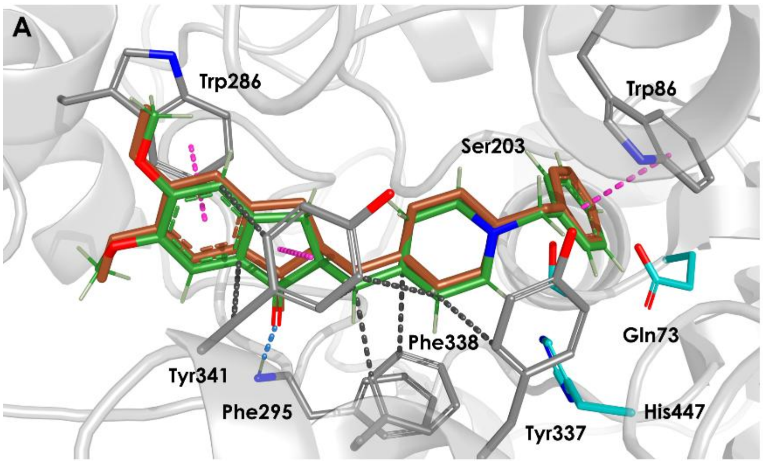

Interaction Type	Protein Residue	Distance [Å]
Hydrophobic	TYR337	3.47
Hydrophobic	PHE338	3.35
Hydrophobic	PHE338	3.49
Hydrophobic	TYR341	3.63
Hydrophobic	TYR341	3.76
Hydrophobic	TYR341	3.59
Hydrophobic	TYR341	3.99
H-bond	PHE295	1.63
Pi-stacking	TRP86	3.96
Pi-stacking	TRP286	4.05
Pi-stacking	TYR341	4.23
ChemPLP score	-128.883	
RMSD	0.974 Å	

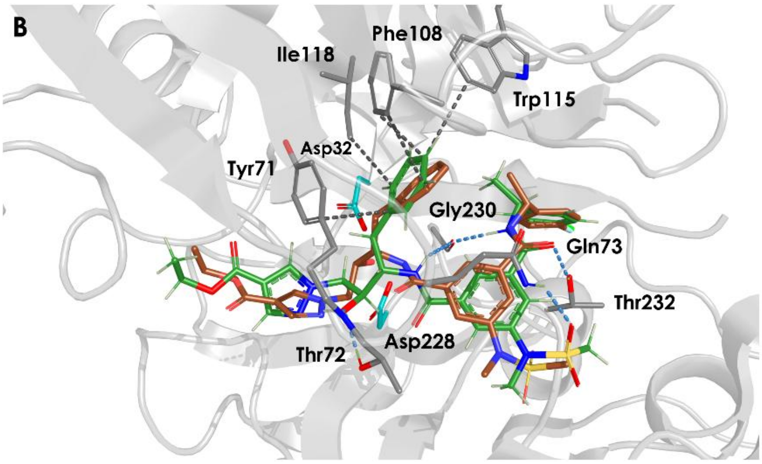

Interaction Type	Protein Residue	Distance [Å]
Hydrophobic	TYR71	3.67
Hydrophobic	PHE108	3.16
Hydrophobic	PHE108	3.67
Hydrophobic	TRP115	3.97
Hydrophobic	ILE118	3.45
H-bond	THR72	1.94
H-bond	THR72	1.91
H-bond	GLN73	2.23
H-bond	GLY230	2.09
H-bond	GLY230	3.05
H-bond	THR232	2.09
ChemPLP score	-127.032	
RMSD	1.613 Å	

Fig. S240. Re-docking poses of AChE and BACE1 ligands aligned with their respective crystal structures.

Docking poses of quercetin in the BACE1 binding site

Representative docking pose and protein–ligand interaction pattern for quercetin in the binding cavity of human beta-secretase 1 (BACE1; PDB ID: 3UQW), based on the docking results. The pose shown correspond to the top-scoring ChemPLP conformations selected from the dominant docking cluster, as described in the Materials and Methods section. Protein–ligand interactions were identified using PLIP. Hydrophobic interactions are shown as gray dashed lines, hydrogen bonds as blue dashed lines, and π–π stacking interactions as magenta dashed lines. The ligand is shown as green sticks, interacting amino acid residues identified by PLIP as gray sticks, and the protein backbone as a semi-transparent gray cartoon representation. Active-site residues are shown in cyan. For better visibility, hydrogen atoms are shown only for the ligand and for protein residues when involved in PLIP-recognized interactions. Hydrogen atoms are shown in pale green. Backbone atoms are shown only when directly involved in a PLIP-recognized hydrogen bond. In stick representations, carbon atoms follow the colour scheme described above, whereas heteroatoms are shown using standard atom colours. Residue numbering follows the reference crystal structure. The ChemPLP score (obtained from PLANTS) corresponding to the pose shown in each panel, together with the individual interaction distances identified for that pose, are summarized in the accompanying table. Distances reported for hydrogen bonds correspond to H⋯A distances generated by PLIP.

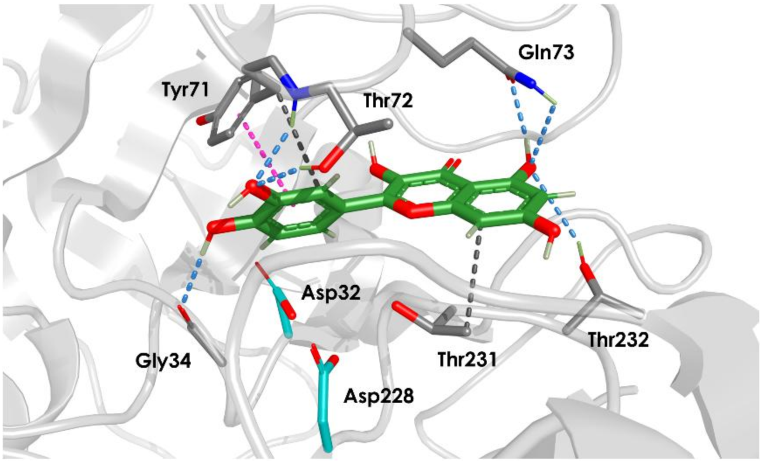

Interaction Type	Protein Residue	Distance [Å]
Hydrophobic	TYR71	3.82
Hydrophobic	THR231	3.46
H-bond	GLY34	2.10
H-bond	THR72	2.22
H-bond	THR72	2.10
H-bond	GLN73	2.78
H-bond	GLN73	2.10
H-bond	THR232	3.03
Pi-stacking	TYR71	5.01
ChemPLP score	-73.1485	

Fig. S241. Docking pose of quercetin in the BACE1 binding cavity.

Docking poses of compounds 1a–1k in the AChE binding site

Representative docking poses and protein–ligand interaction patterns for compounds **1A–1K** in the active site of human acetylcholinesterase (AChE; PDB ID: 4EY7), based on the docking results. The poses shown correspond to the top-scoring ChemPLP conformations selected from the dominant docking cluster, as described in the Materials and Methods section. Protein–ligand interactions were identified using PLIP, except for the −N=C=S···O interaction, which was measured separately. Hydrophobic interactions are shown as gray dashed lines, hydrogen bonds as blue dashed lines, π–π stacking interactions as magenta dashed lines, and −N=C=S···O contacts as yellow dashed lines. The ligand is shown as green sticks, interacting amino acid residues identified by PLIP as gray sticks, and the protein backbone as a semi-transparent gray cartoon representation. Active-site residues and the co-localized Glu202 residue are shown in cyan. For better visibility, hydrogen atoms are shown only for the ligand and for protein residues when involved in PLIP-recognized interactions. Hydrogen atoms are shown in pale green. In stick representations, carbon atoms follow the colour scheme described above, whereas heteroatoms are shown using standard atom colours. Residue numbering follows the reference crystal structure. The ChemPLP score (obtained from PLANTS) corresponding to the pose shown in each panel, together with the individual interaction distances identified for that pose, are summarized in the accompanying table. Distances reported for hydrogen bonds correspond to H···A distances generated by PLIP.

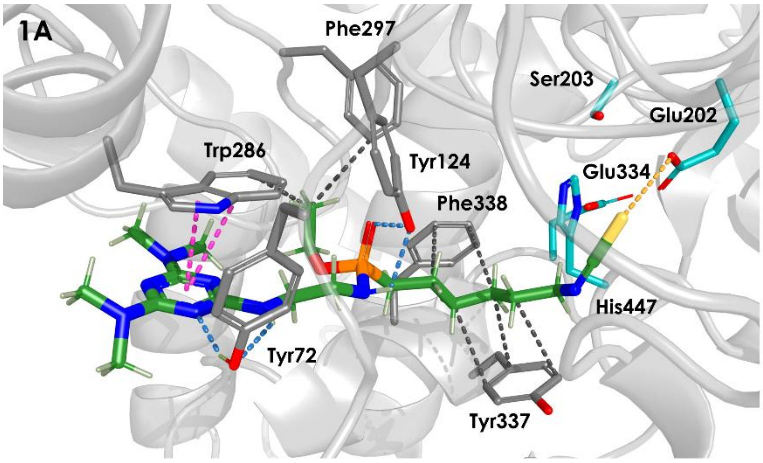

Interaction Type	Protein Residue	Distance [Å]
Hydrophobic	TRP286	3.62
Hydrophobic	PHE297	3.39
Hydrophobic	TYR337	3.51
Hydrophobic	TYR337	3.71
Hydrophobic	TYR337	3.52
Hydrophobic	PHE338	3.48
Hydrophobic	PHE338	3.50
H-bond	TYR72	2.10
H-bond	TYR72	2.64
H-bond	TYR124	2.42
H-bond	TYR124	2.05
Pi-stacking	TRP286	3.63
Pi-stacking	TRP286	4.07
–N=C=S···O	GLU202	3.73
ChemPLP score	-120.021	

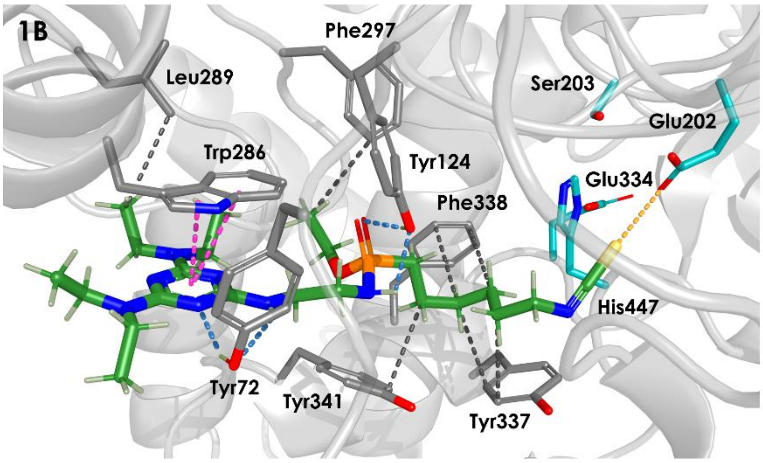

Interaction Type	Protein Residue	Distance [Å]
Hydrophobic	LEU289	3.76
Hydrophobic	PHE297	3.70
Hydrophobic	TYR337	3.51
Hydrophobic	TYR337	3.70
Hydrophobic	PHE338	3.82
Hydrophobic	PHE338	3.52
Hydrophobic	PHE338	3.97
Hydrophobic	TYR341	3.42
H-bond	TYR72	2.35
H-bond	TYR72	2.81
H-bond	TYR124	2.48
H-bond	TYR124	2.03
Pi-stacking	TRP286	3.59
Pi-stacking	TRP286	3.86
–N=C=S···O	GLU202	3.71
ChemPLP score	-121.503	

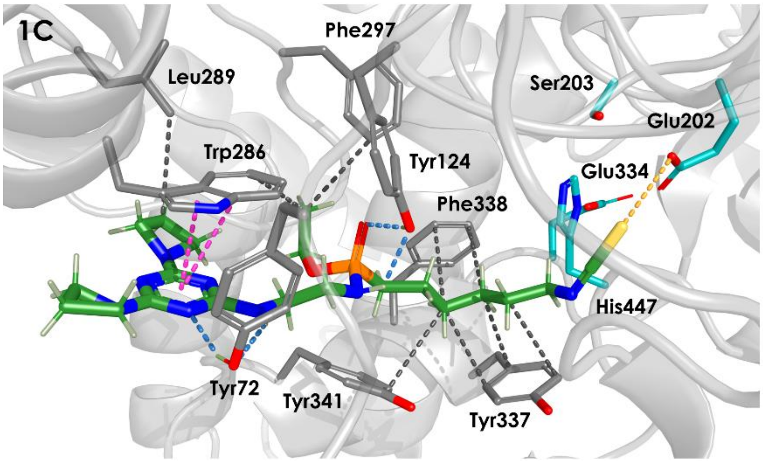

Interaction Type	Protein Residue	Distance [Å]
Hydrophobic	TRP286	3.61
Hydrophobic	LEU289	3.92
Hydrophobic	PHE297	3.60
Hydrophobic	TYR337	3.55
Hydrophobic	TYR337	3.52
Hydrophobic	TYR337	3.68
Hydrophobic	PHE338	3.60
Hydrophobic	PHE338	3.38
Hydrophobic	TYR341	3.70
H-bond	TYR72	2.19
H-bond	TYR72	2.44
H-bond	TYR124	2.37
H-bond	TYR124	2.10
Pi-stacking	TRP286	3.61
Pi-stacking	TRP286	4.11
–N=C=S···O	GLU202	3.72
ChemPLP score	-127.44	

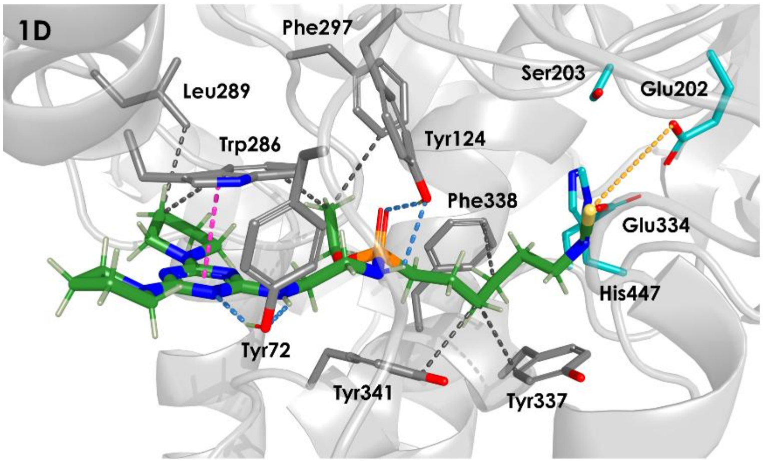

Interaction Type	Protein Residue	Distance [Å]
Hydrophobic	TRP286	3.97
Hydrophobic	TRP286	3.71
Hydrophobic	LEU289	3.50
Hydrophobic	PHE297	3.45
Hydrophobic	TYR337	3.42
Hydrophobic	PHE338	3.35
Hydrophobic	TYR341	3.47
H-bond	TYR72	2.15
H-bond	TYR72	2.30
H-bond	TYR124	2.52
H-bond	TYR124	1.89
Pi-stacking	TRP286	3.63
−N=C=S···O	GLU202	6.04
ChemPLP score	-122.869	

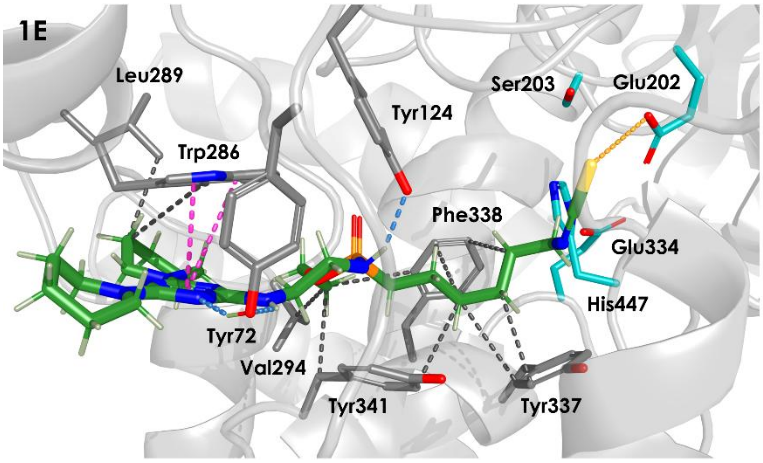

Interaction Type	Protein Residue	Distance [Å]
Hydrophobic	TRP286	3.97
Hydrophobic	LEU289	3.55
Hydrophobic	VAL294	3.53
Hydrophobic	TYR337	3.70
Hydrophobic	TYR337	3.45
Hydrophobic	PHE338	3.88
Hydrophobic	PHE338	3.70
Hydrophobic	PHE338	3.65
Hydrophobic	TYR341	3.54
Hydrophobic	TYR341	3.74
H-bond	TYR72	2.09
H-bond	TYR72	2.35
H-bond	TYR124	2.10
Pi-stacking	TRP286	3.69
Pi-stacking	TRP286	4.25
−N=C=S···O	GLU202	5.08
ChemPLP score	-129.058	

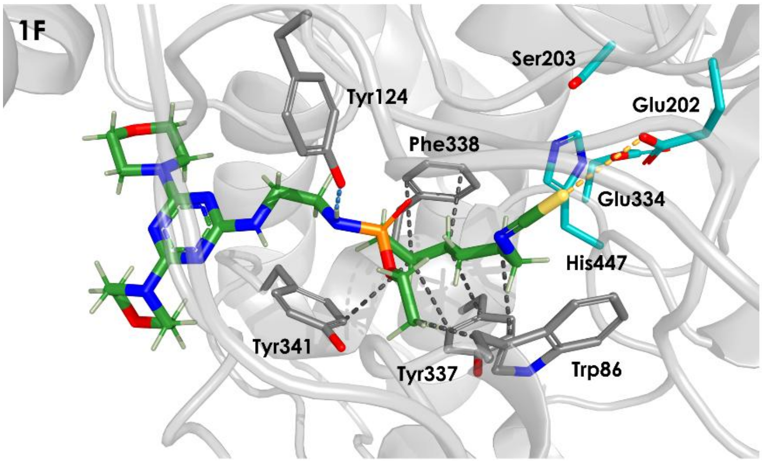

Interaction Type	Protein Residue	Distance [Å]
Hydrophobic	TRP86	3.99
Hydrophobic	TYR337	3.15
Hydrophobic	TYR337	3.33
Hydrophobic	TYR337	3.53
Hydrophobic	PHE338	3.71
Hydrophobic	PHE338	3.33
Hydrophobic	TYR341	3.47
H-bond	TYR124	1.64
S···O	GLU202	3.88
ChemPLP score	-114,74	

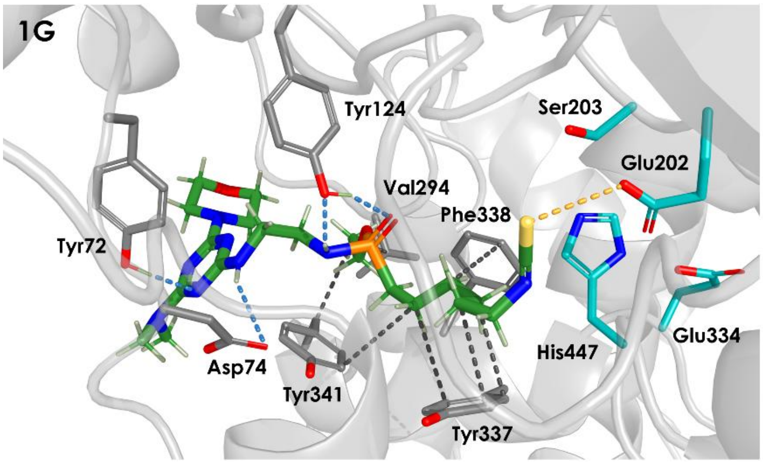

Interaction Type	Protein Residue	Distance [Å]
Hydrophobic	VAL294	3.52
Hydrophobic	TYR337	3.71
Hydrophobic	TYR337	3.72
Hydrophobic	TYR337	3.54
Hydrophobic	PHE338	3.31
Hydrophobic	TYR341	3.53
Hydrophobic	TYR341	3.91
H-bond	TYR72	2.13
H-bond	ASP74	3.05
H-bond	TYR124	2.05
H-bond	TYR124	2.10
−N=C=S···O	GLU202	3.40
ChemPLP score	-122.292	

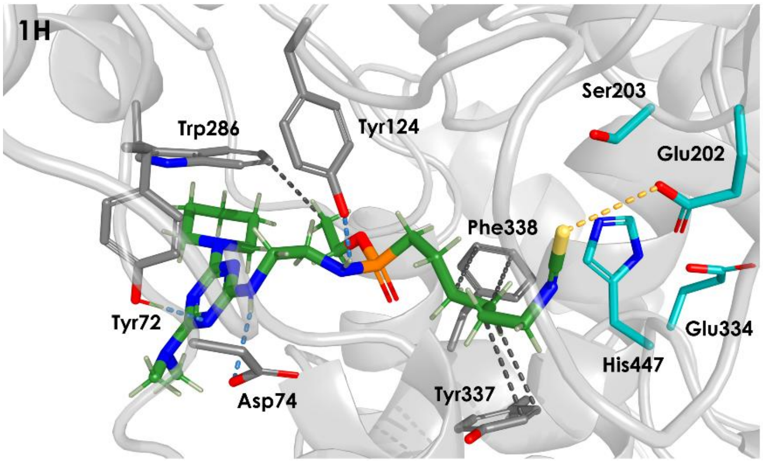

Interaction Type	Protein Residue	Distance [Å]
Hydrophobic	TRP286	3.80
Hydrophobic	TYR337	3.80
Hydrophobic	TYR337	3.91
Hydrophobic	PHE338	3.51
Hydrophobic	PHE338	3.93
H-bond	TYR72	2.10
H-bond	ASP74	2.57
H-bond	TYR124	1.99
−N=C=S···O	GLU202	3.56
ChemPLP score	-118.52	

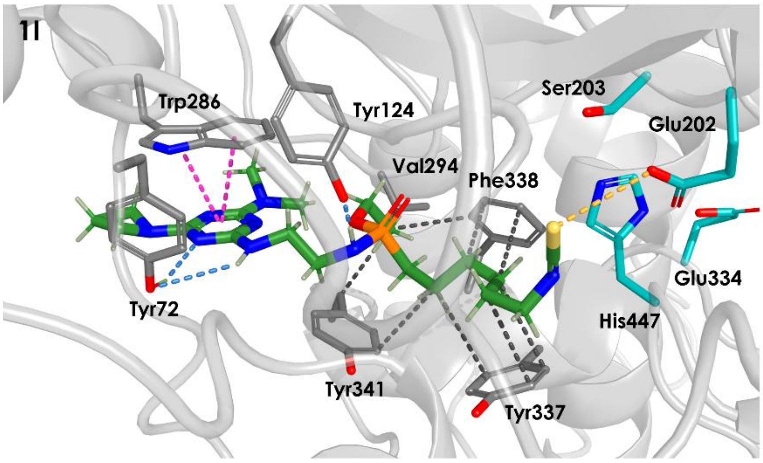

Interaction Type	Protein Residue	Distance [Å]
Hydrophobic	VAL294	3.91
Hydrophobic	TYR337	3.80
Hydrophobic	TYR337	3.70
Hydrophobic	TYR337	3.71
Hydrophobic	PHE338	3.21
Hydrophobic	PHE338	3.31
Hydrophobic	PHE338	3.92
Hydrophobic	TYR341	3.42
Hydrophobic	TYR341	3.59
H-bond	TYR72	2.18
H-bond	TYR72	3.16
H-bond	TYR124	1.43
Pi-stacking	TRP286	3.95
Pi-stacking	TRP286	3.79
−N=C=S···O	GLU202	3.53
ChemPLP score	-122.951	

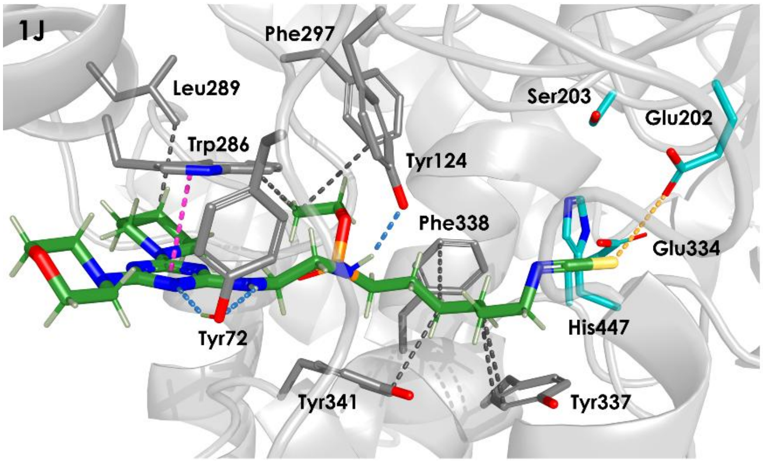

Interaction Type	Protein Residue	Distance [Å]
Hydrophobic	TRP286	3.50
Hydrophobic	LEU289	3.52
Hydrophobic	PHE297	3.70
Hydrophobic	TYR337	3.61
Hydrophobic	TYR337	3.62
Hydrophobic	PHE338	3.53
Hydrophobic	TYR341	3.66
H-bond	TYR72	2.08
H-bond	TYR72	2.45
H-bond	TYR124	2.28
Pi-stacking	TRP286	3.69
–N=C=S···O	GLU202	4.30
ChemPLP score	-121.005	

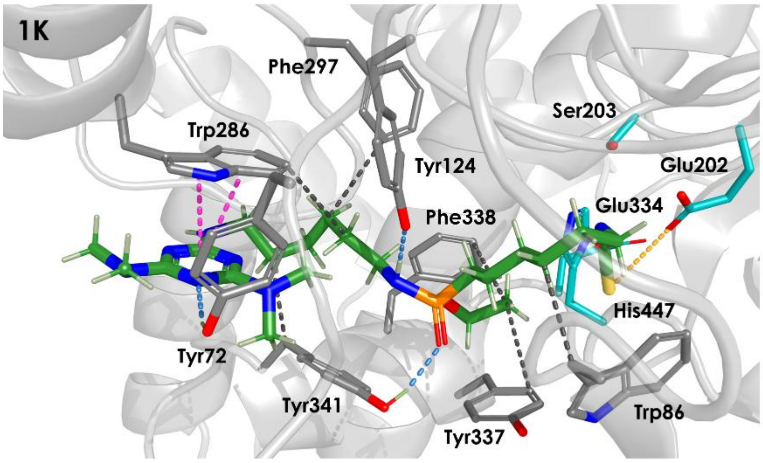

Interaction Type	Protein Residue	Distance [Å]
Hydrophobic	TRP86	3.52
Hydrophobic	TRP286	3.80
Hydrophobic	PHE297	3.52
Hydrophobic	TYR337	3.68
Hydrophobic	PHE338	3.68
Hydrophobic	TYR341	3.37
H-bond	TYR72	2.31
H-bond	TYR124	1.95
H-bond	TYR341	2.09
Pi-stacking	TRP286	3.91
Pi-stacking	TRP286	3.84
–N=C=S···O	GLU202	4.29
ChemPLP score	-129.411	

Fig. S242. Representative docking poses and protein–ligand interaction patterns for compounds **1a–1k** in the active site of acetylcholinesterase (AChE; PDB ID: 4EY7).

Docking poses of compounds 1a–1k in the BACE1 binding site

Representative docking poses and protein–ligand interaction patterns for compounds **1a–1k** in the active site of human beta-secretase 1 (BACE1; PDB ID: 3UQW), based on the docking results. The poses shown correspond to the top-scoring ChemPLP conformations selected from the dominant docking cluster, as described in the Materials and Methods section. Protein–ligand interactions were identified using PLIP. Hydrophobic interactions are shown as gray dashed lines and hydrogen bonds as blue dashed lines. The ligand is shown as green sticks, interacting amino acid residues identified by PLIP as gray sticks, and the protein backbone as a semi-transparent gray cartoon representation. Active-site residues are shown in cyan. For better visibility, hydrogen atoms are shown only for the ligand and for protein residues when involved in PLIP-recognized interactions. Hydrogen atoms are shown in pale green. Backbone atoms are shown only when directly involved in a PLIP-recognized hydrogen bond. In stick representations, carbon atoms follow the colour scheme described above,

whereas heteroatoms are shown using standard atom colours. Residue numbering follows the reference crystal structure. The ChemPLP score (obtained from PLANTS) corresponding to the pose shown in each panel, together with the individual interaction distances identified for that pose, are summarized in the accompanying table. Distances reported for hydrogen bonds correspond to H···A distances generated by PLIP.

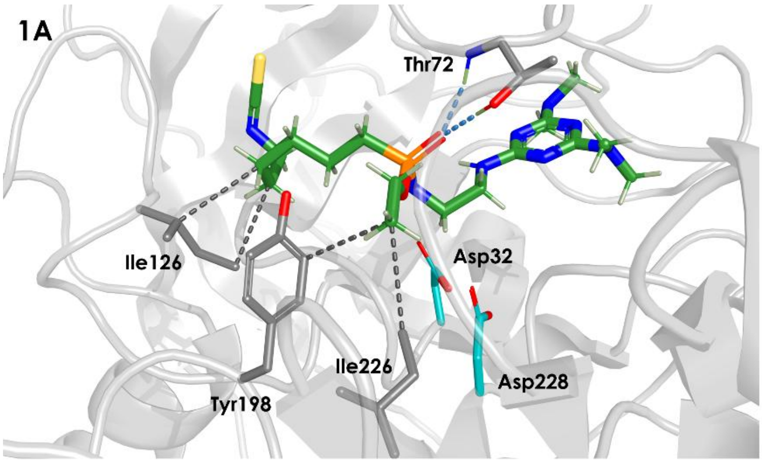

Interaction Type	Protein Residue	Distance [Å]
Hydrophobic	ILE126	3.59
Hydrophobic	ILE126	3.64
Hydrophobic	TYR198	3.56
Hydrophobic	ILE226	3.70
H-bond	THR72	2.03
H-bond	THR72	2.10
ChemPLP score	-98.2629	

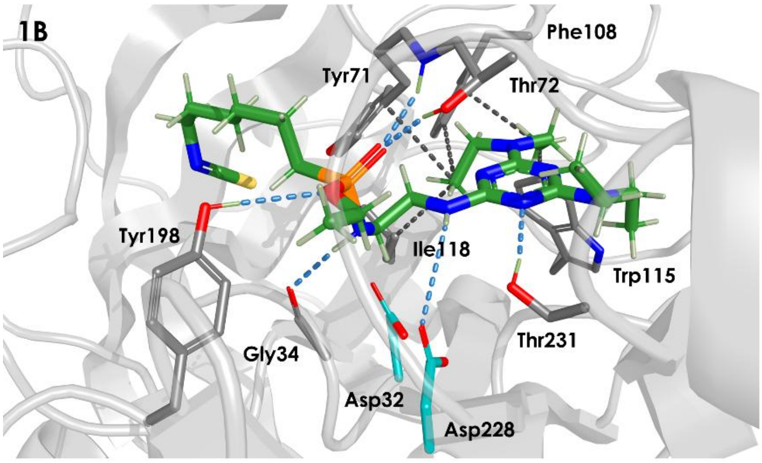

Interaction Type	Protein Residue	Distance [Å]
Hydrophobic	TYR71	3.91
Hydrophobic	PHE108	3.87
Hydrophobic	PHE108	3.98
Hydrophobic	TRP115	3.81
Hydrophobic	ILE118	3.77
H-bond	GLY34	2.18
H-bond	THR72	2.11
H-bond	THR72	2.10
H-bond	TYR198	2.66
H-bond	ASP228	3.35
H-bond	THR231	2.13
ChemPLP score	-103.635	

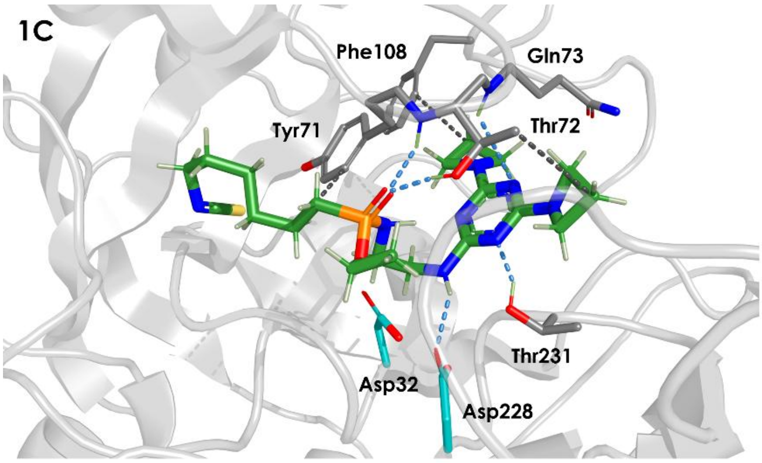

Interaction Type	Protein Residue	Distance [Å]
Hydrophobic	TYR71	3.64
Hydrophobic	THR72	3.93
Hydrophobic	PHE108	3.60
H-bond	THR72	2.12
H-bond	THR72	1.62
H-bond	GLN73	3.20
H-bond	ASP228	2.19
H-bond	THR231	1.66
ChemPLP score	-101,373	

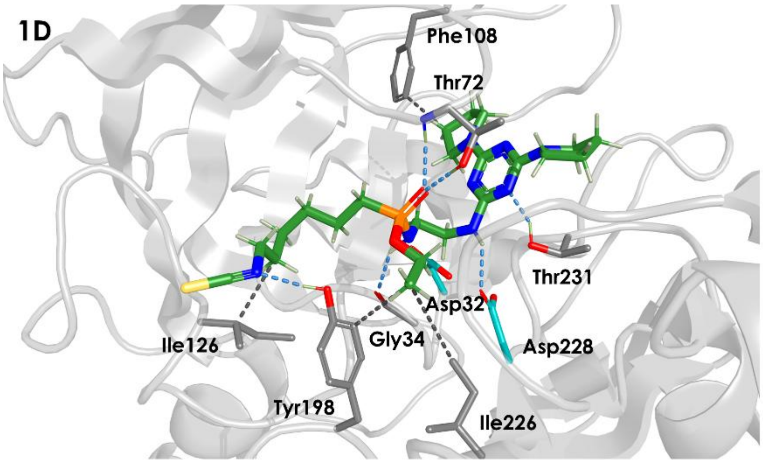

Interaction Type	Protein Residue	Distance [Å]
Hydrophobic	PIIE108	2.88
Hydrophobic	ILE126	3.29
Hydrophobic	TYR198	3.69
Hydrophobic	ILE226	3.98
H-bond	GLY34	2.21
H-bond	THR72	2.10
H-bond	THR72	1.76
H-bond	TYR198	2.04
II-bond	ASP228	2.11
H-bond	THR231	1.74
ChemPLP score	-108.588	

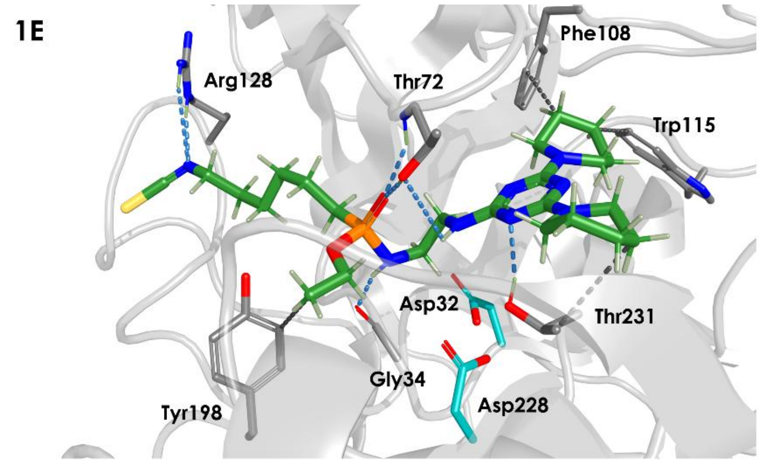

Interaction Type	Protein Residue	Distance [Å]
Hydrophobic	PHE108	3.45
Hydrophobic	TRP115	3.68
Hydrophobic	TYR198	3.97
Hydrophobic	THR321	3.86
H-bond	GLY34	2.10
H-bond	THR72	3.51
H-bond	THR72	2.10
H-bond	THR72	2.08
II-bond	ARG128	1.99
H-bond	ARG128	3.40
H-bond	THR231	2.09
ChemPLP score	-112.539	

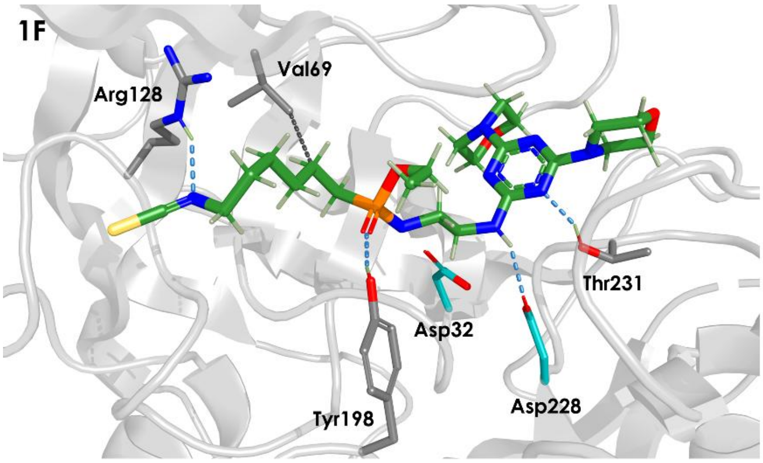

Interaction Type	Protein Residue	Distance [Å]
Hydrophobic	VAL69	3.90
H-bond	ARG128	2.10
H-bond	TYR198	1.72
H-bond	ASP228	2.12
H-bond	THR231	1.84
ChemPLP score	-112.769	

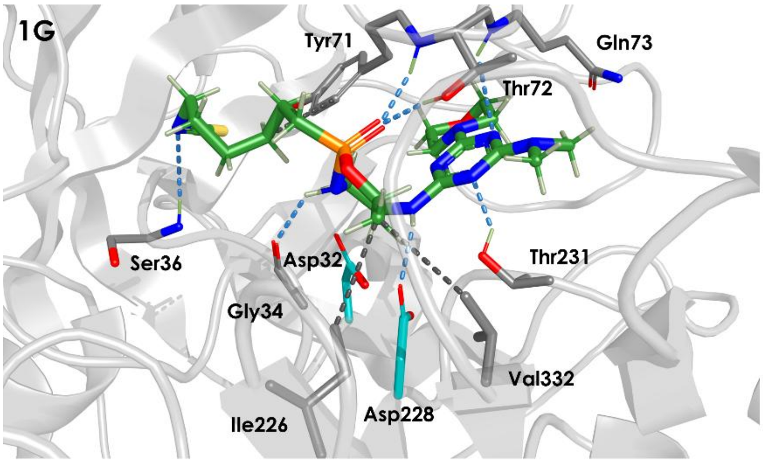

Interaction Type	Protein Residue	Distance [Å]
Hydrophobic	TYR71	3.47
Hydrophobic	ILE226	3.67
Hydrophobic	VAL332	3.76
H-bond	GLY34	2.16
H-bond	SER36	2.91
H-bond	THR72	2.06
H-bond	THR72	1.99
H-bond	GLN73	3.10
II-bond	ASP228	2.19
H-bond	THR231	1.96
ChemPLP score	-107.409	

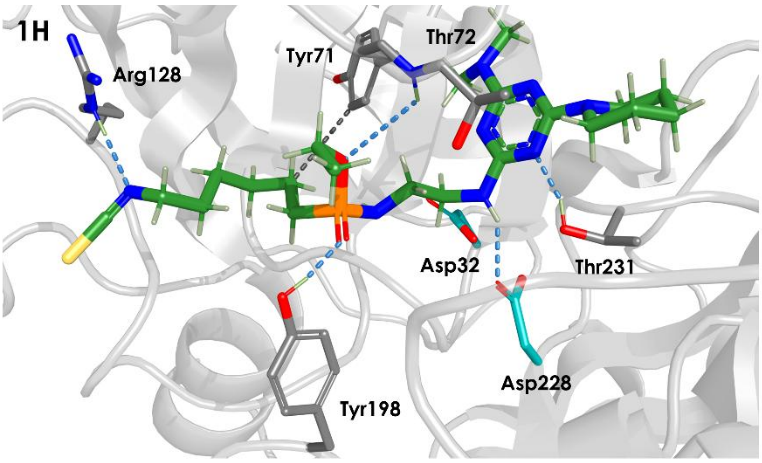

Interaction Type	Protein Residue	Distance [Å]
Hydrophobic	TYR71	3.82
H-bond	THR72	3.36
H-bond	ARG128	2.06
H-bond	TYR198	1.69
H-bond	ASP228	2.18
H-bond	THR231	1.83
ChemPLP score	-104.175	

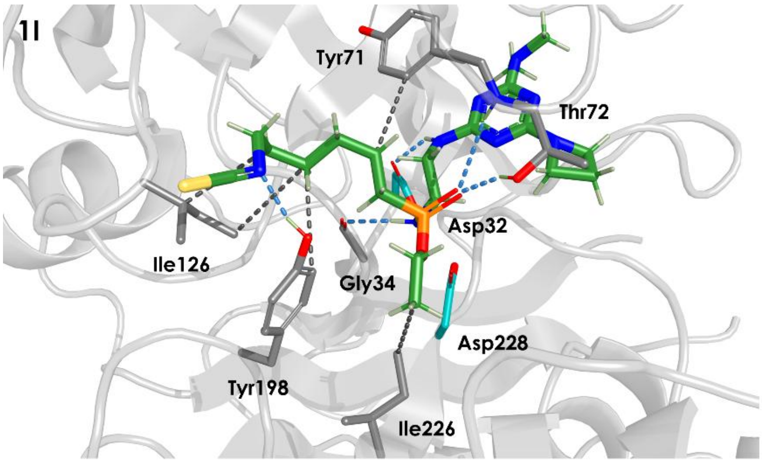

Interaction Type	Protein Residue	Distance [Å]
Hydrophobic	TYR71	3.52
Hydrophobic	ILE126	3.62
Hydrophobic	ILE126	3.98
Hydrophobic	TYR198	3.89
Hydrophobic	ILE226	3.57
H-bond	ASP32	2.01
H-bond	GLY34	1.96
H-bond	THR72	3.01
II-bond	TIIR72	2.11
H-bond	TYR198	1.93
ChemPLP score	-103.983	

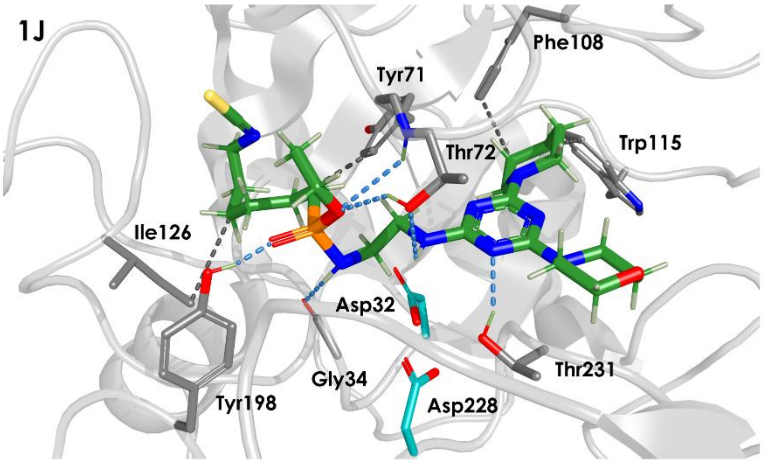

Interaction Type	Protein Residue	Distance [Å]
Hydrophobic	TYR71	3.46
Hydrophobic	PHE108	3.20
Hydrophobic	TRP115	3.61
Hydrophobic	ILE126	3.59
H-bond	GLY34	1.95
H-bond	THR72	3.32
H-bond	THR72	2.75
H-bond	THR72	2.57
II-bond	TYR198	1.50
H-bond	THR231	2.10
ChemPLP score	-109.777	

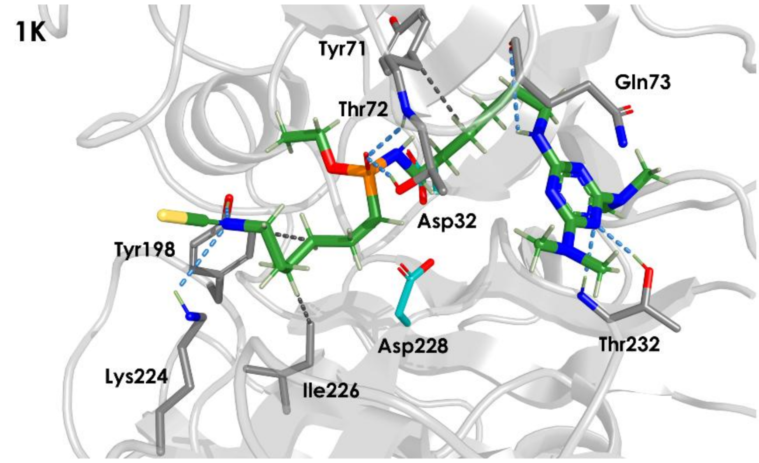

Interaction Type	Protein Residue	Distance [Å]
Hydrophobic	TYR71	3.97
Hydrophobic	TYR198	3.53
Hydrophobic	ILE226	3.63
II-bond	THR72	1.92
H-bond	THR72	1.77
H-bond	GLN73	3.40
H-bond	TYR198	1.94
H-bond	LYS224	3.13
H-bond	THR232	2.14
H-bond	THR232	2.10
ChemPLP score	-104.008	

Fig. S243. Representative docking poses and protein–ligand interaction patterns for compounds 1A–1K in the active site of human beta-secretase 1 (BACE1; PDB ID: 3UQW)
